# Targeted insertional mutagenesis libraries for deep domain insertion profiling

**DOI:** 10.1093/nar/gkz1110

**Published:** 2019-11-20

**Authors:** Willow Coyote-Maestas, David Nedrud, Steffan Okorafor, Yungui He, Daniel Schmidt

**Affiliations:** 1 Dept. of Biochemistry, Molecular Biology & Biophysics, University of Minnesota, Minneapolis, MN 55455, USA; 2 Dept. of Genetics, Cell Biology & Development, University of Minnesota, Minneapolis, MN 55455, USA; 3 Dept. of Neuroscience, University of Minnesota, Minneapolis, MN 55455, USA

## Abstract

Domain recombination is a key principle in protein evolution and protein engineering, but inserting a donor domain into every position of a target protein is not easily experimentally accessible. Most contemporary domain insertion profiling approaches rely on DNA transposons, which are constrained by sequence bias. Here, we establish **S**aturated **P**rogrammable **In**sertion **E**ngineering (SPINE), an unbiased, comprehensive, and targeted domain insertion library generation technique using oligo library synthesis and multi-step Golden Gate cloning. Through benchmarking to MuA transposon-mediated library generation on four ion channel genes, we demonstrate that SPINE-generated libraries are enriched for in-frame insertions, have drastically reduced sequence bias as well as near-complete and highly-redundant coverage. Unlike transposon-mediated domain insertion that was severely biased and sparse for some genes, SPINE generated high-quality libraries for all genes tested. Using the Inward Rectifier K^+^ channel Kir2.1, we validate the practical utility of SPINE by constructing and comparing domain insertion permissibility maps. SPINE is the first technology to enable saturated domain insertion profiling. SPINE could help explore the relationship between domain insertions and protein function, and how this relationship is shaped by evolutionary forces and can be engineered for biomedical applications.

## INTRODUCTION

Up to 80% of metazoan proteins consist of multiple protein domains (1,2). Protein domains are independent units that retain their structure and function (3) as the ‘words’ of the protein universe (4). Domain recombination is an essential process in protein evolution (5,6). Ion channels are a good example of how domain recombination helped rapidly expand functional diversity in the metazoan lineage (7). Inward rectifier K^+^ channels, for example, arose early in cellular life from the combination of a pore domain and a phylogenetically ancient immunoglobulin (Ig)-like domain (7,8), to which different allosteric ligands can bind and affect gating of the pore domain (9).

In biomedical engineering, domain recombination is used to generate synthetic proteins. Many biosensors (10,11) are made by functionally coupling domains that sense a stimulus (e.g. ligand binding, voltage, aberrant protein activity) and domains that report these events (e.g. emitting photons, alter gene expression, induce apoptosis). Similarly, antibodies are joined end-to-end with signaling domains to create chimeric T-cell receptors for immunotherapy (12). Domain recombination enables the design of programmable circuits from multi-domain proteins in living cells (13,14). We recently discovered that domain insertion provides a window into protein dynamics and allostery in ion channels, and it allowed us to generate a light-switchable Inward Rectifier K^+^ channel (15).

Despite the significance of domain recombination in biology and biomedical engineering, saturated domain recombination remains an unsolved problem. By saturated we mean an unbiased approach that redundantly samples all possible insertions of a donor domain into a target protein. To see why saturated approaches are necessary, we should consider that both single amino acid mutations and domain insertions can alter protein structure/function relationships. By comprehensively mapping the impact of these variations, using deep scanning mutagenesis (16) or differential domain insertion profiling (15), we may reveal intrinsic protein properties (17–19), improve our understanding of the mechanistic basis of protein function (20,21), and guide protein engineering (22–24).

Many pioneering contributions have been made to this field but none enable saturated domain recombination. Random insertion approaches include overlap PCR (25,26), and limited nuclease digest with non-homologous recombination (27–29). However, both approaches are inefficient and endonuclease-assisted approaches result in numerous tandem duplications and deletions at insertion sites. Another approach, transposon-mediated domain insertion (30–33), is useful for probing the structure and function of proteins (34,35) (including ion channels (36)), generating new fluorescent proteins (37), or circularly permutating proteins (38,39). The current state of the art is Domain Insertion Profiling through Sequencing (DIP-seq) (24), which combines MuA transposase-assisted library generation with high throughput assays for linking genotype (insertion position) to a phenotype (protein folding, abundance, localization, etc.). DIP-seq has been used to engineer a ligand-sensitive Cas9 (40), a light-switchable ion channel (15) and transcription factors (41).

Transposases, including MuA, have sequence bias (42–48), and create domain insertion libraries with inconsistent insertion frequencies and regions without insertions (15,39,40). Additionally, transposases target random DNA sequences, causing five in six insertions to be in the incorrect reading frame or wrong direction, and the MuA transposition mechanism results in an unavoidable 5 bp replication at the insertion site (49,50). Similar to sequence coverage and depth in genomic analyses (51,52), insertion bias, incomplete coverage, and low redundancy of domain insertion libraries lead to sampling errors that decrease the quality of downstream functional data (53).

Here, we developed a method for domain insertion called Saturated Programmable Insertion Engineering (SPINE). Unlike existing insertional mutagenesis approaches, which rely on the randomness of recombination or transposition, SPINE is a programmed method. It works by dividing a targeted gene into fragments and replacing each fragment with a microarray-synthesized oligo library (54,55). Each oligo in this library contains a genetic handle that can be replaced with a domain of interest by Golden Gate cloning (56). SPINE overcomes many constraints of previous approaches and generates unbiased, saturated, and targeted domain insertion libraries. These improved libraries result in less missing data and improve the dynamic range of assays that measure the impact of domain insertion on target protein expression.

## MATERIALS AND METHODS

### OLS in silico design

Oligo sequences are generated using a custom algorithm (written for Python 3.7.3. and available at https://github.com/schmidt-lab/SPINE) as follows.

#### Target gene fragmentation (Supplemental Figure S1A)

Target gene sequences are submitted in FASTA format. Gene start and end positions within the plasmid are entered manually or calculated from a selected open reading frame. Each gene is divided into evenly distributed fragments to the nearest codon such that the length of each gene fragment does not exceed the length limitations of the synthesized oligo pool (in our case 230 bp) minus additional required components: subpool amplification barcodes (2 × 12 bp), restriction sites (2 × 7 bp), and the domain insertion handle (24 bp). Each fragment break site is adjusted to create unique cut site overhangs for Golden Gate cloning. If adjusting one fragment position causes any fragment to exceed the maximal length, the other fragments are adjusted to equalize fragment distribution below this length threshold.

#### Target gene primer design for inverse PCR (Supplemental Figure S1B)

Forward and reverse plasmid primers are designed to amplify the backbone for each target gene fragment. Additional non-annealing sequences are added to the primer's 5′ end encoding for inward-facing BsmBI recognition sites with the cut site including the first and last codon of the fragment (three bases) plus one base extension for the four base cut site. These primers are optimized for melting temperature and specificity by adjusting the length of the 3′ end. Melting temperatures are set between 55°C and 61°C based on calculations from both Sugimoto *et al.* (57) and SantaLucia and Hicks (58). A primer is flagged as non-specific if annealing temperatures are greater than 35°C at any other position in the plasmid. Non-specific primers are made specific by extending the primer or, if max melting temperatures are exceeded, the fragmented site is adjusted.

#### Design oligos that encode each insertion site (Supplemental Figure S1C)

**Figure 1.**
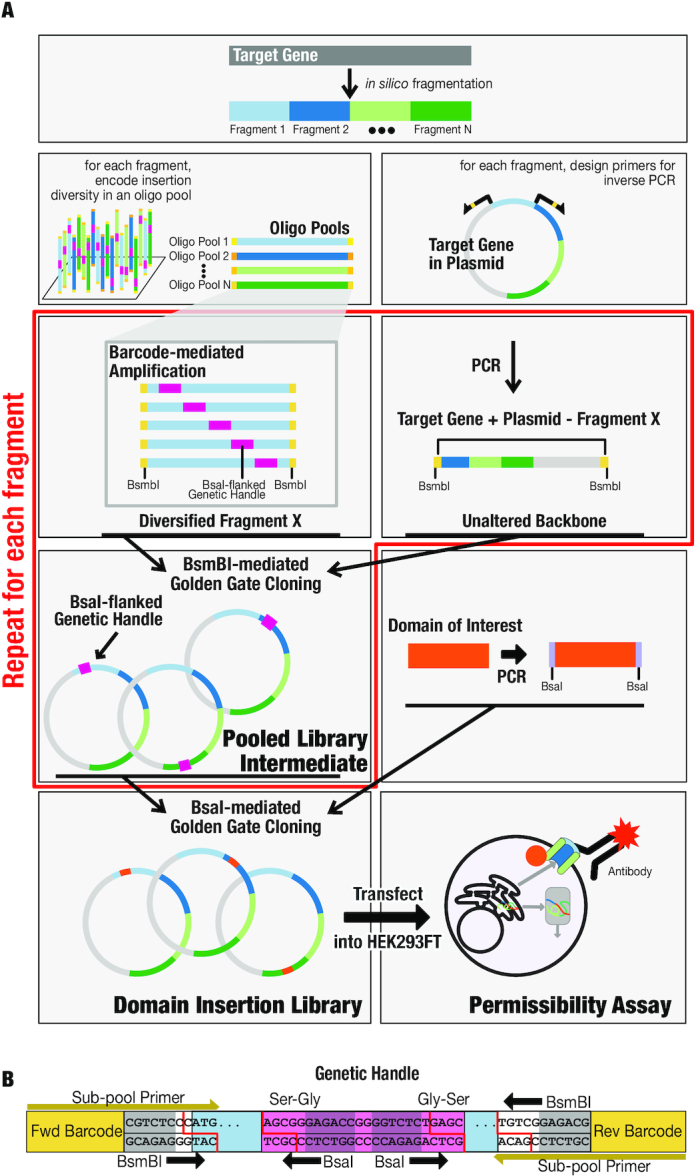
(**A**) SPINE workflow. A target gene sequence is divided into shorter fragments. For each fragment, an oligo pool is generated with a genetic handle (purple) at each amino acid position. Flanking barcodes (different hues of yellow) mediate specific amplification of each subpool, which is then joined with the PCR-amplified target gene backbone in BsmBI-mediated Golden Gate cloning. This process is repeated for each fragment, and the resulting intermediate libraries are pooled. The genetic handle is replaced by a domain of interest (orange) through BsaI-mediated Golden Gate cloning, resulting in the final domain insertion library. (**B**) Barcode Design. Each OLS subpool is designed with a bio-orthogonal barcode followed by a BsmBI recognition site that cuts within the sequence of a gene. Every barcode and BsmBI cut site are unique to a given subpool minimizing the chance for undesired assembly. The genetic handle is designed with outward-facing BsaI recognition sites that enable cutting within the beginning and ends of short flexible serine–glycine linkers. These linkers are the only scars that result from assembly and can be programmed to be any sequence at least 4 bp long.

For each gene fragment, a loop is run to generate an oligo for each insertion position within that fragment, starting after the first codon and ending before the last codon to account for the cloning cut sites. Therefore, sequential fragments overlap by one codon. Oligos consist of a bio-orthogonal barcode for specific subpool amplification, BsmBI recognition sites, and the fragment sequence with a genetic handle insertion (Figure 1B). The genetic handle contains outward-facing BsaI restriction sites, which enable replacement of the handle with a domain of interest, and Ser–Gly and Gly–Ser flexible linkers at the beginning and end of the handle, respectively. Barcodes are courtesy of the Elledge lab (59). In detail, each oligo starts with a forward subpool specific barcode, appended with a forward-facing BsmBI recognition sequence plus one base to bring the cut site into frame. Next, the oligo is appended with the fragment sequence with the insertion handle inserted at the next amino acid position following the previous oligo. Finally, after the gene fragment section one base is added to bring the cut site into frame followed by a reverse facing BsmBI sequence, and a reverse subpool specific barcode.

#### Design of subpool amplifying oligos (Supplemental Figure S1D)

Forward and reverse subpool specific oligo primers are generated by testing annealing of a candidate primer sequence to the respective barcode, BsmBI recognition, and cut sequence. These primers are optimized for annealing temperature as described above, however, because the 3′ end is limited to the cut site, melting temperatures are optimized by adjusting the 5′ end or swapping the barcode sequence.

#### *In silico* quality control (Supplemental Figure S1E)

A final *in silico* quality control is run to check for creation of new BsaI or BsmBI recognition sites and check for non-specific subpool primers across all oligos. If a BsaI or BsmBI recognition site is created, a codon within that recognition site will be changed to an alternative codon maintaining the amino acid sequence. Non-specific subpool primers are identified by an annealing temperature >35°C for any position in any oligo other than the designed position. If a primer is non-specific, that subpool amplification barcode is replaced with another barcode and quality control is repeated. All oligos and primers are exported as FASTA files for ordering.

### Oligo library synthesis (OLS) pool amplification

A 7.5K oligo library synthesis (OLS) pool containing the 2099 oligos for four target proteins (human Kir2.1 (Accession: NP_000882), *Drosophila melanogaster* Shaker (Accession: NP_728123), human α7 nAChR (Accession: NP_000735.1) and human ASIC1a (Accession: NP_001086.2)) was synthesized by Agilent and received as 10 pmol of lyophilized DNA. This DNA was resuspended in 500 μl TE. OLS subpools corresponding to a given gene fragment were PCR amplified using Primestar GXL DNA polymerase (Takara Bio) according to the manufacturer's instructions in 50 μl reactions using 1 μl of the OLS pool as the template and 25 cycles of PCR. The entire PCR reaction was run on 1% agarose gels, visualized with Sybr safe (ThermoFisher) and gel purified (Zymo Research). See also Supplemental Figure S2.

### Combining OLS fragments and target gene backbone

To insert the OLS subpools into target gene backbones, complementary BsmBI sites to those on the OLS fragments of a respective subpool were added by PCR using Primerstar GXL DNA polymerase (Takara) and 100 pg of wildtype channel as template DNA (Supplemental Figure S3A). PCR products were run on 1% agarose gels, visualized with Sybr safe (ThermoFisher) and gel purified (Zymo Research) to remove any undesired PCR by-products.

Target gene backbone PCR product with added BsmBI sites and the corresponding OLS subpools were assembled using BsmBI-mediated Golden Gate cloning (56) (Supplemental Figure S3B). Each 20 μl Golden Gate reaction was composed of 100 ng of backbone DNA, 20 ng of OLS subpool DNA, 0.2 μl BsmBI (New England Biolabs), 0.4 μl T4 DNA ligase (New England Biolabs), 2 μl T4 DNA ligase buffer and 2 μl 10 mg/ml BSA (New England Biolabs). These reactions were placed in a thermocycler overnight with following program: (i) 5 min at 42°C, (ii) 10 min at 16°C, (iii) repeat (i) and (ii) 40 times, (iv) 42°C for 20 min, (v) 80°C for 10 min. Reactions were cleaned up using Zymo Research Clean and Concentrate kits, eluted in 10 μl of elution buffer, transformed into E. cloni^®^ 10G chemically competent cells (Lucigen) according to manufacturer's instructions. Cells were grown overnight at 30°C to avoid overgrowth in 50 ml LB with 40 μg/ml kanamycin with shaking, and library DNA was isolated by miniprep (Zymo Research). A small subset of the transformed cells was plated at varying cell density to assess transformation efficiency and validate successful insertions with colony PCR. All libraries at this step yielded >7000 colonies corresponding to >45× coverage for perfect mutations assuming one-third of mutants are perfect. All libraries (corresponding to different subpools) of a given target gene were pooled together at an equimolar ratio, resulting in a mixture of insertions at every amino acid position (Supplemental Figure S3C).

### Replacing the genetic handle with the domain of interest for ASIC1a, Shaker, and α7nAChR

Cib81 (60) was ordered as a gBlock (IDT DNA). BsaI sites complementary to those in the inserted genetic handle were added to Cib81 by PCR using Primestar Max (Takara Bio) according to the manufacturer's instructions (Supplemental Figure S3D). The genetic handle in each target gene insertion library was replaced with Cib81 by BsaI-mediated Golden Gate cloning. Each 20 μl Golden Gate reaction contained 100 ng of backbone DNA, 15 ng of Cib81 DNA, 0.2 μl BsaI-HFv2 (New England Biolabs), 0.4 μl T4 DNA ligase (New England Biolabs), 2 μl T4 DNA ligase buffer, and 2 μl 10 mg/ml BSA. These reactions were placed in a thermocycler overnight with following program: (i) 5 min at 37°C, (ii) 10 min at 16°C, (iii) repeat (i) and (ii) 40 times, (iv) 37°C for 20 min, (v) 80°C for 10 min. Reactions were cleaned up using Zymo Research Clean and Concentrate kits, eluted in 10 μl of elution buffer, transformed into E. cloni^®^ ELITE electrocompetent cells (Lucigen) in 1.0 mm Biorad cuvettes using a Bio-Rad Gene Pulser II electroporator (settings: 10 μF, 600 Ω, 1.8 kV). Cells were grown overnight at 30°C to avoid overgrowth in 50 ml LB with 40 μg/ml kanamycin with shaking, and library DNA was isolated by miniprep (Zymo Research). A small subset of the transformed cells was plated at varying cell density to assess transformation efficiency and validate successful insertions with colony PCR. All libraries at this step yielded >7000 colonies corresponding to >45× coverage for perfect mutations assuming one-third of mutants are perfect.

### Replacing the genetic handle with the domain of interest for Kir2.1

We noticed that our libraries had contaminating wildtype DNA, which was likely due to trace amounts of template DNA left over from PCR amplification of target gene backbones, and which became enriched from multiple transformations. In preparation for the functional assay on Kir2.1-Cib81, we added an antibiotic selection step to remove WT DNA and enrich insertion variants. A chloramphenicol antibiotic cassette was amplified by PCR with primers to add BsaI sites complementary to the genetic handle, and outward-facing BsmBI sites, which enable replacement of the antibiotic cassette with a domain of interest, in this case, Cib81. BsaI-mediated Golden Gate followed the same scheme as replacing the genetic handle with the chloramphenicol antibiotic cassette. We transformed this Golden Gate reaction into E. cloni^®^ 10G ELITE electrocompetent cells in 1.0 mm Biorad cuvettes using a Bio-Rad Gene Pulser II electroporator (settings: 10 μF, 600 Ω, 1.8 kV). Cells were grown overnight at 30°C in 50 ml LB with μg/ml kanamycin and 25 μg/ml Chloramphenicol LB with shaking to avoid overgrowth. Library DNA was isolated by midiprep (Zymo Research). A small subset of the transformed cells was plated at varying concentrations of cells to assess transformation efficiency and validate successful insertions with colony PCR. This library yielded >100 000 colonies corresponding to >300× coverage for perfect mutations assuming one-third of mutants are perfect.

We PCR-amplified Cib81 with BsmBI sites complementary to the antibiotic cassette. This antibiotic cassette was replaced with PCR amplified Cib81 using BsmBI-mediated Golden Gate as described above. Libraries were transformed into E. cloni^®^ 10G ELITE electrocompetent cells in 1.0 mm Biorad cuvettes using a Bio-Rad Gene Pulser II electroporator (settings: 10 μF, 600 Ω, 1.8 kV). Cells were grown overnight at 30°C in 50 ml LB with 40 μg/ml kanamycin with shaking to prevent overgrowth. Library DNA was isolated by midiprep (Zymo Research). This library yielded >100 000 colonies corresponding to >300× coverage for perfect mutations assuming one-third of mutants are perfect.

### MuA transposon mediated domain insertion

Transposition libraries were generated using 100 ng MuA-BsaI engineered transposon and 1:2 molar ratio transposition target DNA in 20 μl reactions with 4 μl 5× MuA reaction buffer and 1 μl 0.22 μg/μl MuA transposon (ThermoFisher). MuA–BsaI engineered transposon propagation plasmid or pUCKanR-Mu-BsaI was a gift from David Savage (Addgene plasmid # 79769). MuA-BsaI engineered transposon was digested with BglII and HindIII Fastdigest enzymes (ThermoFisher) and gel purified using gel purification kit (Zymo Research).

The transposition targets, human Kir2.1 (Accession: NP_000882), *Drosophila melanogaster* Shaker (Accession: NP_728123), human α7 nAChR (Accession: NP_000735.1) and human ASIC1a (Accession: NP_001086.2) including a porcine teschovirus ribosomal skipping sequence (P2A) (61), were codon-optimized for mouse, synthesized (Gen9) and subcloned with into pATT-Dest using NEB BamHI and HindIII. pATT-Dest was a gift from David Savage (Addgene plasmid # 79770). For Kir2.1, a FLAG tag was inserted after T115 using Q5 site-directed mutagenesis (New England Biolabs). MuA transposition reactions were incubated at 30ºC for 18 hours for transposition, followed by 75°C for 10 min for heat inhibition. DNA from reactions was cleaned up (Zymo Research) and eluted in 10 μl water. All 10 μl were transformed into 30 μl E. cloni^®^ 10G ELITE electrocompetent cells (Lucigen) in 1.0 mm Biorad cuvettes using a Bio-Rad Gene Pulser II electroporator (settings: 10 μF, 600 Ω, 1.8 kV). Cells were rescued and grown without antibiotics for 1 h at 37°C. Aliquots were then serially diluted and plated on LB agar plates containing carbenicillin (100 μg/ml) and chloramphenicol (25 μg/ml) to assess library coverage. The remaining transformation mix was grown in 50 ml LB containing carbenicillin (100 μg/ml) and chloramphenicol (25 μg/ml). All transformed libraries yielded greater than 10^5^ colonies, which for Kir2.1-P2A (1369 bp) is >35× coverage. Plasmid DNA was purified by midi-prep kit (Zymo Research).

Transposition-inserted Kir2.1 variants were subcloned into an expression vector by amplifying channel variant genes adding on BsmBI sites, using 10 cycles of PCR using Primestar GXL (Takara Bio) and run on a 1% agarose gel. The larger band was cut out and gel purified (Zymo Research) to isolate channels with inserted transposons. A mammalian expression vector (pcDNA3.1) with EGFP was amplified to add on BsmBI sites complementary to those on Kir2.1-P2A. The Kir2.1-P2A (BsaI-transposon) variants were subcloned into this vector by BsmBI-mediated Golden Gate cloning (56). Reactions were cleaned (Zymo Research) and eluted with 10ul water. All 10 ul were transformed into 30 μl E. cloni^®^ 10G ELITE electrocompetent cells (Lucigen) in 1.0 mm Biorad cuvettes using a Bio-Rad Gene Pulser II electroporator (settings: 10 μF, 600 Ω, 1.8 kV). Cells were rescued and grown without antibiotics for 1 h at 37°C then with an aliquot serially diluted plated on LB agar plates containing kanamycin (50 μg/ml) and chloramphenicol (25 μg/ml) to assess library coverage. The remaining transformation mix was grown in LB containing kanamycin (50 μg/ml) and chloramphenicol (25 μg/ml). All transformed libraries yielded greater than 10^5^ colonies, which correspond to >35× coverage. Plasmid DNA was purified by midi-prep kit (Zymo Research).

Inserted Transposons were replaced with domains in individual reactions using BsaI-mediated Golden Gate cloning. Cib81 was PCR amplified to add on BsaI and linkers (Ala–Ser and Ser–Ala–Gly), preceding and following the domain insertion) sites complementary to MuA-BsaI transposon sites for Golden Gate cloning. Domain amplicons were gel purified (Zymo Research). The product was further digested with AgeI-HF (NEB) and Plasmid-Safe ATP-dependent DNase (Epicentre) to remove any undigested transposon, then cleaned up (Zymo Research) and eluted with 10 μl water. All 10 μl were transformed into 30 μl E. cloni^®^ 10G ELITE electrocompetent cells (Lucigen) in 1.0 mm Biorad cuvettes using a Bio-Rad Gene Pulser II electroporator (settings: 10 μF, 600 Ω, 1.8 kV). Cells were rescued and grown without antibiotics for 1 hour at 37°C. An aliquot was serially diluted and plated LB agar plates containing kanamycin (50 μg/ml) to assess library coverage. The remaining transformation mix was grown in LB containing kanamycin (50 μg/ml). All transformed libraries yielded >10^5^ colonies meaning there is >35× coverage. Plasmid DNA was purified by midi-prep kit (Zymo Research).

### Permissibility assay

100 ng of MuA-generated and five concentrations of SPINE-generated Kir2.1 insertion library (50 ng, 100 ng, 200 ng, 400 ng, 600 ng, 1.2 μg) were transfected with 36 μl of Turbofect (ThermoFisher) into 50% confluent HEK293FT (Invitrogen) with additional inert plasmid (pATT Dest) added to a total of 12 μg transfected DNA divided across a single six-well dish (9.6 cm^2^/well). Multiple concentrations were used to artificially boost the noise level in the SPINE libraries to further challenge the assay. The 50 ng (0.5%) data was not included in downstream analysis as too few cells expressed Kir2.1 to yield high quality permissibility data.

Cells from each well were detached using 1 ml Accutase (Stemcell Technologies) and twice spun down at 450g and resuspended in FACS buffer (2% of FBS, 0.1% NaN_3_, 1× PBS). Cells were incubated with 1:200 anti-flag mouse antibody (Sigma) 1 hour rocking at 4°C, washed twice with FACS buffer, covered with aluminum foil, and then incubated with 1:400 anti-mouse Alexa Fluorophore 568 (Thermo Fisher) for 30 min rocking at 4°C. We will refer to Alexa Fluorophore 568 as ‘label’ from hereon. Cells were washed twice, resuspended in 3 ml FACS buffers, and filtered using cell strainer 5 ml tubes (Falcon). Cells were kept on ice and protected from light in the transfer to the flow cytometry core. Before cell sorting, a small aliquot of cells was saved as a control sample for sequencing.

Cells were sorted into EGFP high/label low (transfected cells without surface expression) and EGFP high/label high (transfected cells with surface expression) on a BD FACSAria II P69500132 flow cytometer. EGFP fluorescence was excited using a 488 nm laser, recorded with a 525/50 nm bandpass filter and a 505 nm long-pass filter. Alexa fluorophore 568 fluorescence was excited using a 561 nm laser and recorded with a 610/20 nm bandpass filter. Cells were gated on side scattering and forward scattering area to select whole HEK293FT cells, gated on forward scattering height and width to separate single cells, then gated on co-expressed EGFP to gate out cells that received a plasmid, then gated on cells that were labeled using the anti-flag antibody for surface-expressed channels. Gates were determined using single wildtype, EGFP only, and unstained library samples. A representative example of this gating scheme is shown in Supplemental Figure S4. EGFP high/label low and EGFP high/label high cells were collected into catch buffer (20% of FBS, 0.1% NaN_3_, 1× PBS). As many cells as possible (between 2000 and 100 000 cells) were collected for each sample/library pair which is ∼4–250× coverage of all potentially productive (i.e. in-frame and forward) domain insertions.

### NextGen Sequencing

DNA from pre-sort Control, EGFP high/label low, and EGFP high/label high cells for each library were extracted using a Microprep DNA kit (Zymo Research) and triple eluted with water. To remove chromosomal DNA, samples were digested with Plasmid-Safe ATP-dependent DNase (Epicentre). The resulting plasmid DNA was further purified and concentrated using (Zymo Research). The product was used as a template for 12 cycles of PCR using Primestar GXL (Takara Clontech), run on a 1% agarose gel, and gel purified (Zymo Research) to remove primer dimers and non-amplicon DNA. Purified DNA was quantified using Picogreen DNA concentration and equal amounts of each domain insertion sample were pooled by cell sorting category (control, EGFP high/label low, EGFP high/label high). Pooled amplicons were prepared for sequencing using Nextera XT sample preparation workflows. Libraries were sequenced using Illumina MiSEQ in 300 bp paired-end configuration. Read count statistics are provided in Supplemental Table S1.

### Domain insertion permissibility enrichment

Alignments were done individually on both forward and reverse reads using a DIP-seq pipeline (24,40), slightly modified for compatibility with updated python packages. In rare instances, both forward and reverse reads report domain insertion events, which results in duplicated domain insertion calls. In this event, the duplicated domain insertion call is removed to avoid artificially boosting some events. This pipeline results in plaintext files indicating domain insertion positions and whether that insertion is in-frame and in the forward direction. Enrichment was calculated by comparing the change in EGFP high/label low to EGFP high/label high cells. Only positions with reads in both cell groups were used in enrichment calculations. All other positions are treated as ‘NA’ and not considered in downstream analysis and structure mappings, except for calculating correlations between datasets and correlations between insertion sites. In these correlation calculations, we treated ‘NA’s as ‘0’s, because removing all the data will introduce more noise when comparing between datasets due to sampling limits.

Permissibility function for individual datasets comparing surface expressed (SE) and non-surface expressed (NSE) insertion variants:

(1) $$\begin{equation*}F\ \left( {i,j} \right) = \ \frac{{r_{{j_{SE}}}^i}}{{{t_{{j_{SE}}}}}} - \frac{{r_{{j_{NSE}}}^i}}{{{t_{{j_{NSE}}}}}}\end{equation*}$$

where *r* is the number of reads at amino acid position *i*, in the *j*th dataset divided by *t*, the total number of reads in the *j*th given sample.

### Library comparison

To compare read counts across multiple proteins, we normalized each gene by dividing each insertion site read count by the total number of reads for the respective gene. To account for variable gene length, we then multiplied the normalized read count by the number of amino acids for the respective gene to obtain normalized insertions per residue. Ideally, every insertion position would have a value of one, indicating an evenly distributed insertion library. To test how evenly distributed our libraries are, we compared the distribution using empirical cumulative probability density plot, which indicates both mean read count at 0.5 cumulative probability and the distribution of read counts by the slope. We also compared the library coverage (fraction of insertion positions) of each gene at increasing read depth thresholds (genes were normalized to 300 reads per position).

Domain insertion permissibility per position was z-scored:

(2) $$\begin{equation*}{z_i} = \frac{{{x_i} - \mu }}{\sigma }\ \end{equation*}$$

where *x* is permissibility at amino acid position *i*, *μ* is the sample mean permissibility and *σ* is the sample standard deviation.

Z-scored permissibility was mapped onto the structure of chicken Kir2.2 (PDB 3SPI) (62) using Chimera (63).

Sequence logos were generated using the *ggseqlogo* R package using the ‘twosamplelogo’ sequence logo method which enables removal of any sequence background from the sequence logo resulting in a more accurate sequence logo (64).

### Determining depletion of single base pair deletions and enrichment of in-frame domain insertions in the correct direction

To quantify single base pairs deletions (the predominant type of synthesis error with phosphoramidite chemistry (65–67)) in the SPINE permissibility sequencing data, we aligned paired-end reads from each dataset (Control, EGFP high/label low, and EGFP high/label high) to the sequence of Kir2.1 using the BBMap alignment package (68). We calculated the frequency of deletions in each dataset by dividing the number of 1 bp deletions detected in the aligned reads by the total number of aligned reads. To calculate enrichment of 1 bp deletion in EGFP high/label low, and EGFP high/label high datasets, we divided deletion frequency in these datasets by the deletion frequency of the corresponding control dataset.

To quantify incorrect reading frame insertion and directionality, we used the output from the DIP-seq alignment pipeline for each dataset (control, EGFP high/label low, and EGFP high/label high). The DIP-seq alignment pipeline assigns a DNA insertion position and the direction of every insertion into a recipient gene. Using this data, we calculated frequencies for every reading frame (0, +1, +2) and insertion direction (plus, minus) as the number of reads in each of these six classes divided by the total number of reads. Enrichment for each class was then calculated by dividing each of these classes for EGFP high/label low, and EGFP high/label high dataset by the corresponding control dataset.

## RESULTS

### The SPINE workflow

SPINE is enabled by microarray-based massive oligonucleotide library synthesis (OLS) (54,55). OLS libraries are used for large-scale parallel gene synthesis (69,70) and generating saturated mutation libraries through oligo annealing (71) or recombination (72,73). Similarly, we combined OLS library synthesis with multi-step Golden Gate Cloning (56), to generate domain insertion libraries in a programmable fashion (Figure 1A).

Current OLS can produce oligos with a maximum length of 230 base pairs (bp) (55). We broke up each target gene into fragments, whose insertional diversity is encoded by OLS library subpools. Each subpool contains about 170 bp of gene sequence flanked by biorthogonal barcodes for PCR amplification, and Golden Gate-compatible BsmBI sites for cloning the fragment into the target gene (Figure 1B). Varied between the oligos in each subpool is the genetic handle, which is inserted at every amino acid position of the target gene fragment corresponding to this subpool. Genetic handles are designed with Golden Gate-compatible BsaI sites at the beginnings and ends of linkers that allow replacement with any DNA sequence (in our case, a domain). The overhangs generated by these BsaI sites also encode the amino acids that serve as linkers between the target protein and the inserted domain. We here chose a short serine/glycine linker, which is widely used as a flexible linker (74), but any linker at least 2 amino acids long can be encoded in the BsaI overhangs.

For each fragment subpool, we generated corresponding target gene backbones plus complementary BsmBI cut sites by PCR amplifying, from a shuttle plasmid, all of the wildtype gene except for the region of the gene encoded by the fragment subpool. The OLS subpools were assembled with their corresponding backbone fragments in BsmBI-mediated Golden Gate reactions. This process was repeated for all fragment subpools and these libraries are combined in equimolar ratio to yield pooled intermediate libraries. The final domain insertion libraries were generated by replacing the genetic handles with a PCR-amplified domain of interest flanked by complementary BsaI cut sites and flexible linkers using BsaI-mediated Golden Gate cloning.

### Domain insertion library generation

Guided by our interest in probing the relationship between domain recombination and ion channel function, we generated domain insertion libraries with four ion channel genes, inward rectifier K^+^ channel Kir2.1, voltage-dependent K^+^ channel Shaker, α7 nicotinic acetylcholine receptor (α7nAChR), and the acid-sensing ion channel ASIC1a. In this proof-of-principle, we replaced the genetic handle with the 9 kDa plant protein domain Cib81 (60). Cib81 was chosen as a benchmark because we had used Cib81 in transposon-generated libraries (15).

To determine insertion library error rates and contamination with wildtype DNA (a leftover from the inverse PCR to generate the target gene backbone, which becomes enriched in the multistep cloning), we sequenced individual clones by Sanger sequencing from intermediate libraries (contain the genetic handle) and final domain-inserted libraries (contain Cib81). We found that ∼40% of clones had the expected sequences without any errors (Table 1). Conversely, ∼60% of clones had errors, with 1 bp deletions being the most frequent (41%) and 7% of clones were wildtype. In downstream functional assays, a wildtype channel would lead to significant false positives. We, therefore, replaced the genetic handle for Kir2.1 with a chloramphenicol antibiotic cassette to enrich for oligo incorporated plasmids before replacing with Cib81. This removed any contaminating wildtype DNA (Selected Cib81, Table 1). Overall, SPINE yielded similar percentages of perfect insertion libraries and wildtype to comparable targeted mutational approaches that use oligo library synthesis (71,72).

**Table 1.** Insertion library error rates

	Genetic handle counts(%)	Unselected CIB81 counts(%)	Selected CIB81 counts(%)
Colonies sequenced	88(NA)	90(NA)	81(NA)
Perfect clones	35(39.8)	31(34.4)	34(42.0)
Clones with 1 bp deletions	36(40.9)	33(36.7)	36(44.4)
Total 1 bp deletions	40(NA)	44(NA)	51(NA)
1 bp insertions	3(3.4)	2(2.2)	1(1.2)
Missense mutations	1(1.1)	7(7.8)	6(7.4)
> 1 bp deletions	12(13.6)	10(11.1)	1(1.2)
> 1 bp insertions	0(0)	4(4.4)	14(17.3)
Wildtype	7(8.0)	6(6.7)	0
Wildtype: surface trafficked		6/37 = 16%	0

### SPINE libraries have increased and more consistent saturation

The current state-of-the-art for generating domain insertion libraries relies on MuA transposase (24). However, MuA transposon-generated libraries have incomplete coverage (15,40) and strong sequence bias (42–48).

**Figure 2.**
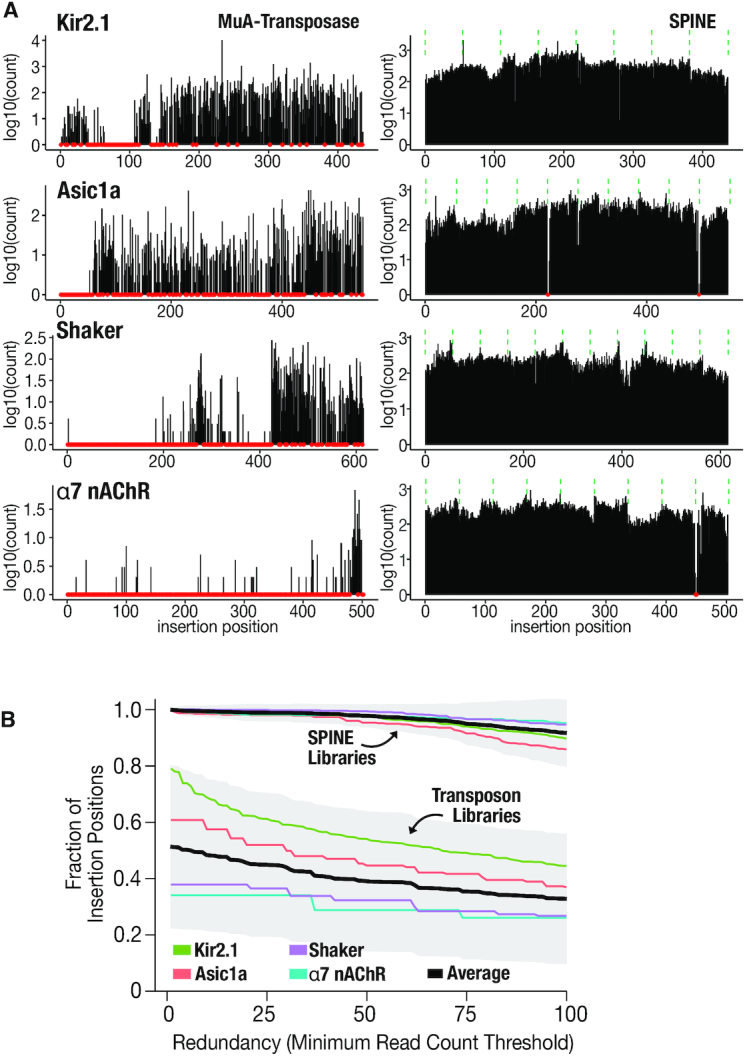
(**A**) SPINE libraries are saturated. Comparison of MuA-tranposase generated insertion libraries and SPINE for four different ion channels. Red dots indicate missing positions. Green dashed lines indicate fragment boundaries for SPINE libraries. (**B**) SPINE libraries have deep coverage. Shown is the fraction of insertion positions for a given target gene that have the indicated coverage for each method. The average for each method is shown as a black line and the 95% confidence interval is shaded grey.

To test whether SPINE libraries can overcome bias and low coverage problems that exist in MuA-based methods, we benchmarked them against transposon-generated libraries. The difference in coverage is easily apparent from visual inspection (Figure 2A log-transformed, Supplemental Figure S5 raw counts). We found that SPINE libraries had an average of 99.97% coverage compared to 49% for MuA transposase. In the most extreme case, α7nAChR, coverage went from less than 40% of positions having at least five reads with MuA transposase to a greater than 95% of positions having at least 55 reads per position using SPINE (Figure 2B). Furthermore, the probability of coverage stays flat for a considerable read depth range (1–80 reads), which suggests that coverage is less variable and more redundant.

### SPINE libraries have drastically reduced sequence bias

**Figure 3.**
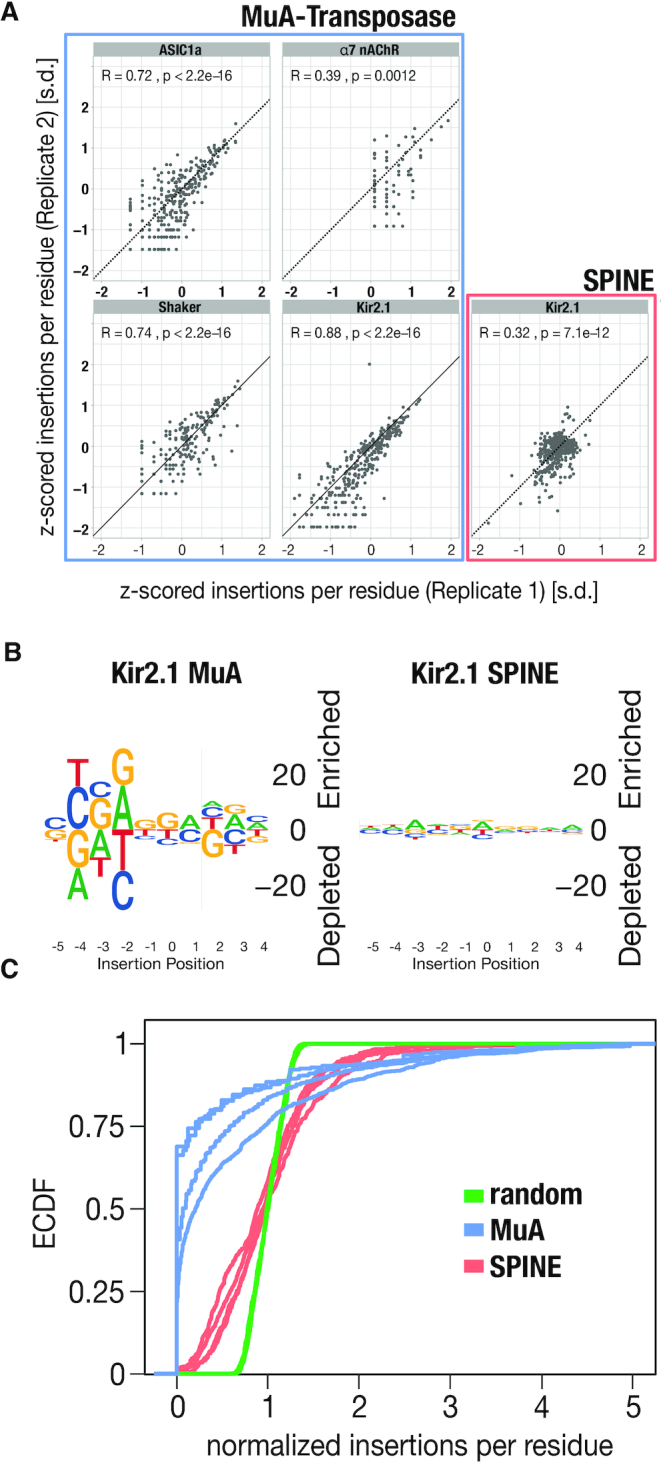
SPINE has drastically reduced bias. (**A**) Scatterplots show z-scored insertions per residue for each biological replicate. Spearman correlation coefficients are inset. (**B**) Sequence logos for insertion sites in Kir2.1 using MuA-transposition (left) and SPINE (right). SPINE libraries are less sensitive to a targeted gene sequence. (**C**) Empirical cumulative density functions of four different target genes generate by MuA-transposition (blue lines) and SPINE (red lines). An idealized random library is shown (green lines). While ECDFs for SPINE libraries are very similar to each other and similar to a random distribution (two-sample Kolmogorov–Smirnov test, *D* = 0.29084, *P*-value < 2.2e–16), for MuA libraries they are highly variable among each other and different from a random distribution (two-sample Kolmogorov–Smirnov test, *D* = 0.74488, *P*-value < 2.2e–16).

We compared replicates of generated libraries to test whether the uneven coverage we observed in MuA transposons was due to sampling or sequence bias. We found similar insertional maps from replicates in Kir2.1, ASIC1a, and Shaker transposon libraries, which reiterates previous reports on MuA transposase bias (43,46–47) (Figure 3A). That bias became apparent when we generated a sequence logo for MuA-mediated insertion positions in Kir2.1 (Figure 3B) and the other channels (Supplemental Figure S6B and C). In agreement with known MuA bias, we found enrichment for insertions at trinucleotide CGG position (43,46–48). In contrast, SPINE library replication had lower insertional map similarity (Figure 3A), and no strong and repeated sequence logo was apparent (Kir2.1 Figure 3B; Shaker, ASIC1a, α7nAChR Supplemental Figure S6B–D), which shows that SPINE has drastically reduced bias. To compare the variability of random insertions with respect to targeted genes, we compared the empirical cumulative probability distribution functions (ECDF) for a simulated random distribution for each target gene with to those from MuA-generated libraries and SPINE (Figure 3C). While ECDFs for SPINE libraries are very similar to each other and similar to a random distribution (two-sample Kolmogorov–Smirnov test, *D* = 0.29084, *P*-value < 2.2e–16), for MuA libraries they are highly variable among each other and different from a random distribution (two-sample Kolmogorov–Smirnov test, *D* = 0.74488, *P*-value < 2.2e–16).

### SPINE libraries only contain productive domain insertions

MuA transposition yields insertions in all six reading frames, which we confirmed for MuA libraries. Only 16% (1/6) of insertions are in the correct reading frame and direction (Supplemental Figure S7B). In contrast, 99% of insertions were in-frame and forward in SPINE libraries due to this technique's programmed nature. Even if we account for SPINE’s 58% error rate (Table 1) and make the best-case scenario assumption that MuA libraries have 0% errors apart from random insertion frame selection, SPINE results in more productive insertions than MuA transposons (44% in-frame and forward for SPINE versus 16%).

Taken together, SPINE enables the generation of saturated domain insertion libraries with drastically reduced insertion position bias, near-complete coverage, and redundant insertions at each position. SPINE libraries are furthermore enriched for productive in-frame insertions in a target gene.

### SPINE enables saturated domain insertion profiling

We previously used transposon-mediated library generation to profile domain insertion permissibility in Kir2.1 (15). We transiently transfected insertion libraries into HEK293 cells and performed a functional assay to measure permissibility. Permissibility is the sensitivity of a channel to the insertion of a domain at a given position and is determined by measuring how well a channel variant folds, assembles, and traffics to the cell surface. All insertion variants express EGFP as a transfection marker, but only surface-expressed variants are fluorescently labeled via an extracellular FLAG tag. Using fluorescently activated cell sorting (Figure 1A, Functional Assay), we isolate cells that express insertion variants that fold, assemble, and traffic well (EGFP high/label high), from insertion variants that do not (EGFP high/label low). We connect genotype (insertion variant) to phenotype (permissibility) by recovering and sequencing plasmids in sorted populations.

A potential problem with transient transfection is that each cell expresses a mix of insertion variants. When we sort a cell that contains a well-expressing insertion variant, sequencing will recover the coding sequence for a folding variant (the signal) and sequences that are unrelated to the phenotype (noise). Second, K^+^ channels form tetramers which might be composed of monomers with different insertion variants; also increasing the noise. While the signal-to-noise was sufficient to conduct our work in Kir2.1 with transposon-generated insertion libraries, we wanted to establish that a surface expression assay coupled to transient transfection with diluted DNA still yields sufficient signal-to-noise in the background of SPINE libraries. As determined earlier, 60% of clones in a SPINE library have errors that stem from inefficiencies in oligo synthesis (69). The predominant errors are 1 bp deletions (65–67). Deletions will lead to frameshift mutations and premature stop codons, which should disrupt ion channel folding, assembly, and surface trafficking. When we determined enrichment/depletion of 1 bp deletions relative to the pre-sort control, we found slight enrichment in cells expressing non-surface trafficked insertion variants (Supplemental Figure S7A). Importantly, in cells with surface-trafficked insertion variants, they were depleted. Degree of enrichment and depletion appears dependent on how much library DNA was used in the transfection. Specifically, when library DNA made up only 0.5% of the total amount of transfected DNA, 1 bp deletions where enriched ∼12% in cells expressing predominantly misfolded Kir2.1, while they were depleted by ∼50% in cells expressing predominantly surface-expressed Kir2.1. With an increasing amount of library DNA, that difference grew smaller until no depletion or enrichment (in comparison to pre-sort control) was observed. We also found that despite the increased noise from increasing amounts of library DNA, permissibility assay with SPINE-generated libraries where more repeatable (0.56 SPINE versus 0.38 MuA transposase mean Spearman correlation coefficients, Supplemental Figures S8 and S9).

In aggregate, these data agree with the expectation that deletions cause frameshift mutations or premature stop codons, which would cause ion channels to incorrectly fold, assemble, or traffic. Given that deletions in a cell with predominantly permissive insertion variants are depleted, suggests that even with transient transfection, our permissibility assay has sufficient signal-to-noise. The data also suggests this phenotype (surface-expression) is unlikely to be influenced by the higher mutation rate in SPINE libraries.

**Figure 4.**
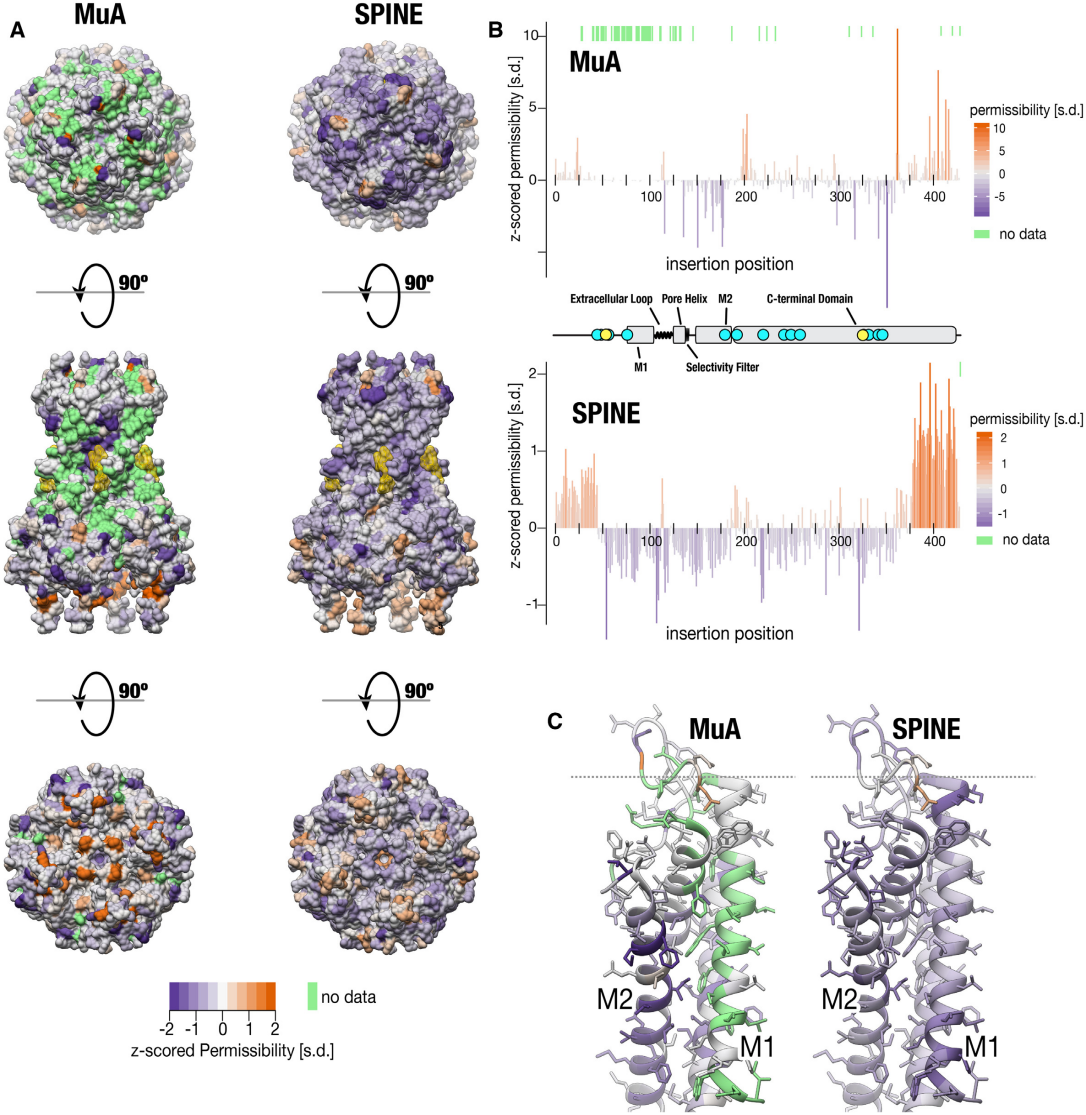
Domain Insertion Permissibility. (**A**) Permissibility data for Cib81 insertion libraries derived from MuA-transposition and SPINE is mapped on the crystal structure of chicken Kir2.2 (PDB: 3SPI (62)) Green indicates missing data. PIP_2_, an allosteric modulator of Kir, is rendered yellow. (**B**) Secondary structure elements (center) are shown along with *z*-scored permissibility for Cib81 insertion for MuA- and SPINE- generated libraries. Cyan dots indicate functionally important sites. Yellow dots indicate trafficking signals important for surface expression. Green lines above each dataset indicate missing data. (**C**) Comparison of permissibility coverage for transmembrane domains (M1 and M2).

Having tested the sensitivity of our permissibility assay, we explored whether SPINE could improve permissibility map resolution in Kir2.1 compared to MuA transposition. With Cib81 as the inserted domain, we found SPINE improved permissibility maps. A visual inspection of permissibility data (averaged across three independent replicates) mapped onto the crystal structure of human Kir2.2 (62), visualizes the striking difference in saturation and dynamic range (Figure 4A). While MuA library data is sparse with 71 sites missing and noisy, SPINE library data is almost complete (1 site is missing) and has a high dynamic range between highest and lowest permissibility. Plotting permissibility along sequence position shows that formerly missing regions are now filled in (Figure 4B). For example, for a large region at the beginning of the gene (amino acid positions 1–150) little permissibility information is available from MuA libraries (which had poor insertion coverage and depth in this region) while permissibility is measured for the entire region with SPINE libraries. In this region, there are the unstructured N terminus, several regulatory sites, and M1 transmembrane domain (Figure 4B, protein topology cartoon) that are functionally important (62). The interface between the M1 and M2 helix is now well resolved, while most positions were missing in MuA libraries (Figure 4C). For all other regions, SPINE conforms to previous permissibility patterns while providing a more complete and dynamic data set. There now appear to be four levels of permissibility in Kir2.1: high permissibility for the unstructured C-terminus, moderate permissibility in the N-terminus, low permissibility in the structured cytosolic regions and no permissibility in transmembrane regions. In addition, within regions with expected high (flexible N/C termini) or no permissibility (transmembrane domain), there are fewer probable false positives or negatives (15/119 MuA versus 6/157 SPINE; two-sided *z* score, *P*-value: <0.0064). Further emphasizing the improved quality of permissibility maps is that insertion into a known Golgi export signal (75) have clearer negative permissibility in the SPINE data.

## DISCUSSION

Transposase-mediated domain insertion is widely used to address both basic science and biomedical engineering questions (30–33). We developed SPINE as an alternative approach that uses oligo library synthesis and multi-step Golden Gate cloning to assemble domain insertion libraries in a programmable fashion. Which approach investigators choose depends on what best meets the experimental requirements; SPINE compares favorably in several aspects.

The sequence bias and variable efficiency of transposases is well established (42–48). We and others showed, in different protein families, that this can result in domain insertion libraries that have bias, incomplete coverage, and variable coverage redundancy (15,24,41). For all tested genes SPINE has reduced bias, near-complete coverage, and superior coverage redundancy. The success of using transposon-based domain insertion to construct, for example, biosensors (24) may suggest that transposon-based approaches work well enough. And in light of the same general trends observed in domain insertion permissibility maps for Kir2.1 in this study, one could argue that bias, lack of coverage, and depth do not matter. However, for some target genes –such as nAChR in this study– transposon-generated domain insertion libraries have such severe bias and marginal saturation that they are effectively unusable for applications that derive insight from comprehensive mapping of all possible domain insertions (15). It is hard to intuit for which target genes transposon-mediated domain insertion will perform poorly and there may be limited recourse. For example, changing codon usage, did not improve nAChR libraries (unpublished observation). In other cases, functionally important regions have no domain insertion events, such as the S4 and S5 linker in Shaker (important in mediating channel opening in response to change in voltage (76)) and a Na^+^ binding pocket in Kir2.1 (77,78). There is value in a domain insertion method that is predictable and dependable. Furthermore, lack of bias, near-complete, and redundant coverage result in richer functional data. In this study, this manifests as improved dynamic range of the Kir2.1 domain insertion permissibility signal. For other engineered proteins the case remains to be made, but we predict that SPINE will produce more complete domain insertion maps, which will increase the likelihood of finding, for example, a functional biosensor.

In the DIP-seq approach, type IIS restriction sites are embedded in the MuA transposase recognition sites to mediate the exchange of the transposon inserted into the target gene with a domain of interest with compatible flanking overhangs (24). The simultaneous requirements of maintaining transposition efficiency (50,79) and restriction efficiency puts sequence constraints on restriction enzyme recognition sequences and overhangs. Because overhang sequences are added to the original target at the insertion site and encode linkers, the amino acid composition of these linkers is constrained. Linker optimization is a critical aspect of fusion protein engineering (74,80). SPINE offers a significant advantage over MuA-transposon approaches because it puts no constraint on linker composition. We used a serine/glycine linker, which is used as a flexible linker (74), but any linker sequence of two amino acids at either side of the inserted domain can be used. This enables full exploration of how linker length and composition impact target protein function independent of the inserted domain. If the first and last two amino acids of the inserted domain are included as overhangs in the genetic handle, it is possible to insert a domain without any linkers. However, this would require a new OLS library for each inserted domain.

MuA can insert a transposon into any of the six reading frames, while the programmable nature of SPINE results in enriched in-frame insertions. When phenotyping assays are coupled to sequencing, as is the case for DIP-seq (24) or CPP-seq (39), SPINE allows for more efficient use of the specified sequencing output because fewer reads are spent on unproductive insertions. Furthermore, SPINE insertion libraries can be targeted to single or multiple regions of the target gene and, thus, avoid undesired insertions. Achieving the same with MuA transposases requires multiple intermediate staging libraries that contain the targeted regions, which then are subcloned with the remainder of the target gene. This feature of SPINE not only simplifies domain insertion workflows, but provide easier access to complex domain insertion library designs. In Kir2.1 for example, targeting domain insertions to known allosteric sites in Kir2.1 while avoiding transmembrane region or trafficking signals could be a promising strategy to efficiently construct light- and drug-switchable versions of this ion channel.

SPINE relies on microchip-synthesized oligonucleotides which have an overall error rate of ∼0.2% (1 in 500 bp) (69). This means that only ∼50% of the oligos in a 230 bp OLS pool are expected to have the correct sequence. Since we do not (but could in the future) use enzymatic error-correction (69,81), the number of assembled domain insertion variants carrying mutations is high (∼60% in this study). Owing to inefficiencies in the phosphoramidite chemistry used in oligo library synthesis, the predominant error is single-base deletions (65–67) (36% in this study). Single-base deletions result in frameshift mutations that introduce premature stop codons and therefore non-functional proteins. Our data supports this by showing that single-base deletions are strongly depleted in cells expressing surface-expressed protein. Missense mutations are rare (1–8% in this study) and considering the large number if possible combinations of missense mutations and domain insert sites (>2 million for Kir2.1) it is unlikely that the same missense mutation occurs frequently enough with the same domain insertion to influence the observed phenotype. Overall, the majority of mutations introduced by SPINE do not substantially impact downstream assay fidelity. Lastly, new chemistries and processes continuously improve oligo synthesis sequence fidelity, which can benefit SPINE in the future.

Transposon-based approaches and SPINE both operate at the nucleic acid level and can be applied to arbitrary protein coding and non-coding sequences. Some domestication is required with SPINE in the form of removing certain type IIS restriction sites (here, BsaI and BsmBI), however, in the age of relatively cheap DNA synthesis this a low barrier. While the same OLS pool can be reused to insert different domains into the same target (protein) sequence, each additional target requires a new pool. In light of these requirements, transposon-based domain insertion library construction holds a measurable cost and ease-of-use advantage, in particular, if the number of targeted proteins in large and the number of inserted domains is small. For applications that require drastically reduced bias, complete coverage, and more redundancy these advantages may be less relevant. Under such circumstances, SPINE offers distinct cost and time advantages as an approach that will likely work on the first try.

SPINE is to our knowledge the first method to enable saturated domain insertion profiling. This puts domain insertion profiling on the same level as deep mutagenesis as a method that enables experimental evolution. Like mutations, domain insertion is a major source of genetic variation that underlies natural evolution. By virtue of the programmable nature of OLS, other types of genetic variation can conceivably be combined with domain insertion, including any combination of single amino acid mutations, insertions, or deletions. This opens up the possibility to study how the effects of domain insertion depend on sequence context, i.e. epistasis (82,83). Saturated domain insertion profiling, made possible by SPINE, can be a window into the relationship between domain insertion and the emergence of new protein function and how this relationship is shaped by other evolutionary forces.

From a practical perspective, SPINE could also prove instrumental in protein engineering. Rational approaches explicitly leverage structural and functional information (84), however in the absence of such information, they reach their limits. Computational approaches (e.g. coevolution analysis (85,86)) work best in large protein families with wide-spread and homogeneously distributed similarity. Rule-based *de novo* protein design (87) is rapidly advancing, but does not capture protein dynamics that underlie allosteric transitions (88,89). Domain insertion profiling is a scalable method that can provide a window into protein evolution, dynamics and allostery. For example, we used this approach to identify sites with engineerable allostery in the Inward Rectifier K^+^ channel Kir2.1, and inserting a light-switchable domain into these sites rendered Kir2.1 activity sensitive to light (15). Perhaps other channel-based opto- and chemogenetic reagents can be constructed in a similar manner. The SPINE-generated insertion library can be used with different downstream genotype-phenotype assays other than measuring surface expression, including measuring abundance as a proxy for protein stability (20) or enzyme activity coupled to cell survival (39). This makes SPINE a broadly useful insertional mutagenesis technique that offers the opportunity to generate large-scale domain insertion datasets to exhaustively explore the critical parameters that contribute to the construction of synthetic fusion proteins, such as, the location of the insertion, linker length, and linker composition. Empirical rules for protein engineering derived from SPINE-generated datasets may be useful to improve algorithms used in rationale, computational, and rule-based approaches.

## CODE AVAILABILITY

The code for handling data from domain insertion library sequencing is available at: https://github.com/SavageLab/dipseq. The SPINE code is available at: https://github.com/schmidt-lab/SPINE.

## DATA AVAILABILITY

Source sequencing data is available in the Sequence Raw Archive (SRA- https://www.ncbi.nlm.nih.gov/sra) and the accession codes for the data are [MuA – PRJNA506141 and SPINE – PRJNA554355].

## Supplementary Material

gkz1110_Supplemental_File
