# Targeted insertional mutagenesis libraries for deep domain insertion profiling

**DOI:** 10.1093/nar/gkz1155

**Published:** 2019-12-04

**Authors:** Willow Coyote-Maestas, David Nedrud, Steffan Okorafor, Yungui He, Daniel Schmidt

**Affiliations:** 1 Dept. of Biochemistry, Molecular Biology & Biophysics, University of Minnesota, Minneapolis, MN 55455, USA; 2 Dept. of Genetics, Cell Biology & Development, University of Minnesota, Minneapolis, MN 55455, USA; 3 Dept. of Neuroscience, University of Minnesota, Minneapolis, MN 55455, USA

*Nucleic Acids Research*,2019, gkz1110, https://doi.org/10.1093/nar/gkz1110

The authors had not realized that the Github enterprise server (https://github.umn.edu/schmidt-lab/SPINE) was inaccessible to users outside their institution. The repository has been moved to the public Github: https://github.com/schmidt-lab/SPINE.

These corrections do not affect the results or conclusion of the article.

The published article has been updated.

