# Supplementary material for: Targeted insertional mutagenesis libraries for deep domain insertion profiling
Source: Nucleic Acids Res. 2019 Nov 20;48(2):e11. doi: 10.1093/nar/gkz1110 (PMC6954442; doi:10.1093/nar/gkz1110)
Supplement: gkz1110_Supplemental_File [file gkz1110_supplemental_file.pdf]

Supplemental Figure 1

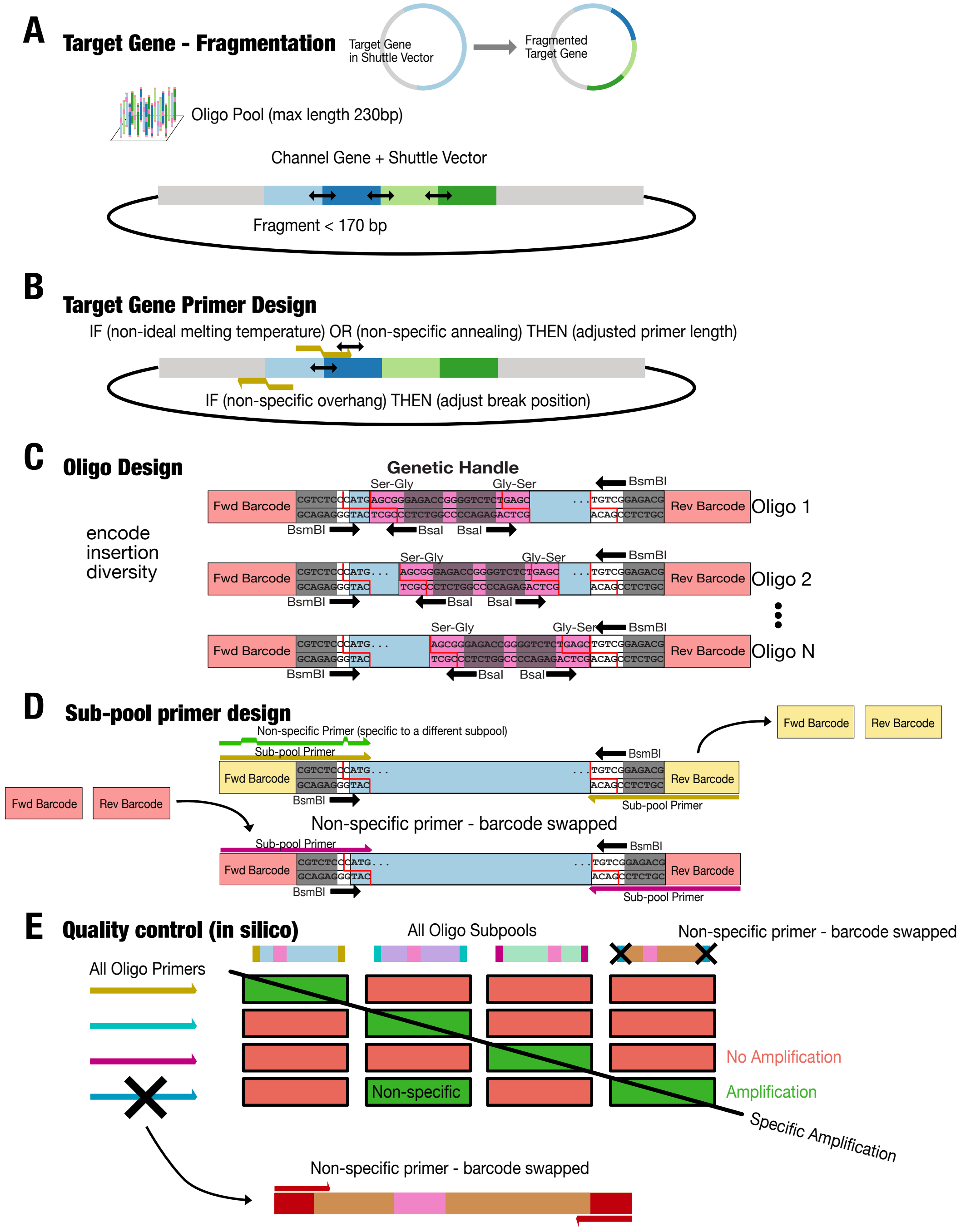

Supplemental Figure 1. In silico design of oligos and primers. (**A**) A target gene (within its shuttle vector) is fragmented such that all fragments are less than the maximum oligo size minus the barcodes, restriction enzyme sequence, and genetic handle. Fragment break sites are adjusted for unique restriction enzyme cut overhangs. (**B**) A set of gene primers are designed for each fragment for inverse PCR. These primers will amplify everything except the fragment and add an inward-facing BsmBI recognition site. (**C**) An oligo pool is designed for each fragment and within the pool an oligo is designed for each insertion position within that fragment. Each oligo consists of the fragment sequence it is replacing, sub-pool specific amplification barcodes, inward-facing BsmBI site that will match the cut site of the gene primers, and a genetic handle at every position in the gene. The genetic handle contains outward-facing BsaI recognition site for replacement with a domain of interest. (**D**) To retrieve a specific sub-pool of oligos, primers are designed based on bio-orthogonal barcodes. This amplification is made specific by swapping barcodes until unique amplification is found. (**E**) When combining the subpools from many genes, there is a chance of non-specific amplification. Quality control is performed on every oligo primer and oligo subpool for non-specific amplification. If found, the barcode is swapped for unique amplification.

Supplemental Figure 2

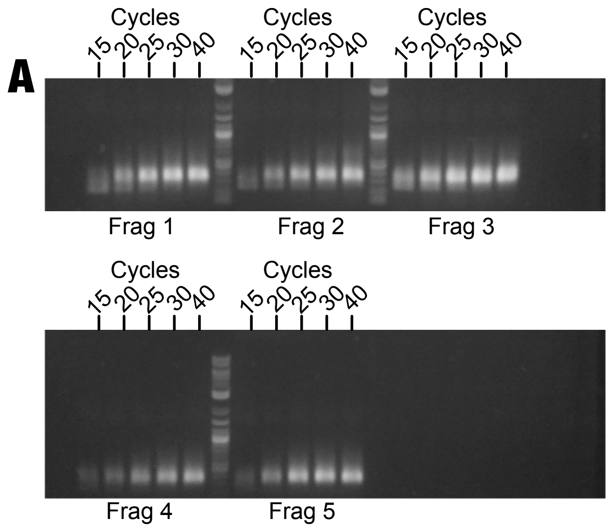

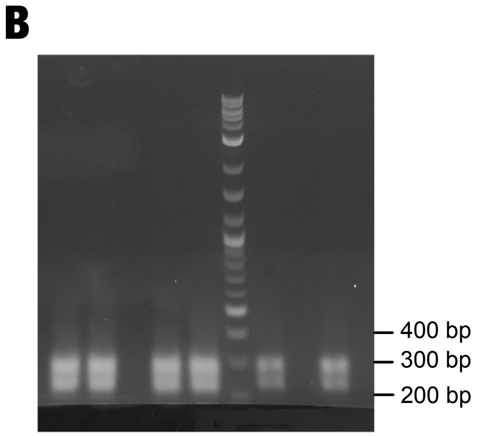

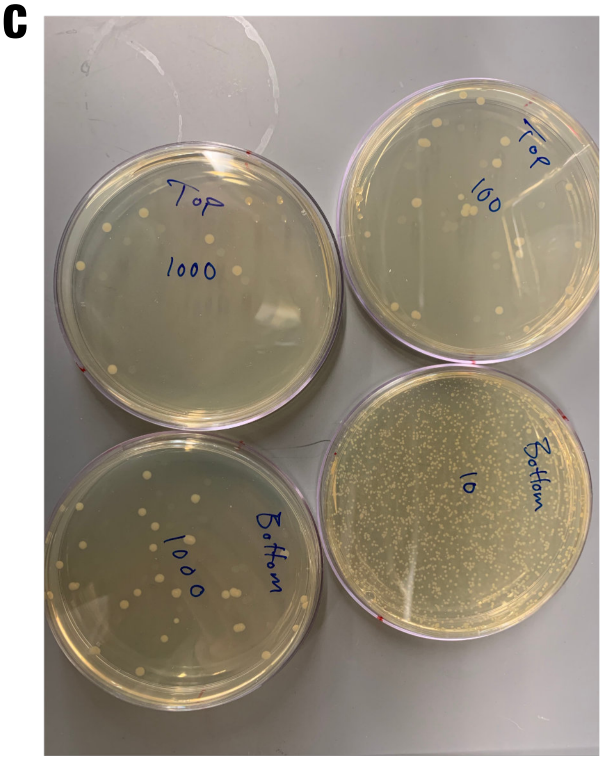

D

	Top<br>Counts(%)	Bottom<br>Counts(%)
Colonies Sequenced	25(44)	24(NA)
Perfect Colonies	11(39.8)	13(54.4)
Clones with 1 bp deletions	9(36)	5(20.8)
1 bp insertions	0(0)	0(0)
missense mutations	1(4)	0(0)
> 1 bp deletions	2(8)	3(12.6)
> 1 bp insertions	1(4)	1(4.2)
Wildtype	1(4)	2(8.4)

Supplemental Figure 2. Optimization of OLS Amplification and purification. OLS subpool amplification is sensitive to the number of PCR cycles, and overamplification can result in library bias and unintended side products. Prior to amplifying all subpools, we therefore optimized the number of PCR cycles. (**A**) Using PrimeStar GXL (Takara Clontech), we tested 15, 20, 25, 30, 40 cycles on five OLS subpools. We decided on 25 cycles as there was sufficient PCR product in all reactions. **(B)** However, we noticed that in many of the reactions there were two PCR bands with the upper bands becoming dominant with more PCR cycles. This upper band is likely an artifact of PCR due to its dominance at higher cycles and it is larger than the expected ~230 bp product. **(C)** To test which band yields better transformation efficiencies, we gel-purified both PCR products for Golden Gate cloning into the recipient backbone, and then transformed the resulting product into chemically competent E.coli cells. Transformed E.coli were plated at different dilution factor (noted on each plate along with the band used). We found that the bottom band yielded about 4x the transformation efficiency. **(D)** We also submitted 25 colonies from both transformations for Sanger Sequencing to test if the different products result in different insertion library fidelity. We found that mutation and wildtype rates were comparable. Based on these results, we suggest using 25 cycles and gel purifying the lower band after OLS amplification for the Deep Domain Insertion Profiling protocol to increase library diversity.

Supplemental Figure 3

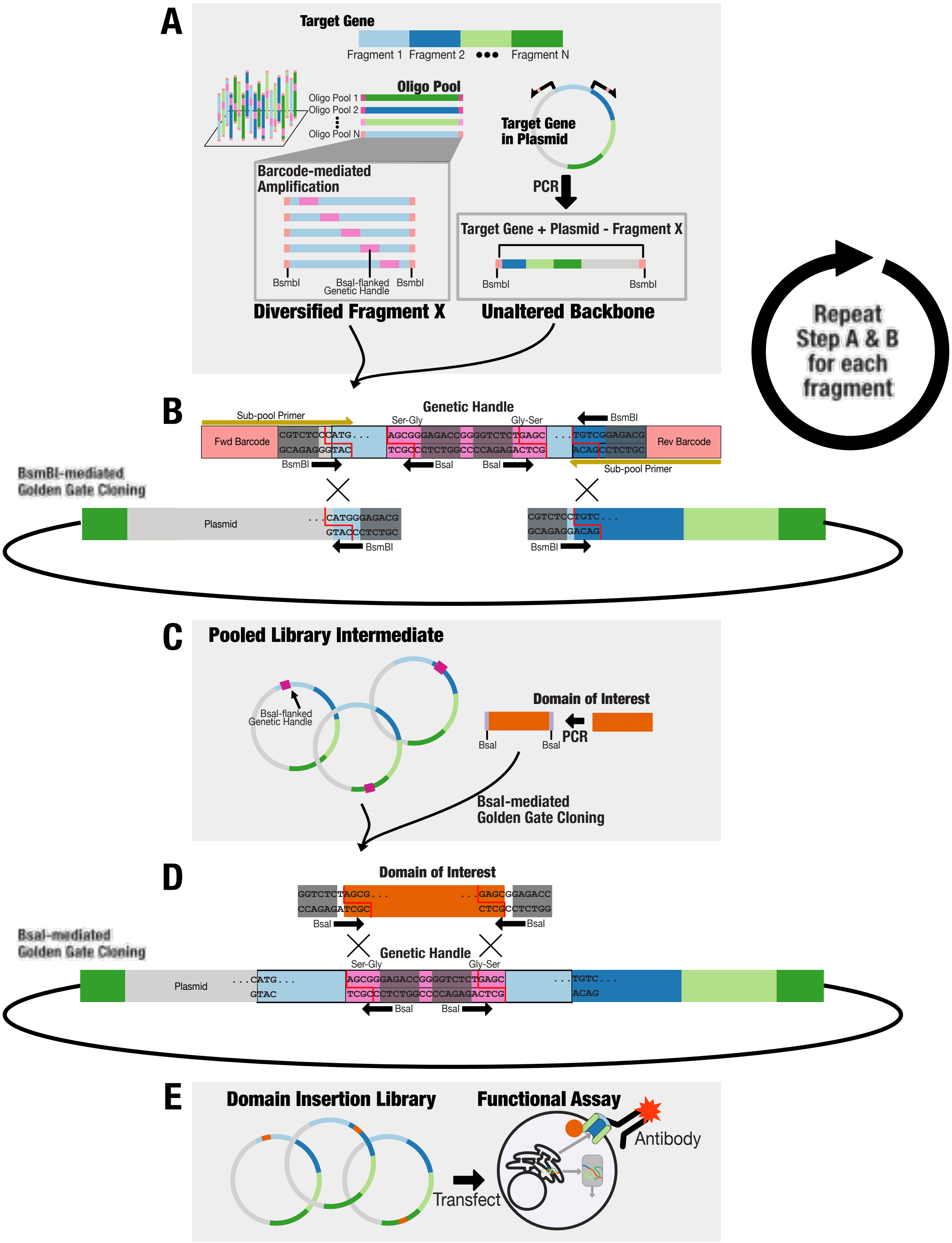

Supplemental Figure 3. Detailed library assembly. (A) Amplification of oligo subpool and inverse PCR of the target gene and shuttle vector. **(B)** These two amplicons are combined by Golden Gate assembly using BsmBI restriction enzymes. Steps **A** and **B** are repeated for every fragment in the gene. **(C)** All assembled supools (result from step **A** and b for each fragment) are mixed in equimolar ratio to yield an Intermediate Library. This mixture contains a genetic handle at every position in the gene and this can be replaced with any domain of interest by adding complementary BsaI recognition sites. **(D)** The domain of interest replaces the genetic handle in the Intermediate Library via Golden Gate cloning. **(E)** This yields the final SPINE library, which is then subjected to a functional assay.

Supplemental Figure 4

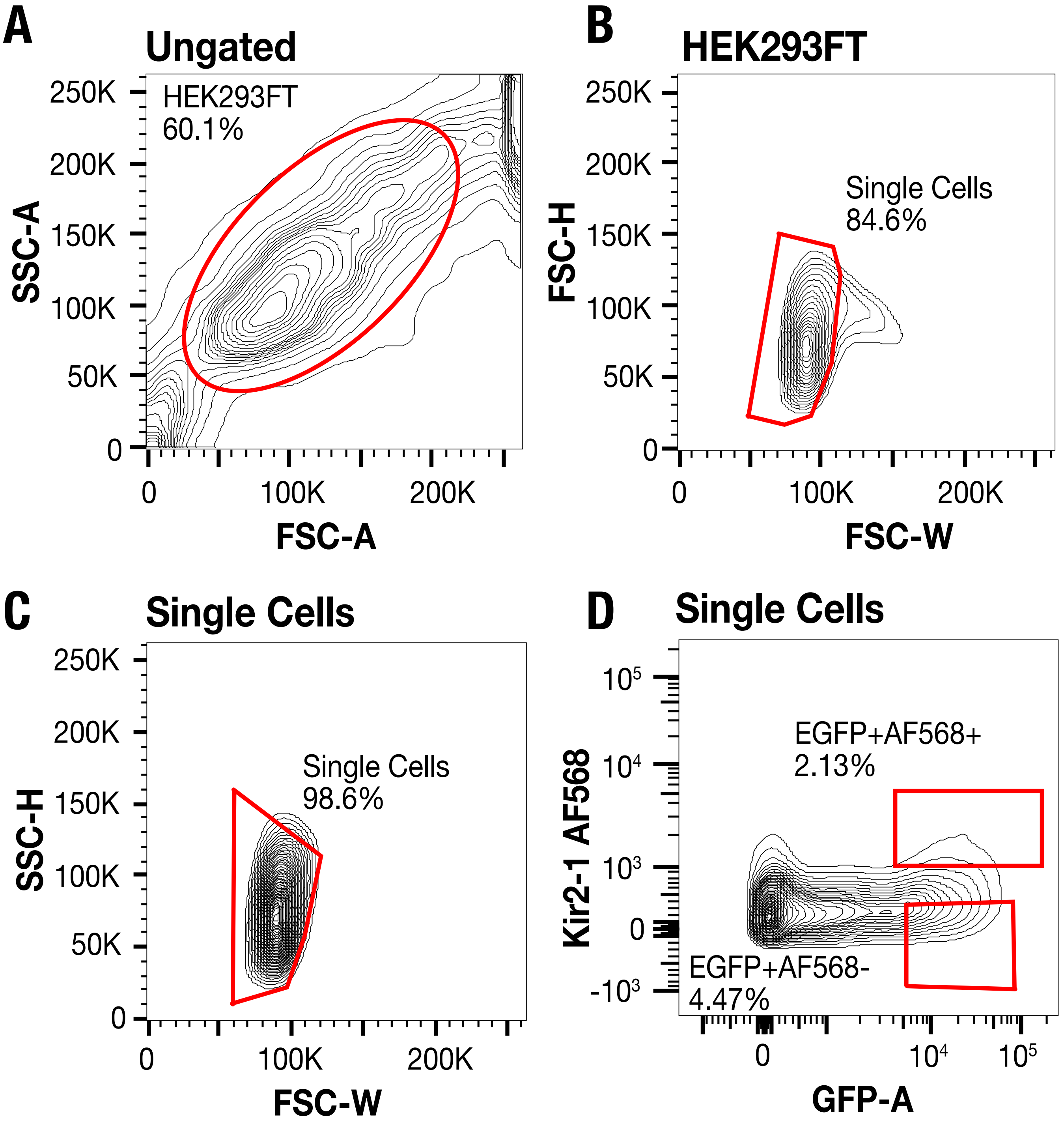

Supplementary Figure 4: Permissibility assay gating scheme. (A) Whole HEK293 cells are gated on side (SSC-A) and forward scattering (FSC-A). **(B-C)** Side scattering height (SSC-H) and forward scattering width (FSC-W) are gated to select single cells. (**D**) EGFP high / Label low and EGFP high / Label high populations are gated based on EGFP (GFP-A) and Alexa fluorophore 568 fluorescence (secondary antibody, KIR2-1 AF568).

Supplemental Figure 5

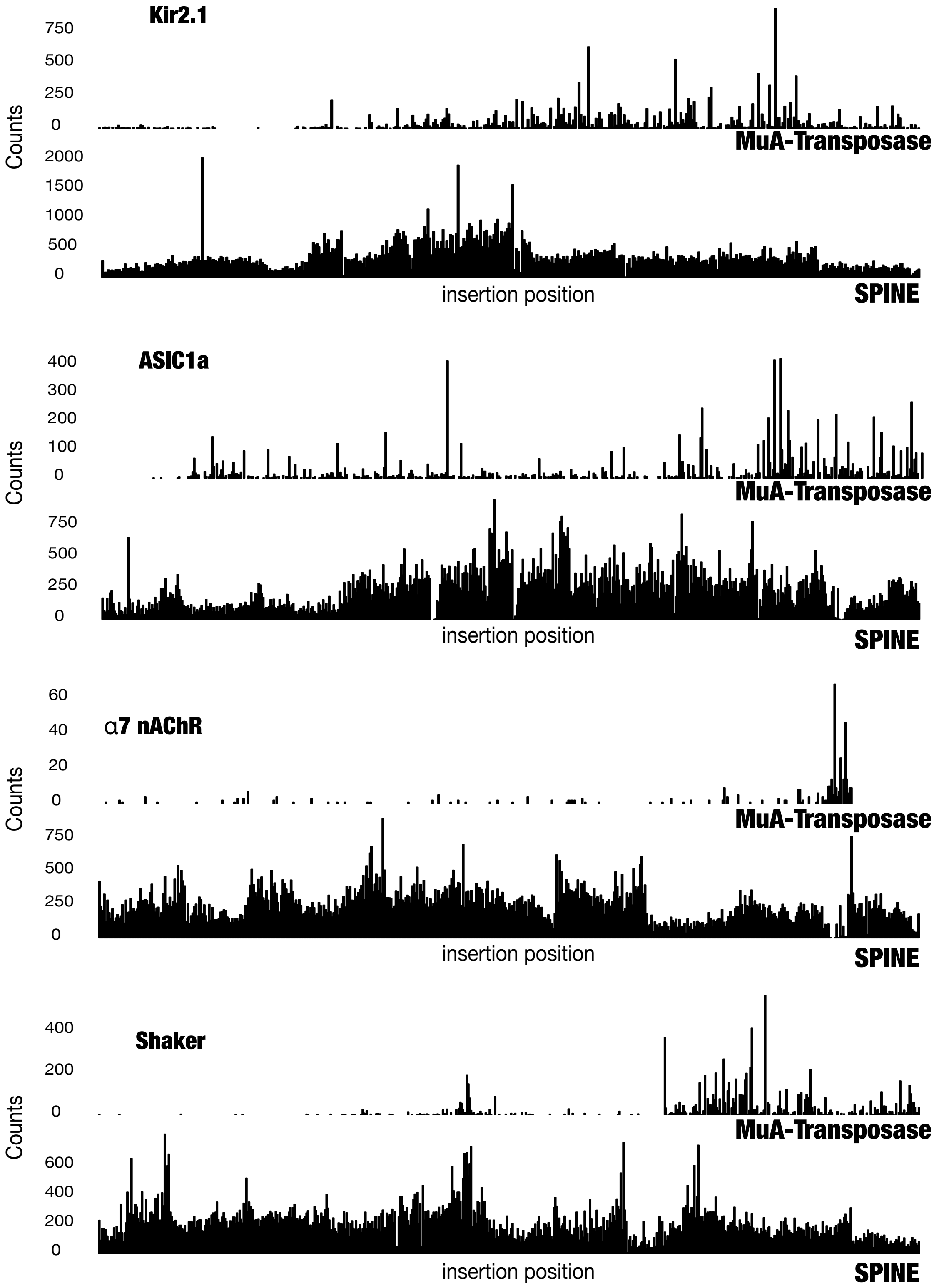

Supplemental Figure 5. Raw Reads. Comparison of MuA-transposase and SPINE-generated insertion libraries for four different ion channels. Histograms show counts of Cib81 insertions for each amino acid position.

Supplemental Figure 6

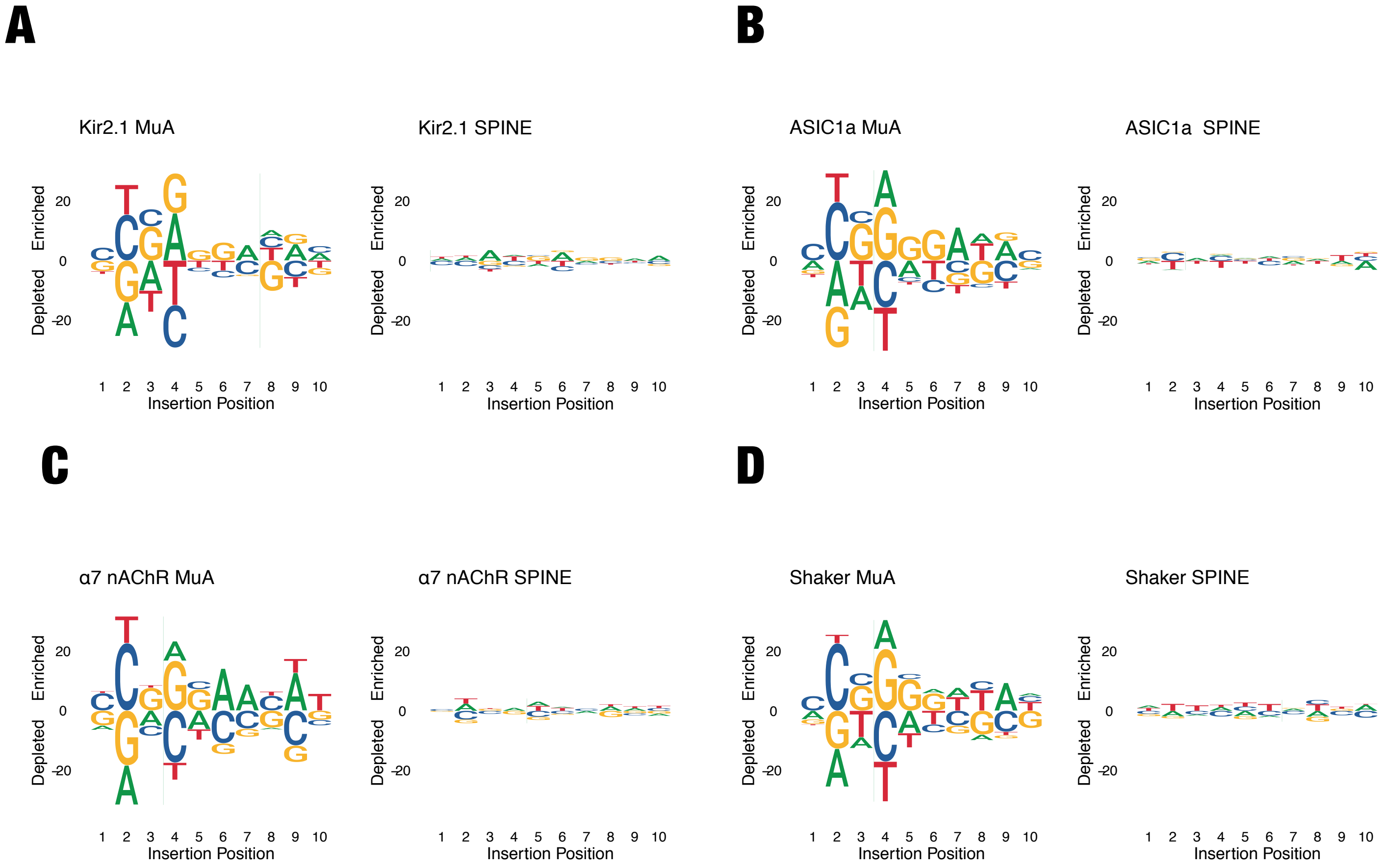

Supplemental Figure 6. Sequence Logos for Kir2.1, ASIC1a, α7 nAChR, and Shaker libraries. Nucleotide bias of insertion position was calculated for MuA- and SPINE libraries using the *ggseqlogo* R package.

Supplemental Figure 7

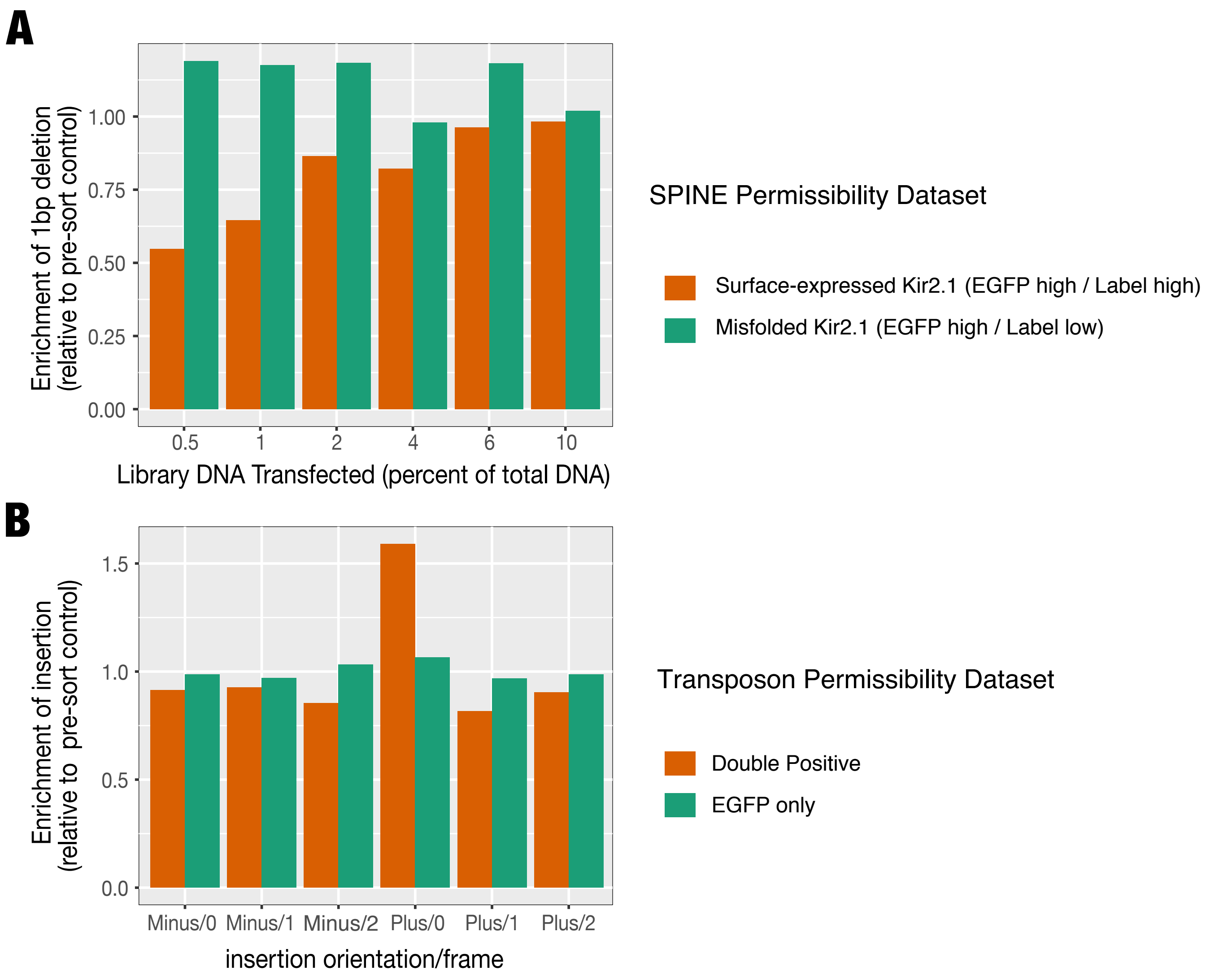

Supplemental Figure 7. Signal-to-noise stemming from transient transfection in permissibility assays. (A) 1 basepair (bp) deletion frequency was quantified for SPINE-derived permissibility datasets from sorted cells (EGFP high / Label high, orange & EGFP high / Label low, green) and normalized to unsorted cells. Each dataset represents a different percentage of insertion library DNA as part of the total DNA amount used in the transient transfection of HEK293 cells. As expected, 1 bp deletions, which cause frameshift mutations, were depleted in cells expressing putative surface-trafficked insertion variants. **(B)** Insertion rates were quantified for all MuA transposase-generated permissibility datasets from from sorted cells (EGFP high / Label high, orange & EGFP high / Label low, green) and normalized to unsorted cells. For insertion variants recovered from cell expressing surface-trafficked Kir2.1, insertions are enriched only in the correct (Plus/0) reading frame.

Supplemental Figure 8

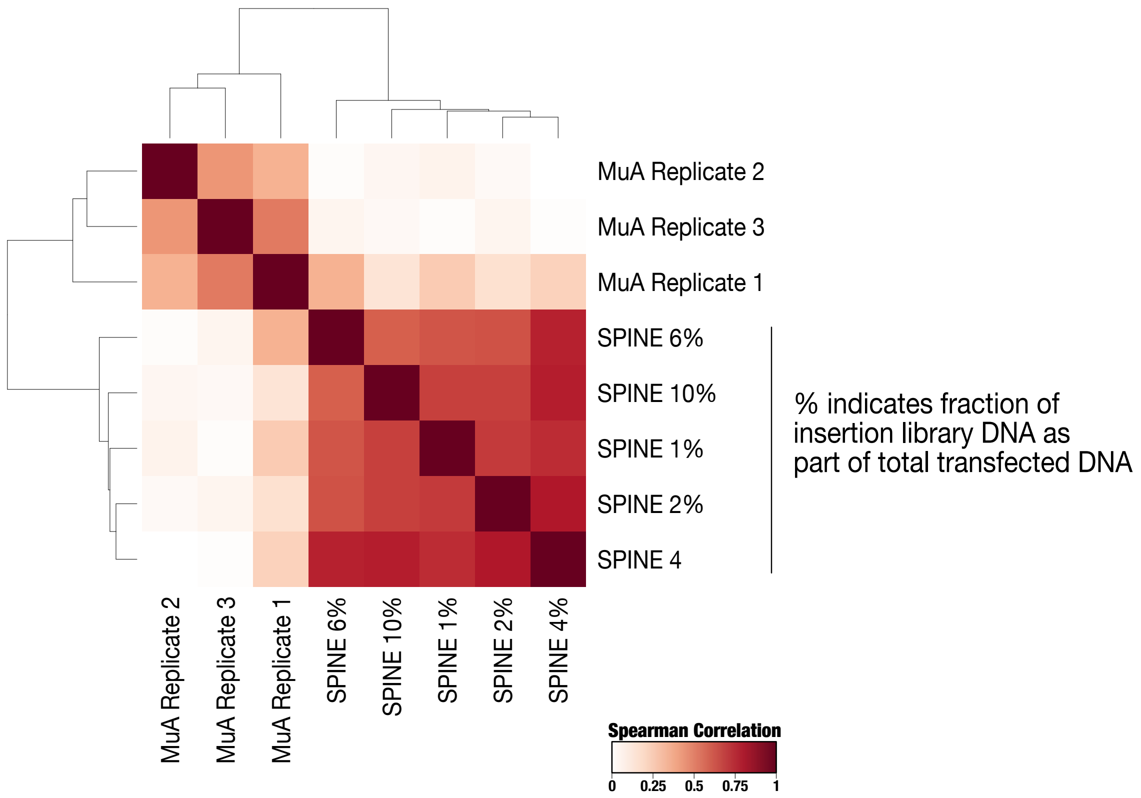

Supplemental Figure 8. Hierarchical Clustering by Spearman correlations between permissibility datasets. Spearman correlation coefficients were calculated for all Kir2.1 permissibility datasets. The three MuA permissibility datasets are derived from transient transfection with the same concentration of library DNA (1% of total transfected DNA). SPINE-derived insertion library permissibility assays are measured at 5 library DNA concentrations (1%, 2%, 4%, 6%, 10% of total DNA). Despite this increased experimental variability, OLS permissibility datasets have higher correlation between replicates. This suggest high reproducibility. Hierarchical clustering of OLS datasets shows that the low concentration replicates (1, 2, 4) and high concentration replicates (6, 10) cluster together. This may reflect the higher probability of different insertion variants assembling into mosaic tetramers at higher library transfection amount, which would decrease the signal to noise in high vs. low concentration experiments.

Supplemental Figure 9

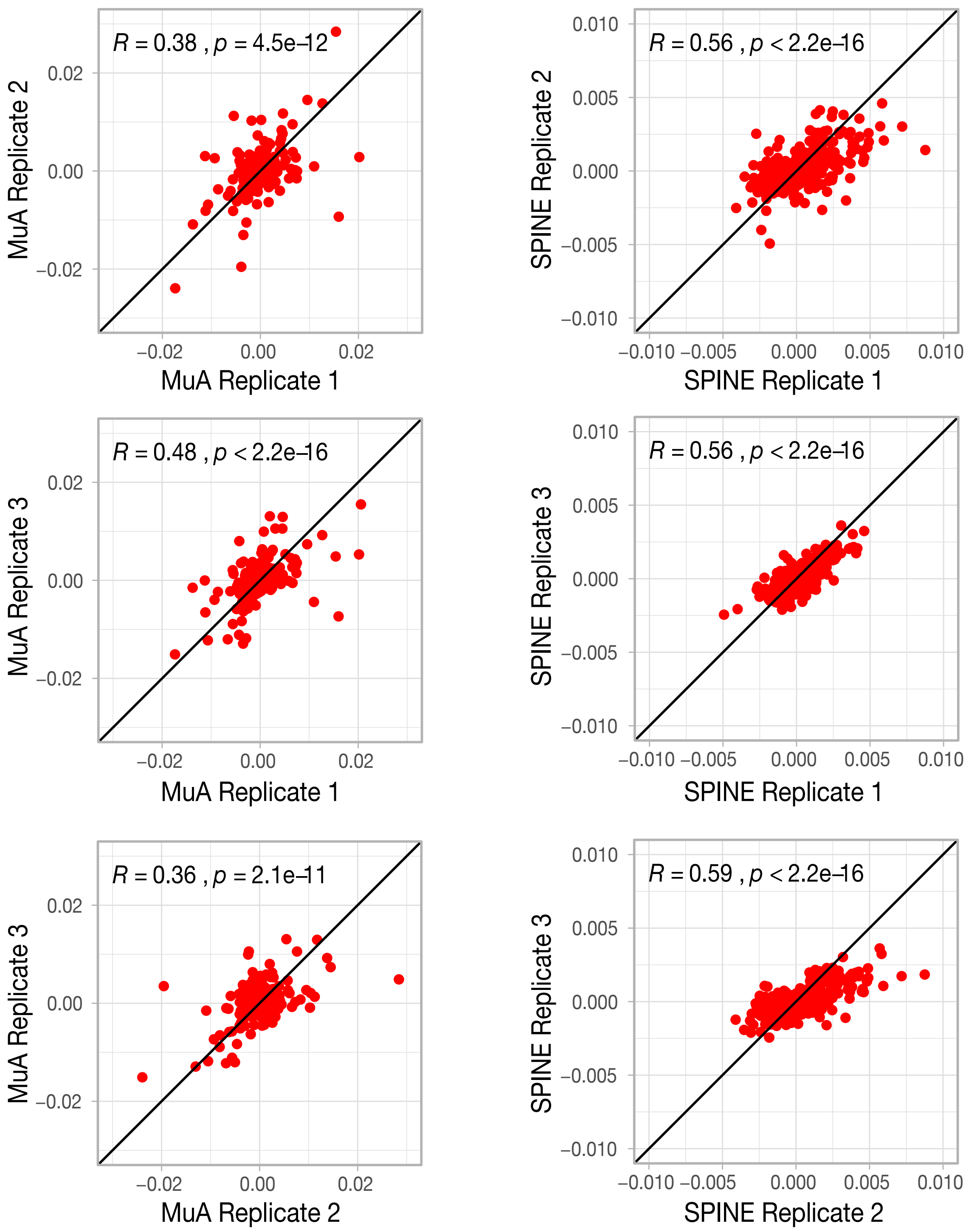

Supplemental Figure 9. Comparison of permissibility assay replicates. Scatterplots show z-scored permissibility (red points) for the indicated Kir2.1 replicates derived from MuA-generated insertion libraries (left panels) or SPINE-generated libraries (right panels). Spearman correlation coefficients (R) for each replicate pair are inset.

Supplemental Table 1

target gene	method	replicate	experiment	sample name	total reads in multiplexed subpool	samples in multiplex	estimated number of reads in subpool	read type	gene length	aligned reads	insertion directions	library size	coverage [fold]
Kir2.1	MuA	1	library	mua_kir2.1_rep1	64,453,785.00	4	16,113,446	50bp PE	1,284	105,154	2	2,568	40.9
Kir2.1	MuA	2	library	mua_kir2.1_rep2	48,756,908.00	2	24,378,454	150 PE	1,284	237,930	2	2,568	92.7
Asic1a	MuA	1	library	mua_asic1a_rep1	64,453,785.00	4	16,113,446	50bp PE	1,638	139,742	2	3,276	42.7
Asic1a	MuA	2	library	mua_asic1a_rep2	48,756,908.00	2	24,378,454	150 PE	1,638	217,153	2	3,276	66.3
nAChR α7	MuA	1	library	mua_nachra7_rep1	64,453,785.00	4	16,113,446	50bp PE	1,506	81,324	2	3,012	27.0
nAChR α7	MuA	2	library	mua_nachra7_rep2	873,258.00	1	873,258	150 PE	1,506	91,808	2	3,012	30.5
Shaker	MuA	1	library	mua_shaker_rep1	2,679,118.00	4	669,780	50bp PE	1,845	87,575	2	3,690	23.7
Shaker	MuA	2	library	mua_shaker_rep2	64,453,785.00	4	16,113,446	150 PE	1,845	158,214	2	3,690	42.9
Kir2.1	MuA	1	permissibility	mua_kir2.1_cib81_gfp_rep1	46,756,908.00	4	11,689,227	50bp PE	1,284	248,369	2	2,568	96.7
Kir2.1	MuA	2	permissibility	mua_kir2.1_cib81_gfp_rep2	47,492,020.00	4	11,873,005	50bp PE	1,284	294,046	2	2,568	114.5
Kir2.1	MuA	1	permissibility	mua_kir2.1_cib81_double_positive_rep1	47,030,083.00	4	11,757,521	50bp PE	1,284	212,110	2	2,568	82.6
Kir2.1	MuA	2	permissibility	mua_kir2.1_cib81_double_positive_rep2	47,858,073.00	4	11,964,518	50bp PE	1,284	209,716	2	2,568	81.7
Kir2.1	SPINE	1	library	ols_kir2.1_rep1	1,676,524.00	4	419,131	300 bp PE	1,284	157,580	1	428	122.7
Kir2.1	SPINE	2	library	ols_kir2.1_rep2	544,107.00	1	544,107	300 bp PE	1,284	133,308	1	428	103.8
Asic1a	SPINE	1	library	ols_asic1a	1,676,524.00	4	419,131	300 bp PE	1,638	135,463	1	546	82.7
nAChR α7	SPINE	1	library	ols_nachra7	1,676,524.00	4	419,131	300 bp PE	1,506	127,928	1	502	84.9
Shaker	SPINE	1	library	ols_shaker	1,676,524.00	4	419,131	300 bp PE	1,845	130,901	1	615	70.9
Kir2.1	SPINE	1	permissibility	ols_kir2.1_cib81_gfp_0.5	471,609.00	1	471,609	300 bp PE	1,284	211,553	1	428	164.8
Kir2.1	SPINE	1	permissibility	ols_kir2.1_cib81_gfp_1	573,447.00	1	573,447	300 bp PE	1,284	253,541	1	428	197.5
Kir2.1	SPINE	1	permissibility	ols_kir2.1_cib81_gfp_2	647,366.00	1	647,366	300 bp PE	1,284	260,941	1	428	203.2
Kir2.1	SPINE	1	permissibility	ols_kir2.1_cib81_gfp_4	572,725.00	1	572,725	300 bp PE	1,284	118,329	1	428	92.2
Kir2.1	SPINE	1	permissibility	ols_kir2.1_cib81_gfp_6	593,625.00	1	593,625	300 bp PE	1,284	234,269	1	428	182.5
Kir2.1	SPINE	1	permissibility	ols_kir2.1_cib81_gfp_10	686,950.00	1	686,950	300 bp PE	1,284	188,548	1	428	146.8
Kir2.1	SPINE	1	permissibility	ols_kir2.1_cib81_double_positive_0.5	566,107.00	1	566,107	300 bp PE	1,284	147,966	1	428	115.2
Kir2.1	SPINE	1	permissibility	ols_kir2.1_cib81_double_positive_1	511,619.00	1	511,619	300 bp PE	1,284	133,073	1	428	103.6
Kir2.1	SPINE	1	permissibility	ols_kir2.1_cib81_double_positive_2	466,423.00	1	466,423	300 bp PE	1,284	174,661	1	428	136.0
Kir2.1	SPINE	1	permissibility	ols_kir2.1_cib81_double_positive_4	549,423.00	1	549,423	300 bp PE	1,284	215,909	1	428	168.2
Kir2.1	SPINE	1	permissibility	ols_kir2.1_cib81_double_positive_6	507,334.00	1	507,334	300 bp PE	1,284	126,152	1	428	98.2
Kir2.1	SPINE	1	permissibility	ols_kir2.1_cib81_double_positive_10	597,820.00	1	597,820	300 bp PE	1,284	197,629	1	428	153.9

OLIGONUCLEOTIDE SEQUENCES

Name	Sequence
**Asic1a backbone**	
ASIC1a_backbone_1_for	ATACGTCTCTGTTCTTCTTTGCGTTTGTACCG
ASIC1a_backbone_1_rev	ATACGTCTCCCATGGTGGCcggatctg
ASIC1a_backbone_2_for	ATACGTCTCTCACGCCGGTGAACTTCTCGC
ASIC1a_backbone_2_rev	ATACGTCTCGAACAGCAAGTGACCCCAGA
ASIC1a_backbone_3_for	ATACGTCTCTTACGATCGCGCTGGCC
ASIC1a_backbone_3_rev	ATACGTCTCCGTGATAGAGATCGTTTTTAGAAACTTGG
ASIC1a_backbone_4_for	ATACGTCTCCGGCACTGGTAACGGACT
ASIC1a_backbone_4_rev	ATACGTCTCCGTAAAACTCTCGCATATTGAAGGG
ASIC1a_backbone_5_for	ATACGTCTCCGGATTCCAGACATTTGTGG
ASIC1a_backbone_5_rev	ATACGTCTCTGCCGCCTTTCATGGTTTTAAG
ASIC1a_backbone_6_for	ATACGTCTCTTGTAACTGTAGGATGGTTCACATGC
ASIC1a_backbone_6_rev	ATACGTCTCATCCGGGTGCTACACCG
ASIC1a_backbone_7_for	ATACGTCTCTAGTATGGTCAAAATTCCAAGTAAAGCC
ASIC1a_backbone_7_rev	ATACGTCTCTACAATTCTCGACAAGATATCTAGTTTCAC
ASIC1a_backbone_8_for	ATACGTCTCTCTTTTGGGAGACATAGGAGGG
ASIC1a_backbone_8_rev	ATACGTCTCTACTAAGTTCCTTACCGTACCGTG
ASIC1a_backbone_9_for	ATACGTCTCAGCTCTGTCTTTGGATGATGTAAAACG
ASIC1a_backbone_9_rev	ATACGTCTCAAAGACCTGCTATCTCGTATGCC
ASIC1a_backbone_10_for	ATACGTCTCAGCAACTAATTTTAGTCTACTGAAACAAGC
ASIC1a_backbone_10_rev	ATACGTCTCGAGCTACGCCTTTATCGGCT
**nAChRa7 backbone**	
nachra7_backbone_1_for	ATACGTCTCCCTGAGCCTCCTGC
nachra7_backbone_1_rev	ATACGTCTCGCATGTGGCcggatct
nachra7_backbone_2_for	ATACGTCTCTCTTCTCTATAACAGTGCTGATGAGCG
nachra7_backbone_2_rev	ATACGTCTCTCAGGGAGAAGTAGACGGTG
nachra7_backbone_3_for	ATACGTCTCTGGGTCCTGGTCTTACGGAG
nachra7_backbone_3_rev	ATACGTCTCGAAGAATGTCTGGTTTCCAAATCTGG
nachra7_backbone_4_for	ATACGTCTCGACCATGCGCCGCAGG
nachra7_backbone_4_rev	ATACGTCTCACCCAAACTTCAGTTTGCAGTGC
nachra7_backbone_5_for	ATACGTCTCTGAGATCATGCCCGCAACA
nachra7_backbone_5_rev	ATACGTCTCTGGTCACTGTGAAGGTGACATCG
nachra7_backbone_6_for	ATACGTCTCGAACTGGTGCGCGTGGT
nachra7_backbone_6_rev	ATACGTCTCTCTCAGCCACGAGCAGCA
nachra7_backbone_7_for	ATACGTCTCGGACGGCGTGCACTGT
nachra7_backbone_7_rev	ATACGTCTCAGTTCAGAAGGATGACTCTGGTC
nachra7_backbone_8_for	ATACGTCTCCCGCTGCCAGGACGAA
nachra7_backbone_8_rev	ATACGTCTCCGTCCAGGCCGCG
nachra7_backbone_9_for	ATACGTCTCGGCTGCTTCATCTGCAGTC
nachra7_backbone_9_rev	ATACGTCTCAGCGGAAGCGGTTGGC
**Shaker backbone**	
Shaker_backbone_1_for	ATACGTCTCGAGAGTCGTGATCAACGTGAGTG
Shaker_backbone_1_rev	ATACGTCTCCCATGGTGGCcggatctg
Shaker_backbone_2_for	ATACGTCTCTCTCTATTACTATCAATCAGGAGGGC
Shaker_backbone_2_rev	ATACGTCTCCTCTCTCACAGAAGTCGTGGTCA
Shaker_backbone_3_for	ATACGTCTCAAAACAGCGGAAAGTGTGGCT
Shaker_backbone_3_rev	ATACGTCTCAGAGAATTGCGTCAAAACTAGGTCG
Shaker_backbone_4_for	ATACGTCTCCACCAAAATTGAGGAGGACGAAG
Shaker_backbone_4_rev	ATACGTCTCGTTTTTCATTGTCGGGCAATGGC
Shaker_backbone_5_for	ATACGTCTCCACCAAAATTGAGGAGGACGAAG
Shaker_backbone_5_rev	ATACGTCTCGTTTTTCATTGTCGGGCAATGGC
Shaker_backbone_6_for	ATACGTCTCTATACCTTATTTTATTACCTTGGCTACAGT
Shaker_backbone_6_rev	ATACGTCTCTGGTGCCGTTGGTAGTTGT
Shaker_backbone_7_for	ATACGTCTCACACTCCAAGGGGCTCCA
Shaker_backbone_7_rev	ATACGTCTCGTATAATTGCTATAATATCAATCACGTTCATTAC
Shaker_backbone_8_for	ATACGTCTCGTGGGCCGTCGTGACT
Shaker_backbone_8_rev	ATACGTCTCAGTGTCGAGACAACTTAAAAATCCTGA
Shaker_backbone_9_for	ATACGTCTCGGAAGAAATGCAGAGTCAGAACTTCA
Shaker_backbone_9_rev	ATACGTCTCCCCACCAGAAAGCGTCAGG
Shaker_backbone_10_for	ATACGTCTCTCGGTCTGCAGTGGCCC
Shaker_backbone_10_rev	ATACGTCTCCTTCCTGGTCAGTCTCTCGATGG
Shaker_backbone_11_for	ATACGTCTCTAAGCAGCAGCAACAACAAACCC
Shaker_backbone_11_rev	ATACGTCTCACCGACCAGGGTGTGTTTC
Shaker_backbone_12_for	ATACGTCTCTGCTGCTTCATCTGCAGTCAATGG
Shaker_backbone_12_rev	ATACGTCTCGCTTAAAACCGTTCTGTTGTTGCT
**Kir2.1 backbone**	
kir2.1_backbone_1_for	ATACGTCTCTAATGTGCAGTTCATAAACGTGGG
kir2.1_backbone_1_rev	ATACGTCTCCCATGGTGGCcggatc
kir2.1_backbone_2_for	ATACGTCTCTGGAGATTTGGACACCGATTATAAAG
kir2.1_backbone_2_rev	ATACGTCTCCATTACAGTGGCCATCTTTCTTGAC
kir2.1_backbone_3_for	ATACGTCTCCGTTTTCATGGTTGTCTTTCAATCA
kir2.1_backbone_3_rev	ATACGTCTCCTCCATGCAACAAAGCGATCAAC
kir2.1_backbone_4_for	ATACGTCTCGAGGGTGGGCAATCTGAGAA
kir2.1_backbone_4_rev	ATACGTCTCAAACGGCTATTGGGCATTCG
kir2.1_backbone_5_for	ATACGTCTCAATCACCATCGTCCACGAGA
kir2.1_backbone_5_rev	ATACGTCTCCCCTCCACATGAGGCACAG
kir2.1_backbone_6_for	ATACGTCTCCCTTTGGGGCCATCGCT
kir2.1_backbone_6_rev	ATACGTCTCTGATTGGGCTAACGAGAAAGATCC
kir2.1_backbone_7_for	ATACGTCTCCTGTTACGAGAACGAAGTAGCTCT
kir2.1_backbone_7_rev	ATACGTCTCAAAGGATTTCATTGGCAAGATAACTACT
kir2.1_backbone_8_for	ATACGTCTCCGCTGCTTCATCTGCAGTCAATG
kir2.1_backbone_8_rev	ATACGTCTCAACAGAAAGAGTTTGCGTTAGACAGA
**Asic1a fragments**	
ASIC1a_fragment_1_for	CGATCACCACGTCTCCCATG
ASIC1a_fragment_1_rev	ATTCCCTAACTCGTCTCCGAAC
ASIC1a_fragment_2_for	TAGATACTAGCTCGTCTCCTGTT
ASIC1a_fragment_2_rev	CCGCCGTCTCCCGTG
ASIC1a_fragment_3_for	GCGTCTTTTCGTCTCCTCAC
ASIC1a_fragment_3_rev	CACCCATTACCGTCTCCCGTA
ASIC1a_fragment_4_for	CACTTGAGGTCCGTCTCCTTAC
ASIC1a_fragment_4_rev	GGTGCTCGTCTCCTGCC
ASIC1a_fragment_5_for	TGTTCCGTCTCCCGGC
ASIC1a_fragment_5_rev	ACTTATGGCCGTCTCCATCC
ASIC1a_fragment_6_for	ACCTAATCCGTCTCCCGGA
ASIC1a_fragment_6_rev	AGGTACTGACTCGTCTCCTACA
ASIC1a_fragment_7_for	GATACTTAACGTCGTCTCCTTGT
ASIC1a_fragment_7_rev	AGGTTCTCCCGTCTCCTACT
ASIC1a_fragment_8_for	AGCGGGATCGTCTCCTAGT
ASIC1a_fragment_8_rev	CGCATGGACCGTCTCCAAAG
ASIC1a_fragment_9_for	CGAGTACTCCCGTCTCCTCTT
ASIC1a_fragment_9_rev	ACGGTCTCGTCTCCGAGC
ASIC1a_fragment_10_for	TGTACCCCGTCTCCAGCT
ASIC1a_fragment_10_rev	CGCGAGACGTCTCCTTGC
**nAChRa7 fragments**	
nachra7_fragment_1_for	TGGTCATGCGTCTCCCATG
nachra7_fragment_1_rev	GATACTCAGACGTCTCCTCAG
nachra7_fragment_2_for	GTGGGTACGTCTCCCCTG
nachra7_fragment_2_rev	CGGATATAGCCGTCTCCGAAG
nachra7_fragment_3_for	CGACGGGCGTCTCCTCTT
nachra7_fragment_3_rev	GGCCAACGTCTCCACCC
nachra7_fragment_4_for	CATGTTTAGCGTCTCCTGGG
nachra7_fragment_4_rev	CCTCCCCGTCTCCTGGT
nachra7_fragment_5_for	GTACATGAACGTCTCCGACC
nachra7_fragment_5_rev	CCTCAGCGCGTCTCCTCTC
nachra7_fragment_6_for	GCGAGAGGCGTCTCCTGAG
nachra7_fragment_6_rev	GCTGTGCACCGTCTCCAGTT
nachra7_fragment_7_for	CCGATAGCCGTCTCCGAAC
nachra7_fragment_7_rev	GCCTGTCGTCTCCCGTC
nachra7_fragment_8_for	TTATAATCATCCGTCTCCGGAC
nachra7_fragment_8_rev	GCTCGCGTCTCCAGCG
nachra7_fragment_9_for	CCCTTCGTCTCCCCGC
nachra7_fragment_9_rev	GTCGGCGTCTCCCAGC
**Shaker fragments**	
Shaker_backbone_1_for	TCCTCGATTCGTCTCCCATG
Shaker_backbone_1_rev	ACATGCGCGTCTCCCTCT
Shaker_backbone_2_for	GCTTAACGCATCGTCTCCGAGA
Shaker_backbone_2_rev	CAGCCTACCGTCTCCAGAG
Shaker_backbone_3_for	GGGCGTTCGTCTCCTCTC
Shaker_backbone_3_rev	CAGTTCGGCGTCTCCGTTT
Shaker_backbone_4_for	CGCGTGAGCGTCTCCAAAA
Shaker_backbone_4_rev	CGCCGTTCGTCTCCTGGT
Shaker_backbone_5_for	ATATCCGCGTCTCCCACC
Shaker_backbone_5_rev	CGTTCGGTCGTCTCCGTAT
Shaker_backbone_6_for	TGTCGCTCCGTCTCCTATAAGC
Shaker_backbone_6_rev	TAGCTGCTCGTCTCCAGTG
Shaker_backbone_7_for	TGTCGCTCCGTCTCCTATAAGC
Shaker_backbone_7_rev	TAGCTGCTCGTCTCCAGTG
Shaker_backbone_8_for	CGGTAAGACGTCTCCACAC
Shaker_backbone_8_rev	AGCTGCGTCTCCCCCA
Shaker_backbone_9_for	CTCACGCGTCTCCGTGG
Shaker_backbone_9_rev	GCGCCCGTCTCCCTTC
Shaker_backbone_10_for	GCTTAGGACGTCTCCGGAA
Shaker_backbone_10_rev	AGTATCTCCGTCTCCACCG
Shaker_backbone_11_for	GCTTTTCTCGTCTCCTCGG
Shaker_backbone_11_rev	GCATCCCGTCTCCGCTT
Shaker_backbone_12_for	ACCATTCTATGGCGTCTCCTAAG
Shaker_backbone_12_rev	AACAGCCGTCTCCCAGC
**Kir2.1 fragments**	
kir2.1_fragment_1_for	CGATCACCACGTCTCCCATG
kir2.1_fragment_1_rev	CGCCATACCGTCTCCCATT
kir2.1_fragment_2_for	CGTCTGTGACGTCTCCTAATAGC
kir2.1_fragment_2_rev	GCACCCGTCTCCCTCC
kir2.1_fragment_3_for	GCGTATTAGAACGTCTCCTGGA
kir2.1_fragment_3_rev	TCTCACCATCCGTCTCCAAAC
kir2.1_fragment_4_for	TGAGAGGTACGTCTCCCGTT
kir2.1_fragment_4_rev	CGCACTCGTCTCCCCCT
kir2.1_fragment_5_for	GCTATTCCCGTCTCCGAGG
kir2.1_fragment_5_rev	CGAGTCGCACGTCTCCTGAT
kir2.1_fragment_6_for	ATTTCCGCTCTCGTCTCCAATC
kir2.1_fragment_6_rev	TTCGTACCCTCGTCTCCAAAG

kir2.1_fragment_7_for	CTAAATAGTGCTCGTCTCCCCTT
kir2.1_fragment_7_rev	CCTCGGGCGTCTCCAACA
kir2.1_fragment_8_for	TCTAAGTGGCGTCTCCCTGT
kir2.1_fragment_8_rev	TTCCGCGTCTCCCAGC
**Sequencing primers**	
CMV_for	ggcaccaaaatcaacggg
P2A_rev	gcagagCGTCTCTTGGTCCTGGATTTTCCTCCACATCACC
**Antibiotic Cassette Primers**	
chlor_ols_bsai_for	agtctgAGCGGGAGACCggatcgatccttttcgaccgaataaatacctg
chlor_ols_bsai_rev	gtacaGCTCAGAGACCcggatcgatcctatcgtcaattattacctc
cib81 primers	
cib81_bsai_for	ATAGGTCTCGAGCGGGATGAATGGGGCAATCGGC
cib81_bsai_rev	TATGGTCTCCGCTCCCACTTATGCTGAGTCTGCTATCACCC
**Construct Cloning Primers**	
kir2.1_insert_patt_hindiii_for	GCAGCTaagcttGCATgaagacatccgGCCACCATGGGA
asic1a_insert_patt_hindiii_for	GCAGCTaagcttGCATgaagacatccgATTGGTCCTGGATTTTCCTCCACATCA
nachra7_insert_patt_hindiii_for	GCAGCTaagcttGCATgaagacatccgGCCACCATGGAAC
shaker_insert_patt_hindiii_for	GCAGCTaagcttGCATgaagacAtccgGCCACCATGGC
kir2.1_insert_cmv_bsmbi_for	gacatacCGTCTCctccgGCCACCATGGGat
asic1a_insert_cmv_bsmbi_for	gacatacCGTCTcatccgGCCACCATGGAA
nachra7_insert_cmv_bsmbi_for	gacatacCGTCTcatccgGCCACCATGGAAC
shaker_insert_cmv_bsmbi_for	gacatacCGTCTCAtccgGCCACCATG
cmv_backbone_bsmbi_for	GACAGCAGCGTCTCgACCAatggtgagcaagggcgag
cmv_backbone_bsmbi_rev	acgtcagCGTCTCAcggatctgacggttcactaaacc
patt_backbone_hindiii_for	GCATCAAGCTTtcctctacgccggacg
patt_backbone_bamhi_rev	acgatGGATCCacggttaagatccggct
p2a_all_bsmbi_rev	gcagagCGTCTCTTGGTCCTGGATTTTCCTCCACATCACC
p2a_patt_bamhi_rev	GCGGATcctaggTGATgaagacATTGGTCCTGGATTTTCCTCCA
**OLS encoded oligos**	
shaker_Frag3-168_insertion3	TCCTCGATTCGTCTCCCATGAGCGGGAGACCGGGGTCTCTGAGCGCCGCCGTGGCACTGCGAGAACAACAGCTCCAACGAAATAGTCTGGATGGATACGGTTCACTGCCTAAACTGTCTAGCCAAGACGAAGAAGGTGGCGCCGGCCATGGCTTCGGTGGGGGCCCTCAGCATTTCGAGCCTATTCCCCATGACCACGACTTCTGTGAGAGAGGGAGACGCGCATGTTTT
shaker_Frag3-168_insertion6	TCCTCGATTCGTCTCCCATGGCCAGCGGGAGACCGGGGTCTCTGAGCGCCGTGGCACTGCGAGAACAACAGCTCCAACGAAATAGTCTGGATGGATACGGTTCACTGCCTAAACTGTCTAGCCAAGACGAAGAAGGTGGCGCCGGCCATGGCTTCGGTGGGGGCCCTCAGCATTTCGAGCCTATTCCCCATGACCACGACTTCTGTGAGAGAGGGAGACGCGCATGTTTT
shaker_Frag3-168_insertion9	TCCTCGATTCGTCTCCCATGGCCGCCAGCGGGAGACCGGGGTCTCTGAGCGTGGCACTGCGAGAACAACAGCTCCAACGAAATAGTCTGGATGGATACGGTTCACTGCCTAAACTGTCTAGCCAAGACGAAGAAGGTGGCGCCGGCCATGGCTTCGGTGGGGGCCCTCAGCATTTCGAGCCTATTCCCCATGACCACGACTTCTGTGAGAGAGGGAGACGCGCATGTTTT
shaker_Frag3-168_insertion12	TCCTCGATTCGTCTCCCATGGCCGCCGTGAGCGGGAGACCGGGGTCTCTGAGCGCACTGCGAGAACAACAGCTCCAACGAAATAGTCTGGATGGATACGGTTCACTGCCTAAACTGTCTAGCCAAGACGAAGAAGGTGGCGCCGGCCATGGCTTCGGTGGGGGCCCTCAGCATTTCGAGCCTATTCCCCATGACCACGACTTCTGTGAGAGAGGGAGACGCGCATGTTTT
shaker_Frag3-168_insertion15	TCCTCGATTCGTCTCCCATGGCCGCCGTGGCAAGCGGGAGACCGGGGTCTCTGAGCCTGCGAGAACAACAGCTCCAACGAAATAGTCTGGATGGATACGGTTCACTGCCTAAACTGTCTAGCCAAGACGAAGAAGGTGGCGCCGGCCATGGCTTCGGTGGGGGCCCTCAGCATTTCGAGCCTATTCCCCATGACCACGACTTCTGTGAGAGAGGGAGACGCGCATGTTTT
shaker_Frag3-168_insertion18	TCCTCGATTCGTCTCCCATGGCCGCCGTGGCACTGAGCGGGAGACCGGGGTCTCTGAGCCGAGAACAACAGCTCCAACGAAATAGTCTGGATGGATACGGTTCACTGCCTAAACTGTCTAGCCAAGACGAAGAAGGTGGCGCCGGCCATGGCTTCGGTGGGGGCCCTCAGCATTTCGAGCCTATTCCCCATGACCACGACTTCTGTGAGAGAGGGAGACGCGCATGTTTT
shaker_Frag3-168_insertion21	TCCTCGATTCGTCTCCCATGGCCGCCGTGGCACTGCGAAGCGGGAGACCGGGGTCTCTGAGCGAACAACAGCTCCAACGAAATAGTCTGGATGGATACGGTTCACTGCCTAAACTGTCTAGCCAAGACGAAGAAGGTGGCGCCGGCCATGGCTTCGGTGGGGGCCCTCAGCATTTCGAGCCTATTCCCCATGACCACGACTTCTGTGAGAGAGGGAGACGCGCATGTTTT
shaker_Frag3-168_insertion24	TCCTCGATTCGTCTCCCATGGCCGCCGTGGCACTGCGAGAAAGCGGGAGACCGGGGTCTCTGAGCCAACAGCTCCAACGAAATAGTCTGGATGGATACGGTTCACTGCCTAAACTGTCTAGCCAAGACGAAGAAGGTGGCGCCGGCCATGGCTTCGGTGGGGGCCCTCAGCATTTCGAGCCTATTCCCCATGACCACGACTTCTGTGAGAGAGGGAGACGCGCATGTTTT
shaker_Frag3-168_insertion27	TCCTCGATTCGTCTCCCATGGCCGCCGTGGCACTGCGAGAACAAAGCGGGAGACCGGGGTCTCTGAGCCAGCTCCAACGAAATAGTCTGGATGGATACGGTTCACTGCCTAAACTGTCTAGCCAAGACGAAGAAGGTGGCGCCGGCCATGGCTTCGGTGGGGGCCCTCAGCATTTCGAGCCTATTCCCCATGACCACGACTTCTGTGAGAGAGGGAGACGCGCATGTTTT
shaker_Frag3-168_insertion30	TCCTCGATTCGTCTCCCATGGCCGCCGTGGCACTGCGAGAACAACAGAGCGGGAGACCGGGGTCTCTGAGCCTCCAACGAAATAGTCTGGATGGATACGGTTCACTGCCTAAACTGTCTAGCCAAGACGAAGAAGGTGGCGCCGGCCATGGCTTCGGTGGGGGCCCTCAGCATTTCGAGCCTATTCCCCATGACCACGACTTCTGTGAGAGAGGGAGACGCGCATGTTTT
shaker_Frag3-168_insertion33	TCCTCGATTCGTCTCCCATGGCCGCCGTGGCACTGCGAGAACAACAGCTCAGCGGGAGACCGGGGTCTCTGAGCCAACGAAATAGTCTGGATGGATACGGTTCACTGCCTAAACTGTCTAGCCAAGACGAAGAAGGTGGCGCCGGCCATGGCTTCGGTGGGGGCCCTCAGCATTTCGAGCCTATTCCCCATGACCACGACTTCTGTGAGAGAGGGAGACGCGCATGTTTT
shaker_Frag3-168_insertion36	TCCTCGATTCGTCTCCCATGGCCGCCGTGGCACTGCGAGAACAACAGCTCCAAAGCGGGAGACCGGGGTCTCTGAGCCGAAATAGTCTGGATGGATACGGTTCACTGCCTAAACTGTCTAGCCAAGACGAAGAAGGTGGCGCCGGCCATGGCTTCGGTGGGGGCCCTCAGCATTTCGAGCCTATTCCCCATGACCACGACTTCTGTGAGAGAGGGAGACGCGCATGTTTT
shaker_Frag3-168_insertion39	TCCTCGATTCGTCTCCCATGGCCGCCGTGGCACTGCGAGAACAACAGCTCCAACGAAGCGGGAGACCGGGGTCTCTGAGCAATAGTCTGGATGGATACGGTTCACTGCCTAAACTGTCTAGCCAAGACGAAGAAGGTGGCGCCGGCCATGGCTTCGGTGGGGGCCCTCAGCATTTCGAGCCTATTCCCCATGACCACGACTTCTGTGAGAGAGGGAGACGCGCATGTTTT
shaker_Frag3-168_insertion42	TCCTCGATTCGTCTCCCATGGCCGCCGTGGCACTGCGAGAACAACAGCTCCAACGAAATAGCGGGAGACCGGGGTCTCTGAGCAGTCTGGATGGATACGGTTCACTGCCTAAACTGTCTAGCCAAGACGAAGAAGGTGGCGCCGGCCATGGCTTCGGTGGGGGCCCTCAGCATTTCGAGCCTATTCCCCATGACCACGACTTCTGTGAGAGAGGGAGACGCGCATGTTTT
shaker_Frag3-168_insertion45	TCCTCGATTCGTCTCCCATGGCCGCCGTGGCACTGCGAGAACAACAGCTCCAACGAAATAGTAGCGGGAGACCGGGGTCTCTGAGCCTGGATGGATACGGTTCACTGCCTAAACTGTCTAGCCAAGACGAAGAAGGTGGCGCCGGCCATGGCTTCGGTGGGGGCCCTCAGCATTTCGAGCCTATTCCCCATGACCACGACTTCTGTGAGAGAGGGAGACGCGCATGTTTT
shaker_Frag3-168_insertion48	TCCTCGATTCGTCTCCCATGGCCGCCGTGGCACTGCGAGAACAACAGCTCCAACGAAATAGTCTGAGCGGGAGACCGGGGTCTCTGAGCGATGGATACGGTTCACTGCCTAAACTGTCTAGCCAAGACGAAGAAGGTGGCGCCGGCCATGGCTTCGGTGGGGGCCCTCAGCATTTCGAGCCTATTCCCCATGACCACGACTTCTGTGAGAGAGGGAGACGCGCATGTTTT
shaker_Frag3-168_insertion51	TCCTCGATTCGTCTCCCATGGCCGCCGTGGCACTGCGAGAACAACAGCTCCAACGAAATAGTCTGGATAGCGGGAGACCGGGGTCTCTGAGCGGATACGGTTCACTGCCTAAACTGTCTAGCCAAGACGAAGAAGGTGGCGCCGGCCATGGCTTCGGTGGGGGCCCTCAGCATTTCGAGCCTATTCCCCATGACCACGACTTCTGTGAGAGAGGGAGACGCGCATGTTTT
shaker_Frag3-168_insertion54	TCCTCGATTCGTCTCCCATGGCCGCCGTGGCACTGCGAGAACAACAGCTCCAACGAAATAGTCTGGATGGAAGCGGGAGACCGGGGTCTCTGAGCTACGGTTCACTGCCTAAACTGTCTAGCCAAGACGAAGAAGGTGGCGCCGGCCATGGCTTCGGTGGGGGCCCTCAGCATTTCGAGCCTATTCCCCATGACCACGACTTCTGTGAGAGAGGGAGACGCGCATGTTTT
shaker_Frag3-168_insertion57	TCCTCGATTCGTCTCCCATGGCCGCCGTGGCACTGCGAGAACAACAGCTCCAACGAAATAGTCTGGATGGATACAGCGGGAGACCGGGGTCTCTGAGCGGTTCACTGCCTAAACTGTCTAGCCAAGACGAAGAAGGTGGCGCCGGCCATGGCTTCGGTGGGGGCCCTCAGCATTTCGAGCCTATTCCCCATGACCACGACTTCTGTGAGAGAGGGAGACGCGCATGTTTT
shaker_Frag3-168_insertion60	TCCTCGATTCGTCTCCCATGGCCGCCGTGGCACTGCGAGAACAACAGCTCCAACGAAATAGTCTGGATGGATACGGTAGCGGGAGACCGGGGTCTCTGAGCTCACTGCCTAAACTGTCTAGCCAAGACGAAGAAGGTGGCGCCGGCCATGGCTTCGGTGGGGGCCCTCAGCATTTCGAGCCTATTCCCCATGACCACGACTTCTGTGAGAGAGGGAGACGCGCATGTTTT
shaker_Frag3-168_insertion63	TCCTCGATTCGTCTCCCATGGCCGCCGTGGCACTGCGAGAACAACAGCTCCAACGAAATAGTCTGGATGGATACGGTTCAAGCGGGAGACCGGGGTCTCTGAGCCTGCCTAAACTGTCTAGCCAAGACGAAGAAGGTGGCGCCGGCCATGGCTTCGGTGGGGGCCCTCAGCATTTCGAGCCTATTCCCCATGACCACGACTTCTGTGAGAGAGGGAGACGCGCATGTTTT
shaker_Frag3-168_insertion66	TCCTCGATTCGTCTCCCATGGCCGCCGTGGCACTGCGAGAACAACAGCTCCAACGAAATAGTCTGGATGGATACGGTTCACTGAGCGGGAGACCGGGGTCTCTGAGCCCTAAACTGTCTAGCCAAGACGAAGAAGGTGGCGCCGGCCATGGCTTCGGTGGGGGCCCTCAGCATTTCGAGCCTATTCCCCATGACCACGACTTCTGTGAGAGAGGGAGACGCGCATGTTTT
shaker_Frag3-168_insertion69	TCCTCGATTCGTCTCCCATGGCCGCCGTGGCACTGCGAGAACAACAGCTCCAACGAAATAGTCTGGATGGATACGGTTCACTGCCTAGCGGGAGACCGGGGTCTCTGAGCAAACTGTCTAGCCAAGACGAAGAAGGTGGCGCCGGCCATGGCTTCGGTGGGGGCCCTCAGCATTTCGAGCCTATTCCCCATGACCACGACTTCTGTGAGAGAGGGAGACGCGCATGTTTT
shaker_Frag3-168_insertion72	TCCTCGATTCGTCTCCCATGGCCGCCGTGGCACTGCGAGAACAACAGCTCCAACGAAATAGTCTGGATGGATACGGTTCACTGCCTAAAAGCGGGAGACCGGGGTCTCTGAGCCTGTCTAGCCAAGACGAAGAAGGTGGCGCCGGCCATGGCTTCGGTGGGGGCCCTCAGCATTTCGAGCCTATTCCCCATGACCACGACTTCTGTGAGAGAGGGAGACGCGCATGTTTT
shaker_Frag3-168_insertion75	TCCTCGATTCGTCTCCCATGGCCGCCGTGGCACTGCGAGAACAACAGCTCCAACGAAATAGTCTGGATGGATACGGTTCACTGCCTAAACTGAGCGGGAGACCGGGGTCTCTGAGCTCTAGCCAAGACGAAGAAGGTGGCGCCGGCCATGGCTTCGGTGGGGGCCCTCAGCATTTCGAGCCTATTCCCCATGACCACGACTTCTGTGAGAGAGGGAGACGCGCATGTTTT
shaker_Frag3-168_insertion78	TCCTCGATTCGTCTCCCATGGCCGCCGTGGCACTGCGAGAACAACAGCTCCAACGAAATAGTCTGGATGGATACGGTTCACTGCCTAAACTGTCTAGCGGGAGACCGGGGTCTCTGAGCAGCCAAGACGAAGAAGGTGGCGCCGGCCATGGCTTCGGTGGGGGCCCTCAGCATTTCGAGCCTATTCCCCATGACCACGACTTCTGTGAGAGAGGGAGACGCGCATGTTTT
shaker_Frag3-168_insertion81	TCCTCGATTCGTCTCCCATGGCCGCCGTGGCACTGCGAGAACAACAGCTCCAACGAAATAGTCTGGATGGATACGGTTCACTGCCTAAACTGTCTAGCAGCGGGAGACCGGGGTCTCTGAGCCAAGACGAAGAAGGTGGCGCCGGCCATGGCTTCGGTGGGGGCCCTCAGCATTTCGAGCCTATTCCCCATGACCACGACTTCTGTGAGAGAGGGAGACGCGCATGTTTT
shaker_Frag3-168_insertion84	TCCTCGATTCGTCTCCCATGGCCGCCGTGGCACTGCGAGAACAACAGCTCCAACGAAATAGTCTGGATGGATACGGTTCACTGCCTAAACTGTCTAGCCAAAGCGGGAGACCGGGGTCTCTGAGCGACGAAGAAGGTGGCGCCGGCCATGGCTTCGGTGGGGGCCCTCAGCATTTCGAGCCTATTCCCCATGACCACGACTTCTGTGAGAGAGGGAGACGCGCATGTTTT
shaker_Frag3-168_insertion87	TCCTCGATTCGTCTCCCATGGCCGCCGTGGCACTGCGAGAACAACAGCTCCAACGAAATAGTCTGGATGGATACGGTTCACTGCCTAAACTGTCTAGCCAAGACAGCGGGAGACCGGGGTCTCTGAGCGAAGAAGGTGGCGCCGGCCATGGCTTCGGTGGGGGCCCTCAGCATTTCGAGCCTATTCCCCATGACCACGACTTCTGTGAGAGAGGGAGACGCGCATGTTTT
shaker_Frag3-168_insertion90	TCCTCGATTCGTCTCCCATGGCCGCCGTGGCACTGCGAGAACAACAGCTCCAACGAAATAGTCTGGATGGATACGGTTCACTGCCTAAACTGTCTAGCCAAGACGAAAGCGGGAGACCGGGGTCTCTGAGCGAAGGTGGCGCCGGCCATGGCTTCGGTGGGGGCCCTCAGCATTTCGAGCCTATTCCCCATGACCACGACTTCTGTGAGAGAGGGAGACGCGCATGTTTT
shaker_Frag3-168_insertion93	TCCTCGATTCGTCTCCCATGGCCGCCGTGGCACTGCGAGAACAACAGCTCCAACGAAATAGTCTGGATGGATACGGTTCACTGCCTAAACTGTCTAGCCAAGACGAAGAAAGCGGGAGACCGGGGTCTCTGAGCGGTGGCGCCGGCCATGGCTTCGGTGGGGGCCCTCAGCATTTCGAGCCTATTCCCCATGACCACGACTTCTGTGAGAGAGGGAGACGCGCATGTTTT
shaker_Frag3-168_insertion96	TCCTCGATTCGTCTCCCATGGCCGCCGTGGCACTGCGAGAACAACAGCTCCAACGAAATAGTCTGGATGGATACGGTTCACTGCCTAAACTGTCTAGCCAAGACGAAGAAGGTAGCGGGAGACCGGGGTCTCTGAGCGGCGCCGGCCATGGCTTCGGTGGGGGCCCTCAGCATTTCGAGCCTATTCCCCATGACCACGACTTCTGTGAGAGAGGGAGACGCGCATGTTTT
shaker_Frag3-168_insertion99	TCCTCGATTCGTCTCCCATGGCCGCCGTGGCACTGCGAGAACAACAGCTCCAACGAAATAGTCTGGATGGATACGGTTCACTGCCTAAACTGTCTAGCCAAGACGAAGAAGGTGGCAGCGGGAGACCGGGGTCTCTGAGCGCCGGCCATGGCTTCGGTGGGGGCCCTCAGCATTTCGAGCCTATTCCCCATGACCACGACTTCTGTGAGAGAGGGAGACGCGCATGTTTT
shaker_Frag3-168_insertion102	TCCTCGATTCGTCTCCCATGGCCGCCGTGGCACTGCGAGAACAACAGCTCCAACGAAATAGTCTGGATGGATACGGTTCACTGCCTAAACTGTCTAGCCAAGACGAAGAAGGTGGCGCCAGCGGGAGACCGGGGTCTCTGAGCGGCCATGGCTTCGGTGGGGGCCCTCAGCATTTCGAGCCTATTCCCCATGACCACGACTTCTGTGAGAGAGGGAGACGCGCATGTTTT
shaker_Frag3-168_insertion105	TCCTCGATTCGTCTCCCATGGCCGCCGTGGCACTGCGAGAACAACAGCTCCAACGAAATAGTCTGGATGGATACGGTTCACTGCCTAAACTGTCTAGCCAAGACGAAGAAGGTGGCGCCGGCAGCGGGAGACCGGGGTCTCTGAGCCATGGCTTCGGTGGGGGCCCTCAGCATTTCGAGCCTATTCCCCATGACCACGACTTCTGTGAGAGAGGGAGACGCGCATGTTTT
shaker_Frag3-168_insertion108	TCCTCGATTCGTCTCCCATGGCCGCCGTGGCACTGCGAGAACAACAGCTCCAACGAAATAGTCTGGATGGATACGGTTCACTGCCTAAACTGTCTAGCCAAGACGAAGAAGGTGGCGCCGGCCATAGCGGGAGACCGGGGTCTCTGAGCGGCTTCGGTGGGGGCCCTCAGCATTTCGAGCCTATTCCCCATGACCACGACTTCTGTGAGAGAGGGAGACGCGCATGTTTT
shaker_Frag3-168_insertion111	TCCTCGATTCGTCTCCCATGGCCGCCGTGGCACTGCGAGAACAACAGCTCCAACGAAATAGTCTGGATGGATACGGTTCACTGCCTAAACTGTCTAGCCAAGACGAAGAAGGTGGCGCCGGCCATGGCAGCGGGAGACCGGGGTCTCTGAGCTTCGGTGGGGGCCCTCAGCATTTCGAGCCTATTCCCCATGACCACGACTTCTGTGAGAGAGGGAGACGCGCATGTTTT
shaker_Frag3-168_insertion114	TCCTCGATTCGTCTCCCATGGCCGCCGTGGCACTGCGAGAACAACAGCTCCAACGAAATAGTCTGGATGGATACGGTTCACTGCCTAAACTGTCTAGCCAAGACGAAGAAGGTGGCGCCGGCCATGGCTTCAGCGGGAGACCGGGGTCTCTGAGCGGTGGGGGCCCTCAGCATTTCGAGCCTATTCCCCATGACCACGACTTCTGTGAGAGAGGGAGACGCGCATGTTTT
shaker_Frag3-168_insertion117	TCCTCGATTCGTCTCCCATGGCCGCCGTGGCACTGCGAGAACAACAGCTCCAACGAAATAGTCTGGATGGATACGGTTCACTGCCTAAACTGTCTAGCCAAGACGAAGAAGGTGGCGCCGGCCATGGCTTCGGTAGCGGGAGACCGGGGTCTCTGAGCGGGGGCCCTCAGCATTTCGAGCCTATTCCCCATGACCACGACTTCTGTGAGAGAGGGAGACGCGCATGTTTT
shaker_Frag3-168_insertion120	TCCTCGATTCGTCTCCCATGGCCGCCGTGGCACTGCGAGAACAACAGCTCCAACGAAATAGTCTGGATGGATACGGTTCACTGCCTAAACTGTCTAGCCAAGACGAAGAAGGTGGCGCCGGCCATGGCTTCGGTGGGAGCGGGAGACCGGGGTCTCTGAGCGGCCCTCAGCATTTCGAGCCTATTCCCCATGACCACGACTTCTGTGAGAGAGGGAGACGCGCATGTTTT
shaker_Frag3-168_insertion123	TCCTCGATTCGTCTCCCATGGCCGCCGTGGCACTGCGAGAACAACAGCTCCAACGAAATAGTCTGGATGGATACGGTTCACTGCCTAAACTGTCTAGCCAAGACGAAGAAGGTGGCGCCGGCCATGGCTTCGGTGGGGGCAGCGGGAGACCGGGGTCTCTGAGCCCTCAGCATTTCGAGCCTATTCCCCATGACCACGACTTCTGTGAGAGAGGGAGACGCGCATGTTTT
shaker_Frag3-168_insertion126	TCCTCGATTCGTCTCCCATGGCCGCCGTGGCACTGCGAGAACAACAGCTCCAACGAAATAGTCTGGATGGATACGGTTCACTGCCTAAACTGTCTAGCCAAGACGAAGAAGGTGGCGCCGGCCATGGCTTCGGTGGGGGCCCTAGCGGGAGACCGGGGTCTCTGAGCCAGCATTTCGAGCCTATTCCCCATGACCACGACTTCTGTGAGAGAGGGAGACGCGCATGTTTT
shaker_Frag3-168_insertion129	TCCTCGATTCGTCTCCCATGGCCGCCGTGGCACTGCGAGAACAACAGCTCCAACGAAATAGTCTGGATGGATACGGTTCACTGCCTAAACTGTCTAGCCAAGACGAAGAAGGTGGCGCCGGCCATGGCTTCGGTGGGGGCCCTCAGAGCGGGAGACCGGGGTCTCTGAGCCATTTCGAGCCTATTCCCCATGACCACGACTTCTGTGAGAGAGGGAGACGCGCATGTTTT
shaker_Frag3-168_insertion132	TCCTCGATTCGTCTCCCATGGCCGCCGTGGCACTGCGAGAACAACAGCTCCAACGAAATAGTCTGGATGGATACGGTTCACTGCCTAAACTGTCTAGCCAAGACGAAGAAGGTGGCGCCGGCCATGGCTTCGGTGGGGGCCCTCAGCATAGCGGGAGACCGGGGTCTCTGAGCTTCGAGCCTATTCCCCATGACCACGACTTCTGTGAGAGAGGGAGACGCGCATGTTTT
shaker_Frag3-168_insertion135	TCCTCGATTCGTCTCCCATGGCCGCCGTGGCACTGCGAGAACAACAGCTCCAACGAAATAGTCTGGATGGATACGGTTCACTGCCTAAACTGTCTAGCCAAGACGAAGAAGGTGGCGCCGGCCATGGCTTCGGTGGGGGCCCTCAGCATTTCAGCGGGAGACCGGGGTCTCTGAGCGAGCCTATTCCCCATGACCACGACTTCTGTGAGAGAGGGAGACGCGCATGTTTT
shaker_Frag3-168_insertion138	TCCTCGATTCGTCTCCCATGGCCGCCGTGGCACTGCGAGAACAACAGCTCCAACGAAATAGTCTGGATGGATACGGTTCACTGCCTAAACTGTCTAGCCAAGACGAAGAAGGTGGCGCCGGCCATGGCTTCGGTGGGGGCCCTCAGCATTTCGAGAGCGGGAGACCGGGGTCTCTGAGCCCTATTCCCCATGACCACGACTTCTGTGAGAGAGGGAGACGCGCATGTTTT
shaker_Frag3-168_insertion141	TCCTCGATTCGTCTCCCATGGCCGCCGTGGCACTGCGAGAACAACAGCTCCAACGAAATAGTCTGGATGGATACGGTTCACTGCCTAAACTGTCTAGCCAAGACGAAGAAGGTGGCGCCGGCCATGGCTTCGGTGGGGGCCCTCAGCATTTCGAGCCTAGCGGGAGACCGGGGTCTCTGAGCATTCCCCATGACCACGACTTCTGTGAGAGAGGGAGACGCGCATGTTTT
shaker_Frag3-168_insertion144	TCCTCGATTCGTCTCCCATGGCCGCCGTGGCACTGCGAGAACAACAGCTCCAACGAAATAGTCTGGATGGATACGGTTCACTGCCTAAACTGTCTAGCCAAGACGAAGAAGGTGGCGCCGGCCATGGCTTCGGTGGGGGCCCTCAGCATTTCGAGCCTATTAGCGGGAGACCGGGGTCTCTGAGCCCCCATGACCACGACTTCTGTGAGAGAGGGAGACGCGCATGTTTT
shaker_Frag3-168_insertion147	TCCTCGATTCGTCTCCCATGGCCGCCGTGGCACTGCGAGAACAACAGCTCCAACGAAATAGTCTGGATGGATACGGTTCACTGCCTAAACTGTCTAGCCAAGACGAAGAAGGTGGCGCCGGCCATGGCTTCGGTGGGGGCCCTCAGCATTTCGAGCCTATTCCCAGCGGGAGACCGGGGTCTCTGAGCCATGACCACGACTTCTGTGAGAGAGGGAGACGCGCATGTTTT
shaker_Frag3-168_insertion150	TCCTCGATTCGTCTCCCATGGCCGCCGTGGCACTGCGAGAACAACAGCTCCAACGAAATAGTCTGGATGGATACGGTTCACTGCCTAAACTGTCTAGCCAAGACGAAGAAGGTGGCGCCGGCCATGGCTTCGGTGGGGGCCCTCAGCATTTCGAGCCTATTCCCCATAGCGGGAGACCGGGGTCTCTGAGCGACCACGACTTCTGTGAGAGAGGGAGACGCGCATGTTTT
shaker_Frag3-168_insertion153	TCCTCGATTCGTCTCCCATGGCCGCCGTGGCACTGCGAGAACAACAGCTCCAACGAAATAGTCTGGATGGATACGGTTCACTGCCTAAACTGTCTAGCCAAGACGAAGAAGGTGGCGCCGGCCATGGCTTCGGTGGGGGCCCTCAGCATTTCGAGCCTATTCCCCATGACAGCGGGAGACCGGGGTCTCTGAGCCACGACTTCTGTGAGAGAGGGAGACGCGCATGTTTT
shaker_Frag3-168_insertion156	TCCTCGATTCGTCTCCCATGGCCGCCGTGGCACTGCGAGAACAACAGCTCCAACGAAATAGTCTGGATGGATACGGTTCACTGCCTAAACTGTCTAGCCAAGACGAAGAAGGTGGCGCCGGCCATGGCTTCGGTGGGGGCCCTCAGCATTTCGAGCCTATTCCCCATGACCACAGCGGGAGACCGGGGTCTCTGAGCGACTTCTGTGAGAGAGGGAGACGCGCATGTTTT
shaker_Frag3-168_insertion159	TCCTCGATTCGTCTCCCATGGCCGCCGTGGCACTGCGAGAACAACAGCTCCAACGAAATAGTCTGGATGGATACGGTTCACTGCCTAAACTGTCTAGCCAAGACGAAGAAGGTGGCGCCGGCCATGGCTTCGGTGGGGGCCCTCAGCATTTCGAGCCTATTCCCCATGACCACGACAGCGGGAGACCGGGGTCTCTGAGCTTCTGTGAGAGAGGGAGACGCGCATGTTTT
shaker_Frag3-168_insertion162	TCCTCGATTCGTCTCCCATGGCCGCCGTGGCACTGCGAGAACAACAGCTCCAACGAAATAGTCTGGATGGATACGGTTCACTGCCTAAACTGTCTAGCCAAGACGAAGAAGGTGGCGCCGGCCATGGCTTCGGTGGGGGCCCTCAGCATTTCGAGCCTATTCCCCATGACCACGACTTCAGCGGGAGACCGGGGTCTCTGAGCTGTGAGAGAGGGAGACGCGCATGTTTT
shaker_Frag3-168_insertion165	TCCTCGATTCGTCTCCCATGGCCGCCGTGGCACTGCGAGAACAACAGCTCCAACGAAATAGTCTGGATGGATACGGTTCACTGCCTAAACTGTCTAGCCAAGACGAAGAAGGTGGCGCCGGCCATGGCTTCGGTGGGGGCCCTCAGCATTTCGAGCCTATTCCCCATGACCACGACTTCTGTAGCGGGAGACCGGGGTCTCTGAGCGAGAGAGGGAGACGCGCATGTTTT
shaker_Frag3-168_insertion168	TCCTCGATTCGTCTCCCATGGCCGCCGTGGCACTGCGAGAACAACAGCTCCAACGAAATAGTCTGGATGGATACGGTTCACTGCCTAAACTGTCTAGCCAAGACGAAGAAGGTGGCGCCGGCCATGGCTTCGGTGGGGGCCCTCAGCATTTCGAGCCTATTCCCCATGACCACGACTTCTGTGAGAGCGGGAGACCGGGGTCTCTGAGCAGAGGGAGACGCGCATGTTTT
shaker_Frag171-333_insertion171	GCTTAACGCATCGTCTCCGAGAAGCGGGAGACCGGGGTCTCTGAGCGTCGTGATCAACGTGAGTGGCCTTCGGTTTGAAACACAACTTCGAACATTGAATCAATTTCCCGATACTCTCTTGGGAGATCCCGCCCGCCGACTGCGATATTTTGACCCATTGCGAAACGAGTATTTTTTTGACCGAAGTCGACCTAGTTTTGACGCAATTCTCTGGAGACGGTAGGCTGAAC
shaker_Frag171-333_insertion174	GCTTAACGCATCGTCTCCGAGAGTCAGCGGGAGACCGGGGTCTCTGAGCGTGATCAACGTGAGTGGCCTTCGGTTTGAAACACAACTTCGAACATTGAATCAATTTCCCGATACTCTCTTGGGAGATCCCGCCCGCCGACTGCGATATTTTGACCCATTGCGAAACGAGTATTTTTTTGACCGAAGTCGACCTAGTTTTGACGCAATTCTCTGGAGACGGTAGGCTGAAC
shaker_Frag171-333_insertion177	GCTTAACGCATCGTCTCCGAGAGTCGTGAGCGGGAGACCGGGGTCTCTGAGCATCAACGTGAGTGGCCTTCGGTTTGAAACACAACTTCGAACATTGAATCAATTTCCCGATACTCTCTTGGGAGATCCCGCCCGCCGACTGCGATATTTTGACCCATTGCGAAACGAGTATTTTTTTGACCGAAGTCGACCTAGTTTTGACGCAATTCTCTGGAGACGGTAGGCTGAAC
shaker_Frag171-333_insertion180	GCTTAACGCATCGTCTCCGAGAGTCGTGATCAGCGGGAGACCGGGGTCTCTGAGCAACGTGAGTGGCCTTCGGTTTGAAACACAACTTCGAACATTGAATCAATTTCCCGATACTCTCTTGGGAGATCCCGCCCGCCGACTGCGATATTTTGACCCATTGCGAAACGAGTATTTTTTTGACCGAAGTCGACCTAGTTTTGACGCAATTCTCTGGAGACGGTAGGCTGAAC
shaker_Frag171-333_insertion183	GCTTAACGCATCGTCTCCGAGAGTCGTGATCAACAGCGGGAGACCGGGGTCTCTGAGCGTGAGTGGCCTTCGGTTTGAAACACAACTTCGAACATTGAATCAATTTCCCGATACTCTCTTGGGAGATCCCGCCCGCCGACTGCGATATTTTGACCCATTGCGAAACGAGTATTTTTTTGACCGAAGTCGACCTAGTTTTGACGCAATTCTCTGGAGACGGTAGGCTGAAC
shaker_Frag171-333_insertion186	GCTTAACGCATCGTCTCCGAGAGTCGTGATCAACGTGAGCGGGAGACCGGGGTCTCTGAGCAGTGGCCTTCGGTTTGAAACACAACTTCGAACATTGAATCAATTTCCCGATACTCTCTTGGGAGATCCCGCCCGCCGACTGCGATATTTTGACCCATTGCGAAACGAGTATTTTTTTGACCGAAGTCGACCTAGTTTTGACGCAATTCTCTGGAGACGGTAGGCTGAAC
shaker_Frag171-333_insertion189	GCTTAACGCATCGTCTCCGAGAGTCGTGATCAACGTGAGTAGCGGGAGACCGGGGTCTCTGAGCGGCCTTCGGTTTGAAACACAACTTCGAACATTGAATCAATTTCCCGATACTCTCTTGGGAGATCCCGCCCGCCGACTGCGATATTTTGACCCATTGCGAAACGAGTATTTTTTTGACCGAAGTCGACCTAGTTTTGACGCAATTCTCTGGAGACGGTAGGCTGAAC
shaker_Frag171-333_insertion192	GCTTAACGCATCGTCTCCGAGAGTCGTGATCAACGTGAGTGGCAGCGGGAGACCGGGGTCTCTGAGCCTTCGGTTTGAAACACAACTTCGAACATTGAATCAATTTCCCGATACTCTCTTGGGAGATCCCGCCCGCCGACTGCGATATTTTGACCCATTGCGAAACGAGTATTTTTTTGACCGAAGTCGACCTAGTTTTGACGCAATTCTCTGGAGACGGTAGGCTGAAC
shaker_Frag171-333_insertion195	GCTTAACGCATCGTCTCCGAGAGTCGTGATCAACGTGAGTGGCCTTAGCGGGAGACCGGGGTCTCTGAGCCGGTTTGAAACACAACTTCGAACATTGAATCAATTTCCCGATACTCTCTTGGGAGATCCCGCCCGCCGACTGCGATATTTTGACCCATTGCGAAACGAGTATTTTTTTGACCGAAGTCGACCTAGTTTTGACGCAATTCTCTGGAGACGGTAGGCTGAAC
shaker_Frag171-333_insertion198	GCTTAACGCATCGTCTCCGAGAGTCGTGATCAACGTGAGTGGCCTTCGGAGCGGGAGACCGGGGTCTCTGAGCTTTGAAACACAACTTCGAACATTGAATCAATTTCCCGATACTCTCTTGGGAGATCCCGCCCGCCGACTGCGATATTTTGACCCATTGCGAAACGAGTATTTTTTTGACCGAAGTCGACCTAGTTTTGACGCAATTCTCTGGAGACGGTAGGCTGAAC
shaker_Frag171-333_insertion201	GCTTAACGCATCGTCTCCGAGAGTCGTGATCAACGTGAGTGGCCTTCGGTTTAGCGGGAGACCGGGGTCTCTGAGCGAAACACAACTTCGAACATTGAATCAATTTCCCGATACTCTCTTGGGAGATCCCGCCCGCCGACTGCGATATTTTGACCCATTGCGAAACGAGTATTTTTTTGACCGAAGTCGACCTAGTTTTGACGCAATTCTCTGGAGACGGTAGGCTGAAC
shaker_Frag171-333_insertion204	GCTTAACGCATCGTCTCCGAGAGTCGTGATCAACGTGAGTGGCCTTCGGTTTGAAAGCGGGAGACCGGGGTCTCTGAGCACACAACTTCGAACATTGAATCAATTTCCCGATACTCTCTTGGGAGATCCCGCCCGCCGACTGCGATATTTTGACCCATTGCGAAACGAGTATTTTTTTGACCGAAGTCGACCTAGTTTTGACGCAATTCTCTGGAGACGGTAGGCTGAAC
shaker_Frag171-333_insertion207	GCTTAACGCATCGTCTCCGAGAGTCGTGATCAACGTGAGTGGCCTTCGGTTTGAAACAAGCGGGAGACCGGGGTCTCTGAGCCAACTTCGAACATTGAATCAATTTCCCGATACTCTCTTGGGAGATCCCGCCCGCCGACTGCGATATTTTGACCCATTGCGAAACGAGTATTTTTTTGACCGAAGTCGACCTAGTTTTGACGCAATTCTCTGGAGACGGTAGGCTGAAC
shaker_Frag171-333_insertion210	GCTTAACGCATCGTCTCCGAGAGTCGTGATCAACGTGAGTGGCCTTCGGTTTGAAACACAAAGCGGGAGACCGGGGTCTCTGAGCCTTCGAACATTGAATCAATTTCCCGATACTCTCTTGGGAGATCCCGCCCGCCGACTGCGATATTTTGACCCATTGCGAAACGAGTATTTTTTTGACCGAAGTCGACCTAGTTTTGACGCAATTCTCTGGAGACGGTAGGCTGAAC
shaker_Frag171-333_insertion213	GCTTAACGCATCGTCTCCGAGAGTCGTGATCAACGTGAGTGGCCTTCGGTTTGAAACACAACTTAGCGGGAGACCGGGGTCTCTGAGCCGAACATTGAATCAATTTCCCGATACTCTCTTGGGAGATCCCGCCCGCCGACTGCGATATTTTGACCCATTGCGAAACGAGTATTTTTTTGACCGAAGTCGACCTAGTTTTGACGCAATTCTCTGGAGACGGTAGGCTGAAC
shaker_Frag171-333_insertion216	GCTTAACGCATCGTCTCCGAGAGTCGTGATCAACGTGAGTGGCCTTCGGTTTGAAACACAACTTCGAAGCGGGAGACCGGGGTCTCTGAGCACATTGAATCAATTTCCCGATACTCTCTTGGGAGATCCCGCCCGCCGACTGCGATATTTTGACCCATTGCGAAACGAGTATTTTTTTGACCGAAGTCGACCTAGTTTTGACGCAATTCTCTGGAGACGGTAGGCTGAAC
shaker_Frag171-333_insertion219	GCTTAACGCATCGTCTCCGAGAGTCGTGATCAACGTGAGTGGCCTTCGGTTTGAAACACAACTTCGAACAAGCGGGAGACCGGGGTCTCTGAGCTTGAATCAATTTCCCGATACTCTCTTGGGAGATCCCGCCCGCCGACTGCGATATTTTGACCCATTGCGAAACGAGTATTTTTTTGACCGAAGTCGACCTAGTTTTGACGCAATTCTCTGGAGACGGTAGGCTGAAC
shaker_Frag171-333_insertion222	GCTTAACGCATCGTCTCCGAGAGTCGTGATCAACGTGAGTGGCCTTCGGTTTGAAACACAACTTCGAACATTGAGCGGGAGACCGGGGTCTCTGAGCAATCAATTTCCCGATACTCTCTTGGGAGATCCCGCCCGCCGACTGCGATATTTTGACCCATTGCGAAACGAGTATTTTTTTGACCGAAGTCGACCTAGTTTTGACGCAATTCTCTGGAGACGGTAGGCTGAAC
shaker_Frag171-333_insertion225	GCTTAACGCATCGTCTCCGAGAGTCGTGATCAACGTGAGTGGCCTTCGGTTTGAAACACAACTTCGAACATTGAATAGCGGGAGACCGGGGTCTCTGAGCCAATTTCCCGATACTCTCTTGGGAGATCCCGCCCGCCGACTGCGATATTTTGACCCATTGCGAAACGAGTATTTTTTTGACCGAAGTCGACCTAGTTTTGACGCAATTCTCTGGAGACGGTAGGCTGAAC
shaker_Frag171-333_insertion228	GCTTAACGCATCGTCTCCGAGAGTCGTGATCAACGTGAGTGGCCTTCGGTTTGAAACACAACTTCGAACATTGAATCAAAGCGGGAGACCGGGGTCTCTGAGCTTTCCCGATACTCTCTTGGGAGATCCCGCCCGCCGACTGCGATATTTTGACCCATTGCGAAACGAGTATTTTTTTGACCGAAGTCGACCTAGTTTTGACGCAATTCTCTGGAGACGGTAGGCTGAAC
shaker_Frag171-333_insertion231	GCTTAACGCATCGTCTCCGAGAGTCGTGATCAACGTGAGTGGCCTTCGGTTTGAAACACAACTTCGAACATTGAATCAATTTAGCGGGAGACCGGGGTCTCTGAGCCCCGATACTCTCTTGGGAGATCCCGCCCGCCGACTGCGATATTTTGACCCATTGCGAAACGAGTATTTTTTTGACCGAAGTCGACCTAGTTTTGACGCAATTCTCTGGAGACGGTAGGCTGAAC
shaker_Frag171-333_insertion234	GCTTAACGCATCGTCTCCGAGAGTCGTGATCAACGTGAGTGGCCTTCGGTTTGAAACACAACTTCGAACATTGAATCAATTTCCCAGCGGGAGACCGGGGTCTCTGAGCGATACTCTCTTGGGAGATCCCGCCCGCCGACTGCGATATTTTGACCCATTGCGAAACGAGTATTTTTTTGACCGAAGTCGACCTAGTTTTGACGCAATTCTCTGGAGACGGTAGGCTGAAC
shaker_Frag171-333_insertion237	GCTTAACGCATCGTCTCCGAGAGTCGTGATCAACGTGAGTGGCCTTCGGTTTGAAACACAACTTCGAACATTGAATCAATTTCCCGATAGCGGGAGACCGGGGTCTCTGAGCACTCTCTTGGGAGATCCCGCCCGCCGACTGCGATATTTTGACCCATTGCGAAACGAGTATTTTTTTGACCGAAGTCGACCTAGTTTTGACGCAATTCTCTGGAGACGGTAGGCTGAAC
shaker_Frag171-333_insertion240	GCTTAACGCATCGTCTCCGAGAGTCGTGATCAACGTGAGTGGCCTTCGGTTTGAAACACAACTTCGAACATTGAATCAATTTCCCGATACTAGCGGGAGACCGGGGTCTCTGAGCCTCTTGGGAGATCCCGCCCGCCGACTGCGATATTTTGACCCATTGCGAAACGAGTATTTTTTTGACCGAAGTCGACCTAGTTTTGACGCAATTCTCTGGAGACGGTAGGCTGAAC
shaker_Frag171-333_insertion243	GCTTAACGCATCGTCTCCGAGAGTCGTGATCAACGTGAGTGGCCTTCGGTTTGAAACACAACTTCGAACATTGAATCAATTTCCCGATACTCTCAGCGGGAGACCGGGGTCTCTGAGCTTGGGAGATCCCGCCCGCCGACTGCGATATTTTGACCCATTGCGAAACGAGTATTTTTTTGACCGAAGTCGACCTAGTTTTGACGCAATTCTCTGGAGACGGTAGGCTGAAC
shaker_Frag171-333_insertion246	GCTTAACGCATCGTCTCCGAGAGTCGTGATCAACGTGAGTGGCCTTCGGTTTGAAACACAACTTCGAACATTGAATCAATTTCCCGATACTCTCTTGAGCGGGAGACCGGGGTCTCTGAGCGGAGATCCCGCCCGCCGACTGCGATATTTTGACCCATTGCGAAACGAGTATTTTTTTGACCGAAGTCGACCTAGTTTTGACGCAATTCTCTGGAGACGGTAGGCTGAAC
shaker_Frag171-333_insertion249	GCTTAACGCATCGTCTCCGAGAGTCGTGATCAACGTGAGTGGCCTTCGGTTTGAAACACAACTTCGAACATTGAATCAATTTCCCGATACTCTCTTGGGAAGCGGGAGACCGGGGTCTCTGAGCGATCCCGCCCGCCGACTGCGATATTTTGACCCATTGCGAAACGAGTATTTTTTTGACCGAAGTCGACCTAGTTTTGACGCAATTCTCTGGAGACGGTAGGCTGAAC
shaker_Frag171-333_insertion252	GCTTAACGCATCGTCTCCGAGAGTCGTGATCAACGTGAGTGGCCTTCGGTTTGAAACACAACTTCGAACATTGAATCAATTTCCCGATACTCTCTTGGGAGATAGCGGGAGACCGGGGTCTCTGAGCCCCGCCCGCCGACTGCGATATTTTGACCCATTGCGAAACGAGTATTTTTTTGACCGAAGTCGACCTAGTTTTGACGCAATTCTCTGGAGACGGTAGGCTGAAC
shaker_Frag171-333_insertion255	GCTTAACGCATCGTCTCCGAGAGTCGTGATCAACGTGAGTGGCCTTCGGTTTGAAACACAACTTCGAACATTGAATCAATTTCCCGATACTCTCTTGGGAGATCCCAGCGGGAGACCGGGGTCTCTGAGCGCCCGCCGACTGCGATATTTTGACCCATTGCGAAACGAGTATTTTTTTGACCGAAGTCGACCTAGTTTTGACGCAATTCTCTGGAGACGGTAGGCTGAAC
shaker_Frag171-333_insertion258	GCTTAACGCATCGTCTCCGAGAGTCGTGATCAACGTGAGTGGCCTTCGGTTTGAAACACAACTTCGAACATTGAATCAATTTCCCGATACTCTCTTGGGAGATCCCGCCAGCGGGAGACCGGGGTCTCTGAGCCGCCGACTGCGATATTTTGACCCATTGCGAAACGAGTATTTTTTTGACCGAAGTCGACCTAGTTTTGACGCAATTCTCTGGAGACGGTAGGCTGAAC
shaker_Frag171-333_insertion261	GCTTAACGCATCGTCTCCGAGAGTCGTGATCAACGTGAGTGGCCTTCGGTTTGAAACACAACTTCGAACATTGAATCAATTTCCCGATACTCTCTTGGGAGATCCCGCCCGCAGCGGGAGACCGGGGTCTCTGAGCCGACTGCGATATTTTGACCCATTGCGAAACGAGTATTTTTTTGACCGAAGTCGACCTAGTTTTGACGCAATTCTCTGGAGACGGTAGGCTGAAC
shaker_Frag171-333_insertion264	GCTTAACGCATCGTCTCCGAGAGTCGTGATCAACGTGAGTGGCCTTCGGTTTGAAACACAACTTCGAACATTGAATCAATTTCCCGATACTCTCTTGGGAGATCCCGCCCGCCGAAGCGGGAGACCGGGGTCTCTGAGCCTGCGATATTTTGACCCATTGCGAAACGAGTATTTTTTTGACCGAAGTCGACCTAGTTTTGACGCAATTCTCTGGAGACGGTAGGCTGAAC
shaker_Frag171-333_insertion267	GCTTAACGCATCGTCTCCGAGAGTCGTGATCAACGTGAGTGGCCTTCGGTTTGAAACACAACTTCGAACATTGAATCAATTTCCCGATACTCTCTTGGGAGATCCCGCCCGCCGACTGAGCGGGAGACCGGGGTCTCTGAGCCGATATTTTGACCCATTGCGAAACGAGTATTTTTTTGACCGAAGTCGACCTAGTTTTGACGCAATTCTCTGGAGACGGTAGGCTGAAC
shaker_Frag171-333_insertion270	GCTTAACGCATCGTCTCCGAGAGTCGTGATCAACGTGAGTGGCCTTCGGTTTGAAACACAACTTCGAACATTGAATCAATTTCCCGATACTCTCTTGGGAGATCCCGCCCGCCGACTGCGAAGCGGGAGACCGGGGTCTCTGAGCTATTTTGACCCATTGCGAAACGAGTATTTTTTTGACCGAAGTCGACCTAGTTTTGACGCAATTCTCTGGAGACGGTAGGCTGAAC
shaker_Frag171-333_insertion273	GCTTAACGCATCGTCTCCGAGAGTCGTGATCAACGTGAGTGGCCTTCGGTTTGAAACACAACTTCGAACATTGAATCAATTTCCCGATACTCTCTTGGGAGATCCCGCCCGCCGACTGCGATATAGCGGGAGACCGGGGTCTCTGAGCTTTGACCCATTGCGAAACGAGTATTTTTTTGACCGAAGTCGACCTAGTTTTGACGCAATTCTCTGGAGACGGTAGGCTGAAC
shaker_Frag171-333_insertion276	GCTTAACGCATCGTCTCCGAGAGTCGTGATCAACGTGAGTGGCCTTCGGTTTGAAACACAACTTCGAACATTGAATCAATTTCCCGATACTCTCTTGGGAGATCCCGCCCGCCGACTGCGATATTTTAGCGGGAGACCGGGGTCTCTGAGCGACCCATTGCGAAACGAGTATTTTTTTGACCGAAGTCGACCTAGTTTTGACGCAATTCTCTGGAGACGGTAGGCTGAAC
shaker_Frag171-333_insertion279	GCTTAACGCATCGTCTCCGAGAGTCGTGATCAACGTGAGTGGCCTTCGGTTTGAAACACAACTTCGAACATTGAATCAATTTCCCGATACTCTCTTGGGAGATCCCGCCCGCCGACTGCGATATTTTGACAGCGGGAGACCGGGGTCTCTGAGCCCATTGCGAAACGAGTATTTTTTTGACCGAAGTCGACCTAGTTTTGACGCAATTCTCTGGAGACGGTAGGCTGAAC
shaker_Frag171-333_insertion282	GCTTAACGCATCGTCTCCGAGAGTCGTGATCAACGTGAGTGGCCTTCGGTTTGAAACACAACTTCGAACATTGAATCAATTTCCCGATACTCTCTTGGGAGATCCCGCCCGCCGACTGCGATATTTTGACCCAAGCGGGAGACCGGGGTCTCTGAGCTTGCGAAACGAGTATTTTTTTGACCGAAGTCGACCTAGTTTTGACGCAATTCTCTGGAGACGGTAGGCTGAAC
shaker_Frag171-333_insertion285	GCTTAACGCATCGTCTCCGAGAGTCGTGATCAACGTGAGTGGCCTTCGGTTTGAAACACAACTTCGAACATTGAATCAATTTCCCGATACTCTCTTGGGAGATCCCGCCCGCCGACTGCGATATTTTGACCCATTGAGCGGGAGACCGGGGTCTCTGAGCCGAAACGAGTATTTTTTTGACCGAAGTCGACCTAGTTTTGACGCAATTCTCTGGAGACGGTAGGCTGAAC
shaker_Frag171-333_insertion288	GCTTAACGCATCGTCTCCGAGAGTCGTGATCAACGTGAGTGGCCTTCGGTTTGAAACACAACTTCGAACATTGAATCAATTTCCCGATACTCTCTTGGGAGATCCCGCCCGCCGACTGCGATATTTTGACCCATTGCGAAGCGGGAGACCGGGGTCTCTGAGCAACGAGTATTTTTTTGACCGAAGTCGACCTAGTTTTGACGCAATTCTCTGGAGACGGTAGGCTGAAC
shaker_Frag171-333_insertion291	GCTTAACGCATCGTCTCCGAGAGTCGTGATCAACGTGAGTGGCCTTCGGTTTGAAACACAACTTCGAACATTGAATCAATTTCCCGATACTCTCTTGGGAGATCCCGCCCGCCGACTGCGATATTTTGACCCATTGCGAAACAGCGGGAGACCGGGGTCTCTGAGCGAGTATTTTTTTGACCGAAGTCGACCTAGTTTTGACGCAATTCTCTGGAGACGGTAGGCTGAAC
shaker_Frag171-333_insertion294	GCTTAACGCATCGTCTCCGAGAGTCGTGATCAACGTGAGTGGCCTTCGGTTTGAAACACAACTTCGAACATTGAATCAATTTCCCGATACTCTCTTGGGAGATCCCGCCCGCCGACTGCGATATTTTGACCCATTGCGAAACGAGAGCGGGAGACCGGGGTCTCTGAGCTATTTTTTTGACCGAAGTCGACCTAGTTTTGACGCAATTCTCTGGAGACGGTAGGCTGAAC
shaker_Frag171-333_insertion297	GCTTAACGCATCGTCTCCGAGAGTCGTGATCAACGTGAGTGGCCTTCGGTTTGAAACACAACTTCGAACATTGAATCAATTTCCCGATACTCTCTTGGGAGATCCCGCCCGCCGACTGCGATATTTTGACCCATTGCGAAACGAGTATAGCGGGAGACCGGGGTCTCTGAGCTTTTTTGACCGAAGTCGACCTAGTTTTGACGCAATTCTCTGGAGACGGTAGGCTGAAC
shaker_Frag171-333_insertion300	GCTTAACGCATCGTCTCCGAGAGTCGTGATCAACGTGAGTGGCCTTCGGTTTGAAACACAACTTCGAACATTGAATCAATTTCCCGATACTCTCTTGGGAGATCCCGCCCGCCGACTGCGATATTTTGACCCATTGCGAAACGAGTATTTTAGCGGGAGACCGGGGTCTCTGAGCTTTGACCGAAGTCGACCTAGTTTTGACGCAATTCTCTGGAGACGGTAGGCTGAAC
shaker_Frag171-333_insertion303	GCTTAACGCATCGTCTCCGAGAGTCGTGATCAACGTGAGTGGCCTTCGGTTTGAAACACAACTTCGAACATTGAATCAATTTCCCGATACTCTCTTGGGAGATCCCGCCCGCCGACTGCGATATTTTGACCCATTGCGAAACGAGTATTTTTTTAGCGGGAGACCGGGGTCTCTGAGCGACCGAAGTCGACCTAGTTTTGACGCAATTCTCTGGAGACGGTAGGCTGAAC
shaker_Frag171-333_insertion306	GCTTAACGCATCGTCTCCGAGAGTCGTGATCAACGTGAGTGGCCTTCGGTTTGAAACACAACTTCGAACATTGAATCAATTTCCCGATACTCTCTTGGGAGATCCCGCCCGCCGACTGCGATATTTTGACCCATTGCGAAACGAGTATTTTTTTGACAGCGGGAGACCGGGGTCTCTGAGCCGAAGTCGACCTAGTTTTGACGCAATTCTCTGGAGACGGTAGGCTGAAC
shaker_Frag171-333_insertion309	GCTTAACGCATCGTCTCCGAGAGTCGTGATCAACGTGAGTGGCCTTCGGTTTGAAACACAACTTCGAACATTGAATCAATTTCCCGATACTCTCTTGGGAGATCCCGCCCGCCGACTGCGATATTTTGACCCATTGCGAAACGAGTATTTTTTTGACCGAAGCGGGAGACCGGGGTCTCTGAGCAGTCGACCTAGTTTTGACGCAATTCTCTGGAGACGGTAGGCTGAAC
shaker_Frag171-333_insertion312	GCTTAACGCATCGTCTCCGAGAGTCGTGATCAACGTGAGTGGCCTTCGGTTTGAAACACAACTTCGAACATTGAATCAATTTCCCGATACTCTCTTGGGAGATCCCGCCCGCCGACTGCGATATTTTGACCCATTGCGAAACGAGTATTTTTTTGACCGAAGTAGCGGGAGACCGGGGTCTCTGAGCCGACCTAGTTTTGACGCAATTCTCTGGAGACGGTAGGCTGAAC
shaker_Frag171-333_insertion315	GCTTAACGCATCGTCTCCGAGAGTCGTGATCAACGTGAGTGGCCTTCGGTTTGAAACACAACTTCGAACATTGAATCAATTTCCCGATACTCTCTTGGGAGATCCCGCCCGCCGACTGCGATATTTTGACCCATTGCGAAACGAGTATTTTTTTGACCGAAGTCGAAGCGGGAGACCGGGGTCTCTGAGCCCTAGTTTTGACGCAATTCTCTGGAGACGGTAGGCTGAAC
shaker_Frag171-333_insertion318	GCTTAACGCATCGTCTCCGAGAGTCGTGATCAACGTGAGTGGCCTTCGGTTTGAAACACAACTTCGAACATTGAATCAATTTCCCGATACTCTCTTGGGAGATCCCGCCCGCCGACTGCGATATTTTGACCCATTGCGAAACGAGTATTTTTTTGACCGAAGTCGACCTAGCGGGAGACCGGGGTCTCTGAGCAGTTTTGACGCAATTCTCTGGAGACGGTAGGCTGAAC
shaker_Frag171-333_insertion321	GCTTAACGCATCGTCTCCGAGAGTCGTGATCAACGTGAGTGGCCTTCGGTTTGAAACACAACTTCGAACATTGAATCAATTTCCCGATACTCTCTTGGGAGATCCCGCCCGCCGACTGCGATATTTTGACCCATTGCGAAACGAGTATTTTTTTGACCGAAGTCGACCTAGTAGCGGGAGACCGGGGTCTCTGAGCTTTGACGCAATTCTCTGGAGACGGTAGGCTGAAC
shaker_Frag171-333_insertion324	GCTTAACGCATCGTCTCCGAGAGTCGTGATCAACGTGAGTGGCCTTCGGTTTGAAACACAACTTCGAACATTGAATCAATTTCCCGATACTCTCTTGGGAGATCCCGCCCGCCGACTGCGATATTTTGACCCATTGCGAAACGAGTATTTTTTTGACCGAAGTCGACCTAGTTTTAGCGGGAGACCGGGGTCTCTGAGCGACGCAATTCTCTGGAGACGGTAGGCTGAAC
shaker_Frag171-333_insertion327	GCTTAACGCATCGTCTCCGAGAGTCGTGATCAACGTGAGTGGCCTTCGGTTTGAAACACAACTTCGAACATTGAATCAATTTCCCGATACTCTCTTGGGAGATCCCGCCCGCCGACTGCGATATTTTGACCCATTGCGAAACGAGTATTTTTTTGACCGAAGTCGACCTAGTTTTGACAGCGGGAGACCGGGGTCTCTGAGCGCAATTCTCTGGAGACGGTAGGCTGAAC
shaker_Frag171-333_insertion330	GCTTAACGCATCGTCTCCGAGAGTCGTGATCAACGTGAGTGGCCTTCGGTTTGAAACACAACTTCGAACATTGAATCAATTTCCCGATACTCTCTTGGGAGATCCCGCCCGCCGACTGCGATATTTTGACCCATTGCGAAACGAGTATTTTTTTGACCGAAGTCGACCTAGTTTTGACGCAAGCGGGAGACCGGGGTCTCTGAGCATTCTCTGGAGACGGTAGGCTGAAC
shaker_Frag171-333_insertion333	GCTTAACGCATCGTCTCCGAGAGTCGTGATCAACGTGAGTGGCCTTCGGTTTGAAACACAACTTCGAACATTGAATCAATTTCCCGATACTCTCTTGGGAGATCCCGCCCGCCGACTGCGATATTTTGACCCATTGCGAAACGAGTATTTTTTTGACCGAAGTCGACCTAGTTTTGACGCAATTAGCGGGAGACCGGGGTCTCTGAGCCTCTGGAGACGGTAGGCTGAAC
shaker_Frag336-501_insertion336	GTGGGCGTTCGTCTCCTCTCAGCGGGAGACCGGGGTCTCTGAGCTATTACTATCAATCAGGAGGGCGCTTGCGCCGGCCCGTAAACGTACCACTTGATGTCTTTAGTGAAGAGATCAAATTTTACGAGCTCGGCGACCAGGCTATTAACAAATTCAGAGAAGATGAAGGCTTCATAAAAGAGGAAGAAAGGCCATTGCCCGACAATGAAAAACGGAGACGCCGAACTGGA
shaker_Frag336-501_insertion339	GTGGGCGTTCGTCTCCTCTCTATAGCGGGAGACCGGGGTCTCTGAGCTACTATCAATCAGGAGGGCGCTTGCGCCGGCCCGTAAACGTACCACTTGATGTCTTTAGTGAAGAGATCAAATTTTACGAGCTCGGCGACCAGGCTATTAACAAATTCAGAGAAGATGAAGGCTTCATAAAAGAGGAAGAAAGGCCATTGCCCGACAATGAAAAACGGAGACGCCGAACTGGA
shaker_Frag336-501_insertion342	GTGGGCGTTCGTCTCCTCTCTATTACAGCGGGAGACCGGGGTCTCTGAGCTATCAATCAGGAGGGCGCTTGCGCCGGCCCGTAAACGTACCACTTGATGTCTTTAGTGAAGAGATCAAATTTTACGAGCTCGGCGACCAGGCTATTAACAAATTCAGAGAAGATGAAGGCTTCATAAAAGAGGAAGAAAGGCCATTGCCCGACAATGAAAAACGGAGACGCCGAACTGGA
shaker_Frag336-501_insertion345	GTGGGCGTTCGTCTCCTCTCTATTACTATAGCGGGAGACCGGGGTCTCTGAGCCAATCAGGAGGGCGCTTGCGCCGGCCCGTAAACGTACCACTTGATGTCTTTAGTGAAGAGATCAAATTTTACGAGCTCGGCGACCAGGCTATTAACAAATTCAGAGAAGATGAAGGCTTCATAAAAGAGGAAGAAAGGCCATTGCCCGACAATGAAAAACGGAGACGCCGAACTGGA
shaker_Frag336-501_insertion348	GTGGGCGTTCGTCTCCTCTCTATTACTATCAAAGCGGGAGACCGGGGTCTCTGAGCTCAGGAGGGCGCTTGCGCCGGCCCGTAAACGTACCACTTGATGTCTTTAGTGAAGAGATCAAATTTTACGAGCTCGGCGACCAGGCTATTAACAAATTCAGAGAAGATGAAGGCTTCATAAAAGAGGAAGAAAGGCCATTGCCCGACAATGAAAAACGGAGACGCCGAACTGGA
shaker_Frag336-501_insertion351	GTGGGCGTTCGTCTCCTCTCTATTACTATCAATCAAGCGGGAGACCGGGGTCTCTGAGCGGAGGGCGCTTGCGCCGGCCCGTAAACGTACCACTTGATGTCTTTAGTGAAGAGATCAAATTTTACGAGCTCGGCGACCAGGCTATTAACAAATTCAGAGAAGATGAAGGCTTCATAAAAGAGGAAGAAAGGCCATTGCCCGACAATGAAAAACGGAGACGCCGAACTGGA
shaker_Frag336-501_insertion354	GTGGGCGTTCGTCTCCTCTCTATTACTATCAATCAGGAAGCGGGAGACCGGGGTCTCTGAGCGGGCGCTTGCGCCGGCCCGTAAACGTACCACTTGATGTCTTTAGTGAAGAGATCAAATTTTACGAGCTCGGCGACCAGGCTATTAACAAATTCAGAGAAGATGAAGGCTTCATAAAAGAGGAAGAAAGGCCATTGCCCGACAATGAAAAACGGAGACGCCGAACTGGA
shaker_Frag336-501_insertion357	GTGGGCGTTCGTCTCCTCTCTATTACTATCAATCAGGAGGGAGCGGGAGACCGGGGTCTCTGAGCCGCTTGCGCCGGCCCGTAAACGTACCACTTGATGTCTTTAGTGAAGAGATCAAATTTTACGAGCTCGGCGACCAGGCTATTAACAAATTCAGAGAAGATGAAGGCTTCATAAAAGAGGAAGAAAGGCCATTGCCCGACAATGAAAAACGGAGACGCCGAACTGGA
shaker_Frag336-501_insertion360	GTGGGCGTTCGTCTCCTCTCTATTACTATCAATCAGGAGGGCGCAGCGGGAGACCGGGGTCTCTGAGCTTGCGCCGGCCCGTAAACGTACCACTTGATGTCTTTAGTGAAGAGATCAAATTTTACGAGCTCGGCGACCAGGCTATTAACAAATTCAGAGAAGATGAAGGCTTCATAAAAGAGGAAGAAAGGCCATTGCCCGACAATGAAAAACGGAGACGCCGAACTGGA
shaker_Frag336-501_insertion363	GTGGGCGTTCGTCTCCTCTCTATTACTATCAATCAGGAGGGCGCTTGAGCGGGAGACCGGGGTCTCTGAGCCGCCGGCCCGTAAACGTACCACTTGATGTCTTTAGTGAAGAGATCAAATTTTACGAGCTCGGCGACCAGGCTATTAACAAATTCAGAGAAGATGAAGGCTTCATAAAAGAGGAAGAAAGGCCATTGCCCGACAATGAAAAACGGAGACGCCGAACTGGA
shaker_Frag336-501_insertion366	GTGGGCGTTCGTCTCCTCTCTATTACTATCAATCAGGAGGGCGCTTGCGCAGCGGGAGACCGGGGTCTCTGAGCCGGCCCGTAAACGTACCACTTGATGTCTTTAGTGAAGAGATCAAATTTTACGAGCTCGGCGACCAGGCTATTAACAAATTCAGAGAAGATGAAGGCTTCATAAAAGAGGAAGAAAGGCCATTGCCCGACAATGAAAAACGGAGACGCCGAACTGGA
shaker_Frag336-501_insertion369	GTGGGCGTTCGTCTCCTCTCTATTACTATCAATCAGGAGGGCGCTTGCGCCGGAGCGGGAGACCGGGGTCTCTGAGCCCCGTAAACGTACCACTTGATGTCTTTAGTGAAGAGATCAAATTTTACGAGCTCGGCGACCAGGCTATTAACAAATTCAGAGAAGATGAAGGCTTCATAAAAGAGGAAGAAAGGCCATTGCCCGACAATGAAAAACGGAGACGCCGAACTGGA
shaker_Frag336-501_insertion372	GTGGGCGTTCGTCTCCTCTCTATTACTATCAATCAGGAGGGCGCTTGCGCCGGCCCAGCGGGAGACCGGGGTCTCTGAGCGTAAACGTACCACTTGATGTCTTTAGTGAAGAGATCAAATTTTACGAGCTCGGCGACCAGGCTATTAACAAATTCAGAGAAGATGAAGGCTTCATAAAAGAGGAAGAAAGGCCATTGCCCGACAATGAAAAACGGAGACGCCGAACTGGA
shaker_Frag336-501_insertion375	GTGGGCGTTCGTCTCCTCTCTATTACTATCAATCAGGAGGGCGCTTGCGCCGGCCCGTAAGCGGGAGACCGGGGTCTCTGAGCAACGTACCACTTGATGTCTTTAGTGAAGAGATCAAATTTTACGAGCTCGGCGACCAGGCTATTAACAAATTCAGAGAAGATGAAGGCTTCATAAAAGAGGAAGAAAGGCCATTGCCCGACAATGAAAAACGGAGACGCCGAACTGGA
shaker_Frag336-501_insertion378	GTGGGCGTTCGTCTCCTCTCTATTACTATCAATCAGGAGGGCGCTTGCGCCGGCCCGTAAACAGCGGGAGACCGGGGTCTCTGAGCGTACCACTTGATGTCTTTAGTGAAGAGATCAAATTTTACGAGCTCGGCGACCAGGCTATTAACAAATTCAGAGAAGATGAAGGCTTCATAAAAGAGGAAGAAAGGCCATTGCCCGACAATGAAAAACGGAGACGCCGAACTGGA
shaker_Frag336-501_insertion381	GTGGGCGTTCGTCTCCTCTCTATTACTATCAATCAGGAGGGCGCTTGCGCCGGCCCGTAAACGTAAGCGGGAGACCGGGGTCTCTGAGCCCACTTGATGTCTTTAGTGAAGAGATCAAATTTTACGAGCTCGGCGACCAGGCTATTAACAAATTCAGAGAAGATGAAGGCTTCATAAAAGAGGAAGAAAGGCCATTGCCCGACAATGAAAAACGGAGACGCCGAACTGGA
shaker_Frag336-501_insertion384	GTGGGCGTTCGTCTCCTCTCTATTACTATCAATCAGGAGGGCGCTTGCGCCGGCCCGTAAACGTACCAAGCGGGAGACCGGGGTCTCTGAGCCTTGATGTCTTTAGTGAAGAGATCAAATTTTACGAGCTCGGCGACCAGGCTATTAACAAATTCAGAGAAGATGAAGGCTTCATAAAAGAGGAAGAAAGGCCATTGCCCGACAATGAAAAACGGAGACGCCGAACTGGA
shaker_Frag336-501_insertion387	GTGGGCGTTCGTCTCCTCTCTATTACTATCAATCAGGAGGGCGCTTGCGCCGGCCCGTAAACGTACCACTTAGCGGGAGACCGGGGTCTCTGAGCGATGTCTTTAGTGAAGAGATCAAATTTTACGAGCTCGGCGACCAGGCTATTAACAAATTCAGAGAAGATGAAGGCTTCATAAAAGAGGAAGAAAGGCCATTGCCCGACAATGAAAAACGGAGACGCCGAACTGGA
shaker_Frag336-501_insertion390	GTGGGCGTTCGTCTCCTCTCTATTACTATCAATCAGGAGGGCGCTTGCGCCGGCCCGTAAACGTACCACTTGATAGCGGGAGACCGGGGTCTCTGAGCGTCTTTAGTGAAGAGATCAAATTTTACGAGCTCGGCGACCAGGCTATTAACAAATTCAGAGAAGATGAAGGCTTCATAAAAGAGGAAGAAAGGCCATTGCCCGACAATGAAAAACGGAGACGCCGAACTGGA
shaker_Frag336-501_insertion393	GTGGGCGTTCGTCTCCTCTCTATTACTATCAATCAGGAGGGCGCTTGCGCCGGCCCGTAAACGTACCACTTGATGTCAGCGGGAGACCGGGGTCTCTGAGCTTTAGTGAAGAGATCAAATTTTACGAGCTCGGCGACCAGGCTATTAACAAATTCAGAGAAGATGAAGGCTTCATAAAAGAGGAAGAAAGGCCATTGCCCGACAATGAAAAACGGAGACGCCGAACTGGA
shaker_Frag336-501_insertion396	GTGGGCGTTCGTCTCCTCTCTATTACTATCAATCAGGAGGGCGCTTGCGCCGGCCCGTAAACGTACCACTTGATGTCTTTAGCGGGAGACCGGGGTCTCTGAGCAGTGAAGAGATCAAATTTTACGAGCTCGGCGACCAGGCTATTAACAAATTCAGAGAAGATGAAGGCTTCATAAAAGAGGAAGAAAGGCCATTGCCCGACAATGAAAAACGGAGACGCCGAACTGGA
shaker_Frag336-501_insertion399	GTGGGCGTTCGTCTCCTCTCTATTACTATCAATCAGGAGGGCGCTTGCGCCGGCCCGTAAACGTACCACTTGATGTCTTTAGTAGCGGGAGACCGGGGTCTCTGAGCGAAGAGATCAAATTTTACGAGCTCGGCGACCAGGCTATTAACAAATTCAGAGAAGATGAAGGCTTCATAAAAGAGGAAGAAAGGCCATTGCCCGACAATGAAAAACGGAGACGCCGAACTGGA

shaker_Frag336-501_insertion402	GTGGGCGTTCGTCTCCTCTCTATTACTATCAATCAGGAGGGCGCTTGCGCCGGCCCGTAAACGTACCACTTGATGTCTTTAGTGAAAGCGGGAGACCGGGGTCTCTGAGCGAGATCAAATTTTACGAGCTCGGCGACCAGGCTATTAACAAATTCAGAGAAGATGAAGGCTTCATAAAAGAGGAAGAAAGGCCATTGCCCGACAATGAAAAACGGAGACGCCGAACTGGA
shaker_Frag336-501_insertion405	GTGGGCGTTCGTCTCCTCTCTATTACTATCAATCAGGAGGGCGCTTGCGCCGGCCCGTAAACGTACCACTTGATGTCTTTAGTGAAGAGAGCGGGAGACCGGGGTCTCTGAGCATCAAATTTTACGAGCTCGGCGACCAGGCTATTAACAAATTCAGAGAAGATGAAGGCTTCATAAAAGAGGAAGAAAGGCCATTGCCCGACAATGAAAAACGGAGACGCCGAACTGGA
shaker_Frag336-501_insertion408	GTGGGCGTTCGTCTCCTCTCTATTACTATCAATCAGGAGGGCGCTTGCGCCGGCCCGTAAACGTACCACTTGATGTCTTTAGTGAAGAGATCAGCGGGAGACCGGGGTCTCTGAGCAAATTTTACGAGCTCGGCGACCAGGCTATTAACAAATTCAGAGAAGATGAAGGCTTCATAAAAGAGGAAGAAAGGCCATTGCCCGACAATGAAAAACGGAGACGCCGAACTGGA
shaker_Frag336-501_insertion411	GTGGGCGTTCGTCTCCTCTCTATTACTATCAATCAGGAGGGCGCTTGCGCCGGCCCGTAAACGTACCACTTGATGTCTTTAGTGAAGAGATCAAAAGCGGGAGACCGGGGTCTCTGAGCTTTTACGAGCTCGGCGACCAGGCTATTAACAAATTCAGAGAAGATGAAGGCTTCATAAAAGAGGAAGAAAGGCCATTGCCCGACAATGAAAAACGGAGACGCCGAACTGGA
shaker_Frag336-501_insertion414	GTGGGCGTTCGTCTCCTCTCTATTACTATCAATCAGGAGGGCGCTTGCGCCGGCCCGTAAACGTACCACTTGATGTCTTTAGTGAAGAGATCAAATTTAGCGGGAGACCGGGGTCTCTGAGCTACGAGCTCGGCGACCAGGCTATTAACAAATTCAGAGAAGATGAAGGCTTCATAAAAGAGGAAGAAAGGCCATTGCCCGACAATGAAAAACGGAGACGCCGAACTGGA
shaker_Frag336-501_insertion417	GTGGGCGTTCGTCTCCTCTCTATTACTATCAATCAGGAGGGCGCTTGCGCCGGCCCGTAAACGTACCACTTGATGTCTTTAGTGAAGAGATCAAATTTTACAGCGGGAGACCGGGGTCTCTGAGCGAGCTCGGCGACCAGGCTATTAACAAATTCAGAGAAGATGAAGGCTTCATAAAAGAGGAAGAAAGGCCATTGCCCGACAATGAAAAACGGAGACGCCGAACTGGA
shaker_Frag336-501_insertion420	GTGGGCGTTCGTCTCCTCTCTATTACTATCAATCAGGAGGGCGCTTGCGCCGGCCCGTAAACGTACCACTTGATGTCTTTAGTGAAGAGATCAAATTTTACGAGAGCGGGAGACCGGGGTCTCTGAGCCTCGGCGACCAGGCTATTAACAAATTCAGAGAAGATGAAGGCTTCATAAAAGAGGAAGAAAGGCCATTGCCCGACAATGAAAAACGGAGACGCCGAACTGGA
shaker_Frag336-501_insertion423	GTGGGCGTTCGTCTCCTCTCTATTACTATCAATCAGGAGGGCGCTTGCGCCGGCCCGTAAACGTACCACTTGATGTCTTTAGTGAAGAGATCAAATTTTACGAGCTCAGCGGGAGACCGGGGTCTCTGAGCGGCGACCAGGCTATTAACAAATTCAGAGAAGATGAAGGCTTCATAAAAGAGGAAGAAAGGCCATTGCCCGACAATGAAAAACGGAGACGCCGAACTGGA
shaker_Frag336-501_insertion426	GTGGGCGTTCGTCTCCTCTCTATTACTATCAATCAGGAGGGCGCTTGCGCCGGCCCGTAAACGTACCACTTGATGTCTTTAGTGAAGAGATCAAATTTTACGAGCTCGGCAGCGGGAGACCGGGGTCTCTGAGCGACCAGGCTATTAACAAATTCAGAGAAGATGAAGGCTTCATAAAAGAGGAAGAAAGGCCATTGCCCGACAATGAAAAACGGAGACGCCGAACTGGA
shaker_Frag336-501_insertion429	GTGGGCGTTCGTCTCCTCTCTATTACTATCAATCAGGAGGGCGCTTGCGCCGGCCCGTAAACGTACCACTTGATGTCTTTAGTGAAGAGATCAAATTTTACGAGCTCGGCGACAGCGGGAGACCGGGGTCTCTGAGCCAGGCTATTAACAAATTCAGAGAAGATGAAGGCTTCATAAAAGAGGAAGAAAGGCCATTGCCCGACAATGAAAAACGGAGACGCCGAACTGGA
shaker_Frag336-501_insertion432	GTGGGCGTTCGTCTCCTCTCTATTACTATCAATCAGGAGGGCGCTTGCGCCGGCCCGTAAACGTACCACTTGATGTCTTTAGTGAAGAGATCAAATTTTACGAGCTCGGCGACCAGAGCGGGAGACCGGGGTCTCTGAGCGCTATTAACAAATTCAGAGAAGATGAAGGCTTCATAAAAGAGGAAGAAAGGCCATTGCCCGACAATGAAAAACGGAGACGCCGAACTGGA
shaker_Frag336-501_insertion435	GTGGGCGTTCGTCTCCTCTCTATTACTATCAATCAGGAGGGCGCTTGCGCCGGCCCGTAAACGTACCACTTGATGTCTTTAGTGAAGAGATCAAATTTTACGAGCTCGGCGACCAGGCTAGCGGGAGACCGGGGTCTCTGAGCATTAACAAATTCAGAGAAGATGAAGGCTTCATAAAAGAGGAAGAAAGGCCATTGCCCGACAATGAAAAACGGAGACGCCGAACTGGA
shaker_Frag336-501_insertion438	GTGGGCGTTCGTCTCCTCTCTATTACTATCAATCAGGAGGGCGCTTGCGCCGGCCCGTAAACGTACCACTTGATGTCTTTAGTGAAGAGATCAAATTTTACGAGCTCGGCGACCAGGCTATTAGCGGGAGACCGGGGTCTCTGAGCAACAAATTCAGAGAAGATGAAGGCTTCATAAAAGAGGAAGAAAGGCCATTGCCCGACAATGAAAAACGGAGACGCCGAACTGGA
shaker_Frag336-501_insertion441	GTGGGCGTTCGTCTCCTCTCTATTACTATCAATCAGGAGGGCGCTTGCGCCGGCCCGTAAACGTACCACTTGATGTCTTTAGTGAAGAGATCAAATTTTACGAGCTCGGCGACCAGGCTATTAACAGCGGGAGACCGGGGTCTCTGAGCAAATTCAGAGAAGATGAAGGCTTCATAAAAGAGGAAGAAAGGCCATTGCCCGACAATGAAAAACGGAGACGCCGAACTGGA
shaker_Frag336-501_insertion444	GTGGGCGTTCGTCTCCTCTCTATTACTATCAATCAGGAGGGCGCTTGCGCCGGCCCGTAAACGTACCACTTGATGTCTTTAGTGAAGAGATCAAATTTTACGAGCTCGGCGACCAGGCTATTAACAAAAGCGGGAGACCGGGGTCTCTGAGCTTCAGAGAAGATGAAGGCTTCATAAAAGAGGAAGAAAGGCCATTGCCCGACAATGAAAAACGGAGACGCCGAACTGGA
shaker_Frag336-501_insertion447	GTGGGCGTTCGTCTCCTCTCTATTACTATCAATCAGGAGGGCGCTTGCGCCGGCCCGTAAACGTACCACTTGATGTCTTTAGTGAAGAGATCAAATTTTACGAGCTCGGCGACCAGGCTATTAACAAATTCAGCGGGAGACCGGGGTCTCTGAGCAGAGAAGATGAAGGCTTCATAAAAGAGGAAGAAAGGCCATTGCCCGACAATGAAAAACGGAGACGCCGAACTGGA
shaker_Frag336-501_insertion450	GTGGGCGTTCGTCTCCTCTCTATTACTATCAATCAGGAGGGCGCTTGCGCCGGCCCGTAAACGTACCACTTGATGTCTTTAGTGAAGAGATCAAATTTTACGAGCTCGGCGACCAGGCTATTAACAAATTCAGAAGCGGGAGACCGGGGTCTCTGAGCGAAGATGAAGGCTTCATAAAAGAGGAAGAAAGGCCATTGCCCGACAATGAAAAACGGAGACGCCGAACTGGA
shaker_Frag336-501_insertion453	GTGGGCGTTCGTCTCCTCTCTATTACTATCAATCAGGAGGGCGCTTGCGCCGGCCCGTAAACGTACCACTTGATGTCTTTAGTGAAGAGATCAAATTTTACGAGCTCGGCGACCAGGCTATTAACAAATTCAGAGAAAGCGGGAGACCGGGGTCTCTGAGCGATGAAGGCTTCATAAAAGAGGAAGAAAGGCCATTGCCCGACAATGAAAAACGGAGACGCCGAACTGGA
shaker_Frag336-501_insertion456	GTGGGCGTTCGTCTCCTCTCTATTACTATCAATCAGGAGGGCGCTTGCGCCGGCCCGTAAACGTACCACTTGATGTCTTTAGTGAAGAGATCAAATTTTACGAGCTCGGCGACCAGGCTATTAACAAATTCAGAGAAGATAGCGGGAGACCGGGGTCTCTGAGCGAAGGCTTCATAAAAGAGGAAGAAAGGCCATTGCCCGACAATGAAAAACGGAGACGCCGAACTGGA
shaker_Frag336-501_insertion459	GTGGGCGTTCGTCTCCTCTCTATTACTATCAATCAGGAGGGCGCTTGCGCCGGCCCGTAAACGTACCACTTGATGTCTTTAGTGAAGAGATCAAATTTTACGAGCTCGGCGACCAGGCTATTAACAAATTCAGAGAAGATGAAAGCGGGAGACCGGGGTCTCTGAGCGGCTTCATAAAAGAGGAAGAAAGGCCATTGCCCGACAATGAAAAACGGAGACGCCGAACTGGA
shaker_Frag336-501_insertion462	GTGGGCGTTCGTCTCCTCTCTATTACTATCAATCAGGAGGGCGCTTGCGCCGGCCCGTAAACGTACCACTTGATGTCTTTAGTGAAGAGATCAAATTTTACGAGCTCGGCGACCAGGCTATTAACAAATTCAGAGAAGATGAAGGCAGCGGGAGACCGGGGTCTCTGAGCTTCATAAAAGAGGAAGAAAGGCCATTGCCCGACAATGAAAAACGGAGACGCCGAACTGGA
shaker_Frag336-501_insertion465	GTGGGCGTTCGTCTCCTCTCTATTACTATCAATCAGGAGGGCGCTTGCGCCGGCCCGTAAACGTACCACTTGATGTCTTTAGTGAAGAGATCAAATTTTACGAGCTCGGCGACCAGGCTATTAACAAATTCAGAGAAGATGAAGGCTTCAGCGGGAGACCGGGGTCTCTGAGCATAAAAGAGGAAGAAAGGCCATTGCCCGACAATGAAAAACGGAGACGCCGAACTGGA
shaker_Frag336-501_insertion468	GTGGGCGTTCGTCTCCTCTCTATTACTATCAATCAGGAGGGCGCTTGCGCCGGCCCGTAAACGTACCACTTGATGTCTTTAGTGAAGAGATCAAATTTTACGAGCTCGGCGACCAGGCTATTAACAAATTCAGAGAAGATGAAGGCTTCATAAGCGGGAGACCGGGGTCTCTGAGCAAAGAGGAAGAAAGGCCATTGCCCGACAATGAAAAACGGAGACGCCGAACTGGA
shaker_Frag336-501_insertion471	GTGGGCGTTCGTCTCCTCTCTATTACTATCAATCAGGAGGGCGCTTGCGCCGGCCCGTAAACGTACCACTTGATGTCTTTAGTGAAGAGATCAAATTTTACGAGCTCGGCGACCAGGCTATTAACAAATTCAGAGAAGATGAAGGCTTCATAAAAAGCGGGAGACCGGGGTCTCTGAGCGAGGAAGAAAGGCCATTGCCCGACAATGAAAAACGGAGACGCCGAACTGGA
shaker_Frag336-501_insertion474	GTGGGCGTTCGTCTCCTCTCTATTACTATCAATCAGGAGGGCGCTTGCGCCGGCCCGTAAACGTACCACTTGATGTCTTTAGTGAAGAGATCAAATTTTACGAGCTCGGCGACCAGGCTATTAACAAATTCAGAGAAGATGAAGGCTTCATAAAAGAGAGCGGGAGACCGGGGTCTCTGAGCGAAGAAAGGCCATTGCCCGACAATGAAAAACGGAGACGCCGAACTGGA
shaker_Frag336-501_insertion477	GTGGGCGTTCGTCTCCTCTCTATTACTATCAATCAGGAGGGCGCTTGCGCCGGCCCGTAAACGTACCACTTGATGTCTTTAGTGAAGAGATCAAATTTTACGAGCTCGGCGACCAGGCTATTAACAAATTCAGAGAAGATGAAGGCTTCATAAAAGAGGAAAGCGGGAGACCGGGGTCTCTGAGCGAAAGGCCATTGCCCGACAATGAAAAACGGAGACGCCGAACTGGA
shaker_Frag336-501_insertion480	GTGGGCGTTCGTCTCCTCTCTATTACTATCAATCAGGAGGGCGCTTGCGCCGGCCCGTAAACGTACCACTTGATGTCTTTAGTGAAGAGATCAAATTTTACGAGCTCGGCGACCAGGCTATTAACAAATTCAGAGAAGATGAAGGCTTCATAAAAGAGGAAGAAAGCGGGAGACCGGGGTCTCTGAGCAGGCCATTGCCCGACAATGAAAAACGGAGACGCCGAACTGGA
shaker_Frag336-501_insertion483	GTGGGCGTTCGTCTCCTCTCTATTACTATCAATCAGGAGGGCGCTTGCGCCGGCCCGTAAACGTACCACTTGATGTCTTTAGTGAAGAGATCAAATTTTACGAGCTCGGCGACCAGGCTATTAACAAATTCAGAGAAGATGAAGGCTTCATAAAAGAGGAAGAAAGGAGCGGGAGACCGGGGTCTCTGAGCCCATTGCCCGACAATGAAAAACGGAGACGCCGAACTGGA
shaker_Frag336-501_insertion486	GTGGGCGTTCGTCTCCTCTCTATTACTATCAATCAGGAGGGCGCTTGCGCCGGCCCGTAAACGTACCACTTGATGTCTTTAGTGAAGAGATCAAATTTTACGAGCTCGGCGACCAGGCTATTAACAAATTCAGAGAAGATGAAGGCTTCATAAAAGAGGAAGAAAGGCCAAGCGGGAGACCGGGGTCTCTGAGCTTGCCCGACAATGAAAAACGGAGACGCCGAACTGGA
shaker_Frag336-501_insertion489	GTGGGCGTTCGTCTCCTCTCTATTACTATCAATCAGGAGGGCGCTTGCGCCGGCCCGTAAACGTACCACTTGATGTCTTTAGTGAAGAGATCAAATTTTACGAGCTCGGCGACCAGGCTATTAACAAATTCAGAGAAGATGAAGGCTTCATAAAAGAGGAAGAAAGGCCATTGAGCGGGAGACCGGGGTCTCTGAGCCCCGACAATGAAAAACGGAGACGCCGAACTGGA
shaker_Frag336-501_insertion492	GTGGGCGTTCGTCTCCTCTCTATTACTATCAATCAGGAGGGCGCTTGCGCCGGCCCGTAAACGTACCACTTGATGTCTTTAGTGAAGAGATCAAATTTTACGAGCTCGGCGACCAGGCTATTAACAAATTCAGAGAAGATGAAGGCTTCATAAAAGAGGAAGAAAGGCCATTGCCCAGCGGGAGACCGGGGTCTCTGAGCGACAATGAAAAACGGAGACGCCGAACTGGA
shaker_Frag336-501_insertion495	GTGGGCGTTCGTCTCCTCTCTATTACTATCAATCAGGAGGGCGCTTGCGCCGGCCCGTAAACGTACCACTTGATGTCTTTAGTGAAGAGATCAAATTTTACGAGCTCGGCGACCAGGCTATTAACAAATTCAGAGAAGATGAAGGCTTCATAAAAGAGGAAGAAAGGCCATTGCCCGACAGCGGGAGACCGGGGTCTCTGAGCAATGAAAAACGGAGACGCCGAACTGGA
shaker_Frag336-501_insertion498	GTGGGCGTTCGTCTCCTCTCTATTACTATCAATCAGGAGGGCGCTTGCGCCGGCCCGTAAACGTACCACTTGATGTCTTTAGTGAAGAGATCAAATTTTACGAGCTCGGCGACCAGGCTATTAACAAATTCAGAGAAGATGAAGGCTTCATAAAAGAGGAAGAAAGGCCATTGCCCGACAATAGCGGGAGACCGGGGTCTCTGAGCGAAAAACGGAGACGCCGAACTGGA
shaker_Frag336-501_insertion501	GTGGGCGTTCGTCTCCTCTCTATTACTATCAATCAGGAGGGCGCTTGCGCCGGCCCGTAAACGTACCACTTGATGTCTTTAGTGAAGAGATCAAATTTTACGAGCTCGGCGACCAGGCTATTAACAAATTCAGAGAAGATGAAGGCTTCATAAAAGAGGAAGAAAGGCCATTGCCCGACAATGAAAGCGGGAGACCGGGGTCTCTGAGCAAACGGAGACGCCGAACTGGA
shaker_Frag504-669_insertion504	TCGCGTGAGCGTCTCCAAAAAGCGGGAGACCGGGGTCTCTGAGCCAGCGGAAAGTGTGGCTGCTGTTTGAGTACCCTGAGAGCTCACAAGCAGCACGCGTAGTCGCAATCATTTCTGTATTTGTGATTCTCCTTAGTATTGTTATCTTTTGTCTCGAAACTCTCCCTGAGTTCAAGCACTACAAAGTTTTCAATACAACTACCAACGGCACCAGGAGACGAACGGCGGAA
shaker_Frag504-669_insertion507	TCGCGTGAGCGTCTCCAAAACAGAGCGGGAGACCGGGGTCTCTGAGCCGGAAAGTGTGGCTGCTGTTTGAGTACCCTGAGAGCTCACAAGCAGCACGCGTAGTCGCAATCATTTCTGTATTTGTGATTCTCCTTAGTATTGTTATCTTTTGTCTCGAAACTCTCCCTGAGTTCAAGCACTACAAAGTTTTCAATACAACTACCAACGGCACCAGGAGACGAACGGCGGAA
shaker_Frag504-669_insertion510	TCGCGTGAGCGTCTCCAAAACAGCGGAGCGGGAGACCGGGGTCTCTGAGCAAAGTGTGGCTGCTGTTTGAGTACCCTGAGAGCTCACAAGCAGCACGCGTAGTCGCAATCATTTCTGTATTTGTGATTCTCCTTAGTATTGTTATCTTTTGTCTCGAAACTCTCCCTGAGTTCAAGCACTACAAAGTTTTCAATACAACTACCAACGGCACCAGGAGACGAACGGCGGAA
shaker_Frag504-669_insertion513	TCGCGTGAGCGTCTCCAAAACAGCGGAAAAGCGGGAGACCGGGGTCTCTGAGCGTGTGGCTGCTGTTTGAGTACCCTGAGAGCTCACAAGCAGCACGCGTAGTCGCAATCATTTCTGTATTTGTGATTCTCCTTAGTATTGTTATCTTTTGTCTCGAAACTCTCCCTGAGTTCAAGCACTACAAAGTTTTCAATACAACTACCAACGGCACCAGGAGACGAACGGCGGAA
shaker_Frag504-669_insertion516	TCGCGTGAGCGTCTCCAAAACAGCGGAAAGTGAGCGGGAGACCGGGGTCTCTGAGCTGGCTGCTGTTTGAGTACCCTGAGAGCTCACAAGCAGCACGCGTAGTCGCAATCATTTCTGTATTTGTGATTCTCCTTAGTATTGTTATCTTTTGTCTCGAAACTCTCCCTGAGTTCAAGCACTACAAAGTTTTCAATACAACTACCAACGGCACCAGGAGACGAACGGCGGAA
shaker_Frag504-669_insertion519	TCGCGTGAGCGTCTCCAAAACAGCGGAAAGTGTGGAGCGGGAGACCGGGGTCTCTGAGCCTGCTGTTTGAGTACCCTGAGAGCTCACAAGCAGCACGCGTAGTCGCAATCATTTCTGTATTTGTGATTCTCCTTAGTATTGTTATCTTTTGTCTCGAAACTCTCCCTGAGTTCAAGCACTACAAAGTTTTCAATACAACTACCAACGGCACCAGGAGACGAACGGCGGAA
shaker_Frag504-669_insertion522	TCGCGTGAGCGTCTCCAAAACAGCGGAAAGTGTGGCTGAGCGGGAGACCGGGGTCTCTGAGCCTGTTTGAGTACCCTGAGAGCTCACAAGCAGCACGCGTAGTCGCAATCATTTCTGTATTTGTGATTCTCCTTAGTATTGTTATCTTTTGTCTCGAAACTCTCCCTGAGTTCAAGCACTACAAAGTTTTCAATACAACTACCAACGGCACCAGGAGACGAACGGCGGAA
shaker_Frag504-669_insertion525	TCGCGTGAGCGTCTCCAAAACAGCGGAAAGTGTGGCTGCTGAGCGGGAGACCGGGGTCTCTGAGCTTTGAGTACCCTGAGAGCTCACAAGCAGCACGCGTAGTCGCAATCATTTCTGTATTTGTGATTCTCCTTAGTATTGTTATCTTTTGTCTCGAAACTCTCCCTGAGTTCAAGCACTACAAAGTTTTCAATACAACTACCAACGGCACCAGGAGACGAACGGCGGAA
shaker_Frag504-669_insertion528	TCGCGTGAGCGTCTCCAAAACAGCGGAAAGTGTGGCTGCTGTTTAGCGGGAGACCGGGGTCTCTGAGCGAGTACCCTGAGAGCTCACAAGCAGCACGCGTAGTCGCAATCATTTCTGTATTTGTGATTCTCCTTAGTATTGTTATCTTTTGTCTCGAAACTCTCCCTGAGTTCAAGCACTACAAAGTTTTCAATACAACTACCAACGGCACCAGGAGACGAACGGCGGAA
shaker_Frag504-669_insertion531	TCGCGTGAGCGTCTCCAAAACAGCGGAAAGTGTGGCTGCTGTTTGAGAGCGGGAGACCGGGGTCTCTGAGCTACCCTGAGAGCTCACAAGCAGCACGCGTAGTCGCAATCATTTCTGTATTTGTGATTCTCCTTAGTATTGTTATCTTTTGTCTCGAAACTCTCCCTGAGTTCAAGCACTACAAAGTTTTCAATACAACTACCAACGGCACCAGGAGACGAACGGCGGAA
shaker_Frag504-669_insertion534	TCGCGTGAGCGTCTCCAAAACAGCGGAAAGTGTGGCTGCTGTTTGAGTACAGCGGGAGACCGGGGTCTCTGAGCCCTGAGAGCTCACAAGCAGCACGCGTAGTCGCAATCATTTCTGTATTTGTGATTCTCCTTAGTATTGTTATCTTTTGTCTCGAAACTCTCCCTGAGTTCAAGCACTACAAAGTTTTCAATACAACTACCAACGGCACCAGGAGACGAACGGCGGAA
shaker_Frag504-669_insertion537	TCGCGTGAGCGTCTCCAAAACAGCGGAAAGTGTGGCTGCTGTTTGAGTACCCTAGCGGGAGACCGGGGTCTCTGAGCGAGAGCTCACAAGCAGCACGCGTAGTCGCAATCATTTCTGTATTTGTGATTCTCCTTAGTATTGTTATCTTTTGTCTCGAAACTCTCCCTGAGTTCAAGCACTACAAAGTTTTCAATACAACTACCAACGGCACCAGGAGACGAACGGCGGAA
shaker_Frag504-669_insertion540	TCGCGTGAGCGTCTCCAAAACAGCGGAAAGTGTGGCTGCTGTTTGAGTACCCTGAGAGCGGGAGACCGGGGTCTCTGAGCAGCTCACAAGCAGCACGCGTAGTCGCAATCATTTCTGTATTTGTGATTCTCCTTAGTATTGTTATCTTTTGTCTCGAAACTCTCCCTGAGTTCAAGCACTACAAAGTTTTCAATACAACTACCAACGGCACCAGGAGACGAACGGCGGAA
shaker_Frag504-669_insertion543	TCGCGTGAGCGTCTCCAAAACAGCGGAAAGTGTGGCTGCTGTTTGAGTACCCTGAGAGCAGCGGGAGACCGGGGTCTCTGAGCTCACAAGCAGCACGCGTAGTCGCAATCATTTCTGTATTTGTGATTCTCCTTAGTATTGTTATCTTTTGTCTCGAAACTCTCCCTGAGTTCAAGCACTACAAAGTTTTCAATACAACTACCAACGGCACCAGGAGACGAACGGCGGAA
shaker_Frag504-669_insertion546	TCGCGTGAGCGTCTCCAAAACAGCGGAAAGTGTGGCTGCTGTTTGAGTACCCTGAGAGCTCAAGCGGGAGACCGGGGTCTCTGAGCCAAGCAGCACGCGTAGTCGCAATCATTTCTGTATTTGTGATTCTCCTTAGTATTGTTATCTTTTGTCTCGAAACTCTCCCTGAGTTCAAGCACTACAAAGTTTTCAATACAACTACCAACGGCACCAGGAGACGAACGGCGGAA
shaker_Frag504-669_insertion549	TCGCGTGAGCGTCTCCAAAACAGCGGAAAGTGTGGCTGCTGTTTGAGTACCCTGAGAGCTCACAAAGCGGGAGACCGGGGTCTCTGAGCGCAGCACGCGTAGTCGCAATCATTTCTGTATTTGTGATTCTCCTTAGTATTGTTATCTTTTGTCTCGAAACTCTCCCTGAGTTCAAGCACTACAAAGTTTTCAATACAACTACCAACGGCACCAGGAGACGAACGGCGGAA
shaker_Frag504-669_insertion552	TCGCGTGAGCGTCTCCAAAACAGCGGAAAGTGTGGCTGCTGTTTGAGTACCCTGAGAGCTCACAAGCAAGCGGGAGACCGGGGTCTCTGAGCGCACGCGTAGTCGCAATCATTTCTGTATTTGTGATTCTCCTTAGTATTGTTATCTTTTGTCTCGAAACTCTCCCTGAGTTCAAGCACTACAAAGTTTTCAATACAACTACCAACGGCACCAGGAGACGAACGGCGGAA
shaker_Frag504-669_insertion555	TCGCGTGAGCGTCTCCAAAACAGCGGAAAGTGTGGCTGCTGTTTGAGTACCCTGAGAGCTCACAAGCAGCAAGCGGGAGACCGGGGTCTCTGAGCCGCGTAGTCGCAATCATTTCTGTATTTGTGATTCTCCTTAGTATTGTTATCTTTTGTCTCGAAACTCTCCCTGAGTTCAAGCACTACAAAGTTTTCAATACAACTACCAACGGCACCAGGAGACGAACGGCGGAA
shaker_Frag504-669_insertion558	TCGCGTGAGCGTCTCCAAAACAGCGGAAAGTGTGGCTGCTGTTTGAGTACCCTGAGAGCTCACAAGCAGCACGCAGCGGGAGACCGGGGTCTCTGAGCGTAGTCGCAATCATTTCTGTATTTGTGATTCTCCTTAGTATTGTTATCTTTTGTCTCGAAACTCTCCCTGAGTTCAAGCACTACAAAGTTTTCAATACAACTACCAACGGCACCAGGAGACGAACGGCGGAA
shaker_Frag504-669_insertion561	TCGCGTGAGCGTCTCCAAAACAGCGGAAAGTGTGGCTGCTGTTTGAGTACCCTGAGAGCTCACAAGCAGCACGCGTAAGCGGGAGACCGGGGTCTCTGAGCGTCGCAATCATTTCTGTATTTGTGATTCTCCTTAGTATTGTTATCTTTTGTCTCGAAACTCTCCCTGAGTTCAAGCACTACAAAGTTTTCAATACAACTACCAACGGCACCAGGAGACGAACGGCGGAA
shaker_Frag504-669_insertion564	TCGCGTGAGCGTCTCCAAAACAGCGGAAAGTGTGGCTGCTGTTTGAGTACCCTGAGAGCTCACAAGCAGCACGCGTAGTCAGCGGGAGACCGGGGTCTCTGAGCGCAATCATTTCTGTATTTGTGATTCTCCTTAGTATTGTTATCTTTTGTCTCGAAACTCTCCCTGAGTTCAAGCACTACAAAGTTTTCAATACAACTACCAACGGCACCAGGAGACGAACGGCGGAA
shaker_Frag504-669_insertion567	TCGCGTGAGCGTCTCCAAAACAGCGGAAAGTGTGGCTGCTGTTTGAGTACCCTGAGAGCTCACAAGCAGCACGCGTAGTCGCAAGCGGGAGACCGGGGTCTCTGAGCATCATTTCTGTATTTGTGATTCTCCTTAGTATTGTTATCTTTTGTCTCGAAACTCTCCCTGAGTTCAAGCACTACAAAGTTTTCAATACAACTACCAACGGCACCAGGAGACGAACGGCGGAA
shaker_Frag504-669_insertion570	TCGCGTGAGCGTCTCCAAAACAGCGGAAAGTGTGGCTGCTGTTTGAGTACCCTGAGAGCTCACAAGCAGCACGCGTAGTCGCAATCAGCGGGAGACCGGGGTCTCTGAGCATTTCTGTATTTGTGATTCTCCTTAGTATTGTTATCTTTTGTCTCGAAACTCTCCCTGAGTTCAAGCACTACAAAGTTTTCAATACAACTACCAACGGCACCAGGAGACGAACGGCGGAA
shaker_Frag504-669_insertion573	TCGCGTGAGCGTCTCCAAAACAGCGGAAAGTGTGGCTGCTGTTTGAGTACCCTGAGAGCTCACAAGCAGCACGCGTAGTCGCAATCATTAGCGGGAGACCGGGGTCTCTGAGCTCTGTATTTGTGATTCTCCTTAGTATTGTTATCTTTTGTCTCGAAACTCTCCCTGAGTTCAAGCACTACAAAGTTTTCAATACAACTACCAACGGCACCAGGAGACGAACGGCGGAA
shaker_Frag504-669_insertion576	TCGCGTGAGCGTCTCCAAAACAGCGGAAAGTGTGGCTGCTGTTTGAGTACCCTGAGAGCTCACAAGCAGCACGCGTAGTCGCAATCATTTCTAGCGGGAGACCGGGGTCTCTGAGCGTATTTGTGATTCTCCTTAGTATTGTTATCTTTTGTCTCGAAACTCTCCCTGAGTTCAAGCACTACAAAGTTTTCAATACAACTACCAACGGCACCAGGAGACGAACGGCGGAA
shaker_Frag504-669_insertion579	TCGCGTGAGCGTCTCCAAAACAGCGGAAAGTGTGGCTGCTGTTTGAGTACCCTGAGAGCTCACAAGCAGCACGCGTAGTCGCAATCATTTCTGTAAGCGGGAGACCGGGGTCTCTGAGCTTTGTGATTCTCCTTAGTATTGTTATCTTTTGTCTCGAAACTCTCCCTGAGTTCAAGCACTACAAAGTTTTCAATACAACTACCAACGGCACCAGGAGACGAACGGCGGAA
shaker_Frag504-669_insertion582	TCGCGTGAGCGTCTCCAAAACAGCGGAAAGTGTGGCTGCTGTTTGAGTACCCTGAGAGCTCACAAGCAGCACGCGTAGTCGCAATCATTTCTGTATTTAGCGGGAGACCGGGGTCTCTGAGCGTGATTCTCCTTAGTATTGTTATCTTTTGTCTCGAAACTCTCCCTGAGTTCAAGCACTACAAAGTTTTCAATACAACTACCAACGGCACCAGGAGACGAACGGCGGAA
shaker_Frag504-669_insertion585	TCGCGTGAGCGTCTCCAAAACAGCGGAAAGTGTGGCTGCTGTTTGAGTACCCTGAGAGCTCACAAGCAGCACGCGTAGTCGCAATCATTTCTGTATTTGTGAGCGGGAGACCGGGGTCTCTGAGCATTCTCCTTAGTATTGTTATCTTTTGTCTCGAAACTCTCCCTGAGTTCAAGCACTACAAAGTTTTCAATACAACTACCAACGGCACCAGGAGACGAACGGCGGAA
shaker_Frag504-669_insertion588	TCGCGTGAGCGTCTCCAAAACAGCGGAAAGTGTGGCTGCTGTTTGAGTACCCTGAGAGCTCACAAGCAGCACGCGTAGTCGCAATCATTTCTGTATTTGTGATTAGCGGGAGACCGGGGTCTCTGAGCCTCCTTAGTATTGTTATCTTTTGTCTCGAAACTCTCCCTGAGTTCAAGCACTACAAAGTTTTCAATACAACTACCAACGGCACCAGGAGACGAACGGCGGAA
shaker_Frag504-669_insertion591	TCGCGTGAGCGTCTCCAAAACAGCGGAAAGTGTGGCTGCTGTTTGAGTACCCTGAGAGCTCACAAGCAGCACGCGTAGTCGCAATCATTTCTGTATTTGTGATTCTCAGCGGGAGACCGGGGTCTCTGAGCCTTAGTATTGTTATCTTTTGTCTCGAAACTCTCCCTGAGTTCAAGCACTACAAAGTTTTCAATACAACTACCAACGGCACCAGGAGACGAACGGCGGAA
shaker_Frag504-669_insertion594	TCGCGTGAGCGTCTCCAAAACAGCGGAAAGTGTGGCTGCTGTTTGAGTACCCTGAGAGCTCACAAGCAGCACGCGTAGTCGCAATCATTTCTGTATTTGTGATTCTCCTTAGCGGGAGACCGGGGTCTCTGAGCAGTATTGTTATCTTTTGTCTCGAAACTCTCCCTGAGTTCAAGCACTACAAAGTTTTCAATACAACTACCAACGGCACCAGGAGACGAACGGCGGAA
shaker_Frag504-669_insertion597	TCGCGTGAGCGTCTCCAAAACAGCGGAAAGTGTGGCTGCTGTTTGAGTACCCTGAGAGCTCACAAGCAGCACGCGTAGTCGCAATCATTTCTGTATTTGTGATTCTCCTTAGTAGCGGGAGACCGGGGTCTCTGAGCATTGTTATCTTTTGTCTCGAAACTCTCCCTGAGTTCAAGCACTACAAAGTTTTCAATACAACTACCAACGGCACCAGGAGACGAACGGCGGAA
shaker_Frag504-669_insertion600	TCGCGTGAGCGTCTCCAAAACAGCGGAAAGTGTGGCTGCTGTTTGAGTACCCTGAGAGCTCACAAGCAGCACGCGTAGTCGCAATCATTTCTGTATTTGTGATTCTCCTTAGTATTAGCGGGAGACCGGGGTCTCTGAGCGTTATCTTTTGTCTCGAAACTCTCCCTGAGTTCAAGCACTACAAAGTTTTCAATACAACTACCAACGGCACCAGGAGACGAACGGCGGAA
shaker_Frag504-669_insertion603	TCGCGTGAGCGTCTCCAAAACAGCGGAAAGTGTGGCTGCTGTTTGAGTACCCTGAGAGCTCACAAGCAGCACGCGTAGTCGCAATCATTTCTGTATTTGTGATTCTCCTTAGTATTGTTAGCGGGAGACCGGGGTCTCTGAGCATCTTTTGTCTCGAAACTCTCCCTGAGTTCAAGCACTACAAAGTTTTCAATACAACTACCAACGGCACCAGGAGACGAACGGCGGAA
shaker_Frag504-669_insertion606	TCGCGTGAGCGTCTCCAAAACAGCGGAAAGTGTGGCTGCTGTTTGAGTACCCTGAGAGCTCACAAGCAGCACGCGTAGTCGCAATCATTTCTGTATTTGTGATTCTCCTTAGTATTGTTATCAGCGGGAGACCGGGGTCTCTGAGCTTTTGTCTCGAAACTCTCCCTGAGTTCAAGCACTACAAAGTTTTCAATACAACTACCAACGGCACCAGGAGACGAACGGCGGAA
shaker_Frag504-669_insertion609	TCGCGTGAGCGTCTCCAAAACAGCGGAAAGTGTGGCTGCTGTTTGAGTACCCTGAGAGCTCACAAGCAGCACGCGTAGTCGCAATCATTTCTGTATTTGTGATTCTCCTTAGTATTGTTATCTTTAGCGGGAGACCGGGGTCTCTGAGCTGTCTCGAAACTCTCCCTGAGTTCAAGCACTACAAAGTTTTCAATACAACTACCAACGGCACCAGGAGACGAACGGCGGAA
shaker_Frag504-669_insertion612	TCGCGTGAGCGTCTCCAAAACAGCGGAAAGTGTGGCTGCTGTTTGAGTACCCTGAGAGCTCACAAGCAGCACGCGTAGTCGCAATCATTTCTGTATTTGTGATTCTCCTTAGTATTGTTATCTTTTGTAGCGGGAGACCGGGGTCTCTGAGCCTCGAAACTCTCCCTGAGTTCAAGCACTACAAAGTTTTCAATACAACTACCAACGGCACCAGGAGACGAACGGCGGAA
shaker_Frag504-669_insertion615	TCGCGTGAGCGTCTCCAAAACAGCGGAAAGTGTGGCTGCTGTTTGAGTACCCTGAGAGCTCACAAGCAGCACGCGTAGTCGCAATCATTTCTGTATTTGTGATTCTCCTTAGTATTGTTATCTTTTGTCTCAGCGGGAGACCGGGGTCTCTGAGCGAAACTCTCCCTGAGTTCAAGCACTACAAAGTTTTCAATACAACTACCAACGGCACCAGGAGACGAACGGCGGAA
shaker_Frag504-669_insertion618	TCGCGTGAGCGTCTCCAAAACAGCGGAAAGTGTGGCTGCTGTTTGAGTACCCTGAGAGCTCACAAGCAGCACGCGTAGTCGCAATCATTTCTGTATTTGTGATTCTCCTTAGTATTGTTATCTTTTGTCTCGAAAGCGGGAGACCGGGGTCTCTGAGCACTCTCCCTGAGTTCAAGCACTACAAAGTTTTCAATACAACTACCAACGGCACCAGGAGACGAACGGCGGAA
shaker_Frag504-669_insertion621	TCGCGTGAGCGTCTCCAAAACAGCGGAAAGTGTGGCTGCTGTTTGAGTACCCTGAGAGCTCACAAGCAGCACGCGTAGTCGCAATCATTTCTGTATTTGTGATTCTCCTTAGTATTGTTATCTTTTGTCTCGAAACTAGCGGGAGACCGGGGTCTCTGAGCCTCCCTGAGTTCAAGCACTACAAAGTTTTCAATACAACTACCAACGGCACCAGGAGACGAACGGCGGAA
shaker_Frag504-669_insertion624	TCGCGTGAGCGTCTCCAAAACAGCGGAAAGTGTGGCTGCTGTTTGAGTACCCTGAGAGCTCACAAGCAGCACGCGTAGTCGCAATCATTTCTGTATTTGTGATTCTCCTTAGTATTGTTATCTTTTGTCTCGAAACTCTCAGCGGGAGACCGGGGTCTCTGAGCCCTGAGTTCAAGCACTACAAAGTTTTCAATACAACTACCAACGGCACCAGGAGACGAACGGCGGAA
shaker_Frag504-669_insertion627	TCGCGTGAGCGTCTCCAAAACAGCGGAAAGTGTGGCTGCTGTTTGAGTACCCTGAGAGCTCACAAGCAGCACGCGTAGTCGCAATCATTTCTGTATTTGTGATTCTCCTTAGTATTGTTATCTTTTGTCTCGAAACTCTCCCTAGCGGGAGACCGGGGTCTCTGAGCGAGTTCAAGCACTACAAAGTTTTCAATACAACTACCAACGGCACCAGGAGACGAACGGCGGAA
shaker_Frag504-669_insertion630	TCGCGTGAGCGTCTCCAAAACAGCGGAAAGTGTGGCTGCTGTTTGAGTACCCTGAGAGCTCACAAGCAGCACGCGTAGTCGCAATCATTTCTGTATTTGTGATTCTCCTTAGTATTGTTATCTTTTGTCTCGAAACTCTCCCTGAGAGCGGGAGACCGGGGTCTCTGAGCTTCAAGCACTACAAAGTTTTCAATACAACTACCAACGGCACCAGGAGACGAACGGCGGAA
shaker_Frag504-669_insertion633	TCGCGTGAGCGTCTCCAAAACAGCGGAAAGTGTGGCTGCTGTTTGAGTACCCTGAGAGCTCACAAGCAGCACGCGTAGTCGCAATCATTTCTGTATTTGTGATTCTCCTTAGTATTGTTATCTTTTGTCTCGAAACTCTCCCTGAGTTCAGCGGGAGACCGGGGTCTCTGAGCAAGCACTACAAAGTTTTCAATACAACTACCAACGGCACCAGGAGACGAACGGCGGAA
shaker_Frag504-669_insertion636	TCGCGTGAGCGTCTCCAAAACAGCGGAAAGTGTGGCTGCTGTTTGAGTACCCTGAGAGCTCACAAGCAGCACGCGTAGTCGCAATCATTTCTGTATTTGTGATTCTCCTTAGTATTGTTATCTTTTGTCTCGAAACTCTCCCTGAGTTCAAGAGCGGGAGACCGGGGTCTCTGAGCCACTACAAAGTTTTCAATACAACTACCAACGGCACCAGGAGACGAACGGCGGAA
shaker_Frag504-669_insertion639	TCGCGTGAGCGTCTCCAAAACAGCGGAAAGTGTGGCTGCTGTTTGAGTACCCTGAGAGCTCACAAGCAGCACGCGTAGTCGCAATCATTTCTGTATTTGTGATTCTCCTTAGTATTGTTATCTTTTGTCTCGAAACTCTCCCTGAGTTCAAGCACAGCGGGAGACCGGGGTCTCTGAGCTACAAAGTTTTCAATACAACTACCAACGGCACCAGGAGACGAACGGCGGAA
shaker_Frag504-669_insertion642	TCGCGTGAGCGTCTCCAAAACAGCGGAAAGTGTGGCTGCTGTTTGAGTACCCTGAGAGCTCACAAGCAGCACGCGTAGTCGCAATCATTTCTGTATTTGTGATTCTCCTTAGTATTGTTATCTTTTGTCTCGAAACTCTCCCTGAGTTCAAGCACTACAGCGGGAGACCGGGGTCTCTGAGCAAAGTTTTCAATACAACTACCAACGGCACCAGGAGACGAACGGCGGAA
shaker_Frag504-669_insertion645	TCGCGTGAGCGTCTCCAAAACAGCGGAAAGTGTGGCTGCTGTTTGAGTACCCTGAGAGCTCACAAGCAGCACGCGTAGTCGCAATCATTTCTGTATTTGTGATTCTCCTTAGTATTGTTATCTTTTGTCTCGAAACTCTCCCTGAGTTCAAGCACTACAAAAGCGGGAGACCGGGGTCTCTGAGCGTTTTCAATACAACTACCAACGGCACCAGGAGACGAACGGCGGAA
shaker_Frag504-669_insertion648	TCGCGTGAGCGTCTCCAAAACAGCGGAAAGTGTGGCTGCTGTTTGAGTACCCTGAGAGCTCACAAGCAGCACGCGTAGTCGCAATCATTTCTGTATTTGTGATTCTCCTTAGTATTGTTATCTTTTGTCTCGAAACTCTCCCTGAGTTCAAGCACTACAAAGTTAGCGGGAGACCGGGGTCTCTGAGCTTCAATACAACTACCAACGGCACCAGGAGACGAACGGCGGAA
shaker_Frag504-669_insertion651	TCGCGTGAGCGTCTCCAAAACAGCGGAAAGTGTGGCTGCTGTTTGAGTACCCTGAGAGCTCACAAGCAGCACGCGTAGTCGCAATCATTTCTGTATTTGTGATTCTCCTTAGTATTGTTATCTTTTGTCTCGAAACTCTCCCTGAGTTCAAGCACTACAAAGTTTTCAGCGGGAGACCGGGGTCTCTGAGCAATACAACTACCAACGGCACCAGGAGACGAACGGCGGAA
shaker_Frag504-669_insertion654	TCGCGTGAGCGTCTCCAAAACAGCGGAAAGTGTGGCTGCTGTTTGAGTACCCTGAGAGCTCACAAGCAGCACGCGTAGTCGCAATCATTTCTGTATTTGTGATTCTCCTTAGTATTGTTATCTTTTGTCTCGAAACTCTCCCTGAGTTCAAGCACTACAAAGTTTTCAATAGCGGGAGACCGGGGTCTCTGAGCACAACTACCAACGGCACCAGGAGACGAACGGCGGAA
shaker_Frag504-669_insertion657	TCGCGTGAGCGTCTCCAAAACAGCGGAAAGTGTGGCTGCTGTTTGAGTACCCTGAGAGCTCACAAGCAGCACGCGTAGTCGCAATCATTTCTGTATTTGTGATTCTCCTTAGTATTGTTATCTTTTGTCTCGAAACTCTCCCTGAGTTCAAGCACTACAAAGTTTTCAATACAAGCGGGAGACCGGGGTCTCTGAGCACTACCAACGGCACCAGGAGACGAACGGCGGAA
shaker_Frag504-669_insertion660	TCGCGTGAGCGTCTCCAAAACAGCGGAAAGTGTGGCTGCTGTTTGAGTACCCTGAGAGCTCACAAGCAGCACGCGTAGTCGCAATCATTTCTGTATTTGTGATTCTCCTTAGTATTGTTATCTTTTGTCTCGAAACTCTCCCTGAGTTCAAGCACTACAAAGTTTTCAATACAACTAGCGGGAGACCGGGGTCTCTGAGCACCAACGGCACCAGGAGACGAACGGCGGAA
shaker_Frag504-669_insertion663	TCGCGTGAGCGTCTCCAAAACAGCGGAAAGTGTGGCTGCTGTTTGAGTACCCTGAGAGCTCACAAGCAGCACGCGTAGTCGCAATCATTTCTGTATTTGTGATTCTCCTTAGTATTGTTATCTTTTGTCTCGAAACTCTCCCTGAGTTCAAGCACTACAAAGTTTTCAATACAACTACCAGCGGGAGACCGGGGTCTCTGAGCAACGGCACCAGGAGACGAACGGCGGAA
shaker_Frag504-669_insertion666	TCGCGTGAGCGTCTCCAAAACAGCGGAAAGTGTGGCTGCTGTTTGAGTACCCTGAGAGCTCACAAGCAGCACGCGTAGTCGCAATCATTTCTGTATTTGTGATTCTCCTTAGTATTGTTATCTTTTGTCTCGAAACTCTCCCTGAGTTCAAGCACTACAAAGTTTTCAATACAACTACCAACAGCGGGAGACCGGGGTCTCTGAGCGGCACCAGGAGACGAACGGCGGAA
shaker_Frag504-669_insertion669	TCGCGTGAGCGTCTCCAAAACAGCGGAAAGTGTGGCTGCTGTTTGAGTACCCTGAGAGCTCACAAGCAGCACGCGTAGTCGCAATCATTTCTGTATTTGTGATTCTCCTTAGTATTGTTATCTTTTGTCTCGAAACTCTCCCTGAGTTCAAGCACTACAAAGTTTTCAATACAACTACCAACGGCAGCGGGAGACCGGGGTCTCTGAGCACCAGGAGACGAACGGCGGAA
shaker_Frag672-837_insertion672	ATATATCCGCGTCTCCCACCAGCGGGAGACCGGGGTCTCTGAGCAAAATTGAGGAGGACGAAGTCCCCGATATTACTGATCCTTTCTTTCTGATAGAGACACTCTGTATCATCTGGTTTACATTCGAACTGACTGTTAGGTTCTTGGCTTGTCCCAACAAGCTCAACTTTTGTCGCGATGTAATGAACGTGATTGATATTATAGCAATTATACGGAGACGACCGAACGGC
shaker_Frag672-837_insertion675	ATATATCCGCGTCTCCCACCAAAAGCGGGAGACCGGGGTCTCTGAGCATTGAGGAGGACGAAGTCCCCGATATTACTGATCCTTTCTTTCTGATAGAGACACTCTGTATCATCTGGTTTACATTCGAACTGACTGTTAGGTTCTTGGCTTGTCCCAACAAGCTCAACTTTTGTCGCGATGTAATGAACGTGATTGATATTATAGCAATTATACGGAGACGACCGAACGGC
shaker_Frag672-837_insertion678	ATATATCCGCGTCTCCCACCAAAATTAGCGGGAGACCGGGGTCTCTGAGCGAGGAGGACGAAGTCCCCGATATTACTGATCCTTTCTTTCTGATAGAGACACTCTGTATCATCTGGTTTACATTCGAACTGACTGTTAGGTTCTTGGCTTGTCCCAACAAGCTCAACTTTTGTCGCGATGTAATGAACGTGATTGATATTATAGCAATTATACGGAGACGACCGAACGGC
shaker_Frag672-837_insertion681	ATATATCCGCGTCTCCCACCAAAATTGAGAGCGGGAGACCGGGGTCTCTGAGCGAGGACGAAGTCCCCGATATTACTGATCCTTTCTTTCTGATAGAGACACTCTGTATCATCTGGTTTACATTCGAACTGACTGTTAGGTTCTTGGCTTGTCCCAACAAGCTCAACTTTTGTCGCGATGTAATGAACGTGATTGATATTATAGCAATTATACGGAGACGACCGAACGGC
shaker_Frag672-837_insertion684	ATATATCCGCGTCTCCCACCAAAATTGAGGAGAGCGGGAGACCGGGGTCTCTGAGCGACGAAGTCCCCGATATTACTGATCCTTTCTTTCTGATAGAGACACTCTGTATCATCTGGTTTACATTCGAACTGACTGTTAGGTTCTTGGCTTGTCCCAACAAGCTCAACTTTTGTCGCGATGTAATGAACGTGATTGATATTATAGCAATTATACGGAGACGACCGAACGGC
shaker_Frag672-837_insertion687	ATATATCCGCGTCTCCCACCAAAATTGAGGAGGACAGCGGGAGACCGGGGTCTCTGAGCGAAGTCCCCGATATTACTGATCCTTTCTTTCTGATAGAGACACTCTGTATCATCTGGTTTACATTCGAACTGACTGTTAGGTTCTTGGCTTGTCCCAACAAGCTCAACTTTTGTCGCGATGTAATGAACGTGATTGATATTATAGCAATTATACGGAGACGACCGAACGGC
shaker_Frag672-837_insertion690	ATATATCCGCGTCTCCCACCAAAATTGAGGAGGACGAAAGCGGGAGACCGGGGTCTCTGAGCGTCCCCGATATTACTGATCCTTTCTTTCTGATAGAGACACTCTGTATCATCTGGTTTACATTCGAACTGACTGTTAGGTTCTTGGCTTGTCCCAACAAGCTCAACTTTTGTCGCGATGTAATGAACGTGATTGATATTATAGCAATTATACGGAGACGACCGAACGGC
shaker_Frag672-837_insertion693	ATATATCCGCGTCTCCCACCAAAATTGAGGAGGACGAAGTCAGCGGGAGACCGGGGTCTCTGAGCCCCGATATTACTGATCCTTTCTTTCTGATAGAGACACTCTGTATCATCTGGTTTACATTCGAACTGACTGTTAGGTTCTTGGCTTGTCCCAACAAGCTCAACTTTTGTCGCGATGTAATGAACGTGATTGATATTATAGCAATTATACGGAGACGACCGAACGGC
shaker_Frag672-837_insertion696	ATATATCCGCGTCTCCCACCAAAATTGAGGAGGACGAAGTCCCCAGCGGGAGACCGGGGTCTCTGAGCGATATTACTGATCCTTTCTTTCTGATAGAGACACTCTGTATCATCTGGTTTACATTCGAACTGACTGTTAGGTTCTTGGCTTGTCCCAACAAGCTCAACTTTTGTCGCGATGTAATGAACGTGATTGATATTATAGCAATTATACGGAGACGACCGAACGGC
shaker_Frag672-837_insertion699	ATATATCCGCGTCTCCCACCAAAATTGAGGAGGACGAAGTCCCCGATAGCGGGAGACCGGGGTCTCTGAGCATTACTGATCCTTTCTTTCTGATAGAGACACTCTGTATCATCTGGTTTACATTCGAACTGACTGTTAGGTTCTTGGCTTGTCCCAACAAGCTCAACTTTTGTCGCGATGTAATGAACGTGATTGATATTATAGCAATTATACGGAGACGACCGAACGGC
shaker_Frag672-837_insertion702	ATATATCCGCGTCTCCCACCAAAATTGAGGAGGACGAAGTCCCCGATATTAGCGGGAGACCGGGGTCTCTGAGCACTGATCCTTTCTTTCTGATAGAGACACTCTGTATCATCTGGTTTACATTCGAACTGACTGTTAGGTTCTTGGCTTGTCCCAACAAGCTCAACTTTTGTCGCGATGTAATGAACGTGATTGATATTATAGCAATTATACGGAGACGACCGAACGGC
shaker_Frag672-837_insertion705	ATATATCCGCGTCTCCCACCAAAATTGAGGAGGACGAAGTCCCCGATATTACTAGCGGGAGACCGGGGTCTCTGAGCGATCCTTTCTTTCTGATAGAGACACTCTGTATCATCTGGTTTACATTCGAACTGACTGTTAGGTTCTTGGCTTGTCCCAACAAGCTCAACTTTTGTCGCGATGTAATGAACGTGATTGATATTATAGCAATTATACGGAGACGACCGAACGGC
shaker_Frag672-837_insertion708	ATATATCCGCGTCTCCCACCAAAATTGAGGAGGACGAAGTCCCCGATATTACTGATAGCGGGAGACCGGGGTCTCTGAGCCCTTTCTTTCTGATAGAGACACTCTGTATCATCTGGTTTACATTCGAACTGACTGTTAGGTTCTTGGCTTGTCCCAACAAGCTCAACTTTTGTCGCGATGTAATGAACGTGATTGATATTATAGCAATTATACGGAGACGACCGAACGGC
shaker_Frag672-837_insertion711	ATATATCCGCGTCTCCCACCAAAATTGAGGAGGACGAAGTCCCCGATATTACTGATCCTAGCGGGAGACCGGGGTCTCTGAGCTTCTTTCTGATAGAGACACTCTGTATCATCTGGTTTACATTCGAACTGACTGTTAGGTTCTTGGCTTGTCCCAACAAGCTCAACTTTTGTCGCGATGTAATGAACGTGATTGATATTATAGCAATTATACGGAGACGACCGAACGGC
shaker_Frag672-837_insertion714	ATATATCCGCGTCTCCCACCAAAATTGAGGAGGACGAAGTCCCCGATATTACTGATCCTTTCAGCGGGAGACCGGGGTCTCTGAGCTTTCTGATAGAGACACTCTGTATCATCTGGTTTACATTCGAACTGACTGTTAGGTTCTTGGCTTGTCCCAACAAGCTCAACTTTTGTCGCGATGTAATGAACGTGATTGATATTATAGCAATTATACGGAGACGACCGAACGGC
shaker_Frag672-837_insertion717	ATATATCCGCGTCTCCCACCAAAATTGAGGAGGACGAAGTCCCCGATATTACTGATCCTTTCTTTAGCGGGAGACCGGGGTCTCTGAGCCTGATAGAGACACTCTGTATCATCTGGTTTACATTCGAACTGACTGTTAGGTTCTTGGCTTGTCCCAACAAGCTCAACTTTTGTCGCGATGTAATGAACGTGATTGATATTATAGCAATTATACGGAGACGACCGAACGGC
shaker_Frag672-837_insertion720	ATATATCCGCGTCTCCCACCAAAATTGAGGAGGACGAAGTCCCCGATATTACTGATCCTTTCTTTCTGAGCGGGAGACCGGGGTCTCTGAGCATAGAGACACTCTGTATCATCTGGTTTACATTCGAACTGACTGTTAGGTTCTTGGCTTGTCCCAACAAGCTCAACTTTTGTCGCGATGTAATGAACGTGATTGATATTATAGCAATTATACGGAGACGACCGAACGGC
shaker_Frag672-837_insertion723	ATATATCCGCGTCTCCCACCAAAATTGAGGAGGACGAAGTCCCCGATATTACTGATCCTTTCTTTCTGATAAGCGGGAGACCGGGGTCTCTGAGCGAGACACTCTGTATCATCTGGTTTACATTCGAACTGACTGTTAGGTTCTTGGCTTGTCCCAACAAGCTCAACTTTTGTCGCGATGTAATGAACGTGATTGATATTATAGCAATTATACGGAGACGACCGAACGGC
shaker_Frag672-837_insertion726	ATATATCCGCGTCTCCCACCAAAATTGAGGAGGACGAAGTCCCCGATATTACTGATCCTTTCTTTCTGATAGAGAGCGGGAGACCGGGGTCTCTGAGCACACTCTGTATCATCTGGTTTACATTCGAACTGACTGTTAGGTTCTTGGCTTGTCCCAACAAGCTCAACTTTTGTCGCGATGTAATGAACGTGATTGATATTATAGCAATTATACGGAGACGACCGAACGGC
shaker_Frag672-837_insertion729	ATATATCCGCGTCTCCCACCAAAATTGAGGAGGACGAAGTCCCCGATATTACTGATCCTTTCTTTCTGATAGAGACAAGCGGGAGACCGGGGTCTCTGAGCCTCTGTATCATCTGGTTTACATTCGAACTGACTGTTAGGTTCTTGGCTTGTCCCAACAAGCTCAACTTTTGTCGCGATGTAATGAACGTGATTGATATTATAGCAATTATACGGAGACGACCGAACGGC
shaker_Frag672-837_insertion732	ATATATCCGCGTCTCCCACCAAAATTGAGGAGGACGAAGTCCCCGATATTACTGATCCTTTCTTTCTGATAGAGACACTCAGCGGGAGACCGGGGTCTCTGAGCTGTATCATCTGGTTTACATTCGAACTGACTGTTAGGTTCTTGGCTTGTCCCAACAAGCTCAACTTTTGTCGCGATGTAATGAACGTGATTGATATTATAGCAATTATACGGAGACGACCGAACGGC
shaker_Frag672-837_insertion735	ATATATCCGCGTCTCCCACCAAAATTGAGGAGGACGAAGTCCCCGATATTACTGATCCTTTCTTTCTGATAGAGACACTCTGTAGCGGGAGACCGGGGTCTCTGAGCATCATCTGGTTTACATTCGAACTGACTGTTAGGTTCTTGGCTTGTCCCAACAAGCTCAACTTTTGTCGCGATGTAATGAACGTGATTGATATTATAGCAATTATACGGAGACGACCGAACGGC
shaker_Frag672-837_insertion738	ATATATCCGCGTCTCCCACCAAAATTGAGGAGGACGAAGTCCCCGATATTACTGATCCTTTCTTTCTGATAGAGACACTCTGTATCAGCGGGAGACCGGGGTCTCTGAGCATCTGGTTTACATTCGAACTGACTGTTAGGTTCTTGGCTTGTCCCAACAAGCTCAACTTTTGTCGCGATGTAATGAACGTGATTGATATTATAGCAATTATACGGAGACGACCGAACGGC
shaker_Frag672-837_insertion741	ATATATCCGCGTCTCCCACCAAAATTGAGGAGGACGAAGTCCCCGATATTACTGATCCTTTCTTTCTGATAGAGACACTCTGTATCATCAGCGGGAGACCGGGGTCTCTGAGCTGGTTTACATTCGAACTGACTGTTAGGTTCTTGGCTTGTCCCAACAAGCTCAACTTTTGTCGCGATGTAATGAACGTGATTGATATTATAGCAATTATACGGAGACGACCGAACGGC
shaker_Frag672-837_insertion744	ATATATCCGCGTCTCCCACCAAAATTGAGGAGGACGAAGTCCCCGATATTACTGATCCTTTCTTTCTGATAGAGACACTCTGTATCATCTGGAGCGGGAGACCGGGGTCTCTGAGCTTTACATTCGAACTGACTGTTAGGTTCTTGGCTTGTCCCAACAAGCTCAACTTTTGTCGCGATGTAATGAACGTGATTGATATTATAGCAATTATACGGAGACGACCGAACGGC
shaker_Frag672-837_insertion747	ATATATCCGCGTCTCCCACCAAAATTGAGGAGGACGAAGTCCCCGATATTACTGATCCTTTCTTTCTGATAGAGACACTCTGTATCATCTGGTTTAGCGGGAGACCGGGGTCTCTGAGCACATTCGAACTGACTGTTAGGTTCTTGGCTTGTCCCAACAAGCTCAACTTTTGTCGCGATGTAATGAACGTGATTGATATTATAGCAATTATACGGAGACGACCGAACGGC
shaker_Frag672-837_insertion750	ATATATCCGCGTCTCCCACCAAAATTGAGGAGGACGAAGTCCCCGATATTACTGATCCTTTCTTTCTGATAGAGACACTCTGTATCATCTGGTTTACAAGCGGGAGACCGGGGTCTCTGAGCTTCGAACTGACTGTTAGGTTCTTGGCTTGTCCCAACAAGCTCAACTTTTGTCGCGATGTAATGAACGTGATTGATATTATAGCAATTATACGGAGACGACCGAACGGC
shaker_Frag672-837_insertion753	ATATATCCGCGTCTCCCACCAAAATTGAGGAGGACGAAGTCCCCGATATTACTGATCCTTTCTTTCTGATAGAGACACTCTGTATCATCTGGTTTACATTCAGCGGGAGACCGGGGTCTCTGAGCGAACTGACTGTTAGGTTCTTGGCTTGTCCCAACAAGCTCAACTTTTGTCGCGATGTAATGAACGTGATTGATATTATAGCAATTATACGGAGACGACCGAACGGC
shaker_Frag672-837_insertion756	ATATATCCGCGTCTCCCACCAAAATTGAGGAGGACGAAGTCCCCGATATTACTGATCCTTTCTTTCTGATAGAGACACTCTGTATCATCTGGTTTACATTCGAAAGCGGGAGACCGGGGTCTCTGAGCCTGACTGTTAGGTTCTTGGCTTGTCCCAACAAGCTCAACTTTTGTCGCGATGTAATGAACGTGATTGATATTATAGCAATTATACGGAGACGACCGAACGGC
shaker_Frag672-837_insertion759	ATATATCCGCGTCTCCCACCAAAATTGAGGAGGACGAAGTCCCCGATATTACTGATCCTTTCTTTCTGATAGAGACACTCTGTATCATCTGGTTTACATTCGAACTGAGCGGGAGACCGGGGTCTCTGAGCACTGTTAGGTTCTTGGCTTGTCCCAACAAGCTCAACTTTTGTCGCGATGTAATGAACGTGATTGATATTATAGCAATTATACGGAGACGACCGAACGGC
shaker_Frag672-837_insertion762	ATATATCCGCGTCTCCCACCAAAATTGAGGAGGACGAAGTCCCCGATATTACTGATCCTTTCTTTCTGATAGAGACACTCTGTATCATCTGGTTTACATTCGAACTGACTAGCGGGAGACCGGGGTCTCTGAGCGTTAGGTTCTTGGCTTGTCCCAACAAGCTCAACTTTTGTCGCGATGTAATGAACGTGATTGATATTATAGCAATTATACGGAGACGACCGAACGGC
shaker_Frag672-837_insertion765	ATATATCCGCGTCTCCCACCAAAATTGAGGAGGACGAAGTCCCCGATATTACTGATCCTTTCTTTCTGATAGAGACACTCTGTATCATCTGGTTTACATTCGAACTGACTGTTAGCGGGAGACCGGGGTCTCTGAGCAGGTTCTTGGCTTGTCCCAACAAGCTCAACTTTTGTCGCGATGTAATGAACGTGATTGATATTATAGCAATTATACGGAGACGACCGAACGGC
shaker_Frag672-837_insertion768	ATATATCCGCGTCTCCCACCAAAATTGAGGAGGACGAAGTCCCCGATATTACTGATCCTTTCTTTCTGATAGAGACACTCTGTATCATCTGGTTTACATTCGAACTGACTGTTAGGAGCGGGAGACCGGGGTCTCTGAGCTTCTTGGCTTGTCCCAACAAGCTCAACTTTTGTCGCGATGTAATGAACGTGATTGATATTATAGCAATTATACGGAGACGACCGAACGGC
shaker_Frag672-837_insertion771	ATATATCCGCGTCTCCCACCAAAATTGAGGAGGACGAAGTCCCCGATATTACTGATCCTTTCTTTCTGATAGAGACACTCTGTATCATCTGGTTTACATTCGAACTGACTGTTAGGTTCAGCGGGAGACCGGGGTCTCTGAGCTTGGCTTGTCCCAACAAGCTCAACTTTTGTCGCGATGTAATGAACGTGATTGATATTATAGCAATTATACGGAGACGACCGAACGGC
shaker_Frag672-837_insertion774	ATATATCCGCGTCTCCCACCAAAATTGAGGAGGACGAAGTCCCCGATATTACTGATCCTTTCTTTCTGATAGAGACACTCTGTATCATCTGGTTTACATTCGAACTGACTGTTAGGTTCTTGAGCGGGAGACCGGGGTCTCTGAGCGCTTGTCCCAACAAGCTCAACTTTTGTCGCGATGTAATGAACGTGATTGATATTATAGCAATTATACGGAGACGACCGAACGGC
shaker_Frag672-837_insertion777	ATATATCCGCGTCTCCCACCAAAATTGAGGAGGACGAAGTCCCCGATATTACTGATCCTTTCTTTCTGATAGAGACACTCTGTATCATCTGGTTTACATTCGAACTGACTGTTAGGTTCTTGGCTAGCGGGAGACCGGGGTCTCTGAGCTGTCCCAACAAGCTCAACTTTTGTCGCGATGTAATGAACGTGATTGATATTATAGCAATTATACGGAGACGACCGAACGGC
shaker_Frag672-837_insertion780	ATATATCCGCGTCTCCCACCAAAATTGAGGAGGACGAAGTCCCCGATATTACTGATCCTTTCTTTCTGATAGAGACACTCTGTATCATCTGGTTTACATTCGAACTGACTGTTAGGTTCTTGGCTTGTAGCGGGAGACCGGGGTCTCTGAGCCCCAACAAGCTCAACTTTTGTCGCGATGTAATGAACGTGATTGATATTATAGCAATTATACGGAGACGACCGAACGGC
shaker_Frag672-837_insertion783	ATATATCCGCGTCTCCCACCAAAATTGAGGAGGACGAAGTCCCCGATATTACTGATCCTTTCTTTCTGATAGAGACACTCTGTATCATCTGGTTTACATTCGAACTGACTGTTAGGTTCTTGGCTTGTCCCAGCGGGAGACCGGGGTCTCTGAGCAACAAGCTCAACTTTTGTCGCGATGTAATGAACGTGATTGATATTATAGCAATTATACGGAGACGACCGAACGGC
shaker_Frag672-837_insertion786	ATATATCCGCGTCTCCCACCAAAATTGAGGAGGACGAAGTCCCCGATATTACTGATCCTTTCTTTCTGATAGAGACACTCTGTATCATCTGGTTTACATTCGAACTGACTGTTAGGTTCTTGGCTTGTCCCAACAGCGGGAGACCGGGGTCTCTGAGCAAGCTCAACTTTTGTCGCGATGTAATGAACGTGATTGATATTATAGCAATTATACGGAGACGACCGAACGGC
shaker_Frag672-837_insertion789	ATATATCCGCGTCTCCCACCAAAATTGAGGAGGACGAAGTCCCCGATATTACTGATCCTTTCTTTCTGATAGAGACACTCTGTATCATCTGGTTTACATTCGAACTGACTGTTAGGTTCTTGGCTTGTCCCAACAAGAGCGGGAGACCGGGGTCTCTGAGCCTCAACTTTTGTCGCGATGTAATGAACGTGATTGATATTATAGCAATTATACGGAGACGACCGAACGGC
shaker_Frag672-837_insertion792	ATATATCCGCGTCTCCCACCAAAATTGAGGAGGACGAAGTCCCCGATATTACTGATCCTTTCTTTCTGATAGAGACACTCTGTATCATCTGGTTTACATTCGAACTGACTGTTAGGTTCTTGGCTTGTCCCAACAAGCTCAGCGGGAGACCGGGGTCTCTGAGCAACTTTTGTCGCGATGTAATGAACGTGATTGATATTATAGCAATTATACGGAGACGACCGAACGGC
shaker_Frag672-837_insertion795	ATATATCCGCGTCTCCCACCAAAATTGAGGAGGACGAAGTCCCCGATATTACTGATCCTTTCTTTCTGATAGAGACACTCTGTATCATCTGGTTTACATTCGAACTGACTGTTAGGTTCTTGGCTTGTCCCAACAAGCTCAACAGCGGGAGACCGGGGTCTCTGAGCTTTTGTCGCGATGTAATGAACGTGATTGATATTATAGCAATTATACGGAGACGACCGAACGGC
shaker_Frag672-837_insertion798	ATATATCCGCGTCTCCCACCAAAATTGAGGAGGACGAAGTCCCCGATATTACTGATCCTTTCTTTCTGATAGAGACACTCTGTATCATCTGGTTTACATTCGAACTGACTGTTAGGTTCTTGGCTTGTCCCAACAAGCTCAACTTTAGCGGGAGACCGGGGTCTCTGAGCTGTCGCGATGTAATGAACGTGATTGATATTATAGCAATTATACGGAGACGACCGAACGGC
shaker_Frag672-837_insertion801	ATATATCCGCGTCTCCCACCAAAATTGAGGAGGACGAAGTCCCCGATATTACTGATCCTTTCTTTCTGATAGAGACACTCTGTATCATCTGGTTTACATTCGAACTGACTGTTAGGTTCTTGGCTTGTCCCAACAAGCTCAACTTTTGTAGCGGGAGACCGGGGTCTCTGAGCCGCGATGTAATGAACGTGATTGATATTATAGCAATTATACGGAGACGACCGAACGGC
shaker_Frag672-837_insertion804	ATATATCCGCGTCTCCCACCAAAATTGAGGAGGACGAAGTCCCCGATATTACTGATCCTTTCTTTCTGATAGAGACACTCTGTATCATCTGGTTTACATTCGAACTGACTGTTAGGTTCTTGGCTTGTCCCAACAAGCTCAACTTTTGTCGCAGCGGGAGACCGGGGTCTCTGAGCGATGTAATGAACGTGATTGATATTATAGCAATTATACGGAGACGACCGAACGGC
shaker_Frag672-837_insertion807	ATATATCCGCGTCTCCCACCAAAATTGAGGAGGACGAAGTCCCCGATATTACTGATCCTTTCTTTCTGATAGAGACACTCTGTATCATCTGGTTTACATTCGAACTGACTGTTAGGTTCTTGGCTTGTCCCAACAAGCTCAACTTTTGTCGCGATAGCGGGAGACCGGGGTCTCTGAGCGTAATGAACGTGATTGATATTATAGCAATTATACGGAGACGACCGAACGGC
shaker_Frag672-837_insertion810	ATATATCCGCGTCTCCCACCAAAATTGAGGAGGACGAAGTCCCCGATATTACTGATCCTTTCTTTCTGATAGAGACACTCTGTATCATCTGGTTTACATTCGAACTGACTGTTAGGTTCTTGGCTTGTCCCAACAAGCTCAACTTTTGTCGCGATGTAAGCGGGAGACCGGGGTCTCTGAGCATGAACGTGATTGATATTATAGCAATTATACGGAGACGACCGAACGGC
shaker_Frag672-837_insertion813	ATATATCCGCGTCTCCCACCAAAATTGAGGAGGACGAAGTCCCCGATATTACTGATCCTTTCTTTCTGATAGAGACACTCTGTATCATCTGGTTTACATTCGAACTGACTGTTAGGTTCTTGGCTTGTCCCAACAAGCTCAACTTTTGTCGCGATGTAATGAGCGGGAGACCGGGGTCTCTGAGCAACGTGATTGATATTATAGCAATTATACGGAGACGACCGAACGGC
shaker_Frag672-837_insertion816	ATATATCCGCGTCTCCCACCAAAATTGAGGAGGACGAAGTCCCCGATATTACTGATCCTTTCTTTCTGATAGAGACACTCTGTATCATCTGGTTTACATTCGAACTGACTGTTAGGTTCTTGGCTTGTCCCAACAAGCTCAACTTTTGTCGCGATGTAATGAACAGCGGGAGACCGGGGTCTCTGAGCGTGATTGATATTATAGCAATTATACGGAGACGACCGAACGGC
shaker_Frag672-837_insertion819	ATATATCCGCGTCTCCCACCAAAATTGAGGAGGACGAAGTCCCCGATATTACTGATCCTTTCTTTCTGATAGAGACACTCTGTATCATCTGGTTTACATTCGAACTGACTGTTAGGTTCTTGGCTTGTCCCAACAAGCTCAACTTTTGTCGCGATGTAATGAACGTGAGCGGGAGACCGGGGTCTCTGAGCATTGATATTATAGCAATTATACGGAGACGACCGAACGGC
shaker_Frag672-837_insertion822	ATATATCCGCGTCTCCCACCAAAATTGAGGAGGACGAAGTCCCCGATATTACTGATCCTTTCTTTCTGATAGAGACACTCTGTATCATCTGGTTTACATTCGAACTGACTGTTAGGTTCTTGGCTTGTCCCAACAAGCTCAACTTTTGTCGCGATGTAATGAACGTGATTAGCGGGAGACCGGGGTCTCTGAGCGATATTATAGCAATTATACGGAGACGACCGAACGGC
shaker_Frag672-837_insertion825	ATATATCCGCGTCTCCCACCAAAATTGAGGAGGACGAAGTCCCCGATATTACTGATCCTTTCTTTCTGATAGAGACACTCTGTATCATCTGGTTTACATTCGAACTGACTGTTAGGTTCTTGGCTTGTCCCAACAAGCTCAACTTTTGTCGCGATGTAATGAACGTGATTGATAGCGGGAGACCGGGGTCTCTGAGCATTATAGCAATTATACGGAGACGACCGAACGGC
shaker_Frag672-837_insertion828	ATATATCCGCGTCTCCCACCAAAATTGAGGAGGACGAAGTCCCCGATATTACTGATCCTTTCTTTCTGATAGAGACACTCTGTATCATCTGGTTTACATTCGAACTGACTGTTAGGTTCTTGGCTTGTCCCAACAAGCTCAACTTTTGTCGCGATGTAATGAACGTGATTGATATTAGCGGGAGACCGGGGTCTCTGAGCATAGCAATTATACGGAGACGACCGAACGGC
shaker_Frag672-837_insertion831	ATATATCCGCGTCTCCCACCAAAATTGAGGAGGACGAAGTCCCCGATATTACTGATCCTTTCTTTCTGATAGAGACACTCTGTATCATCTGGTTTACATTCGAACTGACTGTTAGGTTCTTGGCTTGTCCCAACAAGCTCAACTTTTGTCGCGATGTAATGAACGTGATTGATATTATAAGCGGGAGACCGGGGTCTCTGAGCGCAATTATACGGAGACGACCGAACGGC
shaker_Frag672-837_insertion834	ATATATCCGCGTCTCCCACCAAAATTGAGGAGGACGAAGTCCCCGATATTACTGATCCTTTCTTTCTGATAGAGACACTCTGTATCATCTGGTTTACATTCGAACTGACTGTTAGGTTCTTGGCTTGTCCCAACAAGCTCAACTTTTGTCGCGATGTAATGAACGTGATTGATATTATAGCAAGCGGGAGACCGGGGTCTCTGAGCATTATACGGAGACGACCGAACGGC
shaker_Frag672-837_insertion837	ATATATCCGCGTCTCCCACCAAAATTGAGGAGGACGAAGTCCCCGATATTACTGATCCTTTCTTTCTGATAGAGACACTCTGTATCATCTGGTTTACATTCGAACTGACTGTTAGGTTCTTGGCTTGTCCCAACAAGCTCAACTTTTGTCGCGATGTAATGAACGTGATTGATATTATAGCAATTAGCGGGAGACCGGGGTCTCTGAGCATACGGAGACGACCGAACGGC
shaker_Frag840-1008_insertion840	TGTCGCTCCGTCTCCTATAAGCGGGAGACCGGGGTCTCTGAGCCCTTATTTTATTACCTTGGCTACAGTAGTCGCAGAAGAGGAAGATACTCTGAACCTCCCAAAGGCCCCCGTTTCCCCTCAAGATAAAAGCTCTAACCAGGCAATGTCCCTGGCAATCCTCAGGGTGATAAGGCTGGTCCGGGTGTTCAGGATTTTTAAGTTGTCTCGACACTGGAGACGAGCAGCTA
shaker_Frag840-1008_insertion843	TGTCGCTCCGTCTCCTATACCTAGCGGGAGACCGGGGTCTCTGAGCTTATTTTATTACCTTGGCTACAGTAGTCGCAGAAGAGGAAGATACTCTGAACCTCCCAAAGGCCCCCGTTTCCCCTCAAGATAAAAGCTCTAACCAGGCAATGTCCCTGGCAATCCTCAGGGTGATAAGGCTGGTCCGGGTGTTCAGGATTTTTAAGTTGTCTCGACACTGGAGACGAGCAGCTA
shaker_Frag840-1008_insertion846	TGTCGCTCCGTCTCCTATACCTTATAGCGGGAGACCGGGGTCTCTGAGCTTTATTACCTTGGCTACAGTAGTCGCAGAAGAGGAAGATACTCTGAACCTCCCAAAGGCCCCCGTTTCCCCTCAAGATAAAAGCTCTAACCAGGCAATGTCCCTGGCAATCCTCAGGGTGATAAGGCTGGTCCGGGTGTTCAGGATTTTTAAGTTGTCTCGACACTGGAGACGAGCAGCTA
shaker_Frag840-1008_insertion849	TGTCGCTCCGTCTCCTATACCTTATTTTAGCGGGAGACCGGGGTCTCTGAGCATTACCTTGGCTACAGTAGTCGCAGAAGAGGAAGATACTCTGAACCTCCCAAAGGCCCCCGTTTCCCCTCAAGATAAAAGCTCTAACCAGGCAATGTCCCTGGCAATCCTCAGGGTGATAAGGCTGGTCCGGGTGTTCAGGATTTTTAAGTTGTCTCGACACTGGAGACGAGCAGCTA
shaker_Frag840-1008_insertion852	TGTCGCTCCGTCTCCTATACCTTATTTTATTAGCGGGAGACCGGGGTCTCTGAGCACCTTGGCTACAGTAGTCGCAGAAGAGGAAGATACTCTGAACCTCCCAAAGGCCCCCGTTTCCCCTCAAGATAAAAGCTCTAACCAGGCAATGTCCCTGGCAATCCTCAGGGTGATAAGGCTGGTCCGGGTGTTCAGGATTTTTAAGTTGTCTCGACACTGGAGACGAGCAGCTA
shaker_Frag840-1008_insertion855	TGTCGCTCCGTCTCCTATACCTTATTTTATTACCAGCGGGAGACCGGGGTCTCTGAGCTTGGCTACAGTAGTCGCAGAAGAGGAAGATACTCTGAACCTCCCAAAGGCCCCCGTTTCCCCTCAAGATAAAAGCTCTAACCAGGCAATGTCCCTGGCAATCCTCAGGGTGATAAGGCTGGTCCGGGTGTTCAGGATTTTTAAGTTGTCTCGACACTGGAGACGAGCAGCTA
shaker_Frag840-1008_insertion858	TGTCGCTCCGTCTCCTATACCTTATTTTATTACCTTGAGCGGGAGACCGGGGTCTCTGAGCGCTACAGTAGTCGCAGAAGAGGAAGATACTCTGAACCTCCCAAAGGCCCCCGTTTCCCCTCAAGATAAAAGCTCTAACCAGGCAATGTCCCTGGCAATCCTCAGGGTGATAAGGCTGGTCCGGGTGTTCAGGATTTTTAAGTTGTCTCGACACTGGAGACGAGCAGCTA
shaker_Frag840-1008_insertion861	TGTCGCTCCGTCTCCTATACCTTATTTTATTACCTTGGCTAGCGGGAGACCGGGGTCTCTGAGCACAGTAGTCGCAGAAGAGGAAGATACTCTGAACCTCCCAAAGGCCCCCGTTTCCCCTCAAGATAAAAGCTCTAACCAGGCAATGTCCCTGGCAATCCTCAGGGTGATAAGGCTGGTCCGGGTGTTCAGGATTTTTAAGTTGTCTCGACACTGGAGACGAGCAGCTA
shaker_Frag840-1008_insertion864	TGTCGCTCCGTCTCCTATACCTTATTTTATTACCTTGGCTACAAGCGGGAGACCGGGGTCTCTGAGCGTAGTCGCAGAAGAGGAAGATACTCTGAACCTCCCAAAGGCCCCCGTTTCCCCTCAAGATAAAAGCTCTAACCAGGCAATGTCCCTGGCAATCCTCAGGGTGATAAGGCTGGTCCGGGTGTTCAGGATTTTTAAGTTGTCTCGACACTGGAGACGAGCAGCTA
shaker_Frag840-1008_insertion867	TGTCGCTCCGTCTCCTATACCTTATTTTATTACCTTGGCTACAGTAAGCGGGAGACCGGGGTCTCTGAGCGTCGCAGAAGAGGAAGATACTCTGAACCTCCCAAAGGCCCCCGTTTCCCCTCAAGATAAAAGCTCTAACCAGGCAATGTCCCTGGCAATCCTCAGGGTGATAAGGCTGGTCCGGGTGTTCAGGATTTTTAAGTTGTCTCGACACTGGAGACGAGCAGCTA
shaker_Frag840-1008_insertion870	TGTCGCTCCGTCTCCTATACCTTATTTTATTACCTTGGCTACAGTAGTCAGCGGGAGACCGGGGTCTCTGAGCGCAGAAGAGGAAGATACTCTGAACCTCCCAAAGGCCCCCGTTTCCCCTCAAGATAAAAGCTCTAACCAGGCAATGTCCCTGGCAATCCTCAGGGTGATAAGGCTGGTCCGGGTGTTCAGGATTTTTAAGTTGTCTCGACACTGGAGACGAGCAGCTA
shaker_Frag840-1008_insertion873	TGTCGCTCCGTCTCCTATACCTTATTTTATTACCTTGGCTACAGTAGTCGCAAGCGGGAGACCGGGGTCTCTGAGCGAAGAGGAAGATACTCTGAACCTCCCAAAGGCCCCCGTTTCCCCTCAAGATAAAAGCTCTAACCAGGCAATGTCCCTGGCAATCCTCAGGGTGATAAGGCTGGTCCGGGTGTTCAGGATTTTTAAGTTGTCTCGACACTGGAGACGAGCAGCTA
shaker_Frag840-1008_insertion876	TGTCGCTCCGTCTCCTATACCTTATTTTATTACCTTGGCTACAGTAGTCGCAGAAAGCGGGAGACCGGGGTCTCTGAGCGAGGAAGATACTCTGAACCTCCCAAAGGCCCCCGTTTCCCCTCAAGATAAAAGCTCTAACCAGGCAATGTCCCTGGCAATCCTCAGGGTGATAAGGCTGGTCCGGGTGTTCAGGATTTTTAAGTTGTCTCGACACTGGAGACGAGCAGCTA
shaker_Frag840-1008_insertion879	TGTCGCTCCGTCTCCTATACCTTATTTTATTACCTTGGCTACAGTAGTCGCAGAAGAGAGCGGGAGACCGGGGTCTCTGAGCGAAGATACTCTGAACCTCCCAAAGGCCCCCGTTTCCCCTCAAGATAAAAGCTCTAACCAGGCAATGTCCCTGGCAATCCTCAGGGTGATAAGGCTGGTCCGGGTGTTCAGGATTTTTAAGTTGTCTCGACACTGGAGACGAGCAGCTA
shaker_Frag840-1008_insertion882	TGTCGCTCCGTCTCCTATACCTTATTTTATTACCTTGGCTACAGTAGTCGCAGAAGAGGAAAGCGGGAGACCGGGGTCTCTGAGCGATACTCTGAACCTCCCAAAGGCCCCCGTTTCCCCTCAAGATAAAAGCTCTAACCAGGCAATGTCCCTGGCAATCCTCAGGGTGATAAGGCTGGTCCGGGTGTTCAGGATTTTTAAGTTGTCTCGACACTGGAGACGAGCAGCTA
shaker_Frag840-1008_insertion885	TGTCGCTCCGTCTCCTATACCTTATTTTATTACCTTGGCTACAGTAGTCGCAGAAGAGGAAGATAGCGGGAGACCGGGGTCTCTGAGCACTCTGAACCTCCCAAAGGCCCCCGTTTCCCCTCAAGATAAAAGCTCTAACCAGGCAATGTCCCTGGCAATCCTCAGGGTGATAAGGCTGGTCCGGGTGTTCAGGATTTTTAAGTTGTCTCGACACTGGAGACGAGCAGCTA

shaker_Frag840-1008_insertion888	TGTCGCTCCGTCTCCTATACCTTATTTTATTACCTTGGCTACAGTAGTCGCAGAAGAGGAAGATACTAGCGGGAGACCGGGGTCTCTGAGCCTGAACCTCCCAAAGGCCCCCGTTTCCCCTCAAGATAAAAGCTCTAACCAGGCAATGTCCCTGGCAATCCTCAGGGTGATAAGGCTGGTCCGGGTGTTCAGGATTTTTAAGTTGTCTCGACACTGGAGACGAGCAGCTA
shaker_Frag840-1008_insertion891	TGTCGCTCCGTCTCCTATACCTTATTTTATTACCTTGGCTACAGTAGTCGCAGAAGAGGAAGATACTCTGAGCGGGAGACCGGGGTCTCTGAGCAACCTCCCAAAGGCCCCCGTTTCCCCTCAAGATAAAAGCTCTAACCAGGCAATGTCCCTGGCAATCCTCAGGGTGATAAGGCTGGTCCGGGTGTTCAGGATTTTTAAGTTGTCTCGACACTGGAGACGAGCAGCTA
shaker_Frag840-1008_insertion894	TGTCGCTCCGTCTCCTATACCTTATTTTATTACCTTGGCTACAGTAGTCGCAGAAGAGGAAGATACTCTGAACAGCGGGAGACCGGGGTCTCTGAGCCTCCCAAAGGCCCCCGTTTCCCCTCAAGATAAAAGCTCTAACCAGGCAATGTCCCTGGCAATCCTCAGGGTGATAAGGCTGGTCCGGGTGTTCAGGATTTTTAAGTTGTCTCGACACTGGAGACGAGCAGCTA
shaker_Frag840-1008_insertion897	TGTCGCTCCGTCTCCTATACCTTATTTTATTACCTTGGCTACAGTAGTCGCAGAAGAGGAAGATACTCTGAACCTCAGCGGGAGACCGGGGTCTCTGAGCCCAAAGGCCCCCGTTTCCCCTCAAGATAAAAGCTCTAACCAGGCAATGTCCCTGGCAATCCTCAGGGTGATAAGGCTGGTCCGGGTGTTCAGGATTTTTAAGTTGTCTCGACACTGGAGACGAGCAGCTA
shaker_Frag840-1008_insertion900	TGTCGCTCCGTCTCCTATACCTTATTTTATTACCTTGGCTACAGTAGTCGCAGAAGAGGAAGATACTCTGAACCTCCCAAGCGGGAGACCGGGGTCTCTGAGCAAGGCCCCCGTTTCCCCTCAAGATAAAAGCTCTAACCAGGCAATGTCCCTGGCAATCCTCAGGGTGATAAGGCTGGTCCGGGTGTTCAGGATTTTTAAGTTGTCTCGACACTGGAGACGAGCAGCTA
shaker_Frag840-1008_insertion903	TGTCGCTCCGTCTCCTATACCTTATTTTATTACCTTGGCTACAGTAGTCGCAGAAGAGGAAGATACTCTGAACCTCCCAAAGAGCGGGAGACCGGGGTCTCTGAGCGCCCCCGTTTCCCCTCAAGATAAAAGCTCTAACCAGGCAATGTCCCTGGCAATCCTCAGGGTGATAAGGCTGGTCCGGGTGTTCAGGATTTTTAAGTTGTCTCGACACTGGAGACGAGCAGCTA
shaker_Frag840-1008_insertion906	TGTCGCTCCGTCTCCTATACCTTATTTTATTACCTTGGCTACAGTAGTCGCAGAAGAGGAAGATACTCTGAACCTCCCAAAGGCCAGCGGGAGACCGGGGTCTCTGAGCCCCGTTTCCCCTCAAGATAAAAGCTCTAACCAGGCAATGTCCCTGGCAATCCTCAGGGTGATAAGGCTGGTCCGGGTGTTCAGGATTTTTAAGTTGTCTCGACACTGGAGACGAGCAGCTA
shaker_Frag840-1008_insertion909	TGTCGCTCCGTCTCCTATACCTTATTTTATTACCTTGGCTACAGTAGTCGCAGAAGAGGAAGATACTCTGAACCTCCCAAAGGCCCCCAGCGGGAGACCGGGGTCTCTGAGCGTTTCCCCTCAAGATAAAAGCTCTAACCAGGCAATGTCCCTGGCAATCCTCAGGGTGATAAGGCTGGTCCGGGTGTTCAGGATTTTTAAGTTGTCTCGACACTGGAGACGAGCAGCTA
shaker_Frag840-1008_insertion912	TGTCGCTCCGTCTCCTATACCTTATTTTATTACCTTGGCTACAGTAGTCGCAGAAGAGGAAGATACTCTGAACCTCCCAAAGGCCCCCGTTAGCGGGAGACCGGGGTCTCTGAGCTCCCCTCAAGATAAAAGCTCTAACCAGGCAATGTCCCTGGCAATCCTCAGGGTGATAAGGCTGGTCCGGGTGTTCAGGATTTTTAAGTTGTCTCGACACTGGAGACGAGCAGCTA
shaker_Frag840-1008_insertion915	TGTCGCTCCGTCTCCTATACCTTATTTTATTACCTTGGCTACAGTAGTCGCAGAAGAGGAAGATACTCTGAACCTCCCAAAGGCCCCCGTTTCCAGCGGGAGACCGGGGTCTCTGAGCCCTCAAGATAAAAGCTCTAACCAGGCAATGTCCCTGGCAATCCTCAGGGTGATAAGGCTGGTCCGGGTGTTCAGGATTTTTAAGTTGTCTCGACACTGGAGACGAGCAGCTA
shaker_Frag840-1008_insertion918	TGTCGCTCCGTCTCCTATACCTTATTTTATTACCTTGGCTACAGTAGTCGCAGAAGAGGAAGATACTCTGAACCTCCCAAAGGCCCCCGTTTCCCCTAGCGGGAGACCGGGGTCTCTGAGCCAAGATAAAAGCTCTAACCAGGCAATGTCCCTGGCAATCCTCAGGGTGATAAGGCTGGTCCGGGTGTTCAGGATTTTTAAGTTGTCTCGACACTGGAGACGAGCAGCTA
shaker_Frag840-1008_insertion921	TGTCGCTCCGTCTCCTATACCTTATTTTATTACCTTGGCTACAGTAGTCGCAGAAGAGGAAGATACTCTGAACCTCCCAAAGGCCCCCGTTTCCCCTCAAAGCGGGAGACCGGGGTCTCTGAGCGATAAAAGCTCTAACCAGGCAATGTCCCTGGCAATCCTCAGGGTGATAAGGCTGGTCCGGGTGTTCAGGATTTTTAAGTTGTCTCGACACTGGAGACGAGCAGCTA
shaker_Frag840-1008_insertion924	TGTCGCTCCGTCTCCTATACCTTATTTTATTACCTTGGCTACAGTAGTCGCAGAAGAGGAAGATACTCTGAACCTCCCAAAGGCCCCCGTTTCCCCTCAAGATAGCGGGAGACCGGGGTCTCTGAGCAAAAGCTCTAACCAGGCAATGTCCCTGGCAATCCTCAGGGTGATAAGGCTGGTCCGGGTGTTCAGGATTTTTAAGTTGTCTCGACACTGGAGACGAGCAGCTA
shaker_Frag840-1008_insertion927	TGTCGCTCCGTCTCCTATACCTTATTTTATTACCTTGGCTACAGTAGTCGCAGAAGAGGAAGATACTCTGAACCTCCCAAAGGCCCCCGTTTCCCCTCAAGATAAAAGCGGGAGACCGGGGTCTCTGAGCAGCTCTAACCAGGCAATGTCCCTGGCAATCCTCAGGGTGATAAGGCTGGTCCGGGTGTTCAGGATTTTTAAGTTGTCTCGACACTGGAGACGAGCAGCTA
shaker_Frag840-1008_insertion930	TGTCGCTCCGTCTCCTATACCTTATTTTATTACCTTGGCTACAGTAGTCGCAGAAGAGGAAGATACTCTGAACCTCCCAAAGGCCCCCGTTTCCCCTCAAGATAAAAGCAGCGGGAGACCGGGGTCTCTGAGCTCTAACCAGGCAATGTCCCTGGCAATCCTCAGGGTGATAAGGCTGGTCCGGGTGTTCAGGATTTTTAAGTTGTCTCGACACTGGAGACGAGCAGCTA
shaker_Frag840-1008_insertion933	TGTCGCTCCGTCTCCTATACCTTATTTTATTACCTTGGCTACAGTAGTCGCAGAAGAGGAAGATACTCTGAACCTCCCAAAGGCCCCCGTTTCCCCTCAAGATAAAAGCTCTAGCGGGAGACCGGGGTCTCTGAGCAACCAGGCAATGTCCCTGGCAATCCTCAGGGTGATAAGGCTGGTCCGGGTGTTCAGGATTTTTAAGTTGTCTCGACACTGGAGACGAGCAGCTA
shaker_Frag840-1008_insertion936	TGTCGCTCCGTCTCCTATACCTTATTTTATTACCTTGGCTACAGTAGTCGCAGAAGAGGAAGATACTCTGAACCTCCCAAAGGCCCCCGTTTCCCCTCAAGATAAAAGCTCTAACAGCGGGAGACCGGGGTCTCTGAGCCAGGCAATGTCCCTGGCAATCCTCAGGGTGATAAGGCTGGTCCGGGTGTTCAGGATTTTTAAGTTGTCTCGACACTGGAGACGAGCAGCTA
shaker_Frag840-1008_insertion939	TGTCGCTCCGTCTCCTATACCTTATTTTATTACCTTGGCTACAGTAGTCGCAGAAGAGGAAGATACTCTGAACCTCCCAAAGGCCCCCGTTTCCCCTCAAGATAAAAGCTCTAACCAGAGCGGGAGACCGGGGTCTCTGAGCGCAATGTCCCTGGCAATCCTCAGGGTGATAAGGCTGGTCCGGGTGTTCAGGATTTTTAAGTTGTCTCGACACTGGAGACGAGCAGCTA
shaker_Frag840-1008_insertion942	TGTCGCTCCGTCTCCTATACCTTATTTTATTACCTTGGCTACAGTAGTCGCAGAAGAGGAAGATACTCTGAACCTCCCAAAGGCCCCCGTTTCCCCTCAAGATAAAAGCTCTAACCAGGCAAGCGGGAGACCGGGGTCTCTGAGCATGTCCCTGGCAATCCTCAGGGTGATAAGGCTGGTCCGGGTGTTCAGGATTTTTAAGTTGTCTCGACACTGGAGACGAGCAGCTA
shaker_Frag840-1008_insertion945	TGTCGCTCCGTCTCCTATACCTTATTTTATTACCTTGGCTACAGTAGTCGCAGAAGAGGAAGATACTCTGAACCTCCCAAAGGCCCCCGTTTCCCCTCAAGATAAAAGCTCTAACCAGGCAATGAGCGGGAGACCGGGGTCTCTGAGCTCCCTGGCAATCCTCAGGGTGATAAGGCTGGTCCGGGTGTTCAGGATTTTTAAGTTGTCTCGACACTGGAGACGAGCAGCTA
shaker_Frag840-1008_insertion948	TGTCGCTCCGTCTCCTATACCTTATTTTATTACCTTGGCTACAGTAGTCGCAGAAGAGGAAGATACTCTGAACCTCCCAAAGGCCCCCGTTTCCCCTCAAGATAAAAGCTCTAACCAGGCAATGTCCAGCGGGAGACCGGGGTCTCTGAGCCTGGCAATCCTCAGGGTGATAAGGCTGGTCCGGGTGTTCAGGATTTTTAAGTTGTCTCGACACTGGAGACGAGCAGCTA
shaker_Frag840-1008_insertion951	TGTCGCTCCGTCTCCTATACCTTATTTTATTACCTTGGCTACAGTAGTCGCAGAAGAGGAAGATACTCTGAACCTCCCAAAGGCCCCCGTTTCCCCTCAAGATAAAAGCTCTAACCAGGCAATGTCCCTGAGCGGGAGACCGGGGTCTCTGAGCGCAATCCTCAGGGTGATAAGGCTGGTCCGGGTGTTCAGGATTTTTAAGTTGTCTCGACACTGGAGACGAGCAGCTA
shaker_Frag840-1008_insertion954	TGTCGCTCCGTCTCCTATACCTTATTTTATTACCTTGGCTACAGTAGTCGCAGAAGAGGAAGATACTCTGAACCTCCCAAAGGCCCCCGTTTCCCCTCAAGATAAAAGCTCTAACCAGGCAATGTCCCTGGCAAGCGGGAGACCGGGGTCTCTGAGCATCCTCAGGGTGATAAGGCTGGTCCGGGTGTTCAGGATTTTTAAGTTGTCTCGACACTGGAGACGAGCAGCTA
shaker_Frag840-1008_insertion957	TGTCGCTCCGTCTCCTATACCTTATTTTATTACCTTGGCTACAGTAGTCGCAGAAGAGGAAGATACTCTGAACCTCCCAAAGGCCCCCGTTTCCCCTCAAGATAAAAGCTCTAACCAGGCAATGTCCCTGGCAATCAGCGGGAGACCGGGGTCTCTGAGCCTCAGGGTGATAAGGCTGGTCCGGGTGTTCAGGATTTTTAAGTTGTCTCGACACTGGAGACGAGCAGCTA
shaker_Frag840-1008_insertion960	TGTCGCTCCGTCTCCTATACCTTATTTTATTACCTTGGCTACAGTAGTCGCAGAAGAGGAAGATACTCTGAACCTCCCAAAGGCCCCCGTTTCCCCTCAAGATAAAAGCTCTAACCAGGCAATGTCCCTGGCAATCCTCAGCGGGAGACCGGGGTCTCTGAGCAGGGTGATAAGGCTGGTCCGGGTGTTCAGGATTTTTAAGTTGTCTCGACACTGGAGACGAGCAGCTA
shaker_Frag840-1008_insertion963	TGTCGCTCCGTCTCCTATACCTTATTTTATTACCTTGGCTACAGTAGTCGCAGAAGAGGAAGATACTCTGAACCTCCCAAAGGCCCCCGTTTCCCCTCAAGATAAAAGCTCTAACCAGGCAATGTCCCTGGCAATCCTCAGGAGCGGGAGACCGGGGTCTCTGAGCGTGATAAGGCTGGTCCGGGTGTTCAGGATTTTTAAGTTGTCTCGACACTGGAGACGAGCAGCTA
shaker_Frag840-1008_insertion966	TGTCGCTCCGTCTCCTATACCTTATTTTATTACCTTGGCTACAGTAGTCGCAGAAGAGGAAGATACTCTGAACCTCCCAAAGGCCCCCGTTTCCCCTCAAGATAAAAGCTCTAACCAGGCAATGTCCCTGGCAATCCTCAGGGTGAGCGGGAGACCGGGGTCTCTGAGCATAAGGCTGGTCCGGGTGTTCAGGATTTTTAAGTTGTCTCGACACTGGAGACGAGCAGCTA
shaker_Frag840-1008_insertion969	TGTCGCTCCGTCTCCTATACCTTATTTTATTACCTTGGCTACAGTAGTCGCAGAAGAGGAAGATACTCTGAACCTCCCAAAGGCCCCCGTTTCCCCTCAAGATAAAAGCTCTAACCAGGCAATGTCCCTGGCAATCCTCAGGGTGATAAGCGGGAGACCGGGGTCTCTGAGCAGGCTGGTCCGGGTGTTCAGGATTTTTAAGTTGTCTCGACACTGGAGACGAGCAGCTA
shaker_Frag840-1008_insertion972	TGTCGCTCCGTCTCCTATACCTTATTTTATTACCTTGGCTACAGTAGTCGCAGAAGAGGAAGATACTCTGAACCTCCCAAAGGCCCCCGTTTCCCCTCAAGATAAAAGCTCTAACCAGGCAATGTCCCTGGCAATCCTCAGGGTGATAAGGAGCGGGAGACCGGGGTCTCTGAGCCTGGTCCGGGTGTTCAGGATTTTTAAGTTGTCTCGACACTGGAGACGAGCAGCTA
shaker_Frag840-1008_insertion975	TGTCGCTCCGTCTCCTATACCTTATTTTATTACCTTGGCTACAGTAGTCGCAGAAGAGGAAGATACTCTGAACCTCCCAAAGGCCCCCGTTTCCCCTCAAGATAAAAGCTCTAACCAGGCAATGTCCCTGGCAATCCTCAGGGTGATAAGGCTGAGCGGGAGACCGGGGTCTCTGAGCGTCCGGGTGTTCAGGATTTTTAAGTTGTCTCGACACTGGAGACGAGCAGCTA
shaker_Frag840-1008_insertion978	TGTCGCTCCGTCTCCTATACCTTATTTTATTACCTTGGCTACAGTAGTCGCAGAAGAGGAAGATACTCTGAACCTCCCAAAGGCCCCCGTTTCCCCTCAAGATAAAAGCTCTAACCAGGCAATGTCCCTGGCAATCCTCAGGGTGATAAGGCTGGTCAGCGGGAGACCGGGGTCTCTGAGCCGGGTGTTCAGGATTTTTAAGTTGTCTCGACACTGGAGACGAGCAGCTA
shaker_Frag840-1008_insertion981	TGTCGCTCCGTCTCCTATACCTTATTTTATTACCTTGGCTACAGTAGTCGCAGAAGAGGAAGATACTCTGAACCTCCCAAAGGCCCCCGTTTCCCCTCAAGATAAAAGCTCTAACCAGGCAATGTCCCTGGCAATCCTCAGGGTGATAAGGCTGGTCCGGAGCGGGAGACCGGGGTCTCTGAGCGTGTTCAGGATTTTTAAGTTGTCTCGACACTGGAGACGAGCAGCTA
shaker_Frag840-1008_insertion984	TGTCGCTCCGTCTCCTATACCTTATTTTATTACCTTGGCTACAGTAGTCGCAGAAGAGGAAGATACTCTGAACCTCCCAAAGGCCCCCGTTTCCCCTCAAGATAAAAGCTCTAACCAGGCAATGTCCCTGGCAATCCTCAGGGTGATAAGGCTGGTCCGGGTGAGCGGGAGACCGGGGTCTCTGAGCTTCAGGATTTTTAAGTTGTCTCGACACTGGAGACGAGCAGCTA
shaker_Frag840-1008_insertion987	TGTCGCTCCGTCTCCTATACCTTATTTTATTACCTTGGCTACAGTAGTCGCAGAAGAGGAAGATACTCTGAACCTCCCAAAGGCCCCCGTTTCCCCTCAAGATAAAAGCTCTAACCAGGCAATGTCCCTGGCAATCCTCAGGGTGATAAGGCTGGTCCGGGTGTTCAGCGGGAGACCGGGGTCTCTGAGCAGGATTTTTAAGTTGTCTCGACACTGGAGACGAGCAGCTA
shaker_Frag840-1008_insertion990	TGTCGCTCCGTCTCCTATACCTTATTTTATTACCTTGGCTACAGTAGTCGCAGAAGAGGAAGATACTCTGAACCTCCCAAAGGCCCCCGTTTCCCCTCAAGATAAAAGCTCTAACCAGGCAATGTCCCTGGCAATCCTCAGGGTGATAAGGCTGGTCCGGGTGTTCAGGAGCGGGAGACCGGGGTCTCTGAGCATTTTTAAGTTGTCTCGACACTGGAGACGAGCAGCTA
shaker_Frag840-1008_insertion993	TGTCGCTCCGTCTCCTATACCTTATTTTATTACCTTGGCTACAGTAGTCGCAGAAGAGGAAGATACTCTGAACCTCCCAAAGGCCCCCGTTTCCCCTCAAGATAAAAGCTCTAACCAGGCAATGTCCCTGGCAATCCTCAGGGTGATAAGGCTGGTCCGGGTGTTCAGGATTAGCGGGAGACCGGGGTCTCTGAGCTTTAAGTTGTCTCGACACTGGAGACGAGCAGCTA
shaker_Frag840-1008_insertion996	TGTCGCTCCGTCTCCTATACCTTATTTTATTACCTTGGCTACAGTAGTCGCAGAAGAGGAAGATACTCTGAACCTCCCAAAGGCCCCCGTTTCCCCTCAAGATAAAAGCTCTAACCAGGCAATGTCCCTGGCAATCCTCAGGGTGATAAGGCTGGTCCGGGTGTTCAGGATTTTTAGCGGGAGACCGGGGTCTCTGAGCAAGTTGTCTCGACACTGGAGACGAGCAGCTA
shaker_Frag840-1008_insertion999	TGTCGCTCCGTCTCCTATACCTTATTTTATTACCTTGGCTACAGTAGTCGCAGAAGAGGAAGATACTCTGAACCTCCCAAAGGCCCCCGTTTCCCCTCAAGATAAAAGCTCTAACCAGGCAATGTCCCTGGCAATCCTCAGGGTGATAAGGCTGGTCCGGGTGTTCAGGATTTTTAAGAGCGGGAGACCGGGGTCTCTGAGCTTGTCTCGACACTGGAGACGAGCAGCTA
shaker_Frag840-1008_insertion1002	TGTCGCTCCGTCTCCTATACCTTATTTTATTACCTTGGCTACAGTAGTCGCAGAAGAGGAAGATACTCTGAACCTCCCAAAGGCCCCCGTTTCCCCTCAAGATAAAAGCTCTAACCAGGCAATGTCCCTGGCAATCCTCAGGGTGATAAGGCTGGTCCGGGTGTTCAGGATTTTTAAGTTGAGCGGGAGACCGGGGTCTCTGAGCTCTCGACACTGGAGACGAGCAGCTA
shaker_Frag840-1008_insertion1005	TGTCGCTCCGTCTCCTATACCTTATTTTATTACCTTGGCTACAGTAGTCGCAGAAGAGGAAGATACTCTGAACCTCCCAAAGGCCCCCGTTTCCCCTCAAGATAAAAGCTCTAACCAGGCAATGTCCCTGGCAATCCTCAGGGTGATAAGGCTGGTCCGGGTGTTCAGGATTTTTAAGTTGTCTAGCGGGAGACCGGGGTCTCTGAGCCGACACTGGAGACGAGCAGCTA
shaker_Frag840-1008_insertion1008	TGTCGCTCCGTCTCCTATACCTTATTTTATTACCTTGGCTACAGTAGTCGCAGAAGAGGAAGATACTCTGAACCTCCCAAAGGCCCCCGTTTCCCCTCAAGATAAAAGCTCTAACCAGGCAATGTCCCTGGCAATCCTCAGGGTGATAAGGCTGGTCCGGGTGTTCAGGATTTTTAAGTTGTCTCGAAGCGGGAGACCGGGGTCTCTGAGCCACTGGAGACGAGCAGCTA
shaker_Frag1011-1179_insertion1011	CGGTAAGACGTCTCCACACAGCGGGAGACCGGGGTCTCTGAGCTCCAAGGGGCTCCAAATTCTTGGTCGGACATTGAAGGCTTCTATGAGAGAACTCGGCCTCCTGATTTTTTTTCTCTTCATAGGGGTTGTATTGTTCAGTAGCGCAGTCTACTTTGCAGAAGCAGGATCTGAAAATAGCTTTTTTAAATCAATTCCTGACGCTTTCTGGTGGGGGAGACGCAGCTATA
shaker_Frag1011-1179_insertion1014	CGGTAAGACGTCTCCACACTCCAGCGGGAGACCGGGGTCTCTGAGCAAGGGGCTCCAAATTCTTGGTCGGACATTGAAGGCTTCTATGAGAGAACTCGGCCTCCTGATTTTTTTTCTCTTCATAGGGGTTGTATTGTTCAGTAGCGCAGTCTACTTTGCAGAAGCAGGATCTGAAAATAGCTTTTTTAAATCAATTCCTGACGCTTTCTGGTGGGGGAGACGCAGCTATA
shaker_Frag1011-1179_insertion1017	CGGTAAGACGTCTCCACACTCCAAGAGCGGGAGACCGGGGTCTCTGAGCGGGCTCCAAATTCTTGGTCGGACATTGAAGGCTTCTATGAGAGAACTCGGCCTCCTGATTTTTTTTCTCTTCATAGGGGTTGTATTGTTCAGTAGCGCAGTCTACTTTGCAGAAGCAGGATCTGAAAATAGCTTTTTTAAATCAATTCCTGACGCTTTCTGGTGGGGGAGACGCAGCTATA
shaker_Frag1011-1179_insertion1020	CGGTAAGACGTCTCCACACTCCAAGGGGAGCGGGAGACCGGGGTCTCTGAGCCTCCAAATTCTTGGTCGGACATTGAAGGCTTCTATGAGAGAACTCGGCCTCCTGATTTTTTTTCTCTTCATAGGGGTTGTATTGTTCAGTAGCGCAGTCTACTTTGCAGAAGCAGGATCTGAAAATAGCTTTTTTAAATCAATTCCTGACGCTTTCTGGTGGGGGAGACGCAGCTATA
shaker_Frag1011-1179_insertion1023	CGGTAAGACGTCTCCACACTCCAAGGGGCTCAGCGGGAGACCGGGGTCTCTGAGCCAAATTCTTGGTCGGACATTGAAGGCTTCTATGAGAGAACTCGGCCTCCTGATTTTTTTTCTCTTCATAGGGGTTGTATTGTTCAGTAGCGCAGTCTACTTTGCAGAAGCAGGATCTGAAAATAGCTTTTTTAAATCAATTCCTGACGCTTTCTGGTGGGGGAGACGCAGCTATA
shaker_Frag1011-1179_insertion1026	CGGTAAGACGTCTCCACACTCCAAGGGGCTCCAAAGCGGGAGACCGGGGTCTCTGAGCATTCTTGGTCGGACATTGAAGGCTTCTATGAGAGAACTCGGCCTCCTGATTTTTTTTCTCTTCATAGGGGTTGTATTGTTCAGTAGCGCAGTCTACTTTGCAGAAGCAGGATCTGAAAATAGCTTTTTTAAATCAATTCCTGACGCTTTCTGGTGGGGGAGACGCAGCTATA
shaker_Frag1011-1179_insertion1029	CGGTAAGACGTCTCCACACTCCAAGGGGCTCCAAATTAGCGGGAGACCGGGGTCTCTGAGCCTTGGTCGGACATTGAAGGCTTCTATGAGAGAACTCGGCCTCCTGATTTTTTTTCTCTTCATAGGGGTTGTATTGTTCAGTAGCGCAGTCTACTTTGCAGAAGCAGGATCTGAAAATAGCTTTTTTAAATCAATTCCTGACGCTTTCTGGTGGGGGAGACGCAGCTATA
shaker_Frag1011-1179_insertion1032	CGGTAAGACGTCTCCACACTCCAAGGGGCTCCAAATTCTTAGCGGGAGACCGGGGTCTCTGAGCGGTCGGACATTGAAGGCTTCTATGAGAGAACTCGGCCTCCTGATTTTTTTTCTCTTCATAGGGGTTGTATTGTTCAGTAGCGCAGTCTACTTTGCAGAAGCAGGATCTGAAAATAGCTTTTTTAAATCAATTCCTGACGCTTTCTGGTGGGGGAGACGCAGCTATA
shaker_Frag1011-1179_insertion1035	CGGTAAGACGTCTCCACACTCCAAGGGGCTCCAAATTCTTGGTAGCGGGAGACCGGGGTCTCTGAGCCGGACATTGAAGGCTTCTATGAGAGAACTCGGCCTCCTGATTTTTTTTCTCTTCATAGGGGTTGTATTGTTCAGTAGCGCAGTCTACTTTGCAGAAGCAGGATCTGAAAATAGCTTTTTTAAATCAATTCCTGACGCTTTCTGGTGGGGGAGACGCAGCTATA
shaker_Frag1011-1179_insertion1038	CGGTAAGACGTCTCCACACTCCAAGGGGCTCCAAATTCTTGGTCGGAGCGGGAGACCGGGGTCTCTGAGCACATTGAAGGCTTCTATGAGAGAACTCGGCCTCCTGATTTTTTTTCTCTTCATAGGGGTTGTATTGTTCAGTAGCGCAGTCTACTTTGCAGAAGCAGGATCTGAAAATAGCTTTTTTAAATCAATTCCTGACGCTTTCTGGTGGGGGAGACGCAGCTATA
shaker_Frag1011-1179_insertion1041	CGGTAAGACGTCTCCACACTCCAAGGGGCTCCAAATTCTTGGTCGGACAAGCGGGAGACCGGGGTCTCTGAGCTTGAAGGCTTCTATGAGAGAACTCGGCCTCCTGATTTTTTTTCTCTTCATAGGGGTTGTATTGTTCAGTAGCGCAGTCTACTTTGCAGAAGCAGGATCTGAAAATAGCTTTTTTAAATCAATTCCTGACGCTTTCTGGTGGGGGAGACGCAGCTATA
shaker_Frag1011-1179_insertion1044	CGGTAAGACGTCTCCACACTCCAAGGGGCTCCAAATTCTTGGTCGGACATTGAGCGGGAGACCGGGGTCTCTGAGCAAGGCTTCTATGAGAGAACTCGGCCTCCTGATTTTTTTTCTCTTCATAGGGGTTGTATTGTTCAGTAGCGCAGTCTACTTTGCAGAAGCAGGATCTGAAAATAGCTTTTTTAAATCAATTCCTGACGCTTTCTGGTGGGGGAGACGCAGCTATA
shaker_Frag1011-1179_insertion1047	CGGTAAGACGTCTCCACACTCCAAGGGGCTCCAAATTCTTGGTCGGACATTGAAGAGCGGGAGACCGGGGTCTCTGAGCGCTTCTATGAGAGAACTCGGCCTCCTGATTTTTTTTCTCTTCATAGGGGTTGTATTGTTCAGTAGCGCAGTCTACTTTGCAGAAGCAGGATCTGAAAATAGCTTTTTTAAATCAATTCCTGACGCTTTCTGGTGGGGGAGACGCAGCTATA
shaker_Frag1011-1179_insertion1050	CGGTAAGACGTCTCCACACTCCAAGGGGCTCCAAATTCTTGGTCGGACATTGAAGGCTAGCGGGAGACCGGGGTCTCTGAGCTCTATGAGAGAACTCGGCCTCCTGATTTTTTTTCTCTTCATAGGGGTTGTATTGTTCAGTAGCGCAGTCTACTTTGCAGAAGCAGGATCTGAAAATAGCTTTTTTAAATCAATTCCTGACGCTTTCTGGTGGGGGAGACGCAGCTATA
shaker_Frag1011-1179_insertion1053	CGGTAAGACGTCTCCACACTCCAAGGGGCTCCAAATTCTTGGTCGGACATTGAAGGCTTCTAGCGGGAGACCGGGGTCTCTGAGCATGAGAGAACTCGGCCTCCTGATTTTTTTTCTCTTCATAGGGGTTGTATTGTTCAGTAGCGCAGTCTACTTTGCAGAAGCAGGATCTGAAAATAGCTTTTTTAAATCAATTCCTGACGCTTTCTGGTGGGGGAGACGCAGCTATA
shaker_Frag1011-1179_insertion1056	CGGTAAGACGTCTCCACACTCCAAGGGGCTCCAAATTCTTGGTCGGACATTGAAGGCTTCTATGAGCGGGAGACCGGGGTCTCTGAGCAGAGAACTCGGCCTCCTGATTTTTTTTCTCTTCATAGGGGTTGTATTGTTCAGTAGCGCAGTCTACTTTGCAGAAGCAGGATCTGAAAATAGCTTTTTTAAATCAATTCCTGACGCTTTCTGGTGGGGGAGACGCAGCTATA
shaker_Frag1011-1179_insertion1059	CGGTAAGACGTCTCCACACTCCAAGGGGCTCCAAATTCTTGGTCGGACATTGAAGGCTTCTATGAGAAGCGGGAGACCGGGGTCTCTGAGCGAACTCGGCCTCCTGATTTTTTTTCTCTTCATAGGGGTTGTATTGTTCAGTAGCGCAGTCTACTTTGCAGAAGCAGGATCTGAAAATAGCTTTTTTAAATCAATTCCTGACGCTTTCTGGTGGGGGAGACGCAGCTATA
shaker_Frag1011-1179_insertion1062	CGGTAAGACGTCTCCACACTCCAAGGGGCTCCAAATTCTTGGTCGGACATTGAAGGCTTCTATGAGAGAAAGCGGGAGACCGGGGTCTCTGAGCCTCGGCCTCCTGATTTTTTTTCTCTTCATAGGGGTTGTATTGTTCAGTAGCGCAGTCTACTTTGCAGAAGCAGGATCTGAAAATAGCTTTTTTAAATCAATTCCTGACGCTTTCTGGTGGGGGAGACGCAGCTATA
shaker_Frag1011-1179_insertion1065	CGGTAAGACGTCTCCACACTCCAAGGGGCTCCAAATTCTTGGTCGGACATTGAAGGCTTCTATGAGAGAACTCAGCGGGAGACCGGGGTCTCTGAGCGGCCTCCTGATTTTTTTTCTCTTCATAGGGGTTGTATTGTTCAGTAGCGCAGTCTACTTTGCAGAAGCAGGATCTGAAAATAGCTTTTTTAAATCAATTCCTGACGCTTTCTGGTGGGGGAGACGCAGCTATA
shaker_Frag1011-1179_insertion1068	CGGTAAGACGTCTCCACACTCCAAGGGGCTCCAAATTCTTGGTCGGACATTGAAGGCTTCTATGAGAGAACTCGGCAGCGGGAGACCGGGGTCTCTGAGCCTCCTGATTTTTTTTCTCTTCATAGGGGTTGTATTGTTCAGTAGCGCAGTCTACTTTGCAGAAGCAGGATCTGAAAATAGCTTTTTTAAATCAATTCCTGACGCTTTCTGGTGGGGGAGACGCAGCTATA
shaker_Frag1011-1179_insertion1071	CGGTAAGACGTCTCCACACTCCAAGGGGCTCCAAATTCTTGGTCGGACATTGAAGGCTTCTATGAGAGAACTCGGCCTCAGCGGGAGACCGGGGTCTCTGAGCCTGATTTTTTTTCTCTTCATAGGGGTTGTATTGTTCAGTAGCGCAGTCTACTTTGCAGAAGCAGGATCTGAAAATAGCTTTTTTAAATCAATTCCTGACGCTTTCTGGTGGGGGAGACGCAGCTATA
shaker_Frag1011-1179_insertion1074	CGGTAAGACGTCTCCACACTCCAAGGGGCTCCAAATTCTTGGTCGGACATTGAAGGCTTCTATGAGAGAACTCGGCCTCCTGAGCGGGAGACCGGGGTCTCTGAGCATTTTTTTTCTCTTCATAGGGGTTGTATTGTTCAGTAGCGCAGTCTACTTTGCAGAAGCAGGATCTGAAAATAGCTTTTTTAAATCAATTCCTGACGCTTTCTGGTGGGGGAGACGCAGCTATA
shaker_Frag1011-1179_insertion1077	CGGTAAGACGTCTCCACACTCCAAGGGGCTCCAAATTCTTGGTCGGACATTGAAGGCTTCTATGAGAGAACTCGGCCTCCTGATTAGCGGGAGACCGGGGTCTCTGAGCTTTTTTCTCTTCATAGGGGTTGTATTGTTCAGTAGCGCAGTCTACTTTGCAGAAGCAGGATCTGAAAATAGCTTTTTTAAATCAATTCCTGACGCTTTCTGGTGGGGGAGACGCAGCTATA
shaker_Frag1011-1179_insertion1080	CGGTAAGACGTCTCCACACTCCAAGGGGCTCCAAATTCTTGGTCGGACATTGAAGGCTTCTATGAGAGAACTCGGCCTCCTGATTTTTAGCGGGAGACCGGGGTCTCTGAGCTTTCTCTTCATAGGGGTTGTATTGTTCAGTAGCGCAGTCTACTTTGCAGAAGCAGGATCTGAAAATAGCTTTTTTAAATCAATTCCTGACGCTTTCTGGTGGGGGAGACGCAGCTATA
shaker_Frag1011-1179_insertion1083	CGGTAAGACGTCTCCACACTCCAAGGGGCTCCAAATTCTTGGTCGGACATTGAAGGCTTCTATGAGAGAACTCGGCCTCCTGATTTTTTTTAGCGGGAGACCGGGGTCTCTGAGCCTCTTCATAGGGGTTGTATTGTTCAGTAGCGCAGTCTACTTTGCAGAAGCAGGATCTGAAAATAGCTTTTTTAAATCAATTCCTGACGCTTTCTGGTGGGGGAGACGCAGCTATA
shaker_Frag1011-1179_insertion1086	CGGTAAGACGTCTCCACACTCCAAGGGGCTCCAAATTCTTGGTCGGACATTGAAGGCTTCTATGAGAGAACTCGGCCTCCTGATTTTTTTTCTCAGCGGGAGACCGGGGTCTCTGAGCTTCATAGGGGTTGTATTGTTCAGTAGCGCAGTCTACTTTGCAGAAGCAGGATCTGAAAATAGCTTTTTTAAATCAATTCCTGACGCTTTCTGGTGGGGGAGACGCAGCTATA
shaker_Frag1011-1179_insertion1089	CGGTAAGACGTCTCCACACTCCAAGGGGCTCCAAATTCTTGGTCGGACATTGAAGGCTTCTATGAGAGAACTCGGCCTCCTGATTTTTTTTCTCTTCAGCGGGAGACCGGGGTCTCTGAGCATAGGGGTTGTATTGTTCAGTAGCGCAGTCTACTTTGCAGAAGCAGGATCTGAAAATAGCTTTTTTAAATCAATTCCTGACGCTTTCTGGTGGGGGAGACGCAGCTATA
shaker_Frag1011-1179_insertion1092	CGGTAAGACGTCTCCACACTCCAAGGGGCTCCAAATTCTTGGTCGGACATTGAAGGCTTCTATGAGAGAACTCGGCCTCCTGATTTTTTTTCTCTTCATAAGCGGGAGACCGGGGTCTCTGAGCGGGGTTGTATTGTTCAGTAGCGCAGTCTACTTTGCAGAAGCAGGATCTGAAAATAGCTTTTTTAAATCAATTCCTGACGCTTTCTGGTGGGGGAGACGCAGCTATA
shaker_Frag1011-1179_insertion1095	CGGTAAGACGTCTCCACACTCCAAGGGGCTCCAAATTCTTGGTCGGACATTGAAGGCTTCTATGAGAGAACTCGGCCTCCTGATTTTTTTTCTCTTCATAGGGAGCGGGAGACCGGGGTCTCTGAGCGTTGTATTGTTCAGTAGCGCAGTCTACTTTGCAGAAGCAGGATCTGAAAATAGCTTTTTTAAATCAATTCCTGACGCTTTCTGGTGGGGGAGACGCAGCTATA
shaker_Frag1011-1179_insertion1098	CGGTAAGACGTCTCCACACTCCAAGGGGCTCCAAATTCTTGGTCGGACATTGAAGGCTTCTATGAGAGAACTCGGCCTCCTGATTTTTTTTCTCTTCATAGGGGTTAGCGGGAGACCGGGGTCTCTGAGCGTATTGTTCAGTAGCGCAGTCTACTTTGCAGAAGCAGGATCTGAAAATAGCTTTTTTAAATCAATTCCTGACGCTTTCTGGTGGGGGAGACGCAGCTATA
shaker_Frag1011-1179_insertion1101	CGGTAAGACGTCTCCACACTCCAAGGGGCTCCAAATTCTTGGTCGGACATTGAAGGCTTCTATGAGAGAACTCGGCCTCCTGATTTTTTTTCTCTTCATAGGGGTTGTAAGCGGGAGACCGGGGTCTCTGAGCTTGTTCAGTAGCGCAGTCTACTTTGCAGAAGCAGGATCTGAAAATAGCTTTTTTAAATCAATTCCTGACGCTTTCTGGTGGGGGAGACGCAGCTATA
shaker_Frag1011-1179_insertion1104	CGGTAAGACGTCTCCACACTCCAAGGGGCTCCAAATTCTTGGTCGGACATTGAAGGCTTCTATGAGAGAACTCGGCCTCCTGATTTTTTTTCTCTTCATAGGGGTTGTATTGAGCGGGAGACCGGGGTCTCTGAGCTTCAGTAGCGCAGTCTACTTTGCAGAAGCAGGATCTGAAAATAGCTTTTTTAAATCAATTCCTGACGCTTTCTGGTGGGGGAGACGCAGCTATA
shaker_Frag1011-1179_insertion1107	CGGTAAGACGTCTCCACACTCCAAGGGGCTCCAAATTCTTGGTCGGACATTGAAGGCTTCTATGAGAGAACTCGGCCTCCTGATTTTTTTTCTCTTCATAGGGGTTGTATTGTTCAGCGGGAGACCGGGGTCTCTGAGCAGTAGCGCAGTCTACTTTGCAGAAGCAGGATCTGAAAATAGCTTTTTTAAATCAATTCCTGACGCTTTCTGGTGGGGGAGACGCAGCTATA
shaker_Frag1011-1179_insertion1110	CGGTAAGACGTCTCCACACTCCAAGGGGCTCCAAATTCTTGGTCGGACATTGAAGGCTTCTATGAGAGAACTCGGCCTCCTGATTTTTTTTCTCTTCATAGGGGTTGTATTGTTCAGTAGCGGGAGACCGGGGTCTCTGAGCAGCGCAGTCTACTTTGCAGAAGCAGGATCTGAAAATAGCTTTTTTAAATCAATTCCTGACGCTTTCTGGTGGGGGAGACGCAGCTATA
shaker_Frag1011-1179_insertion1113	CGGTAAGACGTCTCCACACTCCAAGGGGCTCCAAATTCTTGGTCGGACATTGAAGGCTTCTATGAGAGAACTCGGCCTCCTGATTTTTTTTCTCTTCATAGGGGTTGTATTGTTCAGTAGCAGCGGGAGACCGGGGTCTCTGAGCGCAGTCTACTTTGCAGAAGCAGGATCTGAAAATAGCTTTTTTAAATCAATTCCTGACGCTTTCTGGTGGGGGAGACGCAGCTATA
shaker_Frag1011-1179_insertion1116	CGGTAAGACGTCTCCACACTCCAAGGGGCTCCAAATTCTTGGTCGGACATTGAAGGCTTCTATGAGAGAACTCGGCCTCCTGATTTTTTTTCTCTTCATAGGGGTTGTATTGTTCAGTAGCGCAAGCGGGAGACCGGGGTCTCTGAGCGTCTACTTTGCAGAAGCAGGATCTGAAAATAGCTTTTTTAAATCAATTCCTGACGCTTTCTGGTGGGGGAGACGCAGCTATA
shaker_Frag1011-1179_insertion1119	CGGTAAGACGTCTCCACACTCCAAGGGGCTCCAAATTCTTGGTCGGACATTGAAGGCTTCTATGAGAGAACTCGGCCTCCTGATTTTTTTTCTCTTCATAGGGGTTGTATTGTTCAGTAGCGCAGTCAGCGGGAGACCGGGGTCTCTGAGCTACTTTGCAGAAGCAGGATCTGAAAATAGCTTTTTTAAATCAATTCCTGACGCTTTCTGGTGGGGGAGACGCAGCTATA
shaker_Frag1011-1179_insertion1122	CGGTAAGACGTCTCCACACTCCAAGGGGCTCCAAATTCTTGGTCGGACATTGAAGGCTTCTATGAGAGAACTCGGCCTCCTGATTTTTTTTCTCTTCATAGGGGTTGTATTGTTCAGTAGCGCAGTCTACAGCGGGAGACCGGGGTCTCTGAGCTTTGCAGAAGCAGGATCTGAAAATAGCTTTTTTAAATCAATTCCTGACGCTTTCTGGTGGGGGAGACGCAGCTATA
shaker_Frag1011-1179_insertion1125	CGGTAAGACGTCTCCACACTCCAAGGGGCTCCAAATTCTTGGTCGGACATTGAAGGCTTCTATGAGAGAACTCGGCCTCCTGATTTTTTTTCTCTTCATAGGGGTTGTATTGTTCAGTAGCGCAGTCTACTTTAGCGGGAGACCGGGGTCTCTGAGCGCAGAAGCAGGATCTGAAAATAGCTTTTTTAAATCAATTCCTGACGCTTTCTGGTGGGGGAGACGCAGCTATA
shaker_Frag1011-1179_insertion1128	CGGTAAGACGTCTCCACACTCCAAGGGGCTCCAAATTCTTGGTCGGACATTGAAGGCTTCTATGAGAGAACTCGGCCTCCTGATTTTTTTTCTCTTCATAGGGGTTGTATTGTTCAGTAGCGCAGTCTACTTTGCAAGCGGGAGACCGGGGTCTCTGAGCGAAGCAGGATCTGAAAATAGCTTTTTTAAATCAATTCCTGACGCTTTCTGGTGGGGGAGACGCAGCTATA
shaker_Frag1011-1179_insertion1131	CGGTAAGACGTCTCCACACTCCAAGGGGCTCCAAATTCTTGGTCGGACATTGAAGGCTTCTATGAGAGAACTCGGCCTCCTGATTTTTTTTCTCTTCATAGGGGTTGTATTGTTCAGTAGCGCAGTCTACTTTGCAGAAAGCGGGAGACCGGGGTCTCTGAGCGCAGGATCTGAAAATAGCTTTTTTAAATCAATTCCTGACGCTTTCTGGTGGGGGAGACGCAGCTATA
shaker_Frag1011-1179_insertion1134	CGGTAAGACGTCTCCACACTCCAAGGGGCTCCAAATTCTTGGTCGGACATTGAAGGCTTCTATGAGAGAACTCGGCCTCCTGATTTTTTTTCTCTTCATAGGGGTTGTATTGTTCAGTAGCGCAGTCTACTTTGCAGAAGCAAGCGGGAGACCGGGGTCTCTGAGCGGATCTGAAAATAGCTTTTTTAAATCAATTCCTGACGCTTTCTGGTGGGGGAGACGCAGCTATA
shaker_Frag1011-1179_insertion1137	CGGTAAGACGTCTCCACACTCCAAGGGGCTCCAAATTCTTGGTCGGACATTGAAGGCTTCTATGAGAGAACTCGGCCTCCTGATTTTTTTTCTCTTCATAGGGGTTGTATTGTTCAGTAGCGCAGTCTACTTTGCAGAAGCAGGAAGCGGGAGACCGGGGTCTCTGAGCTCTGAAAATAGCTTTTTTAAATCAATTCCTGACGCTTTCTGGTGGGGGAGACGCAGCTATA
shaker_Frag1011-1179_insertion1140	CGGTAAGACGTCTCCACACTCCAAGGGGCTCCAAATTCTTGGTCGGACATTGAAGGCTTCTATGAGAGAACTCGGCCTCCTGATTTTTTTTCTCTTCATAGGGGTTGTATTGTTCAGTAGCGCAGTCTACTTTGCAGAAGCAGGATCTAGCGGGAGACCGGGGTCTCTGAGCGAAAATAGCTTTTTTAAATCAATTCCTGACGCTTTCTGGTGGGGGAGACGCAGCTATA
shaker_Frag1011-1179_insertion1143	CGGTAAGACGTCTCCACACTCCAAGGGGCTCCAAATTCTTGGTCGGACATTGAAGGCTTCTATGAGAGAACTCGGCCTCCTGATTTTTTTTCTCTTCATAGGGGTTGTATTGTTCAGTAGCGCAGTCTACTTTGCAGAAGCAGGATCTGAAAGCGGGAGACCGGGGTCTCTGAGCAATAGCTTTTTTAAATCAATTCCTGACGCTTTCTGGTGGGGGAGACGCAGCTATA
shaker_Frag1011-1179_insertion1146	CGGTAAGACGTCTCCACACTCCAAGGGGCTCCAAATTCTTGGTCGGACATTGAAGGCTTCTATGAGAGAACTCGGCCTCCTGATTTTTTTTCTCTTCATAGGGGTTGTATTGTTCAGTAGCGCAGTCTACTTTGCAGAAGCAGGATCTGAAAATAGCGGGAGACCGGGGTCTCTGAGCAGCTTTTTTAAATCAATTCCTGACGCTTTCTGGTGGGGGAGACGCAGCTATA
shaker_Frag1011-1179_insertion1149	CGGTAAGACGTCTCCACACTCCAAGGGGCTCCAAATTCTTGGTCGGACATTGAAGGCTTCTATGAGAGAACTCGGCCTCCTGATTTTTTTTCTCTTCATAGGGGTTGTATTGTTCAGTAGCGCAGTCTACTTTGCAGAAGCAGGATCTGAAAATAGCAGCGGGAGACCGGGGTCTCTGAGCTTTTTTAAATCAATTCCTGACGCTTTCTGGTGGGGGAGACGCAGCTATA
shaker_Frag1011-1179_insertion1152	CGGTAAGACGTCTCCACACTCCAAGGGGCTCCAAATTCTTGGTCGGACATTGAAGGCTTCTATGAGAGAACTCGGCCTCCTGATTTTTTTTCTCTTCATAGGGGTTGTATTGTTCAGTAGCGCAGTCTACTTTGCAGAAGCAGGATCTGAAAATAGCTTTAGCGGGAGACCGGGGTCTCTGAGCTTTAAATCAATTCCTGACGCTTTCTGGTGGGGGAGACGCAGCTATA
shaker_Frag1011-1179_insertion1155	CGGTAAGACGTCTCCACACTCCAAGGGGCTCCAAATTCTTGGTCGGACATTGAAGGCTTCTATGAGAGAACTCGGCCTCCTGATTTTTTTTCTCTTCATAGGGGTTGTATTGTTCAGTAGCGCAGTCTACTTTGCAGAAGCAGGATCTGAAAATAGCTTTTTTAGCGGGAGACCGGGGTCTCTGAGCAAATCAATTCCTGACGCTTTCTGGTGGGGGAGACGCAGCTATA
shaker_Frag1011-1179_insertion1158	CGGTAAGACGTCTCCACACTCCAAGGGGCTCCAAATTCTTGGTCGGACATTGAAGGCTTCTATGAGAGAACTCGGCCTCCTGATTTTTTTTCTCTTCATAGGGGTTGTATTGTTCAGTAGCGCAGTCTACTTTGCAGAAGCAGGATCTGAAAATAGCTTTTTTAAAAGCGGGAGACCGGGGTCTCTGAGCTCAATTCCTGACGCTTTCTGGTGGGGGAGACGCAGCTATA
shaker_Frag1011-1179_insertion1161	CGGTAAGACGTCTCCACACTCCAAGGGGCTCCAAATTCTTGGTCGGACATTGAAGGCTTCTATGAGAGAACTCGGCCTCCTGATTTTTTTTCTCTTCATAGGGGTTGTATTGTTCAGTAGCGCAGTCTACTTTGCAGAAGCAGGATCTGAAAATAGCTTTTTTAAATCAAGCGGGAGACCGGGGTCTCTGAGCATTCCTGACGCTTTCTGGTGGGGGAGACGCAGCTATA
shaker_Frag1011-1179_insertion1164	CGGTAAGACGTCTCCACACTCCAAGGGGCTCCAAATTCTTGGTCGGACATTGAAGGCTTCTATGAGAGAACTCGGCCTCCTGATTTTTTTTCTCTTCATAGGGGTTGTATTGTTCAGTAGCGCAGTCTACTTTGCAGAAGCAGGATCTGAAAATAGCTTTTTTAAATCAATTAGCGGGAGACCGGGGTCTCTGAGCCCTGACGCTTTCTGGTGGGGGAGACGCAGCTATA
shaker_Frag1011-1179_insertion1167	CGGTAAGACGTCTCCACACTCCAAGGGGCTCCAAATTCTTGGTCGGACATTGAAGGCTTCTATGAGAGAACTCGGCCTCCTGATTTTTTTTCTCTTCATAGGGGTTGTATTGTTCAGTAGCGCAGTCTACTTTGCAGAAGCAGGATCTGAAAATAGCTTTTTTAAATCAATTCCTAGCGGGAGACCGGGGTCTCTGAGCGACGCTTTCTGGTGGGGGAGACGCAGCTATA
shaker_Frag1011-1179_insertion1170	CGGTAAGACGTCTCCACACTCCAAGGGGCTCCAAATTCTTGGTCGGACATTGAAGGCTTCTATGAGAGAACTCGGCCTCCTGATTTTTTTTCTCTTCATAGGGGTTGTATTGTTCAGTAGCGCAGTCTACTTTGCAGAAGCAGGATCTGAAAATAGCTTTTTTAAATCAATTCCTGACAGCGGGAGACCGGGGTCTCTGAGCGCTTTCTGGTGGGGGAGACGCAGCTATA
shaker_Frag1011-1179_insertion1173	CGGTAAGACGTCTCCACACTCCAAGGGGCTCCAAATTCTTGGTCGGACATTGAAGGCTTCTATGAGAGAACTCGGCCTCCTGATTTTTTTTCTCTTCATAGGGGTTGTATTGTTCAGTAGCGCAGTCTACTTTGCAGAAGCAGGATCTGAAAATAGCTTTTTTAAATCAATTCCTGACGCTAGCGGGAGACCGGGGTCTCTGAGCTTCTGGTGGGGGAGACGCAGCTATA
shaker_Frag1011-1179_insertion1176	CGGTAAGACGTCTCCACACTCCAAGGGGCTCCAAATTCTTGGTCGGACATTGAAGGCTTCTATGAGAGAACTCGGCCTCCTGATTTTTTTTCTCTTCATAGGGGTTGTATTGTTCAGTAGCGCAGTCTACTTTGCAGAAGCAGGATCTGAAAATAGCTTTTTTAAATCAATTCCTGACGCTTTCAGCGGGAGACCGGGGTCTCTGAGCTGGTGGGGGAGACGCAGCTATA
shaker_Frag1011-1179_insertion1179	CGGTAAGACGTCTCCACACTCCAAGGGGCTCCAAATTCTTGGTCGGACATTGAAGGCTTCTATGAGAGAACTCGGCCTCCTGATTTTTTTTCTCTTCATAGGGGTTGTATTGTTCAGTAGCGCAGTCTACTTTGCAGAAGCAGGATCTGAAAATAGCTTTTTTAAATCAATTCCTGACGCTTTCTGGAGCGGGAGACCGGGGTCTCTGAGCTGGGGGAGACGCAGCTATA
shaker_Frag1182-1350_insertion1182	AACTCACGCGTCTCCGTGGAGCGGGAGACCGGGGTCTCTGAGCGCCGTCGTGACTATGACAACTGTAGGATACGGAGATATGACCCCTGTTGGAGTTTGGGGCAAAATAGTCGGAAGCCTTTGTGCTATTGCAGGCGTACTGACCATAGCCTTGCCAGTTCCAGTAATCGTTTCTAATTTTAATTATTTCTACCATCGAGAGACTGACCAGGAAGGGAGACGGGCGCAAC
shaker_Frag1182-1350_insertion1185	AACTCACGCGTCTCCGTGGGCCAGCGGGAGACCGGGGTCTCTGAGCGTCGTGACTATGACAACTGTAGGATACGGAGATATGACCCCTGTTGGAGTTTGGGGCAAAATAGTCGGAAGCCTTTGTGCTATTGCAGGCGTACTGACCATAGCCTTGCCAGTTCCAGTAATCGTTTCTAATTTTAATTATTTCTACCATCGAGAGACTGACCAGGAAGGGAGACGGGCGCAAC
shaker_Frag1182-1350_insertion1188	AACTCACGCGTCTCCGTGGGCCGTCAGCGGGAGACCGGGGTCTCTGAGCGTGACTATGACAACTGTAGGATACGGAGATATGACCCCTGTTGGAGTTTGGGGCAAAATAGTCGGAAGCCTTTGTGCTATTGCAGGCGTACTGACCATAGCCTTGCCAGTTCCAGTAATCGTTTCTAATTTTAATTATTTCTACCATCGAGAGACTGACCAGGAAGGGAGACGGGCGCAAC
shaker_Frag1182-1350_insertion1191	AACTCACGCGTCTCCGTGGGCCGTCGTGAGCGGGAGACCGGGGTCTCTGAGCACTATGACAACTGTAGGATACGGAGATATGACCCCTGTTGGAGTTTGGGGCAAAATAGTCGGAAGCCTTTGTGCTATTGCAGGCGTACTGACCATAGCCTTGCCAGTTCCAGTAATCGTTTCTAATTTTAATTATTTCTACCATCGAGAGACTGACCAGGAAGGGAGACGGGCGCAAC
shaker_Frag1182-1350_insertion1194	AACTCACGCGTCTCCGTGGGCCGTCGTGACTAGCGGGAGACCGGGGTCTCTGAGCATGACAACTGTAGGATACGGAGATATGACCCCTGTTGGAGTTTGGGGCAAAATAGTCGGAAGCCTTTGTGCTATTGCAGGCGTACTGACCATAGCCTTGCCAGTTCCAGTAATCGTTTCTAATTTTAATTATTTCTACCATCGAGAGACTGACCAGGAAGGGAGACGGGCGCAAC
shaker_Frag1182-1350_insertion1197	AACTCACGCGTCTCCGTGGGCCGTCGTGACTATGAGCGGGAGACCGGGGTCTCTGAGCACAACTGTAGGATACGGAGATATGACCCCTGTTGGAGTTTGGGGCAAAATAGTCGGAAGCCTTTGTGCTATTGCAGGCGTACTGACCATAGCCTTGCCAGTTCCAGTAATCGTTTCTAATTTTAATTATTTCTACCATCGAGAGACTGACCAGGAAGGGAGACGGGCGCAAC
shaker_Frag1182-1350_insertion1200	AACTCACGCGTCTCCGTGGGCCGTCGTGACTATGACAAGCGGGAGACCGGGGTCTCTGAGCACTGTAGGATACGGAGATATGACCCCTGTTGGAGTTTGGGGCAAAATAGTCGGAAGCCTTTGTGCTATTGCAGGCGTACTGACCATAGCCTTGCCAGTTCCAGTAATCGTTTCTAATTTTAATTATTTCTACCATCGAGAGACTGACCAGGAAGGGAGACGGGCGCAAC
shaker_Frag1182-1350_insertion1203	AACTCACGCGTCTCCGTGGGCCGTCGTGACTATGACAACTAGCGGGAGACCGGGGTCTCTGAGCGTAGGATACGGAGATATGACCCCTGTTGGAGTTTGGGGCAAAATAGTCGGAAGCCTTTGTGCTATTGCAGGCGTACTGACCATAGCCTTGCCAGTTCCAGTAATCGTTTCTAATTTTAATTATTTCTACCATCGAGAGACTGACCAGGAAGGGAGACGGGCGCAAC
shaker_Frag1182-1350_insertion1206	AACTCACGCGTCTCCGTGGGCCGTCGTGACTATGACAACTGTAAGCGGGAGACCGGGGTCTCTGAGCGGATACGGAGATATGACCCCTGTTGGAGTTTGGGGCAAAATAGTCGGAAGCCTTTGTGCTATTGCAGGCGTACTGACCATAGCCTTGCCAGTTCCAGTAATCGTTTCTAATTTTAATTATTTCTACCATCGAGAGACTGACCAGGAAGGGAGACGGGCGCAAC
shaker_Frag1182-1350_insertion1209	AACTCACGCGTCTCCGTGGGCCGTCGTGACTATGACAACTGTAGGAAGCGGGAGACCGGGGTCTCTGAGCTACGGAGATATGACCCCTGTTGGAGTTTGGGGCAAAATAGTCGGAAGCCTTTGTGCTATTGCAGGCGTACTGACCATAGCCTTGCCAGTTCCAGTAATCGTTTCTAATTTTAATTATTTCTACCATCGAGAGACTGACCAGGAAGGGAGACGGGCGCAAC
shaker_Frag1182-1350_insertion1212	AACTCACGCGTCTCCGTGGGCCGTCGTGACTATGACAACTGTAGGATACAGCGGGAGACCGGGGTCTCTGAGCGGAGATATGACCCCTGTTGGAGTTTGGGGCAAAATAGTCGGAAGCCTTTGTGCTATTGCAGGCGTACTGACCATAGCCTTGCCAGTTCCAGTAATCGTTTCTAATTTTAATTATTTCTACCATCGAGAGACTGACCAGGAAGGGAGACGGGCGCAAC
shaker_Frag1182-1350_insertion1215	AACTCACGCGTCTCCGTGGGCCGTCGTGACTATGACAACTGTAGGATACGGAAGCGGGAGACCGGGGTCTCTGAGCGATATGACCCCTGTTGGAGTTTGGGGCAAAATAGTCGGAAGCCTTTGTGCTATTGCAGGCGTACTGACCATAGCCTTGCCAGTTCCAGTAATCGTTTCTAATTTTAATTATTTCTACCATCGAGAGACTGACCAGGAAGGGAGACGGGCGCAAC
shaker_Frag1182-1350_insertion1218	AACTCACGCGTCTCCGTGGGCCGTCGTGACTATGACAACTGTAGGATACGGAGATAGCGGGAGACCGGGGTCTCTGAGCATGACCCCTGTTGGAGTTTGGGGCAAAATAGTCGGAAGCCTTTGTGCTATTGCAGGCGTACTGACCATAGCCTTGCCAGTTCCAGTAATCGTTTCTAATTTTAATTATTTCTACCATCGAGAGACTGACCAGGAAGGGAGACGGGCGCAAC
shaker_Frag1182-1350_insertion1221	AACTCACGCGTCTCCGTGGGCCGTCGTGACTATGACAACTGTAGGATACGGAGATATGAGCGGGAGACCGGGGTCTCTGAGCACCCCTGTTGGAGTTTGGGGCAAAATAGTCGGAAGCCTTTGTGCTATTGCAGGCGTACTGACCATAGCCTTGCCAGTTCCAGTAATCGTTTCTAATTTTAATTATTTCTACCATCGAGAGACTGACCAGGAAGGGAGACGGGCGCAAC
shaker_Frag1182-1350_insertion1224	AACTCACGCGTCTCCGTGGGCCGTCGTGACTATGACAACTGTAGGATACGGAGATATGACCAGCGGGAGACCGGGGTCTCTGAGCCCTGTTGGAGTTTGGGGCAAAATAGTCGGAAGCCTTTGTGCTATTGCAGGCGTACTGACCATAGCCTTGCCAGTTCCAGTAATCGTTTCTAATTTTAATTATTTCTACCATCGAGAGACTGACCAGGAAGGGAGACGGGCGCAAC
shaker_Frag1182-1350_insertion1227	AACTCACGCGTCTCCGTGGGCCGTCGTGACTATGACAACTGTAGGATACGGAGATATGACCCCTAGCGGGAGACCGGGGTCTCTGAGCGTTGGAGTTTGGGGCAAAATAGTCGGAAGCCTTTGTGCTATTGCAGGCGTACTGACCATAGCCTTGCCAGTTCCAGTAATCGTTTCTAATTTTAATTATTTCTACCATCGAGAGACTGACCAGGAAGGGAGACGGGCGCAAC
shaker_Frag1182-1350_insertion1230	AACTCACGCGTCTCCGTGGGCCGTCGTGACTATGACAACTGTAGGATACGGAGATATGACCCCTGTTAGCGGGAGACCGGGGTCTCTGAGCGGAGTTTGGGGCAAAATAGTCGGAAGCCTTTGTGCTATTGCAGGCGTACTGACCATAGCCTTGCCAGTTCCAGTAATCGTTTCTAATTTTAATTATTTCTACCATCGAGAGACTGACCAGGAAGGGAGACGGGCGCAAC
shaker_Frag1182-1350_insertion1233	AACTCACGCGTCTCCGTGGGCCGTCGTGACTATGACAACTGTAGGATACGGAGATATGACCCCTGTTGGAAGCGGGAGACCGGGGTCTCTGAGCGTTTGGGGCAAAATAGTCGGAAGCCTTTGTGCTATTGCAGGCGTACTGACCATAGCCTTGCCAGTTCCAGTAATCGTTTCTAATTTTAATTATTTCTACCATCGAGAGACTGACCAGGAAGGGAGACGGGCGCAAC
shaker_Frag1182-1350_insertion1236	AACTCACGCGTCTCCGTGGGCCGTCGTGACTATGACAACTGTAGGATACGGAGATATGACCCCTGTTGGAGTTAGCGGGAGACCGGGGTCTCTGAGCTGGGGCAAAATAGTCGGAAGCCTTTGTGCTATTGCAGGCGTACTGACCATAGCCTTGCCAGTTCCAGTAATCGTTTCTAATTTTAATTATTTCTACCATCGAGAGACTGACCAGGAAGGGAGACGGGCGCAAC
shaker_Frag1182-1350_insertion1239	AACTCACGCGTCTCCGTGGGCCGTCGTGACTATGACAACTGTAGGATACGGAGATATGACCCCTGTTGGAGTTTGGAGCGGGAGACCGGGGTCTCTGAGCGGCAAAATAGTCGGAAGCCTTTGTGCTATTGCAGGCGTACTGACCATAGCCTTGCCAGTTCCAGTAATCGTTTCTAATTTTAATTATTTCTACCATCGAGAGACTGACCAGGAAGGGAGACGGGCGCAAC
shaker_Frag1182-1350_insertion1242	AACTCACGCGTCTCCGTGGGCCGTCGTGACTATGACAACTGTAGGATACGGAGATATGACCCCTGTTGGAGTTTGGGGCAGCGGGAGACCGGGGTCTCTGAGCAAAATAGTCGGAAGCCTTTGTGCTATTGCAGGCGTACTGACCATAGCCTTGCCAGTTCCAGTAATCGTTTCTAATTTTAATTATTTCTACCATCGAGAGACTGACCAGGAAGGGAGACGGGCGCAAC
shaker_Frag1182-1350_insertion1245	AACTCACGCGTCTCCGTGGGCCGTCGTGACTATGACAACTGTAGGATACGGAGATATGACCCCTGTTGGAGTTTGGGGCAAAAGCGGGAGACCGGGGTCTCTGAGCATAGTCGGAAGCCTTTGTGCTATTGCAGGCGTACTGACCATAGCCTTGCCAGTTCCAGTAATCGTTTCTAATTTTAATTATTTCTACCATCGAGAGACTGACCAGGAAGGGAGACGGGCGCAAC
shaker_Frag1182-1350_insertion1248	AACTCACGCGTCTCCGTGGGCCGTCGTGACTATGACAACTGTAGGATACGGAGATATGACCCCTGTTGGAGTTTGGGGCAAAATAAGCGGGAGACCGGGGTCTCTGAGCGTCGGAAGCCTTTGTGCTATTGCAGGCGTACTGACCATAGCCTTGCCAGTTCCAGTAATCGTTTCTAATTTTAATTATTTCTACCATCGAGAGACTGACCAGGAAGGGAGACGGGCGCAAC
shaker_Frag1182-1350_insertion1251	AACTCACGCGTCTCCGTGGGCCGTCGTGACTATGACAACTGTAGGATACGGAGATATGACCCCTGTTGGAGTTTGGGGCAAAATAGTCAGCGGGAGACCGGGGTCTCTGAGCGGAAGCCTTTGTGCTATTGCAGGCGTACTGACCATAGCCTTGCCAGTTCCAGTAATCGTTTCTAATTTTAATTATTTCTACCATCGAGAGACTGACCAGGAAGGGAGACGGGCGCAAC
shaker_Frag1182-1350_insertion1254	AACTCACGCGTCTCCGTGGGCCGTCGTGACTATGACAACTGTAGGATACGGAGATATGACCCCTGTTGGAGTTTGGGGCAAAATAGTCGGAAGCGGGAGACCGGGGTCTCTGAGCAGCCTTTGTGCTATTGCAGGCGTACTGACCATAGCCTTGCCAGTTCCAGTAATCGTTTCTAATTTTAATTATTTCTACCATCGAGAGACTGACCAGGAAGGGAGACGGGCGCAAC
shaker_Frag1182-1350_insertion1257	AACTCACGCGTCTCCGTGGGCCGTCGTGACTATGACAACTGTAGGATACGGAGATATGACCCCTGTTGGAGTTTGGGGCAAAATAGTCGGAAGCAGCGGGAGACCGGGGTCTCTGAGCCTTTGTGCTATTGCAGGCGTACTGACCATAGCCTTGCCAGTTCCAGTAATCGTTTCTAATTTTAATTATTTCTACCATCGAGAGACTGACCAGGAAGGGAGACGGGCGCAAC
shaker_Frag1182-1350_insertion1260	AACTCACGCGTCTCCGTGGGCCGTCGTGACTATGACAACTGTAGGATACGGAGATATGACCCCTGTTGGAGTTTGGGGCAAAATAGTCGGAAGCCTTAGCGGGAGACCGGGGTCTCTGAGCTGTGCTATTGCAGGCGTACTGACCATAGCCTTGCCAGTTCCAGTAATCGTTTCTAATTTTAATTATTTCTACCATCGAGAGACTGACCAGGAAGGGAGACGGGCGCAAC
shaker_Frag1182-1350_insertion1263	AACTCACGCGTCTCCGTGGGCCGTCGTGACTATGACAACTGTAGGATACGGAGATATGACCCCTGTTGGAGTTTGGGGCAAAATAGTCGGAAGCCTTTGTAGCGGGAGACCGGGGTCTCTGAGCGCTATTGCAGGCGTACTGACCATAGCCTTGCCAGTTCCAGTAATCGTTTCTAATTTTAATTATTTCTACCATCGAGAGACTGACCAGGAAGGGAGACGGGCGCAAC
shaker_Frag1182-1350_insertion1266	AACTCACGCGTCTCCGTGGGCCGTCGTGACTATGACAACTGTAGGATACGGAGATATGACCCCTGTTGGAGTTTGGGGCAAAATAGTCGGAAGCCTTTGTGCTAGCGGGAGACCGGGGTCTCTGAGCATTGCAGGCGTACTGACCATAGCCTTGCCAGTTCCAGTAATCGTTTCTAATTTTAATTATTTCTACCATCGAGAGACTGACCAGGAAGGGAGACGGGCGCAAC
shaker_Frag1182-1350_insertion1269	AACTCACGCGTCTCCGTGGGCCGTCGTGACTATGACAACTGTAGGATACGGAGATATGACCCCTGTTGGAGTTTGGGGCAAAATAGTCGGAAGCCTTTGTGCTATTAGCGGGAGACCGGGGTCTCTGAGCGCAGGCGTACTGACCATAGCCTTGCCAGTTCCAGTAATCGTTTCTAATTTTAATTATTTCTACCATCGAGAGACTGACCAGGAAGGGAGACGGGCGCAAC
shaker_Frag1182-1350_insertion1272	AACTCACGCGTCTCCGTGGGCCGTCGTGACTATGACAACTGTAGGATACGGAGATATGACCCCTGTTGGAGTTTGGGGCAAAATAGTCGGAAGCCTTTGTGCTATTGCAAGCGGGAGACCGGGGTCTCTGAGCGGCGTACTGACCATAGCCTTGCCAGTTCCAGTAATCGTTTCTAATTTTAATTATTTCTACCATCGAGAGACTGACCAGGAAGGGAGACGGGCGCAAC
shaker_Frag1182-1350_insertion1275	AACTCACGCGTCTCCGTGGGCCGTCGTGACTATGACAACTGTAGGATACGGAGATATGACCCCTGTTGGAGTTTGGGGCAAAATAGTCGGAAGCCTTTGTGCTATTGCAGGCAGCGGGAGACCGGGGTCTCTGAGCGTACTGACCATAGCCTTGCCAGTTCCAGTAATCGTTTCTAATTTTAATTATTTCTACCATCGAGAGACTGACCAGGAAGGGAGACGGGCGCAAC
shaker_Frag1182-1350_insertion1278	AACTCACGCGTCTCCGTGGGCCGTCGTGACTATGACAACTGTAGGATACGGAGATATGACCCCTGTTGGAGTTTGGGGCAAAATAGTCGGAAGCCTTTGTGCTATTGCAGGCGTAAGCGGGAGACCGGGGTCTCTGAGCCTGACCATAGCCTTGCCAGTTCCAGTAATCGTTTCTAATTTTAATTATTTCTACCATCGAGAGACTGACCAGGAAGGGAGACGGGCGCAAC
shaker_Frag1182-1350_insertion1281	AACTCACGCGTCTCCGTGGGCCGTCGTGACTATGACAACTGTAGGATACGGAGATATGACCCCTGTTGGAGTTTGGGGCAAAATAGTCGGAAGCCTTTGTGCTATTGCAGGCGTACTGAGCGGGAGACCGGGGTCTCTGAGCACCATAGCCTTGCCAGTTCCAGTAATCGTTTCTAATTTTAATTATTTCTACCATCGAGAGACTGACCAGGAAGGGAGACGGGCGCAAC
shaker_Frag1182-1350_insertion1284	AACTCACGCGTCTCCGTGGGCCGTCGTGACTATGACAACTGTAGGATACGGAGATATGACCCCTGTTGGAGTTTGGGGCAAAATAGTCGGAAGCCTTTGTGCTATTGCAGGCGTACTGACCAGCGGGAGACCGGGGTCTCTGAGCATAGCCTTGCCAGTTCCAGTAATCGTTTCTAATTTTAATTATTTCTACCATCGAGAGACTGACCAGGAAGGGAGACGGGCGCAAC
shaker_Frag1182-1350_insertion1287	AACTCACGCGTCTCCGTGGGCCGTCGTGACTATGACAACTGTAGGATACGGAGATATGACCCCTGTTGGAGTTTGGGGCAAAATAGTCGGAAGCCTTTGTGCTATTGCAGGCGTACTGACCATAAGCGGGAGACCGGGGTCTCTGAGCGCCTTGCCAGTTCCAGTAATCGTTTCTAATTTTAATTATTTCTACCATCGAGAGACTGACCAGGAAGGGAGACGGGCGCAAC
shaker_Frag1182-1350_insertion1290	AACTCACGCGTCTCCGTGGGCCGTCGTGACTATGACAACTGTAGGATACGGAGATATGACCCCTGTTGGAGTTTGGGGCAAAATAGTCGGAAGCCTTTGTGCTATTGCAGGCGTACTGACCATAGCCAGCGGGAGACCGGGGTCTCTGAGCTTGCCAGTTCCAGTAATCGTTTCTAATTTTAATTATTTCTACCATCGAGAGACTGACCAGGAAGGGAGACGGGCGCAAC
shaker_Frag1182-1350_insertion1293	AACTCACGCGTCTCCGTGGGCCGTCGTGACTATGACAACTGTAGGATACGGAGATATGACCCCTGTTGGAGTTTGGGGCAAAATAGTCGGAAGCCTTTGTGCTATTGCAGGCGTACTGACCATAGCCTTGAGCGGGAGACCGGGGTCTCTGAGCCCAGTTCCAGTAATCGTTTCTAATTTTAATTATTTCTACCATCGAGAGACTGACCAGGAAGGGAGACGGGCGCAAC
shaker_Frag1182-1350_insertion1296	AACTCACGCGTCTCCGTGGGCCGTCGTGACTATGACAACTGTAGGATACGGAGATATGACCCCTGTTGGAGTTTGGGGCAAAATAGTCGGAAGCCTTTGTGCTATTGCAGGCGTACTGACCATAGCCTTGCCAAGCGGGAGACCGGGGTCTCTGAGCGTTCCAGTAATCGTTTCTAATTTTAATTATTTCTACCATCGAGAGACTGACCAGGAAGGGAGACGGGCGCAAC
shaker_Frag1182-1350_insertion1299	AACTCACGCGTCTCCGTGGGCCGTCGTGACTATGACAACTGTAGGATACGGAGATATGACCCCTGTTGGAGTTTGGGGCAAAATAGTCGGAAGCCTTTGTGCTATTGCAGGCGTACTGACCATAGCCTTGCCAGTTAGCGGGAGACCGGGGTCTCTGAGCCCAGTAATCGTTTCTAATTTTAATTATTTCTACCATCGAGAGACTGACCAGGAAGGGAGACGGGCGCAAC
shaker_Frag1182-1350_insertion1302	AACTCACGCGTCTCCGTGGGCCGTCGTGACTATGACAACTGTAGGATACGGAGATATGACCCCTGTTGGAGTTTGGGGCAAAATAGTCGGAAGCCTTTGTGCTATTGCAGGCGTACTGACCATAGCCTTGCCAGTTCCAAGCGGGAGACCGGGGTCTCTGAGCGTAATCGTTTCTAATTTTAATTATTTCTACCATCGAGAGACTGACCAGGAAGGGAGACGGGCGCAAC
shaker_Frag1182-1350_insertion1305	AACTCACGCGTCTCCGTGGGCCGTCGTGACTATGACAACTGTAGGATACGGAGATATGACCCCTGTTGGAGTTTGGGGCAAAATAGTCGGAAGCCTTTGTGCTATTGCAGGCGTACTGACCATAGCCTTGCCAGTTCCAGTAAGCGGGAGACCGGGGTCTCTGAGCATCGTTTCTAATTTTAATTATTTCTACCATCGAGAGACTGACCAGGAAGGGAGACGGGCGCAAC
shaker_Frag1182-1350_insertion1308	AACTCACGCGTCTCCGTGGGCCGTCGTGACTATGACAACTGTAGGATACGGAGATATGACCCCTGTTGGAGTTTGGGGCAAAATAGTCGGAAGCCTTTGTGCTATTGCAGGCGTACTGACCATAGCCTTGCCAGTTCCAGTAATCAGCGGGAGACCGGGGTCTCTGAGCGTTTCTAATTTTAATTATTTCTACCATCGAGAGACTGACCAGGAAGGGAGACGGGCGCAAC
shaker_Frag1182-1350_insertion1311	AACTCACGCGTCTCCGTGGGCCGTCGTGACTATGACAACTGTAGGATACGGAGATATGACCCCTGTTGGAGTTTGGGGCAAAATAGTCGGAAGCCTTTGTGCTATTGCAGGCGTACTGACCATAGCCTTGCCAGTTCCAGTAATCGTTAGCGGGAGACCGGGGTCTCTGAGCTCTAATTTTAATTATTTCTACCATCGAGAGACTGACCAGGAAGGGAGACGGGCGCAAC
shaker_Frag1182-1350_insertion1314	AACTCACGCGTCTCCGTGGGCCGTCGTGACTATGACAACTGTAGGATACGGAGATATGACCCCTGTTGGAGTTTGGGGCAAAATAGTCGGAAGCCTTTGTGCTATTGCAGGCGTACTGACCATAGCCTTGCCAGTTCCAGTAATCGTTTCTAGCGGGAGACCGGGGTCTCTGAGCAATTTTAATTATTTCTACCATCGAGAGACTGACCAGGAAGGGAGACGGGCGCAAC
shaker_Frag1182-1350_insertion1317	AACTCACGCGTCTCCGTGGGCCGTCGTGACTATGACAACTGTAGGATACGGAGATATGACCCCTGTTGGAGTTTGGGGCAAAATAGTCGGAAGCCTTTGTGCTATTGCAGGCGTACTGACCATAGCCTTGCCAGTTCCAGTAATCGTTTCTAATAGCGGGAGACCGGGGTCTCTGAGCTTTAATTATTTCTACCATCGAGAGACTGACCAGGAAGGGAGACGGGCGCAAC
shaker_Frag1182-1350_insertion1320	AACTCACGCGTCTCCGTGGGCCGTCGTGACTATGACAACTGTAGGATACGGAGATATGACCCCTGTTGGAGTTTGGGGCAAAATAGTCGGAAGCCTTTGTGCTATTGCAGGCGTACTGACCATAGCCTTGCCAGTTCCAGTAATCGTTTCTAATTTTAGCGGGAGACCGGGGTCTCTGAGCAATTATTTCTACCATCGAGAGACTGACCAGGAAGGGAGACGGGCGCAAC
shaker_Frag1182-1350_insertion1323	AACTCACGCGTCTCCGTGGGCCGTCGTGACTATGACAACTGTAGGATACGGAGATATGACCCCTGTTGGAGTTTGGGGCAAAATAGTCGGAAGCCTTTGTGCTATTGCAGGCGTACTGACCATAGCCTTGCCAGTTCCAGTAATCGTTTCTAATTTTAATAGCGGGAGACCGGGGTCTCTGAGCTATTTCTACCATCGAGAGACTGACCAGGAAGGGAGACGGGCGCAAC
shaker_Frag1182-1350_insertion1326	AACTCACGCGTCTCCGTGGGCCGTCGTGACTATGACAACTGTAGGATACGGAGATATGACCCCTGTTGGAGTTTGGGGCAAAATAGTCGGAAGCCTTTGTGCTATTGCAGGCGTACTGACCATAGCCTTGCCAGTTCCAGTAATCGTTTCTAATTTTAATTATAGCGGGAGACCGGGGTCTCTGAGCTTCTACCATCGAGAGACTGACCAGGAAGGGAGACGGGCGCAAC
shaker_Frag1182-1350_insertion1329	AACTCACGCGTCTCCGTGGGCCGTCGTGACTATGACAACTGTAGGATACGGAGATATGACCCCTGTTGGAGTTTGGGGCAAAATAGTCGGAAGCCTTTGTGCTATTGCAGGCGTACTGACCATAGCCTTGCCAGTTCCAGTAATCGTTTCTAATTTTAATTATTTCAGCGGGAGACCGGGGTCTCTGAGCTACCATCGAGAGACTGACCAGGAAGGGAGACGGGCGCAAC
shaker_Frag1182-1350_insertion1332	AACTCACGCGTCTCCGTGGGCCGTCGTGACTATGACAACTGTAGGATACGGAGATATGACCCCTGTTGGAGTTTGGGGCAAAATAGTCGGAAGCCTTTGTGCTATTGCAGGCGTACTGACCATAGCCTTGCCAGTTCCAGTAATCGTTTCTAATTTTAATTATTTCTACAGCGGGAGACCGGGGTCTCTGAGCCATCGAGAGACTGACCAGGAAGGGAGACGGGCGCAAC
shaker_Frag1182-1350_insertion1335	AACTCACGCGTCTCCGTGGGCCGTCGTGACTATGACAACTGTAGGATACGGAGATATGACCCCTGTTGGAGTTTGGGGCAAAATAGTCGGAAGCCTTTGTGCTATTGCAGGCGTACTGACCATAGCCTTGCCAGTTCCAGTAATCGTTTCTAATTTTAATTATTTCTACCATAGCGGGAGACCGGGGTCTCTGAGCCGAGAGACTGACCAGGAAGGGAGACGGGCGCAAC
shaker_Frag1182-1350_insertion1338	AACTCACGCGTCTCCGTGGGCCGTCGTGACTATGACAACTGTAGGATACGGAGATATGACCCCTGTTGGAGTTTGGGGCAAAATAGTCGGAAGCCTTTGTGCTATTGCAGGCGTACTGACCATAGCCTTGCCAGTTCCAGTAATCGTTTCTAATTTTAATTATTTCTACCATCGAAGCGGGAGACCGGGGTCTCTGAGCGAGACTGACCAGGAAGGGAGACGGGCGCAAC
shaker_Frag1182-1350_insertion1341	AACTCACGCGTCTCCGTGGGCCGTCGTGACTATGACAACTGTAGGATACGGAGATATGACCCCTGTTGGAGTTTGGGGCAAAATAGTCGGAAGCCTTTGTGCTATTGCAGGCGTACTGACCATAGCCTTGCCAGTTCCAGTAATCGTTTCTAATTTTAATTATTTCTACCATCGAGAGAGCGGGAGACCGGGGTCTCTGAGCACTGACCAGGAAGGGAGACGGGCGCAAC
shaker_Frag1182-1350_insertion1344	AACTCACGCGTCTCCGTGGGCCGTCGTGACTATGACAACTGTAGGATACGGAGATATGACCCCTGTTGGAGTTTGGGGCAAAATAGTCGGAAGCCTTTGTGCTATTGCAGGCGTACTGACCATAGCCTTGCCAGTTCCAGTAATCGTTTCTAATTTTAATTATTTCTACCATCGAGAGACTAGCGGGAGACCGGGGTCTCTGAGCGACCAGGAAGGGAGACGGGCGCAAC
shaker_Frag1182-1350_insertion1347	AACTCACGCGTCTCCGTGGGCCGTCGTGACTATGACAACTGTAGGATACGGAGATATGACCCCTGTTGGAGTTTGGGGCAAAATAGTCGGAAGCCTTTGTGCTATTGCAGGCGTACTGACCATAGCCTTGCCAGTTCCAGTAATCGTTTCTAATTTTAATTATTTCTACCATCGAGAGACTGACAGCGGGAGACCGGGGTCTCTGAGCCAGGAAGGGAGACGGGCGCAAC
shaker_Frag1182-1350_insertion1350	AACTCACGCGTCTCCGTGGGCCGTCGTGACTATGACAACTGTAGGATACGGAGATATGACCCCTGTTGGAGTTTGGGGCAAAATAGTCGGAAGCCTTTGTGCTATTGCAGGCGTACTGACCATAGCCTTGCCAGTTCCAGTAATCGTTTCTAATTTTAATTATTTCTACCATCGAGAGACTGACCAGAGCGGGAGACCGGGGTCTCTGAGCGAAGGGAGACGGGCGCAAC
shaker_Frag1353-1521_insertion1353	GCTTAGGACGTCTCGGAAAGCGGGAGACCGGGGTCTCTGAGCGAAATGCAGTCAGAACTTCAACATGTCACTAGCTGCCCTATCTTCCAGCACTCTCGTAGGTCAGCACATGAAGAAGTCATCCTTGTCTGAATCTAGCAGTGACATGATGGATTTGGACGACGGTGTTGAAAGTACCCTGGACTGACAGAACACACCCTGGTCGGTGGAGACGGAGATACT
shaker_Frag1353-1521_insertion1356	GCTTAGGACGTCTCGGAAGAAAGCGGGAGACCGGGGTCTCTGAGCATGCAGTCAGAACTTCAACATGTCACTAGCTGCCCTATCTTCCAGCACTCTCGTAGGTCAGCACATGAAGAAGTCATCCTTGTCTGAATCTAGCAGTGACATGATGGATTTGGACGACGGTGTTGAAAGTACCCTGGACTGACAGAACACACCCTGGTCGGTGGAGACGGAGATACT
shaker_Frag1353-1521_insertion1359	GCTTAGGACGTCTCGGAAGAAATGAGCGGGAGACCGGGGTCTCTGAGCCAGTCAGAACTTCAACATGTCACTAGCTGCCCTATCTTCCAGCACTCTCGTAGGTCAGCACATGAAGAAGTCATCCTTGTCTGAATCTAGCAGTGACATGATGGATTTGGACGACGGTGTTGAAAGTACCCTGGACTGACAGAACACACCCTGGTCGGTGGAGACGGAGATACT
shaker_Frag1353-1521_insertion1362	GCTTAGGACGTCTCGGAAGAAATGCAGAGCGGGAGACCGGGGTCTCTGAGCTCAGAACTTCAACATGTCACTAGCTGCCCTATCTTCCAGCACTCTCGTAGGTCAGCACATGAAGAAGTCATCCTTGTCTGAATCTAGCAGTGACATGATGGATTTGGACGACGGTGTTGAAAGTACCCTGGACTGACAGAACACACCCTGGTCGGTGGAGACGGAGATACT
shaker_Frag1353-1521_insertion1365	GCTTAGGACGTCTCGGAAGAAATGCAGTCAAGCGGGAGACCGGGGTCTCTGAGCGAACTTCAACATGTCACTAGCTGCCCTATCTTCCAGCACTCTCGTAGGTCAGCACATGAAGAAGTCATCCTTGTCTGAATCTAGCAGTGACATGATGGATTTGGACGACGGTGTTGAAAGTACCCTGGACTGACAGAACACACCCTGGTCGGTGGAGACGGAGATACT
shaker_Frag1353-1521_insertion1368	GCTTAGGACGTCTCGGAAGAAATGCAGTCAGAAAGCGGGAGACCGGGGTCTCTGAGCCTTCAACATGTCACTAGCTGCCCTATCTTCCAGCACTCTCGTAGGTCAGCACATGAAGAAGTCATCCTTGTCTGAATCTAGCAGTGACATGATGGATTTGGACGACGGTGTTGAAAGTACCCTGGACTGACAGAACACACCCTGGTCGGTGGAGACGGAGATACT
shaker_Frag1353-1521_insertion1371	GCTTAGGACGTCTCGGAAGAAATGCAGTCAGAACTTAGCGGGAGACCGGGGTCTCTGAGCCAACATGTCACTAGCTGCCCTATCTTCCAGCACTCTCGTAGGTCAGCACATGAAGAAGTCATCCTTGTCTGAATCTAGCAGTGACATGATGGATTTGGACGACGGTGTTGAAAGTACCCTGGACTGACAGAACACACCCTGGTCGGTGGAGACGGAGATACT

shaker_Frag1353-1521_insertion1374	GCTTAGGACGTCTCCGGAAGAAATGCAGAGTCAGAACTTCAGCGGGAGACCGGGGTCTCTGAGCAACCATGTCACTAGCTGCCCTTATCTTCCAGGCACTCTCGTAGGTCAGCACATGAAGAAGTCATCCTTGTCTGAATCTAGCAGTGACATGATGGATTTGGACGACGGTGTTGAAAGTACCCCTGGACTGACAGAAACACACCCTGGTCGGTGGAGACGGAGATACT
shaker_Frag1353-1521_insertion1377	GCTTAGGACGTCTCCGGAAGAAATGCAGAGTCAGAACTTCAACAGCGGGAGACCGGGGTCTCTGAGCCATGTCACTAGCTGCCCTTATCTTCCAGGCACTCTCGTAGGTCAGCACATGAAGAAGTCATCCTTGTCTGAATCTAGCAGTGACATGATGGATTTGGACGACGGTGTTGAAAGTACCCCTGGACTGACAGAAACACACCCTGGTCGGTGGAGACGGAGATACT
shaker_Frag1353-1521_insertion1380	GCTTAGGACGTCTCCGGAAGAAATGCAGAGTCAGAACTTCAACCATAGCGGGAGACCGGGGTCTCTGAGCGTCACTAGCTGCCCTTATCTTCCAGGCACTCTCGTAGGTCAGCACATGAAGAAGTCATCCTTGTCTGAATCTAGCAGTGACATGATGGATTTGGACGACGGTGTTGAAAGTACCCCTGGACTGACAGAAACACACCCTGGTCGGTGGAGACGGAGATACT
shaker_Frag1353-1521_insertion1383	GCTTAGGACGTCTCCGGAAGAAATGCAGAGTCAGAACTTCAACCATGTCAGCGGGAGACCGGGGTCTCTGAGCACTAGCTGCCCTTATCTTCCAGGCACTCTCGTAGGTCAGCACATGAAGAAGTCATCCTTGTCTGAATCTAGCAGTGACATGATGGATTTGGACGACGGTGTTGAAAGTACCCCTGGACTGACAGAAACACACCCTGGTCGGTGGAGACGGAGATACT
shaker_Frag1353-1521_insertion1386	GCTTAGGACGTCTCCGGAAGAAATGCAGAGTCAGAACTTCAACCATGTCACTAGCGGGAGACCGGGGTCTCTGAGCAGCTGCCCTTATCTTCCAGGCACTCTCGTAGGTCAGCACATGAAGAAGTCATCCTTGTCTGAATCTAGCAGTGACATGATGGATTTGGACGACGGTGTTGAAAGTACCCCTGGACTGACAGAAACACACCCTGGTCGGTGGAGACGGAGATACT
shaker_Frag1353-1521_insertion1389	GCTTAGGACGTCTCCGGAAGAAATGCAGAGTCAGAACTTCAACCATGTCACTAGCAGCGGGAGACCGGGGTCTCTGAGCTGCCCTTATCTTCCAGGCACTCTCGTAGGTCAGCACATGAAGAAGTCATCCTTGTCTGAATCTAGCAGTGACATGATGGATTTGGACGACGGTGTTGAAAGTACCCCTGGACTGACAGAAACACACCCTGGTCGGTGGAGACGGAGATACT
shaker_Frag1353-1521_insertion1392	GCTTAGGACGTCTCCGGAAGAAATGCAGAGTCAGAACTTCAACCATGTCACTAGCTGCAGCGGGAGACCGGGGTCTCTGAGCCCTTATCTTCCAGGCACTCTCGTAGGTCAGCACATGAAGAAGTCATCCTTGTCTGAATCTAGCAGTGACATGATGGATTTGGACGACGGTGTTGAAAGTACCCCTGGACTGACAGAAACACACCCTGGTCGGTGGAGACGGAGATACT
shaker_Frag1353-1521_insertion1395	GCTTAGGACGTCTCCGGAAGAAATGCAGAGTCAGAACTTCAACCATGTCACTAGCTGCCCTAGCGGGAGACCGGGGTCTCTGAGCTATCTTCCAGGCACTCTCGTAGGTCAGCACATGAAGAAGTCATCCTTGTCTGAATCTAGCAGTGACATGATGGATTTGGACGACGGTGTTGAAAGTACCCCTGGACTGACAGAAACACACCCTGGTCGGTGGAGACGGAGATACT
shaker_Frag1353-1521_insertion1398	GCTTAGGACGTCTCCGGAAGAAATGCAGAGTCAGAACTTCAACCATGTCACTAGCTGCCCTTATAGCGGGAGACCGGGGTCTCTGAGCCTTCCAGGCACTCTCGTAGGTCAGCACATGAAGAAGTCATCCTTGTCTGAATCTAGCAGTGACATGATGGATTTGGACGACGGTGTTGAAAGTACCCCTGGACTGACAGAAACACACCCTGGTCGGTGGAGACGGAGATACT
shaker_Frag1353-1521_insertion1401	GCTTAGGACGTCTCCGGAAGAAATGCAGAGTCAGAACTTCAACCATGTCACTAGCTGCCCTTATCTTAGCGGGAGACCGGGGTCTCTGAGCCCAGGCACTCTCGTAGGTCAGCACATGAAGAAGTCATCCTTGTCTGAATCTAGCAGTGACATGATGGATTTGGACGACGGTGTTGAAAGTACCCCTGGACTGACAGAAACACACCCTGGTCGGTGGAGACGGAGATACT
shaker_Frag1353-1521_insertion1404	GCTTAGGACGTCTCCGGAAGAAATGCAGAGTCAGAACTTCAACCATGTCACTAGCTGCCCTTATCTTCCAAGCGGGAGACCGGGGTCTCTGAGCGGCACTCTCGTAGGTCAGCACATGAAGAAGTCATCCTTGTCTGAATCTAGCAGTGACATGATGGATTTGGACGACGGTGTTGAAAGTACCCCTGGACTGACAGAAACACACCCTGGTCGGTGGAGACGGAGATACT
shaker_Frag1353-1521_insertion1407	GCTTAGGACGTCTCCGGAAGAAATGCAGAGTCAGAACTTCAACCATGTCACTAGCTGCCCTTATCTTCCAGGCAGCGGGAGACCGGGGTCTCTGAGCACTCTCGTAGGTCAGCACATGAAGAAGTCATCCTTGTCTGAATCTAGCAGTGACATGATGGATTTGGACGACGGTGTTGAAAGTACCCCTGGACTGACAGAAACACACCCTGGTCGGTGGAGACGGAGATACT
shaker_Frag1353-1521_insertion1410	GCTTAGGACGTCTCCGGAAGAAATGCAGAGTCAGAACTTCAACCATGTCACTAGCTGCCCTTATCTTCCAGGCACTAGCGGGAGACCGGGGTCTCTGAGCCTCGTAGGTCAGCACATGAAGAAGTCATCCTTGTCTGAATCTAGCAGTGACATGATGGATTTGGACGACGGTGTTGAAAGTACCCCTGGACTGACAGAAACACACCCTGGTCGGTGGAGACGGAGATACT
shaker_Frag1353-1521_insertion1413	GCTTAGGACGTCTCCGGAAGAAATGCAGAGTCAGAACTTCAACCATGTCACTAGCTGCCCTTATCTTCCAGGCACTCTCAGCGGGAGACCGGGGTCTCTGAGCGTAGGTCAGCACATGAAGAAGTCATCCTTGTCTGAATCTAGCAGTGACATGATGGATTTGGACGACGGTGTTGAAAGTACCCCTGGACTGACAGAAACACACCCTGGTCGGTGGAGACGGAGATACT
shaker_Frag1353-1521_insertion1416	GCTTAGGACGTCTCCGGAAGAAATGCAGAGTCAGAACTTCAACCATGTCACTAGCTGCCCTTATCTTCCAGGCACTCTCGTAAGCGGGAGACCGGGGTCTCTGAGCGGTCAGCACATGAAGAAGTCATCCTTGTCTGAATCTAGCAGTGACATGATGGATTTGGACGACGGTGTTGAAAGTACCCCTGGACTGACAGAAACACACCCTGGTCGGTGGAGACGGAGATACT
shaker_Frag1353-1521_insertion1419	GCTTAGGACGTCTCCGGAAGAAATGCAGAGTCAGAACTTCAACCATGTCACTAGCTGCCCTTATCTTCCAGGCACTCTCGTAGGTAGCGGGAGACCGGGGTCTCTGAGCCAGCACATGAAGAAGTCATCCTTGTCTGAATCTAGCAGTGACATGATGGATTTGGACGACGGTGTTGAAAGTACCCCTGGACTGACAGAAACACACCCTGGTCGGTGGAGACGGAGATACT
shaker_Frag1353-1521_insertion1422	GCTTAGGACGTCTCCGGAAGAAATGCAGAGTCAGAACTTCAACCATGTCACTAGCTGCCCTTATCTTCCAGGCACTCTCGTAGGTCAGAGCGGGAGACCGGGGTCTCTGAGCCACATGAAGAAGTCATCCTTGTCTGAATCTAGCAGTGACATGATGGATTTGGACGACGGTGTTGAAAGTACCCCTGGACTGACAGAAACACACCCTGGTCGGTGGAGACGGAGATACT
shaker_Frag1353-1521_insertion1425	GCTTAGGACGTCTCCGGAAGAAATGCAGAGTCAGAACTTCAACCATGTCACTAGCTGCCCTTATCTTCCAGGCACTCTCGTAGGTCAGCACAGCGGGAGACCGGGGTCTCTGAGCATGAAGAAGTCATCCTTGTCTGAATCTAGCAGTGACATGATGGATTTGGACGACGGTGTTGAAAGTACCCCTGGACTGACAGAAACACACCCTGGTCGGTGGAGACGGAGATACT
shaker_Frag1353-1521_insertion1428	GCTTAGGACGTCTCCGGAAGAAATGCAGAGTCAGAACTTCAACCATGTCACTAGCTGCCCTTATCTTCCAGGCACTCTCGTAGGTCAGCACATGAGCGGGAGACCGGGGTCTCTGAGCAAGAAGTCATCCTTGTCTGAATCTAGCAGTGACATGATGGATTTGGACGACGGTGTTGAAAGTACCCCTGGACTGACAGAAACACACCCTGGTCGGTGGAGACGGAGATACT
shaker_Frag1353-1521_insertion1431	GCTTAGGACGTCTCCGGAAGAAATGCAGAGTCAGAACTTCAACCATGTCACTAGCTGCCCTTATCTTCCAGGCACTCTCGTAGGTCAGCACATGAAGAGCGGGAGACCGGGGTCTCTGAGCAAGTCATCCTTGTCTGAATCTAGCAGTGACATGATGGATTTGGACGACGGTGTTGAAAGTACCCCTGGACTGACAGAAACACACCCTGGTCGGTGGAGACGGAGATACT
shaker_Frag1353-1521_insertion1434	GCTTAGGACGTCTCCGGAAGAAATGCAGAGTCAGAACTTCAACCATGTCACTAGCTGCCCTTATCTTCCAGGCACTCTCGTAGGTCAGCACATGAAGAAGAGCGGGAGACCGGGGTCTCTGAGCTCATCCTTGTCTGAATCTAGCAGTGACATGATGGATTTGGACGACGGTGTTGAAAGTACCCCTGGACTGACAGAAACACACCCTGGTCGGTGGAGACGGAGATACT
shaker_Frag1353-1521_insertion1437	GCTTAGGACGTCTCCGGAAGAAATGCAGAGTCAGAACTTCAACCATGTCACTAGCTGCCCTTATCTTCCAGGCACTCTCGTAGGTCAGCACATGAAGAAGTCAAGCGGGAGACCGGGGTCTCTGAGCTCCTTGTCTGAATCTAGCAGTGACATGATGGATTTGGACGACGGTGTTGAAAGTACCCCTGGACTGACAGAAACACACCCTGGTCGGTGGAGACGGAGATACT
shaker_Frag1353-1521_insertion1440	GCTTAGGACGTCTCCGGAAGAAATGCAGAGTCAGAACTTCAACCATGTCACTAGCTGCCCTTATCTTCCAGGCACTCTCGTAGGTCAGCACATGAAGAAGTCATCCAGCGGGAGACCGGGGTCTCTGAGCTTGTCTGAATCTAGCAGTGACATGATGGATTTGGACGACGGTGTTGAAAGTACCCCTGGACTGACAGAAACACACCCTGGTCGGTGGAGACGGAGATACT
shaker_Frag1353-1521_insertion1443	GCTTAGGACGTCTCCGGAAGAAATGCAGAGTCAGAACTTCAACCATGTCACTAGCTGCCCTTATCTTCCAGGCACTCTCGTAGGTCAGCACATGAAGAAGTCATCCTTGAGCGGGAGACCGGGGTCTCTGAGCTCTGAATCTAGCAGTGACATGATGGATTTGGACGACGGTGTTGAAAGTACCCCTGGACTGACAGAAACACACCCTGGTCGGTGGAGACGGAGATACT
shaker_Frag1353-1521_insertion1446	GCTTAGGACGTCTCCGGAAGAAATGCAGAGTCAGAACTTCAACCATGTCACTAGCTGCCCTTATCTTCCAGGCACTCTCGTAGGTCAGCACATGAAGAAGTCATCCTTGTCTAGCGGGAGACCGGGGTCTCTGAGCGAATCTAGCAGTGACATGATGGATTTGGACGACGGTGTTGAAAGTACCCCTGGACTGACAGAAACACACCCTGGTCGGTGGAGACGGAGATACT
shaker_Frag1353-1521_insertion1449	GCTTAGGACGTCTCCGGAAGAAATGCAGAGTCAGAACTTCAACCATGTCACTAGCTGCCCTTATCTTCCAGGCACTCTCGTAGGTCAGCACATGAAGAAGTCATCCTTGTCTGAAAGCGGGAGACCGGGGTCTCTGAGCTCTAGCAGTGACATGATGGATTTGGACGACGGTGTTGAAAGTACCCCTGGACTGACAGAAACACACCCTGGTCGGTGGAGACGGAGATACT
shaker_Frag1353-1521_insertion1452	GCTTAGGACGTCTCCGGAAGAAATGCAGAGTCAGAACTTCAACCATGTCACTAGCTGCCCTTATCTTCCAGGCACTCTCGTAGGTCAGCACATGAAGAAGTCATCCTTGTCTGAATCTAGCGGGAGACCGGGGTCTCTGAGCAGCAGTGACATGATGGATTTGGACGACGGTGTTGAAAGTACCCCTGGACTGACAGAAACACACCCTGGTCGGTGGAGACGGAGATACT
shaker_Frag1353-1521_insertion1455	GCTTAGGACGTCTCCGGAAGAAATGCAGAGTCAGAACTTCAACCATGTCACTAGCTGCCCTTATCTTCCAGGCACTCTCGTAGGTCAGCACATGAAGAAGTCATCCTTGTCTGAATCTAGCAGCGGGAGACCGGGGTCTCTGAGCAGTGACATGATGGATTTGGACGACGGTGTTGAAAGTACCCCTGGACTGACAGAAACACACCCTGGTCGGTGGAGACGGAGATACT
shaker_Frag1353-1521_insertion1458	GCTTAGGACGTCTCCGGAAGAAATGCAGAGTCAGAACTTCAACCATGTCACTAGCTGCCCTTATCTTCCAGGCACTCTCGTAGGTCAGCACATGAAGAAGTCATCCTTGTCTGAATCTAGCAGTAGCGGGAGACCGGGGTCTCTGAGCGACATGATGGATTTGGACGACGGTGTTGAAAGTACCCCTGGACTGACAGAAACACACCCTGGTCGGTGGAGACGGAGATACT
shaker_Frag1353-1521_insertion1461	GCTTAGGACGTCTCCGGAAGAAATGCAGAGTCAGAACTTCAACCATGTCACTAGCTGCCCTTATCTTCCAGGCACTCTCGTAGGTCAGCACATGAAGAAGTCATCCTTGTCTGAATCTAGCAGTGACAGCGGGAGACCGGGGTCTCTGAGCATGATGGATTTGGACGACGGTGTTGAAAGTACCCCTGGACTGACAGAAACACACCCTGGTCGGTGGAGACGGAGATACT
shaker_Frag1353-1521_insertion1464	GCTTAGGACGTCTCCGGAAGAAATGCAGAGTCAGAACTTCAACCATGTCACTAGCTGCCCTTATCTTCCAGGCACTCTCGTAGGTCAGCACATGAAGAAGTCATCCTTGTCTGAATCTAGCAGTGACATGAGCGGGAGACCGGGGTCTCTGAGCATGGATTTGGACGACGGTGTTGAAAGTACCCCTGGACTGACAGAAACACACCCTGGTCGGTGGAGACGGAGATACT
shaker_Frag1353-1521_insertion1467	GCTTAGGACGTCTCCGGAAGAAATGCAGAGTCAGAACTTCAACCATGTCACTAGCTGCCCTTATCTTCCAGGCACTCTCGTAGGTCAGCACATGAAGAAGTCATCCTTGTCTGAATCTAGCAGTGACATGATGAGCGGGAGACCGGGGTCTCTGAGCGATTTGGACGACGGTGTTGAAAGTACCCCTGGACTGACAGAAACACACCCTGGTCGGTGGAGACGGAGATACT
shaker_Frag1353-1521_insertion1470	GCTTAGGACGTCTCCGGAAGAAATGCAGAGTCAGAACTTCAACCATGTCACTAGCTGCCCTTATCTTCCAGGCACTCTCGTAGGTCAGCACATGAAGAAGTCATCCTTGTCTGAATCTAGCAGTGACATGATGGATAGCGGGAGACCGGGGTCTCTGAGCTTGGACGACGGTGTTGAAAGTACCCCTGGACTGACAGAAACACACCCTGGTCGGTGGAGACGGAGATACT
shaker_Frag1353-1521_insertion1473	GCTTAGGACGTCTCCGGAAGAAATGCAGAGTCAGAACTTCAACCATGTCACTAGCTGCCCTTATCTTCCAGGCACTCTCGTAGGTCAGCACATGAAGAAGTCATCCTTGTCTGAATCTAGCAGTGACATGATGGATTTGAGCGGGAGACCGGGGTCTCTGAGCGACGACGGTGTTGAAAGTACCCCTGGACTGACAGAAACACACCCTGGTCGGTGGAGACGGAGATACT
shaker_Frag1353-1521_insertion1476	GCTTAGGACGTCTCCGGAAGAAATGCAGAGTCAGAACTTCAACCATGTCACTAGCTGCCCTTATCTTCCAGGCACTCTCGTAGGTCAGCACATGAAGAAGTCATCCTTGTCTGAATCTAGCAGTGACATGATGGATTTGGACAGCGGGAGACCGGGGTCTCTGAGCGACGGTGTTGAAAGTACCCCTGGACTGACAGAAACACACCCTGGTCGGTGGAGACGGAGATACT
shaker_Frag1353-1521_insertion1479	GCTTAGGACGTCTCCGGAAGAAATGCAGAGTCAGAACTTCAACCATGTCACTAGCTGCCCTTATCTTCCAGGCACTCTCGTAGGTCAGCACATGAAGAAGTCATCCTTGTCTGAATCTAGCAGTGACATGATGGATTTGGACGACAGCGGGAGACCGGGGTCTCTGAGCGGTGTTGAAAGTACCCCTGGACTGACAGAAACACACCCTGGTCGGTGGAGACGGAGATACT
shaker_Frag1353-1521_insertion1482	GCTTAGGACGTCTCCGGAAGAAATGCAGAGTCAGAACTTCAACCATGTCACTAGCTGCCCTTATCTTCCAGGCACTCTCGTAGGTCAGCACATGAAGAAGTCATCCTTGTCTGAATCTAGCAGTGACATGATGGATTTGGACGACGGTAGCGGGAGACCGGGGTCTCTGAGCGTTGAAAGTACCCCTGGACTGACAGAAACACACCCTGGTCGGTGGAGACGGAGATACT
shaker_Frag1353-1521_insertion1485	GCTTAGGACGTCTCCGGAAGAAATGCAGAGTCAGAACTTCAACCATGTCACTAGCTGCCCTTATCTTCCAGGCACTCTCGTAGGTCAGCACATGAAGAAGTCATCCTTGTCTGAATCTAGCAGTGACATGATGGATTTGGACGACGGTGTTAGCGGGAGACCGGGGTCTCTGAGCGAAAGTACCCCTGGACTGACAGAAACACACCCTGGTCGGTGGAGACGGAGATACT
shaker_Frag1353-1521_insertion1488	GCTTAGGACGTCTCCGGAAGAAATGCAGAGTCAGAACTTCAACCATGTCACTAGCTGCCCTTATCTTCCAGGCACTCTCGTAGGTCAGCACATGAAGAAGTCATCCTTGTCTGAATCTAGCAGTGACATGATGGATTTGGACGACGGTGTTGAAAGCGGGAGACCGGGGTCTCTGAGCAGTACCCCTGGACTGACAGAAACACACCCTGGTCGGTGGAGACGGAGATACT
shaker_Frag1353-1521_insertion1491	GCTTAGGACGTCTCCGGAAGAAATGCAGAGTCAGAACTTCAACCATGTCACTAGCTGCCCTTATCTTCCAGGCACTCTCGTAGGTCAGCACATGAAGAAGTCATCCTTGTCTGAATCTAGCAGTGACATGATGGATTTGGACGACGGTGTTGAAAGTAGCGGGAGACCGGGGTCTCTGAGCACCCCTGGACTGACAGAAACACACCCTGGTCGGTGGAGACGGAGATACT
shaker_Frag1353-1521_insertion1494	GCTTAGGACGTCTCCGGAAGAAATGCAGAGTCAGAACTTCAACCATGTCACTAGCTGCCCTTATCTTCCAGGCACTCTCGTAGGTCAGCACATGAAGAAGTCATCCTTGTCTGAATCTAGCAGTGACATGATGGATTTGGACGACGGTGTTGAAAGTACCAGCGGGAGACCGGGGTCTCTGAGCCCTGGACTGACAGAAACACACCCTGGTCGGTGGAGACGGAGATACT
shaker_Frag1353-1521_insertion1497	GCTTAGGACGTCTCCGGAAGAAATGCAGAGTCAGAACTTCAACCATGTCACTAGCTGCCCTTATCTTCCAGGCACTCTCGTAGGTCAGCACATGAAGAAGTCATCCTTGTCTGAATCTAGCAGTGACATGATGGATTTGGACGACGGTGTTGAAAGTACCCCTAGCGGGAGACCGGGGTCTCTGAGCGGACTGACAGAAACACACCCTGGTCGGTGGAGACGGAGATACT
shaker_Frag1353-1521_insertion1500	GCTTAGGACGTCTCCGGAAGAAATGCAGAGTCAGAACTTCAACCATGTCACTAGCTGCCCTTATCTTCCAGGCACTCTCGTAGGTCAGCACATGAAGAAGTCATCCTTGTCTGAATCTAGCAGTGACATGATGGATTTGGACGACGGTGTTGAAAGTACCCCTGGAAGCGGGAGACCGGGGTCTCTGAGCCTGACAGAAACACACCCTGGTCGGTGGAGACGGAGATACT
shaker_Frag1353-1521_insertion1503	GCTTAGGACGTCTCCGGAAGAAATGCAGAGTCAGAACTTCAACCATGTCACTAGCTGCCCTTATCTTCCAGGCACTCTCGTAGGTCAGCACATGAAGAAGTCATCCTTGTCTGAATCTAGCAGTGACATGATGGATTTGGACGACGGTGTTGAAAGTACCCCTGGACTGAGCGGGAGACCGGGGTCTCTGAGCACAGAAACACACCCTGGTCGGTGGAGACGGAGATACT
shaker_Frag1353-1521_insertion1506	GCTTAGGACGTCTCCGGAAGAAATGCAGAGTCAGAACTTCAACCATGTCACTAGCTGCCCTTATCTTCCAGGCACTCTCGTAGGTCAGCACATGAAGAAGTCATCCTTGTCTGAATCTAGCAGTGACATGATGGATTTGGACGACGGTGTTGAAAGTACCCCTGGACTGACAAGCGGGAGACCGGGGTCTCTGAGCGAAACACACCCTGGTCGGTGGAGACGGAGATACT
shaker_Frag1353-1521_insertion1509	GCTTAGGACGTCTCCGGAAGAAATGCAGAGTCAGAACTTCAACCATGTCACTAGCTGCCCTTATCTTCCAGGCACTCTCGTAGGTCAGCACATGAAGAAGTCATCCTTGTCTGAATCTAGCAGTGACATGATGGATTTGGACGACGGTGTTGAAAGTACCCCTGGACTGACAGAAAGCGGGAGACCGGGGTCTCTGAGCACACACCCTGGTCGGTGGAGACGGAGATACT
shaker_Frag1353-1521_insertion1512	GCTTAGGACGTCTCCGGAAGAAATGCAGAGTCAGAACTTCAACCATGTCACTAGCTGCCCTTATCTTCCAGGCACTCTCGTAGGTCAGCACATGAAGAAGTCATCCTTGTCTGAATCTAGCAGTGACATGATGGATTTGGACGACGGTGTTGAAAGTACCCCTGGACTGACAGAAACAAGCGGGAGACCGGGGTCTCTGAGCCACCCTGGTCGGTGGAGACGGAGATACT
shaker_Frag1353-1521_insertion1515	GCTTAGGACGTCTCCGGAAGAAATGCAGAGTCAGAACTTCAACCATGTCACTAGCTGCCCTTATCTTCCAGGCACTCTCGTAGGTCAGCACATGAAGAAGTCATCCTTGTCTGAATCTAGCAGTGACATGATGGATTTGGACGACGGTGTTGAAAGTACCCCTGGACTGACAGAAACACACAGCGGGAGACCGGGGTCTCTGAGCCCTGGTCGGTGGAGACGGAGATACT
shaker_Frag1353-1521_insertion1518	GCTTAGGACGTCTCCGGAAGAAATGCAGAGTCAGAACTTCAACCATGTCACTAGCTGCCCTTATCTTCCAGGCACTCTCGTAGGTCAGCACATGAAGAAGTCATCCTTGTCTGAATCTAGCAGTGACATGATGGATTTGGACGACGGTGTTGAAAGTACCCCTGGACTGACAGAAACACACCCTAGCGGGAGACCGGGGTCTCTGAGCGGTCGGTGGAGACGGAGATACT
shaker_Frag1353-1521_insertion1521	GCTTAGGACGTCTCCGGAAGAAATGCAGAGTCAGAACTTCAACCATGTCACTAGCTGCCCTTATCTTCCAGGCACTCTCGTAGGTCAGCACATGAAGAAGTCATCCTTGTCTGAATCTAGCAGTGACATGATGGATTTGGACGACGGTGTTGAAAGTACCCCTGGACTGACAGAAACACACCCTGGTAGCGGGAGACCGGGGTCTCTGAGCCGGTGGAGACGGAGATACT
shaker_Frag1524-1692_insertion1524	GCTTTTCTCGTCTCCTCGGAGCGGGAGACCGGGGTCTCTGAGCTCTGCAGTGGCCCCCTTCCTGGGTGCTCAACAACAGCAGCAACAACCCGTGGCCAGTTCATTGTCAATGTCAATAGATAAGCAGCTTCAACACCCTTTGCAACAATTGACACAAACTCAGCTTTATCAACAACAGCAACAACAGCAACAGCAACAACAGAACGGTTTTAAGCGGAGACGGGATGCTA
shaker_Frag1524-1692_insertion1527	GCTTTTCTCGTCTCCTCGGTCTAGCGGGAGACCGGGGTCTCTGAGCGCAGTGGCCCCCTTCCTGGGTGCTCAACAACAGCAGCAACAACCCGTGGCCAGTTCATTGTCAATGTCAATAGATAAGCAGCTTCAACACCCTTTGCAACAATTGACACAAACTCAGCTTTATCAACAACAGCAACAACAGCAACAGCAACAACAGAACGGTTTTAAGCGGAGACGGGATGCTA
shaker_Frag1524-1692_insertion1530	GCTTTTCTCGTCTCCTCGGTCTGCAAGCGGGAGACCGGGGTCTCTGAGCGTGGCCCCCTTCCTGGGTGCTCAACAACAGCAGCAACAACCCGTGGCCAGTTCATTGTCAATGTCAATAGATAAGCAGCTTCAACACCCTTTGCAACAATTGACACAAACTCAGCTTTATCAACAACAGCAACAACAGCAACAGCAACAACAGAACGGTTTTAAGCGGAGACGGGATGCTA
shaker_Frag1524-1692_insertion1533	GCTTTTCTCGTCTCCTCGGTCTGCAGTGAGCGGGAGACCGGGGTCTCTGAGCGCCCCCTTCCTGGGTGCTCAACAACAGCAGCAACAACCCGTGGCCAGTTCATTGTCAATGTCAATAGATAAGCAGCTTCAACACCCTTTGCAACAATTGACACAAACTCAGCTTTATCAACAACAGCAACAACAGCAACAGCAACAACAGAACGGTTTTAAGCGGAGACGGGATGCTA
shaker_Frag1524-1692_insertion1536	GCTTTTCTCGTCTCCTCGGTCTGCAGTGGCCAGCGGGAGACCGGGGTCTCTGAGCCCCTTCCTGGGTGCTCAACAACAGCAGCAACAACCCGTGGCCAGTTCATTGTCAATGTCAATAGATAAGCAGCTTCAACACCCTTTGCAACAATTGACACAAACTCAGCTTTATCAACAACAGCAACAACAGCAACAGCAACAACAGAACGGTTTTAAGCGGAGACGGGATGCTA
shaker_Frag1524-1692_insertion1539	GCTTTTCTCGTCTCCTCGGTCTGCAGTGGCCCCCAGCGGGAGACCGGGGTCTCTGAGCTTCCTGGGTGCTCAACAACAGCAGCAACAACCCGTGGCCAGTTCATTGTCAATGTCAATAGATAAGCAGCTTCAACACCCTTTGCAACAATTGACACAAACTCAGCTTTATCAACAACAGCAACAACAGCAACAGCAACAACAGAACGGTTTTAAGCGGAGACGGGATGCTA
shaker_Frag1524-1692_insertion1542	GCTTTTCTCGTCTCCTCGGTCTGCAGTGGCCCCCTTCAGCGGGAGACCGGGGTCTCTGAGCCTGGGTGCTCAACAACAGCAGCAACAACCCGTGGCCAGTTCATTGTCAATGTCAATAGATAAGCAGCTTCAACACCCTTTGCAACAATTGACACAAACTCAGCTTTATCAACAACAGCAACAACAGCAACAGCAACAACAGAACGGTTTTAAGCGGAGACGGGATGCTA
shaker_Frag1524-1692_insertion1545	GCTTTTCTCGTCTCCTCGGTCTGCAGTGGCCCCCTTCCTGAGCGGGAGACCGGGGTCTCTGAGCGGTGCTCAACAACAGCAGCAACAACCCGTGGCCAGTTCATTGTCAATGTCAATAGATAAGCAGCTTCAACACCCTTTGCAACAATTGACACAAACTCAGCTTTATCAACAACAGCAACAACAGCAACAGCAACAACAGAACGGTTTTAAGCGGAGACGGGATGCTA
shaker_Frag1524-1692_insertion1548	GCTTTTCTCGTCTCCTCGGTCTGCAGTGGCCCCCTTCCTGGGTAGCGGGAGACCGGGGTCTCTGAGCGCTCAACAACAGCAGCAACAACCCGTGGCCAGTTCATTGTCAATGTCAATAGATAAGCAGCTTCAACACCCTTTGCAACAATTGACACAAACTCAGCTTTATCAACAACAGCAACAACAGCAACAGCAACAACAGAACGGTTTTAAGCGGAGACGGGATGCTA
shaker_Frag1524-1692_insertion1551	GCTTTTCTCGTCTCCTCGGTCTGCAGTGGCCCCCTTCCTGGGTGCTAGCGGGAGACCGGGGTCTCTGAGCCAACAACAGCAGCAACAACCCGTGGCCAGTTCATTGTCAATGTCAATAGATAAGCAGCTTCAACACCCTTTGCAACAATTGACACAAACTCAGCTTTATCAACAACAGCAACAACAGCAACAGCAACAACAGAACGGTTTTAAGCGGAGACGGGATGCTA
shaker_Frag1524-1692_insertion1554	GCTTTTCTCGTCTCCTCGGTCTGCAGTGGCCCCCTTCCTGGGTGCTCAAAGCGGGAGACCGGGGTCTCTGAGCCAACAGCAGCAACAACCCGTGGCCAGTTCATTGTCAATGTCAATAGATAAGCAGCTTCAACACCCTTTGCAACAATTGACACAAACTCAGCTTTATCAACAACAGCAACAACAGCAACAGCAACAACAGAACGGTTTTAAGCGGAGACGGGATGCTA
shaker_Frag1524-1692_insertion1557	GCTTTTCTCGTCTCCTCGGTCTGCAGTGGCCCCCTTCCTGGGTGCTCAACAAAGCGGGAGACCGGGGTCTCTGAGCCAGCAGCAACAACCCGTGGCCAGTTCATTGTCAATGTCAATAGATAAGCAGCTTCAACACCCTTTGCAACAATTGACACAAACTCAGCTTTATCAACAACAGCAACAACAGCAACAGCAACAACAGAACGGTTTTAAGCGGAGACGGGATGCTA
shaker_Frag1524-1692_insertion1560	GCTTTTCTCGTCTCCTCGGTCTGCAGTGGCCCCCTTCCTGGGTGCTCAACAACAGAGCGGGAGACCGGGGTCTCTGAGCCAGCAACAACCCGTGGCCAGTTCATTGTCAATGTCAATAGATAAGCAGCTTCAACACCCTTTGCAACAATTGACACAAACTCAGCTTTATCAACAACAGCAACAACAGCAACAGCAACAACAGAACGGTTTTAAGCGGAGACGGGATGCTA
shaker_Frag1524-1692_insertion1563	GCTTTTCTCGTCTCCTCGGTCTGCAGTGGCCCCCTTCCTGGGTGCTCAACAACAGCAGAGCGGGAGACCGGGGTCTCTGAGCCAACAACCCGTGGCCAGTTCATTGTCAATGTCAATAGATAAGCAGCTTCAACACCCTTTGCAACAATTGACACAAACTCAGCTTTATCAACAACAGCAACAACAGCAACAGCAACAACAGAACGGTTTTAAGCGGAGACGGGATGCTA
shaker_Frag1524-1692_insertion1566	GCTTTTCTCGTCTCCTCGGTCTGCAGTGGCCCCCTTCCTGGGTGCTCAACAACAGCAGCAAAGCGGGAGACCGGGGTCTCTGAGCCAACCCGTGGCCAGTTCATTGTCAATGTCAATAGATAAGCAGCTTCAACACCCTTTGCAACAATTGACACAAACTCAGCTTTATCAACAACAGCAACAACAGCAACAGCAACAACAGAACGGTTTTAAGCGGAGACGGGATGCTA
shaker_Frag1524-1692_insertion1569	GCTTTTCTCGTCTCCTCGGTCTGCAGTGGCCCCCTTCCTGGGTGCTCAACAACAGCAGCAACAAAGCGGGAGACCGGGGTCTCTGAGCCCCGTGGCCAGTTCATTGTCAATGTCAATAGATAAGCAGCTTCAACACCCTTTGCAACAATTGACACAAACTCAGCTTTATCAACAACAGCAACAACAGCAACAGCAACAACAGAACGGTTTTAAGCGGAGACGGGATGCTA
shaker_Frag1524-1692_insertion1572	GCTTTTCTCGTCTCCTCGGTCTGCAGTGGCCCCCTTCCTGGGTGCTCAACAACAGCAGCAACAACCCAGCGGGAGACCGGGGTCTCTGAGCGTGGCCAGTTCATTGTCAATGTCAATAGATAAGCAGCTTCAACACCCTTTGCAACAATTGACACAAACTCAGCTTTATCAACAACAGCAACAACAGCAACAGCAACAACAGAACGGTTTTAAGCGGAGACGGGATGCTA
shaker_Frag1524-1692_insertion1575	GCTTTTCTCGTCTCCTCGGTCTGCAGTGGCCCCCTTCCTGGGTGCTCAACAACAGCAGCAACAACCCGTGAGCGGGAGACCGGGGTCTCTGAGCGCCAGTTCATTGTCAATGTCAATAGATAAGCAGCTTCAACACCCTTTGCAACAATTGACACAAACTCAGCTTTATCAACAACAGCAACAACAGCAACAGCAACAACAGAACGGTTTTAAGCGGAGACGGGATGCTA
shaker_Frag1524-1692_insertion1578	GCTTTTCTCGTCTCCTCGGTCTGCAGTGGCCCCCTTCCTGGGTGCTCAACAACAGCAGCAACAACCCGTGGCCAGCGGGAGACCGGGGTCTCTGAGCAGTTCATTGTCAATGTCAATAGATAAGCAGCTTCAACACCCTTTGCAACAATTGACACAAACTCAGCTTTATCAACAACAGCAACAACAGCAACAGCAACAACAGAACGGTTTTAAGCGGAGACGGGATGCTA
shaker_Frag1524-1692_insertion1581	GCTTTTCTCGTCTCCTCGGTCTGCAGTGGCCCCCTTCCTGGGTGCTCAACAACAGCAGCAACAACCCGTGGCCAGTAGCGGGAGACCGGGGTCTCTGAGCTCATTGTCAATGTCAATAGATAAGCAGCTTCAACACCCTTTGCAACAATTGACACAAACTCAGCTTTATCAACAACAGCAACAACAGCAACAGCAACAACAGAACGGTTTTAAGCGGAGACGGGATGCTA
shaker_Frag1524-1692_insertion1584	GCTTTTCTCGTCTCCTCGGTCTGCAGTGGCCCCCTTCCTGGGTGCTCAACAACAGCAGCAACAACCCGTGGCCAGTTCAAGCGGGAGACCGGGGTCTCTGAGCTTGTCAATGTCAATAGATAAGCAGCTTCAACACCCTTTGCAACAATTGACACAAACTCAGCTTTATCAACAACAGCAACAACAGCAACAGCAACAACAGAACGGTTTTAAGCGGAGACGGGATGCTA
shaker_Frag1524-1692_insertion1587	GCTTTTCTCGTCTCCTCGGTCTGCAGTGGCCCCCTTCCTGGGTGCTCAACAACAGCAGCAACAACCCGTGGCCAGTTCATTGAGCGGGAGACCGGGGTCTCTGAGCTCAATGTCAATAGATAAGCAGCTTCAACACCCTTTGCAACAATTGACACAAACTCAGCTTTATCAACAACAGCAACAACAGCAACAGCAACAACAGAACGGTTTTAAGCGGAGACGGGATGCTA
shaker_Frag1524-1692_insertion1590	GCTTTTCTCGTCTCCTCGGTCTGCAGTGGCCCCCTTCCTGGGTGCTCAACAACAGCAGCAACAACCCGTGGCCAGTTCATTGTCAAGCGGGAGACCGGGGTCTCTGAGCATGTCAATAGATAAGCAGCTTCAACACCCTTTGCAACAATTGACACAAACTCAGCTTTATCAACAACAGCAACAACAGCAACAGCAACAACAGAACGGTTTTAAGCGGAGACGGGATGCTA
shaker_Frag1524-1692_insertion1593	GCTTTTCTCGTCTCCTCGGTCTGCAGTGGCCCCCTTCCTGGGTGCTCAACAACAGCAGCAACAACCCGTGGCCAGTTCATTGTCAATGAGCGGGAGACCGGGGTCTCTGAGCTCAATAGATAAGCAGCTTCAACACCCTTTGCAACAATTGACACAAACTCAGCTTTATCAACAACAGCAACAACAGCAACAGCAACAACAGAACGGTTTTAAGCGGAGACGGGATGCTA
shaker_Frag1524-1692_insertion1596	GCTTTTCTCGTCTCCTCGGTCTGCAGTGGCCCCCTTCCTGGGTGCTCAACAACAGCAGCAACAACCCGTGGCCAGTTCATTGTCAATGTCAAGCGGGAGACCGGGGTCTCTGAGCATAGATAAGCAGCTTCAACACCCTTTGCAACAATTGACACAAACTCAGCTTTATCAACAACAGCAACAACAGCAACAGCAACAACAGAACGGTTTTAAGCGGAGACGGGATGCTA
shaker_Frag1524-1692_insertion1599	GCTTTTCTCGTCTCCTCGGTCTGCAGTGGCCCCCTTCCTGGGTGCTCAACAACAGCAGCAACAACCCGTGGCCAGTTCATTGTCAATGTCAATAAGCGGGAGACCGGGGTCTCTGAGCGATAAGCAGCTTCAACACCCTTTGCAACAATTGACACAAACTCAGCTTTATCAACAACAGCAACAACAGCAACAGCAACAACAGAACGGTTTTAAGCGGAGACGGGATGCTA
shaker_Frag1524-1692_insertion1602	GCTTTTCTCGTCTCCTCGGTCTGCAGTGGCCCCCTTCCTGGGTGCTCAACAACAGCAGCAACAACCCGTGGCCAGTTCATTGTCAATGTCAATAGATAGCGGGAGACCGGGGTCTCTGAGCAAGCAGCTTCAACACCCTTTGCAACAATTGACACAAACTCAGCTTTATCAACAACAGCAACAACAGCAACAGCAACAACAGAACGGTTTTAAGCGGAGACGGGATGCTA
shaker_Frag1524-1692_insertion1605	GCTTTTCTCGTCTCCTCGGTCTGCAGTGGCCCCCTTCCTGGGTGCTCAACAACAGCAGCAACAACCCGTGGCCAGTTCATTGTCAATGTCAATAGATAAGAGCGGGAGACCGGGGTCTCTGAGCCAGCTTCAACACCCTTTGCAACAATTGACACAAACTCAGCTTTATCAACAACAGCAACAACAGCAACAGCAACAACAGAACGGTTTTAAGCGGAGACGGGATGCTA
shaker_Frag1524-1692_insertion1608	GCTTTTCTCGTCTCCTCGGTCTGCAGTGGCCCCCTTCCTGGGTGCTCAACAACAGCAGCAACAACCCGTGGCCAGTTCATTGTCAATGTCAATAGATAAGCAGAGCGGGAGACCGGGGTCTCTGAGCCTTCAACACCCTTTGCAACAATTGACACAAACTCAGCTTTATCAACAACAGCAACAACAGCAACAGCAACAACAGAACGGTTTTAAGCGGAGACGGGATGCTA
shaker_Frag1524-1692_insertion1611	GCTTTTCTCGTCTCCTCGGTCTGCAGTGGCCCCCTTCCTGGGTGCTCAACAACAGCAGCAACAACCCGTGGCCAGTTCATTGTCAATGTCAATAGATAAGCAGCTTAGCGGGAGACCGGGGTCTCTGAGCCAACACCCTTTGCAACAATTGACACAAACTCAGCTTTATCAACAACAGCAACAACAGCAACAGCAACAACAGAACGGTTTTAAGCGGAGACGGGATGCTA
shaker_Frag1524-1692_insertion1614	GCTTTTCTCGTCTCCTCGGTCTGCAGTGGCCCCCTTCCTGGGTGCTCAACAACAGCAGCAACAACCCGTGGCCAGTTCATTGTCAATGTCAATAGATAAGCAGCTTCAAAGCGGGAGACCGGGGTCTCTGAGCCACCCTTTGCAACAATTGACACAAACTCAGCTTTATCAACAACAGCAACAACAGCAACAGCAACAACAGAACGGTTTTAAGCGGAGACGGGATGCTA
shaker_Frag1524-1692_insertion1617	GCTTTTCTCGTCTCCTCGGTCTGCAGTGGCCCCCTTCCTGGGTGCTCAACAACAGCAGCAACAACCCGTGGCCAGTTCATTGTCAATGTCAATAGATAAGCAGCTTCAACACAGCGGGAGACCGGGGTCTCTGAGCCCTTTGCAACAATTGACACAAACTCAGCTTTATCAACAACAGCAACAACAGCAACAGCAACAACAGAACGGTTTTAAGCGGAGACGGGATGCTA
shaker_Frag1524-1692_insertion1620	GCTTTTCTCGTCTCCTCGGTCTGCAGTGGCCCCCTTCCTGGGTGCTCAACAACAGCAGCAACAACCCGTGGCCAGTTCATTGTCAATGTCAATAGATAAGCAGCTTCAACACCCTAGCGGGAGACCGGGGTCTCTGAGCTTGCAACAATTGACACAAACTCAGCTTTATCAACAACAGCAACAACAGCAACAGCAACAACAGAACGGTTTTAAGCGGAGACGGGATGCTA
shaker_Frag1524-1692_insertion1623	GCTTTTCTCGTCTCCTCGGTCTGCAGTGGCCCCCTTCCTGGGTGCTCAACAACAGCAGCAACAACCCGTGGCCAGTTCATTGTCAATGTCAATAGATAAGCAGCTTCAACACCCTTTGAGCGGGAGACCGGGGTCTCTGAGCCAACAATTGACACAAACTCAGCTTTATCAACAACAGCAACAACAGCAACAGCAACAACAGAACGGTTTTAAGCGGAGACGGGATGCTA
shaker_Frag1524-1692_insertion1626	GCTTTTCTCGTCTCCTCGGTCTGCAGTGGCCCCCTTCCTGGGTGCTCAACAACAGCAGCAACAACCCGTGGCCAGTTCATTGTCAATGTCAATAGATAAGCAGCTTCAACACCCTTTGCAAAGCGGGAGACCGGGGTCTCTGAGCCAATTGACACAAACTCAGCTTTATCAACAACAGCAACAACAGCAACAGCAACAACAGAACGGTTTTAAGCGGAGACGGGATGCTA
shaker_Frag1524-1692_insertion1629	GCTTTTCTCGTCTCCTCGGTCTGCAGTGGCCCCCTTCCTGGGTGCTCAACAACAGCAGCAACAACCCGTGGCCAGTTCATTGTCAATGTCAATAGATAAGCAGCTTCAACACCCTTTGCAACAAAGCGGGAGACCGGGGTCTCTGAGCTTGACACAAACTCAGCTTTATCAACAACAGCAACAACAGCAACAGCAACAACAGAACGGTTTTAAGCGGAGACGGGATGCTA
shaker_Frag1524-1692_insertion1632	GCTTTTCTCGTCTCCTCGGTCTGCAGTGGCCCCCTTCCTGGGTGCTCAACAACAGCAGCAACAACCCGTGGCCAGTTCATTGTCAATGTCAATAGATAAGCAGCTTCAACACCCTTTGCAACAATTGAGCGGGAGACCGGGGTCTCTGAGCACACAAACTCAGCTTTATCAACAACAGCAACAACAGCAACAGCAACAACAGAACGGTTTTAAGCGGAGACGGGATGCTA
shaker_Frag1524-1692_insertion1635	GCTTTTCTCGTCTCCTCGGTCTGCAGTGGCCCCCTTCCTGGGTGCTCAACAACAGCAGCAACAACCCGTGGCCAGTTCATTGTCAATGTCAATAGATAAGCAGCTTCAACACCCTTTGCAACAATTGACAAGCGGGAGACCGGGGTCTCTGAGCCAAACTCAGCTTTATCAACAACAGCAACAACAGCAACAGCAACAACAGAACGGTTTTAAGCGGAGACGGGATGCTA
shaker_Frag1524-1692_insertion1638	GCTTTTCTCGTCTCCTCGGTCTGCAGTGGCCCCCTTCCTGGGTGCTCAACAACAGCAGCAACAACCCGTGGCCAGTTCATTGTCAATGTCAATAGATAAGCAGCTTCAACACCCTTTGCAACAATTGACACAAAGCGGGAGACCGGGGTCTCTGAGCACTCAGCTTTATCAACAACAGCAACAACAGCAACAGCAACAACAGAACGGTTTTAAGCGGAGACGGGATGCTA
shaker_Frag1524-1692_insertion1641	GCTTTTCTCGTCTCCTCGGTCTGCAGTGGCCCCCTTCCTGGGTGCTCAACAACAGCAGCAACAACCCGTGGCCAGTTCATTGTCAATGTCAATAGATAAGCAGCTTCAACACCCTTTGCAACAATTGACACAAACTAGCGGGAGACCGGGGTCTCTGAGCCAGCTTTATCAACAACAGCAACAACAGCAACAGCAACAACAGAACGGTTTTAAGCGGAGACGGGATGCTA
shaker_Frag1524-1692_insertion1644	GCTTTTCTCGTCTCCTCGGTCTGCAGTGGCCCCCTTCCTGGGTGCTCAACAACAGCAGCAACAACCCGTGGCCAGTTCATTGTCAATGTCAATAGATAAGCAGCTTCAACACCCTTTGCAACAATTGACACAAACTCAGAGCGGGAGACCGGGGTCTCTGAGCCTTTATCAACAACAGCAACAACAGCAACAGCAACAACAGAACGGTTTTAAGCGGAGACGGGATGCTA
shaker_Frag1524-1692_insertion1647	GCTTTTCTCGTCTCCTCGGTCTGCAGTGGCCCCCTTCCTGGGTGCTCAACAACAGCAGCAACAACCCGTGGCCAGTTCATTGTCAATGTCAATAGATAAGCAGCTTCAACACCCTTTGCAACAATTGACACAAACTCAGCTTAGCGGGAGACCGGGGTCTCTGAGCTATCAACAACAGCAACAACAGCAACAGCAACAACAGAACGGTTTTAAGCGGAGACGGGATGCTA
shaker_Frag1524-1692_insertion1650	GCTTTTCTCGTCTCCTCGGTCTGCAGTGGCCCCCTTCCTGGGTGCTCAACAACAGCAGCAACAACCCGTGGCCAGTTCATTGTCAATGTCAATAGATAAGCAGCTTCAACACCCTTTGCAACAATTGACACAAACTCAGCTTTATAGCGGGAGACCGGGGTCTCTGAGCCAACAACAGCAACAACAGCAACAGCAACAACAGAACGGTTTTAAGCGGAGACGGGATGCTA
shaker_Frag1524-1692_insertion1653	GCTTTTCTCGTCTCCTCGGTCTGCAGTGGCCCCCTTCCTGGGTGCTCAACAACAGCAGCAACAACCCGTGGCCAGTTCATTGTCAATGTCAATAGATAAGCAGCTTCAACACCCTTTGCAACAATTGACACAAACTCAGCTTTATCAAAGCGGGAGACCGGGGTCTCTGAGCCAACAGCAACAACAGCAACAGCAACAACAGAACGGTTTTAAGCGGAGACGGGATGCTA
shaker_Frag1524-1692_insertion1656	GCTTTTCTCGTCTCCTCGGTCTGCAGTGGCCCCCTTCCTGGGTGCTCAACAACAGCAGCAACAACCCGTGGCCAGTTCATTGTCAATGTCAATAGATAAGCAGCTTCAACACCCTTTGCAACAATTGACACAAACTCAGCTTTATCAACAAAGCGGGAGACCGGGGTCTCTGAGCCAGCAACAACAGCAACAGCAACAACAGAACGGTTTTAAGCGGAGACGGGATGCTA
shaker_Frag1524-1692_insertion1659	GCTTTTCTCGTCTCCTCGGTCTGCAGTGGCCCCCTTCCTGGGTGCTCAACAACAGCAGCAACAACCCGTGGCCAGTTCATTGTCAATGTCAATAGATAAGCAGCTTCAACACCCTTTGCAACAATTGACACAAACTCAGCTTTATCAACAACAGAGCGGGAGACCGGGGTCTCTGAGCCAACAACAGCAACAGCAACAACAGAACGGTTTTAAGCGGAGACGGGATGCTA
shaker_Frag1524-1692_insertion1662	GCTTTTCTCGTCTCCTCGGTCTGCAGTGGCCCCCTTCCTGGGTGCTCAACAACAGCAGCAACAACCCGTGGCCAGTTCATTGTCAATGTCAATAGATAAGCAGCTTCAACACCCTTTGCAACAATTGACACAAACTCAGCTTTATCAACAACAGCAAAGCGGGAGACCGGGGTCTCTGAGCCAACAGCAACAGCAACAACAGAACGGTTTTAAGCGGAGACGGGATGCTA
shaker_Frag1524-1692_insertion1665	GCTTTTCTCGTCTCCTCGGTCTGCAGTGGCCCCCTTCCTGGGTGCTCAACAACAGCAGCAACAACCCGTGGCCAGTTCATTGTCAATGTCAATAGATAAGCAGCTTCAACACCCTTTGCAACAATTGACACAAACTCAGCTTTATCAACAACAGCAACAAAGCGGGAGACCGGGGTCTCTGAGCCAGCAACAGCAACAACAGAACGGTTTTAAGCGGAGACGGGATGCTA
shaker_Frag1524-1692_insertion1668	GCTTTTCTCGTCTCCTCGGTCTGCAGTGGCCCCCTTCCTGGGTGCTCAACAACAGCAGCAACAACCCGTGGCCAGTTCATTGTCAATGTCAATAGATAAGCAGCTTCAACACCCTTTGCAACAATTGACACAAACTCAGCTTTATCAACAACAGCAACAACAGAGCGGGAGACCGGGGTCTCTGAGCCAACAGCAACAACAGAACGGTTTTAAGCGGAGACGGGATGCTA
shaker_Frag1524-1692_insertion1671	GCTTTTCTCGTCTCCTCGGTCTGCAGTGGCCCCCTTCCTGGGTGCTCAACAACAGCAGCAACAACCCGTGGCCAGTTCATTGTCAATGTCAATAGATAAGCAGCTTCAACACCCTTTGCAACAATTGACACAAACTCAGCTTTATCAACAACAGCAACAACAGCAAAGCGGGAGACCGGGGTCTCTGAGCCAGCAACAACAGAACGGTTTTAAGCGGAGACGGGATGCTA
shaker_Frag1524-1692_insertion1674	GCTTTTCTCGTCTCCTCGGTCTGCAGTGGCCCCCTTCCTGGGTGCTCAACAACAGCAGCAACAACCCGTGGCCAGTTCATTGTCAATGTCAATAGATAAGCAGCTTCAACACCCTTTGCAACAATTGACACAAACTCAGCTTTATCAACAACAGCAACAACAGCAACAGAGCGGGAGACCGGGGTCTCTGAGCCAACAACAGAACGGTTTTAAGCGGAGACGGGATGCTA
shaker_Frag1524-1692_insertion1677	GCTTTTCTCGTCTCCTCGGTCTGCAGTGGCCCCCTTCCTGGGTGCTCAACAACAGCAGCAACAACCCGTGGCCAGTTCATTGTCAATGTCAATAGATAAGCAGCTTCAACACCCTTTGCAACAATTGACACAAACTCAGCTTTATCAACAACAGCAACAACAGCAACAGCAAAGCGGGAGACCGGGGTCTCTGAGCCAACAGAACGGTTTTAAGCGGAGACGGGATGCTA
shaker_Frag1524-1692_insertion1680	GCTTTTCTCGTCTCCTCGGTCTGCAGTGGCCCCCTTCCTGGGTGCTCAACAACAGCAGCAACAACCCGTGGCCAGTTCATTGTCAATGTCAATAGATAAGCAGCTTCAACACCCTTTGCAACAATTGACACAAACTCAGCTTTATCAACAACAGCAACAACAGCAACAGCAACAAAGCGGGAGACCGGGGTCTCTGAGCCAGAACGGTTTTAAGCGGAGACGGGATGCTA
shaker_Frag1524-1692_insertion1683	GCTTTTCTCGTCTCCTCGGTCTGCAGTGGCCCCCTTCCTGGGTGCTCAACAACAGCAGCAACAACCCGTGGCCAGTTCATTGTCAATGTCAATAGATAAGCAGCTTCAACACCCTTTGCAACAATTGACACAAACTCAGCTTTATCAACAACAGCAACAACAGCAACAGCAACAACAGAGCGGGAGACCGGGGTCTCTGAGCAACGGTTTTAAGCGGAGACGGGATGCTA
shaker_Frag1524-1692_insertion1686	GCTTTTCTCGTCTCCTCGGTCTGCAGTGGCCCCCTTCCTGGGTGCTCAACAACAGCAGCAACAACCCGTGGCCAGTTCATTGTCAATGTCAATAGATAAGCAGCTTCAACACCCTTTGCAACAATTGACACAAACTCAGCTTTATCAACAACAGCAACAACAGCAACAGCAACAACAGAACAGCGGGAGACCGGGGTCTCTGAGCGGTTTTAAGCGGAGACGGGATGCTA
shaker_Frag1524-1692_insertion1689	GCTTTTCTCGTCTCCTCGGTCTGCAGTGGCCCCCTTCCTGGGTGCTCAACAACAGCAGCAACAACCCGTGGCCAGTTCATTGTCAATGTCAATAGATAAGCAGCTTCAACACCCTTTGCAACAATTGACACAAACTCAGCTTTATCAACAACAGCAACAACAGCAACAGCAACAACAGAACGGTAGCGGGAGACCGGGGTCTCTGAGCTTTAAGCGGAGACGGGATGCTA
shaker_Frag1524-1692_insertion1692	GCTTTTCTCGTCTCCTCGGTCTGCAGTGGCCCCCTTCCTGGGTGCTCAACAACAGCAGCAACAACCCGTGGCCAGTTCATTGTCAATGTCAATAGATAAGCAGCTTCAACACCCTTTGCAACAATTGACACAAACTCAGCTTTATCAACAACAGCAACAACAGCAACAGCAACAACAGAACGGTTTTAGCGGGAGACCGGGGTCTCTGAGCAAGCGGAGACGGGATGCTA
shaker_Frag1695-1845_insertion1695	TTCGTACCATTCTATGGCGTCTCCTAAGAGCGGGAGACCGGGGTCTCTGAGCCAGCAGCAACAACAAACCCAGCAGCAATTGCAACAACAGCAGTCTCACACAATCAACGCTTCCGCAGCCGCTGCAACCTCAGGATCTGGGAGTAGCGGCCTGACCATGCGACACAATAATGCCTTGGCTGTGTCCATTGAAACTGATGTTGCTGGGAGACGGCTGTTTGCGGTAGTTA
shaker_Frag1695-1845_insertion1698	TTCGTACCATTCTATGGCGTCTCCTAAGCAGAGCGGGAGACCGGGGTCTCTGAGCCAGCAACAACAAACCCAGCAGCAATTGCAACAACAGCAGTCTCACACAATCAACGCTTCCGCAGCCGCTGCAACCTCAGGATCTGGGAGTAGCGGCCTGACCATGCGACACAATAATGCCTTGGCTGTGTCCATTGAAACTGATGTTGCTGGGAGACGGCTGTTTGCGGTAGTTA
shaker_Frag1695-1845_insertion1701	TTCGTACCATTCTATGGCGTCTCCTAAGCAGCAGAGCGGGAGACCGGGGTCTCTGAGCCAACAACAAACCCAGCAGCAATTGCAACAACAGCAGTCTCACACAATCAACGCTTCCGCAGCCGCTGCAACCTCAGGATCTGGGAGTAGCGGCCTGACCATGCGACACAATAATGCCTTGGCTGTGTCCATTGAAACTGATGTTGCTGGGAGACGGCTGTTTGCGGTAGTTA
shaker_Frag1695-1845_insertion1704	TTCGTACCATTCTATGGCGTCTCCTAAGCAGCAGCAAAGCGGGAGACCGGGGTCTCTGAGCCAACAAACCCAGCAGCAATTGCAACAACAGCAGTCTCACACAATCAACGCTTCCGCAGCCGCTGCAACCTCAGGATCTGGGAGTAGCGGCCTGACCATGCGACACAATAATGCCTTGGCTGTGTCCATTGAAACTGATGTTGCTGGGAGACGGCTGTTTGCGGTAGTTA
shaker_Frag1695-1845_insertion1707	TTCGTACCATTCTATGGCGTCTCCTAAGCAGCAGCAACAAAGCGGGAGACCGGGGTCTCTGAGCCAAACCCAGCAGCAATTGCAACAACAGCAGTCTCACACAATCAACGCTTCCGCAGCCGCTGCAACCTCAGGATCTGGGAGTAGCGGCCTGACCATGCGACACAATAATGCCTTGGCTGTGTCCATTGAAACTGATGTTGCTGGGAGACGGCTGTTTGCGGTAGTTA
shaker_Frag1695-1845_insertion1710	TTCGTACCATTCTATGGCGTCTCCTAAGCAGCAGCAACAACAAAGCGGGAGACCGGGGTCTCTGAGCACCCAGCAGCAATTGCAACAACAGCAGTCTCACACAATCAACGCTTCCGCAGCCGCTGCAACCTCAGGATCTGGGAGTAGCGGCCTGACCATGCGACACAATAATGCCTTGGCTGTGTCCATTGAAACTGATGTTGCTGGGAGACGGCTGTTTGCGGTAGTTA
shaker_Frag1695-1845_insertion1713	TTCGTACCATTCTATGGCGTCTCCTAAGCAGCAGCAACAACAAACCAGCGGGAGACCGGGGTCTCTGAGCCAGCAGCAATTGCAACAACAGCAGTCTCACACAATCAACGCTTCCGCAGCCGCTGCAACCTCAGGATCTGGGAGTAGCGGCCTGACCATGCGACACAATAATGCCTTGGCTGTGTCCATTGAAACTGATGTTGCTGGGAGACGGCTGTTTGCGGTAGTTA
shaker_Frag1695-1845_insertion1716	TTCGTACCATTCTATGGCGTCTCCTAAGCAGCAGCAACAACAAACCCAGAGCGGGAGACCGGGGTCTCTGAGCCAGCAATTGCAACAACAGCAGTCTCACACAATCAACGCTTCCGCAGCCGCTGCAACCTCAGGATCTGGGAGTAGCGGCCTGACCATGCGACACAATAATGCCTTGGCTGTGTCCATTGAAACTGATGTTGCTGGGAGACGGCTGTTTGCGGTAGTTA
shaker_Frag1695-1845_insertion1719	TTCGTACCATTCTATGGCGTCTCCTAAGCAGCAGCAACAACAAACCCAGCAGAGCGGGAGACCGGGGTCTCTGAGCCAATTGCAACAACAGCAGTCTCACACAATCAACGCTTCCGCAGCCGCTGCAACCTCAGGATCTGGGAGTAGCGGCCTGACCATGCGACACAATAATGCCTTGGCTGTGTCCATTGAAACTGATGTTGCTGGGAGACGGCTGTTTGCGGTAGTTA
shaker_Frag1695-1845_insertion1722	TTCGTACCATTCTATGGCGTCTCCTAAGCAGCAGCAACAACAAACCCAGCAGCAAAGCGGGAGACCGGGGTCTCTGAGCTTGCAACAACAGCAGTCTCACACAATCAACGCTTCCGCAGCCGCTGCAACCTCAGGATCTGGGAGTAGCGGCCTGACCATGCGACACAATAATGCCTTGGCTGTGTCCATTGAAACTGATGTTGCTGGGAGACGGCTGTTTGCGGTAGTTA
shaker_Frag1695-1845_insertion1725	TTCGTACCATTCTATGGCGTCTCCTAAGCAGCAGCAACAACAAACCCAGCAGCAATTGAGCGGGAGACCGGGGTCTCTGAGCCAACAACAGCAGTCTCACACAATCAACGCTTCCGCAGCCGCTGCAACCTCAGGATCTGGGAGTAGCGGCCTGACCATGCGACACAATAATGCCTTGGCTGTGTCCATTGAAACTGATGTTGCTGGGAGACGGCTGTTTGCGGTAGTTA
shaker_Frag1695-1845_insertion1728	TTCGTACCATTCTATGGCGTCTCCTAAGCAGCAGCAACAACAAACCCAGCAGCAATTGCAAAGCGGGAGACCGGGGTCTCTGAGCCAACAGCAGTCTCACACAATCAACGCTTCCGCAGCCGCTGCAACCTCAGGATCTGGGAGTAGCGGCCTGACCATGCGACACAATAATGCCTTGGCTGTGTCCATTGAAACTGATGTTGCTGGGAGACGGCTGTTTGCGGTAGTTA
shaker_Frag1695-1845_insertion1731	TTCGTACCATTCTATGGCGTCTCCTAAGCAGCAGCAACAACAAACCCAGCAGCAATTGCAACAAAGCGGGAGACCGGGGTCTCTGAGCCAGCAGTCTCACACAATCAACGCTTCCGCAGCCGCTGCAACCTCAGGATCTGGGAGTAGCGGCCTGACCATGCGACACAATAATGCCTTGGCTGTGTCCATTGAAACTGATGTTGCTGGGAGACGGCTGTTTGCGGTAGTTA
shaker_Frag1695-1845_insertion1734	TTCGTACCATTCTATGGCGTCTCCTAAGCAGCAGCAACAACAAACCCAGCAGCAATTGCAACAACAGAGCGGGAGACCGGGGTCTCTGAGCCAGTCTCACACAATCAACGCTTCCGCAGCCGCTGCAACCTCAGGATCTGGGAGTAGCGGCCTGACCATGCGACACAATAATGCCTTGGCTGTGTCCATTGAAACTGATGTTGCTGGGAGACGGCTGTTTGCGGTAGTTA
shaker_Frag1695-1845_insertion1737	TTCGTACCATTCTATGGCGTCTCCTAAGCAGCAGCAACAACAAACCCAGCAGCAATTGCAACAACAGCAGAGCGGGAGACCGGGGTCTCTGAGCTCTCACACAATCAACGCTTCCGCAGCCGCTGCAACCTCAGGATCTGGGAGTAGCGGCCTGACCATGCGACACAATAATGCCTTGGCTGTGTCCATTGAAACTGATGTTGCTGGGAGACGGCTGTTTGCGGTAGTTA
shaker_Frag1695-1845_insertion1740	TTCGTACCATTCTATGGCGTCTCCTAAGCAGCAGCAACAACAAACCCAGCAGCAATTGCAACAACAGCAGTCTAGCGGGAGACCGGGGTCTCTGAGCCACACAATCAACGCTTCCGCAGCCGCTGCAACCTCAGGATCTGGGAGTAGCGGCCTGACCATGCGACACAATAATGCCTTGGCTGTGTCCATTGAAACTGATGTTGCTGGGAGACGGCTGTTTGCGGTAGTTA
shaker_Frag1695-1845_insertion1743	TTCGTACCATTCTATGGCGTCTCCTAAGCAGCAGCAACAACAAACCCAGCAGCAATTGCAACAACAGCAGTCTCACAGCGGGAGACCGGGGTCTCTGAGCACAATCAACGCTTCCGCAGCCGCTGCAACCTCAGGATCTGGGAGTAGCGGCCTGACCATGCGACACAATAATGCCTTGGCTGTGTCCATTGAAACTGATGTTGCTGGGAGACGGCTGTTTGCGGTAGTTA
shaker_Frag1695-1845_insertion1746	TTCGTACCATTCTATGGCGTCTCCTAAGCAGCAGCAACAACAAACCCAGCAGCAATTGCAACAACAGCAGTCTCACACAAGCGGGAGACCGGGGTCTCTGAGCATCAACGCTTCCGCAGCCGCTGCAACCTCAGGATCTGGGAGTAGCGGCCTGACCATGCGACACAATAATGCCTTGGCTGTGTCCATTGAAACTGATGTTGCTGGGAGACGGCTGTTTGCGGTAGTTA
shaker_Frag1695-1845_insertion1749	TTCGTACCATTCTATGGCGTCTCCTAAGCAGCAGCAACAACAAACCCAGCAGCAATTGCAACAACAGCAGTCTCACACAATCAGCGGGAGACCGGGGTCTCTGAGCAACGCTTCCGCAGCCGCTGCAACCTCAGGATCTGGGAGTAGCGGCCTGACCATGCGACACAATAATGCCTTGGCTGTGTCCATTGAAACTGATGTTGCTGGGAGACGGCTGTTTGCGGTAGTTA
shaker_Frag1695-1845_insertion1752	TTCGTACCATTCTATGGCGTCTCCTAAGCAGCAGCAACAACAAACCCAGCAGCAATTGCAACAACAGCAGTCTCACACAATCAACAGCGGGAGACCGGGGTCTCTGAGCGCTTCCGCAGCCGCTGCAACCTCAGGATCTGGGAGTAGCGGCCTGACCATGCGACACAATAATGCCTTGGCTGTGTCCATTGAAACTGATGTTGCTGGGAGACGGCTGTTTGCGGTAGTTA
shaker_Frag1695-1845_insertion1755	TTCGTACCATTCTATGGCGTCTCCTAAGCAGCAGCAACAACAAACCCAGCAGCAATTGCAACAACAGCAGTCTCACACAATCAACGCTAGCGGGAGACCGGGGTCTCTGAGCTCCGCAGCCGCTGCAACCTCAGGATCTGGGAGTAGCGGCCTGACCATGCGACACAATAATGCCTTGGCTGTGTCCATTGAAACTGATGTTGCTGGGAGACGGCTGTTTGCGGTAGTTA
shaker_Frag1695-1845_insertion1758	TTCGTACCATTCTATGGCGTCTCCTAAGCAGCAGCAACAACAAACCCAGCAGCAATTGCAACAACAGCAGTCTCACACAATCAACGCTTCCAGCGGGAGACCGGGGTCTCTGAGCGCAGCCGCTGCAACCTCAGGATCTGGGAGTAGCGGCCTGACCATGCGACACAATAATGCCTTGGCTGTGTCCATTGAAACTGATGTTGCTGGGAGACGGCTGTTTGCGGTAGTTA
shaker_Frag1695-1845_insertion1761	TTCGTACCATTCTATGGCGTCTCCTAAGCAGCAGCAACAACAAACCCAGCAGCAATTGCAACAACAGCAGTCTCACACAATCAACGCTTCCGCAAGCGGGAGACCGGGGTCTCTGAGCGCCGCTGCAACCTCAGGATCTGGGAGTAGCGGCCTGACCATGCGACACAATAATGCCTTGGCTGTGTCCATTGAAACTGATGTTGCTGGGAGACGGCTGTTTGCGGTAGTTA
shaker_Frag1695-1845_insertion1764	TTCGTACCATTCTATGGCGTCTCCTAAGCAGCAGCAACAACAAACCCAGCAGCAATTGCAACAACAGCAGTCTCACACAATCAACGCTTCCGCAGCCAGCGGGAGACCGGGGTCTCTGAGCGCTGCAACCTCAGGATCTGGGAGTAGCGGCCTGACCATGCGACACAATAATGCCTTGGCTGTGTCCATTGAAACTGATGTTGCTGGGAGACGGCTGTTTGCGGTAGTTA
shaker_Frag1695-1845_insertion1767	TTCGTACCATTCTATGGCGTCTCCTAAGCAGCAGCAACAACAAACCCAGCAGCAATTGCAACAACAGCAGTCTCACACAATCAACGCTTCCGCAGCCGCTAGCGGGAGACCGGGGTCTCTGAGCGCAACCTCAGGATCTGGGAGTAGCGGCCTGACCATGCGACACAATAATGCCTTGGCTGTGTCCATTGAAACTGATGTTGCTGGGAGACGGCTGTTTGCGGTAGTTA
shaker_Frag1695-1845_insertion1770	TTCGTACCATTCTATGGCGTCTCCTAAGCAGCAGCAACAACAAACCCAGCAGCAATTGCAACAACAGCAGTCTCACACAATCAACGCTTCCGCAGCCGCTGCAAGCGGGAGACCGGGGTCTCTGAGCACCTCAGGATCTGGGAGTAGCGGCCTGACCATGCGACACAATAATGCCTTGGCTGTGTCCATTGAAACTGATGTTGCTGGGAGACGGCTGTTTGCGGTAGTTA
shaker_Frag1695-1845_insertion1773	TTCGTACCATTCTATGGCGTCTCCTAAGCAGCAGCAACAACAAACCCAGCAGCAATTGCAACAACAGCAGTCTCACACAATCAACGCTTCCGCAGCCGCTGCAACCAGCGGGAGACCGGGGTCTCTGAGCTCAGGATCTGGGAGTAGCGGCCTGACCATGCGACACAATAATGCCTTGGCTGTGTCCATTGAAACTGATGTTGCTGGGAGACGGCTGTTTGCGGTAGTTA
shaker_Frag1695-1845_insertion1776	TTCGTACCATTCTATGGCGTCTCCTAAGCAGCAGCAACAACAAACCCAGCAGCAATTGCAACAACAGCAGTCTCACACAATCAACGCTTCCGCAGCCGCTGCAACCTCAAGCGGGAGACCGGGGTCTCTGAGCGGATCTGGGAGTAGCGGCCTGACCATGCGACACAATAATGCCTTGGCTGTGTCCATTGAAACTGATGTTGCTGGGAGACGGCTGTTTGCGGTAGTTA
shaker_Frag1695-1845_insertion1779	TTCGTACCATTCTATGGCGTCTCCTAAGCAGCAGCAACAACAAACCCAGCAGCAATTGCAACAACAGCAGTCTCACACAATCAACGCTTCCGCAGCCGCTGCAACCTCAGGAAGCGGGAGACCGGGGTCTCTGAGCTCTGGGAGTAGCGGCCTGACCATGCGACACAATAATGCCTTGGCTGTGTCCATTGAAACTGATGTTGCTGGGAGACGGCTGTTTGCGGTAGTTA
shaker_Frag1695-1845_insertion1782	TTCGTACCATTCTATGGCGTCTCCTAAGCAGCAGCAACAACAAACCCAGCAGCAATTGCAACAACAGCAGTCTCACACAATCAACGCTTCCGCAGCCGCTGCAACCTCAGGATCTAGCGGGAGACCGGGGTCTCTGAGCGGGAGTAGCGGCCTGACCATGCGACACAATAATGCCTTGGCTGTGTCCATTGAAACTGATGTTGCTGGGAGACGGCTGTTTGCGGTAGTTA
shaker_Frag1695-1845_insertion1785	TTCGTACCATTCTATGGCGTCTCCTAAGCAGCAGCAACAACAAACCCAGCAGCAATTGCAACAACAGCAGTCTCACACAATCAACGCTTCCGCAGCCGCTGCAACCTCAGGATCTGGGAGCGGGAGACCGGGGTCTCTGAGCAGTAGCGGCCTGACCATGCGACACAATAATGCCTTGGCTGTGTCCATTGAAACTGATGTTGCTGGGAGACGGCTGTTTGCGGTAGTTA
shaker_Frag1695-1845_insertion1788	TTCGTACCATTCTATGGCGTCTCCTAAGCAGCAGCAACAACAAACCCAGCAGCAATTGCAACAACAGCAGTCTCACACAATCAACGCTTCCGCAGCCGCTGCAACCTCAGGATCTGGGAGTAGCGGGAGACCGGGGTCTCTGAGCAGCGGCCTGACCATGCGACACAATAATGCCTTGGCTGTGTCCATTGAAACTGATGTTGCTGGGAGACGGCTGTTTGCGGTAGTTA
shaker_Frag1695-1845_insertion1791	TTCGTACCATTCTATGGCGTCTCCTAAGCAGCAGCAACAACAAACCCAGCAGCAATTGCAACAACAGCAGTCTCACACAATCAACGCTTCCGCAGCCGCTGCAACCTCAGGATCTGGGAGTAGCAGCGGGAGACCGGGGTCTCTGAGCGGCCTGACCATGCGACACAATAATGCCTTGGCTGTGTCCATTGAAACTGATGTTGCTGGGAGACGGCTGTTTGCGGTAGTTA
shaker_Frag1695-1845_insertion1794	TTCGTACCATTCTATGGCGTCTCCTAAGCAGCAGCAACAACAAACCCAGCAGCAATTGCAACAACAGCAGTCTCACACAATCAACGCTTCCGCAGCCGCTGCAACCTCAGGATCTGGGAGTAGCGGCAGCGGGAGACCGGGGTCTCTGAGCCTGACCATGCGACACAATAATGCCTTGGCTGTGTCCATTGAAACTGATGTTGCTGGGAGACGGCTGTTTGCGGTAGTTA
shaker_Frag1695-1845_insertion1797	TTCGTACCATTCTATGGCGTCTCCTAAGCAGCAGCAACAACAAACCCAGCAGCAATTGCAACAACAGCAGTCTCACACAATCAACGCTTCCGCAGCCGCTGCAACCTCAGGATCTGGGAGTAGCGGCCTGAGCGGGAGACCGGGGTCTCTGAGCACCATGCGACACAATAATGCCTTGGCTGTGTCCATTGAAACTGATGTTGCTGGGAGACGGCTGTTTGCGGTAGTTA
shaker_Frag1695-1845_insertion1800	TTCGTACCATTCTATGGCGTCTCCTAAGCAGCAGCAACAACAAACCCAGCAGCAATTGCAACAACAGCAGTCTCACACAATCAACGCTTCCGCAGCCGCTGCAACCTCAGGATCTGGGAGTAGCGGCCTGACCAGCGGGAGACCGGGGTCTCTGAGCATGCGACACAATAATGCCTTGGCTGTGTCCATTGAAACTGATGTTGCTGGGAGACGGCTGTTTGCGGTAGTTA
shaker_Frag1695-1845_insertion1803	TTCGTACCATTCTATGGCGTCTCCTAAGCAGCAGCAACAACAAACCCAGCAGCAATTGCAACAACAGCAGTCTCACACAATCAACGCTTCCGCAGCCGCTGCAACCTCAGGATCTGGGAGTAGCGGCCTGACCATGAGCGGGAGACCGGGGTCTCTGAGCCGACACAATAATGCCTTGGCTGTGTCCATTGAAACTGATGTTGCTGGGAGACGGCTGTTTGCGGTAGTTA
shaker_Frag1695-1845_insertion1806	TTCGTACCATTCTATGGCGTCTCCTAAGCAGCAGCAACAACAAACCCAGCAGCAATTGCAACAACAGCAGTCTCACACAATCAACGCTTCCGCAGCCGCTGCAACCTCAGGATCTGGGAGTAGCGGCCTGACCATGCGAAGCGGGAGACCGGGGTCTCTGAGCCACAATAATGCCTTGGCTGTGTCCATTGAAACTGATGTTGCTGGGAGACGGCTGTTTGCGGTAGTTA
shaker_Frag1695-1845_insertion1809	TTCGTACCATTCTATGGCGTCTCCTAAGCAGCAGCAACAACAAACCCAGCAGCAATTGCAACAACAGCAGTCTCACACAATCAACGCTTCCGCAGCCGCTGCAACCTCAGGATCTGGGAGTAGCGGCCTGACCATGCGACACAGCGGGAGACCGGGGTCTCTGAGCAATAATGCCTTGGCTGTGTCCATTGAAACTGATGTTGCTGGGAGACGGCTGTTTGCGGTAGTTA
shaker_Frag1695-1845_insertion1812	TTCGTACCATTCTATGGCGTCTCCTAAGCAGCAGCAACAACAAACCCAGCAGCAATTGCAACAACAGCAGTCTCACACAATCAACGCTTCCGCAGCCGCTGCAACCTCAGGATCTGGGAGTAGCGGCCTGACCATGCGACACAATAGCGGGAGACCGGGGTCTCTGAGCAATGCCTTGGCTGTGTCCATTGAAACTGATGTTGCTGGGAGACGGCTGTTTGCGGTAGTTA
shaker_Frag1695-1845_insertion1815	TTCGTACCATTCTATGGCGTCTCCTAAGCAGCAGCAACAACAAACCCAGCAGCAATTGCAACAACAGCAGTCTCACACAATCAACGCTTCCGCAGCCGCTGCAACCTCAGGATCTGGGAGTAGCGGCCTGACCATGCGACACAATAATAGCGGGAGACCGGGGTCTCTGAGCGCCTTGGCTGTGTCCATTGAAACTGATGTTGCTGGGAGACGGCTGTTTGCGGTAGTTA
shaker_Frag1695-1845_insertion1818	TTCGTACCATTCTATGGCGTCTCCTAAGCAGCAGCAACAACAAACCCAGCAGCAATTGCAACAACAGCAGTCTCACACAATCAACGCTTCCGCAGCCGCTGCAACCTCAGGATCTGGGAGTAGCGGCCTGACCATGCGACACAATAATGCCAGCGGGAGACCGGGGTCTCTGAGCTTGGCTGTGTCCATTGAAACTGATGTTGCTGGGAGACGGCTGTTTGCGGTAGTTA
shaker_Frag1695-1845_insertion1821	TTCGTACCATTCTATGGCGTCTCCTAAGCAGCAGCAACAACAAACCCAGCAGCAATTGCAACAACAGCAGTCTCACACAATCAACGCTTCCGCAGCCGCTGCAACCTCAGGATCTGGGAGTAGCGGCCTGACCATGCGACACAATAATGCCTTGAGCGGGAGACCGGGGTCTCTGAGCGCTGTGTCCATTGAAACTGATGTTGCTGGGAGACGGCTGTTTGCGGTAGTTA
shaker_Frag1695-1845_insertion1824	TTCGTACCATTCTATGGCGTCTCCTAAGCAGCAGCAACAACAAACCCAGCAGCAATTGCAACAACAGCAGTCTCACACAATCAACGCTTCCGCAGCCGCTGCAACCTCAGGATCTGGGAGTAGCGGCCTGACCATGCGACACAATAATGCCTTGGCTAGCGGGAGACCGGGGTCTCTGAGCGTGTCCATTGAAACTGATGTTGCTGGGAGACGGCTGTTTGCGGTAGTTA
shaker_Frag1695-1845_insertion1827	TTCGTACCATTCTATGGCGTCTCCTAAGCAGCAGCAACAACAAACCCAGCAGCAATTGCAACAACAGCAGTCTCACACAATCAACGCTTCCGCAGCCGCTGCAACCTCAGGATCTGGGAGTAGCGGCCTGACCATGCGACACAATAATGCCTTGGCTGTGAGCGGGAGACCGGGGTCTCTGAGCTCCATTGAAACTGATGTTGCTGGGAGACGGCTGTTTGCGGTAGTTA
shaker_Frag1695-1845_insertion1830	TTCGTACCATTCTATGGCGTCTCCTAAGCAGCAGCAACAACAAACCCAGCAGCAATTGCAACAACAGCAGTCTCACACAATCAACGCTTCCGCAGCCGCTGCAACCTCAGGATCTGGGAGTAGCGGCCTGACCATGCGACACAATAATGCCTTGGCTGTGTCCAGCGGGAGACCGGGGTCTCTGAGCATTGAAACTGATGTTGCTGGGAGACGGCTGTTTGCGGTAGTTA
shaker_Frag1695-1845_insertion1833	TTCGTACCATTCTATGGCGTCTCCTAAGCAGCAGCAACAACAAACCCAGCAGCAATTGCAACAACAGCAGTCTCACACAATCAACGCTTCCGCAGCCGCTGCAACCTCAGGATCTGGGAGTAGCGGCCTGACCATGCGACACAATAATGCCTTGGCTGTGTCCATTAGCGGGAGACCGGGGTCTCTGAGCGAAACTGATGTTGCTGGGAGACGGCTGTTTGCGGTAGTTA
shaker_Frag1695-1845_insertion1836	TTCGTACCATTCTATGGCGTCTCCTAAGCAGCAGCAACAACAAACCCAGCAGCAATTGCAACAACAGCAGTCTCACACAATCAACGCTTCCGCAGCCGCTGCAACCTCAGGATCTGGGAGTAGCGGCCTGACCATGCGACACAATAATGCCTTGGCTGTGTCCATTGAAAGCGGGAGACCGGGGTCTCTGAGCACTGATGTTGCTGGGAGACGGCTGTTTGCGGTAGTTA
shaker_Frag1695-1845_insertion1839	TTCGTACCATTCTATGGCGTCTCCTAAGCAGCAGCAACAACAAACCCAGCAGCAATTGCAACAACAGCAGTCTCACACAATCAACGCTTCCGCAGCCGCTGCAACCTCAGGATCTGGGAGTAGCGGCCTGACCATGCGACACAATAATGCCTTGGCTGTGTCCATTGAAACTAGCGGGAGACCGGGGTCTCTGAGCGATGTTGCTGGGAGACGGCTGTTTGCGGTAGTTA
shaker_Frag1695-1845_insertion1842	TTCGTACCATTCTATGGCGTCTCCTAAGCAGCAGCAACAACAAACCCAGCAGCAATTGCAACAACAGCAGTCTCACACAATCAACGCTTCCGCAGCCGCTGCAACCTCAGGATCTGGGAGTAGCGGCCTGACCATGCGACACAATAATGCCTTGGCTGTGTCCATTGAAACTGATAGCGGGAGACCGGGGTCTCTGAGCGTTGCTGGGAGACGGCTGTTTGCGGTAGTTA
shaker_Frag1695-1845_insertion1845	TTCGTACCATTCTATGGCGTCTCCTAAGCAGCAGCAACAACAAACCCAGCAGCAATTGCAACAACAGCAGTCTCACACAATCAACGCTTCCGCAGCCGCTGCAACCTCAGGATCTGGGAGTAGCGGCCTGACCATGCGACACAATAATGCCTTGGCTGTGTCCATTGAAACTGATGTTAGCGGGAGACCGGGGTCTCTGAGCGCTGGGAGACGGCTGTTTGCGGTAGTTA
asic1a_Frag3-165_insertion3	AACGATCACCACGTCTCCCATGAGCGGGAGACCGGGGTCTCTGAGCGAATTGAAAGCCGAAGAGGAAGAGGTCGGAGGTGTTCAACCAGTTTCTATCCAAGCATTCGCCTCAAGTTCCACTTTGCACGGGTTGGCACACATATTCTCTTACGAACGCCTCAGCCTCAAACGAGCTCTTTGGGCTCTTTGCTTTCTGGGGTCACTTGCTGTTCGGAGACGAGTTAGGGAAT
asic1a_Frag3-165_insertion6	AACGATCACCACGTCTCCCATGGAAAGCGGGAGACCGGGGTCTCTGAGCTTGAAAGCCGAAGAGGAAGAGGTCGGAGGTGTTCAACCAGTTTCTATCCAAGCATTCGCCTCAAGTTCCACTTTGCACGGGTTGGCACACATATTCTCTTACGAACGCCTCAGCCTCAAACGAGCTCTTTGGGCTCTTTGCTTTCTGGGGTCACTTGCTGTTCGGAGACGAGTTAGGGAAT
asic1a_Frag3-165_insertion9	AACGATCACCACGTCTCCCATGGAATTGAGCGGGAGACCGGGGTCTCTGAGCAAAGCCGAAGAGGAAGAGGTCGGAGGTGTTCAACCAGTTTCTATCCAAGCATTCGCCTCAAGTTCCACTTTGCACGGGTTGGCACACATATTCTCTTACGAACGCCTCAGCCTCAAACGAGCTCTTTGGGCTCTTTGCTTTCTGGGGTCACTTGCTGTTCGGAGACGAGTTAGGGAAT
asic1a_Frag3-165_insertion12	AACGATCACCACGTCTCCCATGGAATTGAAAAGCGGGAGACCGGGGTCTCTGAGCGCCGAAGAGGAAGAGGTCGGAGGTGTTCAACCAGTTTCTATCCAAGCATTCGCCTCAAGTTCCACTTTGCACGGGTTGGCACACATATTCTCTTACGAACGCCTCAGCCTCAAACGAGCTCTTTGGGCTCTTTGCTTTCTGGGGTCACTTGCTGTTCGGAGACGAGTTAGGGAAT

asic1a_Frag3-165_insertion15	AACGATCACCACGTCTCCCATGGAATTGAAAGCCAGCGGGAGACCGGGGTCTCTGAGCGAAGAGGAAGAGGTCGGAGGTGTTCAACCAGTTTCTATCCAAGCATTCGCCTCAAGTTCCACTTTGCACGGGTTGGCACACATATTCTCTTACGAACGCCTCAGCCTCAAACGAGCTCTTTGGGCTCTTTGCTTTCTGGGGTCACTTGCTGTTCGGAGACGAGTTAGGGAAT
asic1a_Frag3-165_insertion18	AACGATCACCACGTCTCCCATGGAATTGAAAGCCGAAAGCGGGAGACCGGGGTCTCTGAGCGAGGAAGAGGTCGGAGGTGTTCAACCAGTTTCTATCCAAGCATTCGCCTCAAGTTCCACTTTGCACGGGTTGGCACACATATTCTCTTACGAACGCCTCAGCCTCAAACGAGCTCTTTGGGCTCTTTGCTTTCTGGGGTCACTTGCTGTTCGGAGACGAGTTAGGGAAT
asic1a_Frag3-165_insertion21	AACGATCACCACGTCTCCCATGGAATTGAAAGCCGAAGAGAGCGGGAGACCGGGGTCTCTGAGCGAAGAGGTCGGAGGTGTTCAACCAGTTTCTATCCAAGCATTCGCCTCAAGTTCCACTTTGCACGGGTTGGCACACATATTCTCTTACGAACGCCTCAGCCTCAAACGAGCTCTTTGGGCTCTTTGCTTTCTGGGGTCACTTGCTGTTCGGAGACGAGTTAGGGAAT
asic1a_Frag3-165_insertion24	AACGATCACCACGTCTCCCATGGAATTGAAAGCCGAAGAGGAAAGCGGGAGACCGGGGTCTCTGAGCGAGGTCGGAGGTGTTCAACCAGTTTCTATCCAAGCATTCGCCTCAAGTTCCACTTTGCACGGGTTGGCACACATATTCTCTTACGAACGCCTCAGCCTCAAACGAGCTCTTTGGGCTCTTTGCTTTCTGGGGTCACTTGCTGTTCGGAGACGAGTTAGGGAAT
asic1a_Frag3-165_insertion27	AACGATCACCACGTCTCCCATGGAATTGAAAGCCGAAGAGGAAGAGAGCGGGAGACCGGGGTCTCTGAGCGTCGGAGGTGTTCAACCAGTTTCTATCCAAGCATTCGCCTCAAGTTCCACTTTGCACGGGTTGGCACACATATTCTCTTACGAACGCCTCAGCCTCAAACGAGCTCTTTGGGCTCTTTGCTTTCTGGGGTCACTTGCTGTTCGGAGACGAGTTAGGGAAT
asic1a_Frag3-165_insertion30	AACGATCACCACGTCTCCCATGGAATTGAAAGCCGAAGAGGAAGAGGTCAGCGGGAGACCGGGGTCTCTGAGCGGAGGTGTTCAACCAGTTTCTATCCAAGCATTCGCCTCAAGTTCCACTTTGCACGGGTTGGCACACATATTCTCTTACGAACGCCTCAGCCTCAAACGAGCTCTTTGGGCTCTTTGCTTTCTGGGGTCACTTGCTGTTCGGAGACGAGTTAGGGAAT
asic1a_Frag3-165_insertion33	AACGATCACCACGTCTCCCATGGAATTGAAAGCCGAAGAGGAAGAGGTCGGAAGCGGGAGACCGGGGTCTCTGAGCGGTGTTCAACCAGTTTCTATCCAAGCATTCGCCTCAAGTTCCACTTTGCACGGGTTGGCACACATATTCTCTTACGAACGCCTCAGCCTCAAACGAGCTCTTTGGGCTCTTTGCTTTCTGGGGTCACTTGCTGTTCGGAGACGAGTTAGGGAAT
asic1a_Frag3-165_insertion36	AACGATCACCACGTCTCCCATGGAATTGAAAGCCGAAGAGGAAGAGGTCGGAGGTAGCGGGAGACCGGGGTCTCTGAGCGTTCAACCAGTTTCTATCCAAGCATTCGCCTCAAGTTCCACTTTGCACGGGTTGGCACACATATTCTCTTACGAACGCCTCAGCCTCAAACGAGCTCTTTGGGCTCTTTGCTTTCTGGGGTCACTTGCTGTTCGGAGACGAGTTAGGGAAT
asic1a_Frag3-165_insertion39	AACGATCACCACGTCTCCCATGGAATTGAAAGCCGAAGAGGAAGAGGTCGGAGGTGTTAGCGGGAGACCGGGGTCTCTGAGCCAACCAGTTTCTATCCAAGCATTCGCCTCAAGTTCCACTTTGCACGGGTTGGCACACATATTCTCTTACGAACGCCTCAGCCTCAAACGAGCTCTTTGGGCTCTTTGCTTTCTGGGGTCACTTGCTGTTCGGAGACGAGTTAGGGAAT
asic1a_Frag3-165_insertion42	AACGATCACCACGTCTCCCATGGAATTGAAAGCCGAAGAGGAAGAGGTCGGAGGTGTTCAAAGCGGGAGACCGGGGTCTCTGAGCCCAGTTTCTATCCAAGCATTCGCCTCAAGTTCCACTTTGCACGGGTTGGCACACATATTCTCTTACGAACGCCTCAGCCTCAAACGAGCTCTTTGGGCTCTTTGCTTTCTGGGGTCACTTGCTGTTCGGAGACGAGTTAGGGAAT
asic1a_Frag3-165_insertion45	AACGATCACCACGTCTCCCATGGAATTGAAAGCCGAAGAGGAAGAGGTCGGAGGTGTTCAACCAAGCGGGAGACCGGGGTCTCTGAGCGTTTCTATCCAAGCATTCGCCTCAAGTTCCACTTTGCACGGGTTGGCACACATATTCTCTTACGAACGCCTCAGCCTCAAACGAGCTCTTTGGGCTCTTTGCTTTCTGGGGTCACTTGCTGTTCGGAGACGAGTTAGGGAAT
asic1a_Frag3-165_insertion48	AACGATCACCACGTCTCCCATGGAATTGAAAGCCGAAGAGGAAGAGGTCGGAGGTGTTCAACCAGTTAGCGGGAGACCGGGGTCTCTGAGCTCTATCCAAGCATTCGCCTCAAGTTCCACTTTGCACGGGTTGGCACACATATTCTCTTACGAACGCCTCAGCCTCAAACGAGCTCTTTGGGCTCTTTGCTTTCTGGGGTCACTTGCTGTTCGGAGACGAGTTAGGGAAT
asic1a_Frag3-165_insertion51	AACGATCACCACGTCTCCCATGGAATTGAAAGCCGAAGAGGAAGAGGTCGGAGGTGTTCAACCAGTTTCTAGCGGGAGACCGGGGTCTCTGAGCATCCAAGCATTCGCCTCAAGTTCCACTTTGCACGGGTTGGCACACATATTCTCTTACGAACGCCTCAGCCTCAAACGAGCTCTTTGGGCTCTTTGCTTTCTGGGGTCACTTGCTGTTCGGAGACGAGTTAGGGAAT
asic1a_Frag3-165_insertion54	AACGATCACCACGTCTCCCATGGAATTGAAAGCCGAAGAGGAAGAGGTCGGAGGTGTTCAACCAGTTTCTATCAGCGGGAGACCGGGGTCTCTGAGCCAAGCATTCGCCTCAAGTTCCACTTTGCACGGGTTGGCACACATATTCTCTTACGAACGCCTCAGCCTCAAACGAGCTCTTTGGGCTCTTTGCTTTCTGGGGTCACTTGCTGTTCGGAGACGAGTTAGGGAAT
asic1a_Frag3-165_insertion57	AACGATCACCACGTCTCCCATGGAATTGAAAGCCGAAGAGGAAGAGGTCGGAGGTGTTCAACCAGTTTCTATCCAAAGCGGGAGACCGGGGTCTCTGAGCGCATTCGCCTCAAGTTCCACTTTGCACGGGTTGGCACACATATTCTCTTACGAACGCCTCAGCCTCAAACGAGCTCTTTGGGCTCTTTGCTTTCTGGGGTCACTTGCTGTTCGGAGACGAGTTAGGGAAT
asic1a_Frag3-165_insertion60	AACGATCACCACGTCTCCCATGGAATTGAAAGCCGAAGAGGAAGAGGTCGGAGGTGTTCAACCAGTTTCTATCCAAGCAAGCGGGAGACCGGGGTCTCTGAGCTTCGCCTCAAGTTCCACTTTGCACGGGTTGGCACACATATTCTCTTACGAACGCCTCAGCCTCAAACGAGCTCTTTGGGCTCTTTGCTTTCTGGGGTCACTTGCTGTTCGGAGACGAGTTAGGGAAT
asic1a_Frag3-165_insertion63	AACGATCACCACGTCTCCCATGGAATTGAAAGCCGAAGAGGAAGAGGTCGGAGGTGTTCAACCAGTTTCTATCCAAGCATTCAGCGGGAGACCGGGGTCTCTGAGCGCCTCAAGTTCCACTTTGCACGGGTTGGCACACATATTCTCTTACGAACGCCTCAGCCTCAAACGAGCTCTTTGGGCTCTTTGCTTTCTGGGGTCACTTGCTGTTCGGAGACGAGTTAGGGAAT
asic1a_Frag3-165_insertion66	AACGATCACCACGTCTCCCATGGAATTGAAAGCCGAAGAGGAAGAGGTCGGAGGTGTTCAACCAGTTTCTATCCAAGCATTCGCCAGCGGGAGACCGGGGTCTCTGAGCTCAAGTTCCACTTTGCACGGGTTGGCACACATATTCTCTTACGAACGCCTCAGCCTCAAACGAGCTCTTTGGGCTCTTTGCTTTCTGGGGTCACTTGCTGTTCGGAGACGAGTTAGGGAAT
asic1a_Frag3-165_insertion69	AACGATCACCACGTCTCCCATGGAATTGAAAGCCGAAGAGGAAGAGGTCGGAGGTGTTCAACCAGTTTCTATCCAAGCATTCGCCTCAAGCGGGAGACCGGGGTCTCTGAGCAGTTCCACTTTGCACGGGTTGGCACACATATTCTCTTACGAACGCCTCAGCCTCAAACGAGCTCTTTGGGCTCTTTGCTTTCTGGGGTCACTTGCTGTTCGGAGACGAGTTAGGGAAT
asic1a_Frag3-165_insertion72	AACGATCACCACGTCTCCCATGGAATTGAAAGCCGAAGAGGAAGAGGTCGGAGGTGTTCAACCAGTTTCTATCCAAGCATTCGCCTCAAGTAGCGGGAGACCGGGGTCTCTGAGCTCCACTTTGCACGGGTTGGCACACATATTCTCTTACGAACGCCTCAGCCTCAAACGAGCTCTTTGGGCTCTTTGCTTTCTGGGGTCACTTGCTGTTCGGAGACGAGTTAGGGAAT
asic1a_Frag3-165_insertion75	AACGATCACCACGTCTCCCATGGAATTGAAAGCCGAAGAGGAAGAGGTCGGAGGTGTTCAACCAGTTTCTATCCAAGCATTCGCCTCAAGTTCCAGCGGGAGACCGGGGTCTCTGAGCACTTTGCACGGGTTGGCACACATATTCTCTTACGAACGCCTCAGCCTCAAACGAGCTCTTTGGGCTCTTTGCTTTCTGGGGTCACTTGCTGTTCGGAGACGAGTTAGGGAAT
asic1a_Frag3-165_insertion78	AACGATCACCACGTCTCCCATGGAATTGAAAGCCGAAGAGGAAGAGGTCGGAGGTGTTCAACCAGTTTCTATCCAAGCATTCGCCTCAAGTTCCACTAGCGGGAGACCGGGGTCTCTGAGCTTGCACGGGTTGGCACACATATTCTCTTACGAACGCCTCAGCCTCAAACGAGCTCTTTGGGCTCTTTGCTTTCTGGGGTCACTTGCTGTTCGGAGACGAGTTAGGGAAT
asic1a_Frag3-165_insertion81	AACGATCACCACGTCTCCCATGGAATTGAAAGCCGAAGAGGAAGAGGTCGGAGGTGTTCAACCAGTTTCTATCCAAGCATTCGCCTCAAGTTCCACTTTGAGCGGGAGACCGGGGTCTCTGAGCCACGGGTTGGCACACATATTCTCTTACGAACGCCTCAGCCTCAAACGAGCTCTTTGGGCTCTTTGCTTTCTGGGGTCACTTGCTGTTCGGAGACGAGTTAGGGAAT
asic1a_Frag3-165_insertion84	AACGATCACCACGTCTCCCATGGAATTGAAAGCCGAAGAGGAAGAGGTCGGAGGTGTTCAACCAGTTTCTATCCAAGCATTCGCCTCAAGTTCCACTTTGCACAGCGGGAGACCGGGGTCTCTGAGCGGGTTGGCACACATATTCTCTTACGAACGCCTCAGCCTCAAACGAGCTCTTTGGGCTCTTTGCTTTCTGGGGTCACTTGCTGTTCGGAGACGAGTTAGGGAAT
asic1a_Frag3-165_insertion87	AACGATCACCACGTCTCCCATGGAATTGAAAGCCGAAGAGGAAGAGGTCGGAGGTGTTCAACCAGTTTCTATCCAAGCATTCGCCTCAAGTTCCACTTTGCACGGGAGCGGGAGACCGGGGTCTCTGAGCTTGGCACACATATTCTCTTACGAACGCCTCAGCCTCAAACGAGCTCTTTGGGCTCTTTGCTTTCTGGGGTCACTTGCTGTTCGGAGACGAGTTAGGGAAT
asic1a_Frag3-165_insertion90	AACGATCACCACGTCTCCCATGGAATTGAAAGCCGAAGAGGAAGAGGTCGGAGGTGTTCAACCAGTTTCTATCCAAGCATTCGCCTCAAGTTCCACTTTGCACGGGTTGAGCGGGAGACCGGGGTCTCTGAGCGCACACATATTCTCTTACGAACGCCTCAGCCTCAAACGAGCTCTTTGGGCTCTTTGCTTTCTGGGGTCACTTGCTGTTCGGAGACGAGTTAGGGAAT
asic1a_Frag3-165_insertion93	AACGATCACCACGTCTCCCATGGAATTGAAAGCCGAAGAGGAAGAGGTCGGAGGTGTTCAACCAGTTTCTATCCAAGCATTCGCCTCAAGTTCCACTTTGCACGGGTTGGCAAGCGGGAGACCGGGGTCTCTGAGCCACATATTCTCTTACGAACGCCTCAGCCTCAAACGAGCTCTTTGGGCTCTTTGCTTTCTGGGGTCACTTGCTGTTCGGAGACGAGTTAGGGAAT
asic1a_Frag3-165_insertion96	AACGATCACCACGTCTCCCATGGAATTGAAAGCCGAAGAGGAAGAGGTCGGAGGTGTTCAACCAGTTTCTATCCAAGCATTCGCCTCAAGTTCCACTTTGCACGGGTTGGCACACAGCGGGAGACCGGGGTCTCTGAGCATATTCTCTTACGAACGCCTCAGCCTCAAACGAGCTCTTTGGGCTCTTTGCTTTCTGGGGTCACTTGCTGTTCGGAGACGAGTTAGGGAAT
asic1a_Frag3-165_insertion99	AACGATCACCACGTCTCCCATGGAATTGAAAGCCGAAGAGGAAGAGGTCGGAGGTGTTCAACCAGTTTCTATCCAAGCATTCGCCTCAAGTTCCACTTTGCACGGGTTGGCACACATAAGCGGGAGACCGGGGTCTCTGAGCTTCTCTTACGAACGCCTCAGCCTCAAACGAGCTCTTTGGGCTCTTTGCTTTCTGGGGTCACTTGCTGTTCGGAGACGAGTTAGGGAAT
asic1a_Frag3-165_insertion102	AACGATCACCACGTCTCCCATGGAATTGAAAGCCGAAGAGGAAGAGGTCGGAGGTGTTCAACCAGTTTCTATCCAAGCATTCGCCTCAAGTTCCACTTTGCACGGGTTGGCACACATATTCAGCGGGAGACCGGGGTCTCTGAGCTCTTACGAACGCCTCAGCCTCAAACGAGCTCTTTGGGCTCTTTGCTTTCTGGGGTCACTTGCTGTTCGGAGACGAGTTAGGGAAT
asic1a_Frag3-165_insertion105	AACGATCACCACGTCTCCCATGGAATTGAAAGCCGAAGAGGAAGAGGTCGGAGGTGTTCAACCAGTTTCTATCCAAGCATTCGCCTCAAGTTCCACTTTGCACGGGTTGGCACACATATTCTCTAGCGGGAGACCGGGGTCTCTGAGCTACGAACGCCTCAGCCTCAAACGAGCTCTTTGGGCTCTTTGCTTTCTGGGGTCACTTGCTGTTCGGAGACGAGTTAGGGAAT
asic1a_Frag3-165_insertion108	AACGATCACCACGTCTCCCATGGAATTGAAAGCCGAAGAGGAAGAGGTCGGAGGTGTTCAACCAGTTTCTATCCAAGCATTCGCCTCAAGTTCCACTTTGCACGGGTTGGCACACATATTCTCTTACAGCGGGAGACCGGGGTCTCTGAGCGAACGCCTCAGCCTCAAACGAGCTCTTTGGGCTCTTTGCTTTCTGGGGTCACTTGCTGTTCGGAGACGAGTTAGGGAAT
asic1a_Frag3-165_insertion111	AACGATCACCACGTCTCCCATGGAATTGAAAGCCGAAGAGGAAGAGGTCGGAGGTGTTCAACCAGTTTCTATCCAAGCATTCGCCTCAAGTTCCACTTTGCACGGGTTGGCACACATATTCTCTTACGAAAGCGGGAGACCGGGGTCTCTGAGCCGCCTCAGCCTCAAACGAGCTCTTTGGGCTCTTTGCTTTCTGGGGTCACTTGCTGTTCGGAGACGAGTTAGGGAAT
asic1a_Frag3-165_insertion114	AACGATCACCACGTCTCCCATGGAATTGAAAGCCGAAGAGGAAGAGGTCGGAGGTGTTCAACCAGTTTCTATCCAAGCATTCGCCTCAAGTTCCACTTTGCACGGGTTGGCACACATATTCTCTTACGAACGCAGCGGGAGACCGGGGTCTCTGAGCCTCAGCCTCAAACGAGCTCTTTGGGCTCTTTGCTTTCTGGGGTCACTTGCTGTTCGGAGACGAGTTAGGGAAT
asic1a_Frag3-165_insertion117	AACGATCACCACGTCTCCCATGGAATTGAAAGCCGAAGAGGAAGAGGTCGGAGGTGTTCAACCAGTTTCTATCCAAGCATTCGCCTCAAGTTCCACTTTGCACGGGTTGGCACACATATTCTCTTACGAACGCCTCAGCGGGAGACCGGGGTCTCTGAGCAGCCTCAAACGAGCTCTTTGGGCTCTTTGCTTTCTGGGGTCACTTGCTGTTCGGAGACGAGTTAGGGAAT
asic1a_Frag3-165_insertion120	AACGATCACCACGTCTCCCATGGAATTGAAAGCCGAAGAGGAAGAGGTCGGAGGTGTTCAACCAGTTTCTATCCAAGCATTCGCCTCAAGTTCCACTTTGCACGGGTTGGCACACATATTCTCTTACGAACGCCTCAGCAGCGGGAGACCGGGGTCTCTGAGCCTCAAACGAGCTCTTTGGGCTCTTTGCTTTCTGGGGTCACTTGCTGTTCGGAGACGAGTTAGGGAAT
asic1a_Frag3-165_insertion123	AACGATCACCACGTCTCCCATGGAATTGAAAGCCGAAGAGGAAGAGGTCGGAGGTGTTCAACCAGTTTCTATCCAAGCATTCGCCTCAAGTTCCACTTTGCACGGGTTGGCACACATATTCTCTTACGAACGCCTCAGCCTCAGCGGGAGACCGGGGTCTCTGAGCAAACGAGCTCTTTGGGCTCTTTGCTTTCTGGGGTCACTTGCTGTTCGGAGACGAGTTAGGGAAT
asic1a_Frag3-165_insertion126	AACGATCACCACGTCTCCCATGGAATTGAAAGCCGAAGAGGAAGAGGTCGGAGGTGTTCAACCAGTTTCTATCCAAGCATTCGCCTCAAGTTCCACTTTGCACGGGTTGGCACACATATTCTCTTACGAACGCCTCAGCCTCAAAAGCGGGAGACCGGGGTCTCTGAGCCGAGCTCTTTGGGCTCTTTGCTTTCTGGGGTCACTTGCTGTTCGGAGACGAGTTAGGGAAT
asic1a_Frag3-165_insertion129	AACGATCACCACGTCTCCCATGGAATTGAAAGCCGAAGAGGAAGAGGTCGGAGGTGTTCAACCAGTTTCTATCCAAGCATTCGCCTCAAGTTCCACTTTGCACGGGTTGGCACACATATTCTCTTACGAACGCCTCAGCCTCAAACGAAGCGGGAGACCGGGGTCTCTGAGCGCTCTTTGGGCTCTTTGCTTTCTGGGGTCACTTGCTGTTCGGAGACGAGTTAGGGAAT
asic1a_Frag3-165_insertion132	AACGATCACCACGTCTCCCATGGAATTGAAAGCCGAAGAGGAAGAGGTCGGAGGTGTTCAACCAGTTTCTATCCAAGCATTCGCCTCAAGTTCCACTTTGCACGGGTTGGCACACATATTCTCTTACGAACGCCTCAGCCTCAAACGAGCTAGCGGGAGACCGGGGTCTCTGAGCCTTTGGGCTCTTTGCTTTCTGGGGTCACTTGCTGTTCGGAGACGAGTTAGGGAAT
asic1a_Frag3-165_insertion135	AACGATCACCACGTCTCCCATGGAATTGAAAGCCGAAGAGGAAGAGGTCGGAGGTGTTCAACCAGTTTCTATCCAAGCATTCGCCTCAAGTTCCACTTTGCACGGGTTGGCACACATATTCTCTTACGAACGCCTCAGCCTCAAACGAGCTCTTAGCGGGAGACCGGGGTCTCTGAGCTGGGCTCTTTGCTTTCTGGGGTCACTTGCTGTTCGGAGACGAGTTAGGGAAT
asic1a_Frag3-165_insertion138	AACGATCACCACGTCTCCCATGGAATTGAAAGCCGAAGAGGAAGAGGTCGGAGGTGTTCAACCAGTTTCTATCCAAGCATTCGCCTCAAGTTCCACTTTGCACGGGTTGGCACACATATTCTCTTACGAACGCCTCAGCCTCAAACGAGCTCTTTGGAGCGGGAGACCGGGGTCTCTGAGCGCTCTTTGCTTTCTGGGGTCACTTGCTGTTCGGAGACGAGTTAGGGAAT
asic1a_Frag3-165_insertion141	AACGATCACCACGTCTCCCATGGAATTGAAAGCCGAAGAGGAAGAGGTCGGAGGTGTTCAACCAGTTTCTATCCAAGCATTCGCCTCAAGTTCCACTTTGCACGGGTTGGCACACATATTCTCTTACGAACGCCTCAGCCTCAAACGAGCTCTTTGGGCTAGCGGGAGACCGGGGTCTCTGAGCCTTTGCTTTCTGGGGTCACTTGCTGTTCGGAGACGAGTTAGGGAAT
asic1a_Frag3-165_insertion144	AACGATCACCACGTCTCCCATGGAATTGAAAGCCGAAGAGGAAGAGGTCGGAGGTGTTCAACCAGTTTCTATCCAAGCATTCGCCTCAAGTTCCACTTTGCACGGGTTGGCACACATATTCTCTTACGAACGCCTCAGCCTCAAACGAGCTCTTTGGGCTCTTAGCGGGAGACCGGGGTCTCTGAGCTGCTTTCTGGGGTCACTTGCTGTTCGGAGACGAGTTAGGGAAT
asic1a_Frag3-165_insertion147	AACGATCACCACGTCTCCCATGGAATTGAAAGCCGAAGAGGAAGAGGTCGGAGGTGTTCAACCAGTTTCTATCCAAGCATTCGCCTCAAGTTCCACTTTGCACGGGTTGGCACACATATTCTCTTACGAACGCCTCAGCCTCAAACGAGCTCTTTGGGCTCTTTGCAGCGGGAGACCGGGGTCTCTGAGCTTTCTGGGGTCACTTGCTGTTCGGAGACGAGTTAGGGAAT
asic1a_Frag3-165_insertion150	AACGATCACCACGTCTCCCATGGAATTGAAAGCCGAAGAGGAAGAGGTCGGAGGTGTTCAACCAGTTTCTATCCAAGCATTCGCCTCAAGTTCCACTTTGCACGGGTTGGCACACATATTCTCTTACGAACGCCTCAGCCTCAAACGAGCTCTTTGGGCTCTTTGCTTTAGCGGGAGACCGGGGTCTCTGAGCCTGGGGTCACTTGCTGTTCGGAGACGAGTTAGGGAAT
asic1a_Frag3-165_insertion153	AACGATCACCACGTCTCCCATGGAATTGAAAGCCGAAGAGGAAGAGGTCGGAGGTGTTCAACCAGTTTCTATCCAAGCATTCGCCTCAAGTTCCACTTTGCACGGGTTGGCACACATATTCTCTTACGAACGCCTCAGCCTCAAACGAGCTCTTTGGGCTCTTTGCTTTCTGAGCGGGAGACCGGGGTCTCTGAGCGGGTCACTTGCTGTTCGGAGACGAGTTAGGGAAT
asic1a_Frag3-165_insertion156	AACGATCACCACGTCTCCCATGGAATTGAAAGCCGAAGAGGAAGAGGTCGGAGGTGTTCAACCAGTTTCTATCCAAGCATTCGCCTCAAGTTCCACTTTGCACGGGTTGGCACACATATTCTCTTACGAACGCCTCAGCCTCAAACGAGCTCTTTGGGCTCTTTGCTTTCTGGGGAGCGGGAGACCGGGGTCTCTGAGCTCACTTGCTGTTCGGAGACGAGTTAGGGAAT
asic1a_Frag3-165_insertion159	AACGATCACCACGTCTCCCATGGAATTGAAAGCCGAAGAGGAAGAGGTCGGAGGTGTTCAACCAGTTTCTATCCAAGCATTCGCCTCAAGTTCCACTTTGCACGGGTTGGCACACATATTCTCTTACGAACGCCTCAGCCTCAAACGAGCTCTTTGGGCTCTTTGCTTTCTGGGGTCAAGCGGGAGACCGGGGTCTCTGAGCCTTGCTGTTCGGAGACGAGTTAGGGAAT
asic1a_Frag3-165_insertion162	AACGATCACCACGTCTCCCATGGAATTGAAAGCCGAAGAGGAAGAGGTCGGAGGTGTTCAACCAGTTTCTATCCAAGCATTCGCCTCAAGTTCCACTTTGCACGGGTTGGCACACATATTCTCTTACGAACGCCTCAGCCTCAAACGAGCTCTTTGGGCTCTTTGCTTTCTGGGGTCACTTAGCGGGAGACCGGGGTCTCTGAGCGCTGTTCGGAGACGAGTTAGGGAAT
asic1a_Frag3-165_insertion165	AACGATCACCACGTCTCCCATGGAATTGAAAGCCGAAGAGGAAGAGGTCGGAGGTGTTCAACCAGTTTCTATCCAAGCATTCGCCTCAAGTTCCACTTTGCACGGGTTGGCACACATATTCTCTTACGAACGCCTCAGCCTCAAACGAGCTCTTTGGGCTCTTTGCTTTCTGGGGTCACTTGCTAGCGGGAGACCGGGGTCTCTGAGCGTTCGGAGACGAGTTAGGGAAT
asic1a_Frag168-327_insertion168	TAGATACTAGCTCGTCTCCTGTTAGCGGGAGACCGGGGTCTCTGAGCCTTCTTTGCGTTTGTACCGAAAGGGTTCAGTATTATTTCCATTATCATCATGTTACTAAACTCGACGAAGTCGCCGCATCACAGTTGACCTTCCCTGCTGTAACCCTTTGCAATCTGAACGAATTTAGATTTAGCCAAGTTTCTAAAAACGATCTCTATCACGGGAGACGGCGGCCAAACACT
asic1a_Frag168-327_insertion171	TAGATACTAGCTCGTCTCCTGTTCTTAGCGGGAGACCGGGGTCTCTGAGCCTTTGCGTTTGTACCGAAAGGGTTCAGTATTATTTCCATTATCATCATGTTACTAAACTCGACGAAGTCGCCGCATCACAGTTGACCTTCCCTGCTGTAACCCTTTGCAATCTGAACGAATTTAGATTTAGCCAAGTTTCTAAAAACGATCTCTATCACGGGAGACGGCGGCCAAACACT
asic1a_Frag168-327_insertion174	TAGATACTAGCTCGTCTCCTGTTCTTCTTAGCGGGAGACCGGGGTCTCTGAGCTGCGTTTGTACCGAAAGGGTTCAGTATTATTTCCATTATCATCATGTTACTAAACTCGACGAAGTCGCCGCATCACAGTTGACCTTCCCTGCTGTAACCCTTTGCAATCTGAACGAATTTAGATTTAGCCAAGTTTCTAAAAACGATCTCTATCACGGGAGACGGCGGCCAAACACT
asic1a_Frag168-327_insertion177	TAGATACTAGCTCGTCTCCTGTTCTTCTTTGCAGCGGGAGACCGGGGTCTCTGAGCGTTTGTACCGAAAGGGTTCAGTATTATTTCCATTATCATCATGTTACTAAACTCGACGAAGTCGCCGCATCACAGTTGACCTTCCCTGCTGTAACCCTTTGCAATCTGAACGAATTTAGATTTAGCCAAGTTTCTAAAAACGATCTCTATCACGGGAGACGGCGGCCAAACACT
asic1a_Frag168-327_insertion180	TAGATACTAGCTCGTCTCCTGTTCTTCTTTGCGTTAGCGGGAGACCGGGGTCTCTGAGCTGTACCGAAAGGGTTCAGTATTATTTCCATTATCATCATGTTACTAAACTCGACGAAGTCGCCGCATCACAGTTGACCTTCCCTGCTGTAACCCTTTGCAATCTGAACGAATTTAGATTTAGCCAAGTTTCTAAAAACGATCTCTATCACGGGAGACGGCGGCCAAACACT
asic1a_Frag168-327_insertion183	TAGATACTAGCTCGTCTCCTGTTCTTCTTTGCGTTTGTAGCGGGAGACCGGGGTCTCTGAGCACCGAAAGGGTTCAGTATTATTTCCATTATCATCATGTTACTAAACTCGACGAAGTCGCCGCATCACAGTTGACCTTCCCTGCTGTAACCCTTTGCAATCTGAACGAATTTAGATTTAGCCAAGTTTCTAAAAACGATCTCTATCACGGGAGACGGCGGCCAAACACT
asic1a_Frag168-327_insertion186	TAGATACTAGCTCGTCTCCTGTTCTTCTTTGCGTTTGTACCAGCGGGAGACCGGGGTCTCTGAGCGAAAGGGTTCAGTATTATTTCCATTATCATCATGTTACTAAACTCGACGAAGTCGCCGCATCACAGTTGACCTTCCCTGCTGTAACCCTTTGCAATCTGAACGAATTTAGATTTAGCCAAGTTTCTAAAAACGATCTCTATCACGGGAGACGGCGGCCAAACACT
asic1a_Frag168-327_insertion189	TAGATACTAGCTCGTCTCCTGTTCTTCTTTGCGTTTGTACCGAAAGCGGGAGACCGGGGTCTCTGAGCAGGGTTCAGTATTATTTCCATTATCATCATGTTACTAAACTCGACGAAGTCGCCGCATCACAGTTGACCTTCCCTGCTGTAACCCTTTGCAATCTGAACGAATTTAGATTTAGCCAAGTTTCTAAAAACGATCTCTATCACGGGAGACGGCGGCCAAACACT
asic1a_Frag168-327_insertion192	TAGATACTAGCTCGTCTCCTGTTCTTCTTTGCGTTTGTACCGAAAGGAGCGGGAGACCGGGGTCTCTGAGCGTTCAGTATTATTTCCATTATCATCATGTTACTAAACTCGACGAAGTCGCCGCATCACAGTTGACCTTCCCTGCTGTAACCCTTTGCAATCTGAACGAATTTAGATTTAGCCAAGTTTCTAAAAACGATCTCTATCACGGGAGACGGCGGCCAAACACT
asic1a_Frag168-327_insertion195	TAGATACTAGCTCGTCTCCTGTTCTTCTTTGCGTTTGTACCGAAAGGGTTAGCGGGAGACCGGGGTCTCTGAGCCAGTATTATTTCCATTATCATCATGTTACTAAACTCGACGAAGTCGCCGCATCACAGTTGACCTTCCCTGCTGTAACCCTTTGCAATCTGAACGAATTTAGATTTAGCCAAGTTTCTAAAAACGATCTCTATCACGGGAGACGGCGGCCAAACACT
asic1a_Frag168-327_insertion198	TAGATACTAGCTCGTCTCCTGTTCTTCTTTGCGTTTGTACCGAAAGGGTTCAGAGCGGGAGACCGGGGTCTCTGAGCTATTATTTCCATTATCATCATGTTACTAAACTCGACGAAGTCGCCGCATCACAGTTGACCTTCCCTGCTGTAACCCTTTGCAATCTGAACGAATTTAGATTTAGCCAAGTTTCTAAAAACGATCTCTATCACGGGAGACGGCGGCCAAACACT
asic1a_Frag168-327_insertion201	TAGATACTAGCTCGTCTCCTGTTCTTCTTTGCGTTTGTACCGAAAGGGTTCAGTATAGCGGGAGACCGGGGTCTCTGAGCTATTTCCATTATCATCATGTTACTAAACTCGACGAAGTCGCCGCATCACAGTTGACCTTCCCTGCTGTAACCCTTTGCAATCTGAACGAATTTAGATTTAGCCAAGTTTCTAAAAACGATCTCTATCACGGGAGACGGCGGCCAAACACT
asic1a_Frag168-327_insertion204	TAGATACTAGCTCGTCTCCTGTTCTTCTTTGCGTTTGTACCGAAAGGGTTCAGTATTATAGCGGGAGACCGGGGTCTCTGAGCTTCCATTATCATCATGTTACTAAACTCGACGAAGTCGCCGCATCACAGTTGACCTTCCCTGCTGTAACCCTTTGCAATCTGAACGAATTTAGATTTAGCCAAGTTTCTAAAAACGATCTCTATCACGGGAGACGGCGGCCAAACACT
asic1a_Frag168-327_insertion207	TAGATACTAGCTCGTCTCCTGTTCTTCTTTGCGTTTGTACCGAAAGGGTTCAGTATTATTTCAGCGGGAGACCGGGGTCTCTGAGCCATTATCATCATGTTACTAAACTCGACGAAGTCGCCGCATCACAGTTGACCTTCCCTGCTGTAACCCTTTGCAATCTGAACGAATTTAGATTTAGCCAAGTTTCTAAAAACGATCTCTATCACGGGAGACGGCGGCCAAACACT
asic1a_Frag168-327_insertion210	TAGATACTAGCTCGTCTCCTGTTCTTCTTTGCGTTTGTACCGAAAGGGTTCAGTATTATTTCCATAGCGGGAGACCGGGGTCTCTGAGCTATCATCATGTTACTAAACTCGACGAAGTCGCCGCATCACAGTTGACCTTCCCTGCTGTAACCCTTTGCAATCTGAACGAATTTAGATTTAGCCAAGTTTCTAAAAACGATCTCTATCACGGGAGACGGCGGCCAAACACT
asic1a_Frag168-327_insertion213	TAGATACTAGCTCGTCTCCTGTTCTTCTTTGCGTTTGTACCGAAAGGGTTCAGTATTATTTCCATTATAGCGGGAGACCGGGGTCTCTGAGCCATCATGTTACTAAACTCGACGAAGTCGCCGCATCACAGTTGACCTTCCCTGCTGTAACCCTTTGCAATCTGAACGAATTTAGATTTAGCCAAGTTTCTAAAAACGATCTCTATCACGGGAGACGGCGGCCAAACACT
asic1a_Frag168-327_insertion216	TAGATACTAGCTCGTCTCCTGTTCTTCTTTGCGTTTGTACCGAAAGGGTTCAGTATTATTTCCATTATCATAGCGGGAGACCGGGGTCTCTGAGCCATGTTACTAAACTCGACGAAGTCGCCGCATCACAGTTGACCTTCCCTGCTGTAACCCTTTGCAATCTGAACGAATTTAGATTTAGCCAAGTTTCTAAAAACGATCTCTATCACGGGAGACGGCGGCCAAACACT
asic1a_Frag168-327_insertion219	TAGATACTAGCTCGTCTCCTGTTCTTCTTTGCGTTTGTACCGAAAGGGTTCAGTATTATTTCCATTATCATCATAGCGGGAGACCGGGGTCTCTGAGCGTTACTAAACTCGACGAAGTCGCCGCATCACAGTTGACCTTCCCTGCTGTAACCCTTTGCAATCTGAACGAATTTAGATTTAGCCAAGTTTCTAAAAACGATCTCTATCACGGGAGACGGCGGCCAAACACT
asic1a_Frag168-327_insertion222	TAGATACTAGCTCGTCTCCTGTTCTTCTTTGCGTTTGTACCGAAAGGGTTCAGTATTATTTCCATTATCATCATGTTAGCGGGAGACCGGGGTCTCTGAGCACTAAACTCGACGAAGTCGCCGCATCACAGTTGACCTTCCCTGCTGTAACCCTTTGCAATCTGAACGAATTTAGATTTAGCCAAGTTTCTAAAAACGATCTCTATCACGGGAGACGGCGGCCAAACACT
asic1a_Frag168-327_insertion225	TAGATACTAGCTCGTCTCCTGTTCTTCTTTGCGTTTGTACCGAAAGGGTTCAGTATTATTTCCATTATCATCATGTTACTAGCGGGAGACCGGGGTCTCTGAGCAAACTCGACGAAGTCGCCGCATCACAGTTGACCTTCCCTGCTGTAACCCTTTGCAATCTGAACGAATTTAGATTTAGCCAAGTTTCTAAAAACGATCTCTATCACGGGAGACGGCGGCCAAACACT
asic1a_Frag168-327_insertion228	TAGATACTAGCTCGTCTCCTGTTCTTCTTTGCGTTTGTACCGAAAGGGTTCAGTATTATTTCCATTATCATCATGTTACTAAAAGCGGGAGACCGGGGTCTCTGAGCCTCGACGAAGTCGCCGCATCACAGTTGACCTTCCCTGCTGTAACCCTTTGCAATCTGAACGAATTTAGATTTAGCCAAGTTTCTAAAAACGATCTCTATCACGGGAGACGGCGGCCAAACACT
asic1a_Frag168-327_insertion231	TAGATACTAGCTCGTCTCCTGTTCTTCTTTGCGTTTGTACCGAAAGGGTTCAGTATTATTTCCATTATCATCATGTTACTAAACTCAGCGGGAGACCGGGGTCTCTGAGCGACGAAGTCGCCGCATCACAGTTGACCTTCCCTGCTGTAACCCTTTGCAATCTGAACGAATTTAGATTTAGCCAAGTTTCTAAAAACGATCTCTATCACGGGAGACGGCGGCCAAACACT
asic1a_Frag168-327_insertion234	TAGATACTAGCTCGTCTCCTGTTCTTCTTTGCGTTTGTACCGAAAGGGTTCAGTATTATTTCCATTATCATCATGTTACTAAACTCGACAGCGGGAGACCGGGGTCTCTGAGCGAAGTCGCCGCATCACAGTTGACCTTCCCTGCTGTAACCCTTTGCAATCTGAACGAATTTAGATTTAGCCAAGTTTCTAAAAACGATCTCTATCACGGGAGACGGCGGCCAAACACT
asic1a_Frag168-327_insertion237	TAGATACTAGCTCGTCTCCTGTTCTTCTTTGCGTTTGTACCGAAAGGGTTCAGTATTATTTCCATTATCATCATGTTACTAAACTCGACGAAAGCGGGAGACCGGGGTCTCTGAGCGTCGCCGCATCACAGTTGACCTTCCCTGCTGTAACCCTTTGCAATCTGAACGAATTTAGATTTAGCCAAGTTTCTAAAAACGATCTCTATCACGGGAGACGGCGGCCAAACACT
asic1a_Frag168-327_insertion240	TAGATACTAGCTCGTCTCCTGTTCTTCTTTGCGTTTGTACCGAAAGGGTTCAGTATTATTTCCATTATCATCATGTTACTAAACTCGACGAAGTCAGCGGGAGACCGGGGTCTCTGAGCGCCGCATCACAGTTGACCTTCCCTGCTGTAACCCTTTGCAATCTGAACGAATTTAGATTTAGCCAAGTTTCTAAAAACGATCTCTATCACGGGAGACGGCGGCCAAACACT
asic1a_Frag168-327_insertion243	TAGATACTAGCTCGTCTCCTGTTCTTCTTTGCGTTTGTACCGAAAGGGTTCAGTATTATTTCCATTATCATCATGTTACTAAACTCGACGAAGTCGCCAGCGGGAGACCGGGGTCTCTGAGCGCATCACAGTTGACCTTCCCTGCTGTAACCCTTTGCAATCTGAACGAATTTAGATTTAGCCAAGTTTCTAAAAACGATCTCTATCACGGGAGACGGCGGCCAAACACT
asic1a_Frag168-327_insertion246	TAGATACTAGCTCGTCTCCTGTTCTTCTTTGCGTTTGTACCGAAAGGGTTCAGTATTATTTCCATTATCATCATGTTACTAAACTCGACGAAGTCGCCGCAAGCGGGAGACCGGGGTCTCTGAGCTCACAGTTGACCTTCCCTGCTGTAACCCTTTGCAATCTGAACGAATTTAGATTTAGCCAAGTTTCTAAAAACGATCTCTATCACGGGAGACGGCGGCCAAACACT
asic1a_Frag168-327_insertion249	TAGATACTAGCTCGTCTCCTGTTCTTCTTTGCGTTTGTACCGAAAGGGTTCAGTATTATTTCCATTATCATCATGTTACTAAACTCGACGAAGTCGCCGCATCAAGCGGGAGACCGGGGTCTCTGAGCCAGTTGACCTTCCCTGCTGTAACCCTTTGCAATCTGAACGAATTTAGATTTAGCCAAGTTTCTAAAAACGATCTCTATCACGGGAGACGGCGGCCAAACACT
asic1a_Frag168-327_insertion252	TAGATACTAGCTCGTCTCCTGTTCTTCTTTGCGTTTGTACCGAAAGGGTTCAGTATTATTTCCATTATCATCATGTTACTAAACTCGACGAAGTCGCCGCATCACAGAGCGGGAGACCGGGGTCTCTGAGCTTGACCTTCCCTGCTGTAACCCTTTGCAATCTGAACGAATTTAGATTTAGCCAAGTTTCTAAAAACGATCTCTATCACGGGAGACGGCGGCCAAACACT
asic1a_Frag168-327_insertion255	TAGATACTAGCTCGTCTCCTGTTCTTCTTTGCGTTTGTACCGAAAGGGTTCAGTATTATTTCCATTATCATCATGTTACTAAACTCGACGAAGTCGCCGCATCACAGTTGAGCGGGAGACCGGGGTCTCTGAGCACCTTCCCTGCTGTAACCCTTTGCAATCTGAACGAATTTAGATTTAGCCAAGTTTCTAAAAACGATCTCTATCACGGGAGACGGCGGCCAAACACT
asic1a_Frag168-327_insertion258	TAGATACTAGCTCGTCTCCTGTTCTTCTTTGCGTTTGTACCGAAAGGGTTCAGTATTATTTCCATTATCATCATGTTACTAAACTCGACGAAGTCGCCGCATCACAGTTGACCAGCGGGAGACCGGGGTCTCTGAGCTTCCCTGCTGTAACCCTTTGCAATCTGAACGAATTTAGATTTAGCCAAGTTTCTAAAAACGATCTCTATCACGGGAGACGGCGGCCAAACACT
asic1a_Frag168-327_insertion261	TAGATACTAGCTCGTCTCCTGTTCTTCTTTGCGTTTGTACCGAAAGGGTTCAGTATTATTTCCATTATCATCATGTTACTAAACTCGACGAAGTCGCCGCATCACAGTTGACCTTCAGCGGGAGACCGGGGTCTCTGAGCCCTGCTGTAACCCTTTGCAATCTGAACGAATTTAGATTTAGCCAAGTTTCTAAAAACGATCTCTATCACGGGAGACGGCGGCCAAACACT
asic1a_Frag168-327_insertion264	TAGATACTAGCTCGTCTCCTGTTCTTCTTTGCGTTTGTACCGAAAGGGTTCAGTATTATTTCCATTATCATCATGTTACTAAACTCGACGAAGTCGCCGCATCACAGTTGACCTTCCCTAGCGGGAGACCGGGGTCTCTGAGCGCTGTAACCCTTTGCAATCTGAACGAATTTAGATTTAGCCAAGTTTCTAAAAACGATCTCTATCACGGGAGACGGCGGCCAAACACT
asic1a_Frag168-327_insertion267	TAGATACTAGCTCGTCTCCTGTTCTTCTTTGCGTTTGTACCGAAAGGGTTCAGTATTATTTCCATTATCATCATGTTACTAAACTCGACGAAGTCGCCGCATCACAGTTGACCTTCCCTGCTAGCGGGAGACCGGGGTCTCTGAGCGTAACCCTTTGCAATCTGAACGAATTTAGATTTAGCCAAGTTTCTAAAAACGATCTCTATCACGGGAGACGGCGGCCAAACACT
asic1a_Frag168-327_insertion270	TAGATACTAGCTCGTCTCCTGTTCTTCTTTGCGTTTGTACCGAAAGGGTTCAGTATTATTTCCATTATCATCATGTTACTAAACTCGACGAAGTCGCCGCATCACAGTTGACCTTCCCTGCTGTAAGCGGGAGACCGGGGTCTCTGAGCACCCTTTGCAATCTGAACGAATTTAGATTTAGCCAAGTTTCTAAAAACGATCTCTATCACGGGAGACGGCGGCCAAACACT
asic1a_Frag168-327_insertion273	TAGATACTAGCTCGTCTCCTGTTCTTCTTTGCGTTTGTACCGAAAGGGTTCAGTATTATTTCCATTATCATCATGTTACTAAACTCGACGAAGTCGCCGCATCACAGTTGACCTTCCCTGCTGTAACCAGCGGGAGACCGGGGTCTCTGAGCCTTTGCAATCTGAACGAATTTAGATTTAGCCAAGTTTCTAAAAACGATCTCTATCACGGGAGACGGCGGCCAAACACT
asic1a_Frag168-327_insertion276	TAGATACTAGCTCGTCTCCTGTTCTTCTTTGCGTTTGTACCGAAAGGGTTCAGTATTATTTCCATTATCATCATGTTACTAAACTCGACGAAGTCGCCGCATCACAGTTGACCTTCCCTGCTGTAACCCTTAGCGGGAGACCGGGGTCTCTGAGCTGCAATCTGAACGAATTTAGATTTAGCCAAGTTTCTAAAAACGATCTCTATCACGGGAGACGGCGGCCAAACACT
asic1a_Frag168-327_insertion279	TAGATACTAGCTCGTCTCCTGTTCTTCTTTGCGTTTGTACCGAAAGGGTTCAGTATTATTTCCATTATCATCATGTTACTAAACTCGACGAAGTCGCCGCATCACAGTTGACCTTCCCTGCTGTAACCCTTTGCAGCGGGAGACCGGGGTCTCTGAGCAATCTGAACGAATTTAGATTTAGCCAAGTTTCTAAAAACGATCTCTATCACGGGAGACGGCGGCCAAACACT
asic1a_Frag168-327_insertion282	TAGATACTAGCTCGTCTCCTGTTCTTCTTTGCGTTTGTACCGAAAGGGTTCAGTATTATTTCCATTATCATCATGTTACTAAACTCGACGAAGTCGCCGCATCACAGTTGACCTTCCCTGCTGTAACCCTTTGCAATAGCGGGAGACCGGGGTCTCTGAGCCTGAACGAATTTAGATTTAGCCAAGTTTCTAAAAACGATCTCTATCACGGGAGACGGCGGCCAAACACT
asic1a_Frag168-327_insertion285	TAGATACTAGCTCGTCTCCTGTTCTTCTTTGCGTTTGTACCGAAAGGGTTCAGTATTATTTCCATTATCATCATGTTACTAAACTCGACGAAGTCGCCGCATCACAGTTGACCTTCCCTGCTGTAACCCTTTGCAATCTGAGCGGGAGACCGGGGTCTCTGAGCAACGAATTTAGATTTAGCCAAGTTTCTAAAAACGATCTCTATCACGGGAGACGGCGGCCAAACACT
asic1a_Frag168-327_insertion288	TAGATACTAGCTCGTCTCCTGTTCTTCTTTGCGTTTGTACCGAAAGGGTTCAGTATTATTTCCATTATCATCATGTTACTAAACTCGACGAAGTCGCCGCATCACAGTTGACCTTCCCTGCTGTAACCCTTTGCAATCTGAACAGCGGGAGACCGGGGTCTCTGAGCGAATTTAGATTTAGCCAAGTTTCTAAAAACGATCTCTATCACGGGAGACGGCGGCCAAACACT
asic1a_Frag168-327_insertion291	TAGATACTAGCTCGTCTCCTGTTCTTCTTTGCGTTTGTACCGAAAGGGTTCAGTATTATTTCCATTATCATCATGTTACTAAACTCGACGAAGTCGCCGCATCACAGTTGACCTTCCCTGCTGTAACCCTTTGCAATCTGAACGAAAGCGGGAGACCGGGGTCTCTGAGCTTTAGATTTAGCCAAGTTTCTAAAAACGATCTCTATCACGGGAGACGGCGGCCAAACACT
asic1a_Frag168-327_insertion294	TAGATACTAGCTCGTCTCCTGTTCTTCTTTGCGTTTGTACCGAAAGGGTTCAGTATTATTTCCATTATCATCATGTTACTAAACTCGACGAAGTCGCCGCATCACAGTTGACCTTCCCTGCTGTAACCCTTTGCAATCTGAACGAATTTAGCGGGAGACCGGGGTCTCTGAGCAGATTTAGCCAAGTTTCTAAAAACGATCTCTATCACGGGAGACGGCGGCCAAACACT
asic1a_Frag168-327_insertion297	TAGATACTAGCTCGTCTCCTGTTCTTCTTTGCGTTTGTACCGAAAGGGTTCAGTATTATTTCCATTATCATCATGTTACTAAACTCGACGAAGTCGCCGCATCACAGTTGACCTTCCCTGCTGTAACCCTTTGCAATCTGAACGAATTTAGAAGCGGGAGACCGGGGTCTCTGAGCTTTAGCCAAGTTTCTAAAAACGATCTCTATCACGGGAGACGGCGGCCAAACACT
asic1a_Frag168-327_insertion300	TAGATACTAGCTCGTCTCCTGTTCTTCTTTGCGTTTGTACCGAAAGGGTTCAGTATTATTTCCATTATCATCATGTTACTAAACTCGACGAAGTCGCCGCATCACAGTTGACCTTCCCTGCTGTAACCCTTTGCAATCTGAACGAATTTAGATTTAGCGGGAGACCGGGGTCTCTGAGCAGCCAAGTTTCTAAAAACGATCTCTATCACGGGAGACGGCGGCCAAACACT
asic1a_Frag168-327_insertion303	TAGATACTAGCTCGTCTCCTGTTCTTCTTTGCGTTTGTACCGAAAGGGTTCAGTATTATTTCCATTATCATCATGTTACTAAACTCGACGAAGTCGCCGCATCACAGTTGACCTTCCCTGCTGTAACCCTTTGCAATCTGAACGAATTTAGATTTAGCAGCGGGAGACCGGGGTCTCTGAGCCAAGTTTCTAAAAACGATCTCTATCACGGGAGACGGCGGCCAAACACT
asic1a_Frag168-327_insertion306	TAGATACTAGCTCGTCTCCTGTTCTTCTTTGCGTTTGTACCGAAAGGGTTCAGTATTATTTCCATTATCATCATGTTACTAAACTCGACGAAGTCGCCGCATCACAGTTGACCTTCCCTGCTGTAACCCTTTGCAATCTGAACGAATTTAGATTTAGCCAAAGCGGGAGACCGGGGTCTCTGAGCGTTTCTAAAAACGATCTCTATCACGGGAGACGGCGGCCAAACACT
asic1a_Frag168-327_insertion309	TAGATACTAGCTCGTCTCCTGTTCTTCTTTGCGTTTGTACCGAAAGGGTTCAGTATTATTTCCATTATCATCATGTTACTAAACTCGACGAAGTCGCCGCATCACAGTTGACCTTCCCTGCTGTAACCCTTTGCAATCTGAACGAATTTAGATTTAGCCAAGTTAGCGGGAGACCGGGGTCTCTGAGCTCTAAAAACGATCTCTATCACGGGAGACGGCGGCCAAACACT
asic1a_Frag168-327_insertion312	TAGATACTAGCTCGTCTCCTGTTCTTCTTTGCGTTTGTACCGAAAGGGTTCAGTATTATTTCCATTATCATCATGTTACTAAACTCGACGAAGTCGCCGCATCACAGTTGACCTTCCCTGCTGTAACCCTTTGCAATCTGAACGAATTTAGATTTAGCCAAGTTTCTAGCGGGAGACCGGGGTCTCTGAGCAAAAACGATCTCTATCACGGGAGACGGCGGCCAAACACT
asic1a_Frag168-327_insertion315	TAGATACTAGCTCGTCTCCTGTTCTTCTTTGCGTTTGTACCGAAAGGGTTCAGTATTATTTCCATTATCATCATGTTACTAAACTCGACGAAGTCGCCGCATCACAGTTGACCTTCCCTGCTGTAACCCTTTGCAATCTGAACGAATTTAGATTTAGCCAAGTTTCTAAAAGCGGGAGACCGGGGTCTCTGAGCAACGATCTCTATCACGGGAGACGGCGGCCAAACACT
asic1a_Frag168-327_insertion318	TAGATACTAGCTCGTCTCCTGTTCTTCTTTGCGTTTGTACCGAAAGGGTTCAGTATTATTTCCATTATCATCATGTTACTAAACTCGACGAAGTCGCCGCATCACAGTTGACCTTCCCTGCTGTAACCCTTTGCAATCTGAACGAATTTAGATTTAGCCAAGTTTCTAAAAACAGCGGGAGACCGGGGTCTCTGAGCGATCTCTATCACGGGAGACGGCGGCCAAACACT
asic1a_Frag168-327_insertion321	TAGATACTAGCTCGTCTCCTGTTCTTCTTTGCGTTTGTACCGAAAGGGTTCAGTATTATTTCCATTATCATCATGTTACTAAACTCGACGAAGTCGCCGCATCACAGTTGACCTTCCCTGCTGTAACCCTTTGCAATCTGAACGAATTTAGATTTAGCCAAGTTTCTAAAAACGATAGCGGGAGACCGGGGTCTCTGAGCCTCTATCACGGGAGACGGCGGCCAAACACT
asic1a_Frag168-327_insertion324	TAGATACTAGCTCGTCTCCTGTTCTTCTTTGCGTTTGTACCGAAAGGGTTCAGTATTATTTCCATTATCATCATGTTACTAAACTCGACGAAGTCGCCGCATCACAGTTGACCTTCCCTGCTGTAACCCTTTGCAATCTGAACGAATTTAGATTTAGCCAAGTTTCTAAAAACGATCTCAGCGGGAGACCGGGGTCTCTGAGCTATCACGGGAGACGGCGGCCAAACACT
asic1a_Frag168-327_insertion327	TAGATACTAGCTCGTCTCCTGTTCTTCTTTGCGTTTGTACCGAAAGGGTTCAGTATTATTTCCATTATCATCATGTTACTAAACTCGACGAAGTCGCCGCATCACAGTTGACCTTCCCTGCTGTAACCCTTTGCAATCTGAACGAATTTAGATTTAGCCAAGTTTCTAAAAACGATCTCTATAGCGGGAGACCGGGGTCTCTGAGCCACGGGAGACGGCGGCCAAACACT
asic1a_Frag330-495_insertion330	GCGTCTTTTCGTCTCCTCACAGCGGGAGACCGGGGTCTCTGAGCGCCGGTGAACTTCTCGCCCTCTTGAATAATCGCTATGAGATTCCCGATACACAAATGGCAGATGAAAAGCAACTGGAGATCCTCCAGGATAAGGCCAACTTTCGGTCCTTCGATTATAAAGATGATGATGATAAAAAGCCCAAGCCCTTCAATATGCGAGAGTTTTACGGGAGACGGTAATGGGTG
asic1a_Frag330-495_insertion333	GCGTCTTTTCGTCTCCTCACGCCAGCGGGAGACCGGGGTCTCTGAGCGGTGAACTTCTCGCCCTCTTGAATAATCGCTATGAGATTCCCGATACACAAATGGCAGATGAAAAGCAACTGGAGATCCTCCAGGATAAGGCCAACTTTCGGTCCTTCGATTATAAAGATGATGATGATAAAAAGCCCAAGCCCTTCAATATGCGAGAGTTTTACGGGAGACGGTAATGGGTG
asic1a_Frag330-495_insertion336	GCGTCTTTTCGTCTCCTCACGCCGGTAGCGGGAGACCGGGGTCTCTGAGCGAACTTCTCGCCCTCTTGAATAATCGCTATGAGATTCCCGATACACAAATGGCAGATGAAAAGCAACTGGAGATCCTCCAGGATAAGGCCAACTTTCGGTCCTTCGATTATAAAGATGATGATGATAAAAAGCCCAAGCCCTTCAATATGCGAGAGTTTTACGGGAGACGGTAATGGGTG
asic1a_Frag330-495_insertion339	GCGTCTTTTCGTCTCCTCACGCCGGTGAAAGCGGGAGACCGGGGTCTCTGAGCCTTCTCGCCCTCTTGAATAATCGCTATGAGATTCCCGATACACAAATGGCAGATGAAAAGCAACTGGAGATCCTCCAGGATAAGGCCAACTTTCGGTCCTTCGATTATAAAGATGATGATGATAAAAAGCCCAAGCCCTTCAATATGCGAGAGTTTTACGGGAGACGGTAATGGGTG
asic1a_Frag330-495_insertion342	GCGTCTTTTCGTCTCCTCACGCCGGTGAACTTAGCGGGAGACCGGGGTCTCTGAGCCTCGCCCTCTTGAATAATCGCTATGAGATTCCCGATACACAAATGGCAGATGAAAAGCAACTGGAGATCCTCCAGGATAAGGCCAACTTTCGGTCCTTCGATTATAAAGATGATGATGATAAAAAGCCCAAGCCCTTCAATATGCGAGAGTTTTACGGGAGACGGTAATGGGTG
asic1a_Frag330-495_insertion345	GCGTCTTTTCGTCTCCTCACGCCGGTGAACTTCTCAGCGGGAGACCGGGGTCTCTGAGCGCCCTCTTGAATAATCGCTATGAGATTCCCGATACACAAATGGCAGATGAAAAGCAACTGGAGATCCTCCAGGATAAGGCCAACTTTCGGTCCTTCGATTATAAAGATGATGATGATAAAAAGCCCAAGCCCTTCAATATGCGAGAGTTTTACGGGAGACGGTAATGGGTG
asic1a_Frag330-495_insertion348	GCGTCTTTTCGTCTCCTCACGCCGGTGAACTTCTCGCCAGCGGGAGACCGGGGTCTCTGAGCCTCTTGAATAATCGCTATGAGATTCCCGATACACAAATGGCAGATGAAAAGCAACTGGAGATCCTCCAGGATAAGGCCAACTTTCGGTCCTTCGATTATAAAGATGATGATGATAAAAAGCCCAAGCCCTTCAATATGCGAGAGTTTTACGGGAGACGGTAATGGGTG
asic1a_Frag330-495_insertion351	GCGTCTTTTCGTCTCCTCACGCCGGTGAACTTCTCGCCCTCAGCGGGAGACCGGGGTCTCTGAGCTTGAATAATCGCTATGAGATTCCCGATACACAAATGGCAGATGAAAAGCAACTGGAGATCCTCCAGGATAAGGCCAACTTTCGGTCCTTCGATTATAAAGATGATGATGATAAAAAGCCCAAGCCCTTCAATATGCGAGAGTTTTACGGGAGACGGTAATGGGTG
asic1a_Frag330-495_insertion354	GCGTCTTTTCGTCTCCTCACGCCGGTGAACTTCTCGCCCTCTTGAGCGGGAGACCGGGGTCTCTGAGCAATAATCGCTATGAGATTCCCGATACACAAATGGCAGATGAAAAGCAACTGGAGATCCTCCAGGATAAGGCCAACTTTCGGTCCTTCGATTATAAAGATGATGATGATAAAAAGCCCAAGCCCTTCAATATGCGAGAGTTTTACGGGAGACGGTAATGGGTG
asic1a_Frag330-495_insertion357	GCGTCTTTTCGTCTCCTCACGCCGGTGAACTTCTCGCCCTCTTGAATAGCGGGAGACCGGGGTCTCTGAGCAATCGCTATGAGATTCCCGATACACAAATGGCAGATGAAAAGCAACTGGAGATCCTCCAGGATAAGGCCAACTTTCGGTCCTTCGATTATAAAGATGATGATGATAAAAAGCCCAAGCCCTTCAATATGCGAGAGTTTTACGGGAGACGGTAATGGGTG
asic1a_Frag330-495_insertion360	GCGTCTTTTCGTCTCCTCACGCCGGTGAACTTCTCGCCCTCTTGAATAATAGCGGGAGACCGGGGTCTCTGAGCCGCTATGAGATTCCCGATACACAAATGGCAGATGAAAAGCAACTGGAGATCCTCCAGGATAAGGCCAACTTTCGGTCCTTCGATTATAAAGATGATGATGATAAAAAGCCCAAGCCCTTCAATATGCGAGAGTTTTACGGGAGACGGTAATGGGTG
asic1a_Frag330-495_insertion363	GCGTCTTTTCGTCTCCTCACGCCGGTGAACTTCTCGCCCTCTTGAATAATCGCAGCGGGAGACCGGGGTCTCTGAGCTATGAGATTCCCGATACACAAATGGCAGATGAAAAGCAACTGGAGATCCTCCAGGATAAGGCCAACTTTCGGTCCTTCGATTATAAAGATGATGATGATAAAAAGCCCAAGCCCTTCAATATGCGAGAGTTTTACGGGAGACGGTAATGGGTG
asic1a_Frag330-495_insertion366	GCGTCTTTTCGTCTCCTCACGCCGGTGAACTTCTCGCCCTCTTGAATAATCGCTATAGCGGGAGACCGGGGTCTCTGAGCGAGATTCCCGATACACAAATGGCAGATGAAAAGCAACTGGAGATCCTCCAGGATAAGGCCAACTTTCGGTCCTTCGATTATAAAGATGATGATGATAAAAAGCCCAAGCCCTTCAATATGCGAGAGTTTTACGGGAGACGGTAATGGGTG
asic1a_Frag330-495_insertion369	GCGTCTTTTCGTCTCCTCACGCCGGTGAACTTCTCGCCCTCTTGAATAATCGCTATGAGAGCGGGAGACCGGGGTCTCTGAGCATTCCCGATACACAAATGGCAGATGAAAAGCAACTGGAGATCCTCCAGGATAAGGCCAACTTTCGGTCCTTCGATTATAAAGATGATGATGATAAAAAGCCCAAGCCCTTCAATATGCGAGAGTTTTACGGGAGACGGTAATGGGTG
asic1a_Frag330-495_insertion372	GCGTCTTTTCGTCTCCTCACGCCGGTGAACTTCTCGCCCTCTTGAATAATCGCTATGAGATTAGCGGGAGACCGGGGTCTCTGAGCCCCGATACACAAATGGCAGATGAAAAGCAACTGGAGATCCTCCAGGATAAGGCCAACTTTCGGTCCTTCGATTATAAAGATGATGATGATAAAAAGCCCAAGCCCTTCAATATGCGAGAGTTTTACGGGAGACGGTAATGGGTG
asic1a_Frag330-495_insertion375	GCGTCTTTTCGTCTCCTCACGCCGGTGAACTTCTCGCCCTCTTGAATAATCGCTATGAGATTCCCAGCGGGAGACCGGGGTCTCTGAGCGATACACAAATGGCAGATGAAAAGCAACTGGAGATCCTCCAGGATAAGGCCAACTTTCGGTCCTTCGATTATAAAGATGATGATGATAAAAAGCCCAAGCCCTTCAATATGCGAGAGTTTTACGGGAGACGGTAATGGGTG
asic1a_Frag330-495_insertion378	GCGTCTTTTCGTCTCCTCACGCCGGTGAACTTCTCGCCCTCTTGAATAATCGCTATGAGATTCCCGATAGCGGGAGACCGGGGTCTCTGAGCACACAAATGGCAGATGAAAAGCAACTGGAGATCCTCCAGGATAAGGCCAACTTTCGGTCCTTCGATTATAAAGATGATGATGATAAAAAGCCCAAGCCCTTCAATATGCGAGAGTTTTACGGGAGACGGTAATGGGTG
asic1a_Frag330-495_insertion381	GCGTCTTTTCGTCTCCTCACGCCGGTGAACTTCTCGCCCTCTTGAATAATCGCTATGAGATTCCCGATACAAGCGGGAGACCGGGGTCTCTGAGCCAAATGGCAGATGAAAAGCAACTGGAGATCCTCCAGGATAAGGCCAACTTTCGGTCCTTCGATTATAAAGATGATGATGATAAAAAGCCCAAGCCCTTCAATATGCGAGAGTTTTACGGGAGACGGTAATGGGTG
asic1a_Frag330-495_insertion384	GCGTCTTTTCGTCTCCTCACGCCGGTGAACTTCTCGCCCTCTTGAATAATCGCTATGAGATTCCCGATACACAAAGCGGGAGACCGGGGTCTCTGAGCATGGCAGATGAAAAGCAACTGGAGATCCTCCAGGATAAGGCCAACTTTCGGTCCTTCGATTATAAAGATGATGATGATAAAAAGCCCAAGCCCTTCAATATGCGAGAGTTTTACGGGAGACGGTAATGGGTG
asic1a_Frag330-495_insertion387	GCGTCTTTTCGTCTCCTCACGCCGGTGAACTTCTCGCCCTCTTGAATAATCGCTATGAGATTCCCGATACACAAATGAGCGGGAGACCGGGGTCTCTGAGCGCAGATGAAAAGCAACTGGAGATCCTCCAGGATAAGGCCAACTTTCGGTCCTTCGATTATAAAGATGATGATGATAAAAAGCCCAAGCCCTTCAATATGCGAGAGTTTTACGGGAGACGGTAATGGGTG
asic1a_Frag330-495_insertion390	GCGTCTTTTCGTCTCCTCACGCCGGTGAACTTCTCGCCCTCTTGAATAATCGCTATGAGATTCCCGATACACAAATGGCAAGCGGGAGACCGGGGTCTCTGAGCGATGAAAAGCAACTGGAGATCCTCCAGGATAAGGCCAACTTTCGGTCCTTCGATTATAAAGATGATGATGATAAAAAGCCCAAGCCCTTCAATATGCGAGAGTTTTACGGGAGACGGTAATGGGTG
asic1a_Frag330-495_insertion393	GCGTCTTTTCGTCTCCTCACGCCGGTGAACTTCTCGCCCTCTTGAATAATCGCTATGAGATTCCCGATACACAAATGGCAGATAGCGGGAGACCGGGGTCTCTGAGCGAAAAGCAACTGGAGATCCTCCAGGATAAGGCCAACTTTCGGTCCTTCGATTATAAAGATGATGATGATAAAAAGCCCAAGCCCTTCAATATGCGAGAGTTTTACGGGAGACGGTAATGGGTG
asic1a_Frag330-495_insertion396	GCGTCTTTTCGTCTCCTCACGCCGGTGAACTTCTCGCCCTCTTGAATAATCGCTATGAGATTCCCGATACACAAATGGCAGATGAAAGCGGGAGACCGGGGTCTCTGAGCAAGCAACTGGAGATCCTCCAGGATAAGGCCAACTTTCGGTCCTTCGATTATAAAGATGATGATGATAAAAAGCCCAAGCCCTTCAATATGCGAGAGTTTTACGGGAGACGGTAATGGGTG
asic1a_Frag330-495_insertion399	GCGTCTTTTCGTCTCCTCACGCCGGTGAACTTCTCGCCCTCTTGAATAATCGCTATGAGATTCCCGATACACAAATGGCAGATGAAAAGAGCGGGAGACCGGGGTCTCTGAGCCAACTGGAGATCCTCCAGGATAAGGCCAACTTTCGGTCCTTCGATTATAAAGATGATGATGATAAAAAGCCCAAGCCCTTCAATATGCGAGAGTTTTACGGGAGACGGTAATGGGTG
asic1a_Frag330-495_insertion402	GCGTCTTTTCGTCTCCTCACGCCGGTGAACTTCTCGCCCTCTTGAATAATCGCTATGAGATTCCCGATACACAAATGGCAGATGAAAAGCAAAGCGGGAGACCGGGGTCTCTGAGCCTGGAGATCCTCCAGGATAAGGCCAACTTTCGGTCCTTCGATTATAAAGATGATGATGATAAAAAGCCCAAGCCCTTCAATATGCGAGAGTTTTACGGGAGACGGTAATGGGTG
asic1a_Frag330-495_insertion405	GCGTCTTTTCGTCTCCTCACGCCGGTGAACTTCTCGCCCTCTTGAATAATCGCTATGAGATTCCCGATACACAAATGGCAGATGAAAAGCAACTGAGCGGGAGACCGGGGTCTCTGAGCGAGATCCTCCAGGATAAGGCCAACTTTCGGTCCTTCGATTATAAAGATGATGATGATAAAAAGCCCAAGCCCTTCAATATGCGAGAGTTTTACGGGAGACGGTAATGGGTG
asic1a_Frag330-495_insertion408	GCGTCTTTTCGTCTCCTCACGCCGGTGAACTTCTCGCCCTCTTGAATAATCGCTATGAGATTCCCGATACACAAATGGCAGATGAAAAGCAACTGGAGAGCGGGAGACCGGGGTCTCTGAGCATCCTCCAGGATAAGGCCAACTTTCGGTCCTTCGATTATAAAGATGATGATGATAAAAAGCCCAAGCCCTTCAATATGCGAGAGTTTTACGGGAGACGGTAATGGGTG
asic1a_Frag330-495_insertion411	GCGTCTTTTCGTCTCCTCACGCCGGTGAACTTCTCGCCCTCTTGAATAATCGCTATGAGATTCCCGATACACAAATGGCAGATGAAAAGCAACTGGAGATCAGCGGGAGACCGGGGTCTCTGAGCCTCCAGGATAAGGCCAACTTTCGGTCCTTCGATTATAAAGATGATGATGATAAAAAGCCCAAGCCCTTCAATATGCGAGAGTTTTACGGGAGACGGTAATGGGTG
asic1a_Frag330-495_insertion414	GCGTCTTTTCGTCTCCTCACGCCGGTGAACTTCTCGCCCTCTTGAATAATCGCTATGAGATTCCCGATACACAAATGGCAGATGAAAAGCAACTGGAGATCCTCAGCGGGAGACCGGGGTCTCTGAGCCAGGATAAGGCCAACTTTCGGTCCTTCGATTATAAAGATGATGATGATAAAAAGCCCAAGCCCTTCAATATGCGAGAGTTTTACGGGAGACGGTAATGGGTG
asic1a_Frag330-495_insertion417	GCGTCTTTTCGTCTCCTCACGCCGGTGAACTTCTCGCCCTCTTGAATAATCGCTATGAGATTCCCGATACACAAATGGCAGATGAAAAGCAACTGGAGATCCTCCAGAGCGGGAGACCGGGGTCTCTGAGCGATAAGGCCAACTTTCGGTCCTTCGATTATAAAGATGATGATGATAAAAAGCCCAAGCCCTTCAATATGCGAGAGTTTTACGGGAGACGGTAATGGGTG
asic1a_Frag330-495_insertion420	GCGTCTTTTCGTCTCCTCACGCCGGTGAACTTCTCGCCCTCTTGAATAATCGCTATGAGATTCCCGATACACAAATGGCAGATGAAAAGCAACTGGAGATCCTCCAGGATAGCGGGAGACCGGGGTCTCTGAGCAAGGCCAACTTTCGGTCCTTCGATTATAAAGATGATGATGATAAAAAGCCCAAGCCCTTCAATATGCGAGAGTTTTACGGGAGACGGTAATGGGTG
asic1a_Frag330-495_insertion423	GCGTCTTTTCGTCTCCTCACGCCGGTGAACTTCTCGCCCTCTTGAATAATCGCTATGAGATTCCCGATACACAAATGGCAGATGAAAAGCAACTGGAGATCCTCCAGGATAAGAGCGGGAGACCGGGGTCTCTGAGCGCCAACTTTCGGTCCTTCGATTATAAAGATGATGATGATAAAAAGCCCAAGCCCTTCAATATGCGAGAGTTTTACGGGAGACGGTAATGGGTG
asic1a_Frag330-495_insertion426	GCGTCTTTTCGTCTCCTCACGCCGGTGAACTTCTCGCCCTCTTGAATAATCGCTATGAGATTCCCGATACACAAATGGCAGATGAAAAGCAACTGGAGATCCTCCAGGATAAGGCCAGCGGGAGACCGGGGTCTCTGAGCAACTTTCGGTCCTTCGATTATAAAGATGATGATGATAAAAAGCCCAAGCCCTTCAATATGCGAGAGTTTTACGGGAGACGGTAATGGGTG
asic1a_Frag330-495_insertion429	GCGTCTTTTCGTCTCCTCACGCCGGTGAACTTCTCGCCCTCTTGAATAATCGCTATGAGATTCCCGATACACAAATGGCAGATGAAAAGCAACTGGAGATCCTCCAGGATAAGGCCAACAGCGGGAGACCGGGGTCTCTGAGCTTTCGGTCCTTCGATTATAAAGATGATGATGATAAAAAGCCCAAGCCCTTCAATATGCGAGAGTTTTACGGGAGACGGTAATGGGTG
asic1a_Frag330-495_insertion432	GCGTCTTTTCGTCTCCTCACGCCGGTGAACTTCTCGCCCTCTTGAATAATCGCTATGAGATTCCCGATACACAAATGGCAGATGAAAAGCAACTGGAGATCCTCCAGGATAAGGCCAACTTTAGCGGGAGACCGGGGTCTCTGAGCCGGTCCTTCGATTATAAAGATGATGATGATAAAAAGCCCAAGCCCTTCAATATGCGAGAGTTTTACGGGAGACGGTAATGGGTG
asic1a_Frag330-495_insertion435	GCGTCTTTTCGTCTCCTCACGCCGGTGAACTTCTCGCCCTCTTGAATAATCGCTATGAGATTCCCGATACACAAATGGCAGATGAAAAGCAACTGGAGATCCTCCAGGATAAGGCCAACTTTCGGAGCGGGAGACCGGGGTCTCTGAGCTCCTTCGATTATAAAGATGATGATGATAAAAAGCCCAAGCCCTTCAATATGCGAGAGTTTTACGGGAGACGGTAATGGGTG
asic1a_Frag330-495_insertion438	GCGTCTTTTCGTCTCCTCACGCCGGTGAACTTCTCGCCCTCTTGAATAATCGCTATGAGATTCCCGATACACAAATGGCAGATGAAAAGCAACTGGAGATCCTCCAGGATAAGGCCAACTTTCGGTCCAGCGGGAGACCGGGGTCTCTGAGCTTCGATTATAAAGATGATGATGATAAAAAGCCCAAGCCCTTCAATATGCGAGAGTTTTACGGGAGACGGTAATGGGTG
asic1a_Frag330-495_insertion441	GCGTCTTTTCGTCTCCTCACGCCGGTGAACTTCTCGCCCTCTTGAATAATCGCTATGAGATTCCCGATACACAAATGGCAGATGAAAAGCAACTGGAGATCCTCCAGGATAAGGCCAACTTTCGGTCCTTCAGCGGGAGACCGGGGTCTCTGAGCGATTATAAAGATGATGATGATAAAAAGCCCAAGCCCTTCAATATGCGAGAGTTTTACGGGAGACGGTAATGGGTG
asic1a_Frag330-495_insertion444	GCGTCTTTTCGTCTCCTCACGCCGGTGAACTTCTCGCCCTCTTGAATAATCGCTATGAGATTCCCGATACACAAATGGCAGATGAAAAGCAACTGGAGATCCTCCAGGATAAGGCCAACTTTCGGTCCTTCGATAGCGGGAGACCGGGGTCTCTGAGCTATAAAGATGATGATGATAAAAAGCCCAAGCCCTTCAATATGCGAGAGTTTTACGGGAGACGGTAATGGGTG
asic1a_Frag330-495_insertion447	GCGTCTTTTCGTCTCCTCACGCCGGTGAACTTCTCGCCCTCTTGAATAATCGCTATGAGATTCCCGATACACAAATGGCAGATGAAAAGCAACTGGAGATCCTCCAGGATAAGGCCAACTTTCGGTCCTTCGATTATAGCGGGAGACCGGGGTCTCTGAGCAAAGATGATGATGATAAAAAGCCCAAGCCCTTCAATATGCGAGAGTTTTACGGGAGACGGTAATGGGTG
asic1a_Frag330-495_insertion450	GCGTCTTTTCGTCTCCTCACGCCGGTGAACTTCTCGCCCTCTTGAATAATCGCTATGAGATTCCCGATACACAAATGGCAGATGAAAAGCAACTGGAGATCCTCCAGGATAAGGCCAACTTTCGGTCCTTCGATTATAAAAGCGGGAGACCGGGGTCTCTGAGCGATGATGATGATAAAAAGCCCAAGCCCTTCAATATGCGAGAGTTTTACGGGAGACGGTAATGGGTG
asic1a_Frag330-495_insertion453	GCGTCTTTTCGTCTCCTCACGCCGGTGAACTTCTCGCCCTCTTGAATAATCGCTATGAGATTCCCGATACACAAATGGCAGATGAAAAGCAACTGGAGATCCTCCAGGATAAGGCCAACTTTCGGTCCTTCGATTATAAAGATAGCGGGAGACCGGGGTCTCTGAGCGATGATGATAAAAAGCCCAAGCCCTTCAATATGCGAGAGTTTTACGGGAGACGGTAATGGGTG
asic1a_Frag330-495_insertion456	GCGTCTTTTCGTCTCCTCACGCCGGTGAACTTCTCGCCCTCTTGAATAATCGCTATGAGATTCCCGATACACAAATGGCAGATGAAAAGCAACTGGAGATCCTCCAGGATAAGGCCAACTTTCGGTCCTTCGATTATAAAGATGATAGCGGGAGACCGGGGTCTCTGAGCGATGATAAAAAGCCCAAGCCCTTCAATATGCGAGAGTTTTACGGGAGACGGTAATGGGTG
asic1a_Frag330-495_insertion459	GCGTCTTTTCGTCTCCTCACGCCGGTGAACTTCTCGCCCTCTTGAATAATCGCTATGAGATTCCCGATACACAAATGGCAGATGAAAAGCAACTGGAGATCCTCCAGGATAAGGCCAACTTTCGGTCCTTCGATTATAAAGATGATGATAGCGGGAGACCGGGGTCTCTGAGCGATAAAAAGCCCAAGCCCTTCAATATGCGAGAGTTTTACGGGAGACGGTAATGGGTG
asic1a_Frag330-495_insertion462	GCGTCTTTTCGTCTCCTCACGCCGGTGAACTTCTCGCCCTCTTGAATAATCGCTATGAGATTCCCGATACACAAATGGCAGATGAAAAGCAACTGGAGATCCTCCAGGATAAGGCCAACTTTCGGTCCTTCGATTATAAAGATGATGATGATAGCGGGAGACCGGGGTCTCTGAGCAAAAAGCCCAAGCCCTTCAATATGCGAGAGTTTTACGGGAGACGGTAATGGGTG
asic1a_Frag330-495_insertion465	GCGTCTTTTCGTCTCCTCACGCCGGTGAACTTCTCGCCCTCTTGAATAATCGCTATGAGATTCCCGATACACAAATGGCAGATGAAAAGCAACTGGAGATCCTCCAGGATAAGGCCAACTTTCGGTCCTTCGATTATAAAGATGATGATGATAAAAGCGGGAGACCGGGGTCTCTGAGCAAGCCCAAGCCCTTCAATATGCGAGAGTTTTACGGGAGACGGTAATGGGTG
asic1a_Frag330-495_insertion468	GCGTCTTTTCGTCTCCTCACGCCGGTGAACTTCTCGCCCTCTTGAATAATCGCTATGAGATTCCCGATACACAAATGGCAGATGAAAAGCAACTGGAGATCCTCCAGGATAAGGCCAACTTTCGGTCCTTCGATTATAAAGATGATGATGATAAAAAGAGCGGGAGACCGGGGTCTCTGAGCCCCAAGCCCTTCAATATGCGAGAGTTTTACGGGAGACGGTAATGGGTG
asic1a_Frag330-495_insertion471	GCGTCTTTTCGTCTCCTCACGCCGGTGAACTTCTCGCCCTCTTGAATAATCGCTATGAGATTCCCGATACACAAATGGCAGATGAAAAGCAACTGGAGATCCTCCAGGATAAGGCCAACTTTCGGTCCTTCGATTATAAAGATGATGATGATAAAAAGCCCAGCGGGAGACCGGGGTCTCTGAGCAAGCCCTTCAATATGCGAGAGTTTTACGGGAGACGGTAATGGGTG
asic1a_Frag330-495_insertion474	GCGTCTTTTCGTCTCCTCACGCCGGTGAACTTCTCGCCCTCTTGAATAATCGCTATGAGATTCCCGATACACAAATGGCAGATGAAAAGCAACTGGAGATCCTCCAGGATAAGGCCAACTTTCGGTCCTTCGATTATAAAGATGATGATGATAAAAAGCCCAAGAGCGGGAGACCGGGGTCTCTGAGCCCCTTCAATATGCGAGAGTTTTACGGGAGACGGTAATGGGTG
asic1a_Frag330-495_insertion477	GCGTCTTTTCGTCTCCTCACGCCGGTGAACTTCTCGCCCTCTTGAATAATCGCTATGAGATTCCCGATACACAAATGGCAGATGAAAAGCAACTGGAGATCCTCCAGGATAAGGCCAACTTTCGGTCCTTCGATTATAAAGATGATGATGATAAAAAGCCCAAGCCCAGCGGGAGACCGGGGTCTCTGAGCTTCAATATGCGAGAGTTTTACGGGAGACGGTAATGGGTG
asic1a_Frag330-495_insertion480	GCGTCTTTTCGTCTCCTCACGCCGGTGAACTTCTCGCCCTCTTGAATAATCGCTATGAGATTCCCGATACACAAATGGCAGATGAAAAGCAACTGGAGATCCTCCAGGATAAGGCCAACTTTCGGTCCTTCGATTATAAAGATGATGATGATAAAAAGCCCAAGCCCTTCAGCGGGAGACCGGGGTCTCTGAGCAATATGCGAGAGTTTTACGGGAGACGGTAATGGGTG
asic1a_Frag330-495_insertion483	GCGTCTTTTCGTCTCCTCACGCCGGTGAACTTCTCGCCCTCTTGAATAATCGCTATGAGATTCCCGATACACAAATGGCAGATGAAAAGCAACTGGAGATCCTCCAGGATAAGGCCAACTTTCGGTCCTTCGATTATAAAGATGATGATGATAAAAAGCCCAAGCCCTTCAATAGCGGGAGACCGGGGTCTCTGAGCATGCGAGAGTTTTACGGGAGACGGTAATGGGTG
asic1a_Frag330-495_insertion486	GCGTCTTTTCGTCTCCTCACGCCGGTGAACTTCTCGCCCTCTTGAATAATCGCTATGAGATTCCCGATACACAAATGGCAGATGAAAAGCAACTGGAGATCCTCCAGGATAAGGCCAACTTTCGGTCCTTCGATTATAAAGATGATGATGATAAAAAGCCCAAGCCCTTCAATATGAGCGGGAGACCGGGGTCTCTGAGCCGAGAGTTTTACGGGAGACGGTAATGGGTG
asic1a_Frag330-495_insertion489	GCGTCTTTTCGTCTCCTCACGCCGGTGAACTTCTCGCCCTCTTGAATAATCGCTATGAGATTCCCGATACACAAATGGCAGATGAAAAGCAACTGGAGATCCTCCAGGATAAGGCCAACTTTCGGTCCTTCGATTATAAAGATGATGATGATAAAAAGCCCAAGCCCTTCAATATGCGAAGCGGGAGACCGGGGTCTCTGAGCGAGTTTTACGGGAGACGGTAATGGGTG
asic1a_Frag330-495_insertion492	GCGTCTTTTCGTCTCCTCACGCCGGTGAACTTCTCGCCCTCTTGAATAATCGCTATGAGATTCCCGATACACAAATGGCAGATGAAAAGCAACTGGAGATCCTCCAGGATAAGGCCAACTTTCGGTCCTTCGATTATAAAGATGATGATGATAAAAAGCCCAAGCCCTTCAATATGCGAGAGAGCGGGAGACCGGGGTCTCTGAGCTTTTACGGGAGACGGTAATGGGTG
asic1a_Frag330-495_insertion495	GCGTCTTTTCGTCTCCTCACGCCGGTGAACTTCTCGCCCTCTTGAATAATCGCTATGAGATTCCCGATACACAAATGGCAGATGAAAAGCAACTGGAGATCCTCCAGGATAAGGCCAACTTTCGGTCCTTCGATTATAAAGATGATGATGATAAAAAGCCCAAGCCCTTCAATATGCGAGAGTTTAGCGGGAGACCGGGGTCTCTGAGCTACGGGAGACGGTAATGGGTG
asic1a_Frag498-660_insertion498	CACTTGAGGTCCGTCTCCTTACAGCGGGAGACCGGGGTCTCTGAGCGATCGCGCTGGCCATGATATTCGGGATATGCTTCTCTCATGCCACTTCAGGGGGGAGGTTTGTTCCGCAGAGGACTTCAAGGTCGTGTTTACCCGCTACGGCAAGTGTTATACCTTCAACAGCGGTCGCGACGGGCGCCCTCGGCTTAAAACCATGAAAGGCGGCAGGAGACGAGCACCATAGG

asic1a_Frag498-660_insertion501	CACTTGAGGTCCGTCTCCTTACGATAGCGGGAGACCGGGGTCTCTGAGCCGCGCTGGCCATGATATTCGGGATATGCTTCTCTCATGCCACTTCAGGGGGGAGGTTTGTTCCGCAGAGGACTTCAAGGTCGTGTTTACCCGCTACGGCAAGTGTTATACCTTCAACAGCGGTCGCGACGGGCGCCCTCGGCTTAAAACCATGAAAGGCGGCAGGAGACGAGCACCATAGG
asic1a_Frag498-660_insertion504	CACTTGAGGTCCGTCTCCTTACGATCGCAGCGGGAGACCGGGGTCTCTGAGCGCTGGCCATGATATTCGGGATATGCTTCTCTCATGCCACTTCAGGGGGGAGGTTTGTTCCGCAGAGGACTTCAAGGTCGTGTTTACCCGCTACGGCAAGTGTTATACCTTCAACAGCGGTCGCGACGGGCGCCCTCGGCTTAAAACCATGAAAGGCGGCAGGAGACGAGCACCATAGG
asic1a_Frag498-660_insertion507	CACTTGAGGTCCGTCTCCTTACGATCGCGCTAGCGGGAGACCGGGGTCTCTGAGCGGCCATGATATTCGGGATATGCTTCTCTCATGCCACTTCAGGGGGGAGGTTTGTTCCGCAGAGGACTTCAAGGTCGTGTTTACCCGCTACGGCAAGTGTTATACCTTCAACAGCGGTCGCGACGGGCGCCCTCGGCTTAAAACCATGAAAGGCGGCAGGAGACGAGCACCATAGG
asic1a_Frag498-660_insertion510	CACTTGAGGTCCGTCTCCTTACGATCGCGCTGGCAGCGGGAGACCGGGGTCTCTGAGCCATGATATTCGGGATATGCTTCTCTCATGCCACTTCAGGGGGGAGGTTTGTTCCGCAGAGGACTTCAAGGTCGTGTTTACCCGCTACGGCAAGTGTTATACCTTCAACAGCGGTCGCGACGGGCGCCCTCGGCTTAAAACCATGAAAGGCGGCAGGAGACGAGCACCATAGG
asic1a_Frag498-660_insertion513	CACTTGAGGTCCGTCTCCTTACGATCGCGCTGGCCATAGCGGGAGACCGGGGTCTCTGAGCGATATTCGGGATATGCTTCTCTCATGCCACTTCAGGGGGGAGGTTTGTTCCGCAGAGGACTTCAAGGTCGTGTTTACCCGCTACGGCAAGTGTTATACCTTCAACAGCGGTCGCGACGGGCGCCCTCGGCTTAAAACCATGAAAGGCGGCAGGAGACGAGCACCATAGG
asic1a_Frag498-660_insertion516	CACTTGAGGTCCGTCTCCTTACGATCGCGCTGGCCATGATAGCGGGAGACCGGGGTCTCTGAGCATTCGGGATATGCTTCTCTCATGCCACTTCAGGGGGGAGGTTTGTTCCGCAGAGGACTTCAAGGTCGTGTTTACCCGCTACGGCAAGTGTTATACCTTCAACAGCGGTCGCGACGGGCGCCCTCGGCTTAAAACCATGAAAGGCGGCAGGAGACGAGCACCATAGG
asic1a_Frag498-660_insertion519	CACTTGAGGTCCGTCTCCTTACGATCGCGCTGGCCATGATATTAGCGGGAGACCGGGGTCTCTGAGCCGGGATATGCTTCTCTCATGCCACTTCAGGGGGGAGGTTTGTTCCGCAGAGGACTTCAAGGTCGTGTTTACCCGCTACGGCAAGTGTTATACCTTCAACAGCGGTCGCGACGGGCGCCCTCGGCTTAAAACCATGAAAGGCGGCAGGAGACGAGCACCATAGG
asic1a_Frag498-660_insertion522	CACTTGAGGTCCGTCTCCTTACGATCGCGCTGGCCATGATATTCGGAGCGGGAGACCGGGGTCTCTGAGCGATATGCTTCTCTCATGCCACTTCAGGGGGGAGGTTTGTTCCGCAGAGGACTTCAAGGTCGTGTTTACCCGCTACGGCAAGTGTTATACCTTCAACAGCGGTCGCGACGGGCGCCCTCGGCTTAAAACCATGAAAGGCGGCAGGAGACGAGCACCATAGG
asic1a_Frag498-660_insertion525	CACTTGAGGTCCGTCTCCTTACGATCGCGCTGGCCATGATATTCGGGATAGCGGGAGACCGGGGTCTCTGAGCATGCTTCTCTCATGCCACTTCAGGGGGGAGGTTTGTTCCGCAGAGGACTTCAAGGTCGTGTTTACCCGCTACGGCAAGTGTTATACCTTCAACAGCGGTCGCGACGGGCGCCCTCGGCTTAAAACCATGAAAGGCGGCAGGAGACGAGCACCATAGG
asic1a_Frag498-660_insertion528	CACTTGAGGTCCGTCTCCTTACGATCGCGCTGGCCATGATATTCGGGATATGAGCGGGAGACCGGGGTCTCTGAGCCTTCTCTCATGCCACTTCAGGGGGGAGGTTTGTTCCGCAGAGGACTTCAAGGTCGTGTTTACCCGCTACGGCAAGTGTTATACCTTCAACAGCGGTCGCGACGGGCGCCCTCGGCTTAAAACCATGAAAGGCGGCAGGAGACGAGCACCATAGG
asic1a_Frag498-660_insertion531	CACTTGAGGTCCGTCTCCTTACGATCGCGCTGGCCATGATATTCGGGATATGCTTAGCGGGAGACCGGGGTCTCTGAGCCTCTCATGCCACTTCAGGGGGGAGGTTTGTTCCGCAGAGGACTTCAAGGTCGTGTTTACCCGCTACGGCAAGTGTTATACCTTCAACAGCGGTCGCGACGGGCGCCCTCGGCTTAAAACCATGAAAGGCGGCAGGAGACGAGCACCATAGG
asic1a_Frag498-660_insertion534	CACTTGAGGTCCGTCTCCTTACGATCGCGCTGGCCATGATATTCGGGATATGCTTCTCAGCGGGAGACCGGGGTCTCTGAGCTCATGCCACTTCAGGGGGGAGGTTTGTTCCGCAGAGGACTTCAAGGTCGTGTTTACCCGCTACGGCAAGTGTTATACCTTCAACAGCGGTCGCGACGGGCGCCCTCGGCTTAAAACCATGAAAGGCGGCAGGAGACGAGCACCATAGG
asic1a_Frag498-660_insertion537	CACTTGAGGTCCGTCTCCTTACGATCGCGCTGGCCATGATATTCGGGATATGCTTCTCTCAAGCGGGAGACCGGGGTCTCTGAGCTGCCACTTCAGGGGGGAGGTTTGTTCCGCAGAGGACTTCAAGGTCGTGTTTACCCGCTACGGCAAGTGTTATACCTTCAACAGCGGTCGCGACGGGCGCCCTCGGCTTAAAACCATGAAAGGCGGCAGGAGACGAGCACCATAGG
asic1a_Frag498-660_insertion540	CACTTGAGGTCCGTCTCCTTACGATCGCGCTGGCCATGATATTCGGGATATGCTTCTCTCATGCAGCGGGAGACCGGGGTCTCTGAGCCACTTCAGGGGGGAGGTTTGTTCCGCAGAGGACTTCAAGGTCGTGTTTACCCGCTACGGCAAGTGTTATACCTTCAACAGCGGTCGCGACGGGCGCCCTCGGCTTAAAACCATGAAAGGCGGCAGGAGACGAGCACCATAGG
asic1a_Frag498-660_insertion543	CACTTGAGGTCCGTCTCCTTACGATCGCGCTGGCCATGATATTCGGGATATGCTTCTCTCATGCCACAGCGGGAGACCGGGGTCTCTGAGCTTCAGGGGGGAGGTTTGTTCCGCAGAGGACTTCAAGGTCGTGTTTACCCGCTACGGCAAGTGTTATACCTTCAACAGCGGTCGCGACGGGCGCCCTCGGCTTAAAACCATGAAAGGCGGCAGGAGACGAGCACCATAGG
asic1a_Frag498-660_insertion546	CACTTGAGGTCCGTCTCCTTACGATCGCGCTGGCCATGATATTCGGGATATGCTTCTCTCATGCCACTTCAGCGGGAGACCGGGGTCTCTGAGCAGGGGGGAGGTTTGTTCCGCAGAGGACTTCAAGGTCGTGTTTACCCGCTACGGCAAGTGTTATACCTTCAACAGCGGTCGCGACGGGCGCCCTCGGCTTAAAACCATGAAAGGCGGCAGGAGACGAGCACCATAGG
asic1a_Frag498-660_insertion549	CACTTGAGGTCCGTCTCCTTACGATCGCGCTGGCCATGATATTCGGGATATGCTTCTCTCATGCCACTTCAGGAGCGGGAGACCGGGGTCTCTGAGCGGGGAGGTTTGTTCCGCAGAGGACTTCAAGGTCGTGTTTACCCGCTACGGCAAGTGTTATACCTTCAACAGCGGTCGCGACGGGCGCCCTCGGCTTAAAACCATGAAAGGCGGCAGGAGACGAGCACCATAGG
asic1a_Frag498-660_insertion552	CACTTGAGGTCCGTCTCCTTACGATCGCGCTGGCCATGATATTCGGGATATGCTTCTCTCATGCCACTTCAGGGGGAGCGGGAGACCGGGGTCTCTGAGCGAGGTTTGTTCCGCAGAGGACTTCAAGGTCGTGTTTACCCGCTACGGCAAGTGTTATACCTTCAACAGCGGTCGCGACGGGCGCCCTCGGCTTAAAACCATGAAAGGCGGCAGGAGACGAGCACCATAGG
asic1a_Frag498-660_insertion555	CACTTGAGGTCCGTCTCCTTACGATCGCGCTGGCCATGATATTCGGGATATGCTTCTCTCATGCCACTTCAGGGGGGAGAGCGGGAGACCGGGGTCTCTGAGCGTTTGTTCCGCAGAGGACTTCAAGGTCGTGTTTACCCGCTACGGCAAGTGTTATACCTTCAACAGCGGTCGCGACGGGCGCCCTCGGCTTAAAACCATGAAAGGCGGCAGGAGACGAGCACCATAGG
asic1a_Frag498-660_insertion558	CACTTGAGGTCCGTCTCCTTACGATCGCGCTGGCCATGATATTCGGGATATGCTTCTCTCATGCCACTTCAGGGGGGAGGTTAGCGGGAGACCGGGGTCTCTGAGCTGTTCCGCAGAGGACTTCAAGGTCGTGTTTACCCGCTACGGCAAGTGTTATACCTTCAACAGCGGTCGCGACGGGCGCCCTCGGCTTAAAACCATGAAAGGCGGCAGGAGACGAGCACCATAGG
asic1a_Frag498-660_insertion561	CACTTGAGGTCCGTCTCCTTACGATCGCGCTGGCCATGATATTCGGGATATGCTTCTCTCATGCCACTTCAGGGGGGAGGTTTGTAGCGGGAGACCGGGGTCTCTGAGCTCCGCAGAGGACTTCAAGGTCGTGTTTACCCGCTACGGCAAGTGTTATACCTTCAACAGCGGTCGCGACGGGCGCCCTCGGCTTAAAACCATGAAAGGCGGCAGGAGACGAGCACCATAGG
asic1a_Frag498-660_insertion564	CACTTGAGGTCCGTCTCCTTACGATCGCGCTGGCCATGATATTCGGGATATGCTTCTCTCATGCCACTTCAGGGGGGAGGTTTGTTCCAGCGGGAGACCGGGGTCTCTGAGCGCAGAGGACTTCAAGGTCGTGTTTACCCGCTACGGCAAGTGTTATACCTTCAACAGCGGTCGCGACGGGCGCCCTCGGCTTAAAACCATGAAAGGCGGCAGGAGACGAGCACCATAGG
asic1a_Frag498-660_insertion567	CACTTGAGGTCCGTCTCCTTACGATCGCGCTGGCCATGATATTCGGGATATGCTTCTCTCATGCCACTTCAGGGGGGAGGTTTGTTCCGCAAGCGGGAGACCGGGGTCTCTGAGCGAGGACTTCAAGGTCGTGTTTACCCGCTACGGCAAGTGTTATACCTTCAACAGCGGTCGCGACGGGCGCCCTCGGCTTAAAACCATGAAAGGCGGCAGGAGACGAGCACCATAGG
asic1a_Frag498-660_insertion570	CACTTGAGGTCCGTCTCCTTACGATCGCGCTGGCCATGATATTCGGGATATGCTTCTCTCATGCCACTTCAGGGGGGAGGTTTGTTCCGCAGAGAGCGGGAGACCGGGGTCTCTGAGCGACTTCAAGGTCGTGTTTACCCGCTACGGCAAGTGTTATACCTTCAACAGCGGTCGCGACGGGCGCCCTCGGCTTAAAACCATGAAAGGCGGCAGGAGACGAGCACCATAGG
asic1a_Frag498-660_insertion573	CACTTGAGGTCCGTCTCCTTACGATCGCGCTGGCCATGATATTCGGGATATGCTTCTCTCATGCCACTTCAGGGGGGAGGTTTGTTCCGCAGAGGACAGCGGGAGACCGGGGTCTCTGAGCTTCAAGGTCGTGTTTACCCGCTACGGCAAGTGTTATACCTTCAACAGCGGTCGCGACGGGCGCCCTCGGCTTAAAACCATGAAAGGCGGCAGGAGACGAGCACCATAGG
asic1a_Frag498-660_insertion576	CACTTGAGGTCCGTCTCCTTACGATCGCGCTGGCCATGATATTCGGGATATGCTTCTCTCATGCCACTTCAGGGGGGAGGTTTGTTCCGCAGAGGACTTCAGCGGGAGACCGGGGTCTCTGAGCAAGGTCGTGTTTACCCGCTACGGCAAGTGTTATACCTTCAACAGCGGTCGCGACGGGCGCCCTCGGCTTAAAACCATGAAAGGCGGCAGGAGACGAGCACCATAGG
asic1a_Frag498-660_insertion579	CACTTGAGGTCCGTCTCCTTACGATCGCGCTGGCCATGATATTCGGGATATGCTTCTCTCATGCCACTTCAGGGGGGAGGTTTGTTCCGCAGAGGACTTCAAGAGCGGGAGACCGGGGTCTCTGAGCGTCGTGTTTACCCGCTACGGCAAGTGTTATACCTTCAACAGCGGTCGCGACGGGCGCCCTCGGCTTAAAACCATGAAAGGCGGCAGGAGACGAGCACCATAGG
asic1a_Frag498-660_insertion582	CACTTGAGGTCCGTCTCCTTACGATCGCGCTGGCCATGATATTCGGGATATGCTTCTCTCATGCCACTTCAGGGGGGAGGTTTGTTCCGCAGAGGACTTCAAGGTCAGCGGGAGACCGGGGTCTCTGAGCGTGTTTACCCGCTACGGCAAGTGTTATACCTTCAACAGCGGTCGCGACGGGCGCCCTCGGCTTAAAACCATGAAAGGCGGCAGGAGACGAGCACCATAGG
asic1a_Frag498-660_insertion585	CACTTGAGGTCCGTCTCCTTACGATCGCGCTGGCCATGATATTCGGGATATGCTTCTCTCATGCCACTTCAGGGGGGAGGTTTGTTCCGCAGAGGACTTCAAGGTCGTGAGCGGGAGACCGGGGTCTCTGAGCTTTACCCGCTACGGCAAGTGTTATACCTTCAACAGCGGTCGCGACGGGCGCCCTCGGCTTAAAACCATGAAAGGCGGCAGGAGACGAGCACCATAGG
asic1a_Frag498-660_insertion588	CACTTGAGGTCCGTCTCCTTACGATCGCGCTGGCCATGATATTCGGGATATGCTTCTCTCATGCCACTTCAGGGGGGAGGTTTGTTCCGCAGAGGACTTCAAGGTCGTGTTTAGCGGGAGACCGGGGTCTCTGAGCACCCGCTACGGCAAGTGTTATACCTTCAACAGCGGTCGCGACGGGCGCCCTCGGCTTAAAACCATGAAAGGCGGCAGGAGACGAGCACCATAGG
asic1a_Frag498-660_insertion591	CACTTGAGGTCCGTCTCCTTACGATCGCGCTGGCCATGATATTCGGGATATGCTTCTCTCATGCCACTTCAGGGGGGAGGTTTGTTCCGCAGAGGACTTCAAGGTCGTGTTTACCAGCGGGAGACCGGGGTCTCTGAGCCGCTACGGCAAGTGTTATACCTTCAACAGCGGTCGCGACGGGCGCCCTCGGCTTAAAACCATGAAAGGCGGCAGGAGACGAGCACCATAGG
asic1a_Frag498-660_insertion594	CACTTGAGGTCCGTCTCCTTACGATCGCGCTGGCCATGATATTCGGGATATGCTTCTCTCATGCCACTTCAGGGGGGAGGTTTGTTCCGCAGAGGACTTCAAGGTCGTGTTTACCCGCAGCGGGAGACCGGGGTCTCTGAGCTACGGCAAGTGTTATACCTTCAACAGCGGTCGCGACGGGCGCCCTCGGCTTAAAACCATGAAAGGCGGCAGGAGACGAGCACCATAGG
asic1a_Frag498-660_insertion597	CACTTGAGGTCCGTCTCCTTACGATCGCGCTGGCCATGATATTCGGGATATGCTTCTCTCATGCCACTTCAGGGGGGAGGTTTGTTCCGCAGAGGACTTCAAGGTCGTGTTTACCCGCTACAGCGGGAGACCGGGGTCTCTGAGCGGCAAGTGTTATACCTTCAACAGCGGTCGCGACGGGCGCCCTCGGCTTAAAACCATGAAAGGCGGCAGGAGACGAGCACCATAGG
asic1a_Frag498-660_insertion600	CACTTGAGGTCCGTCTCCTTACGATCGCGCTGGCCATGATATTCGGGATATGCTTCTCTCATGCCACTTCAGGGGGGAGGTTTGTTCCGCAGAGGACTTCAAGGTCGTGTTTACCCGCTACGGCAGCGGGAGACCGGGGTCTCTGAGCAAGTGTTATACCTTCAACAGCGGTCGCGACGGGCGCCCTCGGCTTAAAACCATGAAAGGCGGCAGGAGACGAGCACCATAGG
asic1a_Frag498-660_insertion603	CACTTGAGGTCCGTCTCCTTACGATCGCGCTGGCCATGATATTCGGGATATGCTTCTCTCATGCCACTTCAGGGGGGAGGTTTGTTCCGCAGAGGACTTCAAGGTCGTGTTTACCCGCTACGGCAAGAGCGGGAGACCGGGGTCTCTGAGCTGTTATACCTTCAACAGCGGTCGCGACGGGCGCCCTCGGCTTAAAACCATGAAAGGCGGCAGGAGACGAGCACCATAGG
asic1a_Frag498-660_insertion606	CACTTGAGGTCCGTCTCCTTACGATCGCGCTGGCCATGATATTCGGGATATGCTTCTCTCATGCCACTTCAGGGGGGAGGTTTGTTCCGCAGAGGACTTCAAGGTCGTGTTTACCCGCTACGGCAAGTGTAGCGGGAGACCGGGGTCTCTGAGCTATACCTTCAACAGCGGTCGCGACGGGCGCCCTCGGCTTAAAACCATGAAAGGCGGCAGGAGACGAGCACCATAGG
asic1a_Frag498-660_insertion609	CACTTGAGGTCCGTCTCCTTACGATCGCGCTGGCCATGATATTCGGGATATGCTTCTCTCATGCCACTTCAGGGGGGAGGTTTGTTCCGCAGAGGACTTCAAGGTCGTGTTTACCCGCTACGGCAAGTGTTATAGCGGGAGACCGGGGTCTCTGAGCACCTTCAACAGCGGTCGCGACGGGCGCCCTCGGCTTAAAACCATGAAAGGCGGCAGGAGACGAGCACCATAGG
asic1a_Frag498-660_insertion612	CACTTGAGGTCCGTCTCCTTACGATCGCGCTGGCCATGATATTCGGGATATGCTTCTCTCATGCCACTTCAGGGGGGAGGTTTGTTCCGCAGAGGACTTCAAGGTCGTGTTTACCCGCTACGGCAAGTGTTATACCAGCGGGAGACCGGGGTCTCTGAGCTTCAACAGCGGTCGCGACGGGCGCCCTCGGCTTAAAACCATGAAAGGCGGCAGGAGACGAGCACCATAGG
asic1a_Frag498-660_insertion615	CACTTGAGGTCCGTCTCCTTACGATCGCGCTGGCCATGATATTCGGGATATGCTTCTCTCATGCCACTTCAGGGGGGAGGTTTGTTCCGCAGAGGACTTCAAGGTCGTGTTTACCCGCTACGGCAAGTGTTATACCTTCAGCGGGAGACCGGGGTCTCTGAGCAACAGCGGTCGCGACGGGCGCCCTCGGCTTAAAACCATGAAAGGCGGCAGGAGACGAGCACCATAGG
asic1a_Frag498-660_insertion618	CACTTGAGGTCCGTCTCCTTACGATCGCGCTGGCCATGATATTCGGGATATGCTTCTCTCATGCCACTTCAGGGGGGAGGTTTGTTCCGCAGAGGACTTCAAGGTCGTGTTTACCCGCTACGGCAAGTGTTATACCTTCAACAGCGGGAGACCGGGGTCTCTGAGCAGCGGTCGCGACGGGCGCCCTCGGCTTAAAACCATGAAAGGCGGCAGGAGACGAGCACCATAGG
asic1a_Frag498-660_insertion621	CACTTGAGGTCCGTCTCCTTACGATCGCGCTGGCCATGATATTCGGGATATGCTTCTCTCATGCCACTTCAGGGGGGAGGTTTGTTCCGCAGAGGACTTCAAGGTCGTGTTTACCCGCTACGGCAAGTGTTATACCTTCAACAGCAGCGGGAGACCGGGGTCTCTGAGCGGTCGCGACGGGCGCCCTCGGCTTAAAACCATGAAAGGCGGCAGGAGACGAGCACCATAGG
asic1a_Frag498-660_insertion624	CACTTGAGGTCCGTCTCCTTACGATCGCGCTGGCCATGATATTCGGGATATGCTTCTCTCATGCCACTTCAGGGGGGAGGTTTGTTCCGCAGAGGACTTCAAGGTCGTGTTTACCCGCTACGGCAAGTGTTATACCTTCAACAGCGGTAGCGGGAGACCGGGGTCTCTGAGCCGCGACGGGCGCCCTCGGCTTAAAACCATGAAAGGCGGCAGGAGACGAGCACCATAGG
asic1a_Frag498-660_insertion627	CACTTGAGGTCCGTCTCCTTACGATCGCGCTGGCCATGATATTCGGGATATGCTTCTCTCATGCCACTTCAGGGGGGAGGTTTGTTCCGCAGAGGACTTCAAGGTCGTGTTTACCCGCTACGGCAAGTGTTATACCTTCAACAGCGGTCGCAGCGGGAGACCGGGGTCTCTGAGCGACGGGCGCCCTCGGCTTAAAACCATGAAAGGCGGCAGGAGACGAGCACCATAGG
asic1a_Frag498-660_insertion630	CACTTGAGGTCCGTCTCCTTACGATCGCGCTGGCCATGATATTCGGGATATGCTTCTCTCATGCCACTTCAGGGGGGAGGTTTGTTCCGCAGAGGACTTCAAGGTCGTGTTTACCCGCTACGGCAAGTGTTATACCTTCAACAGCGGTCGCGACAGCGGGAGACCGGGGTCTCTGAGCGGGCGCCCTCGGCTTAAAACCATGAAAGGCGGCAGGAGACGAGCACCATAGG
asic1a_Frag498-660_insertion633	CACTTGAGGTCCGTCTCCTTACGATCGCGCTGGCCATGATATTCGGGATATGCTTCTCTCATGCCACTTCAGGGGGGAGGTTTGTTCCGCAGAGGACTTCAAGGTCGTGTTTACCCGCTACGGCAAGTGTTATACCTTCAACAGCGGTCGCGACGGGAGCGGGAGACCGGGGTCTCTGAGCCGCCCTCGGCTTAAAACCATGAAAGGCGGCAGGAGACGAGCACCATAGG
asic1a_Frag498-660_insertion636	CACTTGAGGTCCGTCTCCTTACGATCGCGCTGGCCATGATATTCGGGATATGCTTCTCTCATGCCACTTCAGGGGGGAGGTTTGTTCCGCAGAGGACTTCAAGGTCGTGTTTACCCGCTACGGCAAGTGTTATACCTTCAACAGCGGTCGCGACGGGCGCAGCGGGAGACCGGGGTCTCTGAGCCCTCGGCTTAAAACCATGAAAGGCGGCAGGAGACGAGCACCATAGG
asic1a_Frag498-660_insertion639	CACTTGAGGTCCGTCTCCTTACGATCGCGCTGGCCATGATATTCGGGATATGCTTCTCTCATGCCACTTCAGGGGGGAGGTTTGTTCCGCAGAGGACTTCAAGGTCGTGTTTACCCGCTACGGCAAGTGTTATACCTTCAACAGCGGTCGCGACGGGCGCCCTAGCGGGAGACCGGGGTCTCTGAGCCGGCTTAAAACCATGAAAGGCGGCAGGAGACGAGCACCATAGG
asic1a_Frag498-660_insertion642	CACTTGAGGTCCGTCTCCTTACGATCGCGCTGGCCATGATATTCGGGATATGCTTCTCTCATGCCACTTCAGGGGGGAGGTTTGTTCCGCAGAGGACTTCAAGGTCGTGTTTACCCGCTACGGCAAGTGTTATACCTTCAACAGCGGTCGCGACGGGCGCCCTCGGAGCGGGAGACCGGGGTCTCTGAGCCTTAAAACCATGAAAGGCGGCAGGAGACGAGCACCATAGG
asic1a_Frag498-660_insertion645	CACTTGAGGTCCGTCTCCTTACGATCGCGCTGGCCATGATATTCGGGATATGCTTCTCTCATGCCACTTCAGGGGGGAGGTTTGTTCCGCAGAGGACTTCAAGGTCGTGTTTACCCGCTACGGCAAGTGTTATACCTTCAACAGCGGTCGCGACGGGCGCCCTCGGCTTAGCGGGAGACCGGGGTCTCTGAGCAAAACCATGAAAGGCGGCAGGAGACGAGCACCATAGG
asic1a_Frag498-660_insertion648	CACTTGAGGTCCGTCTCCTTACGATCGCGCTGGCCATGATATTCGGGATATGCTTCTCTCATGCCACTTCAGGGGGGAGGTTTGTTCCGCAGAGGACTTCAAGGTCGTGTTTACCCGCTACGGCAAGTGTTATACCTTCAACAGCGGTCGCGACGGGCGCCCTCGGCTTAAAAGCGGGAGACCGGGGTCTCTGAGCACCATGAAAGGCGGCAGGAGACGAGCACCATAGG
asic1a_Frag498-660_insertion651	CACTTGAGGTCCGTCTCCTTACGATCGCGCTGGCCATGATATTCGGGATATGCTTCTCTCATGCCACTTCAGGGGGGAGGTTTGTTCCGCAGAGGACTTCAAGGTCGTGTTTACCCGCTACGGCAAGTGTTATACCTTCAACAGCGGTCGCGACGGGCGCCCTCGGCTTAAAACCAGCGGGAGACCGGGGTCTCTGAGCATGAAAGGCGGCAGGAGACGAGCACCATAGG
asic1a_Frag498-660_insertion654	CACTTGAGGTCCGTCTCCTTACGATCGCGCTGGCCATGATATTCGGGATATGCTTCTCTCATGCCACTTCAGGGGGGAGGTTTGTTCCGCAGAGGACTTCAAGGTCGTGTTTACCCGCTACGGCAAGTGTTATACCTTCAACAGCGGTCGCGACGGGCGCCCTCGGCTTAAAACCATGAGCGGGAGACCGGGGTCTCTGAGCAAAGGCGGCAGGAGACGAGCACCATAGG
asic1a_Frag498-660_insertion657	CACTTGAGGTCCGTCTCCTTACGATCGCGCTGGCCATGATATTCGGGATATGCTTCTCTCATGCCACTTCAGGGGGGAGGTTTGTTCCGCAGAGGACTTCAAGGTCGTGTTTACCCGCTACGGCAAGTGTTATACCTTCAACAGCGGTCGCGACGGGCGCCCTCGGCTTAAAACCATGAAAAGCGGGAGACCGGGGTCTCTGAGCGGCGGCAGGAGACGAGCACCATAGG
asic1a_Frag498-660_insertion660	CACTTGAGGTCCGTCTCCTTACGATCGCGCTGGCCATGATATTCGGGATATGCTTCTCTCATGCCACTTCAGGGGGGAGGTTTGTTCCGCAGAGGACTTCAAGGTCGTGTTTACCCGCTACGGCAAGTGTTATACCTTCAACAGCGGTCGCGACGGGCGCCCTCGGCTTAAAACCATGAAAGGCAGCGGGAGACCGGGGTCTCTGAGCGGCAGGAGACGAGCACCATAGG
asic1a_Frag663-825_insertion663	AAATGTTGTTCCGTCTCCCGGCAGCGGGAGACCGGGGTCTCTGAGCACTGGTAACGGACTCGAAATCATGCTGGACATCCAACAAGATGAATACCTCCCCGTGTGGGGTGAAACAGATGAAACCAGTTTCGAAGCTGGTATAAAGGTACAAATACATAGCCAAGATGAGCCCCCCTTTATTGACCAACTTGGCTTCGGTGTAGCACCCGGATGGAGACGGCCATAAGTGA
asic1a_Frag663-825_insertion666	AAATGTTGTTCCGTCTCCCGGCACTAGCGGGAGACCGGGGTCTCTGAGCGGTAACGGACTCGAAATCATGCTGGACATCCAACAAGATGAATACCTCCCCGTGTGGGGTGAAACAGATGAAACCAGTTTCGAAGCTGGTATAAAGGTACAAATACATAGCCAAGATGAGCCCCCCTTTATTGACCAACTTGGCTTCGGTGTAGCACCCGGATGGAGACGGCCATAAGTGA
asic1a_Frag663-825_insertion669	AAATGTTGTTCCGTCTCCCGGCACTGGTAGCGGGAGACCGGGGTCTCTGAGCAACGGACTCGAAATCATGCTGGACATCCAACAAGATGAATACCTCCCCGTGTGGGGTGAAACAGATGAAACCAGTTTCGAAGCTGGTATAAAGGTACAAATACATAGCCAAGATGAGCCCCCCTTTATTGACCAACTTGGCTTCGGTGTAGCACCCGGATGGAGACGGCCATAAGTGA
asic1a_Frag663-825_insertion672	AAATGTTGTTCCGTCTCCCGGCACTGGTAACAGCGGGAGACCGGGGTCTCTGAGCGGACTCGAAATCATGCTGGACATCCAACAAGATGAATACCTCCCCGTGTGGGGTGAAACAGATGAAACCAGTTTCGAAGCTGGTATAAAGGTACAAATACATAGCCAAGATGAGCCCCCCTTTATTGACCAACTTGGCTTCGGTGTAGCACCCGGATGGAGACGGCCATAAGTGA
asic1a_Frag663-825_insertion675	AAATGTTGTTCCGTCTCCCGGCACTGGTAACGGAAGCGGGAGACCGGGGTCTCTGAGCCTCGAAATCATGCTGGACATCCAACAAGATGAATACCTCCCCGTGTGGGGTGAAACAGATGAAACCAGTTTCGAAGCTGGTATAAAGGTACAAATACATAGCCAAGATGAGCCCCCCTTTATTGACCAACTTGGCTTCGGTGTAGCACCCGGATGGAGACGGCCATAAGTGA
asic1a_Frag663-825_insertion678	AAATGTTGTTCCGTCTCCCGGCACTGGTAACGGACTCAGCGGGAGACCGGGGTCTCTGAGCGAAATCATGCTGGACATCCAACAAGATGAATACCTCCCCGTGTGGGGTGAAACAGATGAAACCAGTTTCGAAGCTGGTATAAAGGTACAAATACATAGCCAAGATGAGCCCCCCTTTATTGACCAACTTGGCTTCGGTGTAGCACCCGGATGGAGACGGCCATAAGTGA
asic1a_Frag663-825_insertion681	AAATGTTGTTCCGTCTCCCGGCACTGGTAACGGACTCGAAAGCGGGAGACCGGGGTCTCTGAGCATCATGCTGGACATCCAACAAGATGAATACCTCCCCGTGTGGGGTGAAACAGATGAAACCAGTTTCGAAGCTGGTATAAAGGTACAAATACATAGCCAAGATGAGCCCCCCTTTATTGACCAACTTGGCTTCGGTGTAGCACCCGGATGGAGACGGCCATAAGTGA
asic1a_Frag663-825_insertion684	AAATGTTGTTCCGTCTCCCGGCACTGGTAACGGACTCGAAATCAGCGGGAGACCGGGGTCTCTGAGCATGCTGGACATCCAACAAGATGAATACCTCCCCGTGTGGGGTGAAACAGATGAAACCAGTTTCGAAGCTGGTATAAAGGTACAAATACATAGCCAAGATGAGCCCCCCTTTATTGACCAACTTGGCTTCGGTGTAGCACCCGGATGGAGACGGCCATAAGTGA
asic1a_Frag663-825_insertion687	AAATGTTGTTCCGTCTCCCGGCACTGGTAACGGACTCGAAATCATGAGCGGGAGACCGGGGTCTCTGAGCCTGGACATCCAACAAGATGAATACCTCCCCGTGTGGGGTGAAACAGATGAAACCAGTTTCGAAGCTGGTATAAAGGTACAAATACATAGCCAAGATGAGCCCCCCTTTATTGACCAACTTGGCTTCGGTGTAGCACCCGGATGGAGACGGCCATAAGTGA
asic1a_Frag663-825_insertion690	AAATGTTGTTCCGTCTCCCGGCACTGGTAACGGACTCGAAATCATGCTGAGCGGGAGACCGGGGTCTCTGAGCGACATCCAACAAGATGAATACCTCCCCGTGTGGGGTGAAACAGATGAAACCAGTTTCGAAGCTGGTATAAAGGTACAAATACATAGCCAAGATGAGCCCCCCTTTATTGACCAACTTGGCTTCGGTGTAGCACCCGGATGGAGACGGCCATAAGTGA
asic1a_Frag663-825_insertion693	AAATGTTGTTCCGTCTCCCGGCACTGGTAACGGACTCGAAATCATGCTGGACAGCGGGAGACCGGGGTCTCTGAGCATCCAACAAGATGAATACCTCCCCGTGTGGGGTGAAACAGATGAAACCAGTTTCGAAGCTGGTATAAAGGTACAAATACATAGCCAAGATGAGCCCCCCTTTATTGACCAACTTGGCTTCGGTGTAGCACCCGGATGGAGACGGCCATAAGTGA
asic1a_Frag663-825_insertion696	AAATGTTGTTCCGTCTCCCGGCACTGGTAACGGACTCGAAATCATGCTGGACATCAGCGGGAGACCGGGGTCTCTGAGCCAACAAGATGAATACCTCCCCGTGTGGGGTGAAACAGATGAAACCAGTTTCGAAGCTGGTATAAAGGTACAAATACATAGCCAAGATGAGCCCCCCTTTATTGACCAACTTGGCTTCGGTGTAGCACCCGGATGGAGACGGCCATAAGTGA
asic1a_Frag663-825_insertion699	AAATGTTGTTCCGTCTCCCGGCACTGGTAACGGACTCGAAATCATGCTGGACATCCAAAGCGGGAGACCGGGGTCTCTGAGCCAAGATGAATACCTCCCCGTGTGGGGTGAAACAGATGAAACCAGTTTCGAAGCTGGTATAAAGGTACAAATACATAGCCAAGATGAGCCCCCCTTTATTGACCAACTTGGCTTCGGTGTAGCACCCGGATGGAGACGGCCATAAGTGA
asic1a_Frag663-825_insertion702	AAATGTTGTTCCGTCTCCCGGCACTGGTAACGGACTCGAAATCATGCTGGACATCCAACAAAGCGGGAGACCGGGGTCTCTGAGCGATGAATACCTCCCCGTGTGGGGTGAAACAGATGAAACCAGTTTCGAAGCTGGTATAAAGGTACAAATACATAGCCAAGATGAGCCCCCCTTTATTGACCAACTTGGCTTCGGTGTAGCACCCGGATGGAGACGGCCATAAGTGA
asic1a_Frag663-825_insertion705	AAATGTTGTTCCGTCTCCCGGCACTGGTAACGGACTCGAAATCATGCTGGACATCCAACAAGATAGCGGGAGACCGGGGTCTCTGAGCGAATACCTCCCCGTGTGGGGTGAAACAGATGAAACCAGTTTCGAAGCTGGTATAAAGGTACAAATACATAGCCAAGATGAGCCCCCCTTTATTGACCAACTTGGCTTCGGTGTAGCACCCGGATGGAGACGGCCATAAGTGA
asic1a_Frag663-825_insertion708	AAATGTTGTTCCGTCTCCCGGCACTGGTAACGGACTCGAAATCATGCTGGACATCCAACAAGATGAAAGCGGGAGACCGGGGTCTCTGAGCTACCTCCCCGTGTGGGGTGAAACAGATGAAACCAGTTTCGAAGCTGGTATAAAGGTACAAATACATAGCCAAGATGAGCCCCCCTTTATTGACCAACTTGGCTTCGGTGTAGCACCCGGATGGAGACGGCCATAAGTGA
asic1a_Frag663-825_insertion711	AAATGTTGTTCCGTCTCCCGGCACTGGTAACGGACTCGAAATCATGCTGGACATCCAACAAGATGAATACAGCGGGAGACCGGGGTCTCTGAGCCTCCCCGTGTGGGGTGAAACAGATGAAACCAGTTTCGAAGCTGGTATAAAGGTACAAATACATAGCCAAGATGAGCCCCCCTTTATTGACCAACTTGGCTTCGGTGTAGCACCCGGATGGAGACGGCCATAAGTGA
asic1a_Frag663-825_insertion714	AAATGTTGTTCCGTCTCCCGGCACTGGTAACGGACTCGAAATCATGCTGGACATCCAACAAGATGAATACCTCAGCGGGAGACCGGGGTCTCTGAGCCCCGTGTGGGGTGAAACAGATGAAACCAGTTTCGAAGCTGGTATAAAGGTACAAATACATAGCCAAGATGAGCCCCCCTTTATTGACCAACTTGGCTTCGGTGTAGCACCCGGATGGAGACGGCCATAAGTGA
asic1a_Frag663-825_insertion717	AAATGTTGTTCCGTCTCCCGGCACTGGTAACGGACTCGAAATCATGCTGGACATCCAACAAGATGAATACCTCCCCAGCGGGAGACCGGGGTCTCTGAGCGTGTGGGGTGAAACAGATGAAACCAGTTTCGAAGCTGGTATAAAGGTACAAATACATAGCCAAGATGAGCCCCCCTTTATTGACCAACTTGGCTTCGGTGTAGCACCCGGATGGAGACGGCCATAAGTGA
asic1a_Frag663-825_insertion720	AAATGTTGTTCCGTCTCCCGGCACTGGTAACGGACTCGAAATCATGCTGGACATCCAACAAGATGAATACCTCCCCGTGAGCGGGAGACCGGGGTCTCTGAGCTGGGGTGAAACAGATGAAACCAGTTTCGAAGCTGGTATAAAGGTACAAATACATAGCCAAGATGAGCCCCCCTTTATTGACCAACTTGGCTTCGGTGTAGCACCCGGATGGAGACGGCCATAAGTGA
asic1a_Frag663-825_insertion723	AAATGTTGTTCCGTCTCCCGGCACTGGTAACGGACTCGAAATCATGCTGGACATCCAACAAGATGAATACCTCCCCGTGTGGAGCGGGAGACCGGGGTCTCTGAGCGGTGAAACAGATGAAACCAGTTTCGAAGCTGGTATAAAGGTACAAATACATAGCCAAGATGAGCCCCCCTTTATTGACCAACTTGGCTTCGGTGTAGCACCCGGATGGAGACGGCCATAAGTGA
asic1a_Frag663-825_insertion726	AAATGTTGTTCCGTCTCCCGGCACTGGTAACGGACTCGAAATCATGCTGGACATCCAACAAGATGAATACCTCCCCGTGTGGGGTAGCGGGAGACCGGGGTCTCTGAGCGAAACAGATGAAACCAGTTTCGAAGCTGGTATAAAGGTACAAATACATAGCCAAGATGAGCCCCCCTTTATTGACCAACTTGGCTTCGGTGTAGCACCCGGATGGAGACGGCCATAAGTGA
asic1a_Frag663-825_insertion729	AAATGTTGTTCCGTCTCCCGGCACTGGTAACGGACTCGAAATCATGCTGGACATCCAACAAGATGAATACCTCCCCGTGTGGGGTGAAAGCGGGAGACCGGGGTCTCTGAGCACAGATGAAACCAGTTTCGAAGCTGGTATAAAGGTACAAATACATAGCCAAGATGAGCCCCCCTTTATTGACCAACTTGGCTTCGGTGTAGCACCCGGATGGAGACGGCCATAAGTGA
asic1a_Frag663-825_insertion732	AAATGTTGTTCCGTCTCCCGGCACTGGTAACGGACTCGAAATCATGCTGGACATCCAACAAGATGAATACCTCCCCGTGTGGGGTGAAACAAGCGGGAGACCGGGGTCTCTGAGCGATGAAACCAGTTTCGAAGCTGGTATAAAGGTACAAATACATAGCCAAGATGAGCCCCCCTTTATTGACCAACTTGGCTTCGGTGTAGCACCCGGATGGAGACGGCCATAAGTGA
asic1a_Frag663-825_insertion735	AAATGTTGTTCCGTCTCCCGGCACTGGTAACGGACTCGAAATCATGCTGGACATCCAACAAGATGAATACCTCCCCGTGTGGGGTGAAACAGATAGCGGGAGACCGGGGTCTCTGAGCGAAACCAGTTTCGAAGCTGGTATAAAGGTACAAATACATAGCCAAGATGAGCCCCCCTTTATTGACCAACTTGGCTTCGGTGTAGCACCCGGATGGAGACGGCCATAAGTGA
asic1a_Frag663-825_insertion738	AAATGTTGTTCCGTCTCCCGGCACTGGTAACGGACTCGAAATCATGCTGGACATCCAACAAGATGAATACCTCCCCGTGTGGGGTGAAACAGATGAAAGCGGGAGACCGGGGTCTCTGAGCACCAGTTTCGAAGCTGGTATAAAGGTACAAATACATAGCCAAGATGAGCCCCCCTTTATTGACCAACTTGGCTTCGGTGTAGCACCCGGATGGAGACGGCCATAAGTGA
asic1a_Frag663-825_insertion741	AAATGTTGTTCCGTCTCCCGGCACTGGTAACGGACTCGAAATCATGCTGGACATCCAACAAGATGAATACCTCCCCGTGTGGGGTGAAACAGATGAAACCAGCGGGAGACCGGGGTCTCTGAGCAGTTTCGAAGCTGGTATAAAGGTACAAATACATAGCCAAGATGAGCCCCCCTTTATTGACCAACTTGGCTTCGGTGTAGCACCCGGATGGAGACGGCCATAAGTGA
asic1a_Frag663-825_insertion744	AAATGTTGTTCCGTCTCCCGGCACTGGTAACGGACTCGAAATCATGCTGGACATCCAACAAGATGAATACCTCCCCGTGTGGGGTGAAACAGATGAAACCAGTAGCGGGAGACCGGGGTCTCTGAGCTTCGAAGCTGGTATAAAGGTACAAATACATAGCCAAGATGAGCCCCCCTTTATTGACCAACTTGGCTTCGGTGTAGCACCCGGATGGAGACGGCCATAAGTGA
asic1a_Frag663-825_insertion747	AAATGTTGTTCCGTCTCCCGGCACTGGTAACGGACTCGAAATCATGCTGGACATCCAACAAGATGAATACCTCCCCGTGTGGGGTGAAACAGATGAAACCAGTTTCAGCGGGAGACCGGGGTCTCTGAGCGAAGCTGGTATAAAGGTACAAATACATAGCCAAGATGAGCCCCCCTTTATTGACCAACTTGGCTTCGGTGTAGCACCCGGATGGAGACGGCCATAAGTGA
asic1a_Frag663-825_insertion750	AAATGTTGTTCCGTCTCCCGGCACTGGTAACGGACTCGAAATCATGCTGGACATCCAACAAGATGAATACCTCCCCGTGTGGGGTGAAACAGATGAAACCAGTTTCGAAAGCGGGAGACCGGGGTCTCTGAGCGCTGGTATAAAGGTACAAATACATAGCCAAGATGAGCCCCCCTTTATTGACCAACTTGGCTTCGGTGTAGCACCCGGATGGAGACGGCCATAAGTGA
asic1a_Frag663-825_insertion753	AAATGTTGTTCCGTCTCCCGGCACTGGTAACGGACTCGAAATCATGCTGGACATCCAACAAGATGAATACCTCCCCGTGTGGGGTGAAACAGATGAAACCAGTTTCGAAGCTAGCGGGAGACCGGGGTCTCTGAGCGGTATAAAGGTACAAATACATAGCCAAGATGAGCCCCCCTTTATTGACCAACTTGGCTTCGGTGTAGCACCCGGATGGAGACGGCCATAAGTGA
asic1a_Frag663-825_insertion756	AAATGTTGTTCCGTCTCCCGGCACTGGTAACGGACTCGAAATCATGCTGGACATCCAACAAGATGAATACCTCCCCGTGTGGGGTGAAACAGATGAAACCAGTTTCGAAGCTGGTAGCGGGAGACCGGGGTCTCTGAGCATAAAGGTACAAATACATAGCCAAGATGAGCCCCCCTTTATTGACCAACTTGGCTTCGGTGTAGCACCCGGATGGAGACGGCCATAAGTGA
asic1a_Frag663-825_insertion759	AAATGTTGTTCCGTCTCCCGGCACTGGTAACGGACTCGAAATCATGCTGGACATCCAACAAGATGAATACCTCCCCGTGTGGGGTGAAACAGATGAAACCAGTTTCGAAGCTGGTATAAGCGGGAGACCGGGGTCTCTGAGCAAGGTACAAATACATAGCCAAGATGAGCCCCCCTTTATTGACCAACTTGGCTTCGGTGTAGCACCCGGATGGAGACGGCCATAAGTGA
asic1a_Frag663-825_insertion762	AAATGTTGTTCCGTCTCCCGGCACTGGTAACGGACTCGAAATCATGCTGGACATCCAACAAGATGAATACCTCCCCGTGTGGGGTGAAACAGATGAAACCAGTTTCGAAGCTGGTATAAAGAGCGGGAGACCGGGGTCTCTGAGCGTACAAATACATAGCCAAGATGAGCCCCCCTTTATTGACCAACTTGGCTTCGGTGTAGCACCCGGATGGAGACGGCCATAAGTGA
asic1a_Frag663-825_insertion765	AAATGTTGTTCCGTCTCCCGGCACTGGTAACGGACTCGAAATCATGCTGGACATCCAACAAGATGAATACCTCCCCGTGTGGGGTGAAACAGATGAAACCAGTTTCGAAGCTGGTATAAAGGTAAGCGGGAGACCGGGGTCTCTGAGCCAAATACATAGCCAAGATGAGCCCCCCTTTATTGACCAACTTGGCTTCGGTGTAGCACCCGGATGGAGACGGCCATAAGTGA
asic1a_Frag663-825_insertion768	AAATGTTGTTCCGTCTCCCGGCACTGGTAACGGACTCGAAATCATGCTGGACATCCAACAAGATGAATACCTCCCCGTGTGGGGTGAAACAGATGAAACCAGTTTCGAAGCTGGTATAAAGGTACAAAGCGGGAGACCGGGGTCTCTGAGCATACATAGCCAAGATGAGCCCCCCTTTATTGACCAACTTGGCTTCGGTGTAGCACCCGGATGGAGACGGCCATAAGTGA
asic1a_Frag663-825_insertion771	AAATGTTGTTCCGTCTCCCGGCACTGGTAACGGACTCGAAATCATGCTGGACATCCAACAAGATGAATACCTCCCCGTGTGGGGTGAAACAGATGAAACCAGTTTCGAAGCTGGTATAAAGGTACAAATAAGCGGGAGACCGGGGTCTCTGAGCCATAGCCAAGATGAGCCCCCCTTTATTGACCAACTTGGCTTCGGTGTAGCACCCGGATGGAGACGGCCATAAGTGA
asic1a_Frag663-825_insertion774	AAATGTTGTTCCGTCTCCCGGCACTGGTAACGGACTCGAAATCATGCTGGACATCCAACAAGATGAATACCTCCCCGTGTGGGGTGAAACAGATGAAACCAGTTTCGAAGCTGGTATAAAGGTACAAATACATAGCGGGAGACCGGGGTCTCTGAGCAGCCAAGATGAGCCCCCCTTTATTGACCAACTTGGCTTCGGTGTAGCACCCGGATGGAGACGGCCATAAGTGA
asic1a_Frag663-825_insertion777	AAATGTTGTTCCGTCTCCCGGCACTGGTAACGGACTCGAAATCATGCTGGACATCCAACAAGATGAATACCTCCCCGTGTGGGGTGAAACAGATGAAACCAGTTTCGAAGCTGGTATAAAGGTACAAATACATAGCAGCGGGAGACCGGGGTCTCTGAGCCAAGATGAGCCCCCCTTTATTGACCAACTTGGCTTCGGTGTAGCACCCGGATGGAGACGGCCATAAGTGA
asic1a_Frag663-825_insertion780	AAATGTTGTTCCGTCTCCCGGCACTGGTAACGGACTCGAAATCATGCTGGACATCCAACAAGATGAATACCTCCCCGTGTGGGGTGAAACAGATGAAACCAGTTTCGAAGCTGGTATAAAGGTACAAATACATAGCCAAAGCGGGAGACCGGGGTCTCTGAGCGATGAGCCCCCCTTTATTGACCAACTTGGCTTCGGTGTAGCACCCGGATGGAGACGGCCATAAGTGA
asic1a_Frag663-825_insertion783	AAATGTTGTTCCGTCTCCCGGCACTGGTAACGGACTCGAAATCATGCTGGACATCCAACAAGATGAATACCTCCCCGTGTGGGGTGAAACAGATGAAACCAGTTTCGAAGCTGGTATAAAGGTACAAATACATAGCCAAGATAGCGGGAGACCGGGGTCTCTGAGCGAGCCCCCCTTTATTGACCAACTTGGCTTCGGTGTAGCACCCGGATGGAGACGGCCATAAGTGA
asic1a_Frag663-825_insertion786	AAATGTTGTTCCGTCTCCCGGCACTGGTAACGGACTCGAAATCATGCTGGACATCCAACAAGATGAATACCTCCCCGTGTGGGGTGAAACAGATGAAACCAGTTTCGAAGCTGGTATAAAGGTACAAATACATAGCCAAGATGAGAGCGGGAGACCGGGGTCTCTGAGCCCCCCCTTTATTGACCAACTTGGCTTCGGTGTAGCACCCGGATGGAGACGGCCATAAGTGA
asic1a_Frag663-825_insertion789	AAATGTTGTTCCGTCTCCCGGCACTGGTAACGGACTCGAAATCATGCTGGACATCCAACAAGATGAATACCTCCCCGTGTGGGGTGAAACAGATGAAACCAGTTTCGAAGCTGGTATAAAGGTACAAATACATAGCCAAGATGAGCCCAGCGGGAGACCGGGGTCTCTGAGCCCCTTTATTGACCAACTTGGCTTCGGTGTAGCACCCGGATGGAGACGGCCATAAGTGA
asic1a_Frag663-825_insertion792	AAATGTTGTTCCGTCTCCCGGCACTGGTAACGGACTCGAAATCATGCTGGACATCCAACAAGATGAATACCTCCCCGTGTGGGGTGAAACAGATGAAACCAGTTTCGAAGCTGGTATAAAGGTACAAATACATAGCCAAGATGAGCCCCCCAGCGGGAGACCGGGGTCTCTGAGCTTTATTGACCAACTTGGCTTCGGTGTAGCACCCGGATGGAGACGGCCATAAGTGA
asic1a_Frag663-825_insertion795	AAATGTTGTTCCGTCTCCCGGCACTGGTAACGGACTCGAAATCATGCTGGACATCCAACAAGATGAATACCTCCCCGTGTGGGGTGAAACAGATGAAACCAGTTTCGAAGCTGGTATAAAGGTACAAATACATAGCCAAGATGAGCCCCCCTTTAGCGGGAGACCGGGGTCTCTGAGCATTGACCAACTTGGCTTCGGTGTAGCACCCGGATGGAGACGGCCATAAGTGA
asic1a_Frag663-825_insertion798	AAATGTTGTTCCGTCTCCCGGCACTGGTAACGGACTCGAAATCATGCTGGACATCCAACAAGATGAATACCTCCCCGTGTGGGGTGAAACAGATGAAACCAGTTTCGAAGCTGGTATAAAGGTACAAATACATAGCCAAGATGAGCCCCCCTTTATTAGCGGGAGACCGGGGTCTCTGAGCGACCAACTTGGCTTCGGTGTAGCACCCGGATGGAGACGGCCATAAGTGA
asic1a_Frag663-825_insertion801	AAATGTTGTTCCGTCTCCCGGCACTGGTAACGGACTCGAAATCATGCTGGACATCCAACAAGATGAATACCTCCCCGTGTGGGGTGAAACAGATGAAACCAGTTTCGAAGCTGGTATAAAGGTACAAATACATAGCCAAGATGAGCCCCCCTTTATTGACAGCGGGAGACCGGGGTCTCTGAGCCAACTTGGCTTCGGTGTAGCACCCGGATGGAGACGGCCATAAGTGA
asic1a_Frag663-825_insertion804	AAATGTTGTTCCGTCTCCCGGCACTGGTAACGGACTCGAAATCATGCTGGACATCCAACAAGATGAATACCTCCCCGTGTGGGGTGAAACAGATGAAACCAGTTTCGAAGCTGGTATAAAGGTACAAATACATAGCCAAGATGAGCCCCCCTTTATTGACCAAAGCGGGAGACCGGGGTCTCTGAGCCTTGGCTTCGGTGTAGCACCCGGATGGAGACGGCCATAAGTGA
asic1a_Frag663-825_insertion807	AAATGTTGTTCCGTCTCCCGGCACTGGTAACGGACTCGAAATCATGCTGGACATCCAACAAGATGAATACCTCCCCGTGTGGGGTGAAACAGATGAAACCAGTTTCGAAGCTGGTATAAAGGTACAAATACATAGCCAAGATGAGCCCCCCTTTATTGACCAACTTAGCGGGAGACCGGGGTCTCTGAGCGGCTTCGGTGTAGCACCCGGATGGAGACGGCCATAAGTGA
asic1a_Frag663-825_insertion810	AAATGTTGTTCCGTCTCCCGGCACTGGTAACGGACTCGAAATCATGCTGGACATCCAACAAGATGAATACCTCCCCGTGTGGGGTGAAACAGATGAAACCAGTTTCGAAGCTGGTATAAAGGTACAAATACATAGCCAAGATGAGCCCCCCTTTATTGACCAACTTGGCAGCGGGAGACCGGGGTCTCTGAGCTTCGGTGTAGCACCCGGATGGAGACGGCCATAAGTGA
asic1a_Frag663-825_insertion813	AAATGTTGTTCCGTCTCCCGGCACTGGTAACGGACTCGAAATCATGCTGGACATCCAACAAGATGAATACCTCCCCGTGTGGGGTGAAACAGATGAAACCAGTTTCGAAGCTGGTATAAAGGTACAAATACATAGCCAAGATGAGCCCCCCTTTATTGACCAACTTGGCTTCAGCGGGAGACCGGGGTCTCTGAGCGGTGTAGCACCCGGATGGAGACGGCCATAAGTGA
asic1a_Frag663-825_insertion816	AAATGTTGTTCCGTCTCCCGGCACTGGTAACGGACTCGAAATCATGCTGGACATCCAACAAGATGAATACCTCCCCGTGTGGGGTGAAACAGATGAAACCAGTTTCGAAGCTGGTATAAAGGTACAAATACATAGCCAAGATGAGCCCCCCTTTATTGACCAACTTGGCTTCGGTAGCGGGAGACCGGGGTCTCTGAGCGTAGCACCCGGATGGAGACGGCCATAAGTGA
asic1a_Frag663-825_insertion819	AAATGTTGTTCCGTCTCCCGGCACTGGTAACGGACTCGAAATCATGCTGGACATCCAACAAGATGAATACCTCCCCGTGTGGGGTGAAACAGATGAAACCAGTTTCGAAGCTGGTATAAAGGTACAAATACATAGCCAAGATGAGCCCCCCTTTATTGACCAACTTGGCTTCGGTGTAAGCGGGAGACCGGGGTCTCTGAGCGCACCCGGATGGAGACGGCCATAAGTGA
asic1a_Frag663-825_insertion822	AAATGTTGTTCCGTCTCCCGGCACTGGTAACGGACTCGAAATCATGCTGGACATCCAACAAGATGAATACCTCCCCGTGTGGGGTGAAACAGATGAAACCAGTTTCGAAGCTGGTATAAAGGTACAAATACATAGCCAAGATGAGCCCCCCTTTATTGACCAACTTGGCTTCGGTGTAGCAAGCGGGAGACCGGGGTCTCTGAGCCCCGGATGGAGACGGCCATAAGTGA
asic1a_Frag663-825_insertion825	AAATGTTGTTCCGTCTCCCGGCACTGGTAACGGACTCGAAATCATGCTGGACATCCAACAAGATGAATACCTCCCCGTGTGGGGTGAAACAGATGAAACCAGTTTCGAAGCTGGTATAAAGGTACAAATACATAGCCAAGATGAGCCCCCCTTTATTGACCAACTTGGCTTCGGTGTAGCACCCAGCGGGAGACCGGGGTCTCTGAGCGGATGGAGACGGCCATAAGTGA
asic1a_Frag828-990_insertion828	GTTACCTAATCCGTCTCCCGGAAGCGGGAGACCGGGGTCTCTGAGCTTCCAGACATTTGTGGCCTGTCAAGAACAAAGGTTGATATATCTGCCCCCTCCCTGGGGGACCTGTAAGGCCGTAACAATGGACTCCGACCTGGACTTTTTTGACTCCTACTCCATAACAGCTTGTCGAATTGACTGTGAAACTAGATATCTTGTCGAGAATTGTAGGAGACGAGTCAGTACCT
asic1a_Frag828-990_insertion831	GTTACCTAATCCGTCTCCCGGATTCAGCGGGAGACCGGGGTCTCTGAGCCAGACATTTGTGGCCTGTCAAGAACAAAGGTTGATATATCTGCCCCCTCCCTGGGGGACCTGTAAGGCCGTAACAATGGACTCCGACCTGGACTTTTTTGACTCCTACTCCATAACAGCTTGTCGAATTGACTGTGAAACTAGATATCTTGTCGAGAATTGTAGGAGACGAGTCAGTACCT
asic1a_Frag828-990_insertion834	GTTACCTAATCCGTCTCCCGGATTCCAGAGCGGGAGACCGGGGTCTCTGAGCACATTTGTGGCCTGTCAAGAACAAAGGTTGATATATCTGCCCCCTCCCTGGGGGACCTGTAAGGCCGTAACAATGGACTCCGACCTGGACTTTTTTGACTCCTACTCCATAACAGCTTGTCGAATTGACTGTGAAACTAGATATCTTGTCGAGAATTGTAGGAGACGAGTCAGTACCT
asic1a_Frag828-990_insertion837	GTTACCTAATCCGTCTCCCGGATTCCAGACAAGCGGGAGACCGGGGTCTCTGAGCTTTGTGGCCTGTCAAGAACAAAGGTTGATATATCTGCCCCCTCCCTGGGGGACCTGTAAGGCCGTAACAATGGACTCCGACCTGGACTTTTTTGACTCCTACTCCATAACAGCTTGTCGAATTGACTGTGAAACTAGATATCTTGTCGAGAATTGTAGGAGACGAGTCAGTACCT
asic1a_Frag828-990_insertion840	GTTACCTAATCCGTCTCCCGGATTCCAGACATTTAGCGGGAGACCGGGGTCTCTGAGCGTGGCCTGTCAAGAACAAAGGTTGATATATCTGCCCCCTCCCTGGGGGACCTGTAAGGCCGTAACAATGGACTCCGACCTGGACTTTTTTGACTCCTACTCCATAACAGCTTGTCGAATTGACTGTGAAACTAGATATCTTGTCGAGAATTGTAGGAGACGAGTCAGTACCT
asic1a_Frag828-990_insertion843	GTTACCTAATCCGTCTCCCGGATTCCAGACATTTGTGAGCGGGAGACCGGGGTCTCTGAGCGCCTGTCAAGAACAAAGGTTGATATATCTGCCCCCTCCCTGGGGGACCTGTAAGGCCGTAACAATGGACTCCGACCTGGACTTTTTTGACTCCTACTCCATAACAGCTTGTCGAATTGACTGTGAAACTAGATATCTTGTCGAGAATTGTAGGAGACGAGTCAGTACCT
asic1a_Frag828-990_insertion846	GTTACCTAATCCGTCTCCCGGATTCCAGACATTTGTGGCCAGCGGGAGACCGGGGTCTCTGAGCTGTCAAGAACAAAGGTTGATATATCTGCCCCCTCCCTGGGGGACCTGTAAGGCCGTAACAATGGACTCCGACCTGGACTTTTTTGACTCCTACTCCATAACAGCTTGTCGAATTGACTGTGAAACTAGATATCTTGTCGAGAATTGTAGGAGACGAGTCAGTACCT
asic1a_Frag828-990_insertion849	GTTACCTAATCCGTCTCCCGGATTCCAGACATTTGTGGCCTGTAGCGGGAGACCGGGGTCTCTGAGCCAAGAACAAAGGTTGATATATCTGCCCCCTCCCTGGGGGACCTGTAAGGCCGTAACAATGGACTCCGACCTGGACTTTTTTGACTCCTACTCCATAACAGCTTGTCGAATTGACTGTGAAACTAGATATCTTGTCGAGAATTGTAGGAGACGAGTCAGTACCT
asic1a_Frag828-990_insertion852	GTTACCTAATCCGTCTCCCGGATTCCAGACATTTGTGGCCTGTCAAAGCGGGAGACCGGGGTCTCTGAGCGAACAAAGGTTGATATATCTGCCCCCTCCCTGGGGGACCTGTAAGGCCGTAACAATGGACTCCGACCTGGACTTTTTTGACTCCTACTCCATAACAGCTTGTCGAATTGACTGTGAAACTAGATATCTTGTCGAGAATTGTAGGAGACGAGTCAGTACCT
asic1a_Frag828-990_insertion855	GTTACCTAATCCGTCTCCCGGATTCCAGACATTTGTGGCCTGTCAAGAAAGCGGGAGACCGGGGTCTCTGAGCCAAAGGTTGATATATCTGCCCCCTCCCTGGGGGACCTGTAAGGCCGTAACAATGGACTCCGACCTGGACTTTTTTGACTCCTACTCCATAACAGCTTGTCGAATTGACTGTGAAACTAGATATCTTGTCGAGAATTGTAGGAGACGAGTCAGTACCT
asic1a_Frag828-990_insertion858	GTTACCTAATCCGTCTCCCGGATTCCAGACATTTGTGGCCTGTCAAGAACAAAGCGGGAGACCGGGGTCTCTGAGCAGGTTGATATATCTGCCCCCTCCCTGGGGGACCTGTAAGGCCGTAACAATGGACTCCGACCTGGACTTTTTTGACTCCTACTCCATAACAGCTTGTCGAATTGACTGTGAAACTAGATATCTTGTCGAGAATTGTAGGAGACGAGTCAGTACCT
asic1a_Frag828-990_insertion861	GTTACCTAATCCGTCTCCCGGATTCCAGACATTTGTGGCCTGTCAAGAACAAAGGAGCGGGAGACCGGGGTCTCTGAGCTTGATATATCTGCCCCCTCCCTGGGGGACCTGTAAGGCCGTAACAATGGACTCCGACCTGGACTTTTTTGACTCCTACTCCATAACAGCTTGTCGAATTGACTGTGAAACTAGATATCTTGTCGAGAATTGTAGGAGACGAGTCAGTACCT
asic1a_Frag828-990_insertion864	GTTACCTAATCCGTCTCCCGGATTCCAGACATTTGTGGCCTGTCAAGAACAAAGGTTGAGCGGGAGACCGGGGTCTCTGAGCATATATCTGCCCCCTCCCTGGGGGACCTGTAAGGCCGTAACAATGGACTCCGACCTGGACTTTTTTGACTCCTACTCCATAACAGCTTGTCGAATTGACTGTGAAACTAGATATCTTGTCGAGAATTGTAGGAGACGAGTCAGTACCT
asic1a_Frag828-990_insertion867	GTTACCTAATCCGTCTCCCGGATTCCAGACATTTGTGGCCTGTCAAGAACAAAGGTTGATAAGCGGGAGACCGGGGTCTCTGAGCTATCTGCCCCCTCCCTGGGGGACCTGTAAGGCCGTAACAATGGACTCCGACCTGGACTTTTTTGACTCCTACTCCATAACAGCTTGTCGAATTGACTGTGAAACTAGATATCTTGTCGAGAATTGTAGGAGACGAGTCAGTACCT
asic1a_Frag828-990_insertion870	GTTACCTAATCCGTCTCCCGGATTCCAGACATTTGTGGCCTGTCAAGAACAAAGGTTGATATATAGCGGGAGACCGGGGTCTCTGAGCCTGCCCCCTCCCTGGGGGACCTGTAAGGCCGTAACAATGGACTCCGACCTGGACTTTTTTGACTCCTACTCCATAACAGCTTGTCGAATTGACTGTGAAACTAGATATCTTGTCGAGAATTGTAGGAGACGAGTCAGTACCT
asic1a_Frag828-990_insertion873	GTTACCTAATCCGTCTCCCGGATTCCAGACATTTGTGGCCTGTCAAGAACAAAGGTTGATATATCTGAGCGGGAGACCGGGGTCTCTGAGCCCCCCTCCCTGGGGGACCTGTAAGGCCGTAACAATGGACTCCGACCTGGACTTTTTTGACTCCTACTCCATAACAGCTTGTCGAATTGACTGTGAAACTAGATATCTTGTCGAGAATTGTAGGAGACGAGTCAGTACCT
asic1a_Frag828-990_insertion876	GTTACCTAATCCGTCTCCCGGATTCCAGACATTTGTGGCCTGTCAAGAACAAAGGTTGATATATCTGCCCAGCGGGAGACCGGGGTCTCTGAGCCCTCCCTGGGGGACCTGTAAGGCCGTAACAATGGACTCCGACCTGGACTTTTTTGACTCCTACTCCATAACAGCTTGTCGAATTGACTGTGAAACTAGATATCTTGTCGAGAATTGTAGGAGACGAGTCAGTACCT
asic1a_Frag828-990_insertion879	GTTACCTAATCCGTCTCCCGGATTCCAGACATTTGTGGCCTGTCAAGAACAAAGGTTGATATATCTGCCCCCTAGCGGGAGACCGGGGTCTCTGAGCCCCTGGGGGACCTGTAAGGCCGTAACAATGGACTCCGACCTGGACTTTTTTGACTCCTACTCCATAACAGCTTGTCGAATTGACTGTGAAACTAGATATCTTGTCGAGAATTGTAGGAGACGAGTCAGTACCT
asic1a_Frag828-990_insertion882	GTTACCTAATCCGTCTCCCGGATTCCAGACATTTGTGGCCTGTCAAGAACAAAGGTTGATATATCTGCCCCCTCCCAGCGGGAGACCGGGGTCTCTGAGCTGGGGGACCTGTAAGGCCGTAACAATGGACTCCGACCTGGACTTTTTTGACTCCTACTCCATAACAGCTTGTCGAATTGACTGTGAAACTAGATATCTTGTCGAGAATTGTAGGAGACGAGTCAGTACCT
asic1a_Frag828-990_insertion885	GTTACCTAATCCGTCTCCCGGATTCCAGACATTTGTGGCCTGTCAAGAACAAAGGTTGATATATCTGCCCCCTCCCTGGAGCGGGAGACCGGGGTCTCTGAGCGGGACCTGTAAGGCCGTAACAATGGACTCCGACCTGGACTTTTTTGACTCCTACTCCATAACAGCTTGTCGAATTGACTGTGAAACTAGATATCTTGTCGAGAATTGTAGGAGACGAGTCAGTACCT
asic1a_Frag828-990_insertion888	GTTACCTAATCCGTCTCCCGGATTCCAGACATTTGTGGCCTGTCAAGAACAAAGGTTGATATATCTGCCCCCTCCCTGGGGGAGCGGGAGACCGGGGTCTCTGAGCACCTGTAAGGCCGTAACAATGGACTCCGACCTGGACTTTTTTGACTCCTACTCCATAACAGCTTGTCGAATTGACTGTGAAACTAGATATCTTGTCGAGAATTGTAGGAGACGAGTCAGTACCT
asic1a_Frag828-990_insertion891	GTTACCTAATCCGTCTCCCGGATTCCAGACATTTGTGGCCTGTCAAGAACAAAGGTTGATATATCTGCCCCCTCCCTGGGGGACCAGCGGGAGACCGGGGTCTCTGAGCTGTAAGGCCGTAACAATGGACTCCGACCTGGACTTTTTTGACTCCTACTCCATAACAGCTTGTCGAATTGACTGTGAAACTAGATATCTTGTCGAGAATTGTAGGAGACGAGTCAGTACCT
asic1a_Frag828-990_insertion894	GTTACCTAATCCGTCTCCCGGATTCCAGACATTTGTGGCCTGTCAAGAACAAAGGTTGATATATCTGCCCCCTCCCTGGGGGACCTGTAGCGGGAGACCGGGGTCTCTGAGCAAGGCCGTAACAATGGACTCCGACCTGGACTTTTTTGACTCCTACTCCATAACAGCTTGTCGAATTGACTGTGAAACTAGATATCTTGTCGAGAATTGTAGGAGACGAGTCAGTACCT
asic1a_Frag828-990_insertion897	GTTACCTAATCCGTCTCCCGGATTCCAGACATTTGTGGCCTGTCAAGAACAAAGGTTGATATATCTGCCCCCTCCCTGGGGGACCTGTAAGAGCGGGAGACCGGGGTCTCTGAGCGCCGTAACAATGGACTCCGACCTGGACTTTTTTGACTCCTACTCCATAACAGCTTGTCGAATTGACTGTGAAACTAGATATCTTGTCGAGAATTGTAGGAGACGAGTCAGTACCT
asic1a_Frag828-990_insertion900	GTTACCTAATCCGTCTCCCGGATTCCAGACATTTGTGGCCTGTCAAGAACAAAGGTTGATATATCTGCCCCCTCCCTGGGGGACCTGTAAGGCCAGCGGGAGACCGGGGTCTCTGAGCGTAACAATGGACTCCGACCTGGACTTTTTTGACTCCTACTCCATAACAGCTTGTCGAATTGACTGTGAAACTAGATATCTTGTCGAGAATTGTAGGAGACGAGTCAGTACCT
asic1a_Frag828-990_insertion903	GTTACCTAATCCGTCTCCCGGATTCCAGACATTTGTGGCCTGTCAAGAACAAAGGTTGATATATCTGCCCCCTCCCTGGGGGACCTGTAAGGCCGTAAGCGGGAGACCGGGGTCTCTGAGCACAATGGACTCCGACCTGGACTTTTTTGACTCCTACTCCATAACAGCTTGTCGAATTGACTGTGAAACTAGATATCTTGTCGAGAATTGTAGGAGACGAGTCAGTACCT
asic1a_Frag828-990_insertion906	GTTACCTAATCCGTCTCCCGGATTCCAGACATTTGTGGCCTGTCAAGAACAAAGGTTGATATATCTGCCCCCTCCCTGGGGGACCTGTAAGGCCGTAACAAGCGGGAGACCGGGGTCTCTGAGCATGGACTCCGACCTGGACTTTTTTGACTCCTACTCCATAACAGCTTGTCGAATTGACTGTGAAACTAGATATCTTGTCGAGAATTGTAGGAGACGAGTCAGTACCT
asic1a_Frag828-990_insertion909	GTTACCTAATCCGTCTCCCGGATTCCAGACATTTGTGGCCTGTCAAGAACAAAGGTTGATATATCTGCCCCCTCCCTGGGGGACCTGTAAGGCCGTAACAATGAGCGGGAGACCGGGGTCTCTGAGCGACTCCGACCTGGACTTTTTTGACTCCTACTCCATAACAGCTTGTCGAATTGACTGTGAAACTAGATATCTTGTCGAGAATTGTAGGAGACGAGTCAGTACCT
asic1a_Frag828-990_insertion912	GTTACCTAATCCGTCTCCCGGATTCCAGACATTTGTGGCCTGTCAAGAACAAAGGTTGATATATCTGCCCCCTCCCTGGGGGACCTGTAAGGCCGTAACAATGGACAGCGGGAGACCGGGGTCTCTGAGCTCCGACCTGGACTTTTTTGACTCCTACTCCATAACAGCTTGTCGAATTGACTGTGAAACTAGATATCTTGTCGAGAATTGTAGGAGACGAGTCAGTACCT
asic1a_Frag828-990_insertion915	GTTACCTAATCCGTCTCCCGGATTCCAGACATTTGTGGCCTGTCAAGAACAAAGGTTGATATATCTGCCCCCTCCCTGGGGGACCTGTAAGGCCGTAACAATGGACTCCAGCGGGAGACCGGGGTCTCTGAGCGACCTGGACTTTTTTGACTCCTACTCCATAACAGCTTGTCGAATTGACTGTGAAACTAGATATCTTGTCGAGAATTGTAGGAGACGAGTCAGTACCT
asic1a_Frag828-990_insertion918	GTTACCTAATCCGTCTCCCGGATTCCAGACATTTGTGGCCTGTCAAGAACAAAGGTTGATATATCTGCCCCCTCCCTGGGGGACCTGTAAGGCCGTAACAATGGACTCCGACAGCGGGAGACCGGGGTCTCTGAGCCTGGACTTTTTTGACTCCTACTCCATAACAGCTTGTCGAATTGACTGTGAAACTAGATATCTTGTCGAGAATTGTAGGAGACGAGTCAGTACCT
asic1a_Frag828-990_insertion921	GTTACCTAATCCGTCTCCCGGATTCCAGACATTTGTGGCCTGTCAAGAACAAAGGTTGATATATCTGCCCCCTCCCTGGGGGACCTGTAAGGCCGTAACAATGGACTCCGACCTGAGCGGGAGACCGGGGTCTCTGAGCGACTTTTTTGACTCCTACTCCATAACAGCTTGTCGAATTGACTGTGAAACTAGATATCTTGTCGAGAATTGTAGGAGACGAGTCAGTACCT
asic1a_Frag828-990_insertion924	GTTACCTAATCCGTCTCCCGGATTCCAGACATTTGTGGCCTGTCAAGAACAAAGGTTGATATATCTGCCCCCTCCCTGGGGGACCTGTAAGGCCGTAACAATGGACTCCGACCTGGACAGCGGGAGACCGGGGTCTCTGAGCTTTTTTGACTCCTACTCCATAACAGCTTGTCGAATTGACTGTGAAACTAGATATCTTGTCGAGAATTGTAGGAGACGAGTCAGTACCT
asic1a_Frag828-990_insertion927	GTTACCTAATCCGTCTCCCGGATTCCAGACATTTGTGGCCTGTCAAGAACAAAGGTTGATATATCTGCCCCCTCCCTGGGGGACCTGTAAGGCCGTAACAATGGACTCCGACCTGGACTTTAGCGGGAGACCGGGGTCTCTGAGCTTTGACTCCTACTCCATAACAGCTTGTCGAATTGACTGTGAAACTAGATATCTTGTCGAGAATTGTAGGAGACGAGTCAGTACCT
asic1a_Frag828-990_insertion930	GTTACCTAATCCGTCTCCCGGATTCCAGACATTTGTGGCCTGTCAAGAACAAAGGTTGATATATCTGCCCCCTCCCTGGGGGACCTGTAAGGCCGTAACAATGGACTCCGACCTGGACTTTTTTAGCGGGAGACCGGGGTCTCTGAGCGACTCCTACTCCATAACAGCTTGTCGAATTGACTGTGAAACTAGATATCTTGTCGAGAATTGTAGGAGACGAGTCAGTACCT
asic1a_Frag828-990_insertion933	GTTACCTAATCCGTCTCCCGGATTCCAGACATTTGTGGCCTGTCAAGAACAAAGGTTGATATATCTGCCCCCTCCCTGGGGGACCTGTAAGGCCGTAACAATGGACTCCGACCTGGACTTTTTTGACAGCGGGAGACCGGGGTCTCTGAGCTCCTACTCCATAACAGCTTGTCGAATTGACTGTGAAACTAGATATCTTGTCGAGAATTGTAGGAGACGAGTCAGTACCT
asic1a_Frag828-990_insertion936	GTTACCTAATCCGTCTCCCGGATTCCAGACATTTGTGGCCTGTCAAGAACAAAGGTTGATATATCTGCCCCCTCCCTGGGGGACCTGTAAGGCCGTAACAATGGACTCCGACCTGGACTTTTTTGACTCCAGCGGGAGACCGGGGTCTCTGAGCTACTCCATAACAGCTTGTCGAATTGACTGTGAAACTAGATATCTTGTCGAGAATTGTAGGAGACGAGTCAGTACCT
asic1a_Frag828-990_insertion939	GTTACCTAATCCGTCTCCCGGATTCCAGACATTTGTGGCCTGTCAAGAACAAAGGTTGATATATCTGCCCCCTCCCTGGGGGACCTGTAAGGCCGTAACAATGGACTCCGACCTGGACTTTTTTGACTCCTACAGCGGGAGACCGGGGTCTCTGAGCTCCATAACAGCTTGTCGAATTGACTGTGAAACTAGATATCTTGTCGAGAATTGTAGGAGACGAGTCAGTACCT
asic1a_Frag828-990_insertion942	GTTACCTAATCCGTCTCCCGGATTCCAGACATTTGTGGCCTGTCAAGAACAAAGGTTGATATATCTGCCCCCTCCCTGGGGGACCTGTAAGGCCGTAACAATGGACTCCGACCTGGACTTTTTTGACTCCTACTCCAGCGGGAGACCGGGGTCTCTGAGCATAACAGCTTGTCGAATTGACTGTGAAACTAGATATCTTGTCGAGAATTGTAGGAGACGAGTCAGTACCT
asic1a_Frag828-990_insertion945	GTTACCTAATCCGTCTCCCGGATTCCAGACATTTGTGGCCTGTCAAGAACAAAGGTTGATATATCTGCCCCCTCCCTGGGGGACCTGTAAGGCCGTAACAATGGACTCCGACCTGGACTTTTTTGACTCCTACTCCATAAGCGGGAGACCGGGGTCTCTGAGCACAGCTTGTCGAATTGACTGTGAAACTAGATATCTTGTCGAGAATTGTAGGAGACGAGTCAGTACCT
asic1a_Frag828-990_insertion948	GTTACCTAATCCGTCTCCCGGATTCCAGACATTTGTGGCCTGTCAAGAACAAAGGTTGATATATCTGCCCCCTCCCTGGGGGACCTGTAAGGCCGTAACAATGGACTCCGACCTGGACTTTTTTGACTCCTACTCCATAACAAGCGGGAGACCGGGGTCTCTGAGCGCTTGTCGAATTGACTGTGAAACTAGATATCTTGTCGAGAATTGTAGGAGACGAGTCAGTACCT
asic1a_Frag828-990_insertion951	GTTACCTAATCCGTCTCCCGGATTCCAGACATTTGTGGCCTGTCAAGAACAAAGGTTGATATATCTGCCCCCTCCCTGGGGGACCTGTAAGGCCGTAACAATGGACTCCGACCTGGACTTTTTTGACTCCTACTCCATAACAGCTAGCGGGAGACCGGGGTCTCTGAGCTGTCGAATTGACTGTGAAACTAGATATCTTGTCGAGAATTGTAGGAGACGAGTCAGTACCT
asic1a_Frag828-990_insertion954	GTTACCTAATCCGTCTCCCGGATTCCAGACATTTGTGGCCTGTCAAGAACAAAGGTTGATATATCTGCCCCCTCCCTGGGGGACCTGTAAGGCCGTAACAATGGACTCCGACCTGGACTTTTTTGACTCCTACTCCATAACAGCTTGTAGCGGGAGACCGGGGTCTCTGAGCCGAATTGACTGTGAAACTAGATATCTTGTCGAGAATTGTAGGAGACGAGTCAGTACCT
asic1a_Frag828-990_insertion957	GTTACCTAATCCGTCTCCCGGATTCCAGACATTTGTGGCCTGTCAAGAACAAAGGTTGATATATCTGCCCCCTCCCTGGGGGACCTGTAAGGCCGTAACAATGGACTCCGACCTGGACTTTTTTGACTCCTACTCCATAACAGCTTGTCGAAGCGGGAGACCGGGGTCTCTGAGCATTGACTGTGAAACTAGATATCTTGTCGAGAATTGTAGGAGACGAGTCAGTACCT
asic1a_Frag828-990_insertion960	GTTACCTAATCCGTCTCCCGGATTCCAGACATTTGTGGCCTGTCAAGAACAAAGGTTGATATATCTGCCCCCTCCCTGGGGGACCTGTAAGGCCGTAACAATGGACTCCGACCTGGACTTTTTTGACTCCTACTCCATAACAGCTTGTCGAATTAGCGGGAGACCGGGGTCTCTGAGCGACTGTGAAACTAGATATCTTGTCGAGAATTGTAGGAGACGAGTCAGTACCT
asic1a_Frag828-990_insertion963	GTTACCTAATCCGTCTCCCGGATTCCAGACATTTGTGGCCTGTCAAGAACAAAGGTTGATATATCTGCCCCCTCCCTGGGGGACCTGTAAGGCCGTAACAATGGACTCCGACCTGGACTTTTTTGACTCCTACTCCATAACAGCTTGTCGAATTGACAGCGGGAGACCGGGGTCTCTGAGCTGTGAAACTAGATATCTTGTCGAGAATTGTAGGAGACGAGTCAGTACCT
asic1a_Frag828-990_insertion966	GTTACCTAATCCGTCTCCCGGATTCCAGACATTTGTGGCCTGTCAAGAACAAAGGTTGATATATCTGCCCCCTCCCTGGGGGACCTGTAAGGCCGTAACAATGGACTCCGACCTGGACTTTTTTGACTCCTACTCCATAACAGCTTGTCGAATTGACTGTAGCGGGAGACCGGGGTCTCTGAGCGAAACTAGATATCTTGTCGAGAATTGTAGGAGACGAGTCAGTACCT
asic1a_Frag828-990_insertion969	GTTACCTAATCCGTCTCCCGGATTCCAGACATTTGTGGCCTGTCAAGAACAAAGGTTGATATATCTGCCCCCTCCCTGGGGGACCTGTAAGGCCGTAACAATGGACTCCGACCTGGACTTTTTTGACTCCTACTCCATAACAGCTTGTCGAATTGACTGTGAAAGCGGGAGACCGGGGTCTCTGAGCACTAGATATCTTGTCGAGAATTGTAGGAGACGAGTCAGTACCT
asic1a_Frag828-990_insertion972	GTTACCTAATCCGTCTCCCGGATTCCAGACATTTGTGGCCTGTCAAGAACAAAGGTTGATATATCTGCCCCCTCCCTGGGGGACCTGTAAGGCCGTAACAATGGACTCCGACCTGGACTTTTTTGACTCCTACTCCATAACAGCTTGTCGAATTGACTGTGAAACTAGCGGGAGACCGGGGTCTCTGAGCAGATATCTTGTCGAGAATTGTAGGAGACGAGTCAGTACCT
asic1a_Frag828-990_insertion975	GTTACCTAATCCGTCTCCCGGATTCCAGACATTTGTGGCCTGTCAAGAACAAAGGTTGATATATCTGCCCCCTCCCTGGGGGACCTGTAAGGCCGTAACAATGGACTCCGACCTGGACTTTTTTGACTCCTACTCCATAACAGCTTGTCGAATTGACTGTGAAACTAGAAGCGGGAGACCGGGGTCTCTGAGCTATCTTGTCGAGAATTGTAGGAGACGAGTCAGTACCT
asic1a_Frag828-990_insertion978	GTTACCTAATCCGTCTCCCGGATTCCAGACATTTGTGGCCTGTCAAGAACAAAGGTTGATATATCTGCCCCCTCCCTGGGGGACCTGTAAGGCCGTAACAATGGACTCCGACCTGGACTTTTTTGACTCCTACTCCATAACAGCTTGTCGAATTGACTGTGAAACTAGATATAGCGGGAGACCGGGGTCTCTGAGCCTTGTCGAGAATTGTAGGAGACGAGTCAGTACCT
asic1a_Frag828-990_insertion981	GTTACCTAATCCGTCTCCCGGATTCCAGACATTTGTGGCCTGTCAAGAACAAAGGTTGATATATCTGCCCCCTCCCTGGGGGACCTGTAAGGCCGTAACAATGGACTCCGACCTGGACTTTTTTGACTCCTACTCCATAACAGCTTGTCGAATTGACTGTGAAACTAGATATCTTAGCGGGAGACCGGGGTCTCTGAGCGTCGAGAATTGTAGGAGACGAGTCAGTACCT
asic1a_Frag828-990_insertion984	GTTACCTAATCCGTCTCCCGGATTCCAGACATTTGTGGCCTGTCAAGAACAAAGGTTGATATATCTGCCCCCTCCCTGGGGGACCTGTAAGGCCGTAACAATGGACTCCGACCTGGACTTTTTTGACTCCTACTCCATAACAGCTTGTCGAATTGACTGTGAAACTAGATATCTTGTCAGCGGGAGACCGGGGTCTCTGAGCGAGAATTGTAGGAGACGAGTCAGTACCT

asic1a_Frag828-990_insertion987	GTTACCTAATCCGTCTCCCGGATTCCAGACATTTGTGGCCTGTCAAGAACAAAGGTTGATATATCTGCCCCCTCCCTGGGGGACCTGTAAGGCCGTAACAATGGACTCCGACCTGGACTTTTTTGACTCCTACTCCATAACAGCTTGTCGAATTGACTGTGAAACTAGATATCTTGTCGAGAGCGGGAGACCGGGGTCTCTGAGCAATTGTAGGAGACGAGTCAGTACCT
asic1a_Frag828-990_insertion990	GTTACCTAATCCGTCTCCCGGATTCCAGACATTTGTGGCCTGTCAAGAACAAAGGTTGATATATCTGCCCCCTCCCTGGGGGACCTGTAAGGCCGTAACAATGGACTCCGACCTGGACTTTTTTGACTCCTACTCCATAACAGCTTGTCGAATTGACTGTGAAACTAGATATCTTGTCGAGAATAGCGGGAGACCGGGGTCTCTGAGCTGTAGGAGACGAGTCAGTACCT
asic1a_Frag993-1152_insertion993	GATACTTAACGTCGTCTCCTTGTAGCGGGAGACCGGGGTCTCTGAGCAACTGTAGGATGGTTCACATGCCCGGAGATGCCCCATACTGCACTCCCGAACAATACAAGGAGTGTGCCGACCCTGCACTTGACTTTCTCGTTGAAAAAGATCAGGAGTATTGCGTGTGCGAGATGCCCTGTAATCTTACACGGTACGGTAAGGAACTTAGTAGGAGACGGGAGAACCTATCC
asic1a_Frag993-1152_insertion996	GATACTTAACGTCGTCTCCTTGTAACAGCGGGAGACCGGGGTCTCTGAGCTGTAGGATGGTTCACATGCCCGGAGATGCCCCATACTGCACTCCCGAACAATACAAGGAGTGTGCCGACCCTGCACTTGACTTTCTCGTTGAAAAAGATCAGGAGTATTGCGTGTGCGAGATGCCCTGTAATCTTACACGGTACGGTAAGGAACTTAGTAGGAGACGGGAGAACCTATCC
asic1a_Frag993-1152_insertion999	GATACTTAACGTCGTCTCCTTGTAACTGTAGCGGGAGACCGGGGTCTCTGAGCAGGATGGTTCACATGCCCGGAGATGCCCCATACTGCACTCCCGAACAATACAAGGAGTGTGCCGACCCTGCACTTGACTTTCTCGTTGAAAAAGATCAGGAGTATTGCGTGTGCGAGATGCCCTGTAATCTTACACGGTACGGTAAGGAACTTAGTAGGAGACGGGAGAACCTATCC
asic1a_Frag993-1152_insertion1002	GATACTTAACGTCGTCTCCTTGTAACTGTAGGAGCGGGAGACCGGGGTCTCTGAGCATGGTTCACATGCCCGGAGATGCCCCATACTGCACTCCCGAACAATACAAGGAGTGTGCCGACCCTGCACTTGACTTTCTCGTTGAAAAAGATCAGGAGTATTGCGTGTGCGAGATGCCCTGTAATCTTACACGGTACGGTAAGGAACTTAGTAGGAGACGGGAGAACCTATCC
asic1a_Frag993-1152_insertion1005	GATACTTAACGTCGTCTCCTTGTAACTGTAGGATGAGCGGGAGACCGGGGTCTCTGAGCGTTCACATGCCCGGAGATGCCCCATACTGCACTCCCGAACAATACAAGGAGTGTGCCGACCCTGCACTTGACTTTCTCGTTGAAAAAGATCAGGAGTATTGCGTGTGCGAGATGCCCTGTAATCTTACACGGTACGGTAAGGAACTTAGTAGGAGACGGGAGAACCTATCC
asic1a_Frag993-1152_insertion1008	GATACTTAACGTCGTCTCCTTGTAACTGTAGGATGGTTAGCGGGAGACCGGGGTCTCTGAGCCACATGCCCGGAGATGCCCCATACTGCACTCCCGAACAATACAAGGAGTGTGCCGACCCTGCACTTGACTTTCTCGTTGAAAAAGATCAGGAGTATTGCGTGTGCGAGATGCCCTGTAATCTTACACGGTACGGTAAGGAACTTAGTAGGAGACGGGAGAACCTATCC
asic1a_Frag993-1152_insertion1011	GATACTTAACGTCGTCTCCTTGTAACTGTAGGATGGTTCACAGCGGGAGACCGGGGTCTCTGAGCATGCCCGGAGATGCCCCATACTGCACTCCCGAACAATACAAGGAGTGTGCCGACCCTGCACTTGACTTTCTCGTTGAAAAAGATCAGGAGTATTGCGTGTGCGAGATGCCCTGTAATCTTACACGGTACGGTAAGGAACTTAGTAGGAGACGGGAGAACCTATCC
asic1a_Frag993-1152_insertion1014	GATACTTAACGTCGTCTCCTTGTAACTGTAGGATGGTTCACATGAGCGGGAGACCGGGGTCTCTGAGCCCCGGAGATGCCCCATACTGCACTCCCGAACAATACAAGGAGTGTGCCGACCCTGCACTTGACTTTCTCGTTGAAAAAGATCAGGAGTATTGCGTGTGCGAGATGCCCTGTAATCTTACACGGTACGGTAAGGAACTTAGTAGGAGACGGGAGAACCTATCC
asic1a_Frag993-1152_insertion1017	GATACTTAACGTCGTCTCCTTGTAACTGTAGGATGGTTCACATGCCCAGCGGGAGACCGGGGTCTCTGAGCGGAGATGCCCCATACTGCACTCCCGAACAATACAAGGAGTGTGCCGACCCTGCACTTGACTTTCTCGTTGAAAAAGATCAGGAGTATTGCGTGTGCGAGATGCCCTGTAATCTTACACGGTACGGTAAGGAACTTAGTAGGAGACGGGAGAACCTATCC
asic1a_Frag993-1152_insertion1020	GATACTTAACGTCGTCTCCTTGTAACTGTAGGATGGTTCACATGCCCGGAAGCGGGAGACCGGGGTCTCTGAGCGATGCCCCATACTGCACTCCCGAACAATACAAGGAGTGTGCCGACCCTGCACTTGACTTTCTCGTTGAAAAAGATCAGGAGTATTGCGTGTGCGAGATGCCCTGTAATCTTACACGGTACGGTAAGGAACTTAGTAGGAGACGGGAGAACCTATCC
asic1a_Frag993-1152_insertion1023	GATACTTAACGTCGTCTCCTTGTAACTGTAGGATGGTTCACATGCCCGGAGATAGCGGGAGACCGGGGTCTCTGAGCGCCCCATACTGCACTCCCGAACAATACAAGGAGTGTGCCGACCCTGCACTTGACTTTCTCGTTGAAAAAGATCAGGAGTATTGCGTGTGCGAGATGCCCTGTAATCTTACACGGTACGGTAAGGAACTTAGTAGGAGACGGGAGAACCTATCC
asic1a_Frag993-1152_insertion1026	GATACTTAACGTCGTCTCCTTGTAACTGTAGGATGGTTCACATGCCCGGAGATGCCAGCGGGAGACCGGGGTCTCTGAGCCCATACTGCACTCCCGAACAATACAAGGAGTGTGCCGACCCTGCACTTGACTTTCTCGTTGAAAAAGATCAGGAGTATTGCGTGTGCGAGATGCCCTGTAATCTTACACGGTACGGTAAGGAACTTAGTAGGAGACGGGAGAACCTATCC
asic1a_Frag993-1152_insertion1029	GATACTTAACGTCGTCTCCTTGTAACTGTAGGATGGTTCACATGCCCGGAGATGCCCCAAGCGGGAGACCGGGGTCTCTGAGCTACTGCACTCCCGAACAATACAAGGAGTGTGCCGACCCTGCACTTGACTTTCTCGTTGAAAAAGATCAGGAGTATTGCGTGTGCGAGATGCCCTGTAATCTTACACGGTACGGTAAGGAACTTAGTAGGAGACGGGAGAACCTATCC
asic1a_Frag993-1152_insertion1032	GATACTTAACGTCGTCTCCTTGTAACTGTAGGATGGTTCACATGCCCGGAGATGCCCCATACAGCGGGAGACCGGGGTCTCTGAGCTGCACTCCCGAACAATACAAGGAGTGTGCCGACCCTGCACTTGACTTTCTCGTTGAAAAAGATCAGGAGTATTGCGTGTGCGAGATGCCCTGTAATCTTACACGGTACGGTAAGGAACTTAGTAGGAGACGGGAGAACCTATCC
asic1a_Frag993-1152_insertion1035	GATACTTAACGTCGTCTCCTTGTAACTGTAGGATGGTTCACATGCCCGGAGATGCCCCATACTGCAGCGGGAGACCGGGGTCTCTGAGCACTCCCGAACAATACAAGGAGTGTGCCGACCCTGCACTTGACTTTCTCGTTGAAAAAGATCAGGAGTATTGCGTGTGCGAGATGCCCTGTAATCTTACACGGTACGGTAAGGAACTTAGTAGGAGACGGGAGAACCTATCC
asic1a_Frag993-1152_insertion1038	GATACTTAACGTCGTCTCCTTGTAACTGTAGGATGGTTCACATGCCCGGAGATGCCCCATACTGCACTAGCGGGAGACCGGGGTCTCTGAGCCCCGAACAATACAAGGAGTGTGCCGACCCTGCACTTGACTTTCTCGTTGAAAAAGATCAGGAGTATTGCGTGTGCGAGATGCCCTGTAATCTTACACGGTACGGTAAGGAACTTAGTAGGAGACGGGAGAACCTATCC
asic1a_Frag993-1152_insertion1041	GATACTTAACGTCGTCTCCTTGTAACTGTAGGATGGTTCACATGCCCGGAGATGCCCCATACTGCACTCCCAGCGGGAGACCGGGGTCTCTGAGCGAACAATACAAGGAGTGTGCCGACCCTGCACTTGACTTTCTCGTTGAAAAAGATCAGGAGTATTGCGTGTGCGAGATGCCCTGTAATCTTACACGGTACGGTAAGGAACTTAGTAGGAGACGGGAGAACCTATCC
asic1a_Frag993-1152_insertion1044	GATACTTAACGTCGTCTCCTTGTAACTGTAGGATGGTTCACATGCCCGGAGATGCCCCATACTGCACTCCCGAAAGCGGGAGACCGGGGTCTCTGAGCCAATACAAGGAGTGTGCCGACCCTGCACTTGACTTTCTCGTTGAAAAAGATCAGGAGTATTGCGTGTGCGAGATGCCCTGTAATCTTACACGGTACGGTAAGGAACTTAGTAGGAGACGGGAGAACCTATCC
asic1a_Frag993-1152_insertion1047	GATACTTAACGTCGTCTCCTTGTAACTGTAGGATGGTTCACATGCCCGGAGATGCCCCATACTGCACTCCCGAACAAAGCGGGAGACCGGGGTCTCTGAGCTACAAGGAGTGTGCCGACCCTGCACTTGACTTTCTCGTTGAAAAAGATCAGGAGTATTGCGTGTGCGAGATGCCCTGTAATCTTACACGGTACGGTAAGGAACTTAGTAGGAGACGGGAGAACCTATCC
asic1a_Frag993-1152_insertion1050	GATACTTAACGTCGTCTCCTTGTAACTGTAGGATGGTTCACATGCCCGGAGATGCCCCATACTGCACTCCCGAACAATACAGCGGGAGACCGGGGTCTCTGAGCAAGGAGTGTGCCGACCCTGCACTTGACTTTCTCGTTGAAAAAGATCAGGAGTATTGCGTGTGCGAGATGCCCTGTAATCTTACACGGTACGGTAAGGAACTTAGTAGGAGACGGGAGAACCTATCC
asic1a_Frag993-1152_insertion1053	GATACTTAACGTCGTCTCCTTGTAACTGTAGGATGGTTCACATGCCCGGAGATGCCCCATACTGCACTCCCGAACAATACAAGAGCGGGAGACCGGGGTCTCTGAGCGAGTGTGCCGACCCTGCACTTGACTTTCTCGTTGAAAAAGATCAGGAGTATTGCGTGTGCGAGATGCCCTGTAATCTTACACGGTACGGTAAGGAACTTAGTAGGAGACGGGAGAACCTATCC
asic1a_Frag993-1152_insertion1056	GATACTTAACGTCGTCTCCTTGTAACTGTAGGATGGTTCACATGCCCGGAGATGCCCCATACTGCACTCCCGAACAATACAAGGAGAGCGGGAGACCGGGGTCTCTGAGCTGTGCCGACCCTGCACTTGACTTTCTCGTTGAAAAAGATCAGGAGTATTGCGTGTGCGAGATGCCCTGTAATCTTACACGGTACGGTAAGGAACTTAGTAGGAGACGGGAGAACCTATCC
asic1a_Frag993-1152_insertion1059	GATACTTAACGTCGTCTCCTTGTAACTGTAGGATGGTTCACATGCCCGGAGATGCCCCATACTGCACTCCCGAACAATACAAGGAGTGTAGCGGGAGACCGGGGTCTCTGAGCGCCGACCCTGCACTTGACTTTCTCGTTGAAAAAGATCAGGAGTATTGCGTGTGCGAGATGCCCTGTAATCTTACACGGTACGGTAAGGAACTTAGTAGGAGACGGGAGAACCTATCC
asic1a_Frag993-1152_insertion1062	GATACTTAACGTCGTCTCCTTGTAACTGTAGGATGGTTCACATGCCCGGAGATGCCCCATACTGCACTCCCGAACAATACAAGGAGTGTGCCAGCGGGAGACCGGGGTCTCTGAGCGACCCTGCACTTGACTTTCTCGTTGAAAAAGATCAGGAGTATTGCGTGTGCGAGATGCCCTGTAATCTTACACGGTACGGTAAGGAACTTAGTAGGAGACGGGAGAACCTATCC
asic1a_Frag993-1152_insertion1065	GATACTTAACGTCGTCTCCTTGTAACTGTAGGATGGTTCACATGCCCGGAGATGCCCCATACTGCACTCCCGAACAATACAAGGAGTGTGCCGACAGCGGGAGACCGGGGTCTCTGAGCCCTGCACTTGACTTTCTCGTTGAAAAAGATCAGGAGTATTGCGTGTGCGAGATGCCCTGTAATCTTACACGGTACGGTAAGGAACTTAGTAGGAGACGGGAGAACCTATCC
asic1a_Frag993-1152_insertion1068	GATACTTAACGTCGTCTCCTTGTAACTGTAGGATGGTTCACATGCCCGGAGATGCCCCATACTGCACTCCCGAACAATACAAGGAGTGTGCCGACCCTAGCGGGAGACCGGGGTCTCTGAGCGCACTTGACTTTCTCGTTGAAAAAGATCAGGAGTATTGCGTGTGCGAGATGCCCTGTAATCTTACACGGTACGGTAAGGAACTTAGTAGGAGACGGGAGAACCTATCC
asic1a_Frag993-1152_insertion1071	GATACTTAACGTCGTCTCCTTGTAACTGTAGGATGGTTCACATGCCCGGAGATGCCCCATACTGCACTCCCGAACAATACAAGGAGTGTGCCGACCCTGCAAGCGGGAGACCGGGGTCTCTGAGCCTTGACTTTCTCGTTGAAAAAGATCAGGAGTATTGCGTGTGCGAGATGCCCTGTAATCTTACACGGTACGGTAAGGAACTTAGTAGGAGACGGGAGAACCTATCC
asic1a_Frag993-1152_insertion1074	GATACTTAACGTCGTCTCCTTGTAACTGTAGGATGGTTCACATGCCCGGAGATGCCCCATACTGCACTCCCGAACAATACAAGGAGTGTGCCGACCCTGCACTTAGCGGGAGACCGGGGTCTCTGAGCGACTTTCTCGTTGAAAAAGATCAGGAGTATTGCGTGTGCGAGATGCCCTGTAATCTTACACGGTACGGTAAGGAACTTAGTAGGAGACGGGAGAACCTATCC
asic1a_Frag993-1152_insertion1077	GATACTTAACGTCGTCTCCTTGTAACTGTAGGATGGTTCACATGCCCGGAGATGCCCCATACTGCACTCCCGAACAATACAAGGAGTGTGCCGACCCTGCACTTGACAGCGGGAGACCGGGGTCTCTGAGCTTTCTCGTTGAAAAAGATCAGGAGTATTGCGTGTGCGAGATGCCCTGTAATCTTACACGGTACGGTAAGGAACTTAGTAGGAGACGGGAGAACCTATCC
asic1a_Frag993-1152_insertion1080	GATACTTAACGTCGTCTCCTTGTAACTGTAGGATGGTTCACATGCCCGGAGATGCCCCATACTGCACTCCCGAACAATACAAGGAGTGTGCCGACCCTGCACTTGACTTTAGCGGGAGACCGGGGTCTCTGAGCCTCGTTGAAAAAGATCAGGAGTATTGCGTGTGCGAGATGCCCTGTAATCTTACACGGTACGGTAAGGAACTTAGTAGGAGACGGGAGAACCTATCC
asic1a_Frag993-1152_insertion1083	GATACTTAACGTCGTCTCCTTGTAACTGTAGGATGGTTCACATGCCCGGAGATGCCCCATACTGCACTCCCGAACAATACAAGGAGTGTGCCGACCCTGCACTTGACTTTCTCAGCGGGAGACCGGGGTCTCTGAGCGTTGAAAAAGATCAGGAGTATTGCGTGTGCGAGATGCCCTGTAATCTTACACGGTACGGTAAGGAACTTAGTAGGAGACGGGAGAACCTATCC
asic1a_Frag993-1152_insertion1086	GATACTTAACGTCGTCTCCTTGTAACTGTAGGATGGTTCACATGCCCGGAGATGCCCCATACTGCACTCCCGAACAATACAAGGAGTGTGCCGACCCTGCACTTGACTTTCTCGTTAGCGGGAGACCGGGGTCTCTGAGCGAAAAAGATCAGGAGTATTGCGTGTGCGAGATGCCCTGTAATCTTACACGGTACGGTAAGGAACTTAGTAGGAGACGGGAGAACCTATCC
asic1a_Frag993-1152_insertion1089	GATACTTAACGTCGTCTCCTTGTAACTGTAGGATGGTTCACATGCCCGGAGATGCCCCATACTGCACTCCCGAACAATACAAGGAGTGTGCCGACCCTGCACTTGACTTTCTCGTTGAAAGCGGGAGACCGGGGTCTCTGAGCAAAGATCAGGAGTATTGCGTGTGCGAGATGCCCTGTAATCTTACACGGTACGGTAAGGAACTTAGTAGGAGACGGGAGAACCTATCC
asic1a_Frag993-1152_insertion1092	GATACTTAACGTCGTCTCCTTGTAACTGTAGGATGGTTCACATGCCCGGAGATGCCCCATACTGCACTCCCGAACAATACAAGGAGTGTGCCGACCCTGCACTTGACTTTCTCGTTGAAAAAAGCGGGAGACCGGGGTCTCTGAGCGATCAGGAGTATTGCGTGTGCGAGATGCCCTGTAATCTTACACGGTACGGTAAGGAACTTAGTAGGAGACGGGAGAACCTATCC
asic1a_Frag993-1152_insertion1095	GATACTTAACGTCGTCTCCTTGTAACTGTAGGATGGTTCACATGCCCGGAGATGCCCCATACTGCACTCCCGAACAATACAAGGAGTGTGCCGACCCTGCACTTGACTTTCTCGTTGAAAAAGATAGCGGGAGACCGGGGTCTCTGAGCCAGGAGTATTGCGTGTGCGAGATGCCCTGTAATCTTACACGGTACGGTAAGGAACTTAGTAGGAGACGGGAGAACCTATCC
asic1a_Frag993-1152_insertion1098	GATACTTAACGTCGTCTCCTTGTAACTGTAGGATGGTTCACATGCCCGGAGATGCCCCATACTGCACTCCCGAACAATACAAGGAGTGTGCCGACCCTGCACTTGACTTTCTCGTTGAAAAAGATCAGAGCGGGAGACCGGGGTCTCTGAGCGAGTATTGCGTGTGCGAGATGCCCTGTAATCTTACACGGTACGGTAAGGAACTTAGTAGGAGACGGGAGAACCTATCC
asic1a_Frag993-1152_insertion1101	GATACTTAACGTCGTCTCCTTGTAACTGTAGGATGGTTCACATGCCCGGAGATGCCCCATACTGCACTCCCGAACAATACAAGGAGTGTGCCGACCCTGCACTTGACTTTCTCGTTGAAAAAGATCAGGAGAGCGGGAGACCGGGGTCTCTGAGCTATTGCGTGTGCGAGATGCCCTGTAATCTTACACGGTACGGTAAGGAACTTAGTAGGAGACGGGAGAACCTATCC
asic1a_Frag993-1152_insertion1104	GATACTTAACGTCGTCTCCTTGTAACTGTAGGATGGTTCACATGCCCGGAGATGCCCCATACTGCACTCCCGAACAATACAAGGAGTGTGCCGACCCTGCACTTGACTTTCTCGTTGAAAAAGATCAGGAGTATAGCGGGAGACCGGGGTCTCTGAGCTGCGTGTGCGAGATGCCCTGTAATCTTACACGGTACGGTAAGGAACTTAGTAGGAGACGGGAGAACCTATCC
asic1a_Frag993-1152_insertion1107	GATACTTAACGTCGTCTCCTTGTAACTGTAGGATGGTTCACATGCCCGGAGATGCCCCATACTGCACTCCCGAACAATACAAGGAGTGTGCCGACCCTGCACTTGACTTTCTCGTTGAAAAAGATCAGGAGTATTGCAGCGGGAGACCGGGGTCTCTGAGCGTGTGCGAGATGCCCTGTAATCTTACACGGTACGGTAAGGAACTTAGTAGGAGACGGGAGAACCTATCC
asic1a_Frag993-1152_insertion1110	GATACTTAACGTCGTCTCCTTGTAACTGTAGGATGGTTCACATGCCCGGAGATGCCCCATACTGCACTCCCGAACAATACAAGGAGTGTGCCGACCCTGCACTTGACTTTCTCGTTGAAAAAGATCAGGAGTATTGCGTGAGCGGGAGACCGGGGTCTCTGAGCTGCGAGATGCCCTGTAATCTTACACGGTACGGTAAGGAACTTAGTAGGAGACGGGAGAACCTATCC
asic1a_Frag993-1152_insertion1113	GATACTTAACGTCGTCTCCTTGTAACTGTAGGATGGTTCACATGCCCGGAGATGCCCCATACTGCACTCCCGAACAATACAAGGAGTGTGCCGACCCTGCACTTGACTTTCTCGTTGAAAAAGATCAGGAGTATTGCGTGTGCAGCGGGAGACCGGGGTCTCTGAGCGAGATGCCCTGTAATCTTACACGGTACGGTAAGGAACTTAGTAGGAGACGGGAGAACCTATCC
asic1a_Frag993-1152_insertion1116	GATACTTAACGTCGTCTCCTTGTAACTGTAGGATGGTTCACATGCCCGGAGATGCCCCATACTGCACTCCCGAACAATACAAGGAGTGTGCCGACCCTGCACTTGACTTTCTCGTTGAAAAAGATCAGGAGTATTGCGTGTGCGAGAGCGGGAGACCGGGGTCTCTGAGCATGCCCTGTAATCTTACACGGTACGGTAAGGAACTTAGTAGGAGACGGGAGAACCTATCC
asic1a_Frag993-1152_insertion1119	GATACTTAACGTCGTCTCCTTGTAACTGTAGGATGGTTCACATGCCCGGAGATGCCCCATACTGCACTCCCGAACAATACAAGGAGTGTGCCGACCCTGCACTTGACTTTCTCGTTGAAAAAGATCAGGAGTATTGCGTGTGCGAGATGAGCGGGAGACCGGGGTCTCTGAGCCCCTGTAATCTTACACGGTACGGTAAGGAACTTAGTAGGAGACGGGAGAACCTATCC
asic1a_Frag993-1152_insertion1122	GATACTTAACGTCGTCTCCTTGTAACTGTAGGATGGTTCACATGCCCGGAGATGCCCCATACTGCACTCCCGAACAATACAAGGAGTGTGCCGACCCTGCACTTGACTTTCTCGTTGAAAAAGATCAGGAGTATTGCGTGTGCGAGATGCCCAGCGGGAGACCGGGGTCTCTGAGCTGTAATCTTACACGGTACGGTAAGGAACTTAGTAGGAGACGGGAGAACCTATCC
asic1a_Frag993-1152_insertion1125	GATACTTAACGTCGTCTCCTTGTAACTGTAGGATGGTTCACATGCCCGGAGATGCCCCATACTGCACTCCCGAACAATACAAGGAGTGTGCCGACCCTGCACTTGACTTTCTCGTTGAAAAAGATCAGGAGTATTGCGTGTGCGAGATGCCCTGTAGCGGGAGACCGGGGTCTCTGAGCAATCTTACACGGTACGGTAAGGAACTTAGTAGGAGACGGGAGAACCTATCC
asic1a_Frag993-1152_insertion1128	GATACTTAACGTCGTCTCCTTGTAACTGTAGGATGGTTCACATGCCCGGAGATGCCCCATACTGCACTCCCGAACAATACAAGGAGTGTGCCGACCCTGCACTTGACTTTCTCGTTGAAAAAGATCAGGAGTATTGCGTGTGCGAGATGCCCTGTAATAGCGGGAGACCGGGGTCTCTGAGCCTTACACGGTACGGTAAGGAACTTAGTAGGAGACGGGAGAACCTATCC
asic1a_Frag993-1152_insertion1131	GATACTTAACGTCGTCTCCTTGTAACTGTAGGATGGTTCACATGCCCGGAGATGCCCCATACTGCACTCCCGAACAATACAAGGAGTGTGCCGACCCTGCACTTGACTTTCTCGTTGAAAAAGATCAGGAGTATTGCGTGTGCGAGATGCCCTGTAATCTTAGCGGGAGACCGGGGTCTCTGAGCACACGGTACGGTAAGGAACTTAGTAGGAGACGGGAGAACCTATCC
asic1a_Frag993-1152_insertion1134	GATACTTAACGTCGTCTCCTTGTAACTGTAGGATGGTTCACATGCCCGGAGATGCCCCATACTGCACTCCCGAACAATACAAGGAGTGTGCCGACCCTGCACTTGACTTTCTCGTTGAAAAAGATCAGGAGTATTGCGTGTGCGAGATGCCCTGTAATCTTACAAGCGGGAGACCGGGGTCTCTGAGCCGGTACGGTAAGGAACTTAGTAGGAGACGGGAGAACCTATCC
asic1a_Frag993-1152_insertion1137	GATACTTAACGTCGTCTCCTTGTAACTGTAGGATGGTTCACATGCCCGGAGATGCCCCATACTGCACTCCCGAACAATACAAGGAGTGTGCCGACCCTGCACTTGACTTTCTCGTTGAAAAAGATCAGGAGTATTGCGTGTGCGAGATGCCCTGTAATCTTACACGGAGCGGGAGACCGGGGTCTCTGAGCTACGGTAAGGAACTTAGTAGGAGACGGGAGAACCTATCC
asic1a_Frag993-1152_insertion1140	GATACTTAACGTCGTCTCCTTGTAACTGTAGGATGGTTCACATGCCCGGAGATGCCCCATACTGCACTCCCGAACAATACAAGGAGTGTGCCGACCCTGCACTTGACTTTCTCGTTGAAAAAGATCAGGAGTATTGCGTGTGCGAGATGCCCTGTAATCTTACACGGTACAGCGGGAGACCGGGGTCTCTGAGCGGTAAGGAACTTAGTAGGAGACGGGAGAACCTATCC
asic1a_Frag993-1152_insertion1143	GATACTTAACGTCGTCTCCTTGTAACTGTAGGATGGTTCACATGCCCGGAGATGCCCCATACTGCACTCCCGAACAATACAAGGAGTGTGCCGACCCTGCACTTGACTTTCTCGTTGAAAAAGATCAGGAGTATTGCGTGTGCGAGATGCCCTGTAATCTTACACGGTACGGTAGCGGGAGACCGGGGTCTCTGAGCAAGGAACTTAGTAGGAGACGGGAGAACCTATCC
asic1a_Frag993-1152_insertion1146	GATACTTAACGTCGTCTCCTTGTAACTGTAGGATGGTTCACATGCCCGGAGATGCCCCATACTGCACTCCCGAACAATACAAGGAGTGTGCCGACCCTGCACTTGACTTTCTCGTTGAAAAAGATCAGGAGTATTGCGTGTGCGAGATGCCCTGTAATCTTACACGGTACGGTAAGAGCGGGAGACCGGGGTCTCTGAGCGAACTTAGTAGGAGACGGGAGAACCTATCC
asic1a_Frag993-1152_insertion1149	GATACTTAACGTCGTCTCCTTGTAACTGTAGGATGGTTCACATGCCCGGAGATGCCCCATACTGCACTCCCGAACAATACAAGGAGTGTGCCGACCCTGCACTTGACTTTCTCGTTGAAAAAGATCAGGAGTATTGCGTGTGCGAGATGCCCTGTAATCTTACACGGTACGGTAAGGAAAGCGGGAGACCGGGGTCTCTGAGCCTTAGTAGGAGACGGGAGAACCTATCC
asic1a_Frag993-1152_insertion1152	GATACTTAACGTCGTCTCCTTGTAACTGTAGGATGGTTCACATGCCCGGAGATGCCCCATACTGCACTCCCGAACAATACAAGGAGTGTGCCGACCCTGCACTTGACTTTCTCGTTGAAAAAGATCAGGAGTATTGCGTGTGCGAGATGCCCTGTAATCTTACACGGTACGGTAAGGAACTTAGCGGGAGACCGGGGTCTCTGAGCAGTAGGAGACGGGAGAACCTATCC
asic1a_Frag1155-1314_insertion1155	TAGGAGCGGGATCGTCTCCTAGTAGCGGGAGACCGGGGTCTCTGAGCATGGTCAAAATTCCAAGTAAAGCCAGTGCAAAATACTTGGCTAAGAAGTTCAACAAAAGCGAGCAGTACATCGGCGAGAACATTTTGGTTCTCGACATATTCTTCGAAGTCCTGAACTACGAAACTATTGAACAGAAAAAGGCATACGAGATAGCAGGTCTTTGGAGACGGTCCATGCGGATA
asic1a_Frag1155-1314_insertion1158	TAGGAGCGGGATCGTCTCCTAGTATGAGCGGGAGACCGGGGTCTCTGAGCGTCAAAATTCCAAGTAAAGCCAGTGCAAAATACTTGGCTAAGAAGTTCAACAAAAGCGAGCAGTACATCGGCGAGAACATTTTGGTTCTCGACATATTCTTCGAAGTCCTGAACTACGAAACTATTGAACAGAAAAAGGCATACGAGATAGCAGGTCTTTGGAGACGGTCCATGCGGATA
asic1a_Frag1155-1314_insertion1161	TAGGAGCGGGATCGTCTCCTAGTATGGTCAGCGGGAGACCGGGGTCTCTGAGCAAAATTCCAAGTAAAGCCAGTGCAAAATACTTGGCTAAGAAGTTCAACAAAAGCGAGCAGTACATCGGCGAGAACATTTTGGTTCTCGACATATTCTTCGAAGTCCTGAACTACGAAACTATTGAACAGAAAAAGGCATACGAGATAGCAGGTCTTTGGAGACGGTCCATGCGGATA
asic1a_Frag1155-1314_insertion1164	TAGGAGCGGGATCGTCTCCTAGTATGGTCAAAAGCGGGAGACCGGGGTCTCTGAGCATTCCAAGTAAAGCCAGTGCAAAATACTTGGCTAAGAAGTTCAACAAAAGCGAGCAGTACATCGGCGAGAACATTTTGGTTCTCGACATATTCTTCGAAGTCCTGAACTACGAAACTATTGAACAGAAAAAGGCATACGAGATAGCAGGTCTTTGGAGACGGTCCATGCGGATA
asic1a_Frag1155-1314_insertion1167	TAGGAGCGGGATCGTCTCCTAGTATGGTCAAAATTAGCGGGAGACCGGGGTCTCTGAGCCCAAGTAAAGCCAGTGCAAAATACTTGGCTAAGAAGTTCAACAAAAGCGAGCAGTACATCGGCGAGAACATTTTGGTTCTCGACATATTCTTCGAAGTCCTGAACTACGAAACTATTGAACAGAAAAAGGCATACGAGATAGCAGGTCTTTGGAGACGGTCCATGCGGATA
asic1a_Frag1155-1314_insertion1170	TAGGAGCGGGATCGTCTCCTAGTATGGTCAAAATTCCAAGCGGGAGACCGGGGTCTCTGAGCAGTAAAGCCAGTGCAAAATACTTGGCTAAGAAGTTCAACAAAAGCGAGCAGTACATCGGCGAGAACATTTTGGTTCTCGACATATTCTTCGAAGTCCTGAACTACGAAACTATTGAACAGAAAAAGGCATACGAGATAGCAGGTCTTTGGAGACGGTCCATGCGGATA
asic1a_Frag1155-1314_insertion1173	TAGGAGCGGGATCGTCTCCTAGTATGGTCAAAATTCCAAGTAGCGGGAGACCGGGGTCTCTGAGCAAAGCCAGTGCAAAATACTTGGCTAAGAAGTTCAACAAAAGCGAGCAGTACATCGGCGAGAACATTTTGGTTCTCGACATATTCTTCGAAGTCCTGAACTACGAAACTATTGAACAGAAAAAGGCATACGAGATAGCAGGTCTTTGGAGACGGTCCATGCGGATA
asic1a_Frag1155-1314_insertion1176	TAGGAGCGGGATCGTCTCCTAGTATGGTCAAAATTCCAAGTAAAAGCGGGAGACCGGGGTCTCTGAGCGCCAGTGCAAAATACTTGGCTAAGAAGTTCAACAAAAGCGAGCAGTACATCGGCGAGAACATTTTGGTTCTCGACATATTCTTCGAAGTCCTGAACTACGAAACTATTGAACAGAAAAAGGCATACGAGATAGCAGGTCTTTGGAGACGGTCCATGCGGATA
asic1a_Frag1155-1314_insertion1179	TAGGAGCGGGATCGTCTCCTAGTATGGTCAAAATTCCAAGTAAAGCCAGCGGGAGACCGGGGTCTCTGAGCAGTGCAAAATACTTGGCTAAGAAGTTCAACAAAAGCGAGCAGTACATCGGCGAGAACATTTTGGTTCTCGACATATTCTTCGAAGTCCTGAACTACGAAACTATTGAACAGAAAAAGGCATACGAGATAGCAGGTCTTTGGAGACGGTCCATGCGGATA
asic1a_Frag1155-1314_insertion1182	TAGGAGCGGGATCGTCTCCTAGTATGGTCAAAATTCCAAGTAAAGCCAGTAGCGGGAGACCGGGGTCTCTGAGCGCAAAATACTTGGCTAAGAAGTTCAACAAAAGCGAGCAGTACATCGGCGAGAACATTTTGGTTCTCGACATATTCTTCGAAGTCCTGAACTACGAAACTATTGAACAGAAAAAGGCATACGAGATAGCAGGTCTTTGGAGACGGTCCATGCGGATA
asic1a_Frag1155-1314_insertion1185	TAGGAGCGGGATCGTCTCCTAGTATGGTCAAAATTCCAAGTAAAGCCAGTGCAAGCGGGAGACCGGGGTCTCTGAGCAAATACTTGGCTAAGAAGTTCAACAAAAGCGAGCAGTACATCGGCGAGAACATTTTGGTTCTCGACATATTCTTCGAAGTCCTGAACTACGAAACTATTGAACAGAAAAAGGCATACGAGATAGCAGGTCTTTGGAGACGGTCCATGCGGATA
asic1a_Frag1155-1314_insertion1188	TAGGAGCGGGATCGTCTCCTAGTATGGTCAAAATTCCAAGTAAAGCCAGTGCAAAAAGCGGGAGACCGGGGTCTCTGAGCTACTTGGCTAAGAAGTTCAACAAAAGCGAGCAGTACATCGGCGAGAACATTTTGGTTCTCGACATATTCTTCGAAGTCCTGAACTACGAAACTATTGAACAGAAAAAGGCATACGAGATAGCAGGTCTTTGGAGACGGTCCATGCGGATA
asic1a_Frag1155-1314_insertion1191	TAGGAGCGGGATCGTCTCCTAGTATGGTCAAAATTCCAAGTAAAGCCAGTGCAAAATACAGCGGGAGACCGGGGTCTCTGAGCTTGGCTAAGAAGTTCAACAAAAGCGAGCAGTACATCGGCGAGAACATTTTGGTTCTCGACATATTCTTCGAAGTCCTGAACTACGAAACTATTGAACAGAAAAAGGCATACGAGATAGCAGGTCTTTGGAGACGGTCCATGCGGATA
asic1a_Frag1155-1314_insertion1194	TAGGAGCGGGATCGTCTCCTAGTATGGTCAAAATTCCAAGTAAAGCCAGTGCAAAATACTTGAGCGGGAGACCGGGGTCTCTGAGCGCTAAGAAGTTCAACAAAAGCGAGCAGTACATCGGCGAGAACATTTTGGTTCTCGACATATTCTTCGAAGTCCTGAACTACGAAACTATTGAACAGAAAAAGGCATACGAGATAGCAGGTCTTTGGAGACGGTCCATGCGGATA
asic1a_Frag1155-1314_insertion1197	TAGGAGCGGGATCGTCTCCTAGTATGGTCAAAATTCCAAGTAAAGCCAGTGCAAAATACTTGGCTAGCGGGAGACCGGGGTCTCTGAGCAAGAAGTTCAACAAAAGCGAGCAGTACATCGGCGAGAACATTTTGGTTCTCGACATATTCTTCGAAGTCCTGAACTACGAAACTATTGAACAGAAAAAGGCATACGAGATAGCAGGTCTTTGGAGACGGTCCATGCGGATA
asic1a_Frag1155-1314_insertion1200	TAGGAGCGGGATCGTCTCCTAGTATGGTCAAAATTCCAAGTAAAGCCAGTGCAAAATACTTGGCTAAGAGCGGGAGACCGGGGTCTCTGAGCAAGTTCAACAAAAGCGAGCAGTACATCGGCGAGAACATTTTGGTTCTCGACATATTCTTCGAAGTCCTGAACTACGAAACTATTGAACAGAAAAAGGCATACGAGATAGCAGGTCTTTGGAGACGGTCCATGCGGATA
asic1a_Frag1155-1314_insertion1203	TAGGAGCGGGATCGTCTCCTAGTATGGTCAAAATTCCAAGTAAAGCCAGTGCAAAATACTTGGCTAAGAAGAGCGGGAGACCGGGGTCTCTGAGCTTCAACAAAAGCGAGCAGTACATCGGCGAGAACATTTTGGTTCTCGACATATTCTTCGAAGTCCTGAACTACGAAACTATTGAACAGAAAAAGGCATACGAGATAGCAGGTCTTTGGAGACGGTCCATGCGGATA
asic1a_Frag1155-1314_insertion1206	TAGGAGCGGGATCGTCTCCTAGTATGGTCAAAATTCCAAGTAAAGCCAGTGCAAAATACTTGGCTAAGAAGTTCAGCGGGAGACCGGGGTCTCTGAGCAACAAAAGCGAGCAGTACATCGGCGAGAACATTTTGGTTCTCGACATATTCTTCGAAGTCCTGAACTACGAAACTATTGAACAGAAAAAGGCATACGAGATAGCAGGTCTTTGGAGACGGTCCATGCGGATA
asic1a_Frag1155-1314_insertion1209	TAGGAGCGGGATCGTCTCCTAGTATGGTCAAAATTCCAAGTAAAGCCAGTGCAAAATACTTGGCTAAGAAGTTCAACAGCGGGAGACCGGGGTCTCTGAGCAAAAGCGAGCAGTACATCGGCGAGAACATTTTGGTTCTCGACATATTCTTCGAAGTCCTGAACTACGAAACTATTGAACAGAAAAAGGCATACGAGATAGCAGGTCTTTGGAGACGGTCCATGCGGATA
asic1a_Frag1155-1314_insertion1212	TAGGAGCGGGATCGTCTCCTAGTATGGTCAAAATTCCAAGTAAAGCCAGTGCAAAATACTTGGCTAAGAAGTTCAACAAAAGCGGGAGACCGGGGTCTCTGAGCAGCGAGCAGTACATCGGCGAGAACATTTTGGTTCTCGACATATTCTTCGAAGTCCTGAACTACGAAACTATTGAACAGAAAAAGGCATACGAGATAGCAGGTCTTTGGAGACGGTCCATGCGGATA
asic1a_Frag1155-1314_insertion1215	TAGGAGCGGGATCGTCTCCTAGTATGGTCAAAATTCCAAGTAAAGCCAGTGCAAAATACTTGGCTAAGAAGTTCAACAAAAGCAGCGGGAGACCGGGGTCTCTGAGCGAGCAGTACATCGGCGAGAACATTTTGGTTCTCGACATATTCTTCGAAGTCCTGAACTACGAAACTATTGAACAGAAAAAGGCATACGAGATAGCAGGTCTTTGGAGACGGTCCATGCGGATA
asic1a_Frag1155-1314_insertion1218	TAGGAGCGGGATCGTCTCCTAGTATGGTCAAAATTCCAAGTAAAGCCAGTGCAAAATACTTGGCTAAGAAGTTCAACAAAAGCGAGAGCGGGAGACCGGGGTCTCTGAGCCAGTACATCGGCGAGAACATTTTGGTTCTCGACATATTCTTCGAAGTCCTGAACTACGAAACTATTGAACAGAAAAAGGCATACGAGATAGCAGGTCTTTGGAGACGGTCCATGCGGATA
asic1a_Frag1155-1314_insertion1221	TAGGAGCGGGATCGTCTCCTAGTATGGTCAAAATTCCAAGTAAAGCCAGTGCAAAATACTTGGCTAAGAAGTTCAACAAAAGCGAGCAGAGCGGGAGACCGGGGTCTCTGAGCTACATCGGCGAGAACATTTTGGTTCTCGACATATTCTTCGAAGTCCTGAACTACGAAACTATTGAACAGAAAAAGGCATACGAGATAGCAGGTCTTTGGAGACGGTCCATGCGGATA
asic1a_Frag1155-1314_insertion1224	TAGGAGCGGGATCGTCTCCTAGTATGGTCAAAATTCCAAGTAAAGCCAGTGCAAAATACTTGGCTAAGAAGTTCAACAAAAGCGAGCAGTACAGCGGGAGACCGGGGTCTCTGAGCATCGGCGAGAACATTTTGGTTCTCGACATATTCTTCGAAGTCCTGAACTACGAAACTATTGAACAGAAAAAGGCATACGAGATAGCAGGTCTTTGGAGACGGTCCATGCGGATA
asic1a_Frag1155-1314_insertion1227	TAGGAGCGGGATCGTCTCCTAGTATGGTCAAAATTCCAAGTAAAGCCAGTGCAAAATACTTGGCTAAGAAGTTCAACAAAAGCGAGCAGTACATCAGCGGGAGACCGGGGTCTCTGAGCGGCGAGAACATTTTGGTTCTCGACATATTCTTCGAAGTCCTGAACTACGAAACTATTGAACAGAAAAAGGCATACGAGATAGCAGGTCTTTGGAGACGGTCCATGCGGATA
asic1a_Frag1155-1314_insertion1230	TAGGAGCGGGATCGTCTCCTAGTATGGTCAAAATTCCAAGTAAAGCCAGTGCAAAATACTTGGCTAAGAAGTTCAACAAAAGCGAGCAGTACATCGGCAGCGGGAGACCGGGGTCTCTGAGCGAGAACATTTTGGTTCTCGACATATTCTTCGAAGTCCTGAACTACGAAACTATTGAACAGAAAAAGGCATACGAGATAGCAGGTCTTTGGAGACGGTCCATGCGGATA
asic1a_Frag1155-1314_insertion1233	TAGGAGCGGGATCGTCTCCTAGTATGGTCAAAATTCCAAGTAAAGCCAGTGCAAAATACTTGGCTAAGAAGTTCAACAAAAGCGAGCAGTACATCGGCGAGAGCGGGAGACCGGGGTCTCTGAGCAACATTTTGGTTCTCGACATATTCTTCGAAGTCCTGAACTACGAAACTATTGAACAGAAAAAGGCATACGAGATAGCAGGTCTTTGGAGACGGTCCATGCGGATA
asic1a_Frag1155-1314_insertion1236	TAGGAGCGGGATCGTCTCCTAGTATGGTCAAAATTCCAAGTAAAGCCAGTGCAAAATACTTGGCTAAGAAGTTCAACAAAAGCGAGCAGTACATCGGCGAGAACAGCGGGAGACCGGGGTCTCTGAGCATTTTGGTTCTCGACATATTCTTCGAAGTCCTGAACTACGAAACTATTGAACAGAAAAAGGCATACGAGATAGCAGGTCTTTGGAGACGGTCCATGCGGATA
asic1a_Frag1155-1314_insertion1239	TAGGAGCGGGATCGTCTCCTAGTATGGTCAAAATTCCAAGTAAAGCCAGTGCAAAATACTTGGCTAAGAAGTTCAACAAAAGCGAGCAGTACATCGGCGAGAACATTAGCGGGAGACCGGGGTCTCTGAGCTTGGTTCTCGACATATTCTTCGAAGTCCTGAACTACGAAACTATTGAACAGAAAAAGGCATACGAGATAGCAGGTCTTTGGAGACGGTCCATGCGGATA
asic1a_Frag1155-1314_insertion1242	TAGGAGCGGGATCGTCTCCTAGTATGGTCAAAATTCCAAGTAAAGCCAGTGCAAAATACTTGGCTAAGAAGTTCAACAAAAGCGAGCAGTACATCGGCGAGAACATTTTGAGCGGGAGACCGGGGTCTCTGAGCGTTCTCGACATATTCTTCGAAGTCCTGAACTACGAAACTATTGAACAGAAAAAGGCATACGAGATAGCAGGTCTTTGGAGACGGTCCATGCGGATA
asic1a_Frag1155-1314_insertion1245	TAGGAGCGGGATCGTCTCCTAGTATGGTCAAAATTCCAAGTAAAGCCAGTGCAAAATACTTGGCTAAGAAGTTCAACAAAAGCGAGCAGTACATCGGCGAGAACATTTTGGTTAGCGGGAGACCGGGGTCTCTGAGCCTCGACATATTCTTCGAAGTCCTGAACTACGAAACTATTGAACAGAAAAAGGCATACGAGATAGCAGGTCTTTGGAGACGGTCCATGCGGATA
asic1a_Frag1155-1314_insertion1248	TAGGAGCGGGATCGTCTCCTAGTATGGTCAAAATTCCAAGTAAAGCCAGTGCAAAATACTTGGCTAAGAAGTTCAACAAAAGCGAGCAGTACATCGGCGAGAACATTTTGGTTCTCAGCGGGAGACCGGGGTCTCTGAGCGACATATTCTTCGAAGTCCTGAACTACGAAACTATTGAACAGAAAAAGGCATACGAGATAGCAGGTCTTTGGAGACGGTCCATGCGGATA
asic1a_Frag1155-1314_insertion1251	TAGGAGCGGGATCGTCTCCTAGTATGGTCAAAATTCCAAGTAAAGCCAGTGCAAAATACTTGGCTAAGAAGTTCAACAAAAGCGAGCAGTACATCGGCGAGAACATTTTGGTTCTCGACAGCGGGAGACCGGGGTCTCTGAGCATATTCTTCGAAGTCCTGAACTACGAAACTATTGAACAGAAAAAGGCATACGAGATAGCAGGTCTTTGGAGACGGTCCATGCGGATA
asic1a_Frag1155-1314_insertion1254	TAGGAGCGGGATCGTCTCCTAGTATGGTCAAAATTCCAAGTAAAGCCAGTGCAAAATACTTGGCTAAGAAGTTCAACAAAAGCGAGCAGTACATCGGCGAGAACATTTTGGTTCTCGACATAAGCGGGAGACCGGGGTCTCTGAGCTTCTTCGAAGTCCTGAACTACGAAACTATTGAACAGAAAAAGGCATACGAGATAGCAGGTCTTTGGAGACGGTCCATGCGGATA
asic1a_Frag1155-1314_insertion1257	TAGGAGCGGGATCGTCTCCTAGTATGGTCAAAATTCCAAGTAAAGCCAGTGCAAAATACTTGGCTAAGAAGTTCAACAAAAGCGAGCAGTACATCGGCGAGAACATTTTGGTTCTCGACATATTCAGCGGGAGACCGGGGTCTCTGAGCTTCGAAGTCCTGAACTACGAAACTATTGAACAGAAAAAGGCATACGAGATAGCAGGTCTTTGGAGACGGTCCATGCGGATA
asic1a_Frag1155-1314_insertion1260	TAGGAGCGGGATCGTCTCCTAGTATGGTCAAAATTCCAAGTAAAGCCAGTGCAAAATACTTGGCTAAGAAGTTCAACAAAAGCGAGCAGTACATCGGCGAGAACATTTTGGTTCTCGACATATTCTTCAGCGGGAGACCGGGGTCTCTGAGCGAAGTCCTGAACTACGAAACTATTGAACAGAAAAAGGCATACGAGATAGCAGGTCTTTGGAGACGGTCCATGCGGATA
asic1a_Frag1155-1314_insertion1263	TAGGAGCGGGATCGTCTCCTAGTATGGTCAAAATTCCAAGTAAAGCCAGTGCAAAATACTTGGCTAAGAAGTTCAACAAAAGCGAGCAGTACATCGGCGAGAACATTTTGGTTCTCGACATATTCTTCGAAAGCGGGAGACCGGGGTCTCTGAGCGTCCTGAACTACGAAACTATTGAACAGAAAAAGGCATACGAGATAGCAGGTCTTTGGAGACGGTCCATGCGGATA
asic1a_Frag1155-1314_insertion1266	TAGGAGCGGGATCGTCTCCTAGTATGGTCAAAATTCCAAGTAAAGCCAGTGCAAAATACTTGGCTAAGAAGTTCAACAAAAGCGAGCAGTACATCGGCGAGAACATTTTGGTTCTCGACATATTCTTCGAAGTCAGCGGGAGACCGGGGTCTCTGAGCCTGAACTACGAAACTATTGAACAGAAAAAGGCATACGAGATAGCAGGTCTTTGGAGACGGTCCATGCGGATA
asic1a_Frag1155-1314_insertion1269	TAGGAGCGGGATCGTCTCCTAGTATGGTCAAAATTCCAAGTAAAGCCAGTGCAAAATACTTGGCTAAGAAGTTCAACAAAAGCGAGCAGTACATCGGCGAGAACATTTTGGTTCTCGACATATTCTTCGAAGTCCTGAGCGGGAGACCGGGGTCTCTGAGCAACTACGAAACTATTGAACAGAAAAAGGCATACGAGATAGCAGGTCTTTGGAGACGGTCCATGCGGATA
asic1a_Frag1155-1314_insertion1272	TAGGAGCGGGATCGTCTCCTAGTATGGTCAAAATTCCAAGTAAAGCCAGTGCAAAATACTTGGCTAAGAAGTTCAACAAAAGCGAGCAGTACATCGGCGAGAACATTTTGGTTCTCGACATATTCTTCGAAGTCCTGAACAGCGGGAGACCGGGGTCTCTGAGCTACGAAACTATTGAACAGAAAAAGGCATACGAGATAGCAGGTCTTTGGAGACGGTCCATGCGGATA
asic1a_Frag1155-1314_insertion1275	TAGGAGCGGGATCGTCTCCTAGTATGGTCAAAATTCCAAGTAAAGCCAGTGCAAAATACTTGGCTAAGAAGTTCAACAAAAGCGAGCAGTACATCGGCGAGAACATTTTGGTTCTCGACATATTCTTCGAAGTCCTGAACTACAGCGGGAGACCGGGGTCTCTGAGCGAAACTATTGAACAGAAAAAGGCATACGAGATAGCAGGTCTTTGGAGACGGTCCATGCGGATA
asic1a_Frag1155-1314_insertion1278	TAGGAGCGGGATCGTCTCCTAGTATGGTCAAAATTCCAAGTAAAGCCAGTGCAAAATACTTGGCTAAGAAGTTCAACAAAAGCGAGCAGTACATCGGCGAGAACATTTTGGTTCTCGACATATTCTTCGAAGTCCTGAACTACGAAAGCGGGAGACCGGGGTCTCTGAGCACTATTGAACAGAAAAAGGCATACGAGATAGCAGGTCTTTGGAGACGGTCCATGCGGATA
asic1a_Frag1155-1314_insertion1281	TAGGAGCGGGATCGTCTCCTAGTATGGTCAAAATTCCAAGTAAAGCCAGTGCAAAATACTTGGCTAAGAAGTTCAACAAAAGCGAGCAGTACATCGGCGAGAACATTTTGGTTCTCGACATATTCTTCGAAGTCCTGAACTACGAAACTAGCGGGAGACCGGGGTCTCTGAGCATTGAACAGAAAAAGGCATACGAGATAGCAGGTCTTTGGAGACGGTCCATGCGGATA
asic1a_Frag1155-1314_insertion1284	TAGGAGCGGGATCGTCTCCTAGTATGGTCAAAATTCCAAGTAAAGCCAGTGCAAAATACTTGGCTAAGAAGTTCAACAAAAGCGAGCAGTACATCGGCGAGAACATTTTGGTTCTCGACATATTCTTCGAAGTCCTGAACTACGAAACTATTAGCGGGAGACCGGGGTCTCTGAGCGAACAGAAAAAGGCATACGAGATAGCAGGTCTTTGGAGACGGTCCATGCGGATA
asic1a_Frag1155-1314_insertion1287	TAGGAGCGGGATCGTCTCCTAGTATGGTCAAAATTCCAAGTAAAGCCAGTGCAAAATACTTGGCTAAGAAGTTCAACAAAAGCGAGCAGTACATCGGCGAGAACATTTTGGTTCTCGACATATTCTTCGAAGTCCTGAACTACGAAACTATTGAAAGCGGGAGACCGGGGTCTCTGAGCCAGAAAAAGGCATACGAGATAGCAGGTCTTTGGAGACGGTCCATGCGGATA
asic1a_Frag1155-1314_insertion1290	TAGGAGCGGGATCGTCTCCTAGTATGGTCAAAATTCCAAGTAAAGCCAGTGCAAAATACTTGGCTAAGAAGTTCAACAAAAGCGAGCAGTACATCGGCGAGAACATTTTGGTTCTCGACATATTCTTCGAAGTCCTGAACTACGAAACTATTGAACAGAGCGGGAGACCGGGGTCTCTGAGCAAAAAGGCATACGAGATAGCAGGTCTTTGGAGACGGTCCATGCGGATA
asic1a_Frag1155-1314_insertion1293	TAGGAGCGGGATCGTCTCCTAGTATGGTCAAAATTCCAAGTAAAGCCAGTGCAAAATACTTGGCTAAGAAGTTCAACAAAAGCGAGCAGTACATCGGCGAGAACATTTTGGTTCTCGACATATTCTTCGAAGTCCTGAACTACGAAACTATTGAACAGAAAAGCGGGAGACCGGGGTCTCTGAGCAAGGCATACGAGATAGCAGGTCTTTGGAGACGGTCCATGCGGATA
asic1a_Frag1155-1314_insertion1296	TAGGAGCGGGATCGTCTCCTAGTATGGTCAAAATTCCAAGTAAAGCCAGTGCAAAATACTTGGCTAAGAAGTTCAACAAAAGCGAGCAGTACATCGGCGAGAACATTTTGGTTCTCGACATATTCTTCGAAGTCCTGAACTACGAAACTATTGAACAGAAAAAGAGCGGGAGACCGGGGTCTCTGAGCGCATACGAGATAGCAGGTCTTTGGAGACGGTCCATGCGGATA
asic1a_Frag1155-1314_insertion1299	TAGGAGCGGGATCGTCTCCTAGTATGGTCAAAATTCCAAGTAAAGCCAGTGCAAAATACTTGGCTAAGAAGTTCAACAAAAGCGAGCAGTACATCGGCGAGAACATTTTGGTTCTCGACATATTCTTCGAAGTCCTGAACTACGAAACTATTGAACAGAAAAAGGCAAGCGGGAGACCGGGGTCTCTGAGCTACGAGATAGCAGGTCTTTGGAGACGGTCCATGCGGATA
asic1a_Frag1155-1314_insertion1302	TAGGAGCGGGATCGTCTCCTAGTATGGTCAAAATTCCAAGTAAAGCCAGTGCAAAATACTTGGCTAAGAAGTTCAACAAAAGCGAGCAGTACATCGGCGAGAACATTTTGGTTCTCGACATATTCTTCGAAGTCCTGAACTACGAAACTATTGAACAGAAAAAGGCATACAGCGGGAGACCGGGGTCTCTGAGCGAGATAGCAGGTCTTTGGAGACGGTCCATGCGGATA
asic1a_Frag1155-1314_insertion1305	TAGGAGCGGGATCGTCTCCTAGTATGGTCAAAATTCCAAGTAAAGCCAGTGCAAAATACTTGGCTAAGAAGTTCAACAAAAGCGAGCAGTACATCGGCGAGAACATTTTGGTTCTCGACATATTCTTCGAAGTCCTGAACTACGAAACTATTGAACAGAAAAAGGCATACGAGAGCGGGAGACCGGGGTCTCTGAGCATAGCAGGTCTTTGGAGACGGTCCATGCGGATA
asic1a_Frag1155-1314_insertion1308	TAGGAGCGGGATCGTCTCCTAGTATGGTCAAAATTCCAAGTAAAGCCAGTGCAAAATACTTGGCTAAGAAGTTCAACAAAAGCGAGCAGTACATCGGCGAGAACATTTTGGTTCTCGACATATTCTTCGAAGTCCTGAACTACGAAACTATTGAACAGAAAAAGGCATACGAGATAAGCGGGAGACCGGGGTCTCTGAGCGCAGGTCTTTGGAGACGGTCCATGCGGATA
asic1a_Frag1155-1314_insertion1311	TAGGAGCGGGATCGTCTCCTAGTATGGTCAAAATTCCAAGTAAAGCCAGTGCAAAATACTTGGCTAAGAAGTTCAACAAAAGCGAGCAGTACATCGGCGAGAACATTTTGGTTCTCGACATATTCTTCGAAGTCCTGAACTACGAAACTATTGAACAGAAAAAGGCATACGAGATAGCAAGCGGGAGACCGGGGTCTCTGAGCGGTCTTTGGAGACGGTCCATGCGGATA
asic1a_Frag1155-1314_insertion1314	TAGGAGCGGGATCGTCTCCTAGTATGGTCAAAATTCCAAGTAAAGCCAGTGCAAAATACTTGGCTAAGAAGTTCAACAAAAGCGAGCAGTACATCGGCGAGAACATTTTGGTTCTCGACATATTCTTCGAAGTCCTGAACTACGAAACTATTGAACAGAAAAAGGCATACGAGATAGCAGGTAGCGGGAGACCGGGGTCTCTGAGCCTTTGGAGACGGTCCATGCGGATA
asic1a_Frag1317-1476_insertion1317	ACCGAGTACTCCCGTCTCCTCTTAGCGGGAGACCGGGGTCTCTGAGCTTGGGAGACATAGGAGGGCAGATGGGGCTGTTTATAGGGGCTTCTATTCTGACTGTACTCGAACTGTTTGACTACGCTTATGAAGTCATTAAACACAAGCTGTGTCGCCGCGGGAAATGTCAGAAGGAGGCTAAGAGAAGCTCAGCCGATAAAGGCGTAGCTCGGAGACGAGACCGTGCTGAA
asic1a_Frag1317-1476_insertion1320	ACCGAGTACTCCCGTCTCCTCTTTGGAGCGGGAGACCGGGGTCTCTGAGCGAGACATAGGAGGGCAGATGGGGCTGTTTATAGGGGCTTCTATTCTGACTGTACTCGAACTGTTTGACTACGCTTATGAAGTCATTAAACACAAGCTGTGTCGCCGCGGGAAATGTCAGAAGGAGGCTAAGAGAAGCTCAGCCGATAAAGGCGTAGCTCGGAGACGAGACCGTGCTGAA
asic1a_Frag1317-1476_insertion1323	ACCGAGTACTCCCGTCTCCTCTTTGGGAGAGCGGGAGACCGGGGTCTCTGAGCACATAGGAGGGCAGATGGGGCTGTTTATAGGGGCTTCTATTCTGACTGTACTCGAACTGTTTGACTACGCTTATGAAGTCATTAAACACAAGCTGTGTCGCCGCGGGAAATGTCAGAAGGAGGCTAAGAGAAGCTCAGCCGATAAAGGCGTAGCTCGGAGACGAGACCGTGCTGAA
asic1a_Frag1317-1476_insertion1326	ACCGAGTACTCCCGTCTCCTCTTTGGGAGACAAGCGGGAGACCGGGGTCTCTGAGCTAGGAGGGCAGATGGGGCTGTTTATAGGGGCTTCTATTCTGACTGTACTCGAACTGTTTGACTACGCTTATGAAGTCATTAAACACAAGCTGTGTCGCCGCGGGAAATGTCAGAAGGAGGCTAAGAGAAGCTCAGCCGATAAAGGCGTAGCTCGGAGACGAGACCGTGCTGAA
asic1a_Frag1317-1476_insertion1329	ACCGAGTACTCCCGTCTCCTCTTTGGGAGACATAGAGCGGGAGACCGGGGTCTCTGAGCGAGGGCAGATGGGGCTGTTTATAGGGGCTTCTATTCTGACTGTACTCGAACTGTTTGACTACGCTTATGAAGTCATTAAACACAAGCTGTGTCGCCGCGGGAAATGTCAGAAGGAGGCTAAGAGAAGCTCAGCCGATAAAGGCGTAGCTCGGAGACGAGACCGTGCTGAA
asic1a_Frag1317-1476_insertion1332	ACCGAGTACTCCCGTCTCCTCTTTGGGAGACATAGGAGAGCGGGAGACCGGGGTCTCTGAGCGGCAGATGGGGCTGTTTATAGGGGCTTCTATTCTGACTGTACTCGAACTGTTTGACTACGCTTATGAAGTCATTAAACACAAGCTGTGTCGCCGCGGGAAATGTCAGAAGGAGGCTAAGAGAAGCTCAGCCGATAAAGGCGTAGCTCGGAGACGAGACCGTGCTGAA
asic1a_Frag1317-1476_insertion1335	ACCGAGTACTCCCGTCTCCTCTTTGGGAGACATAGGAGGGCAGCGGGAGACCGGGGTCTCTGAGCAGATGGGGCTGTTTATAGGGGCTTCTATTCTGACTGTACTCGAACTGTTTGACTACGCTTATGAAGTCATTAAACACAAGCTGTGTCGCCGCGGGAAATGTCAGAAGGAGGCTAAGAGAAGCTCAGCCGATAAAGGCGTAGCTCGGAGACGAGACCGTGCTGAA
asic1a_Frag1317-1476_insertion1338	ACCGAGTACTCCCGTCTCCTCTTTGGGAGACATAGGAGGGCAGAAGCGGGAGACCGGGGTCTCTGAGCTGGGGCTGTTTATAGGGGCTTCTATTCTGACTGTACTCGAACTGTTTGACTACGCTTATGAAGTCATTAAACACAAGCTGTGTCGCCGCGGGAAATGTCAGAAGGAGGCTAAGAGAAGCTCAGCCGATAAAGGCGTAGCTCGGAGACGAGACCGTGCTGAA
asic1a_Frag1317-1476_insertion1341	ACCGAGTACTCCCGTCTCCTCTTTGGGAGACATAGGAGGGCAGATGGAGCGGGAGACCGGGGTCTCTGAGCGGCTGTTTATAGGGGCTTCTATTCTGACTGTACTCGAACTGTTTGACTACGCTTATGAAGTCATTAAACACAAGCTGTGTCGCCGCGGGAAATGTCAGAAGGAGGCTAAGAGAAGCTCAGCCGATAAAGGCGTAGCTCGGAGACGAGACCGTGCTGAA
asic1a_Frag1317-1476_insertion1344	ACCGAGTACTCCCGTCTCCTCTTTGGGAGACATAGGAGGGCAGATGGGGCAGCGGGAGACCGGGGTCTCTGAGCTGTTTATAGGGGCTTCTATTCTGACTGTACTCGAACTGTTTGACTACGCTTATGAAGTCATTAAACACAAGCTGTGTCGCCGCGGGAAATGTCAGAAGGAGGCTAAGAGAAGCTCAGCCGATAAAGGCGTAGCTCGGAGACGAGACCGTGCTGAA
asic1a_Frag1317-1476_insertion1347	ACCGAGTACTCCCGTCTCCTCTTTGGGAGACATAGGAGGGCAGATGGGGCTGTAGCGGGAGACCGGGGTCTCTGAGCTTATAGGGGCTTCTATTCTGACTGTACTCGAACTGTTTGACTACGCTTATGAAGTCATTAAACACAAGCTGTGTCGCCGCGGGAAATGTCAGAAGGAGGCTAAGAGAAGCTCAGCCGATAAAGGCGTAGCTCGGAGACGAGACCGTGCTGAA
asic1a_Frag1317-1476_insertion1350	ACCGAGTACTCCCGTCTCCTCTTTGGGAGACATAGGAGGGCAGATGGGGCTGTTTAAGCGGGAGACCGGGGTCTCTGAGCTAGGGGCTTCTATTCTGACTGTACTCGAACTGTTTGACTACGCTTATGAAGTCATTAAACACAAGCTGTGTCGCCGCGGGAAATGTCAGAAGGAGGCTAAGAGAAGCTCAGCCGATAAAGGCGTAGCTCGGAGACGAGACCGTGCTGAA
asic1a_Frag1317-1476_insertion1353	ACCGAGTACTCCCGTCTCCTCTTTGGGAGACATAGGAGGGCAGATGGGGCTGTTTATAGAGCGGGAGACCGGGGTCTCTGAGCGGGCTTCTATTCTGACTGTACTCGAACTGTTTGACTACGCTTATGAAGTCATTAAACACAAGCTGTGTCGCCGCGGGAAATGTCAGAAGGAGGCTAAGAGAAGCTCAGCCGATAAAGGCGTAGCTCGGAGACGAGACCGTGCTGAA
asic1a_Frag1317-1476_insertion1356	ACCGAGTACTCCCGTCTCCTCTTTGGGAGACATAGGAGGGCAGATGGGGCTGTTTATAGGGGAGCGGGAGACCGGGGTCTCTGAGCCTTCTATTCTGACTGTACTCGAACTGTTTGACTACGCTTATGAAGTCATTAAACACAAGCTGTGTCGCCGCGGGAAATGTCAGAAGGAGGCTAAGAGAAGCTCAGCCGATAAAGGCGTAGCTCGGAGACGAGACCGTGCTGAA
asic1a_Frag1317-1476_insertion1359	ACCGAGTACTCCCGTCTCCTCTTTGGGAGACATAGGAGGGCAGATGGGGCTGTTTATAGGGGCTTAGCGGGAGACCGGGGTCTCTGAGCCTATTCTGACTGTACTCGAACTGTTTGACTACGCTTATGAAGTCATTAAACACAAGCTGTGTCGCCGCGGGAAATGTCAGAAGGAGGCTAAGAGAAGCTCAGCCGATAAAGGCGTAGCTCGGAGACGAGACCGTGCTGAA
asic1a_Frag1317-1476_insertion1362	ACCGAGTACTCCCGTCTCCTCTTTGGGAGACATAGGAGGGCAGATGGGGCTGTTTATAGGGGCTTCTAAGCGGGAGACCGGGGTCTCTGAGCTTCTGACTGTACTCGAACTGTTTGACTACGCTTATGAAGTCATTAAACACAAGCTGTGTCGCCGCGGGAAATGTCAGAAGGAGGCTAAGAGAAGCTCAGCCGATAAAGGCGTAGCTCGGAGACGAGACCGTGCTGAA
asic1a_Frag1317-1476_insertion1365	ACCGAGTACTCCCGTCTCCTCTTTGGGAGACATAGGAGGGCAGATGGGGCTGTTTATAGGGGCTTCTATTCAGCGGGAGACCGGGGTCTCTGAGCTGACTGTACTCGAACTGTTTGACTACGCTTATGAAGTCATTAAACACAAGCTGTGTCGCCGCGGGAAATGTCAGAAGGAGGCTAAGAGAAGCTCAGCCGATAAAGGCGTAGCTCGGAGACGAGACCGTGCTGAA
asic1a_Frag1317-1476_insertion1368	ACCGAGTACTCCCGTCTCCTCTTTGGGAGACATAGGAGGGCAGATGGGGCTGTTTATAGGGGCTTCTATTCTGAAGCGGGAGACCGGGGTCTCTGAGCCTGTACTCGAACTGTTTGACTACGCTTATGAAGTCATTAAACACAAGCTGTGTCGCCGCGGGAAATGTCAGAAGGAGGCTAAGAGAAGCTCAGCCGATAAAGGCGTAGCTCGGAGACGAGACCGTGCTGAA
asic1a_Frag1317-1476_insertion1371	ACCGAGTACTCCCGTCTCCTCTTTGGGAGACATAGGAGGGCAGATGGGGCTGTTTATAGGGGCTTCTATTCTGACTGAGCGGGAGACCGGGGTCTCTGAGCTACTCGAACTGTTTGACTACGCTTATGAAGTCATTAAACACAAGCTGTGTCGCCGCGGGAAATGTCAGAAGGAGGCTAAGAGAAGCTCAGCCGATAAAGGCGTAGCTCGGAGACGAGACCGTGCTGAA
asic1a_Frag1317-1476_insertion1374	ACCGAGTACTCCCGTCTCCTCTTTGGGAGACATAGGAGGGCAGATGGGGCTGTTTATAGGGGCTTCTATTCTGACTGTACAGCGGGAGACCGGGGTCTCTGAGCTCGAACTGTTTGACTACGCTTATGAAGTCATTAAACACAAGCTGTGTCGCCGCGGGAAATGTCAGAAGGAGGCTAAGAGAAGCTCAGCCGATAAAGGCGTAGCTCGGAGACGAGACCGTGCTGAA
asic1a_Frag1317-1476_insertion1377	ACCGAGTACTCCCGTCTCCTCTTTGGGAGACATAGGAGGGCAGATGGGGCTGTTTATAGGGGCTTCTATTCTGACTGTACTCGAGCGGGAGACCGGGGTCTCTGAGCAACTGTTTGACTACGCTTATGAAGTCATTAAACACAAGCTGTGTCGCCGCGGGAAATGTCAGAAGGAGGCTAAGAGAAGCTCAGCCGATAAAGGCGTAGCTCGGAGACGAGACCGTGCTGAA
asic1a_Frag1317-1476_insertion1380	ACCGAGTACTCCCGTCTCCTCTTTGGGAGACATAGGAGGGCAGATGGGGCTGTTTATAGGGGCTTCTATTCTGACTGTACTCGAACAGCGGGAGACCGGGGTCTCTGAGCTGTTTGACTACGCTTATGAAGTCATTAAACACAAGCTGTGTCGCCGCGGGAAATGTCAGAAGGAGGCTAAGAGAAGCTCAGCCGATAAAGGCGTAGCTCGGAGACGAGACCGTGCTGAA
asic1a_Frag1317-1476_insertion1383	ACCGAGTACTCCCGTCTCCTCTTTGGGAGACATAGGAGGGCAGATGGGGCTGTTTATAGGGGCTTCTATTCTGACTGTACTCGAACTGTAGCGGGAGACCGGGGTCTCTGAGCTTGACTACGCTTATGAAGTCATTAAACACAAGCTGTGTCGCCGCGGGAAATGTCAGAAGGAGGCTAAGAGAAGCTCAGCCGATAAAGGCGTAGCTCGGAGACGAGACCGTGCTGAA
asic1a_Frag1317-1476_insertion1386	ACCGAGTACTCCCGTCTCCTCTTTGGGAGACATAGGAGGGCAGATGGGGCTGTTTATAGGGGCTTCTATTCTGACTGTACTCGAACTGTTTGAGCGGGAGACCGGGGTCTCTGAGCACTACGCTTATGAAGTCATTAAACACAAGCTGTGTCGCCGCGGGAAATGTCAGAAGGAGGCTAAGAGAAGCTCAGCCGATAAAGGCGTAGCTCGGAGACGAGACCGTGCTGAA
asic1a_Frag1317-1476_insertion1389	ACCGAGTACTCCCGTCTCCTCTTTGGGAGACATAGGAGGGCAGATGGGGCTGTTTATAGGGGCTTCTATTCTGACTGTACTCGAACTGTTTGACTAGCGGGAGACCGGGGTCTCTGAGCACGCTTATGAAGTCATTAAACACAAGCTGTGTCGCCGCGGGAAATGTCAGAAGGAGGCTAAGAGAAGCTCAGCCGATAAAGGCGTAGCTCGGAGACGAGACCGTGCTGAA
asic1a_Frag1317-1476_insertion1392	ACCGAGTACTCCCGTCTCCTCTTTGGGAGACATAGGAGGGCAGATGGGGCTGTTTATAGGGGCTTCTATTCTGACTGTACTCGAACTGTTTGACTACGAGCGGGAGACCGGGGTCTCTGAGCCTTATGAAGTCATTAAACACAAGCTGTGTCGCCGCGGGAAATGTCAGAAGGAGGCTAAGAGAAGCTCAGCCGATAAAGGCGTAGCTCGGAGACGAGACCGTGCTGAA
asic1a_Frag1317-1476_insertion1395	ACCGAGTACTCCCGTCTCCTCTTTGGGAGACATAGGAGGGCAGATGGGGCTGTTTATAGGGGCTTCTATTCTGACTGTACTCGAACTGTTTGACTACGCTTAGCGGGAGACCGGGGTCTCTGAGCATGAAGTCATTAAACACAAGCTGTGTCGCCGCGGGAAATGTCAGAAGGAGGCTAAGAGAAGCTCAGCCGATAAAGGCGTAGCTCGGAGACGAGACCGTGCTGAA
asic1a_Frag1317-1476_insertion1398	ACCGAGTACTCCCGTCTCCTCTTTGGGAGACATAGGAGGGCAGATGGGGCTGTTTATAGGGGCTTCTATTCTGACTGTACTCGAACTGTTTGACTACGCTTATGAGCGGGAGACCGGGGTCTCTGAGCAAGTCATTAAACACAAGCTGTGTCGCCGCGGGAAATGTCAGAAGGAGGCTAAGAGAAGCTCAGCCGATAAAGGCGTAGCTCGGAGACGAGACCGTGCTGAA
asic1a_Frag1317-1476_insertion1401	ACCGAGTACTCCCGTCTCCTCTTTGGGAGACATAGGAGGGCAGATGGGGCTGTTTATAGGGGCTTCTATTCTGACTGTACTCGAACTGTTTGACTACGCTTATGAAGAGCGGGAGACCGGGGTCTCTGAGCTCATTAAACACAAGCTGTGTCGCCGCGGGAAATGTCAGAAGGAGGCTAAGAGAAGCTCAGCCGATAAAGGCGTAGCTCGGAGACGAGACCGTGCTGAA
asic1a_Frag1317-1476_insertion1404	ACCGAGTACTCCCGTCTCCTCTTTGGGAGACATAGGAGGGCAGATGGGGCTGTTTATAGGGGCTTCTATTCTGACTGTACTCGAACTGTTTGACTACGCTTATGAAGTCAAGCGGGAGACCGGGGTCTCTGAGCTTAAACACAAGCTGTGTCGCCGCGGGAAATGTCAGAAGGAGGCTAAGAGAAGCTCAGCCGATAAAGGCGTAGCTCGGAGACGAGACCGTGCTGAA
asic1a_Frag1317-1476_insertion1407	ACCGAGTACTCCCGTCTCCTCTTTGGGAGACATAGGAGGGCAGATGGGGCTGTTTATAGGGGCTTCTATTCTGACTGTACTCGAACTGTTTGACTACGCTTATGAAGTCATTAAGCGGGAGACCGGGGTCTCTGAGCAACACAAGCTGTGTCGCCGCGGGAAATGTCAGAAGGAGGCTAAGAGAAGCTCAGCCGATAAAGGCGTAGCTCGGAGACGAGACCGTGCTGAA
asic1a_Frag1317-1476_insertion1410	ACCGAGTACTCCCGTCTCCTCTTTGGGAGACATAGGAGGGCAGATGGGGCTGTTTATAGGGGCTTCTATTCTGACTGTACTCGAACTGTTTGACTACGCTTATGAAGTCATTAAACAGCGGGAGACCGGGGTCTCTGAGCACAAGCTGTGTCGCCGCGGGAAATGTCAGAAGGAGGCTAAGAGAAGCTCAGCCGATAAAGGCGTAGCTCGGAGACGAGACCGTGCTGAA
asic1a_Frag1317-1476_insertion1413	ACCGAGTACTCCCGTCTCCTCTTTGGGAGACATAGGAGGGCAGATGGGGCTGTTTATAGGGGCTTCTATTCTGACTGTACTCGAACTGTTTGACTACGCTTATGAAGTCATTAAACACAAGCGGGAGACCGGGGTCTCTGAGCAGCTGTGTCGCCGCGGGAAATGTCAGAAGGAGGCTAAGAGAAGCTCAGCCGATAAAGGCGTAGCTCGGAGACGAGACCGTGCTGAA
asic1a_Frag1317-1476_insertion1416	ACCGAGTACTCCCGTCTCCTCTTTGGGAGACATAGGAGGGCAGATGGGGCTGTTTATAGGGGCTTCTATTCTGACTGTACTCGAACTGTTTGACTACGCTTATGAAGTCATTAAACACAAGCAGCGGGAGACCGGGGTCTCTGAGCTGTGTCGCCGCGGGAAATGTCAGAAGGAGGCTAAGAGAAGCTCAGCCGATAAAGGCGTAGCTCGGAGACGAGACCGTGCTGAA
asic1a_Frag1317-1476_insertion1419	ACCGAGTACTCCCGTCTCCTCTTTGGGAGACATAGGAGGGCAGATGGGGCTGTTTATAGGGGCTTCTATTCTGACTGTACTCGAACTGTTTGACTACGCTTATGAAGTCATTAAACACAAGCTGTAGCGGGAGACCGGGGTCTCTGAGCGTCGCCGCGGGAAATGTCAGAAGGAGGCTAAGAGAAGCTCAGCCGATAAAGGCGTAGCTCGGAGACGAGACCGTGCTGAA
asic1a_Frag1317-1476_insertion1422	ACCGAGTACTCCCGTCTCCTCTTTGGGAGACATAGGAGGGCAGATGGGGCTGTTTATAGGGGCTTCTATTCTGACTGTACTCGAACTGTTTGACTACGCTTATGAAGTCATTAAACACAAGCTGTGTCAGCGGGAGACCGGGGTCTCTGAGCGCCGCGGGAAATGTCAGAAGGAGGCTAAGAGAAGCTCAGCCGATAAAGGCGTAGCTCGGAGACGAGACCGTGCTGAA
asic1a_Frag1317-1476_insertion1425	ACCGAGTACTCCCGTCTCCTCTTTGGGAGACATAGGAGGGCAGATGGGGCTGTTTATAGGGGCTTCTATTCTGACTGTACTCGAACTGTTTGACTACGCTTATGAAGTCATTAAACACAAGCTGTGTCGCCAGCGGGAGACCGGGGTCTCTGAGCGCGGGAAATGTCAGAAGGAGGCTAAGAGAAGCTCAGCCGATAAAGGCGTAGCTCGGAGACGAGACCGTGCTGAA
asic1a_Frag1317-1476_insertion1428	ACCGAGTACTCCCGTCTCCTCTTTGGGAGACATAGGAGGGCAGATGGGGCTGTTTATAGGGGCTTCTATTCTGACTGTACTCGAACTGTTTGACTACGCTTATGAAGTCATTAAACACAAGCTGTGTCGCCGCGAGCGGGAGACCGGGGTCTCTGAGCGGAAATGTCAGAAGGAGGCTAAGAGAAGCTCAGCCGATAAAGGCGTAGCTCGGAGACGAGACCGTGCTGAA
asic1a_Frag1317-1476_insertion1431	ACCGAGTACTCCCGTCTCCTCTTTGGGAGACATAGGAGGGCAGATGGGGCTGTTTATAGGGGCTTCTATTCTGACTGTACTCGAACTGTTTGACTACGCTTATGAAGTCATTAAACACAAGCTGTGTCGCCGCGGGAAGCGGGAGACCGGGGTCTCTGAGCAATGTCAGAAGGAGGCTAAGAGAAGCTCAGCCGATAAAGGCGTAGCTCGGAGACGAGACCGTGCTGAA
asic1a_Frag1317-1476_insertion1434	ACCGAGTACTCCCGTCTCCTCTTTGGGAGACATAGGAGGGCAGATGGGGCTGTTTATAGGGGCTTCTATTCTGACTGTACTCGAACTGTTTGACTACGCTTATGAAGTCATTAAACACAAGCTGTGTCGCCGCGGGAAATAGCGGGAGACCGGGGTCTCTGAGCGTCAGAAGGAGGCTAAGAGAAGCTCAGCCGATAAAGGCGTAGCTCGGAGACGAGACCGTGCTGAA
asic1a_Frag1317-1476_insertion1437	ACCGAGTACTCCCGTCTCCTCTTTGGGAGACATAGGAGGGCAGATGGGGCTGTTTATAGGGGCTTCTATTCTGACTGTACTCGAACTGTTTGACTACGCTTATGAAGTCATTAAACACAAGCTGTGTCGCCGCGGGAAATGTCAGCGGGAGACCGGGGTCTCTGAGCAGAAGGAGGCTAAGAGAAGCTCAGCCGATAAAGGCGTAGCTCGGAGACGAGACCGTGCTGAA
asic1a_Frag1317-1476_insertion1440	ACCGAGTACTCCCGTCTCCTCTTTGGGAGACATAGGAGGGCAGATGGGGCTGTTTATAGGGGCTTCTATTCTGACTGTACTCGAACTGTTTGACTACGCTTATGAAGTCATTAAACACAAGCTGTGTCGCCGCGGGAAATGTCAGAAGCGGGAGACCGGGGTCTCTGAGCAGGAGGCTAAGAGAAGCTCAGCCGATAAAGGCGTAGCTCGGAGACGAGACCGTGCTGAA
asic1a_Frag1317-1476_insertion1443	ACCGAGTACTCCCGTCTCCTCTTTGGGAGACATAGGAGGGCAGATGGGGCTGTTTATAGGGGCTTCTATTCTGACTGTACTCGAACTGTTTGACTACGCTTATGAAGTCATTAAACACAAGCTGTGTCGCCGCGGGAAATGTCAGAAGGAGCGGGAGACCGGGGTCTCTGAGCAGGCTAAGAGAAGCTCAGCCGATAAAGGCGTAGCTCGGAGACGAGACCGTGCTGAA
asic1a_Frag1317-1476_insertion1446	ACCGAGTACTCCCGTCTCCTCTTTGGGAGACATAGGAGGGCAGATGGGGCTGTTTATAGGGGCTTCTATTCTGACTGTACTCGAACTGTTTGACTACGCTTATGAAGTCATTAAACACAAGCTGTGTCGCCGCGGGAAATGTCAGAAGGAGGAGCGGGAGACCGGGGTCTCTGAGCCTAAGAGAAGCTCAGCCGATAAAGGCGTAGCTCGGAGACGAGACCGTGCTGAA
asic1a_Frag1317-1476_insertion1449	ACCGAGTACTCCCGTCTCCTCTTTGGGAGACATAGGAGGGCAGATGGGGCTGTTTATAGGGGCTTCTATTCTGACTGTACTCGAACTGTTTGACTACGCTTATGAAGTCATTAAACACAAGCTGTGTCGCCGCGGGAAATGTCAGAAGGAGGCTAAGCGGGAGACCGGGGTCTCTGAGCAGAGAAGCTCAGCCGATAAAGGCGTAGCTCGGAGACGAGACCGTGCTGAA
asic1a_Frag1317-1476_insertion1452	ACCGAGTACTCCCGTCTCCTCTTTGGGAGACATAGGAGGGCAGATGGGGCTGTTTATAGGGGCTTCTATTCTGACTGTACTCGAACTGTTTGACTACGCTTATGAAGTCATTAAACACAAGCTGTGTCGCCGCGGGAAATGTCAGAAGGAGGCTAAGAAGCGGGAGACCGGGGTCTCTGAGCGAAGCTCAGCCGATAAAGGCGTAGCTCGGAGACGAGACCGTGCTGAA
asic1a_Frag1317-1476_insertion1455	ACCGAGTACTCCCGTCTCCTCTTTGGGAGACATAGGAGGGCAGATGGGGCTGTTTATAGGGGCTTCTATTCTGACTGTACTCGAACTGTTTGACTACGCTTATGAAGTCATTAAACACAAGCTGTGTCGCCGCGGGAAATGTCAGAAGGAGGCTAAGAGAAGCGGGAGACCGGGGTCTCTGAGCAGCTCAGCCGATAAAGGCGTAGCTCGGAGACGAGACCGTGCTGAA
asic1a_Frag1317-1476_insertion1458	ACCGAGTACTCCCGTCTCCTCTTTGGGAGACATAGGAGGGCAGATGGGGCTGTTTATAGGGGCTTCTATTCTGACTGTACTCGAACTGTTTGACTACGCTTATGAAGTCATTAAACACAAGCTGTGTCGCCGCGGGAAATGTCAGAAGGAGGCTAAGAGAAGCAGCGGGAGACCGGGGTCTCTGAGCTCAGCCGATAAAGGCGTAGCTCGGAGACGAGACCGTGCTGAA
asic1a_Frag1317-1476_insertion1461	ACCGAGTACTCCCGTCTCCTCTTTGGGAGACATAGGAGGGCAGATGGGGCTGTTTATAGGGGCTTCTATTCTGACTGTACTCGAACTGTTTGACTACGCTTATGAAGTCATTAAACACAAGCTGTGTCGCCGCGGGAAATGTCAGAAGGAGGCTAAGAGAAGCTCAAGCGGGAGACCGGGGTCTCTGAGCGCCGATAAAGGCGTAGCTCGGAGACGAGACCGTGCTGAA
asic1a_Frag1317-1476_insertion1464	ACCGAGTACTCCCGTCTCCTCTTTGGGAGACATAGGAGGGCAGATGGGGCTGTTTATAGGGGCTTCTATTCTGACTGTACTCGAACTGTTTGACTACGCTTATGAAGTCATTAAACACAAGCTGTGTCGCCGCGGGAAATGTCAGAAGGAGGCTAAGAGAAGCTCAGCCAGCGGGAGACCGGGGTCTCTGAGCGATAAAGGCGTAGCTCGGAGACGAGACCGTGCTGAA
asic1a_Frag1317-1476_insertion1467	ACCGAGTACTCCCGTCTCCTCTTTGGGAGACATAGGAGGGCAGATGGGGCTGTTTATAGGGGCTTCTATTCTGACTGTACTCGAACTGTTTGACTACGCTTATGAAGTCATTAAACACAAGCTGTGTCGCCGCGGGAAATGTCAGAAGGAGGCTAAGAGAAGCTCAGCCGATAGCGGGAGACCGGGGTCTCTGAGCAAAGGCGTAGCTCGGAGACGAGACCGTGCTGAA
asic1a_Frag1317-1476_insertion1470	ACCGAGTACTCCCGTCTCCTCTTTGGGAGACATAGGAGGGCAGATGGGGCTGTTTATAGGGGCTTCTATTCTGACTGTACTCGAACTGTTTGACTACGCTTATGAAGTCATTAAACACAAGCTGTGTCGCCGCGGGAAATGTCAGAAGGAGGCTAAGAGAAGCTCAGCCGATAAAGAGCGGGAGACCGGGGTCTCTGAGCGCGTAGCTCGGAGACGAGACCGTGCTGAA

asic1a_Frag1317-1476_insertion1473	ACCGAGTACTCCCGTCTCCTCTTTTGGGAGACATAGGAGGGCAGATGGGGCTGTTTATAGGGGCTTCTATTCTGACTGTACTCGAACTGTTTGACTACGCTTATGAAGTCATTAAACACAAGCTGTGTCGCCGCGGGAAATGTCAGAAGGAGGCTAAGAGAAGCTCAGCCGATAAAGGCAGCGGGAGACCGGGGTCTCTGAGCGTAGCTCGGAGACGAGACCGTGCTGAA
asic1a_Frag1317-1476_insertion1476	ACCGAGTACTCCCGTCTCCTCTTTTGGGAGACATAGGAGGGCAGATGGGGCTGTTTATAGGGGCTTCTATTCTGACTGTACTCGAACTGTTTGACTACGCTTATGAAGTCATTAAACACAAGCTGTGTCGCCGCGGGAAATGTCAGAAGGAGGCTAAGAGAAGCTCAGCCGATAAAGGCGTAAGCGGGAGACCGGGGTCTCTGAGCGCTCGGAGACGAGACCGTGCTGAA
asic1a_Frag1479-1638_insertion1479	GAGTCTGTACCCCGTCTCCAGCTAGCGGGAGACCGGGGTCTCTGAGCCTGTCTTTGGATGATGTAAAACGCCATAATCCTTGCGAATCTCTTCGCGGGCACCCAGCCGGCATGACTTACGCCGCAAACATCCTGCCCCATCATCCCGCACGAGGCACCTTCGAAGATTTTACATGTGCTGCCAGCTCTGCTGTGAATGGTTCTGGAGCAAGGAGACGTCTCGCGCCTATC
asic1a_Frag1479-1638_insertion1482	GAGTCTGTACCCCGTCTCCAGCTCTGAGCGGGAGACCGGGGTCTCTGAGCTCTTTGGATGATGTAAAACGCCATAATCCTTGCGAATCTCTTCGCGGGCACCCAGCCGGCATGACTTACGCCGCAAACATCCTGCCCCATCATCCCGCACGAGGCACCTTCGAAGATTTTACATGTGCTGCCAGCTCTGCTGTGAATGGTTCTGGAGCAAGGAGACGTCTCGCGCCTATC
asic1a_Frag1479-1638_insertion1485	GAGTCTGTACCCCGTCTCCAGCTCTGTCTAGCGGGAGACCGGGGTCTCTGAGCTTGGATGATGTAAAACGCCATAATCCTTGCGAATCTCTTCGCGGGCACCCAGCCGGCATGACTTACGCCGCAAACATCCTGCCCCATCATCCCGCACGAGGCACCTTCGAAGATTTTACATGTGCTGCCAGCTCTGCTGTGAATGGTTCTGGAGCAAGGAGACGTCTCGCGCCTATC
asic1a_Frag1479-1638_insertion1488	GAGTCTGTACCCCGTCTCCAGCTCTGTCTTTGAGCGGGAGACCGGGGTCTCTGAGCGATGATGTAAAACGCCATAATCCTTGCGAATCTCTTCGCGGGCACCCAGCCGGCATGACTTACGCCGCAAACATCCTGCCCCATCATCCCGCACGAGGCACCTTCGAAGATTTTACATGTGCTGCCAGCTCTGCTGTGAATGGTTCTGGAGCAAGGAGACGTCTCGCGCCTATC
asic1a_Frag1479-1638_insertion1491	GAGTCTGTACCCCGTCTCCAGCTCTGTCTTTGGATAGCGGGAGACCGGGGTCTCTGAGCGATGTAAAACGCCATAATCCTTGCGAATCTCTTCGCGGGCACCCAGCCGGCATGACTTACGCCGCAAACATCCTGCCCCATCATCCCGCACGAGGCACCTTCGAAGATTTTACATGTGCTGCCAGCTCTGCTGTGAATGGTTCTGGAGCAAGGAGACGTCTCGCGCCTATC
asic1a_Frag1479-1638_insertion1494	GAGTCTGTACCCCGTCTCCAGCTCTGTCTTTGGATGATAGCGGGAGACCGGGGTCTCTGAGCGTAAAACGCCATAATCCTTGCGAATCTCTTCGCGGGCACCCAGCCGGCATGACTTACGCCGCAAACATCCTGCCCCATCATCCCGCACGAGGCACCTTCGAAGATTTTACATGTGCTGCCAGCTCTGCTGTGAATGGTTCTGGAGCAAGGAGACGTCTCGCGCCTATC
asic1a_Frag1479-1638_insertion1497	GAGTCTGTACCCCGTCTCCAGCTCTGTCTTTGGATGATGTAAGCGGGAGACCGGGGTCTCTGAGCAAACGCCATAATCCTTGCGAATCTCTTCGCGGGCACCCAGCCGGCATGACTTACGCCGCAAACATCCTGCCCCATCATCCCGCACGAGGCACCTTCGAAGATTTTACATGTGCTGCCAGCTCTGCTGTGAATGGTTCTGGAGCAAGGAGACGTCTCGCGCCTATC
asic1a_Frag1479-1638_insertion1500	GAGTCTGTACCCCGTCTCCAGCTCTGTCTTTGGATGATGTAAAAAGCGGGAGACCGGGGTCTCTGAGCCGCCATAATCCTTGCGAATCTCTTCGCGGGCACCCAGCCGGCATGACTTACGCCGCAAACATCCTGCCCCATCATCCCGCACGAGGCACCTTCGAAGATTTTACATGTGCTGCCAGCTCTGCTGTGAATGGTTCTGGAGCAAGGAGACGTCTCGCGCCTATC
asic1a_Frag1479-1638_insertion1503	GAGTCTGTACCCCGTCTCCAGCTCTGTCTTTGGATGATGTAAAACGCAGCGGGAGACCGGGGTCTCTGAGCCATAATCCTTGCGAATCTCTTCGCGGGCACCCAGCCGGCATGACTTACGCCGCAAACATCCTGCCCCATCATCCCGCACGAGGCACCTTCGAAGATTTTACATGTGCTGCCAGCTCTGCTGTGAATGGTTCTGGAGCAAGGAGACGTCTCGCGCCTATC
asic1a_Frag1479-1638_insertion1506	GAGTCTGTACCCCGTCTCCAGCTCTGTCTTTGGATGATGTAAAACGCCATAGCGGGAGACCGGGGTCTCTGAGCAATCCTTGCGAATCTCTTCGCGGGCACCCAGCCGGCATGACTTACGCCGCAAACATCCTGCCCCATCATCCCGCACGAGGCACCTTCGAAGATTTTACATGTGCTGCCAGCTCTGCTGTGAATGGTTCTGGAGCAAGGAGACGTCTCGCGCCTATC
asic1a_Frag1479-1638_insertion1509	GAGTCTGTACCCCGTCTCCAGCTCTGTCTTTGGATGATGTAAAACGCCATAATAGCGGGAGACCGGGGTCTCTGAGCCCTTGCGAATCTCTTCGCGGGCACCCAGCCGGCATGACTTACGCCGCAAACATCCTGCCCCATCATCCCGCACGAGGCACCTTCGAAGATTTTACATGTGCTGCCAGCTCTGCTGTGAATGGTTCTGGAGCAAGGAGACGTCTCGCGCCTATC
asic1a_Frag1479-1638_insertion1512	GAGTCTGTACCCCGTCTCCAGCTCTGTCTTTGGATGATGTAAAACGCCATAATCCTAGCGGGAGACCGGGGTCTCTGAGCTGCGAATCTCTTCGCGGGCACCCAGCCGGCATGACTTACGCCGCAAACATCCTGCCCCATCATCCCGCACGAGGCACCTTCGAAGATTTTACATGTGCTGCCAGCTCTGCTGTGAATGGTTCTGGAGCAAGGAGACGTCTCGCGCCTATC
asic1a_Frag1479-1638_insertion1515	GAGTCTGTACCCCGTCTCCAGCTCTGTCTTTGGATGATGTAAAACGCCATAATCCTTGCAGCGGGAGACCGGGGTCTCTGAGCGAATCTCTTCGCGGGCACCCAGCCGGCATGACTTACGCCGCAAACATCCTGCCCCATCATCCCGCACGAGGCACCTTCGAAGATTTTACATGTGCTGCCAGCTCTGCTGTGAATGGTTCTGGAGCAAGGAGACGTCTCGCGCCTATC
asic1a_Frag1479-1638_insertion1518	GAGTCTGTACCCCGTCTCCAGCTCTGTCTTTGGATGATGTAAAACGCCATAATCCTTGCGAAAGCGGGAGACCGGGGTCTCTGAGCTCTCTTCGCGGGCACCCAGCCGGCATGACTTACGCCGCAAACATCCTGCCCCATCATCCCGCACGAGGCACCTTCGAAGATTTTACATGTGCTGCCAGCTCTGCTGTGAATGGTTCTGGAGCAAGGAGACGTCTCGCGCCTATC
asic1a_Frag1479-1638_insertion1521	GAGTCTGTACCCCGTCTCCAGCTCTGTCTTTGGATGATGTAAAACGCCATAATCCTTGCGAATCTAGCGGGAGACCGGGGTCTCTGAGCCTTCGCGGGCACCCAGCCGGCATGACTTACGCCGCAAACATCCTGCCCCATCATCCCGCACGAGGCACCTTCGAAGATTTTACATGTGCTGCCAGCTCTGCTGTGAATGGTTCTGGAGCAAGGAGACGTCTCGCGCCTATC
asic1a_Frag1479-1638_insertion1524	GAGTCTGTACCCCGTCTCCAGCTCTGTCTTTGGATGATGTAAAACGCCATAATCCTTGCGAATCTCTTAGCGGGAGACCGGGGTCTCTGAGCCGCGGGCACCCAGCCGGCATGACTTACGCCGCAAACATCCTGCCCCATCATCCCGCACGAGGCACCTTCGAAGATTTTACATGTGCTGCCAGCTCTGCTGTGAATGGTTCTGGAGCAAGGAGACGTCTCGCGCCTATC
asic1a_Frag1479-1638_insertion1527	GAGTCTGTACCCCGTCTCCAGCTCTGTCTTTGGATGATGTAAAACGCCATAATCCTTGCGAATCTCTTCGCAGCGGGAGACCGGGGTCTCTGAGCGGGCACCCAGCCGGCATGACTTACGCCGCAAACATCCTGCCCCATCATCCCGCACGAGGCACCTTCGAAGATTTTACATGTGCTGCCAGCTCTGCTGTGAATGGTTCTGGAGCAAGGAGACGTCTCGCGCCTATC
asic1a_Frag1479-1638_insertion1530	GAGTCTGTACCCCGTCTCCAGCTCTGTCTTTGGATGATGTAAAACGCCATAATCCTTGCGAATCTCTTCGCGGGAGCGGGAGACCGGGGTCTCTGAGCCACCCAGCCGGCATGACTTACGCCGCAAACATCCTGCCCCATCATCCCGCACGAGGCACCTTCGAAGATTTTACATGTGCTGCCAGCTCTGCTGTGAATGGTTCTGGAGCAAGGAGACGTCTCGCGCCTATC
asic1a_Frag1479-1638_insertion1533	GAGTCTGTACCCCGTCTCCAGCTCTGTCTTTGGATGATGTAAAACGCCATAATCCTTGCGAATCTCTTCGCGGGCACAGCGGGAGACCGGGGTCTCTGAGCCCAGCCGGCATGACTTACGCCGCAAACATCCTGCCCCATCATCCCGCACGAGGCACCTTCGAAGATTTTACATGTGCTGCCAGCTCTGCTGTGAATGGTTCTGGAGCAAGGAGACGTCTCGCGCCTATC
asic1a_Frag1479-1638_insertion1536	GAGTCTGTACCCCGTCTCCAGCTCTGTCTTTGGATGATGTAAAACGCCATAATCCTTGCGAATCTCTTCGCGGGCACCCAAGCGGGAGACCGGGGTCTCTGAGCGCCGGCATGACTTACGCCGCAAACATCCTGCCCCATCATCCCGCACGAGGCACCTTCGAAGATTTTACATGTGCTGCCAGCTCTGCTGTGAATGGTTCTGGAGCAAGGAGACGTCTCGCGCCTATC
asic1a_Frag1479-1638_insertion1539	GAGTCTGTACCCCGTCTCCAGCTCTGTCTTTGGATGATGTAAAACGCCATAATCCTTGCGAATCTCTTCGCGGGCACCCAGCCAGCGGGAGACCGGGGTCTCTGAGCGGCATGACTTACGCCGCAAACATCCTGCCCCATCATCCCGCACGAGGCACCTTCGAAGATTTTACATGTGCTGCCAGCTCTGCTGTGAATGGTTCTGGAGCAAGGAGACGTCTCGCGCCTATC
asic1a_Frag1479-1638_insertion1542	GAGTCTGTACCCCGTCTCCAGCTCTGTCTTTGGATGATGTAAAACGCCATAATCCTTGCGAATCTCTTCGCGGGCACCCAGCCGGCAGCGGGAGACCGGGGTCTCTGAGCATGACTTACGCCGCAAACATCCTGCCCCATCATCCCGCACGAGGCACCTTCGAAGATTTTACATGTGCTGCCAGCTCTGCTGTGAATGGTTCTGGAGCAAGGAGACGTCTCGCGCCTATC
asic1a_Frag1479-1638_insertion1545	GAGTCTGTACCCCGTCTCCAGCTCTGTCTTTGGATGATGTAAAACGCCATAATCCTTGCGAATCTCTTCGCGGGCACCCAGCCGGCATGAGCGGGAGACCGGGGTCTCTGAGCACTTACGCCGCAAACATCCTGCCCCATCATCCCGCACGAGGCACCTTCGAAGATTTTACATGTGCTGCCAGCTCTGCTGTGAATGGTTCTGGAGCAAGGAGACGTCTCGCGCCTATC
asic1a_Frag1479-1638_insertion1548	GAGTCTGTACCCCGTCTCCAGCTCTGTCTTTGGATGATGTAAAACGCCATAATCCTTGCGAATCTCTTCGCGGGCACCCAGCCGGCATGACTAGCGGGAGACCGGGGTCTCTGAGCTACGCCGCAAACATCCTGCCCCATCATCCCGCACGAGGCACCTTCGAAGATTTTACATGTGCTGCCAGCTCTGCTGTGAATGGTTCTGGAGCAAGGAGACGTCTCGCGCCTATC
asic1a_Frag1479-1638_insertion1551	GAGTCTGTACCCCGTCTCCAGCTCTGTCTTTGGATGATGTAAAACGCCATAATCCTTGCGAATCTCTTCGCGGGCACCCAGCCGGCATGACTTACAGCGGGAGACCGGGGTCTCTGAGCGCCGCAAACATCCTGCCCCATCATCCCGCACGAGGCACCTTCGAAGATTTTACATGTGCTGCCAGCTCTGCTGTGAATGGTTCTGGAGCAAGGAGACGTCTCGCGCCTATC
asic1a_Frag1479-1638_insertion1554	GAGTCTGTACCCCGTCTCCAGCTCTGTCTTTGGATGATGTAAAACGCCATAATCCTTGCGAATCTCTTCGCGGGCACCCAGCCGGCATGACTTACGCCAGCGGGAGACCGGGGTCTCTGAGCGCAAACATCCTGCCCCATCATCCCGCACGAGGCACCTTCGAAGATTTTACATGTGCTGCCAGCTCTGCTGTGAATGGTTCTGGAGCAAGGAGACGTCTCGCGCCTATC
asic1a_Frag1479-1638_insertion1557	GAGTCTGTACCCCGTCTCCAGCTCTGTCTTTGGATGATGTAAAACGCCATAATCCTTGCGAATCTCTTCGCGGGCACCCAGCCGGCATGACTTACGCCGCAAGCGGGAGACCGGGGTCTCTGAGCAACATCCTGCCCCATCATCCCGCACGAGGCACCTTCGAAGATTTTACATGTGCTGCCAGCTCTGCTGTGAATGGTTCTGGAGCAAGGAGACGTCTCGCGCCTATC
asic1a_Frag1479-1638_insertion1560	GAGTCTGTACCCCGTCTCCAGCTCTGTCTTTGGATGATGTAAAACGCCATAATCCTTGCGAATCTCTTCGCGGGCACCCAGCCGGCATGACTTACGCCGCAAACAGCGGGAGACCGGGGTCTCTGAGCATCCTGCCCCATCATCCCGCACGAGGCACCTTCGAAGATTTTACATGTGCTGCCAGCTCTGCTGTGAATGGTTCTGGAGCAAGGAGACGTCTCGCGCCTATC
asic1a_Frag1479-1638_insertion1563	GAGTCTGTACCCCGTCTCCAGCTCTGTCTTTGGATGATGTAAAACGCCATAATCCTTGCGAATCTCTTCGCGGGCACCCAGCCGGCATGACTTACGCCGCAAACATCAGCGGGAGACCGGGGTCTCTGAGCCTGCCCCATCATCCCGCACGAGGCACCTTCGAAGATTTTACATGTGCTGCCAGCTCTGCTGTGAATGGTTCTGGAGCAAGGAGACGTCTCGCGCCTATC
asic1a_Frag1479-1638_insertion1566	GAGTCTGTACCCCGTCTCCAGCTCTGTCTTTGGATGATGTAAAACGCCATAATCCTTGCGAATCTCTTCGCGGGCACCCAGCCGGCATGACTTACGCCGCAAACATCCTGAGCGGGAGACCGGGGTCTCTGAGCCCCCATCATCCCGCACGAGGCACCTTCGAAGATTTTACATGTGCTGCCAGCTCTGCTGTGAATGGTTCTGGAGCAAGGAGACGTCTCGCGCCTATC
asic1a_Frag1479-1638_insertion1569	GAGTCTGTACCCCGTCTCCAGCTCTGTCTTTGGATGATGTAAAACGCCATAATCCTTGCGAATCTCTTCGCGGGCACCCAGCCGGCATGACTTACGCCGCAAACATCCTGCCCAGCGGGAGACCGGGGTCTCTGAGCCATCATCCCGCACGAGGCACCTTCGAAGATTTTACATGTGCTGCCAGCTCTGCTGTGAATGGTTCTGGAGCAAGGAGACGTCTCGCGCCTATC
asic1a_Frag1479-1638_insertion1572	GAGTCTGTACCCCGTCTCCAGCTCTGTCTTTGGATGATGTAAAACGCCATAATCCTTGCGAATCTCTTCGCGGGCACCCAGCCGGCATGACTTACGCCGCAAACATCCTGCCCCATAGCGGGAGACCGGGGTCTCTGAGCCATCCCGCACGAGGCACCTTCGAAGATTTTACATGTGCTGCCAGCTCTGCTGTGAATGGTTCTGGAGCAAGGAGACGTCTCGCGCCTATC
asic1a_Frag1479-1638_insertion1575	GAGTCTGTACCCCGTCTCCAGCTCTGTCTTTGGATGATGTAAAACGCCATAATCCTTGCGAATCTCTTCGCGGGCACCCAGCCGGCATGACTTACGCCGCAAACATCCTGCCCCATCATAGCGGGAGACCGGGGTCTCTGAGCCCCGCACGAGGCACCTTCGAAGATTTTACATGTGCTGCCAGCTCTGCTGTGAATGGTTCTGGAGCAAGGAGACGTCTCGCGCCTATC
asic1a_Frag1479-1638_insertion1578	GAGTCTGTACCCCGTCTCCAGCTCTGTCTTTGGATGATGTAAAACGCCATAATCCTTGCGAATCTCTTCGCGGGCACCCAGCCGGCATGACTTACGCCGCAAACATCCTGCCCCATCATCCCAGCGGGAGACCGGGGTCTCTGAGCGCACGAGGCACCTTCGAAGATTTTACATGTGCTGCCAGCTCTGCTGTGAATGGTTCTGGAGCAAGGAGACGTCTCGCGCCTATC
asic1a_Frag1479-1638_insertion1581	GAGTCTGTACCCCGTCTCCAGCTCTGTCTTTGGATGATGTAAAACGCCATAATCCTTGCGAATCTCTTCGCGGGCACCCAGCCGGCATGACTTACGCCGCAAACATCCTGCCCCATCATCCCGCAAGCGGGAGACCGGGGTCTCTGAGCCGAGGCACCTTCGAAGATTTTACATGTGCTGCCAGCTCTGCTGTGAATGGTTCTGGAGCAAGGAGACGTCTCGCGCCTATC
asic1a_Frag1479-1638_insertion1584	GAGTCTGTACCCCGTCTCCAGCTCTGTCTTTGGATGATGTAAAACGCCATAATCCTTGCGAATCTCTTCGCGGGCACCCAGCCGGCATGACTTACGCCGCAAACATCCTGCCCCATCATCCCGCACGAAGCGGGAGACCGGGGTCTCTGAGCGGCACCTTCGAAGATTTTACATGTGCTGCCAGCTCTGCTGTGAATGGTTCTGGAGCAAGGAGACGTCTCGCGCCTATC
asic1a_Frag1479-1638_insertion1587	GAGTCTGTACCCCGTCTCCAGCTCTGTCTTTGGATGATGTAAAACGCCATAATCCTTGCGAATCTCTTCGCGGGCACCCAGCCGGCATGACTTACGCCGCAAACATCCTGCCCCATCATCCCGCACGAGGCAGCGGGAGACCGGGGTCTCTGAGCACCTTCGAAGATTTTACATGTGCTGCCAGCTCTGCTGTGAATGGTTCTGGAGCAAGGAGACGTCTCGCGCCTATC
asic1a_Frag1479-1638_insertion1590	GAGTCTGTACCCCGTCTCCAGCTCTGTCTTTGGATGATGTAAAACGCCATAATCCTTGCGAATCTCTTCGCGGGCACCCAGCCGGCATGACTTACGCCGCAAACATCCTGCCCCATCATCCCGCACGAGGCACCAGCGGGAGACCGGGGTCTCTGAGCTTCGAAGATTTTACATGTGCTGCCAGCTCTGCTGTGAATGGTTCTGGAGCAAGGAGACGTCTCGCGCCTATC
asic1a_Frag1479-1638_insertion1593	GAGTCTGTACCCCGTCTCCAGCTCTGTCTTTGGATGATGTAAAACGCCATAATCCTTGCGAATCTCTTCGCGGGCACCCAGCCGGCATGACTTACGCCGCAAACATCCTGCCCCATCATCCCGCACGAGGCACCTTCAGCGGGAGACCGGGGTCTCTGAGCGAAGATTTTACATGTGCTGCCAGCTCTGCTGTGAATGGTTCTGGAGCAAGGAGACGTCTCGCGCCTATC
asic1a_Frag1479-1638_insertion1596	GAGTCTGTACCCCGTCTCCAGCTCTGTCTTTGGATGATGTAAAACGCCATAATCCTTGCGAATCTCTTCGCGGGCACCCAGCCGGCATGACTTACGCCGCAAACATCCTGCCCCATCATCCCGCACGAGGCACCTTCGAAAGCGGGAGACCGGGGTCTCTGAGCGATTTTACATGTGCTGCCAGCTCTGCTGTGAATGGTTCTGGAGCAAGGAGACGTCTCGCGCCTATC
asic1a_Frag1479-1638_insertion1599	GAGTCTGTACCCCGTCTCCAGCTCTGTCTTTGGATGATGTAAAACGCCATAATCCTTGCGAATCTCTTCGCGGGCACCCAGCCGGCATGACTTACGCCGCAAACATCCTGCCCCATCATCCCGCACGAGGCACCTTCGAAGATAGCGGGAGACCGGGGTCTCTGAGCTTTACATGTGCTGCCAGCTCTGCTGTGAATGGTTCTGGAGCAAGGAGACGTCTCGCGCCTATC
asic1a_Frag1479-1638_insertion1602	GAGTCTGTACCCCGTCTCCAGCTCTGTCTTTGGATGATGTAAAACGCCATAATCCTTGCGAATCTCTTCGCGGGCACCCAGCCGGCATGACTTACGCCGCAAACATCCTGCCCCATCATCCCGCACGAGGCACCTTCGAAGATTTTAGCGGGAGACCGGGGTCTCTGAGCACATGTGCTGCCAGCTCTGCTGTGAATGGTTCTGGAGCAAGGAGACGTCTCGCGCCTATC
asic1a_Frag1479-1638_insertion1605	GAGTCTGTACCCCGTCTCCAGCTCTGTCTTTGGATGATGTAAAACGCCATAATCCTTGCGAATCTCTTCGCGGGCACCCAGCCGGCATGACTTACGCCGCAAACATCCTGCCCCATCATCCCGCACGAGGCACCTTCGAAGATTTTACAAGCGGGAGACCGGGGTCTCTGAGCTGTGCTGCCAGCTCTGCTGTGAATGGTTCTGGAGCAAGGAGACGTCTCGCGCCTATC
asic1a_Frag1479-1638_insertion1608	GAGTCTGTACCCCGTCTCCAGCTCTGTCTTTGGATGATGTAAAACGCCATAATCCTTGCGAATCTCTTCGCGGGCACCCAGCCGGCATGACTTACGCCGCAAACATCCTGCCCCATCATCCCGCACGAGGCACCTTCGAAGATTTTACATGTAGCGGGAGACCGGGGTCTCTGAGCGCTGCCAGCTCTGCTGTGAATGGTTCTGGAGCAAGGAGACGTCTCGCGCCTATC
asic1a_Frag1479-1638_insertion1611	GAGTCTGTACCCCGTCTCCAGCTCTGTCTTTGGATGATGTAAAACGCCATAATCCTTGCGAATCTCTTCGCGGGCACCCAGCCGGCATGACTTACGCCGCAAACATCCTGCCCCATCATCCCGCACGAGGCACCTTCGAAGATTTTACATGTGCTAGCGGGAGACCGGGGTCTCTGAGCGCCAGCTCTGCTGTGAATGGTTCTGGAGCAAGGAGACGTCTCGCGCCTATC
asic1a_Frag1479-1638_insertion1614	GAGTCTGTACCCCGTCTCCAGCTCTGTCTTTGGATGATGTAAAACGCCATAATCCTTGCGAATCTCTTCGCGGGCACCCAGCCGGCATGACTTACGCCGCAAACATCCTGCCCCATCATCCCGCACGAGGCACCTTCGAAGATTTTACATGTGCTGCCAGCGGGAGACCGGGGTCTCTGAGCAGCTCTGCTGTGAATGGTTCTGGAGCAAGGAGACGTCTCGCGCCTATC
asic1a_Frag1479-1638_insertion1617	GAGTCTGTACCCCGTCTCCAGCTCTGTCTTTGGATGATGTAAAACGCCATAATCCTTGCGAATCTCTTCGCGGGCACCCAGCCGGCATGACTTACGCCGCAAACATCCTGCCCCATCATCCCGCACGAGGCACCTTCGAAGATTTTACATGTGCTGCCAGCAGCGGGAGACCGGGGTCTCTGAGCTCTGCTGTGAATGGTTCTGGAGCAAGGAGACGTCTCGCGCCTATC
asic1a_Frag1479-1638_insertion1620	GAGTCTGTACCCCGTCTCCAGCTCTGTCTTTGGATGATGTAAAACGCCATAATCCTTGCGAATCTCTTCGCGGGCACCCAGCCGGCATGACTTACGCCGCAAACATCCTGCCCCATCATCCCGCACGAGGCACCTTCGAAGATTTTACATGTGCTGCCAGCTCTAGCGGGAGACCGGGGTCTCTGAGCGCTGTGAATGGTTCTGGAGCAAGGAGACGTCTCGCGCCTATC
asic1a_Frag1479-1638_insertion1623	GAGTCTGTACCCCGTCTCCAGCTCTGTCTTTGGATGATGTAAAACGCCATAATCCTTGCGAATCTCTTCGCGGGCACCCAGCCGGCATGACTTACGCCGCAAACATCCTGCCCCATCATCCCGCACGAGGCACCTTCGAAGATTTTACATGTGCTGCCAGCTCTGCTAGCGGGAGACCGGGGTCTCTGAGCGTGAATGGTTCTGGAGCAAGGAGACGTCTCGCGCCTATC
asic1a_Frag1479-1638_insertion1626	GAGTCTGTACCCCGTCTCCAGCTCTGTCTTTGGATGATGTAAAACGCCATAATCCTTGCGAATCTCTTCGCGGGCACCCAGCCGGCATGACTTACGCCGCAAACATCCTGCCCCATCATCCCGCACGAGGCACCTTCGAAGATTTTACATGTGCTGCCAGCTCTGCTGTGAGCGGGAGACCGGGGTCTCTGAGCAATGGTTCTGGAGCAAGGAGACGTCTCGCGCCTATC
asic1a_Frag1479-1638_insertion1629	GAGTCTGTACCCCGTCTCCAGCTCTGTCTTTGGATGATGTAAAACGCCATAATCCTTGCGAATCTCTTCGCGGGCACCCAGCCGGCATGACTTACGCCGCAAACATCCTGCCCCATCATCCCGCACGAGGCACCTTCGAAGATTTTACATGTGCTGCCAGCTCTGCTGTGAATAGCGGGAGACCGGGGTCTCTGAGCGGTTCTGGAGCAAGGAGACGTCTCGCGCCTATC
asic1a_Frag1479-1638_insertion1632	GAGTCTGTACCCCGTCTCCAGCTCTGTCTTTGGATGATGTAAAACGCCATAATCCTTGCGAATCTCTTCGCGGGCACCCAGCCGGCATGACTTACGCCGCAAACATCCTGCCCCATCATCCCGCACGAGGCACCTTCGAAGATTTTACATGTGCTGCCAGCTCTGCTGTGAATGGTAGCGGGAGACCGGGGTCTCTGAGCTCTGGAGCAAGGAGACGTCTCGCGCCTATC
asic1a_Frag1479-1638_insertion1635	GAGTCTGTACCCCGTCTCCAGCTCTGTCTTTGGATGATGTAAAACGCCATAATCCTTGCGAATCTCTTCGCGGGCACCCAGCCGGCATGACTTACGCCGCAAACATCCTGCCCCATCATCCCGCACGAGGCACCTTCGAAGATTTTACATGTGCTGCCAGCTCTGCTGTGAATGGTTCTAGCGGGAGACCGGGGTCTCTGAGCGGAGCAAGGAGACGTCTCGCGCCTATC
asic1a_Frag1479-1638_insertion1638	GAGTCTGTACCCCGTCTCCAGCTCTGTCTTTGGATGATGTAAAACGCCATAATCCTTGCGAATCTCTTCGCGGGCACCCAGCCGGCATGACTTACGCCGCAAACATCCTGCCCCATCATCCCGCACGAGGCACCTTCGAAGATTTTACATGTGCTGCCAGCTCTGCTGTGAATGGTTCTGGAAGCGGGAGACCGGGGTCTCTGAGCGCAAGGAGACGTCTCGCGCCTATC
nachra7_Frag3-168_insertion3	TTGGTCATGCGTCTCCCATGAGCGGGAGACCGGGGTCTCTGAGCCGCTGCTCGCCGGGAGGCGTCTGGCTGGCGCTGGCCGCGTCGCTCCTGCACGTGTCCCTGCAAGGCGAGTTCCAGAGGAAGCTTTACAAGGAGCTGGTCAAGAACTACAATCCCTTGGAGAGGCCCGTGGCCAATGACTCGCAACCACTCACCGTCTACTTCTCCCTGAGGAGACGTCTGAGTATC
nachra7_Frag3-168_insertion6	TTGGTCATGCGTCTCCCATGCGCAGCGGGAGACCGGGGTCTCTGAGCTGCTCGCCGGGAGGCGTCTGGCTGGCGCTGGCCGCGTCGCTCCTGCACGTGTCCCTGCAAGGCGAGTTCCAGAGGAAGCTTTACAAGGAGCTGGTCAAGAACTACAATCCCTTGGAGAGGCCCGTGGCCAATGACTCGCAACCACTCACCGTCTACTTCTCCCTGAGGAGACGTCTGAGTATC
nachra7_Frag3-168_insertion9	TTGGTCATGCGTCTCCCATGCGCTGCAGCGGGAGACCGGGGTCTCTGAGCTCGCCGGGAGGCGTCTGGCTGGCGCTGGCCGCGTCGCTCCTGCACGTGTCCCTGCAAGGCGAGTTCCAGAGGAAGCTTTACAAGGAGCTGGTCAAGAACTACAATCCCTTGGAGAGGCCCGTGGCCAATGACTCGCAACCACTCACCGTCTACTTCTCCCTGAGGAGACGTCTGAGTATC
nachra7_Frag3-168_insertion12	TTGGTCATGCGTCTCCCATGCGCTGCTCGAGCGGGAGACCGGGGTCTCTGAGCCCGGGAGGCGTCTGGCTGGCGCTGGCCGCGTCGCTCCTGCACGTGTCCCTGCAAGGCGAGTTCCAGAGGAAGCTTTACAAGGAGCTGGTCAAGAACTACAATCCCTTGGAGAGGCCCGTGGCCAATGACTCGCAACCACTCACCGTCTACTTCTCCCTGAGGAGACGTCTGAGTATC
nachra7_Frag3-168_insertion15	TTGGTCATGCGTCTCCCATGCGCTGCTCGCCGAGCGGGAGACCGGGGTCTCTGAGCGGAGGCGTCTGGCTGGCGCTGGCCGCGTCGCTCCTGCACGTGTCCCTGCAAGGCGAGTTCCAGAGGAAGCTTTACAAGGAGCTGGTCAAGAACTACAATCCCTTGGAGAGGCCCGTGGCCAATGACTCGCAACCACTCACCGTCTACTTCTCCCTGAGGAGACGTCTGAGTATC
nachra7_Frag3-168_insertion18	TTGGTCATGCGTCTCCCATGCGCTGCTCGCCGGGAAGCGGGAGACCGGGGTCTCTGAGCGGCGTCTGGCTGGCGCTGGCCGCGTCGCTCCTGCACGTGTCCCTGCAAGGCGAGTTCCAGAGGAAGCTTTACAAGGAGCTGGTCAAGAACTACAATCCCTTGGAGAGGCCCGTGGCCAATGACTCGCAACCACTCACCGTCTACTTCTCCCTGAGGAGACGTCTGAGTATC
nachra7_Frag3-168_insertion21	TTGGTCATGCGTCTCCCATGCGCTGCTCGCCGGGAGGCAGCGGGAGACCGGGGTCTCTGAGCGTCTGGCTGGCGCTGGCCGCGTCGCTCCTGCACGTGTCCCTGCAAGGCGAGTTCCAGAGGAAGCTTTACAAGGAGCTGGTCAAGAACTACAATCCCTTGGAGAGGCCCGTGGCCAATGACTCGCAACCACTCACCGTCTACTTCTCCCTGAGGAGACGTCTGAGTATC
nachra7_Frag3-168_insertion24	TTGGTCATGCGTCTCCCATGCGCTGCTCGCCGGGAGGCGTCAGCGGGAGACCGGGGTCTCTGAGCTGGCTGGCGCTGGCCGCGTCGCTCCTGCACGTGTCCCTGCAAGGCGAGTTCCAGAGGAAGCTTTACAAGGAGCTGGTCAAGAACTACAATCCCTTGGAGAGGCCCGTGGCCAATGACTCGCAACCACTCACCGTCTACTTCTCCCTGAGGAGACGTCTGAGTATC
nachra7_Frag3-168_insertion27	TTGGTCATGCGTCTCCCATGCGCTGCTCGCCGGGAGGCGTCTGGAGCGGGAGACCGGGGTCTCTGAGCCTGGCGCTGGCCGCGTCGCTCCTGCACGTGTCCCTGCAAGGCGAGTTCCAGAGGAAGCTTTACAAGGAGCTGGTCAAGAACTACAATCCCTTGGAGAGGCCCGTGGCCAATGACTCGCAACCACTCACCGTCTACTTCTCCCTGAGGAGACGTCTGAGTATC
nachra7_Frag3-168_insertion30	TTGGTCATGCGTCTCCCATGCGCTGCTCGCCGGGAGGCGTCTGGCTGAGCGGGAGACCGGGGTCTCTGAGCGCGCTGGCCGCGTCGCTCCTGCACGTGTCCCTGCAAGGCGAGTTCCAGAGGAAGCTTTACAAGGAGCTGGTCAAGAACTACAATCCCTTGGAGAGGCCCGTGGCCAATGACTCGCAACCACTCACCGTCTACTTCTCCCTGAGGAGACGTCTGAGTATC
nachra7_Frag3-168_insertion33	TTGGTCATGCGTCTCCCATGCGCTGCTCGCCGGGAGGCGTCTGGCTGGCGAGCGGGAGACCGGGGTCTCTGAGCCTGGCCGCGTCGCTCCTGCACGTGTCCCTGCAAGGCGAGTTCCAGAGGAAGCTTTACAAGGAGCTGGTCAAGAACTACAATCCCTTGGAGAGGCCCGTGGCCAATGACTCGCAACCACTCACCGTCTACTTCTCCCTGAGGAGACGTCTGAGTATC
nachra7_Frag3-168_insertion36	TTGGTCATGCGTCTCCCATGCGCTGCTCGCCGGGAGGCGTCTGGCTGGCGCTGAGCGGGAGACCGGGGTCTCTGAGCGCCGCGTCGCTCCTGCACGTGTCCCTGCAAGGCGAGTTCCAGAGGAAGCTTTACAAGGAGCTGGTCAAGAACTACAATCCCTTGGAGAGGCCCGTGGCCAATGACTCGCAACCACTCACCGTCTACTTCTCCCTGAGGAGACGTCTGAGTATC
nachra7_Frag3-168_insertion39	TTGGTCATGCGTCTCCCATGCGCTGCTCGCCGGGAGGCGTCTGGCTGGCGCTGGCCAGCGGGAGACCGGGGTCTCTGAGCGCGTCGCTCCTGCACGTGTCCCTGCAAGGCGAGTTCCAGAGGAAGCTTTACAAGGAGCTGGTCAAGAACTACAATCCCTTGGAGAGGCCCGTGGCCAATGACTCGCAACCACTCACCGTCTACTTCTCCCTGAGGAGACGTCTGAGTATC
nachra7_Frag3-168_insertion42	TTGGTCATGCGTCTCCCATGCGCTGCTCGCCGGGAGGCGTCTGGCTGGCGCTGGCCGCGAGCGGGAGACCGGGGTCTCTGAGCTCGCTCCTGCACGTGTCCCTGCAAGGCGAGTTCCAGAGGAAGCTTTACAAGGAGCTGGTCAAGAACTACAATCCCTTGGAGAGGCCCGTGGCCAATGACTCGCAACCACTCACCGTCTACTTCTCCCTGAGGAGACGTCTGAGTATC
nachra7_Frag3-168_insertion45	TTGGTCATGCGTCTCCCATGCGCTGCTCGCCGGGAGGCGTCTGGCTGGCGCTGGCCGCGTCGAGCGGGAGACCGGGGTCTCTGAGCCTCCTGCACGTGTCCCTGCAAGGCGAGTTCCAGAGGAAGCTTTACAAGGAGCTGGTCAAGAACTACAATCCCTTGGAGAGGCCCGTGGCCAATGACTCGCAACCACTCACCGTCTACTTCTCCCTGAGGAGACGTCTGAGTATC
nachra7_Frag3-168_insertion48	TTGGTCATGCGTCTCCCATGCGCTGCTCGCCGGGAGGCGTCTGGCTGGCGCTGGCCGCGTCGCTCAGCGGGAGACCGGGGTCTCTGAGCCTGCACGTGTCCCTGCAAGGCGAGTTCCAGAGGAAGCTTTACAAGGAGCTGGTCAAGAACTACAATCCCTTGGAGAGGCCCGTGGCCAATGACTCGCAACCACTCACCGTCTACTTCTCCCTGAGGAGACGTCTGAGTATC
nachra7_Frag3-168_insertion51	TTGGTCATGCGTCTCCCATGCGCTGCTCGCCGGGAGGCGTCTGGCTGGCGCTGGCCGCGTCGCTCCTGAGCGGGAGACCGGGGTCTCTGAGCCACGTGTCCCTGCAAGGCGAGTTCCAGAGGAAGCTTTACAAGGAGCTGGTCAAGAACTACAATCCCTTGGAGAGGCCCGTGGCCAATGACTCGCAACCACTCACCGTCTACTTCTCCCTGAGGAGACGTCTGAGTATC
nachra7_Frag3-168_insertion54	TTGGTCATGCGTCTCCCATGCGCTGCTCGCCGGGAGGCGTCTGGCTGGCGCTGGCCGCGTCGCTCCTGCACAGCGGGAGACCGGGGTCTCTGAGCGTGTCCCTGCAAGGCGAGTTCCAGAGGAAGCTTTACAAGGAGCTGGTCAAGAACTACAATCCCTTGGAGAGGCCCGTGGCCAATGACTCGCAACCACTCACCGTCTACTTCTCCCTGAGGAGACGTCTGAGTATC
nachra7_Frag3-168_insertion57	TTGGTCATGCGTCTCCCATGCGCTGCTCGCCGGGAGGCGTCTGGCTGGCGCTGGCCGCGTCGCTCCTGCACGTGAGCGGGAGACCGGGGTCTCTGAGCTCCCTGCAAGGCGAGTTCCAGAGGAAGCTTTACAAGGAGCTGGTCAAGAACTACAATCCCTTGGAGAGGCCCGTGGCCAATGACTCGCAACCACTCACCGTCTACTTCTCCCTGAGGAGACGTCTGAGTATC
nachra7_Frag3-168_insertion60	TTGGTCATGCGTCTCCCATGCGCTGCTCGCCGGGAGGCGTCTGGCTGGCGCTGGCCGCGTCGCTCCTGCACGTGTCCAGCGGGAGACCGGGGTCTCTGAGCCTGCAAGGCGAGTTCCAGAGGAAGCTTTACAAGGAGCTGGTCAAGAACTACAATCCCTTGGAGAGGCCCGTGGCCAATGACTCGCAACCACTCACCGTCTACTTCTCCCTGAGGAGACGTCTGAGTATC
nachra7_Frag3-168_insertion63	TTGGTCATGCGTCTCCCATGCGCTGCTCGCCGGGAGGCGTCTGGCTGGCGCTGGCCGCGTCGCTCCTGCACGTGTCCCTGAGCGGGAGACCGGGGTCTCTGAGCCAAGGCGAGTTCCAGAGGAAGCTTTACAAGGAGCTGGTCAAGAACTACAATCCCTTGGAGAGGCCCGTGGCCAATGACTCGCAACCACTCACCGTCTACTTCTCCCTGAGGAGACGTCTGAGTATC
nachra7_Frag3-168_insertion66	TTGGTCATGCGTCTCCCATGCGCTGCTCGCCGGGAGGCGTCTGGCTGGCGCTGGCCGCGTCGCTCCTGCACGTGTCCCTGCAAAGCGGGAGACCGGGGTCTCTGAGCGGCGAGTTCCAGAGGAAGCTTTACAAGGAGCTGGTCAAGAACTACAATCCCTTGGAGAGGCCCGTGGCCAATGACTCGCAACCACTCACCGTCTACTTCTCCCTGAGGAGACGTCTGAGTATC
nachra7_Frag3-168_insertion69	TTGGTCATGCGTCTCCCATGCGCTGCTCGCCGGGAGGCGTCTGGCTGGCGCTGGCCGCGTCGCTCCTGCACGTGTCCCTGCAAGGCAGCGGGAGACCGGGGTCTCTGAGCGAGTTCCAGAGGAAGCTTTACAAGGAGCTGGTCAAGAACTACAATCCCTTGGAGAGGCCCGTGGCCAATGACTCGCAACCACTCACCGTCTACTTCTCCCTGAGGAGACGTCTGAGTATC
nachra7_Frag3-168_insertion72	TTGGTCATGCGTCTCCCATGCGCTGCTCGCCGGGAGGCGTCTGGCTGGCGCTGGCCGCGTCGCTCCTGCACGTGTCCCTGCAAGGCGAGAGCGGGAGACCGGGGTCTCTGAGCTTCCAGAGGAAGCTTTACAAGGAGCTGGTCAAGAACTACAATCCCTTGGAGAGGCCCGTGGCCAATGACTCGCAACCACTCACCGTCTACTTCTCCCTGAGGAGACGTCTGAGTATC
nachra7_Frag3-168_insertion75	TTGGTCATGCGTCTCCCATGCGCTGCTCGCCGGGAGGCGTCTGGCTGGCGCTGGCCGCGTCGCTCCTGCACGTGTCCCTGCAAGGCGAGTTCAGCGGGAGACCGGGGTCTCTGAGCCAGAGGAAGCTTTACAAGGAGCTGGTCAAGAACTACAATCCCTTGGAGAGGCCCGTGGCCAATGACTCGCAACCACTCACCGTCTACTTCTCCCTGAGGAGACGTCTGAGTATC
nachra7_Frag3-168_insertion78	TTGGTCATGCGTCTCCCATGCGCTGCTCGCCGGGAGGCGTCTGGCTGGCGCTGGCCGCGTCGCTCCTGCACGTGTCCCTGCAAGGCGAGTTCCAGAGCGGGAGACCGGGGTCTCTGAGCAGGAAGCTTTACAAGGAGCTGGTCAAGAACTACAATCCCTTGGAGAGGCCCGTGGCCAATGACTCGCAACCACTCACCGTCTACTTCTCCCTGAGGAGACGTCTGAGTATC
nachra7_Frag3-168_insertion81	TTGGTCATGCGTCTCCCATGCGCTGCTCGCCGGGAGGCGTCTGGCTGGCGCTGGCCGCGTCGCTCCTGCACGTGTCCCTGCAAGGCGAGTTCCAGAGGAGCGGGAGACCGGGGTCTCTGAGCAAGCTTTACAAGGAGCTGGTCAAGAACTACAATCCCTTGGAGAGGCCCGTGGCCAATGACTCGCAACCACTCACCGTCTACTTCTCCCTGAGGAGACGTCTGAGTATC
nachra7_Frag3-168_insertion84	TTGGTCATGCGTCTCCCATGCGCTGCTCGCCGGGAGGCGTCTGGCTGGCGCTGGCCGCGTCGCTCCTGCACGTGTCCCTGCAAGGCGAGTTCCAGAGGAAGAGCGGGAGACCGGGGTCTCTGAGCCTTTACAAGGAGCTGGTCAAGAACTACAATCCCTTGGAGAGGCCCGTGGCCAATGACTCGCAACCACTCACCGTCTACTTCTCCCTGAGGAGACGTCTGAGTATC
nachra7_Frag3-168_insertion87	TTGGTCATGCGTCTCCCATGCGCTGCTCGCCGGGAGGCGTCTGGCTGGCGCTGGCCGCGTCGCTCCTGCACGTGTCCCTGCAAGGCGAGTTCCAGAGGAAGCTTAGCGGGAGACCGGGGTCTCTGAGCTACAAGGAGCTGGTCAAGAACTACAATCCCTTGGAGAGGCCCGTGGCCAATGACTCGCAACCACTCACCGTCTACTTCTCCCTGAGGAGACGTCTGAGTATC
nachra7_Frag3-168_insertion90	TTGGTCATGCGTCTCCCATGCGCTGCTCGCCGGGAGGCGTCTGGCTGGCGCTGGCCGCGTCGCTCCTGCACGTGTCCCTGCAAGGCGAGTTCCAGAGGAAGCTTTACAGCGGGAGACCGGGGTCTCTGAGCAAGGAGCTGGTCAAGAACTACAATCCCTTGGAGAGGCCCGTGGCCAATGACTCGCAACCACTCACCGTCTACTTCTCCCTGAGGAGACGTCTGAGTATC
nachra7_Frag3-168_insertion93	TTGGTCATGCGTCTCCCATGCGCTGCTCGCCGGGAGGCGTCTGGCTGGCGCTGGCCGCGTCGCTCCTGCACGTGTCCCTGCAAGGCGAGTTCCAGAGGAAGCTTTACAAGAGCGGGAGACCGGGGTCTCTGAGCGAGCTGGTCAAGAACTACAATCCCTTGGAGAGGCCCGTGGCCAATGACTCGCAACCACTCACCGTCTACTTCTCCCTGAGGAGACGTCTGAGTATC
nachra7_Frag3-168_insertion96	TTGGTCATGCGTCTCCCATGCGCTGCTCGCCGGGAGGCGTCTGGCTGGCGCTGGCCGCGTCGCTCCTGCACGTGTCCCTGCAAGGCGAGTTCCAGAGGAAGCTTTACAAGGAGAGCGGGAGACCGGGGTCTCTGAGCCTGGTCAAGAACTACAATCCCTTGGAGAGGCCCGTGGCCAATGACTCGCAACCACTCACCGTCTACTTCTCCCTGAGGAGACGTCTGAGTATC
nachra7_Frag3-168_insertion99	TTGGTCATGCGTCTCCCATGCGCTGCTCGCCGGGAGGCGTCTGGCTGGCGCTGGCCGCGTCGCTCCTGCACGTGTCCCTGCAAGGCGAGTTCCAGAGGAAGCTTTACAAGGAGCTGAGCGGGAGACCGGGGTCTCTGAGCGTCAAGAACTACAATCCCTTGGAGAGGCCCGTGGCCAATGACTCGCAACCACTCACCGTCTACTTCTCCCTGAGGAGACGTCTGAGTATC
nachra7_Frag3-168_insertion102	TTGGTCATGCGTCTCCCATGCGCTGCTCGCCGGGAGGCGTCTGGCTGGCGCTGGCCGCGTCGCTCCTGCACGTGTCCCTGCAAGGCGAGTTCCAGAGGAAGCTTTACAAGGAGCTGGTCAGCGGGAGACCGGGGTCTCTGAGCAAGAACTACAATCCCTTGGAGAGGCCCGTGGCCAATGACTCGCAACCACTCACCGTCTACTTCTCCCTGAGGAGACGTCTGAGTATC
nachra7_Frag3-168_insertion105	TTGGTCATGCGTCTCCCATGCGCTGCTCGCCGGGAGGCGTCTGGCTGGCGCTGGCCGCGTCGCTCCTGCACGTGTCCCTGCAAGGCGAGTTCCAGAGGAAGCTTTACAAGGAGCTGGTCAAGAGCGGGAGACCGGGGTCTCTGAGCAACTACAATCCCTTGGAGAGGCCCGTGGCCAATGACTCGCAACCACTCACCGTCTACTTCTCCCTGAGGAGACGTCTGAGTATC
nachra7_Frag3-168_insertion108	TTGGTCATGCGTCTCCCATGCGCTGCTCGCCGGGAGGCGTCTGGCTGGCGCTGGCCGCGTCGCTCCTGCACGTGTCCCTGCAAGGCGAGTTCCAGAGGAAGCTTTACAAGGAGCTGGTCAAGAACAGCGGGAGACCGGGGTCTCTGAGCTACAATCCCTTGGAGAGGCCCGTGGCCAATGACTCGCAACCACTCACCGTCTACTTCTCCCTGAGGAGACGTCTGAGTATC
nachra7_Frag3-168_insertion111	TTGGTCATGCGTCTCCCATGCGCTGCTCGCCGGGAGGCGTCTGGCTGGCGCTGGCCGCGTCGCTCCTGCACGTGTCCCTGCAAGGCGAGTTCCAGAGGAAGCTTTACAAGGAGCTGGTCAAGAACTACAGCGGGAGACCGGGGTCTCTGAGCAATCCCTTGGAGAGGCCCGTGGCCAATGACTCGCAACCACTCACCGTCTACTTCTCCCTGAGGAGACGTCTGAGTATC
nachra7_Frag3-168_insertion114	TTGGTCATGCGTCTCCCATGCGCTGCTCGCCGGGAGGCGTCTGGCTGGCGCTGGCCGCGTCGCTCCTGCACGTGTCCCTGCAAGGCGAGTTCCAGAGGAAGCTTTACAAGGAGCTGGTCAAGAACTACAATAGCGGGAGACCGGGGTCTCTGAGCCCCTTGGAGAGGCCCGTGGCCAATGACTCGCAACCACTCACCGTCTACTTCTCCCTGAGGAGACGTCTGAGTATC
nachra7_Frag3-168_insertion117	TTGGTCATGCGTCTCCCATGCGCTGCTCGCCGGGAGGCGTCTGGCTGGCGCTGGCCGCGTCGCTCCTGCACGTGTCCCTGCAAGGCGAGTTCCAGAGGAAGCTTTACAAGGAGCTGGTCAAGAACTACAATCCCAGCGGGAGACCGGGGTCTCTGAGCTTGGAGAGGCCCGTGGCCAATGACTCGCAACCACTCACCGTCTACTTCTCCCTGAGGAGACGTCTGAGTATC
nachra7_Frag3-168_insertion120	TTGGTCATGCGTCTCCCATGCGCTGCTCGCCGGGAGGCGTCTGGCTGGCGCTGGCCGCGTCGCTCCTGCACGTGTCCCTGCAAGGCGAGTTCCAGAGGAAGCTTTACAAGGAGCTGGTCAAGAACTACAATCCCTTGAGCGGGAGACCGGGGTCTCTGAGCGAGAGGCCCGTGGCCAATGACTCGCAACCACTCACCGTCTACTTCTCCCTGAGGAGACGTCTGAGTATC
nachra7_Frag3-168_insertion123	TTGGTCATGCGTCTCCCATGCGCTGCTCGCCGGGAGGCGTCTGGCTGGCGCTGGCCGCGTCGCTCCTGCACGTGTCCCTGCAAGGCGAGTTCCAGAGGAAGCTTTACAAGGAGCTGGTCAAGAACTACAATCCCTTGGAGAGCGGGAGACCGGGGTCTCTGAGCAGGCCCGTGGCCAATGACTCGCAACCACTCACCGTCTACTTCTCCCTGAGGAGACGTCTGAGTATC
nachra7_Frag3-168_insertion126	TTGGTCATGCGTCTCCCATGCGCTGCTCGCCGGGAGGCGTCTGGCTGGCGCTGGCCGCGTCGCTCCTGCACGTGTCCCTGCAAGGCGAGTTCCAGAGGAAGCTTTACAAGGAGCTGGTCAAGAACTACAATCCCTTGGAGAGGAGCGGGAGACCGGGGTCTCTGAGCCCCGTGGCCAATGACTCGCAACCACTCACCGTCTACTTCTCCCTGAGGAGACGTCTGAGTATC
nachra7_Frag3-168_insertion129	TTGGTCATGCGTCTCCCATGCGCTGCTCGCCGGGAGGCGTCTGGCTGGCGCTGGCCGCGTCGCTCCTGCACGTGTCCCTGCAAGGCGAGTTCCAGAGGAAGCTTTACAAGGAGCTGGTCAAGAACTACAATCCCTTGGAGAGGCCCAGCGGGAGACCGGGGTCTCTGAGCGTGGCCAATGACTCGCAACCACTCACCGTCTACTTCTCCCTGAGGAGACGTCTGAGTATC
nachra7_Frag3-168_insertion132	TTGGTCATGCGTCTCCCATGCGCTGCTCGCCGGGAGGCGTCTGGCTGGCGCTGGCCGCGTCGCTCCTGCACGTGTCCCTGCAAGGCGAGTTCCAGAGGAAGCTTTACAAGGAGCTGGTCAAGAACTACAATCCCTTGGAGAGGCCCGTGAGCGGGAGACCGGGGTCTCTGAGCGCCAATGACTCGCAACCACTCACCGTCTACTTCTCCCTGAGGAGACGTCTGAGTATC
nachra7_Frag3-168_insertion135	TTGGTCATGCGTCTCCCATGCGCTGCTCGCCGGGAGGCGTCTGGCTGGCGCTGGCCGCGTCGCTCCTGCACGTGTCCCTGCAAGGCGAGTTCCAGAGGAAGCTTTACAAGGAGCTGGTCAAGAACTACAATCCCTTGGAGAGGCCCGTGGCCAGCGGGAGACCGGGGTCTCTGAGCAATGACTCGCAACCACTCACCGTCTACTTCTCCCTGAGGAGACGTCTGAGTATC
nachra7_Frag3-168_insertion138	TTGGTCATGCGTCTCCCATGCGCTGCTCGCCGGGAGGCGTCTGGCTGGCGCTGGCCGCGTCGCTCCTGCACGTGTCCCTGCAAGGCGAGTTCCAGAGGAAGCTTTACAAGGAGCTGGTCAAGAACTACAATCCCTTGGAGAGGCCCGTGGCCAATAGCGGGAGACCGGGGTCTCTGAGCGACTCGCAACCACTCACCGTCTACTTCTCCCTGAGGAGACGTCTGAGTATC
nachra7_Frag3-168_insertion141	TTGGTCATGCGTCTCCCATGCGCTGCTCGCCGGGAGGCGTCTGGCTGGCGCTGGCCGCGTCGCTCCTGCACGTGTCCCTGCAAGGCGAGTTCCAGAGGAAGCTTTACAAGGAGCTGGTCAAGAACTACAATCCCTTGGAGAGGCCCGTGGCCAATGACAGCGGGAGACCGGGGTCTCTGAGCTCGCAACCACTCACCGTCTACTTCTCCCTGAGGAGACGTCTGAGTATC
nachra7_Frag3-168_insertion144	TTGGTCATGCGTCTCCCATGCGCTGCTCGCCGGGAGGCGTCTGGCTGGCGCTGGCCGCGTCGCTCCTGCACGTGTCCCTGCAAGGCGAGTTCCAGAGGAAGCTTTACAAGGAGCTGGTCAAGAACTACAATCCCTTGGAGAGGCCCGTGGCCAATGACTCGAGCGGGAGACCGGGGTCTCTGAGCCAACCACTCACCGTCTACTTCTCCCTGAGGAGACGTCTGAGTATC
nachra7_Frag3-168_insertion147	TTGGTCATGCGTCTCCCATGCGCTGCTCGCCGGGAGGCGTCTGGCTGGCGCTGGCCGCGTCGCTCCTGCACGTGTCCCTGCAAGGCGAGTTCCAGAGGAAGCTTTACAAGGAGCTGGTCAAGAACTACAATCCCTTGGAGAGGCCCGTGGCCAATGACTCGCAAAGCGGGAGACCGGGGTCTCTGAGCCCACTCACCGTCTACTTCTCCCTGAGGAGACGTCTGAGTATC
nachra7_Frag3-168_insertion150	TTGGTCATGCGTCTCCCATGCGCTGCTCGCCGGGAGGCGTCTGGCTGGCGCTGGCCGCGTCGCTCCTGCACGTGTCCCTGCAAGGCGAGTTCCAGAGGAAGCTTTACAAGGAGCTGGTCAAGAACTACAATCCCTTGGAGAGGCCCGTGGCCAATGACTCGCAACCAAGCGGGAGACCGGGGTCTCTGAGCCTCACCGTCTACTTCTCCCTGAGGAGACGTCTGAGTATC
nachra7_Frag3-168_insertion153	TTGGTCATGCGTCTCCCATGCGCTGCTCGCCGGGAGGCGTCTGGCTGGCGCTGGCCGCGTCGCTCCTGCACGTGTCCCTGCAAGGCGAGTTCCAGAGGAAGCTTTACAAGGAGCTGGTCAAGAACTACAATCCCTTGGAGAGGCCCGTGGCCAATGACTCGCAACCACTCAGCGGGAGACCGGGGTCTCTGAGCACCGTCTACTTCTCCCTGAGGAGACGTCTGAGTATC
nachra7_Frag3-168_insertion156	TTGGTCATGCGTCTCCCATGCGCTGCTCGCCGGGAGGCGTCTGGCTGGCGCTGGCCGCGTCGCTCCTGCACGTGTCCCTGCAAGGCGAGTTCCAGAGGAAGCTTTACAAGGAGCTGGTCAAGAACTACAATCCCTTGGAGAGGCCCGTGGCCAATGACTCGCAACCACTCACCAGCGGGAGACCGGGGTCTCTGAGCGTCTACTTCTCCCTGAGGAGACGTCTGAGTATC
nachra7_Frag3-168_insertion159	TTGGTCATGCGTCTCCCATGCGCTGCTCGCCGGGAGGCGTCTGGCTGGCGCTGGCCGCGTCGCTCCTGCACGTGTCCCTGCAAGGCGAGTTCCAGAGGAAGCTTTACAAGGAGCTGGTCAAGAACTACAATCCCTTGGAGAGGCCCGTGGCCAATGACTCGCAACCACTCACCGTCAGCGGGAGACCGGGGTCTCTGAGCTACTTCTCCCTGAGGAGACGTCTGAGTATC
nachra7_Frag3-168_insertion162	TTGGTCATGCGTCTCCCATGCGCTGCTCGCCGGGAGGCGTCTGGCTGGCGCTGGCCGCGTCGCTCCTGCACGTGTCCCTGCAAGGCGAGTTCCAGAGGAAGCTTTACAAGGAGCTGGTCAAGAACTACAATCCCTTGGAGAGGCCCGTGGCCAATGACTCGCAACCACTCACCGTCTACAGCGGGAGACCGGGGTCTCTGAGCTTCTCCCTGAGGAGACGTCTGAGTATC
nachra7_Frag3-168_insertion165	TTGGTCATGCGTCTCCCATGCGCTGCTCGCCGGGAGGCGTCTGGCTGGCGCTGGCCGCGTCGCTCCTGCACGTGTCCCTGCAAGGCGAGTTCCAGAGGAAGCTTTACAAGGAGCTGGTCAAGAACTACAATCCCTTGGAGAGGCCCGTGGCCAATGACTCGCAACCACTCACCGTCTACTTCAGCGGGAGACCGGGGTCTCTGAGCTCCCTGAGGAGACGTCTGAGTATC
nachra7_Frag3-168_insertion168	TTGGTCATGCGTCTCCCATGCGCTGCTCGCCGGGAGGCGTCTGGCTGGCGCTGGCCGCGTCGCTCCTGCACGTGTCCCTGCAAGGCGAGTTCCAGAGGAAGCTTTACAAGGAGCTGGTCAAGAACTACAATCCCTTGGAGAGGCCCGTGGCCAATGACTCGCAACCACTCACCGTCTACTTCTCCAGCGGGAGACCGGGGTCTCTGAGCCTGAGGAGACGTCTGAGTATC
nachra7_Frag171-336_insertion171	GGGTGGGTACGTCTCCCCTGAGCGGGAGACCGGGGTCTCTGAGCCTCCTGCAGATCATGGACGTGGATGAGAAGAACCAAGTTTTAACCACCAACATTTGGCTGCAAATGTCTTGGACAGATCACTATTTACAGTGGAATGTGTCAGAATATCCAGGGGTGAAGACTGTTCGTTTCCCAGATGGCCAGATTTGGAAACCAGACATTCTTCGGAGACGGCTATATCCG
nachra7_Frag171-336_insertion174	GGGTGGGTACGTCTCCCCTGAGCAGCGGGAGACCGGGGTCTCTGAGCCTCCTGCAGATCATGGACGTGGATGAGAAGAACCAAGTTTTAACCACCAACATTTGGCTGCAAATGTCTTGGACAGATCACTATTTACAGTGGAATGTGTCAGAATATCCAGGGGTGAAGACTGTTCGTTTCCCAGATGGCCAGATTTGGAAACCAGACATTCTTCGGAGACGGCTATATCCG
nachra7_Frag171-336_insertion177	GGGTGGGTACGTCTCCCCTGAGCCTCAGCGGGAGACCGGGGTCTCTGAGCCTGCAGATCATGGACGTGGATGAGAAGAACCAAGTTTTAACCACCAACATTTGGCTGCAAATGTCTTGGACAGATCACTATTTACAGTGGAATGTGTCAGAATATCCAGGGGTGAAGACTGTTCGTTTCCCAGATGGCCAGATTTGGAAACCAGACATTCTTCGGAGACGGCTATATCCG
nachra7_Frag171-336_insertion180	GGGTGGGTACGTCTCCCCTGAGCCTCCTGAGCGGGAGACCGGGGTCTCTGAGCCAGATCATGGACGTGGATGAGAAGAACCAAGTTTTAACCACCAACATTTGGCTGCAAATGTCTTGGACAGATCACTATTTACAGTGGAATGTGTCAGAATATCCAGGGGTGAAGACTGTTCGTTTCCCAGATGGCCAGATTTGGAAACCAGACATTCTTCGGAGACGGCTATATCCG
nachra7_Frag171-336_insertion183	GGGTGGGTACGTCTCCCCTGAGCCTCCTGCAGAGCGGGAGACCGGGGTCTCTGAGCATCATGGACGTGGATGAGAAGAACCAAGTTTTAACCACCAACATTTGGCTGCAAATGTCTTGGACAGATCACTATTTACAGTGGAATGTGTCAGAATATCCAGGGGTGAAGACTGTTCGTTTCCCAGATGGCCAGATTTGGAAACCAGACATTCTTCGGAGACGGCTATATCCG
nachra7_Frag171-336_insertion186	GGGTGGGTACGTCTCCCCTGAGCCTCCTGCAGATCAGCGGGAGACCGGGGTCTCTGAGCATGGACGTGGATGAGAAGAACCAAGTTTTAACCACCAACATTTGGCTGCAAATGTCTTGGACAGATCACTATTTACAGTGGAATGTGTCAGAATATCCAGGGGTGAAGACTGTTCGTTTCCCAGATGGCCAGATTTGGAAACCAGACATTCTTCGGAGACGGCTATATCCG
nachra7_Frag171-336_insertion189	GGGTGGGTACGTCTCCCCTGAGCCTCCTGCAGATCATGAGCGGGAGACCGGGGTCTCTGAGCGACGTGGATGAGAAGAACCAAGTTTTAACCACCAACATTTGGCTGCAAATGTCTTGGACAGATCACTATTTACAGTGGAATGTGTCAGAATATCCAGGGGTGAAGACTGTTCGTTTCCCAGATGGCCAGATTTGGAAACCAGACATTCTTCGGAGACGGCTATATCCG
nachra7_Frag171-336_insertion192	GGGTGGGTACGTCTCCCCTGAGCCTCCTGCAGATCATGGACAGCGGGAGACCGGGGTCTCTGAGCGTGGATGAGAAGAACCAAGTTTTAACCACCAACATTTGGCTGCAAATGTCTTGGACAGATCACTATTTACAGTGGAATGTGTCAGAATATCCAGGGGTGAAGACTGTTCGTTTCCCAGATGGCCAGATTTGGAAACCAGACATTCTTCGGAGACGGCTATATCCG
nachra7_Frag171-336_insertion195	GGGTGGGTACGTCTCCCCTGAGCCTCCTGCAGATCATGGACGTGAGCGGGAGACCGGGGTCTCTGAGCGATGAGAAGAACCAAGTTTTAACCACCAACATTTGGCTGCAAATGTCTTGGACAGATCACTATTTACAGTGGAATGTGTCAGAATATCCAGGGGTGAAGACTGTTCGTTTCCCAGATGGCCAGATTTGGAAACCAGACATTCTTCGGAGACGGCTATATCCG
nachra7_Frag171-336_insertion198	GGGTGGGTACGTCTCCCCTGAGCCTCCTGCAGATCATGGACGTGGATAGCGGGAGACCGGGGTCTCTGAGCGAGAAGAACCAAGTTTTAACCACCAACATTTGGCTGCAAATGTCTTGGACAGATCACTATTTACAGTGGAATGTGTCAGAATATCCAGGGGTGAAGACTGTTCGTTTCCCAGATGGCCAGATTTGGAAACCAGACATTCTTCGGAGACGGCTATATCCG
nachra7_Frag171-336_insertion201	GGGTGGGTACGTCTCCCCTGAGCCTCCTGCAGATCATGGACGTGGATGAGAGCGGGAGACCGGGGTCTCTGAGCAAGAACCAAGTTTTAACCACCAACATTTGGCTGCAAATGTCTTGGACAGATCACTATTTACAGTGGAATGTGTCAGAATATCCAGGGGTGAAGACTGTTCGTTTCCCAGATGGCCAGATTTGGAAACCAGACATTCTTCGGAGACGGCTATATCCG
nachra7_Frag171-336_insertion204	GGGTGGGTACGTCTCCCCTGAGCCTCCTGCAGATCATGGACGTGGATGAGAAGAGCGGGAGACCGGGGTCTCTGAGCAACCAAGTTTTAACCACCAACATTTGGCTGCAAATGTCTTGGACAGATCACTATTTACAGTGGAATGTGTCAGAATATCCAGGGGTGAAGACTGTTCGTTTCCCAGATGGCCAGATTTGGAAACCAGACATTCTTCGGAGACGGCTATATCCG
nachra7_Frag171-336_insertion207	GGGTGGGTACGTCTCCCCTGAGCCTCCTGCAGATCATGGACGTGGATGAGAAGAACAGCGGGAGACCGGGGTCTCTGAGCCAAGTTTTAACCACCAACATTTGGCTGCAAATGTCTTGGACAGATCACTATTTACAGTGGAATGTGTCAGAATATCCAGGGGTGAAGACTGTTCGTTTCCCAGATGGCCAGATTTGGAAACCAGACATTCTTCGGAGACGGCTATATCCG
nachra7_Frag171-336_insertion210	GGGTGGGTACGTCTCCCCTGAGCCTCCTGCAGATCATGGACGTGGATGAGAAGAACCAAAGCGGGAGACCGGGGTCTCTGAGCGTTTTAACCACCAACATTTGGCTGCAAATGTCTTGGACAGATCACTATTTACAGTGGAATGTGTCAGAATATCCAGGGGTGAAGACTGTTCGTTTCCCAGATGGCCAGATTTGGAAACCAGACATTCTTCGGAGACGGCTATATCCG
nachra7_Frag171-336_insertion213	GGGTGGGTACGTCTCCCCTGAGCCTCCTGCAGATCATGGACGTGGATGAGAAGAACCAAGTTAGCGGGAGACCGGGGTCTCTGAGCTTAACCACCAACATTTGGCTGCAAATGTCTTGGACAGATCACTATTTACAGTGGAATGTGTCAGAATATCCAGGGGTGAAGACTGTTCGTTTCCCAGATGGCCAGATTTGGAAACCAGACATTCTTCGGAGACGGCTATATCCG
nachra7_Frag171-336_insertion216	GGGTGGGTACGTCTCCCCTGAGCCTCCTGCAGATCATGGACGTGGATGAGAAGAACCAAGTTTTAAGCGGGAGACCGGGGTCTCTGAGCACCACCAACATTTGGCTGCAAATGTCTTGGACAGATCACTATTTACAGTGGAATGTGTCAGAATATCCAGGGGTGAAGACTGTTCGTTTCCCAGATGGCCAGATTTGGAAACCAGACATTCTTCGGAGACGGCTATATCCG
nachra7_Frag171-336_insertion219	GGGTGGGTACGTCTCCCCTGAGCCTCCTGCAGATCATGGACGTGGATGAGAAGAACCAAGTTTTAACCAGCGGGAGACCGGGGTCTCTGAGCACCAACATTTGGCTGCAAATGTCTTGGACAGATCACTATTTACAGTGGAATGTGTCAGAATATCCAGGGGTGAAGACTGTTCGTTTCCCAGATGGCCAGATTTGGAAACCAGACATTCTTCGGAGACGGCTATATCCG
nachra7_Frag171-336_insertion222	GGGTGGGTACGTCTCCCCTGAGCCTCCTGCAGATCATGGACGTGGATGAGAAGAACCAAGTTTTAACCACCAGCGGGAGACCGGGGTCTCTGAGCAACATTTGGCTGCAAATGTCTTGGACAGATCACTATTTACAGTGGAATGTGTCAGAATATCCAGGGGTGAAGACTGTTCGTTTCCCAGATGGCCAGATTTGGAAACCAGACATTCTTCGGAGACGGCTATATCCG
nachra7_Frag171-336_insertion225	GGGTGGGTACGTCTCCCCTGAGCCTCCTGCAGATCATGGACGTGGATGAGAAGAACCAAGTTTTAACCACCAACAGCGGGAGACCGGGGTCTCTGAGCATTTGGCTGCAAATGTCTTGGACAGATCACTATTTACAGTGGAATGTGTCAGAATATCCAGGGGTGAAGACTGTTCGTTTCCCAGATGGCCAGATTTGGAAACCAGACATTCTTCGGAGACGGCTATATCCG
nachra7_Frag171-336_insertion228	GGGTGGGTACGTCTCCCCTGAGCCTCCTGCAGATCATGGACGTGGATGAGAAGAACCAAGTTTTAACCACCAACATTAGCGGGAGACCGGGGTCTCTGAGCTGGCTGCAAATGTCTTGGACAGATCACTATTTACAGTGGAATGTGTCAGAATATCCAGGGGTGAAGACTGTTCGTTTCCCAGATGGCCAGATTTGGAAACCAGACATTCTTCGGAGACGGCTATATCCG
nachra7_Frag171-336_insertion231	GGGTGGGTACGTCTCCCCTGAGCCTCCTGCAGATCATGGACGTGGATGAGAAGAACCAAGTTTTAACCACCAACATTTGGAGCGGGAGACCGGGGTCTCTGAGCCTGCAAATGTCTTGGACAGATCACTATTTACAGTGGAATGTGTCAGAATATCCAGGGGTGAAGACTGTTCGTTTCCCAGATGGCCAGATTTGGAAACCAGACATTCTTCGGAGACGGCTATATCCG
nachra7_Frag171-336_insertion234	GGGTGGGTACGTCTCCCCTGAGCCTCCTGCAGATCATGGACGTGGATGAGAAGAACCAAGTTTTAACCACCAACATTTGGCTGAGCGGGAGACCGGGGTCTCTGAGCCAAATGTCTTGGACAGATCACTATTTACAGTGGAATGTGTCAGAATATCCAGGGGTGAAGACTGTTCGTTTCCCAGATGGCCAGATTTGGAAACCAGACATTCTTCGGAGACGGCTATATCCG
nachra7_Frag171-336_insertion237	GGGTGGGTACGTCTCCCCTGAGCCTCCTGCAGATCATGGACGTGGATGAGAAGAACCAAGTTTTAACCACCAACATTTGGCTGCAAAGCGGGAGACCGGGGTCTCTGAGCATGTCTTGGACAGATCACTATTTACAGTGGAATGTGTCAGAATATCCAGGGGTGAAGACTGTTCGTTTCCCAGATGGCCAGATTTGGAAACCAGACATTCTTCGGAGACGGCTATATCCG
nachra7_Frag171-336_insertion240	GGGTGGGTACGTCTCCCCTGAGCCTCCTGCAGATCATGGACGTGGATGAGAAGAACCAAGTTTTAACCACCAACATTTGGCTGCAAATGAGCGGGAGACCGGGGTCTCTGAGCTCTTGGACAGATCACTATTTACAGTGGAATGTGTCAGAATATCCAGGGGTGAAGACTGTTCGTTTCCCAGATGGCCAGATTTGGAAACCAGACATTCTTCGGAGACGGCTATATCCG
nachra7_Frag171-336_insertion243	GGGTGGGTACGTCTCCCCTGAGCCTCCTGCAGATCATGGACGTGGATGAGAAGAACCAAGTTTTAACCACCAACATTTGGCTGCAAATGTCTAGCGGGAGACCGGGGTCTCTGAGCTGGACAGATCACTATTTACAGTGGAATGTGTCAGAATATCCAGGGGTGAAGACTGTTCGTTTCCCAGATGGCCAGATTTGGAAACCAGACATTCTTCGGAGACGGCTATATCCG
nachra7_Frag171-336_insertion246	GGGTGGGTACGTCTCCCCTGAGCCTCCTGCAGATCATGGACGTGGATGAGAAGAACCAAGTTTTAACCACCAACATTTGGCTGCAAATGTCTTGGAGCGGGAGACCGGGGTCTCTGAGCACAGATCACTATTTACAGTGGAATGTGTCAGAATATCCAGGGGTGAAGACTGTTCGTTTCCCAGATGGCCAGATTTGGAAACCAGACATTCTTCGGAGACGGCTATATCCG
nachra7_Frag171-336_insertion249	GGGTGGGTACGTCTCCCCTGAGCCTCCTGCAGATCATGGACGTGGATGAGAAGAACCAAGTTTTAACCACCAACATTTGGCTGCAAATGTCTTGGACAAGCGGGAGACCGGGGTCTCTGAGCGATCACTATTTACAGTGGAATGTGTCAGAATATCCAGGGGTGAAGACTGTTCGTTTCCCAGATGGCCAGATTTGGAAACCAGACATTCTTCGGAGACGGCTATATCCG
nachra7_Frag171-336_insertion252	GGGTGGGTACGTCTCCCCTGAGCCTCCTGCAGATCATGGACGTGGATGAGAAGAACCAAGTTTTAACCACCAACATTTGGCTGCAAATGTCTTGGACAGATAGCGGGAGACCGGGGTCTCTGAGCCACTATTTACAGTGGAATGTGTCAGAATATCCAGGGGTGAAGACTGTTCGTTTCCCAGATGGCCAGATTTGGAAACCAGACATTCTTCGGAGACGGCTATATCCG
nachra7_Frag171-336_insertion255	GGGTGGGTACGTCTCCCCTGAGCCTCCTGCAGATCATGGACGTGGATGAGAAGAACCAAGTTTTAACCACCAACATTTGGCTGCAAATGTCTTGGACAGATCACAGCGGGAGACCGGGGTCTCTGAGCTATTTACAGTGGAATGTGTCAGAATATCCAGGGGTGAAGACTGTTCGTTTCCCAGATGGCCAGATTTGGAAACCAGACATTCTTCGGAGACGGCTATATCCG
nachra7_Frag171-336_insertion258	GGGTGGGTACGTCTCCCCTGAGCCTCCTGCAGATCATGGACGTGGATGAGAAGAACCAAGTTTTAACCACCAACATTTGGCTGCAAATGTCTTGGACAGATCACTATAGCGGGAGACCGGGGTCTCTGAGCTTACAGTGGAATGTGTCAGAATATCCAGGGGTGAAGACTGTTCGTTTCCCAGATGGCCAGATTTGGAAACCAGACATTCTTCGGAGACGGCTATATCCG
nachra7_Frag171-336_insertion261	GGGTGGGTACGTCTCCCCTGAGCCTCCTGCAGATCATGGACGTGGATGAGAAGAACCAAGTTTTAACCACCAACATTTGGCTGCAAATGTCTTGGACAGATCACTATTTAAGCGGGAGACCGGGGTCTCTGAGCCAGTGGAATGTGTCAGAATATCCAGGGGTGAAGACTGTTCGTTTCCCAGATGGCCAGATTTGGAAACCAGACATTCTTCGGAGACGGCTATATCCG
nachra7_Frag171-336_insertion264	GGGTGGGTACGTCTCCCCTGAGCCTCCTGCAGATCATGGACGTGGATGAGAAGAACCAAGTTTTAACCACCAACATTTGGCTGCAAATGTCTTGGACAGATCACTATTTACAGAGCGGGAGACCGGGGTCTCTGAGCTGGAATGTGTCAGAATATCCAGGGGTGAAGACTGTTCGTTTCCCAGATGGCCAGATTTGGAAACCAGACATTCTTCGGAGACGGCTATATCCG
nachra7_Frag171-336_insertion267	GGGTGGGTACGTCTCCCCTGAGCCTCCTGCAGATCATGGACGTGGATGAGAAGAACCAAGTTTTAACCACCAACATTTGGCTGCAAATGTCTTGGACAGATCACTATTTACAGTGGAGCGGGAGACCGGGGTCTCTGAGCAATGTGTCAGAATATCCAGGGGTGAAGACTGTTCGTTTCCCAGATGGCCAGATTTGGAAACCAGACATTCTTCGGAGACGGCTATATCCG
nachra7_Frag171-336_insertion270	GGGTGGGTACGTCTCCCCTGAGCCTCCTGCAGATCATGGACGTGGATGAGAAGAACCAAGTTTTAACCACCAACATTTGGCTGCAAATGTCTTGGACAGATCACTATTTACAGTGGAATAGCGGGAGACCGGGGTCTCTGAGCGTGTCAGAATATCCAGGGGTGAAGACTGTTCGTTTCCCAGATGGCCAGATTTGGAAACCAGACATTCTTCGGAGACGGCTATATCCG
nachra7_Frag171-336_insertion273	GGGTGGGTACGTCTCCCCTGAGCCTCCTGCAGATCATGGACGTGGATGAGAAGAACCAAGTTTTAACCACCAACATTTGGCTGCAAATGTCTTGGACAGATCACTATTTACAGTGGAATGTGAGCGGGAGACCGGGGTCTCTGAGCTCAGAATATCCAGGGGTGAAGACTGTTCGTTTCCCAGATGGCCAGATTTGGAAACCAGACATTCTTCGGAGACGGCTATATCCG
nachra7_Frag171-336_insertion276	GGGTGGGTACGTCTCCCCTGAGCCTCCTGCAGATCATGGACGTGGATGAGAAGAACCAAGTTTTAACCACCAACATTTGGCTGCAAATGTCTTGGACAGATCACTATTTACAGTGGAATGTGTCAAGCGGGAGACCGGGGTCTCTGAGCGAATATCCAGGGGTGAAGACTGTTCGTTTCCCAGATGGCCAGATTTGGAAACCAGACATTCTTCGGAGACGGCTATATCCG
nachra7_Frag171-336_insertion279	GGGTGGGTACGTCTCCCCTGAGCCTCCTGCAGATCATGGACGTGGATGAGAAGAACCAAGTTTTAACCACCAACATTTGGCTGCAAATGTCTTGGACAGATCACTATTTACAGTGGAATGTGTCAGAAAGCGGGAGACCGGGGTCTCTGAGCTATCCAGGGGTGAAGACTGTTCGTTTCCCAGATGGCCAGATTTGGAAACCAGACATTCTTCGGAGACGGCTATATCCG
nachra7_Frag171-336_insertion282	GGGTGGGTACGTCTCCCCTGAGCCTCCTGCAGATCATGGACGTGGATGAGAAGAACCAAGTTTTAACCACCAACATTTGGCTGCAAATGTCTTGGACAGATCACTATTTACAGTGGAATGTGTCAGAATATAGCGGGAGACCGGGGTCTCTGAGCCCAGGGGTGAAGACTGTTCGTTTCCCAGATGGCCAGATTTGGAAACCAGACATTCTTCGGAGACGGCTATATCCG
nachra7_Frag171-336_insertion285	GGGTGGGTACGTCTCCCCTGAGCCTCCTGCAGATCATGGACGTGGATGAGAAGAACCAAGTTTTAACCACCAACATTTGGCTGCAAATGTCTTGGACAGATCACTATTTACAGTGGAATGTGTCAGAATATCCAAGCGGGAGACCGGGGTCTCTGAGCGGGGTGAAGACTGTTCGTTTCCCAGATGGCCAGATTTGGAAACCAGACATTCTTCGGAGACGGCTATATCCG
nachra7_Frag171-336_insertion288	GGGTGGGTACGTCTCCCCTGAGCCTCCTGCAGATCATGGACGTGGATGAGAAGAACCAAGTTTTAACCACCAACATTTGGCTGCAAATGTCTTGGACAGATCACTATTTACAGTGGAATGTGTCAGAATATCCAGGGAGCGGGAGACCGGGGTCTCTGAGCGTGAAGACTGTTCGTTTCCCAGATGGCCAGATTTGGAAACCAGACATTCTTCGGAGACGGCTATATCCG
nachra7_Frag171-336_insertion291	GGGTGGGTACGTCTCCCCTGAGCCTCCTGCAGATCATGGACGTGGATGAGAAGAACCAAGTTTTAACCACCAACATTTGGCTGCAAATGTCTTGGACAGATCACTATTTACAGTGGAATGTGTCAGAATATCCAGGGGTGAGCGGGAGACCGGGGTCTCTGAGCAAGACTGTTCGTTTCCCAGATGGCCAGATTTGGAAACCAGACATTCTTCGGAGACGGCTATATCCG
nachra7_Frag171-336_insertion294	GGGTGGGTACGTCTCCCCTGAGCCTCCTGCAGATCATGGACGTGGATGAGAAGAACCAAGTTTTAACCACCAACATTTGGCTGCAAATGTCTTGGACAGATCACTATTTACAGTGGAATGTGTCAGAATATCCAGGGGTGAAGAGCGGGAGACCGGGGTCTCTGAGCACTGTTCGTTTCCCAGATGGCCAGATTTGGAAACCAGACATTCTTCGGAGACGGCTATATCCG
nachra7_Frag171-336_insertion297	GGGTGGGTACGTCTCCCCTGAGCCTCCTGCAGATCATGGACGTGGATGAGAAGAACCAAGTTTTAACCACCAACATTTGGCTGCAAATGTCTTGGACAGATCACTATTTACAGTGGAATGTGTCAGAATATCCAGGGGTGAAGACTAGCGGGAGACCGGGGTCTCTGAGCGTTCGTTTCCCAGATGGCCAGATTTGGAAACCAGACATTCTTCGGAGACGGCTATATCCG
nachra7_Frag171-336_insertion300	GGGTGGGTACGTCTCCCCTGAGCCTCCTGCAGATCATGGACGTGGATGAGAAGAACCAAGTTTTAACCACCAACATTTGGCTGCAAATGTCTTGGACAGATCACTATTTACAGTGGAATGTGTCAGAATATCCAGGGGTGAAGACTGTTAGCGGGAGACCGGGGTCTCTGAGCCGTTTCCCAGATGGCCAGATTTGGAAACCAGACATTCTTCGGAGACGGCTATATCCG
nachra7_Frag171-336_insertion303	GGGTGGGTACGTCTCCCCTGAGCCTCCTGCAGATCATGGACGTGGATGAGAAGAACCAAGTTTTAACCACCAACATTTGGCTGCAAATGTCTTGGACAGATCACTATTTACAGTGGAATGTGTCAGAATATCCAGGGGTGAAGACTGTTCGTAGCGGGAGACCGGGGTCTCTGAGCTTCCCAGATGGCCAGATTTGGAAACCAGACATTCTTCGGAGACGGCTATATCCG
nachra7_Frag171-336_insertion306	GGGTGGGTACGTCTCCCCTGAGCCTCCTGCAGATCATGGACGTGGATGAGAAGAACCAAGTTTTAACCACCAACATTTGGCTGCAAATGTCTTGGACAGATCACTATTTACAGTGGAATGTGTCAGAATATCCAGGGGTGAAGACTGTTCGTTTCAGCGGGAGACCGGGGTCTCTGAGCCCAGATGGCCAGATTTGGAAACCAGACATTCTTCGGAGACGGCTATATCCG
nachra7_Frag171-336_insertion309	GGGTGGGTACGTCTCCCCTGAGCCTCCTGCAGATCATGGACGTGGATGAGAAGAACCAAGTTTTAACCACCAACATTTGGCTGCAAATGTCTTGGACAGATCACTATTTACAGTGGAATGTGTCAGAATATCCAGGGGTGAAGACTGTTCGTTTCCCAAGCGGGAGACCGGGGTCTCTGAGCGATGGCCAGATTTGGAAACCAGACATTCTTCGGAGACGGCTATATCCG
nachra7_Frag171-336_insertion312	GGGTGGGTACGTCTCCCCTGAGCCTCCTGCAGATCATGGACGTGGATGAGAAGAACCAAGTTTTAACCACCAACATTTGGCTGCAAATGTCTTGGACAGATCACTATTTACAGTGGAATGTGTCAGAATATCCAGGGGTGAAGACTGTTCGTTTCCCAGATAGCGGGAGACCGGGGTCTCTGAGCGGCCAGATTTGGAAACCAGACATTCTTCGGAGACGGCTATATCCG
nachra7_Frag171-336_insertion315	GGGTGGGTACGTCTCCCCTGAGCCTCCTGCAGATCATGGACGTGGATGAGAAGAACCAAGTTTTAACCACCAACATTTGGCTGCAAATGTCTTGGACAGATCACTATTTACAGTGGAATGTGTCAGAATATCCAGGGGTGAAGACTGTTCGTTTCCCAGATGGCAGCGGGAGACCGGGGTCTCTGAGCCAGATTTGGAAACCAGACATTCTTCGGAGACGGCTATATCCG
nachra7_Frag171-336_insertion318	GGGTGGGTACGTCTCCCCTGAGCCTCCTGCAGATCATGGACGTGGATGAGAAGAACCAAGTTTTAACCACCAACATTTGGCTGCAAATGTCTTGGACAGATCACTATTTACAGTGGAATGTGTCAGAATATCCAGGGGTGAAGACTGTTCGTTTCCCAGATGGCCAGAGCGGGAGACCGGGGTCTCTGAGCATTTGGAAACCAGACATTCTTCGGAGACGGCTATATCCG

nachra7_Frag171-336_insertion321	GGGTGGGTACGTCTCCCCTGAGCCTCCTGCAGATCATGGACGTGGATGAGAAGAACCAAGTTTTAACCACCAACATTTGGCTGCAAATGTCTTGGACAGATCACTATTTACAGTGGAATGTGTCAGAATATCCAGGGGTGAAGACTGTTCGTTTCCCAGATGGCCAGATTAGCGGGAGACCGGGGTCTCTGAGCTGGAAACCAGACATTCTTCGGAGACGGCTATATCCG
nachra7_Frag171-336_insertion324	GGGTGGGTACGTCTCCCCTGAGCCTCCTGCAGATCATGGACGTGGATGAGAAGAACCAAGTTTTAACCACCAACATTTGGCTGCAAATGTCTTGGACAGATCACTATTTACAGTGGAATGTGTCAGAATATCCAGGGGTGAAGACTGTTCGTTTCCCAGATGGCCAGATTTGGAGCGGGAGACCGGGGTCTCTGAGCAAACCAGACATTCTTCGGAGACGGCTATATCCG
nachra7_Frag171-336_insertion327	GGGTGGGTACGTCTCCCCTGAGCCTCCTGCAGATCATGGACGTGGATGAGAAGAACCAAGTTTTAACCACCAACATTTGGCTGCAAATGTCTTGGACAGATCACTATTTACAGTGGAATGTGTCAGAATATCCAGGGGTGAAGACTGTTCGTTTCCCAGATGGCCAGATTTGGAAAAGCGGGAGACCGGGGTCTCTGAGCCCAGACATTCTTCGGAGACGGCTATATCCG
nachra7_Frag171-336_insertion330	GGGTGGGTACGTCTCCCCTGAGCCTCCTGCAGATCATGGACGTGGATGAGAAGAACCAAGTTTTAACCACCAACATTTGGCTGCAAATGTCTTGGACAGATCACTATTTACAGTGGAATGTGTCAGAATATCCAGGGGTGAAGACTGTTCGTTTCCCAGATGGCCAGATTTGGAAACCAAGCGGGAGACCGGGGTCTCTGAGCGACATTCTTCGGAGACGGCTATATCCG
nachra7_Frag171-336_insertion333	GGGTGGGTACGTCTCCCCTGAGCCTCCTGCAGATCATGGACGTGGATGAGAAGAACCAAGTTTTAACCACCAACATTTGGCTGCAAATGTCTTGGACAGATCACTATTTACAGTGGAATGTGTCAGAATATCCAGGGGTGAAGACTGTTCGTTTCCCAGATGGCCAGATTTGGAAACCAGACAGCGGGAGACCGGGGTCTCTGAGCATTCTTCGGAGACGGCTATATCCG
nachra7_Frag171-336_insertion336	GGGTGGGTACGTCTCCCCTGAGCCTCCTGCAGATCATGGACGTGGATGAGAAGAACCAAGTTTTAACCACCAACATTTGGCTGCAAATGTCTTGGACAGATCACTATTTACAGTGGAATGTGTCAGAATATCCAGGGGTGAAGACTGTTCGTTTCCCAGATGGCCAGATTTGGAAACCAGACATTAGCGGGAGACCGGGGTCTCTGAGCCTTCGGAGACGGCTATATCCG
nachra7_Frag339-504_insertion339	TCCGACGGGCGTCTCCTCTTAGCGGGAGACCGGGGTCTCTGAGCCTCTATAACAGTGCTGATGAGCGCTTTGACGCCACATTCCACACTAACGTGTTGGTGAATTCTTCTGGGCATTGCCAGTACCTGCCTCCAGGCATATTCAAGAGTTCCTGCTACATCGATGTACGCTGGTTTCCCTTTGATGTGCAGCACTGCAAACTGAAGTTTGGGTGGAGACGTTGGCCTGAA
nachra7_Frag339-504_insertion342	TCCGACGGGCGTCTCCTCTTCTCAGCGGGAGACCGGGGTCTCTGAGCTATAACAGTGCTGATGAGCGCTTTGACGCCACATTCCACACTAACGTGTTGGTGAATTCTTCTGGGCATTGCCAGTACCTGCCTCCAGGCATATTCAAGAGTTCCTGCTACATCGATGTACGCTGGTTTCCCTTTGATGTGCAGCACTGCAAACTGAAGTTTGGGTGGAGACGTTGGCCTGAA
nachra7_Frag339-504_insertion345	TCCGACGGGCGTCTCCTCTTCTCTATAGCGGGAGACCGGGGTCTCTGAGCAACAGTGCTGATGAGCGCTTTGACGCCACATTCCACACTAACGTGTTGGTGAATTCTTCTGGGCATTGCCAGTACCTGCCTCCAGGCATATTCAAGAGTTCCTGCTACATCGATGTACGCTGGTTTCCCTTTGATGTGCAGCACTGCAAACTGAAGTTTGGGTGGAGACGTTGGCCTGAA
nachra7_Frag339-504_insertion348	TCCGACGGGCGTCTCCTCTTCTCTATAACAGCGGGAGACCGGGGTCTCTGAGCAGTGCTGATGAGCGCTTTGACGCCACATTCCACACTAACGTGTTGGTGAATTCTTCTGGGCATTGCCAGTACCTGCCTCCAGGCATATTCAAGAGTTCCTGCTACATCGATGTACGCTGGTTTCCCTTTGATGTGCAGCACTGCAAACTGAAGTTTGGGTGGAGACGTTGGCCTGAA
nachra7_Frag339-504_insertion351	TCCGACGGGCGTCTCCTCTTCTCTATAACAGTAGCGGGAGACCGGGGTCTCTGAGCGCTGATGAGCGCTTTGACGCCACATTCCACACTAACGTGTTGGTGAATTCTTCTGGGCATTGCCAGTACCTGCCTCCAGGCATATTCAAGAGTTCCTGCTACATCGATGTACGCTGGTTTCCCTTTGATGTGCAGCACTGCAAACTGAAGTTTGGGTGGAGACGTTGGCCTGAA
nachra7_Frag339-504_insertion354	TCCGACGGGCGTCTCCTCTTCTCTATAACAGTGCTAGCGGGAGACCGGGGTCTCTGAGCGATGAGCGCTTTGACGCCACATTCCACACTAACGTGTTGGTGAATTCTTCTGGGCATTGCCAGTACCTGCCTCCAGGCATATTCAAGAGTTCCTGCTACATCGATGTACGCTGGTTTCCCTTTGATGTGCAGCACTGCAAACTGAAGTTTGGGTGGAGACGTTGGCCTGAA
nachra7_Frag339-504_insertion357	TCCGACGGGCGTCTCCTCTTCTCTATAACAGTGCTGATAGCGGGAGACCGGGGTCTCTGAGCGAGCGCTTTGACGCCACATTCCACACTAACGTGTTGGTGAATTCTTCTGGGCATTGCCAGTACCTGCCTCCAGGCATATTCAAGAGTTCCTGCTACATCGATGTACGCTGGTTTCCCTTTGATGTGCAGCACTGCAAACTGAAGTTTGGGTGGAGACGTTGGCCTGAA
nachra7_Frag339-504_insertion360	TCCGACGGGCGTCTCCTCTTCTCTATAACAGTGCTGATGAGAGCGGGAGACCGGGGTCTCTGAGCCGCTTTGACGCCACATTCCACACTAACGTGTTGGTGAATTCTTCTGGGCATTGCCAGTACCTGCCTCCAGGCATATTCAAGAGTTCCTGCTACATCGATGTACGCTGGTTTCCCTTTGATGTGCAGCACTGCAAACTGAAGTTTGGGTGGAGACGTTGGCCTGAA
nachra7_Frag339-504_insertion363	TCCGACGGGCGTCTCCTCTTCTCTATAACAGTGCTGATGAGCGCAGCGGGAGACCGGGGTCTCTGAGCTTTGACGCCACATTCCACACTAACGTGTTGGTGAATTCTTCTGGGCATTGCCAGTACCTGCCTCCAGGCATATTCAAGAGTTCCTGCTACATCGATGTACGCTGGTTTCCCTTTGATGTGCAGCACTGCAAACTGAAGTTTGGGTGGAGACGTTGGCCTGAA
nachra7_Frag339-504_insertion366	TCCGACGGGCGTCTCCTCTTCTCTATAACAGTGCTGATGAGCGCTTTAGCGGGAGACCGGGGTCTCTGAGCGACGCCACATTCCACACTAACGTGTTGGTGAATTCTTCTGGGCATTGCCAGTACCTGCCTCCAGGCATATTCAAGAGTTCCTGCTACATCGATGTACGCTGGTTTCCCTTTGATGTGCAGCACTGCAAACTGAAGTTTGGGTGGAGACGTTGGCCTGAA
nachra7_Frag339-504_insertion369	TCCGACGGGCGTCTCCTCTTCTCTATAACAGTGCTGATGAGCGCTTTGACAGCGGGAGACCGGGGTCTCTGAGCGCCACATTCCACACTAACGTGTTGGTGAATTCTTCTGGGCATTGCCAGTACCTGCCTCCAGGCATATTCAAGAGTTCCTGCTACATCGATGTACGCTGGTTTCCCTTTGATGTGCAGCACTGCAAACTGAAGTTTGGGTGGAGACGTTGGCCTGAA
nachra7_Frag339-504_insertion372	TCCGACGGGCGTCTCCTCTTCTCTATAACAGTGCTGATGAGCGCTTTGACGCCAGCGGGAGACCGGGGTCTCTGAGCACATTCCACACTAACGTGTTGGTGAATTCTTCTGGGCATTGCCAGTACCTGCCTCCAGGCATATTCAAGAGTTCCTGCTACATCGATGTACGCTGGTTTCCCTTTGATGTGCAGCACTGCAAACTGAAGTTTGGGTGGAGACGTTGGCCTGAA
nachra7_Frag339-504_insertion375	TCCGACGGGCGTCTCCTCTTCTCTATAACAGTGCTGATGAGCGCTTTGACGCCACAAGCGGGAGACCGGGGTCTCTGAGCTTCCACACTAACGTGTTGGTGAATTCTTCTGGGCATTGCCAGTACCTGCCTCCAGGCATATTCAAGAGTTCCTGCTACATCGATGTACGCTGGTTTCCCTTTGATGTGCAGCACTGCAAACTGAAGTTTGGGTGGAGACGTTGGCCTGAA
nachra7_Frag339-504_insertion378	TCCGACGGGCGTCTCCTCTTCTCTATAACAGTGCTGATGAGCGCTTTGACGCCACATTCAGCGGGAGACCGGGGTCTCTGAGCCACACTAACGTGTTGGTGAATTCTTCTGGGCATTGCCAGTACCTGCCTCCAGGCATATTCAAGAGTTCCTGCTACATCGATGTACGCTGGTTTCCCTTTGATGTGCAGCACTGCAAACTGAAGTTTGGGTGGAGACGTTGGCCTGAA
nachra7_Frag339-504_insertion381	TCCGACGGGCGTCTCCTCTTCTCTATAACAGTGCTGATGAGCGCTTTGACGCCACATTCCACAGCGGGAGACCGGGGTCTCTGAGCACTAACGTGTTGGTGAATTCTTCTGGGCATTGCCAGTACCTGCCTCCAGGCATATTCAAGAGTTCCTGCTACATCGATGTACGCTGGTTTCCCTTTGATGTGCAGCACTGCAAACTGAAGTTTGGGTGGAGACGTTGGCCTGAA
nachra7_Frag339-504_insertion384	TCCGACGGGCGTCTCCTCTTCTCTATAACAGTGCTGATGAGCGCTTTGACGCCACATTCCACACTAGCGGGAGACCGGGGTCTCTGAGCAACGTGTTGGTGAATTCTTCTGGGCATTGCCAGTACCTGCCTCCAGGCATATTCAAGAGTTCCTGCTACATCGATGTACGCTGGTTTCCCTTTGATGTGCAGCACTGCAAACTGAAGTTTGGGTGGAGACGTTGGCCTGAA
nachra7_Frag339-504_insertion387	TCCGACGGGCGTCTCCTCTTCTCTATAACAGTGCTGATGAGCGCTTTGACGCCACATTCCACACTAACAGCGGGAGACCGGGGTCTCTGAGCGTGTTGGTGAATTCTTCTGGGCATTGCCAGTACCTGCCTCCAGGCATATTCAAGAGTTCCTGCTACATCGATGTACGCTGGTTTCCCTTTGATGTGCAGCACTGCAAACTGAAGTTTGGGTGGAGACGTTGGCCTGAA
nachra7_Frag339-504_insertion390	TCCGACGGGCGTCTCCTCTTCTCTATAACAGTGCTGATGAGCGCTTTGACGCCACATTCCACACTAACGTGAGCGGGAGACCGGGGTCTCTGAGCTTGGTGAATTCTTCTGGGCATTGCCAGTACCTGCCTCCAGGCATATTCAAGAGTTCCTGCTACATCGATGTACGCTGGTTTCCCTTTGATGTGCAGCACTGCAAACTGAAGTTTGGGTGGAGACGTTGGCCTGAA
nachra7_Frag339-504_insertion393	TCCGACGGGCGTCTCCTCTTCTCTATAACAGTGCTGATGAGCGCTTTGACGCCACATTCCACACTAACGTGTTGAGCGGGAGACCGGGGTCTCTGAGCGTGAATTCTTCTGGGCATTGCCAGTACCTGCCTCCAGGCATATTCAAGAGTTCCTGCTACATCGATGTACGCTGGTTTCCCTTTGATGTGCAGCACTGCAAACTGAAGTTTGGGTGGAGACGTTGGCCTGAA
nachra7_Frag339-504_insertion396	TCCGACGGGCGTCTCCTCTTCTCTATAACAGTGCTGATGAGCGCTTTGACGCCACATTCCACACTAACGTGTTGGTGAGCGGGAGACCGGGGTCTCTGAGCAATTCTTCTGGGCATTGCCAGTACCTGCCTCCAGGCATATTCAAGAGTTCCTGCTACATCGATGTACGCTGGTTTCCCTTTGATGTGCAGCACTGCAAACTGAAGTTTGGGTGGAGACGTTGGCCTGAA
nachra7_Frag339-504_insertion399	TCCGACGGGCGTCTCCTCTTCTCTATAACAGTGCTGATGAGCGCTTTGACGCCACATTCCACACTAACGTGTTGGTGAATAGCGGGAGACCGGGGTCTCTGAGCTCTTCTGGGCATTGCCAGTACCTGCCTCCAGGCATATTCAAGAGTTCCTGCTACATCGATGTACGCTGGTTTCCCTTTGATGTGCAGCACTGCAAACTGAAGTTTGGGTGGAGACGTTGGCCTGAA
nachra7_Frag339-504_insertion402	TCCGACGGGCGTCTCCTCTTCTCTATAACAGTGCTGATGAGCGCTTTGACGCCACATTCCACACTAACGTGTTGGTGAATTCTAGCGGGAGACCGGGGTCTCTGAGCTCTGGGCATTGCCAGTACCTGCCTCCAGGCATATTCAAGAGTTCCTGCTACATCGATGTACGCTGGTTTCCCTTTGATGTGCAGCACTGCAAACTGAAGTTTGGGTGGAGACGTTGGCCTGAA
nachra7_Frag339-504_insertion405	TCCGACGGGCGTCTCCTCTTCTCTATAACAGTGCTGATGAGCGCTTTGACGCCACATTCCACACTAACGTGTTGGTGAATTCTTCTAGCGGGAGACCGGGGTCTCTGAGCGGGCATTGCCAGTACCTGCCTCCAGGCATATTCAAGAGTTCCTGCTACATCGATGTACGCTGGTTTCCCTTTGATGTGCAGCACTGCAAACTGAAGTTTGGGTGGAGACGTTGGCCTGAA
nachra7_Frag339-504_insertion408	TCCGACGGGCGTCTCCTCTTCTCTATAACAGTGCTGATGAGCGCTTTGACGCCACATTCCACACTAACGTGTTGGTGAATTCTTCTGGGAGCGGGAGACCGGGGTCTCTGAGCCATTGCCAGTACCTGCCTCCAGGCATATTCAAGAGTTCCTGCTACATCGATGTACGCTGGTTTCCCTTTGATGTGCAGCACTGCAAACTGAAGTTTGGGTGGAGACGTTGGCCTGAA
nachra7_Frag339-504_insertion411	TCCGACGGGCGTCTCCTCTTCTCTATAACAGTGCTGATGAGCGCTTTGACGCCACATTCCACACTAACGTGTTGGTGAATTCTTCTGGGCATAGCGGGAGACCGGGGTCTCTGAGCTGCCAGTACCTGCCTCCAGGCATATTCAAGAGTTCCTGCTACATCGATGTACGCTGGTTTCCCTTTGATGTGCAGCACTGCAAACTGAAGTTTGGGTGGAGACGTTGGCCTGAA
nachra7_Frag339-504_insertion414	TCCGACGGGCGTCTCCTCTTCTCTATAACAGTGCTGATGAGCGCTTTGACGCCACATTCCACACTAACGTGTTGGTGAATTCTTCTGGGCATTGCAGCGGGAGACCGGGGTCTCTGAGCCAGTACCTGCCTCCAGGCATATTCAAGAGTTCCTGCTACATCGATGTACGCTGGTTTCCCTTTGATGTGCAGCACTGCAAACTGAAGTTTGGGTGGAGACGTTGGCCTGAA
nachra7_Frag339-504_insertion417	TCCGACGGGCGTCTCCTCTTCTCTATAACAGTGCTGATGAGCGCTTTGACGCCACATTCCACACTAACGTGTTGGTGAATTCTTCTGGGCATTGCCAGAGCGGGAGACCGGGGTCTCTGAGCTACCTGCCTCCAGGCATATTCAAGAGTTCCTGCTACATCGATGTACGCTGGTTTCCCTTTGATGTGCAGCACTGCAAACTGAAGTTTGGGTGGAGACGTTGGCCTGAA
nachra7_Frag339-504_insertion420	TCCGACGGGCGTCTCCTCTTCTCTATAACAGTGCTGATGAGCGCTTTGACGCCACATTCCACACTAACGTGTTGGTGAATTCTTCTGGGCATTGCCAGTACAGCGGGAGACCGGGGTCTCTGAGCCTGCCTCCAGGCATATTCAAGAGTTCCTGCTACATCGATGTACGCTGGTTTCCCTTTGATGTGCAGCACTGCAAACTGAAGTTTGGGTGGAGACGTTGGCCTGAA
nachra7_Frag339-504_insertion423	TCCGACGGGCGTCTCCTCTTCTCTATAACAGTGCTGATGAGCGCTTTGACGCCACATTCCACACTAACGTGTTGGTGAATTCTTCTGGGCATTGCCAGTACCTGAGCGGGAGACCGGGGTCTCTGAGCCCTCCAGGCATATTCAAGAGTTCCTGCTACATCGATGTACGCTGGTTTCCCTTTGATGTGCAGCACTGCAAACTGAAGTTTGGGTGGAGACGTTGGCCTGAA
nachra7_Frag339-504_insertion426	TCCGACGGGCGTCTCCTCTTCTCTATAACAGTGCTGATGAGCGCTTTGACGCCACATTCCACACTAACGTGTTGGTGAATTCTTCTGGGCATTGCCAGTACCTGCCTAGCGGGAGACCGGGGTCTCTGAGCCCAGGCATATTCAAGAGTTCCTGCTACATCGATGTACGCTGGTTTCCCTTTGATGTGCAGCACTGCAAACTGAAGTTTGGGTGGAGACGTTGGCCTGAA
nachra7_Frag339-504_insertion429	TCCGACGGGCGTCTCCTCTTCTCTATAACAGTGCTGATGAGCGCTTTGACGCCACATTCCACACTAACGTGTTGGTGAATTCTTCTGGGCATTGCCAGTACCTGCCTCCAAGCGGGAGACCGGGGTCTCTGAGCGGCATATTCAAGAGTTCCTGCTACATCGATGTACGCTGGTTTCCCTTTGATGTGCAGCACTGCAAACTGAAGTTTGGGTGGAGACGTTGGCCTGAA
nachra7_Frag339-504_insertion432	TCCGACGGGCGTCTCCTCTTCTCTATAACAGTGCTGATGAGCGCTTTGACGCCACATTCCACACTAACGTGTTGGTGAATTCTTCTGGGCATTGCCAGTACCTGCCTCCAGGCAGCGGGAGACCGGGGTCTCTGAGCATATTCAAGAGTTCCTGCTACATCGATGTACGCTGGTTTCCCTTTGATGTGCAGCACTGCAAACTGAAGTTTGGGTGGAGACGTTGGCCTGAA
nachra7_Frag339-504_insertion435	TCCGACGGGCGTCTCCTCTTCTCTATAACAGTGCTGATGAGCGCTTTGACGCCACATTCCACACTAACGTGTTGGTGAATTCTTCTGGGCATTGCCAGTACCTGCCTCCAGGCATAAGCGGGAGACCGGGGTCTCTGAGCTTCAAGAGTTCCTGCTACATCGATGTACGCTGGTTTCCCTTTGATGTGCAGCACTGCAAACTGAAGTTTGGGTGGAGACGTTGGCCTGAA
nachra7_Frag339-504_insertion438	TCCGACGGGCGTCTCCTCTTCTCTATAACAGTGCTGATGAGCGCTTTGACGCCACATTCCACACTAACGTGTTGGTGAATTCTTCTGGGCATTGCCAGTACCTGCCTCCAGGCATATTCAGCGGGAGACCGGGGTCTCTGAGCAAGAGTTCCTGCTACATCGATGTACGCTGGTTTCCCTTTGATGTGCAGCACTGCAAACTGAAGTTTGGGTGGAGACGTTGGCCTGAA
nachra7_Frag339-504_insertion441	TCCGACGGGCGTCTCCTCTTCTCTATAACAGTGCTGATGAGCGCTTTGACGCCACATTCCACACTAACGTGTTGGTGAATTCTTCTGGGCATTGCCAGTACCTGCCTCCAGGCATATTCAAGAGCGGGAGACCGGGGTCTCTGAGCAGTTCCTGCTACATCGATGTACGCTGGTTTCCCTTTGATGTGCAGCACTGCAAACTGAAGTTTGGGTGGAGACGTTGGCCTGAA
nachra7_Frag339-504_insertion444	TCCGACGGGCGTCTCCTCTTCTCTATAACAGTGCTGATGAGCGCTTTGACGCCACATTCCACACTAACGTGTTGGTGAATTCTTCTGGGCATTGCCAGTACCTGCCTCCAGGCATATTCAAGAGTAGCGGGAGACCGGGGTCTCTGAGCTCCTGCTACATCGATGTACGCTGGTTTCCCTTTGATGTGCAGCACTGCAAACTGAAGTTTGGGTGGAGACGTTGGCCTGAA
nachra7_Frag339-504_insertion447	TCCGACGGGCGTCTCCTCTTCTCTATAACAGTGCTGATGAGCGCTTTGACGCCACATTCCACACTAACGTGTTGGTGAATTCTTCTGGGCATTGCCAGTACCTGCCTCCAGGCATATTCAAGAGTTCCAGCGGGAGACCGGGGTCTCTGAGCTGCTACATCGATGTACGCTGGTTTCCCTTTGATGTGCAGCACTGCAAACTGAAGTTTGGGTGGAGACGTTGGCCTGAA
nachra7_Frag339-504_insertion450	TCCGACGGGCGTCTCCTCTTCTCTATAACAGTGCTGATGAGCGCTTTGACGCCACATTCCACACTAACGTGTTGGTGAATTCTTCTGGGCATTGCCAGTACCTGCCTCCAGGCATATTCAAGAGTTCCTGCAGCGGGAGACCGGGGTCTCTGAGCTACATCGATGTACGCTGGTTTCCCTTTGATGTGCAGCACTGCAAACTGAAGTTTGGGTGGAGACGTTGGCCTGAA
nachra7_Frag339-504_insertion453	TCCGACGGGCGTCTCCTCTTCTCTATAACAGTGCTGATGAGCGCTTTGACGCCACATTCCACACTAACGTGTTGGTGAATTCTTCTGGGCATTGCCAGTACCTGCCTCCAGGCATATTCAAGAGTTCCTGCTACAGCGGGAGACCGGGGTCTCTGAGCATCGATGTACGCTGGTTTCCCTTTGATGTGCAGCACTGCAAACTGAAGTTTGGGTGGAGACGTTGGCCTGAA
nachra7_Frag339-504_insertion456	TCCGACGGGCGTCTCCTCTTCTCTATAACAGTGCTGATGAGCGCTTTGACGCCACATTCCACACTAACGTGTTGGTGAATTCTTCTGGGCATTGCCAGTACCTGCCTCCAGGCATATTCAAGAGTTCCTGCTACATCAGCGGGAGACCGGGGTCTCTGAGCGATGTACGCTGGTTTCCCTTTGATGTGCAGCACTGCAAACTGAAGTTTGGGTGGAGACGTTGGCCTGAA
nachra7_Frag339-504_insertion459	TCCGACGGGCGTCTCCTCTTCTCTATAACAGTGCTGATGAGCGCTTTGACGCCACATTCCACACTAACGTGTTGGTGAATTCTTCTGGGCATTGCCAGTACCTGCCTCCAGGCATATTCAAGAGTTCCTGCTACATCGATAGCGGGAGACCGGGGTCTCTGAGCGTACGCTGGTTTCCCTTTGATGTGCAGCACTGCAAACTGAAGTTTGGGTGGAGACGTTGGCCTGAA
nachra7_Frag339-504_insertion462	TCCGACGGGCGTCTCCTCTTCTCTATAACAGTGCTGATGAGCGCTTTGACGCCACATTCCACACTAACGTGTTGGTGAATTCTTCTGGGCATTGCCAGTACCTGCCTCCAGGCATATTCAAGAGTTCCTGCTACATCGATGTAAGCGGGAGACCGGGGTCTCTGAGCCGCTGGTTTCCCTTTGATGTGCAGCACTGCAAACTGAAGTTTGGGTGGAGACGTTGGCCTGAA
nachra7_Frag339-504_insertion465	TCCGACGGGCGTCTCCTCTTCTCTATAACAGTGCTGATGAGCGCTTTGACGCCACATTCCACACTAACGTGTTGGTGAATTCTTCTGGGCATTGCCAGTACCTGCCTCCAGGCATATTCAAGAGTTCCTGCTACATCGATGTACGCAGCGGGAGACCGGGGTCTCTGAGCTGGTTTCCCTTTGATGTGCAGCACTGCAAACTGAAGTTTGGGTGGAGACGTTGGCCTGAA
nachra7_Frag339-504_insertion468	TCCGACGGGCGTCTCCTCTTCTCTATAACAGTGCTGATGAGCGCTTTGACGCCACATTCCACACTAACGTGTTGGTGAATTCTTCTGGGCATTGCCAGTACCTGCCTCCAGGCATATTCAAGAGTTCCTGCTACATCGATGTACGCTGGAGCGGGAGACCGGGGTCTCTGAGCTTTCCCTTTGATGTGCAGCACTGCAAACTGAAGTTTGGGTGGAGACGTTGGCCTGAA
nachra7_Frag339-504_insertion471	TCCGACGGGCGTCTCCTCTTCTCTATAACAGTGCTGATGAGCGCTTTGACGCCACATTCCACACTAACGTGTTGGTGAATTCTTCTGGGCATTGCCAGTACCTGCCTCCAGGCATATTCAAGAGTTCCTGCTACATCGATGTACGCTGGTTTAGCGGGAGACCGGGGTCTCTGAGCCCCTTTGATGTGCAGCACTGCAAACTGAAGTTTGGGTGGAGACGTTGGCCTGAA
nachra7_Frag339-504_insertion474	TCCGACGGGCGTCTCCTCTTCTCTATAACAGTGCTGATGAGCGCTTTGACGCCACATTCCACACTAACGTGTTGGTGAATTCTTCTGGGCATTGCCAGTACCTGCCTCCAGGCATATTCAAGAGTTCCTGCTACATCGATGTACGCTGGTTTCCCAGCGGGAGACCGGGGTCTCTGAGCTTTGATGTGCAGCACTGCAAACTGAAGTTTGGGTGGAGACGTTGGCCTGAA
nachra7_Frag339-504_insertion477	TCCGACGGGCGTCTCCTCTTCTCTATAACAGTGCTGATGAGCGCTTTGACGCCACATTCCACACTAACGTGTTGGTGAATTCTTCTGGGCATTGCCAGTACCTGCCTCCAGGCATATTCAAGAGTTCCTGCTACATCGATGTACGCTGGTTTCCCTTTAGCGGGAGACCGGGGTCTCTGAGCGATGTGCAGCACTGCAAACTGAAGTTTGGGTGGAGACGTTGGCCTGAA
nachra7_Frag339-504_insertion480	TCCGACGGGCGTCTCCTCTTCTCTATAACAGTGCTGATGAGCGCTTTGACGCCACATTCCACACTAACGTGTTGGTGAATTCTTCTGGGCATTGCCAGTACCTGCCTCCAGGCATATTCAAGAGTTCCTGCTACATCGATGTACGCTGGTTTCCCTTTGATAGCGGGAGACCGGGGTCTCTGAGCGTGCAGCACTGCAAACTGAAGTTTGGGTGGAGACGTTGGCCTGAA
nachra7_Frag339-504_insertion483	TCCGACGGGCGTCTCCTCTTCTCTATAACAGTGCTGATGAGCGCTTTGACGCCACATTCCACACTAACGTGTTGGTGAATTCTTCTGGGCATTGCCAGTACCTGCCTCCAGGCATATTCAAGAGTTCCTGCTACATCGATGTACGCTGGTTTCCCTTTGATGTGAGCGGGAGACCGGGGTCTCTGAGCCAGCACTGCAAACTGAAGTTTGGGTGGAGACGTTGGCCTGAA
nachra7_Frag339-504_insertion486	TCCGACGGGCGTCTCCTCTTCTCTATAACAGTGCTGATGAGCGCTTTGACGCCACATTCCACACTAACGTGTTGGTGAATTCTTCTGGGCATTGCCAGTACCTGCCTCCAGGCATATTCAAGAGTTCCTGCTACATCGATGTACGCTGGTTTCCCTTTGATGTGCAGAGCGGGAGACCGGGGTCTCTGAGCCACTGCAAACTGAAGTTTGGGTGGAGACGTTGGCCTGAA
nachra7_Frag339-504_insertion489	TCCGACGGGCGTCTCCTCTTCTCTATAACAGTGCTGATGAGCGCTTTGACGCCACATTCCACACTAACGTGTTGGTGAATTCTTCTGGGCATTGCCAGTACCTGCCTCCAGGCATATTCAAGAGTTCCTGCTACATCGATGTACGCTGGTTTCCCTTTGATGTGCAGCACAGCGGGAGACCGGGGTCTCTGAGCTGCAAACTGAAGTTTGGGTGGAGACGTTGGCCTGAA
nachra7_Frag339-504_insertion492	TCCGACGGGCGTCTCCTCTTCTCTATAACAGTGCTGATGAGCGCTTTGACGCCACATTCCACACTAACGTGTTGGTGAATTCTTCTGGGCATTGCCAGTACCTGCCTCCAGGCATATTCAAGAGTTCCTGCTACATCGATGTACGCTGGTTTCCCTTTGATGTGCAGCACTGCAGCGGGAGACCGGGGTCTCTGAGCAAACTGAAGTTTGGGTGGAGACGTTGGCCTGAA
nachra7_Frag339-504_insertion495	TCCGACGGGCGTCTCCTCTTCTCTATAACAGTGCTGATGAGCGCTTTGACGCCACATTCCACACTAACGTGTTGGTGAATTCTTCTGGGCATTGCCAGTACCTGCCTCCAGGCATATTCAAGAGTTCCTGCTACATCGATGTACGCTGGTTTCCCTTTGATGTGCAGCACTGCAAAAGCGGGAGACCGGGGTCTCTGAGCCTGAAGTTTGGGTGGAGACGTTGGCCTGAA
nachra7_Frag339-504_insertion498	TCCGACGGGCGTCTCCTCTTCTCTATAACAGTGCTGATGAGCGCTTTGACGCCACATTCCACACTAACGTGTTGGTGAATTCTTCTGGGCATTGCCAGTACCTGCCTCCAGGCATATTCAAGAGTTCCTGCTACATCGATGTACGCTGGTTTCCCTTTGATGTGCAGCACTGCAAACTGAGCGGGAGACCGGGGTCTCTGAGCAAGTTTGGGTGGAGACGTTGGCCTGAA
nachra7_Frag339-504_insertion501	TCCGACGGGCGTCTCCTCTTCTCTATAACAGTGCTGATGAGCGCTTTGACGCCACATTCCACACTAACGTGTTGGTGAATTCTTCTGGGCATTGCCAGTACCTGCCTCCAGGCATATTCAAGAGTTCCTGCTACATCGATGTACGCTGGTTTCCCTTTGATGTGCAGCACTGCAAACTGAAGAGCGGGAGACCGGGGTCTCTGAGCTTTGGGTGGAGACGTTGGCCTGAA
nachra7_Frag339-504_insertion504	TCCGACGGGCGTCTCCTCTTCTCTATAACAGTGCTGATGAGCGCTTTGACGCCACATTCCACACTAACGTGTTGGTGAATTCTTCTGGGCATTGCCAGTACCTGCCTCCAGGCATATTCAAGAGTTCCTGCTACATCGATGTACGCTGGTTTCCCTTTGATGTGCAGCACTGCAAACTGAAGTTTAGCGGGAGACCGGGGTCTCTGAGCGGGTGGAGACGTTGGCCTGAA
nachra7_Frag507-672_insertion507	CATGTTTAGCGTCTCCTGGGAGCGGGAGACCGGGGTCTCTGAGCTCCTGGTCTTACGGAGGCTGGTCCTTGGATCTGCAGATGCAGGAGGCAGATATCAGTGGCTATATCCCCAATGGAGAATGGGACCTAGTGGGAATCCCCGGCAAGAGGAGTGAAAGGTTCTATGAGTGCTGCAAAGAGCCCTACCCCGATGTCACCTTCACAGTGACCAGGAGACGGGGAGGGAAC
nachra7_Frag507-672_insertion510	CATGTTTAGCGTCTCCTGGGTCCAGCGGGAGACCGGGGTCTCTGAGCTGGTCTTACGGAGGCTGGTCCTTGGATCTGCAGATGCAGGAGGCAGATATCAGTGGCTATATCCCCAATGGAGAATGGGACCTAGTGGGAATCCCCGGCAAGAGGAGTGAAAGGTTCTATGAGTGCTGCAAAGAGCCCTACCCCGATGTCACCTTCACAGTGACCAGGAGACGGGGAGGGAAC
nachra7_Frag507-672_insertion513	CATGTTTAGCGTCTCCTGGGTCCTGGAGCGGGAGACCGGGGTCTCTGAGCTCTTACGGAGGCTGGTCCTTGGATCTGCAGATGCAGGAGGCAGATATCAGTGGCTATATCCCCAATGGAGAATGGGACCTAGTGGGAATCCCCGGCAAGAGGAGTGAAAGGTTCTATGAGTGCTGCAAAGAGCCCTACCCCGATGTCACCTTCACAGTGACCAGGAGACGGGGAGGGAAC
nachra7_Frag507-672_insertion516	CATGTTTAGCGTCTCCTGGGTCCTGGTCTAGCGGGAGACCGGGGTCTCTGAGCTACGGAGGCTGGTCCTTGGATCTGCAGATGCAGGAGGCAGATATCAGTGGCTATATCCCCAATGGAGAATGGGACCTAGTGGGAATCCCCGGCAAGAGGAGTGAAAGGTTCTATGAGTGCTGCAAAGAGCCCTACCCCGATGTCACCTTCACAGTGACCAGGAGACGGGGAGGGAAC
nachra7_Frag507-672_insertion519	CATGTTTAGCGTCTCCTGGGTCCTGGTCTTACAGCGGGAGACCGGGGTCTCTGAGCGGAGGCTGGTCCTTGGATCTGCAGATGCAGGAGGCAGATATCAGTGGCTATATCCCCAATGGAGAATGGGACCTAGTGGGAATCCCCGGCAAGAGGAGTGAAAGGTTCTATGAGTGCTGCAAAGAGCCCTACCCCGATGTCACCTTCACAGTGACCAGGAGACGGGGAGGGAAC
nachra7_Frag507-672_insertion522	CATGTTTAGCGTCTCCTGGGTCCTGGTCTTACGGAAGCGGGAGACCGGGGTCTCTGAGCGGCTGGTCCTTGGATCTGCAGATGCAGGAGGCAGATATCAGTGGCTATATCCCCAATGGAGAATGGGACCTAGTGGGAATCCCCGGCAAGAGGAGTGAAAGGTTCTATGAGTGCTGCAAAGAGCCCTACCCCGATGTCACCTTCACAGTGACCAGGAGACGGGGAGGGAAC
nachra7_Frag507-672_insertion525	CATGTTTAGCGTCTCCTGGGTCCTGGTCTTACGGAGGCAGCGGGAGACCGGGGTCTCTGAGCTGGTCCTTGGATCTGCAGATGCAGGAGGCAGATATCAGTGGCTATATCCCCAATGGAGAATGGGACCTAGTGGGAATCCCCGGCAAGAGGAGTGAAAGGTTCTATGAGTGCTGCAAAGAGCCCTACCCCGATGTCACCTTCACAGTGACCAGGAGACGGGGAGGGAAC
nachra7_Frag507-672_insertion528	CATGTTTAGCGTCTCCTGGGTCCTGGTCTTACGGAGGCTGGAGCGGGAGACCGGGGTCTCTGAGCTCCTTGGATCTGCAGATGCAGGAGGCAGATATCAGTGGCTATATCCCCAATGGAGAATGGGACCTAGTGGGAATCCCCGGCAAGAGGAGTGAAAGGTTCTATGAGTGCTGCAAAGAGCCCTACCCCGATGTCACCTTCACAGTGACCAGGAGACGGGGAGGGAAC
nachra7_Frag507-672_insertion531	CATGTTTAGCGTCTCCTGGGTCCTGGTCTTACGGAGGCTGGTCCAGCGGGAGACCGGGGTCTCTGAGCTTGGATCTGCAGATGCAGGAGGCAGATATCAGTGGCTATATCCCCAATGGAGAATGGGACCTAGTGGGAATCCCCGGCAAGAGGAGTGAAAGGTTCTATGAGTGCTGCAAAGAGCCCTACCCCGATGTCACCTTCACAGTGACCAGGAGACGGGGAGGGAAC
nachra7_Frag507-672_insertion534	CATGTTTAGCGTCTCCTGGGTCCTGGTCTTACGGAGGCTGGTCCTTGAGCGGGAGACCGGGGTCTCTGAGCGATCTGCAGATGCAGGAGGCAGATATCAGTGGCTATATCCCCAATGGAGAATGGGACCTAGTGGGAATCCCCGGCAAGAGGAGTGAAAGGTTCTATGAGTGCTGCAAAGAGCCCTACCCCGATGTCACCTTCACAGTGACCAGGAGACGGGGAGGGAAC
nachra7_Frag507-672_insertion537	CATGTTTAGCGTCTCCTGGGTCCTGGTCTTACGGAGGCTGGTCCTTGGATAGCGGGAGACCGGGGTCTCTGAGCCTGCAGATGCAGGAGGCAGATATCAGTGGCTATATCCCCAATGGAGAATGGGACCTAGTGGGAATCCCCGGCAAGAGGAGTGAAAGGTTCTATGAGTGCTGCAAAGAGCCCTACCCCGATGTCACCTTCACAGTGACCAGGAGACGGGGAGGGAAC
nachra7_Frag507-672_insertion540	CATGTTTAGCGTCTCCTGGGTCCTGGTCTTACGGAGGCTGGTCCTTGGATCTGAGCGGGAGACCGGGGTCTCTGAGCCAGATGCAGGAGGCAGATATCAGTGGCTATATCCCCAATGGAGAATGGGACCTAGTGGGAATCCCCGGCAAGAGGAGTGAAAGGTTCTATGAGTGCTGCAAAGAGCCCTACCCCGATGTCACCTTCACAGTGACCAGGAGACGGGGAGGGAAC
nachra7_Frag507-672_insertion543	CATGTTTAGCGTCTCCTGGGTCCTGGTCTTACGGAGGCTGGTCCTTGGATCTGCAGAGCGGGAGACCGGGGTCTCTGAGCATGCAGGAGGCAGATATCAGTGGCTATATCCCCAATGGAGAATGGGACCTAGTGGGAATCCCCGGCAAGAGGAGTGAAAGGTTCTATGAGTGCTGCAAAGAGCCCTACCCCGATGTCACCTTCACAGTGACCAGGAGACGGGGAGGGAAC
nachra7_Frag507-672_insertion546	CATGTTTAGCGTCTCCTGGGTCCTGGTCTTACGGAGGCTGGTCCTTGGATCTGCAGATGAGCGGGAGACCGGGGTCTCTGAGCCAGGAGGCAGATATCAGTGGCTATATCCCCAATGGAGAATGGGACCTAGTGGGAATCCCCGGCAAGAGGAGTGAAAGGTTCTATGAGTGCTGCAAAGAGCCCTACCCCGATGTCACCTTCACAGTGACCAGGAGACGGGGAGGGAAC
nachra7_Frag507-672_insertion549	CATGTTTAGCGTCTCCTGGGTCCTGGTCTTACGGAGGCTGGTCCTTGGATCTGCAGATGCAGAGCGGGAGACCGGGGTCTCTGAGCGAGGCAGATATCAGTGGCTATATCCCCAATGGAGAATGGGACCTAGTGGGAATCCCCGGCAAGAGGAGTGAAAGGTTCTATGAGTGCTGCAAAGAGCCCTACCCCGATGTCACCTTCACAGTGACCAGGAGACGGGGAGGGAAC
nachra7_Frag507-672_insertion552	CATGTTTAGCGTCTCCTGGGTCCTGGTCTTACGGAGGCTGGTCCTTGGATCTGCAGATGCAGGAGAGCGGGAGACCGGGGTCTCTGAGCGCAGATATCAGTGGCTATATCCCCAATGGAGAATGGGACCTAGTGGGAATCCCCGGCAAGAGGAGTGAAAGGTTCTATGAGTGCTGCAAAGAGCCCTACCCCGATGTCACCTTCACAGTGACCAGGAGACGGGGAGGGAAC
nachra7_Frag507-672_insertion555	CATGTTTAGCGTCTCCTGGGTCCTGGTCTTACGGAGGCTGGTCCTTGGATCTGCAGATGCAGGAGGCAAGCGGGAGACCGGGGTCTCTGAGCGATATCAGTGGCTATATCCCCAATGGAGAATGGGACCTAGTGGGAATCCCCGGCAAGAGGAGTGAAAGGTTCTATGAGTGCTGCAAAGAGCCCTACCCCGATGTCACCTTCACAGTGACCAGGAGACGGGGAGGGAAC
nachra7_Frag507-672_insertion558	CATGTTTAGCGTCTCCTGGGTCCTGGTCTTACGGAGGCTGGTCCTTGGATCTGCAGATGCAGGAGGCAGATAGCGGGAGACCGGGGTCTCTGAGCATCAGTGGCTATATCCCCAATGGAGAATGGGACCTAGTGGGAATCCCCGGCAAGAGGAGTGAAAGGTTCTATGAGTGCTGCAAAGAGCCCTACCCCGATGTCACCTTCACAGTGACCAGGAGACGGGGAGGGAAC
nachra7_Frag507-672_insertion561	CATGTTTAGCGTCTCCTGGGTCCTGGTCTTACGGAGGCTGGTCCTTGGATCTGCAGATGCAGGAGGCAGATATCAGCGGGAGACCGGGGTCTCTGAGCAGTGGCTATATCCCCAATGGAGAATGGGACCTAGTGGGAATCCCCGGCAAGAGGAGTGAAAGGTTCTATGAGTGCTGCAAAGAGCCCTACCCCGATGTCACCTTCACAGTGACCAGGAGACGGGGAGGGAAC
nachra7_Frag507-672_insertion564	CATGTTTAGCGTCTCCTGGGTCCTGGTCTTACGGAGGCTGGTCCTTGGATCTGCAGATGCAGGAGGCAGATATCAGTAGCGGGAGACCGGGGTCTCTGAGCGGCTATATCCCCAATGGAGAATGGGACCTAGTGGGAATCCCCGGCAAGAGGAGTGAAAGGTTCTATGAGTGCTGCAAAGAGCCCTACCCCGATGTCACCTTCACAGTGACCAGGAGACGGGGAGGGAAC
nachra7_Frag507-672_insertion567	CATGTTTAGCGTCTCCTGGGTCCTGGTCTTACGGAGGCTGGTCCTTGGATCTGCAGATGCAGGAGGCAGATATCAGTGGCAGCGGGAGACCGGGGTCTCTGAGCTATATCCCCAATGGAGAATGGGACCTAGTGGGAATCCCCGGCAAGAGGAGTGAAAGGTTCTATGAGTGCTGCAAAGAGCCCTACCCCGATGTCACCTTCACAGTGACCAGGAGACGGGGAGGGAAC
nachra7_Frag507-672_insertion570	CATGTTTAGCGTCTCCTGGGTCCTGGTCTTACGGAGGCTGGTCCTTGGATCTGCAGATGCAGGAGGCAGATATCAGTGGCTATAGCGGGAGACCGGGGTCTCTGAGCATCCCCAATGGAGAATGGGACCTAGTGGGAATCCCCGGCAAGAGGAGTGAAAGGTTCTATGAGTGCTGCAAAGAGCCCTACCCCGATGTCACCTTCACAGTGACCAGGAGACGGGGAGGGAAC
nachra7_Frag507-672_insertion573	CATGTTTAGCGTCTCCTGGGTCCTGGTCTTACGGAGGCTGGTCCTTGGATCTGCAGATGCAGGAGGCAGATATCAGTGGCTATATCAGCGGGAGACCGGGGTCTCTGAGCCCCAATGGAGAATGGGACCTAGTGGGAATCCCCGGCAAGAGGAGTGAAAGGTTCTATGAGTGCTGCAAAGAGCCCTACCCCGATGTCACCTTCACAGTGACCAGGAGACGGGGAGGGAAC
nachra7_Frag507-672_insertion576	CATGTTTAGCGTCTCCTGGGTCCTGGTCTTACGGAGGCTGGTCCTTGGATCTGCAGATGCAGGAGGCAGATATCAGTGGCTATATCCCCAGCGGGAGACCGGGGTCTCTGAGCAATGGAGAATGGGACCTAGTGGGAATCCCCGGCAAGAGGAGTGAAAGGTTCTATGAGTGCTGCAAAGAGCCCTACCCCGATGTCACCTTCACAGTGACCAGGAGACGGGGAGGGAAC
nachra7_Frag507-672_insertion579	CATGTTTAGCGTCTCCTGGGTCCTGGTCTTACGGAGGCTGGTCCTTGGATCTGCAGATGCAGGAGGCAGATATCAGTGGCTATATCCCCAATAGCGGGAGACCGGGGTCTCTGAGCGGAGAATGGGACCTAGTGGGAATCCCCGGCAAGAGGAGTGAAAGGTTCTATGAGTGCTGCAAAGAGCCCTACCCCGATGTCACCTTCACAGTGACCAGGAGACGGGGAGGGAAC
nachra7_Frag507-672_insertion582	CATGTTTAGCGTCTCCTGGGTCCTGGTCTTACGGAGGCTGGTCCTTGGATCTGCAGATGCAGGAGGCAGATATCAGTGGCTATATCCCCAATGGAAGCGGGAGACCGGGGTCTCTGAGCGAATGGGACCTAGTGGGAATCCCCGGCAAGAGGAGTGAAAGGTTCTATGAGTGCTGCAAAGAGCCCTACCCCGATGTCACCTTCACAGTGACCAGGAGACGGGGAGGGAAC
nachra7_Frag507-672_insertion585	CATGTTTAGCGTCTCCTGGGTCCTGGTCTTACGGAGGCTGGTCCTTGGATCTGCAGATGCAGGAGGCAGATATCAGTGGCTATATCCCCAATGGAGAAAGCGGGAGACCGGGGTCTCTGAGCTGGGACCTAGTGGGAATCCCCGGCAAGAGGAGTGAAAGGTTCTATGAGTGCTGCAAAGAGCCCTACCCCGATGTCACCTTCACAGTGACCAGGAGACGGGGAGGGAAC
nachra7_Frag507-672_insertion588	CATGTTTAGCGTCTCCTGGGTCCTGGTCTTACGGAGGCTGGTCCTTGGATCTGCAGATGCAGGAGGCAGATATCAGTGGCTATATCCCCAATGGAGAATGGAGCGGGAGACCGGGGTCTCTGAGCGACCTAGTGGGAATCCCCGGCAAGAGGAGTGAAAGGTTCTATGAGTGCTGCAAAGAGCCCTACCCCGATGTCACCTTCACAGTGACCAGGAGACGGGGAGGGAAC
nachra7_Frag507-672_insertion591	CATGTTTAGCGTCTCCTGGGTCCTGGTCTTACGGAGGCTGGTCCTTGGATCTGCAGATGCAGGAGGCAGATATCAGTGGCTATATCCCCAATGGAGAATGGGACAGCGGGAGACCGGGGTCTCTGAGCCTAGTGGGAATCCCCGGCAAGAGGAGTGAAAGGTTCTATGAGTGCTGCAAAGAGCCCTACCCCGATGTCACCTTCACAGTGACCAGGAGACGGGGAGGGAAC
nachra7_Frag507-672_insertion594	CATGTTTAGCGTCTCCTGGGTCCTGGTCTTACGGAGGCTGGTCCTTGGATCTGCAGATGCAGGAGGCAGATATCAGTGGCTATATCCCCAATGGAGAATGGGACCTAAGCGGGAGACCGGGGTCTCTGAGCGTGGGAATCCCCGGCAAGAGGAGTGAAAGGTTCTATGAGTGCTGCAAAGAGCCCTACCCCGATGTCACCTTCACAGTGACCAGGAGACGGGGAGGGAAC
nachra7_Frag507-672_insertion597	CATGTTTAGCGTCTCCTGGGTCCTGGTCTTACGGAGGCTGGTCCTTGGATCTGCAGATGCAGGAGGCAGATATCAGTGGCTATATCCCCAATGGAGAATGGGACCTAGTGAGCGGGAGACCGGGGTCTCTGAGCGGAATCCCCGGCAAGAGGAGTGAAAGGTTCTATGAGTGCTGCAAAGAGCCCTACCCCGATGTCACCTTCACAGTGACCAGGAGACGGGGAGGGAAC
nachra7_Frag507-672_insertion600	CATGTTTAGCGTCTCCTGGGTCCTGGTCTTACGGAGGCTGGTCCTTGGATCTGCAGATGCAGGAGGCAGATATCAGTGGCTATATCCCCAATGGAGAATGGGACCTAGTGGGAAGCGGGAGACCGGGGTCTCTGAGCATCCCCGGCAAGAGGAGTGAAAGGTTCTATGAGTGCTGCAAAGAGCCCTACCCCGATGTCACCTTCACAGTGACCAGGAGACGGGGAGGGAAC
nachra7_Frag507-672_insertion603	CATGTTTAGCGTCTCCTGGGTCCTGGTCTTACGGAGGCTGGTCCTTGGATCTGCAGATGCAGGAGGCAGATATCAGTGGCTATATCCCCAATGGAGAATGGGACCTAGTGGGAATCAGCGGGAGACCGGGGTCTCTGAGCCCCGGCAAGAGGAGTGAAAGGTTCTATGAGTGCTGCAAAGAGCCCTACCCCGATGTCACCTTCACAGTGACCAGGAGACGGGGAGGGAAC
nachra7_Frag507-672_insertion606	CATGTTTAGCGTCTCCTGGGTCCTGGTCTTACGGAGGCTGGTCCTTGGATCTGCAGATGCAGGAGGCAGATATCAGTGGCTATATCCCCAATGGAGAATGGGACCTAGTGGGAATCCCCAGCGGGAGACCGGGGTCTCTGAGCGGCAAGAGGAGTGAAAGGTTCTATGAGTGCTGCAAAGAGCCCTACCCCGATGTCACCTTCACAGTGACCAGGAGACGGGGAGGGAAC
nachra7_Frag507-672_insertion609	CATGTTTAGCGTCTCCTGGGTCCTGGTCTTACGGAGGCTGGTCCTTGGATCTGCAGATGCAGGAGGCAGATATCAGTGGCTATATCCCCAATGGAGAATGGGACCTAGTGGGAATCCCCGGCAGCGGGAGACCGGGGTCTCTGAGCAAGAGGAGTGAAAGGTTCTATGAGTGCTGCAAAGAGCCCTACCCCGATGTCACCTTCACAGTGACCAGGAGACGGGGAGGGAAC
nachra7_Frag507-672_insertion612	CATGTTTAGCGTCTCCTGGGTCCTGGTCTTACGGAGGCTGGTCCTTGGATCTGCAGATGCAGGAGGCAGATATCAGTGGCTATATCCCCAATGGAGAATGGGACCTAGTGGGAATCCCCGGCAAGAGCGGGAGACCGGGGTCTCTGAGCAGGAGTGAAAGGTTCTATGAGTGCTGCAAAGAGCCCTACCCCGATGTCACCTTCACAGTGACCAGGAGACGGGGAGGGAAC
nachra7_Frag507-672_insertion615	CATGTTTAGCGTCTCCTGGGTCCTGGTCTTACGGAGGCTGGTCCTTGGATCTGCAGATGCAGGAGGCAGATATCAGTGGCTATATCCCCAATGGAGAATGGGACCTAGTGGGAATCCCCGGCAAGAGGAGCGGGAGACCGGGGTCTCTGAGCAGTGAAAGGTTCTATGAGTGCTGCAAAGAGCCCTACCCCGATGTCACCTTCACAGTGACCAGGAGACGGGGAGGGAAC
nachra7_Frag507-672_insertion618	CATGTTTAGCGTCTCCTGGGTCCTGGTCTTACGGAGGCTGGTCCTTGGATCTGCAGATGCAGGAGGCAGATATCAGTGGCTATATCCCCAATGGAGAATGGGACCTAGTGGGAATCCCCGGCAAGAGGAGTAGCGGGAGACCGGGGTCTCTGAGCGAAAGGTTCTATGAGTGCTGCAAAGAGCCCTACCCCGATGTCACCTTCACAGTGACCAGGAGACGGGGAGGGAAC
nachra7_Frag507-672_insertion621	CATGTTTAGCGTCTCCTGGGTCCTGGTCTTACGGAGGCTGGTCCTTGGATCTGCAGATGCAGGAGGCAGATATCAGTGGCTATATCCCCAATGGAGAATGGGACCTAGTGGGAATCCCCGGCAAGAGGAGTGAAAGCGGGAGACCGGGGTCTCTGAGCAGGTTCTATGAGTGCTGCAAAGAGCCCTACCCCGATGTCACCTTCACAGTGACCAGGAGACGGGGAGGGAAC
nachra7_Frag507-672_insertion624	CATGTTTAGCGTCTCCTGGGTCCTGGTCTTACGGAGGCTGGTCCTTGGATCTGCAGATGCAGGAGGCAGATATCAGTGGCTATATCCCCAATGGAGAATGGGACCTAGTGGGAATCCCCGGCAAGAGGAGTGAAAGGAGCGGGAGACCGGGGTCTCTGAGCTTCTATGAGTGCTGCAAAGAGCCCTACCCCGATGTCACCTTCACAGTGACCAGGAGACGGGGAGGGAAC
nachra7_Frag507-672_insertion627	CATGTTTAGCGTCTCCTGGGTCCTGGTCTTACGGAGGCTGGTCCTTGGATCTGCAGATGCAGGAGGCAGATATCAGTGGCTATATCCCCAATGGAGAATGGGACCTAGTGGGAATCCCCGGCAAGAGGAGTGAAAGGTTCAGCGGGAGACCGGGGTCTCTGAGCTATGAGTGCTGCAAAGAGCCCTACCCCGATGTCACCTTCACAGTGACCAGGAGACGGGGAGGGAAC
nachra7_Frag507-672_insertion630	CATGTTTAGCGTCTCCTGGGTCCTGGTCTTACGGAGGCTGGTCCTTGGATCTGCAGATGCAGGAGGCAGATATCAGTGGCTATATCCCCAATGGAGAATGGGACCTAGTGGGAATCCCCGGCAAGAGGAGTGAAAGGTTCTATAGCGGGAGACCGGGGTCTCTGAGCGAGTGCTGCAAAGAGCCCTACCCCGATGTCACCTTCACAGTGACCAGGAGACGGGGAGGGAAC
nachra7_Frag507-672_insertion633	CATGTTTAGCGTCTCCTGGGTCCTGGTCTTACGGAGGCTGGTCCTTGGATCTGCAGATGCAGGAGGCAGATATCAGTGGCTATATCCCCAATGGAGAATGGGACCTAGTGGGAATCCCCGGCAAGAGGAGTGAAAGGTTCTATGAGAGCGGGAGACCGGGGTCTCTGAGCTGCTGCAAAGAGCCCTACCCCGATGTCACCTTCACAGTGACCAGGAGACGGGGAGGGAAC
nachra7_Frag507-672_insertion636	CATGTTTAGCGTCTCCTGGGTCCTGGTCTTACGGAGGCTGGTCCTTGGATCTGCAGATGCAGGAGGCAGATATCAGTGGCTATATCCCCAATGGAGAATGGGACCTAGTGGGAATCCCCGGCAAGAGGAGTGAAAGGTTCTATGAGTGCAGCGGGAGACCGGGGTCTCTGAGCTGCAAAGAGCCCTACCCCGATGTCACCTTCACAGTGACCAGGAGACGGGGAGGGAAC
nachra7_Frag507-672_insertion639	CATGTTTAGCGTCTCCTGGGTCCTGGTCTTACGGAGGCTGGTCCTTGGATCTGCAGATGCAGGAGGCAGATATCAGTGGCTATATCCCCAATGGAGAATGGGACCTAGTGGGAATCCCCGGCAAGAGGAGTGAAAGGTTCTATGAGTGCTGCAGCGGGAGACCGGGGTCTCTGAGCAAAGAGCCCTACCCCGATGTCACCTTCACAGTGACCAGGAGACGGGGAGGGAAC
nachra7_Frag507-672_insertion642	CATGTTTAGCGTCTCCTGGGTCCTGGTCTTACGGAGGCTGGTCCTTGGATCTGCAGATGCAGGAGGCAGATATCAGTGGCTATATCCCCAATGGAGAATGGGACCTAGTGGGAATCCCCGGCAAGAGGAGTGAAAGGTTCTATGAGTGCTGCAAAAGCGGGAGACCGGGGTCTCTGAGCGAGCCCTACCCCGATGTCACCTTCACAGTGACCAGGAGACGGGGAGGGAAC
nachra7_Frag507-672_insertion645	CATGTTTAGCGTCTCCTGGGTCCTGGTCTTACGGAGGCTGGTCCTTGGATCTGCAGATGCAGGAGGCAGATATCAGTGGCTATATCCCCAATGGAGAATGGGACCTAGTGGGAATCCCCGGCAAGAGGAGTGAAAGGTTCTATGAGTGCTGCAAAGAGAGCGGGAGACCGGGGTCTCTGAGCCCCTACCCCGATGTCACCTTCACAGTGACCAGGAGACGGGGAGGGAAC
nachra7_Frag507-672_insertion648	CATGTTTAGCGTCTCCTGGGTCCTGGTCTTACGGAGGCTGGTCCTTGGATCTGCAGATGCAGGAGGCAGATATCAGTGGCTATATCCCCAATGGAGAATGGGACCTAGTGGGAATCCCCGGCAAGAGGAGTGAAAGGTTCTATGAGTGCTGCAAAGAGCCCAGCGGGAGACCGGGGTCTCTGAGCTACCCCGATGTCACCTTCACAGTGACCAGGAGACGGGGAGGGAAC
nachra7_Frag507-672_insertion651	CATGTTTAGCGTCTCCTGGGTCCTGGTCTTACGGAGGCTGGTCCTTGGATCTGCAGATGCAGGAGGCAGATATCAGTGGCTATATCCCCAATGGAGAATGGGACCTAGTGGGAATCCCCGGCAAGAGGAGTGAAAGGTTCTATGAGTGCTGCAAAGAGCCCTACAGCGGGAGACCGGGGTCTCTGAGCCCCGATGTCACCTTCACAGTGACCAGGAGACGGGGAGGGAAC
nachra7_Frag507-672_insertion654	CATGTTTAGCGTCTCCTGGGTCCTGGTCTTACGGAGGCTGGTCCTTGGATCTGCAGATGCAGGAGGCAGATATCAGTGGCTATATCCCCAATGGAGAATGGGACCTAGTGGGAATCCCCGGCAAGAGGAGTGAAAGGTTCTATGAGTGCTGCAAAGAGCCCTACCCCAGCGGGAGACCGGGGTCTCTGAGCGATGTCACCTTCACAGTGACCAGGAGACGGGGAGGGAAC
nachra7_Frag507-672_insertion657	CATGTTTAGCGTCTCCTGGGTCCTGGTCTTACGGAGGCTGGTCCTTGGATCTGCAGATGCAGGAGGCAGATATCAGTGGCTATATCCCCAATGGAGAATGGGACCTAGTGGGAATCCCCGGCAAGAGGAGTGAAAGGTTCTATGAGTGCTGCAAAGAGCCCTACCCCGATAGCGGGAGACCGGGGTCTCTGAGCGTCACCTTCACAGTGACCAGGAGACGGGGAGGGAAC
nachra7_Frag507-672_insertion660	CATGTTTAGCGTCTCCTGGGTCCTGGTCTTACGGAGGCTGGTCCTTGGATCTGCAGATGCAGGAGGCAGATATCAGTGGCTATATCCCCAATGGAGAATGGGACCTAGTGGGAATCCCCGGCAAGAGGAGTGAAAGGTTCTATGAGTGCTGCAAAGAGCCCTACCCCGATGTCAGCGGGAGACCGGGGTCTCTGAGCACCTTCACAGTGACCAGGAGACGGGGAGGGAAC
nachra7_Frag507-672_insertion663	CATGTTTAGCGTCTCCTGGGTCCTGGTCTTACGGAGGCTGGTCCTTGGATCTGCAGATGCAGGAGGCAGATATCAGTGGCTATATCCCCAATGGAGAATGGGACCTAGTGGGAATCCCCGGCAAGAGGAGTGAAAGGTTCTATGAGTGCTGCAAAGAGCCCTACCCCGATGTCACCAGCGGGAGACCGGGGTCTCTGAGCTTCACAGTGACCAGGAGACGGGGAGGGAAC
nachra7_Frag507-672_insertion666	CATGTTTAGCGTCTCCTGGGTCCTGGTCTTACGGAGGCTGGTCCTTGGATCTGCAGATGCAGGAGGCAGATATCAGTGGCTATATCCCCAATGGAGAATGGGACCTAGTGGGAATCCCCGGCAAGAGGAGTGAAAGGTTCTATGAGTGCTGCAAAGAGCCCTACCCCGATGTCACCTTCAGCGGGAGACCGGGGTCTCTGAGCACAGTGACCAGGAGACGGGGAGGGAAC
nachra7_Frag507-672_insertion669	CATGTTTAGCGTCTCCTGGGTCCTGGTCTTACGGAGGCTGGTCCTTGGATCTGCAGATGCAGGAGGCAGATATCAGTGGCTATATCCCCAATGGAGAATGGGACCTAGTGGGAATCCCCGGCAAGAGGAGTGAAAGGTTCTATGAGTGCTGCAAAGAGCCCTACCCCGATGTCACCTTCACAAGCGGGAGACCGGGGTCTCTGAGCGTGACCAGGAGACGGGGAGGGAAC
nachra7_Frag507-672_insertion672	CATGTTTAGCGTCTCCTGGGTCCTGGTCTTACGGAGGCTGGTCCTTGGATCTGCAGATGCAGGAGGCAGATATCAGTGGCTATATCCCCAATGGAGAATGGGACCTAGTGGGAATCCCCGGCAAGAGGAGTGAAAGGTTCTATGAGTGCTGCAAAGAGCCCTACCCCGATGTCACCTTCACAGTGAGCGGGAGACCGGGGTCTCTGAGCACCAGGAGACGGGGAGGGAAC
nachra7_Frag675-840_insertion675	GTACATGAACGTCTCCGACCAGCGGGAGACCGGGGTCTCTGAGCATGCGCCGCAGGACGCTCTACTATGGCCTCAACCTGCTGATCCCCTGTGTGCTCATCTCCGCCCTCGCCCTGCTGGTGTTCCTGCTTCCTGCAGATTCCGGGGAGAAGATTTCCCTGGGGATAACAGTCTTACTCTCTCTTACCGTCTTCATGCTGCTCGTGGCTGAGAGGAGACGCGCTGAGGAG
nachra7_Frag675-840_insertion678	GTACATGAACGTCTCCGACCATGAGCGGGAGACCGGGGTCTCTGAGCCGCCGCAGGACGCTCTACTATGGCCTCAACCTGCTGATCCCCTGTGTGCTCATCTCCGCCCTCGCCCTGCTGGTGTTCCTGCTTCCTGCAGATTCCGGGGAGAAGATTTCCCTGGGGATAACAGTCTTACTCTCTCTTACCGTCTTCATGCTGCTCGTGGCTGAGAGGAGACGCGCTGAGGAG
nachra7_Frag675-840_insertion681	GTACATGAACGTCTCCGACCATGCGCAGCGGGAGACCGGGGTCTCTGAGCCGCAGGACGCTCTACTATGGCCTCAACCTGCTGATCCCCTGTGTGCTCATCTCCGCCCTCGCCCTGCTGGTGTTCCTGCTTCCTGCAGATTCCGGGGAGAAGATTTCCCTGGGGATAACAGTCTTACTCTCTCTTACCGTCTTCATGCTGCTCGTGGCTGAGAGGAGACGCGCTGAGGAG
nachra7_Frag675-840_insertion684	GTACATGAACGTCTCCGACCATGCGCCGCAGCGGGAGACCGGGGTCTCTGAGCAGGACGCTCTACTATGGCCTCAACCTGCTGATCCCCTGTGTGCTCATCTCCGCCCTCGCCCTGCTGGTGTTCCTGCTTCCTGCAGATTCCGGGGAGAAGATTTCCCTGGGGATAACAGTCTTACTCTCTCTTACCGTCTTCATGCTGCTCGTGGCTGAGAGGAGACGCGCTGAGGAG
nachra7_Frag675-840_insertion687	GTACATGAACGTCTCCGACCATGCGCCGCAGGAGCGGGAGACCGGGGTCTCTGAGCACGCTCTACTATGGCCTCAACCTGCTGATCCCCTGTGTGCTCATCTCCGCCCTCGCCCTGCTGGTGTTCCTGCTTCCTGCAGATTCCGGGGAGAAGATTTCCCTGGGGATAACAGTCTTACTCTCTCTTACCGTCTTCATGCTGCTCGTGGCTGAGAGGAGACGCGCTGAGGAG
nachra7_Frag675-840_insertion690	GTACATGAACGTCTCCGACCATGCGCCGCAGGACGAGCGGGAGACCGGGGTCTCTGAGCCTCTACTATGGCCTCAACCTGCTGATCCCCTGTGTGCTCATCTCCGCCCTCGCCCTGCTGGTGTTCCTGCTTCCTGCAGATTCCGGGGAGAAGATTTCCCTGGGGATAACAGTCTTACTCTCTCTTACCGTCTTCATGCTGCTCGTGGCTGAGAGGAGACGCGCTGAGGAG
nachra7_Frag675-840_insertion693	GTACATGAACGTCTCCGACCATGCGCCGCAGGACGCTCAGCGGGAGACCGGGGTCTCTGAGCTACTATGGCCTCAACCTGCTGATCCCCTGTGTGCTCATCTCCGCCCTCGCCCTGCTGGTGTTCCTGCTTCCTGCAGATTCCGGGGAGAAGATTTCCCTGGGGATAACAGTCTTACTCTCTCTTACCGTCTTCATGCTGCTCGTGGCTGAGAGGAGACGCGCTGAGGAG
nachra7_Frag675-840_insertion696	GTACATGAACGTCTCCGACCATGCGCCGCAGGACGCTCTACAGCGGGAGACCGGGGTCTCTGAGCTATGGCCTCAACCTGCTGATCCCCTGTGTGCTCATCTCCGCCCTCGCCCTGCTGGTGTTCCTGCTTCCTGCAGATTCCGGGGAGAAGATTTCCCTGGGGATAACAGTCTTACTCTCTCTTACCGTCTTCATGCTGCTCGTGGCTGAGAGGAGACGCGCTGAGGAG
nachra7_Frag675-840_insertion699	GTACATGAACGTCTCCGACCATGCGCCGCAGGACGCTCTACTATAGCGGGAGACCGGGGTCTCTGAGCGGCCTCAACCTGCTGATCCCCTGTGTGCTCATCTCCGCCCTCGCCCTGCTGGTGTTCCTGCTTCCTGCAGATTCCGGGGAGAAGATTTCCCTGGGGATAACAGTCTTACTCTCTCTTACCGTCTTCATGCTGCTCGTGGCTGAGAGGAGACGCGCTGAGGAG
nachra7_Frag675-840_insertion702	GTACATGAACGTCTCCGACCATGCGCCGCAGGACGCTCTACTATGGCAGCGGGAGACCGGGGTCTCTGAGCCTCAACCTGCTGATCCCCTGTGTGCTCATCTCCGCCCTCGCCCTGCTGGTGTTCCTGCTTCCTGCAGATTCCGGGGAGAAGATTTCCCTGGGGATAACAGTCTTACTCTCTCTTACCGTCTTCATGCTGCTCGTGGCTGAGAGGAGACGCGCTGAGGAG
nachra7_Frag675-840_insertion705	GTACATGAACGTCTCCGACCATGCGCCGCAGGACGCTCTACTATGGCCTCAGCGGGAGACCGGGGTCTCTGAGCAACCTGCTGATCCCCTGTGTGCTCATCTCCGCCCTCGCCCTGCTGGTGTTCCTGCTTCCTGCAGATTCCGGGGAGAAGATTTCCCTGGGGATAACAGTCTTACTCTCTCTTACCGTCTTCATGCTGCTCGTGGCTGAGAGGAGACGCGCTGAGGAG
nachra7_Frag675-840_insertion708	GTACATGAACGTCTCCGACCATGCGCCGCAGGACGCTCTACTATGGCCTCAACAGCGGGAGACCGGGGTCTCTGAGCCTGCTGATCCCCTGTGTGCTCATCTCCGCCCTCGCCCTGCTGGTGTTCCTGCTTCCTGCAGATTCCGGGGAGAAGATTTCCCTGGGGATAACAGTCTTACTCTCTCTTACCGTCTTCATGCTGCTCGTGGCTGAGAGGAGACGCGCTGAGGAG
nachra7_Frag675-840_insertion711	GTACATGAACGTCTCCGACCATGCGCCGCAGGACGCTCTACTATGGCCTCAACCTGAGCGGGAGACCGGGGTCTCTGAGCCTGATCCCCTGTGTGCTCATCTCCGCCCTCGCCCTGCTGGTGTTCCTGCTTCCTGCAGATTCCGGGGAGAAGATTTCCCTGGGGATAACAGTCTTACTCTCTCTTACCGTCTTCATGCTGCTCGTGGCTGAGAGGAGACGCGCTGAGGAG
nachra7_Frag675-840_insertion714	GTACATGAACGTCTCCGACCATGCGCCGCAGGACGCTCTACTATGGCCTCAACCTGCTGAGCGGGAGACCGGGGTCTCTGAGCATCCCCTGTGTGCTCATCTCCGCCCTCGCCCTGCTGGTGTTCCTGCTTCCTGCAGATTCCGGGGAGAAGATTTCCCTGGGGATAACAGTCTTACTCTCTCTTACCGTCTTCATGCTGCTCGTGGCTGAGAGGAGACGCGCTGAGGAG
nachra7_Frag675-840_insertion717	GTACATGAACGTCTCCGACCATGCGCCGCAGGACGCTCTACTATGGCCTCAACCTGCTGATCAGCGGGAGACCGGGGTCTCTGAGCCCCTGTGTGCTCATCTCCGCCCTCGCCCTGCTGGTGTTCCTGCTTCCTGCAGATTCCGGGGAGAAGATTTCCCTGGGGATAACAGTCTTACTCTCTCTTACCGTCTTCATGCTGCTCGTGGCTGAGAGGAGACGCGCTGAGGAG
nachra7_Frag675-840_insertion720	GTACATGAACGTCTCCGACCATGCGCCGCAGGACGCTCTACTATGGCCTCAACCTGCTGATCCCCAGCGGGAGACCGGGGTCTCTGAGCTGTGTGCTCATCTCCGCCCTCGCCCTGCTGGTGTTCCTGCTTCCTGCAGATTCCGGGGAGAAGATTTCCCTGGGGATAACAGTCTTACTCTCTCTTACCGTCTTCATGCTGCTCGTGGCTGAGAGGAGACGCGCTGAGGAG
nachra7_Frag675-840_insertion723	GTACATGAACGTCTCCGACCATGCGCCGCAGGACGCTCTACTATGGCCTCAACCTGCTGATCCCCTGTAGCGGGAGACCGGGGTCTCTGAGCGTGCTCATCTCCGCCCTCGCCCTGCTGGTGTTCCTGCTTCCTGCAGATTCCGGGGAGAAGATTTCCCTGGGGATAACAGTCTTACTCTCTCTTACCGTCTTCATGCTGCTCGTGGCTGAGAGGAGACGCGCTGAGGAG
nachra7_Frag675-840_insertion726	GTACATGAACGTCTCCGACCATGCGCCGCAGGACGCTCTACTATGGCCTCAACCTGCTGATCCCCTGTGTGAGCGGGAGACCGGGGTCTCTGAGCCTCATCTCCGCCCTCGCCCTGCTGGTGTTCCTGCTTCCTGCAGATTCCGGGGAGAAGATTTCCCTGGGGATAACAGTCTTACTCTCTCTTACCGTCTTCATGCTGCTCGTGGCTGAGAGGAGACGCGCTGAGGAG
nachra7_Frag675-840_insertion729	GTACATGAACGTCTCCGACCATGCGCCGCAGGACGCTCTACTATGGCCTCAACCTGCTGATCCCCTGTGTGCTCAGCGGGAGACCGGGGTCTCTGAGCATCTCCGCCCTCGCCCTGCTGGTGTTCCTGCTTCCTGCAGATTCCGGGGAGAAGATTTCCCTGGGGATAACAGTCTTACTCTCTCTTACCGTCTTCATGCTGCTCGTGGCTGAGAGGAGACGCGCTGAGGAG
nachra7_Frag675-840_insertion732	GTACATGAACGTCTCCGACCATGCGCCGCAGGACGCTCTACTATGGCCTCAACCTGCTGATCCCCTGTGTGCTCATCAGCGGGAGACCGGGGTCTCTGAGCTCCGCCCTCGCCCTGCTGGTGTTCCTGCTTCCTGCAGATTCCGGGGAGAAGATTTCCCTGGGGATAACAGTCTTACTCTCTCTTACCGTCTTCATGCTGCTCGTGGCTGAGAGGAGACGCGCTGAGGAG
nachra7_Frag675-840_insertion735	GTACATGAACGTCTCCGACCATGCGCCGCAGGACGCTCTACTATGGCCTCAACCTGCTGATCCCCTGTGTGCTCATCTCCAGCGGGAGACCGGGGTCTCTGAGCGCCCTCGCCCTGCTGGTGTTCCTGCTTCCTGCAGATTCCGGGGAGAAGATTTCCCTGGGGATAACAGTCTTACTCTCTCTTACCGTCTTCATGCTGCTCGTGGCTGAGAGGAGACGCGCTGAGGAG
nachra7_Frag675-840_insertion738	GTACATGAACGTCTCCGACCATGCGCCGCAGGACGCTCTACTATGGCCTCAACCTGCTGATCCCCTGTGTGCTCATCTCCGCCAGCGGGAGACCGGGGTCTCTGAGCCTCGCCCTGCTGGTGTTCCTGCTTCCTGCAGATTCCGGGGAGAAGATTTCCCTGGGGATAACAGTCTTACTCTCTCTTACCGTCTTCATGCTGCTCGTGGCTGAGAGGAGACGCGCTGAGGAG
nachra7_Frag675-840_insertion741	GTACATGAACGTCTCCGACCATGCGCCGCAGGACGCTCTACTATGGCCTCAACCTGCTGATCCCCTGTGTGCTCATCTCCGCCCTCAGCGGGAGACCGGGGTCTCTGAGCGCCCTGCTGGTGTTCCTGCTTCCTGCAGATTCCGGGGAGAAGATTTCCCTGGGGATAACAGTCTTACTCTCTCTTACCGTCTTCATGCTGCTCGTGGCTGAGAGGAGACGCGCTGAGGAG
nachra7_Frag675-840_insertion744	GTACATGAACGTCTCCGACCATGCGCCGCAGGACGCTCTACTATGGCCTCAACCTGCTGATCCCCTGTGTGCTCATCTCCGCCCTCGCCAGCGGGAGACCGGGGTCTCTGAGCCTGCTGGTGTTCCTGCTTCCTGCAGATTCCGGGGAGAAGATTTCCCTGGGGATAACAGTCTTACTCTCTCTTACCGTCTTCATGCTGCTCGTGGCTGAGAGGAGACGCGCTGAGGAG
nachra7_Frag675-840_insertion747	GTACATGAACGTCTCCGACCATGCGCCGCAGGACGCTCTACTATGGCCTCAACCTGCTGATCCCCTGTGTGCTCATCTCCGCCCTCGCCCTGAGCGGGAGACCGGGGTCTCTGAGCCTGGTGTTCCTGCTTCCTGCAGATTCCGGGGAGAAGATTTCCCTGGGGATAACAGTCTTACTCTCTCTTACCGTCTTCATGCTGCTCGTGGCTGAGAGGAGACGCGCTGAGGAG
nachra7_Frag675-840_insertion750	GTACATGAACGTCTCCGACCATGCGCCGCAGGACGCTCTACTATGGCCTCAACCTGCTGATCCCCTGTGTGCTCATCTCCGCCCTCGCCCTGCTGAGCGGGAGACCGGGGTCTCTGAGCGTGTTCCTGCTTCCTGCAGATTCCGGGGAGAAGATTTCCCTGGGGATAACAGTCTTACTCTCTCTTACCGTCTTCATGCTGCTCGTGGCTGAGAGGAGACGCGCTGAGGAG
nachra7_Frag675-840_insertion753	GTACATGAACGTCTCCGACCATGCGCCGCAGGACGCTCTACTATGGCCTCAACCTGCTGATCCCCTGTGTGCTCATCTCCGCCCTCGCCCTGCTGGTGAGCGGGAGACCGGGGTCTCTGAGCTTCCTGCTTCCTGCAGATTCCGGGGAGAAGATTTCCCTGGGGATAACAGTCTTACTCTCTCTTACCGTCTTCATGCTGCTCGTGGCTGAGAGGAGACGCGCTGAGGAG
nachra7_Frag675-840_insertion756	GTACATGAACGTCTCCGACCATGCGCCGCAGGACGCTCTACTATGGCCTCAACCTGCTGATCCCCTGTGTGCTCATCTCCGCCCTCGCCCTGCTGGTGTTCAGCGGGAGACCGGGGTCTCTGAGCCTGCTTCCTGCAGATTCCGGGGAGAAGATTTCCCTGGGGATAACAGTCTTACTCTCTCTTACCGTCTTCATGCTGCTCGTGGCTGAGAGGAGACGCGCTGAGGAG
nachra7_Frag675-840_insertion759	GTACATGAACGTCTCCGACCATGCGCCGCAGGACGCTCTACTATGGCCTCAACCTGCTGATCCCCTGTGTGCTCATCTCCGCCCTCGCCCTGCTGGTGTTCCTGAGCGGGAGACCGGGGTCTCTGAGCCTTCCTGCAGATTCCGGGGAGAAGATTTCCCTGGGGATAACAGTCTTACTCTCTCTTACCGTCTTCATGCTGCTCGTGGCTGAGAGGAGACGCGCTGAGGAG
nachra7_Frag675-840_insertion762	GTACATGAACGTCTCCGACCATGCGCCGCAGGACGCTCTACTATGGCCTCAACCTGCTGATCCCCTGTGTGCTCATCTCCGCCCTCGCCCTGCTGGTGTTCCTGCTTAGCGGGAGACCGGGGTCTCTGAGCCCTGCAGATTCCGGGGAGAAGATTTCCCTGGGGATAACAGTCTTACTCTCTCTTACCGTCTTCATGCTGCTCGTGGCTGAGAGGAGACGCGCTGAGGAG
nachra7_Frag675-840_insertion765	GTACATGAACGTCTCCGACCATGCGCCGCAGGACGCTCTACTATGGCCTCAACCTGCTGATCCCCTGTGTGCTCATCTCCGCCCTCGCCCTGCTGGTGTTCCTGCTTCCTAGCGGGAGACCGGGGTCTCTGAGCGCAGATTCCGGGGAGAAGATTTCCCTGGGGATAACAGTCTTACTCTCTCTTACCGTCTTCATGCTGCTCGTGGCTGAGAGGAGACGCGCTGAGGAG
nachra7_Frag675-840_insertion768	GTACATGAACGTCTCCGACCATGCGCCGCAGGACGCTCTACTATGGCCTCAACCTGCTGATCCCCTGTGTGCTCATCTCCGCCCTCGCCCTGCTGGTGTTCCTGCTTCCTGCAAGCGGGAGACCGGGGTCTCTGAGCGATTCCGGGGAGAAGATTTCCCTGGGGATAACAGTCTTACTCTCTCTTACCGTCTTCATGCTGCTCGTGGCTGAGAGGAGACGCGCTGAGGAG
nachra7_Frag675-840_insertion771	GTACATGAACGTCTCCGACCATGCGCCGCAGGACGCTCTACTATGGCCTCAACCTGCTGATCCCCTGTGTGCTCATCTCCGCCCTCGCCCTGCTGGTGTTCCTGCTTCCTGCAGATAGCGGGAGACCGGGGTCTCTGAGCTCCGGGGAGAAGATTTCCCTGGGGATAACAGTCTTACTCTCTCTTACCGTCTTCATGCTGCTCGTGGCTGAGAGGAGACGCGCTGAGGAG
nachra7_Frag675-840_insertion774	GTACATGAACGTCTCCGACCATGCGCCGCAGGACGCTCTACTATGGCCTCAACCTGCTGATCCCCTGTGTGCTCATCTCCGCCCTCGCCCTGCTGGTGTTCCTGCTTCCTGCAGATTCCAGCGGGAGACCGGGGTCTCTGAGCGGGGAGAAGATTTCCCTGGGGATAACAGTCTTACTCTCTCTTACCGTCTTCATGCTGCTCGTGGCTGAGAGGAGACGCGCTGAGGAG
nachra7_Frag675-840_insertion777	GTACATGAACGTCTCCGACCATGCGCCGCAGGACGCTCTACTATGGCCTCAACCTGCTGATCCCCTGTGTGCTCATCTCCGCCCTCGCCCTGCTGGTGTTCCTGCTTCCTGCAGATTCCGGGAGCGGGAGACCGGGGTCTCTGAGCGAGAAGATTTCCCTGGGGATAACAGTCTTACTCTCTCTTACCGTCTTCATGCTGCTCGTGGCTGAGAGGAGACGCGCTGAGGAG
nachra7_Frag675-840_insertion780	GTACATGAACGTCTCCGACCATGCGCCGCAGGACGCTCTACTATGGCCTCAACCTGCTGATCCCCTGTGTGCTCATCTCCGCCCTCGCCCTGCTGGTGTTCCTGCTTCCTGCAGATTCCGGGGAGAGCGGGAGACCGGGGTCTCTGAGCAAGATTTCCCTGGGGATAACAGTCTTACTCTCTCTTACCGTCTTCATGCTGCTCGTGGCTGAGAGGAGACGCGCTGAGGAG
nachra7_Frag675-840_insertion783	GTACATGAACGTCTCCGACCATGCGCCGCAGGACGCTCTACTATGGCCTCAACCTGCTGATCCCCTGTGTGCTCATCTCCGCCCTCGCCCTGCTGGTGTTCCTGCTTCCTGCAGATTCCGGGGAGAAGAGCGGGAGACCGGGGTCTCTGAGCATTTCCCTGGGGATAACAGTCTTACTCTCTCTTACCGTCTTCATGCTGCTCGTGGCTGAGAGGAGACGCGCTGAGGAG
nachra7_Frag675-840_insertion786	GTACATGAACGTCTCCGACCATGCGCCGCAGGACGCTCTACTATGGCCTCAACCTGCTGATCCCCTGTGTGCTCATCTCCGCCCTCGCCCTGCTGGTGTTCCTGCTTCCTGCAGATTCCGGGGAGAAGATTAGCGGGAGACCGGGGTCTCTGAGCTCCCTGGGGATAACAGTCTTACTCTCTCTTACCGTCTTCATGCTGCTCGTGGCTGAGAGGAGACGCGCTGAGGAG
nachra7_Frag675-840_insertion789	GTACATGAACGTCTCCGACCATGCGCCGCAGGACGCTCTACTATGGCCTCAACCTGCTGATCCCCTGTGTGCTCATCTCCGCCCTCGCCCTGCTGGTGTTCCTGCTTCCTGCAGATTCCGGGGAGAAGATTTCCAGCGGGAGACCGGGGTCTCTGAGCCTGGGGATAACAGTCTTACTCTCTCTTACCGTCTTCATGCTGCTCGTGGCTGAGAGGAGACGCGCTGAGGAG
nachra7_Frag675-840_insertion792	GTACATGAACGTCTCCGACCATGCGCCGCAGGACGCTCTACTATGGCCTCAACCTGCTGATCCCCTGTGTGCTCATCTCCGCCCTCGCCCTGCTGGTGTTCCTGCTTCCTGCAGATTCCGGGGAGAAGATTTCCCTGAGCGGGAGACCGGGGTCTCTGAGCGGGATAACAGTCTTACTCTCTCTTACCGTCTTCATGCTGCTCGTGGCTGAGAGGAGACGCGCTGAGGAG
nachra7_Frag675-840_insertion795	GTACATGAACGTCTCCGACCATGCGCCGCAGGACGCTCTACTATGGCCTCAACCTGCTGATCCCCTGTGTGCTCATCTCCGCCCTCGCCCTGCTGGTGTTCCTGCTTCCTGCAGATTCCGGGGAGAAGATTTCCCTGGGGAGCGGGAGACCGGGGTCTCTGAGCATAACAGTCTTACTCTCTCTTACCGTCTTCATGCTGCTCGTGGCTGAGAGGAGACGCGCTGAGGAG
nachra7_Frag675-840_insertion798	GTACATGAACGTCTCCGACCATGCGCCGCAGGACGCTCTACTATGGCCTCAACCTGCTGATCCCCTGTGTGCTCATCTCCGCCCTCGCCCTGCTGGTGTTCCTGCTTCCTGCAGATTCCGGGGAGAAGATTTCCCTGGGGATAAGCGGGAGACCGGGGTCTCTGAGCACAGTCTTACTCTCTCTTACCGTCTTCATGCTGCTCGTGGCTGAGAGGAGACGCGCTGAGGAG
nachra7_Frag675-840_insertion801	GTACATGAACGTCTCCGACCATGCGCCGCAGGACGCTCTACTATGGCCTCAACCTGCTGATCCCCTGTGTGCTCATCTCCGCCCTCGCCCTGCTGGTGTTCCTGCTTCCTGCAGATTCCGGGGAGAAGATTTCCCTGGGGATAACAAGCGGGAGACCGGGGTCTCTGAGCGTCTTACTCTCTCTTACCGTCTTCATGCTGCTCGTGGCTGAGAGGAGACGCGCTGAGGAG
nachra7_Frag675-840_insertion804	GTACATGAACGTCTCCGACCATGCGCCGCAGGACGCTCTACTATGGCCTCAACCTGCTGATCCCCTGTGTGCTCATCTCCGCCCTCGCCCTGCTGGTGTTCCTGCTTCCTGCAGATTCCGGGGAGAAGATTTCCCTGGGGATAACAGTCAGCGGGAGACCGGGGTCTCTGAGCTTACTCTCTCTTACCGTCTTCATGCTGCTCGTGGCTGAGAGGAGACGCGCTGAGGAG

nachra7_Frag675-840_insertion807	GTACATGAACGTCTCCGACCATGCGCCGCAGGACGCTCTACTATGGCCTCAACCTGCTGATCCCCTGTGTGCTCATCTCCGCCCTCGCCCTGCTGGTGTTCCTGCTTCCTGCAGATTCCGGGGAGAAGATTTCCCTGGGGATAACAGTCTTAAGCGGGAGACCGGGGTCTCTGAGCCTCTCTCTTACCGTCTTCATGCTGCTCGTGGCTGAGAGGAGACGCGCTGAGGAG
nachra7_Frag675-840_insertion810	GTACATGAACGTCTCCGACCATGCGCCGCAGGACGCTCTACTATGGCCTCAACCTGCTGATCCCCTGTGTGCTCATCTCCGCCCTCGCCCTGCTGGTGTTCCTGCTTCCTGCAGATTCCGGGGAGAAGATTTCCCTGGGGATAACAGTCTTACTCAGCGGGAGACCGGGGTCTCTGAGCTCTCTTACCGTCTTCATGCTGCTCGTGGCTGAGAGGAGACGCGCTGAGGAG
nachra7_Frag675-840_insertion813	GTACATGAACGTCTCCGACCATGCGCCGCAGGACGCTCTACTATGGCCTCAACCTGCTGATCCCCTGTGTGCTCATCTCCGCCCTCGCCCTGCTGGTGTTCCTGCTTCCTGCAGATTCCGGGGAGAAGATTTCCCTGGGGATAACAGTCTTACTCTCTAGCGGGAGACCGGGGTCTCTGAGCCTTACCGTCTTCATGCTGCTCGTGGCTGAGAGGAGACGCGCTGAGGAG
nachra7_Frag675-840_insertion816	GTACATGAACGTCTCCGACCATGCGCCGCAGGACGCTCTACTATGGCCTCAACCTGCTGATCCCCTGTGTGCTCATCTCCGCCCTCGCCCTGCTGGTGTTCCTGCTTCCTGCAGATTCCGGGGAGAAGATTTCCCTGGGGATAACAGTCTTACTCTCTCTTAGCGGGAGACCGGGGTCTCTGAGCACCGTCTTCATGCTGCTCGTGGCTGAGAGGAGACGCGCTGAGGAG
nachra7_Frag675-840_insertion819	GTACATGAACGTCTCCGACCATGCGCCGCAGGACGCTCTACTATGGCCTCAACCTGCTGATCCCCTGTGTGCTCATCTCCGCCCTCGCCCTGCTGGTGTTCCTGCTTCCTGCAGATTCCGGGGAGAAGATTTCCCTGGGGATAACAGTCTTACTCTCTCTTACCAGCGGGAGACCGGGGTCTCTGAGCGTCTTCATGCTGCTCGTGGCTGAGAGGAGACGCGCTGAGGAG
nachra7_Frag675-840_insertion822	GTACATGAACGTCTCCGACCATGCGCCGCAGGACGCTCTACTATGGCCTCAACCTGCTGATCCCCTGTGTGCTCATCTCCGCCCTCGCCCTGCTGGTGTTCCTGCTTCCTGCAGATTCCGGGGAGAAGATTTCCCTGGGGATAACAGTCTTACTCTCTCTTACCGTCAGCGGGAGACCGGGGTCTCTGAGCTTCATGCTGCTCGTGGCTGAGAGGAGACGCGCTGAGGAG
nachra7_Frag675-840_insertion825	GTACATGAACGTCTCCGACCATGCGCCGCAGGACGCTCTACTATGGCCTCAACCTGCTGATCCCCTGTGTGCTCATCTCCGCCCTCGCCCTGCTGGTGTTCCTGCTTCCTGCAGATTCCGGGGAGAAGATTTCCCTGGGGATAACAGTCTTACTCTCTCTTACCGTCTTCAGCGGGAGACCGGGGTCTCTGAGCATGCTGCTCGTGGCTGAGAGGAGACGCGCTGAGGAG
nachra7_Frag675-840_insertion828	GTACATGAACGTCTCCGACCATGCGCCGCAGGACGCTCTACTATGGCCTCAACCTGCTGATCCCCTGTGTGCTCATCTCCGCCCTCGCCCTGCTGGTGTTCCTGCTTCCTGCAGATTCCGGGGAGAAGATTTCCCTGGGGATAACAGTCTTACTCTCTCTTACCGTCTTCATGAGCGGGAGACCGGGGTCTCTGAGCCTGCTCGTGGCTGAGAGGAGACGCGCTGAGGAG
nachra7_Frag675-840_insertion831	GTACATGAACGTCTCCGACCATGCGCCGCAGGACGCTCTACTATGGCCTCAACCTGCTGATCCCCTGTGTGCTCATCTCCGCCCTCGCCCTGCTGGTGTTCCTGCTTCCTGCAGATTCCGGGGAGAAGATTTCCCTGGGGATAACAGTCTTACTCTCTCTTACCGTCTTCATGCTGAGCGGGAGACCGGGGTCTCTGAGCCTCGTGGCTGAGAGGAGACGCGCTGAGGAG
nachra7_Frag675-840_insertion834	GTACATGAACGTCTCCGACCATGCGCCGCAGGACGCTCTACTATGGCCTCAACCTGCTGATCCCCTGTGTGCTCATCTCCGCCCTCGCCCTGCTGGTGTTCCTGCTTCCTGCAGATTCCGGGGAGAAGATTTCCCTGGGGATAACAGTCTTACTCTCTCTTACCGTCTTCATGCTGCTCAGCGGGAGACCGGGGTCTCTGAGCGTGGCTGAGAGGAGACGCGCTGAGGAG
nachra7_Frag675-840_insertion837	GTACATGAACGTCTCCGACCATGCGCCGCAGGACGCTCTACTATGGCCTCAACCTGCTGATCCCCTGTGTGCTCATCTCCGCCCTCGCCCTGCTGGTGTTCCTGCTTCCTGCAGATTCCGGGGAGAAGATTTCCCTGGGGATAACAGTCTTACTCTCTCTTACCGTCTTCATGCTGCTCGTGAGCGGGAGACCGGGGTCTCTGAGCGCTGAGAGGAGACGCGCTGAGGAG
nachra7_Frag675-840_insertion840	GTACATGAACGTCTCCGACCATGCGCCGCAGGACGCTCTACTATGGCCTCAACCTGCTGATCCCCTGTGTGCTCATCTCCGCCCTCGCCCTGCTGGTGTTCCTGCTTCCTGCAGATTCCGGGGAGAAGATTTCCCTGGGGATAACAGTCTTACTCTCTCTTACCGTCTTCATGCTGCTCGTGGCTAGCGGGAGACCGGGGTCTCTGAGCGAGAGGAGACGCGCTGAGGAG
nachra7_Frag843-1008_insertion843	GGCGAGAGGCGTCTCCTGAGAGCGGGAGACCGGGGTCTCTGAGCATCATGCCCGCAACATCCGATTCGGTACCATTGATAGCCCAGTACTTCGCCAGCACCATGATCATCGTGGGCCTCTCGGTGGTGGTGACAGTGATCGTGCTGCAGTACCACCACCACGACCCCGACGGGGGCAAGATGCCCAAGTGGACCAGAGTCATCCTTCTGAACTGGAGACGGTGCACAGCT
nachra7_Frag843-1008_insertion846	GGCGAGAGGCGTCTCCTGAGATCAGCGGGAGACCGGGGTCTCTGAGCATGCCCGCAACATCCGATTCGGTACCATTGATAGCCCAGTACTTCGCCAGCACCATGATCATCGTGGGCCTCTCGGTGGTGGTGACAGTGATCGTGCTGCAGTACCACCACCACGACCCCGACGGGGGCAAGATGCCCAAGTGGACCAGAGTCATCCTTCTGAACTGGAGACGGTGCACAGCT
nachra7_Frag843-1008_insertion849	GGCGAGAGGCGTCTCCTGAGATCATGAGCGGGAGACCGGGGTCTCTGAGCCCCGCAACATCCGATTCGGTACCATTGATAGCCCAGTACTTCGCCAGCACCATGATCATCGTGGGCCTCTCGGTGGTGGTGACAGTGATCGTGCTGCAGTACCACCACCACGACCCCGACGGGGGCAAGATGCCCAAGTGGACCAGAGTCATCCTTCTGAACTGGAGACGGTGCACAGCT
nachra7_Frag843-1008_insertion852	GGCGAGAGGCGTCTCCTGAGATCATGCCCAGCGGGAGACCGGGGTCTCTGAGCGCAACATCCGATTCGGTACCATTGATAGCCCAGTACTTCGCCAGCACCATGATCATCGTGGGCCTCTCGGTGGTGGTGACAGTGATCGTGCTGCAGTACCACCACCACGACCCCGACGGGGGCAAGATGCCCAAGTGGACCAGAGTCATCCTTCTGAACTGGAGACGGTGCACAGCT
nachra7_Frag843-1008_insertion855	GGCGAGAGGCGTCTCCTGAGATCATGCCCGCAAGCGGGAGACCGGGGTCTCTGAGCACATCCGATTCGGTACCATTGATAGCCCAGTACTTCGCCAGCACCATGATCATCGTGGGCCTCTCGGTGGTGGTGACAGTGATCGTGCTGCAGTACCACCACCACGACCCCGACGGGGGCAAGATGCCCAAGTGGACCAGAGTCATCCTTCTGAACTGGAGACGGTGCACAGCT
nachra7_Frag843-1008_insertion858	GGCGAGAGGCGTCTCCTGAGATCATGCCCGCAACAAGCGGGAGACCGGGGTCTCTGAGCTCCGATTCGGTACCATTGATAGCCCAGTACTTCGCCAGCACCATGATCATCGTGGGCCTCTCGGTGGTGGTGACAGTGATCGTGCTGCAGTACCACCACCACGACCCCGACGGGGGCAAGATGCCCAAGTGGACCAGAGTCATCCTTCTGAACTGGAGACGGTGCACAGCT
nachra7_Frag843-1008_insertion861	GGCGAGAGGCGTCTCCTGAGATCATGCCCGCAACATCCAGCGGGAGACCGGGGTCTCTGAGCGATTCGGTACCATTGATAGCCCAGTACTTCGCCAGCACCATGATCATCGTGGGCCTCTCGGTGGTGGTGACAGTGATCGTGCTGCAGTACCACCACCACGACCCCGACGGGGGCAAGATGCCCAAGTGGACCAGAGTCATCCTTCTGAACTGGAGACGGTGCACAGCT
nachra7_Frag843-1008_insertion864	GGCGAGAGGCGTCTCCTGAGATCATGCCCGCAACATCCGATAGCGGGAGACCGGGGTCTCTGAGCTCGGTACCATTGATAGCCCAGTACTTCGCCAGCACCATGATCATCGTGGGCCTCTCGGTGGTGGTGACAGTGATCGTGCTGCAGTACCACCACCACGACCCCGACGGGGGCAAGATGCCCAAGTGGACCAGAGTCATCCTTCTGAACTGGAGACGGTGCACAGCT
nachra7_Frag843-1008_insertion867	GGCGAGAGGCGTCTCCTGAGATCATGCCCGCAACATCCGATTCGAGCGGGAGACCGGGGTCTCTGAGCGTACCATTGATAGCCCAGTACTTCGCCAGCACCATGATCATCGTGGGCCTCTCGGTGGTGGTGACAGTGATCGTGCTGCAGTACCACCACCACGACCCCGACGGGGGCAAGATGCCCAAGTGGACCAGAGTCATCCTTCTGAACTGGAGACGGTGCACAGCT
nachra7_Frag843-1008_insertion870	GGCGAGAGGCGTCTCCTGAGATCATGCCCGCAACATCCGATTCGGTAAGCGGGAGACCGGGGTCTCTGAGCCCATTGATAGCCCAGTACTTCGCCAGCACCATGATCATCGTGGGCCTCTCGGTGGTGGTGACAGTGATCGTGCTGCAGTACCACCACCACGACCCCGACGGGGGCAAGATGCCCAAGTGGACCAGAGTCATCCTTCTGAACTGGAGACGGTGCACAGCT
nachra7_Frag843-1008_insertion873	GGCGAGAGGCGTCTCCTGAGATCATGCCCGCAACATCCGATTCGGTACCAAGCGGGAGACCGGGGTCTCTGAGCTTGATAGCCCAGTACTTCGCCAGCACCATGATCATCGTGGGCCTCTCGGTGGTGGTGACAGTGATCGTGCTGCAGTACCACCACCACGACCCCGACGGGGGCAAGATGCCCAAGTGGACCAGAGTCATCCTTCTGAACTGGAGACGGTGCACAGCT
nachra7_Frag843-1008_insertion876	GGCGAGAGGCGTCTCCTGAGATCATGCCCGCAACATCCGATTCGGTACCATTGAGCGGGAGACCGGGGTCTCTGAGCATAGCCCAGTACTTCGCCAGCACCATGATCATCGTGGGCCTCTCGGTGGTGGTGACAGTGATCGTGCTGCAGTACCACCACCACGACCCCGACGGGGGCAAGATGCCCAAGTGGACCAGAGTCATCCTTCTGAACTGGAGACGGTGCACAGCT
nachra7_Frag843-1008_insertion879	GGCGAGAGGCGTCTCCTGAGATCATGCCCGCAACATCCGATTCGGTACCATTGATAAGCGGGAGACCGGGGTCTCTGAGCGCCCAGTACTTCGCCAGCACCATGATCATCGTGGGCCTCTCGGTGGTGGTGACAGTGATCGTGCTGCAGTACCACCACCACGACCCCGACGGGGGCAAGATGCCCAAGTGGACCAGAGTCATCCTTCTGAACTGGAGACGGTGCACAGCT
nachra7_Frag843-1008_insertion882	GGCGAGAGGCGTCTCCTGAGATCATGCCCGCAACATCCGATTCGGTACCATTGATAGCCAGCGGGAGACCGGGGTCTCTGAGCCAGTACTTCGCCAGCACCATGATCATCGTGGGCCTCTCGGTGGTGGTGACAGTGATCGTGCTGCAGTACCACCACCACGACCCCGACGGGGGCAAGATGCCCAAGTGGACCAGAGTCATCCTTCTGAACTGGAGACGGTGCACAGCT
nachra7_Frag843-1008_insertion885	GGCGAGAGGCGTCTCCTGAGATCATGCCCGCAACATCCGATTCGGTACCATTGATAGCCCAGAGCGGGAGACCGGGGTCTCTGAGCTACTTCGCCAGCACCATGATCATCGTGGGCCTCTCGGTGGTGGTGACAGTGATCGTGCTGCAGTACCACCACCACGACCCCGACGGGGGCAAGATGCCCAAGTGGACCAGAGTCATCCTTCTGAACTGGAGACGGTGCACAGCT
nachra7_Frag843-1008_insertion888	GGCGAGAGGCGTCTCCTGAGATCATGCCCGCAACATCCGATTCGGTACCATTGATAGCCCAGTACAGCGGGAGACCGGGGTCTCTGAGCTTCGCCAGCACCATGATCATCGTGGGCCTCTCGGTGGTGGTGACAGTGATCGTGCTGCAGTACCACCACCACGACCCCGACGGGGGCAAGATGCCCAAGTGGACCAGAGTCATCCTTCTGAACTGGAGACGGTGCACAGCT
nachra7_Frag843-1008_insertion891	GGCGAGAGGCGTCTCCTGAGATCATGCCCGCAACATCCGATTCGGTACCATTGATAGCCCAGTACTTCAGCGGGAGACCGGGGTCTCTGAGCGCCAGCACCATGATCATCGTGGGCCTCTCGGTGGTGGTGACAGTGATCGTGCTGCAGTACCACCACCACGACCCCGACGGGGGCAAGATGCCCAAGTGGACCAGAGTCATCCTTCTGAACTGGAGACGGTGCACAGCT
nachra7_Frag843-1008_insertion894	GGCGAGAGGCGTCTCCTGAGATCATGCCCGCAACATCCGATTCGGTACCATTGATAGCCCAGTACTTCGCCAGCGGGAGACCGGGGTCTCTGAGCAGCACCATGATCATCGTGGGCCTCTCGGTGGTGGTGACAGTGATCGTGCTGCAGTACCACCACCACGACCCCGACGGGGGCAAGATGCCCAAGTGGACCAGAGTCATCCTTCTGAACTGGAGACGGTGCACAGCT
nachra7_Frag843-1008_insertion897	GGCGAGAGGCGTCTCCTGAGATCATGCCCGCAACATCCGATTCGGTACCATTGATAGCCCAGTACTTCGCCAGCAGCGGGAGACCGGGGTCTCTGAGCACCATGATCATCGTGGGCCTCTCGGTGGTGGTGACAGTGATCGTGCTGCAGTACCACCACCACGACCCCGACGGGGGCAAGATGCCCAAGTGGACCAGAGTCATCCTTCTGAACTGGAGACGGTGCACAGCT
nachra7_Frag843-1008_insertion900	GGCGAGAGGCGTCTCCTGAGATCATGCCCGCAACATCCGATTCGGTACCATTGATAGCCCAGTACTTCGCCAGCACCAGCGGGAGACCGGGGTCTCTGAGCATGATCATCGTGGGCCTCTCGGTGGTGGTGACAGTGATCGTGCTGCAGTACCACCACCACGACCCCGACGGGGGCAAGATGCCCAAGTGGACCAGAGTCATCCTTCTGAACTGGAGACGGTGCACAGCT
nachra7_Frag843-1008_insertion903	GGCGAGAGGCGTCTCCTGAGATCATGCCCGCAACATCCGATTCGGTACCATTGATAGCCCAGTACTTCGCCAGCACCATGAGCGGGAGACCGGGGTCTCTGAGCATCATCGTGGGCCTCTCGGTGGTGGTGACAGTGATCGTGCTGCAGTACCACCACCACGACCCCGACGGGGGCAAGATGCCCAAGTGGACCAGAGTCATCCTTCTGAACTGGAGACGGTGCACAGCT
nachra7_Frag843-1008_insertion906	GGCGAGAGGCGTCTCCTGAGATCATGCCCGCAACATCCGATTCGGTACCATTGATAGCCCAGTACTTCGCCAGCACCATGATCAGCGGGAGACCGGGGTCTCTGAGCATCGTGGGCCTCTCGGTGGTGGTGACAGTGATCGTGCTGCAGTACCACCACCACGACCCCGACGGGGGCAAGATGCCCAAGTGGACCAGAGTCATCCTTCTGAACTGGAGACGGTGCACAGCT
nachra7_Frag843-1008_insertion909	GGCGAGAGGCGTCTCCTGAGATCATGCCCGCAACATCCGATTCGGTACCATTGATAGCCCAGTACTTCGCCAGCACCATGATCATCAGCGGGAGACCGGGGTCTCTGAGCGTGGGCCTCTCGGTGGTGGTGACAGTGATCGTGCTGCAGTACCACCACCACGACCCCGACGGGGGCAAGATGCCCAAGTGGACCAGAGTCATCCTTCTGAACTGGAGACGGTGCACAGCT
nachra7_Frag843-1008_insertion912	GGCGAGAGGCGTCTCCTGAGATCATGCCCGCAACATCCGATTCGGTACCATTGATAGCCCAGTACTTCGCCAGCACCATGATCATCGTGAGCGGGAGACCGGGGTCTCTGAGCGGCCTCTCGGTGGTGGTGACAGTGATCGTGCTGCAGTACCACCACCACGACCCCGACGGGGGCAAGATGCCCAAGTGGACCAGAGTCATCCTTCTGAACTGGAGACGGTGCACAGCT
nachra7_Frag843-1008_insertion915	GGCGAGAGGCGTCTCCTGAGATCATGCCCGCAACATCCGATTCGGTACCATTGATAGCCCAGTACTTCGCCAGCACCATGATCATCGTGGGCAGCGGGAGACCGGGGTCTCTGAGCCTCTCGGTGGTGGTGACAGTGATCGTGCTGCAGTACCACCACCACGACCCCGACGGGGGCAAGATGCCCAAGTGGACCAGAGTCATCCTTCTGAACTGGAGACGGTGCACAGCT
nachra7_Frag843-1008_insertion918	GGCGAGAGGCGTCTCCTGAGATCATGCCCGCAACATCCGATTCGGTACCATTGATAGCCCAGTACTTCGCCAGCACCATGATCATCGTGGGCCTCAGCGGGAGACCGGGGTCTCTGAGCTCGGTGGTGGTGACAGTGATCGTGCTGCAGTACCACCACCACGACCCCGACGGGGGCAAGATGCCCAAGTGGACCAGAGTCATCCTTCTGAACTGGAGACGGTGCACAGCT
nachra7_Frag843-1008_insertion921	GGCGAGAGGCGTCTCCTGAGATCATGCCCGCAACATCCGATTCGGTACCATTGATAGCCCAGTACTTCGCCAGCACCATGATCATCGTGGGCCTCTCGAGCGGGAGACCGGGGTCTCTGAGCGTGGTGGTGACAGTGATCGTGCTGCAGTACCACCACCACGACCCCGACGGGGGCAAGATGCCCAAGTGGACCAGAGTCATCCTTCTGAACTGGAGACGGTGCACAGCT
nachra7_Frag843-1008_insertion924	GGCGAGAGGCGTCTCCTGAGATCATGCCCGCAACATCCGATTCGGTACCATTGATAGCCCAGTACTTCGCCAGCACCATGATCATCGTGGGCCTCTCGGTGAGCGGGAGACCGGGGTCTCTGAGCGTGGTGACAGTGATCGTGCTGCAGTACCACCACCACGACCCCGACGGGGGCAAGATGCCCAAGTGGACCAGAGTCATCCTTCTGAACTGGAGACGGTGCACAGCT
nachra7_Frag843-1008_insertion927	GGCGAGAGGCGTCTCCTGAGATCATGCCCGCAACATCCGATTCGGTACCATTGATAGCCCAGTACTTCGCCAGCACCATGATCATCGTGGGCCTCTCGGTGGTGAGCGGGAGACCGGGGTCTCTGAGCGTGACAGTGATCGTGCTGCAGTACCACCACCACGACCCCGACGGGGGCAAGATGCCCAAGTGGACCAGAGTCATCCTTCTGAACTGGAGACGGTGCACAGCT
nachra7_Frag843-1008_insertion930	GGCGAGAGGCGTCTCCTGAGATCATGCCCGCAACATCCGATTCGGTACCATTGATAGCCCAGTACTTCGCCAGCACCATGATCATCGTGGGCCTCTCGGTGGTGGTGAGCGGGAGACCGGGGTCTCTGAGCACAGTGATCGTGCTGCAGTACCACCACCACGACCCCGACGGGGGCAAGATGCCCAAGTGGACCAGAGTCATCCTTCTGAACTGGAGACGGTGCACAGCT
nachra7_Frag843-1008_insertion933	GGCGAGAGGCGTCTCCTGAGATCATGCCCGCAACATCCGATTCGGTACCATTGATAGCCCAGTACTTCGCCAGCACCATGATCATCGTGGGCCTCTCGGTGGTGGTGACAAGCGGGAGACCGGGGTCTCTGAGCGTGATCGTGCTGCAGTACCACCACCACGACCCCGACGGGGGCAAGATGCCCAAGTGGACCAGAGTCATCCTTCTGAACTGGAGACGGTGCACAGCT
nachra7_Frag843-1008_insertion936	GGCGAGAGGCGTCTCCTGAGATCATGCCCGCAACATCCGATTCGGTACCATTGATAGCCCAGTACTTCGCCAGCACCATGATCATCGTGGGCCTCTCGGTGGTGGTGACAGTGAGCGGGAGACCGGGGTCTCTGAGCATCGTGCTGCAGTACCACCACCACGACCCCGACGGGGGCAAGATGCCCAAGTGGACCAGAGTCATCCTTCTGAACTGGAGACGGTGCACAGCT
nachra7_Frag843-1008_insertion939	GGCGAGAGGCGTCTCCTGAGATCATGCCCGCAACATCCGATTCGGTACCATTGATAGCCCAGTACTTCGCCAGCACCATGATCATCGTGGGCCTCTCGGTGGTGGTGACAGTGATCAGCGGGAGACCGGGGTCTCTGAGCGTGCTGCAGTACCACCACCACGACCCCGACGGGGGCAAGATGCCCAAGTGGACCAGAGTCATCCTTCTGAACTGGAGACGGTGCACAGCT
nachra7_Frag843-1008_insertion942	GGCGAGAGGCGTCTCCTGAGATCATGCCCGCAACATCCGATTCGGTACCATTGATAGCCCAGTACTTCGCCAGCACCATGATCATCGTGGGCCTCTCGGTGGTGGTGACAGTGATCGTGAGCGGGAGACCGGGGTCTCTGAGCCTGCAGTACCACCACCACGACCCCGACGGGGGCAAGATGCCCAAGTGGACCAGAGTCATCCTTCTGAACTGGAGACGGTGCACAGCT
nachra7_Frag843-1008_insertion945	GGCGAGAGGCGTCTCCTGAGATCATGCCCGCAACATCCGATTCGGTACCATTGATAGCCCAGTACTTCGCCAGCACCATGATCATCGTGGGCCTCTCGGTGGTGGTGACAGTGATCGTGCTGAGCGGGAGACCGGGGTCTCTGAGCCAGTACCACCACCACGACCCCGACGGGGGCAAGATGCCCAAGTGGACCAGAGTCATCCTTCTGAACTGGAGACGGTGCACAGCT
nachra7_Frag843-1008_insertion948	GGCGAGAGGCGTCTCCTGAGATCATGCCCGCAACATCCGATTCGGTACCATTGATAGCCCAGTACTTCGCCAGCACCATGATCATCGTGGGCCTCTCGGTGGTGGTGACAGTGATCGTGCTGCAGAGCGGGAGACCGGGGTCTCTGAGCTACCACCACCACGACCCCGACGGGGGCAAGATGCCCAAGTGGACCAGAGTCATCCTTCTGAACTGGAGACGGTGCACAGCT
nachra7_Frag843-1008_insertion951	GGCGAGAGGCGTCTCCTGAGATCATGCCCGCAACATCCGATTCGGTACCATTGATAGCCCAGTACTTCGCCAGCACCATGATCATCGTGGGCCTCTCGGTGGTGGTGACAGTGATCGTGCTGCAGTACAGCGGGAGACCGGGGTCTCTGAGCCACCACCACGACCCCGACGGGGGCAAGATGCCCAAGTGGACCAGAGTCATCCTTCTGAACTGGAGACGGTGCACAGCT
nachra7_Frag843-1008_insertion954	GGCGAGAGGCGTCTCCTGAGATCATGCCCGCAACATCCGATTCGGTACCATTGATAGCCCAGTACTTCGCCAGCACCATGATCATCGTGGGCCTCTCGGTGGTGGTGACAGTGATCGTGCTGCAGTACCACAGCGGGAGACCGGGGTCTCTGAGCCACCACGACCCCGACGGGGGCAAGATGCCCAAGTGGACCAGAGTCATCCTTCTGAACTGGAGACGGTGCACAGCT
nachra7_Frag843-1008_insertion957	GGCGAGAGGCGTCTCCTGAGATCATGCCCGCAACATCCGATTCGGTACCATTGATAGCCCAGTACTTCGCCAGCACCATGATCATCGTGGGCCTCTCGGTGGTGGTGACAGTGATCGTGCTGCAGTACCACCACAGCGGGAGACCGGGGTCTCTGAGCCACGACCCCGACGGGGGCAAGATGCCCAAGTGGACCAGAGTCATCCTTCTGAACTGGAGACGGTGCACAGCT
nachra7_Frag843-1008_insertion960	GGCGAGAGGCGTCTCCTGAGATCATGCCCGCAACATCCGATTCGGTACCATTGATAGCCCAGTACTTCGCCAGCACCATGATCATCGTGGGCCTCTCGGTGGTGGTGACAGTGATCGTGCTGCAGTACCACCACCACAGCGGGAGACCGGGGTCTCTGAGCGACCCCGACGGGGGCAAGATGCCCAAGTGGACCAGAGTCATCCTTCTGAACTGGAGACGGTGCACAGCT
nachra7_Frag843-1008_insertion963	GGCGAGAGGCGTCTCCTGAGATCATGCCCGCAACATCCGATTCGGTACCATTGATAGCCCAGTACTTCGCCAGCACCATGATCATCGTGGGCCTCTCGGTGGTGGTGACAGTGATCGTGCTGCAGTACCACCACCACGACAGCGGGAGACCGGGGTCTCTGAGCCCCGACGGGGGCAAGATGCCCAAGTGGACCAGAGTCATCCTTCTGAACTGGAGACGGTGCACAGCT
nachra7_Frag843-1008_insertion966	GGCGAGAGGCGTCTCCTGAGATCATGCCCGCAACATCCGATTCGGTACCATTGATAGCCCAGTACTTCGCCAGCACCATGATCATCGTGGGCCTCTCGGTGGTGGTGACAGTGATCGTGCTGCAGTACCACCACCACGACCCCAGCGGGAGACCGGGGTCTCTGAGCGACGGGGGCAAGATGCCCAAGTGGACCAGAGTCATCCTTCTGAACTGGAGACGGTGCACAGCT
nachra7_Frag843-1008_insertion969	GGCGAGAGGCGTCTCCTGAGATCATGCCCGCAACATCCGATTCGGTACCATTGATAGCCCAGTACTTCGCCAGCACCATGATCATCGTGGGCCTCTCGGTGGTGGTGACAGTGATCGTGCTGCAGTACCACCACCACGACCCCGACAGCGGGAGACCGGGGTCTCTGAGCGGGGGCAAGATGCCCAAGTGGACCAGAGTCATCCTTCTGAACTGGAGACGGTGCACAGCT
nachra7_Frag843-1008_insertion972	GGCGAGAGGCGTCTCCTGAGATCATGCCCGCAACATCCGATTCGGTACCATTGATAGCCCAGTACTTCGCCAGCACCATGATCATCGTGGGCCTCTCGGTGGTGGTGACAGTGATCGTGCTGCAGTACCACCACCACGACCCCGACGGGAGCGGGAGACCGGGGTCTCTGAGCGGCAAGATGCCCAAGTGGACCAGAGTCATCCTTCTGAACTGGAGACGGTGCACAGCT
nachra7_Frag843-1008_insertion975	GGCGAGAGGCGTCTCCTGAGATCATGCCCGCAACATCCGATTCGGTACCATTGATAGCCCAGTACTTCGCCAGCACCATGATCATCGTGGGCCTCTCGGTGGTGGTGACAGTGATCGTGCTGCAGTACCACCACCACGACCCCGACGGGGGCAGCGGGAGACCGGGGTCTCTGAGCAAGATGCCCAAGTGGACCAGAGTCATCCTTCTGAACTGGAGACGGTGCACAGCT
nachra7_Frag843-1008_insertion978	GGCGAGAGGCGTCTCCTGAGATCATGCCCGCAACATCCGATTCGGTACCATTGATAGCCCAGTACTTCGCCAGCACCATGATCATCGTGGGCCTCTCGGTGGTGGTGACAGTGATCGTGCTGCAGTACCACCACCACGACCCCGACGGGGGCAAGAGCGGGAGACCGGGGTCTCTGAGCATGCCCAAGTGGACCAGAGTCATCCTTCTGAACTGGAGACGGTGCACAGCT
nachra7_Frag843-1008_insertion981	GGCGAGAGGCGTCTCCTGAGATCATGCCCGCAACATCCGATTCGGTACCATTGATAGCCCAGTACTTCGCCAGCACCATGATCATCGTGGGCCTCTCGGTGGTGGTGACAGTGATCGTGCTGCAGTACCACCACCACGACCCCGACGGGGGCAAGATGAGCGGGAGACCGGGGTCTCTGAGCCCCAAGTGGACCAGAGTCATCCTTCTGAACTGGAGACGGTGCACAGCT
nachra7_Frag843-1008_insertion984	GGCGAGAGGCGTCTCCTGAGATCATGCCCGCAACATCCGATTCGGTACCATTGATAGCCCAGTACTTCGCCAGCACCATGATCATCGTGGGCCTCTCGGTGGTGGTGACAGTGATCGTGCTGCAGTACCACCACCACGACCCCGACGGGGGCAAGATGCCCAGCGGGAGACCGGGGTCTCTGAGCAAGTGGACCAGAGTCATCCTTCTGAACTGGAGACGGTGCACAGCT
nachra7_Frag843-1008_insertion987	GGCGAGAGGCGTCTCCTGAGATCATGCCCGCAACATCCGATTCGGTACCATTGATAGCCCAGTACTTCGCCAGCACCATGATCATCGTGGGCCTCTCGGTGGTGGTGACAGTGATCGTGCTGCAGTACCACCACCACGACCCCGACGGGGGCAAGATGCCCAAGAGCGGGAGACCGGGGTCTCTGAGCTGGACCAGAGTCATCCTTCTGAACTGGAGACGGTGCACAGCT
nachra7_Frag843-1008_insertion990	GGCGAGAGGCGTCTCCTGAGATCATGCCCGCAACATCCGATTCGGTACCATTGATAGCCCAGTACTTCGCCAGCACCATGATCATCGTGGGCCTCTCGGTGGTGGTGACAGTGATCGTGCTGCAGTACCACCACCACGACCCCGACGGGGGCAAGATGCCCAAGTGGAGCGGGAGACCGGGGTCTCTGAGCACCAGAGTCATCCTTCTGAACTGGAGACGGTGCACAGCT
nachra7_Frag843-1008_insertion993	GGCGAGAGGCGTCTCCTGAGATCATGCCCGCAACATCCGATTCGGTACCATTGATAGCCCAGTACTTCGCCAGCACCATGATCATCGTGGGCCTCTCGGTGGTGGTGACAGTGATCGTGCTGCAGTACCACCACCACGACCCCGACGGGGGCAAGATGCCCAAGTGGACCAGCGGGAGACCGGGGTCTCTGAGCAGAGTCATCCTTCTGAACTGGAGACGGTGCACAGCT
nachra7_Frag843-1008_insertion996	GGCGAGAGGCGTCTCCTGAGATCATGCCCGCAACATCCGATTCGGTACCATTGATAGCCCAGTACTTCGCCAGCACCATGATCATCGTGGGCCTCTCGGTGGTGGTGACAGTGATCGTGCTGCAGTACCACCACCACGACCCCGACGGGGGCAAGATGCCCAAGTGGACCAGAAGCGGGAGACCGGGGTCTCTGAGCGTCATCCTTCTGAACTGGAGACGGTGCACAGCT
nachra7_Frag843-1008_insertion999	GGCGAGAGGCGTCTCCTGAGATCATGCCCGCAACATCCGATTCGGTACCATTGATAGCCCAGTACTTCGCCAGCACCATGATCATCGTGGGCCTCTCGGTGGTGGTGACAGTGATCGTGCTGCAGTACCACCACCACGACCCCGACGGGGGCAAGATGCCCAAGTGGACCAGAGTCAGCGGGAGACCGGGGTCTCTGAGCATCCTTCTGAACTGGAGACGGTGCACAGCT
nachra7_Frag843-1008_insertion1002	GGCGAGAGGCGTCTCCTGAGATCATGCCCGCAACATCCGATTCGGTACCATTGATAGCCCAGTACTTCGCCAGCACCATGATCATCGTGGGCCTCTCGGTGGTGGTGACAGTGATCGTGCTGCAGTACCACCACCACGACCCCGACGGGGGCAAGATGCCCAAGTGGACCAGAGTCATCAGCGGGAGACCGGGGTCTCTGAGCCTTCTGAACTGGAGACGGTGCACAGCT
nachra7_Frag843-1008_insertion1005	GGCGAGAGGCGTCTCCTGAGATCATGCCCGCAACATCCGATTCGGTACCATTGATAGCCCAGTACTTCGCCAGCACCATGATCATCGTGGGCCTCTCGGTGGTGGTGACAGTGATCGTGCTGCAGTACCACCACCACGACCCCGACGGGGGCAAGATGCCCAAGTGGACCAGAGTCATCCTTAGCGGGAGACCGGGGTCTCTGAGCCTGAACTGGAGACGGTGCACAGCT
nachra7_Frag843-1008_insertion1008	GGCGAGAGGCGTCTCCTGAGATCATGCCCGCAACATCCGATTCGGTACCATTGATAGCCCAGTACTTCGCCAGCACCATGATCATCGTGGGCCTCTCGGTGGTGGTGACAGTGATCGTGCTGCAGTACCACCACCACGACCCCGACGGGGGCAAGATGCCCAAGTGGACCAGAGTCATCCTTCTGAGCGGGAGACCGGGGTCTCTGAGCAACTGGAGACGGTGCACAGCT
nachra7_Frag1011-1176_insertion1011	CCCGATAGCCGTCTCCGAACAGCGGGAGACCGGGGTCTCTGAGCTGGTGCGCGTGGTTCCTGCGAATGAAGAGGCCCGGGGAGGACAAGGTGCGCCCGGCCTGCCAGCACAAGCAGCGGCGCTGCAGCCTGGCCAGTGTGGAGATGAGCGCCGTGGCGCCGCGCCCGCCAGCAACGGGAACCTGCTGTACATCGGCTTCCGCGGCCTGGACGGGAGACGACAGGCGTGT
nachra7_Frag1011-1176_insertion1014	CCCGATAGCCGTCTCCGAACTGGAGCGGGAGACCGGGGTCTCTGAGCTGCGCGTGGTTCCTGCGAATGAAGAGGCCCGGGGAGGACAAGGTGCGCCCGGCCTGCCAGCACAAGCAGCGGCGCTGCAGCCTGGCCAGTGTGGAGATGAGCGCCGTGGCGCCGCGCCCGCCAGCAACGGGAACCTGCTGTACATCGGCTTCCGCGGCCTGGACGGGAGACGACAGGCGTGT
nachra7_Frag1011-1176_insertion1017	CCCGATAGCCGTCTCCGAACTGGTGCAGCGGGAGACCGGGGTCTCTGAGCGCGTGGTTCCTGCGAATGAAGAGGCCCGGGGAGGACAAGGTGCGCCCGGCCTGCCAGCACAAGCAGCGGCGCTGCAGCCTGGCCAGTGTGGAGATGAGCGCCGTGGCGCCGCGCCCGCCAGCAACGGGAACCTGCTGTACATCGGCTTCCGCGGCCTGGACGGGAGACGACAGGCGTGT
nachra7_Frag1011-1176_insertion1020	CCCGATAGCCGTCTCCGAACTGGTGCGCGAGCGGGAGACCGGGGTCTCTGAGCTGGTTCCTGCGAATGAAGAGGCCCGGGGAGGACAAGGTGCGCCCGGCCTGCCAGCACAAGCAGCGGCGCTGCAGCCTGGCCAGTGTGGAGATGAGCGCCGTGGCGCCGCGCCCGCCAGCAACGGGAACCTGCTGTACATCGGCTTCCGCGGCCTGGACGGGAGACGACAGGCGTGT
nachra7_Frag1011-1176_insertion1023	CCCGATAGCCGTCTCCGAACTGGTGCGCGTGGAGCGGGAGACCGGGGTCTCTGAGCTTCCTGCGAATGAAGAGGCCCGGGGAGGACAAGGTGCGCCCGGCCTGCCAGCACAAGCAGCGGCGCTGCAGCCTGGCCAGTGTGGAGATGAGCGCCGTGGCGCCGCGCCCGCCAGCAACGGGAACCTGCTGTACATCGGCTTCCGCGGCCTGGACGGGAGACGACAGGCGTGT
nachra7_Frag1011-1176_insertion1026	CCCGATAGCCGTCTCCGAACTGGTGCGCGTGGTTCAGCGGGAGACCGGGGTCTCTGAGCCTGCGAATGAAGAGGCCCGGGGAGGACAAGGTGCGCCCGGCCTGCCAGCACAAGCAGCGGCGCTGCAGCCTGGCCAGTGTGGAGATGAGCGCCGTGGCGCCGCGCCCGCCAGCAACGGGAACCTGCTGTACATCGGCTTCCGCGGCCTGGACGGGAGACGACAGGCGTGT
nachra7_Frag1011-1176_insertion1029	CCCGATAGCCGTCTCCGAACTGGTGCGCGTGGTTCCTGAGCGGGAGACCGGGGTCTCTGAGCCGAATGAAGAGGCCCGGGGAGGACAAGGTGCGCCCGGCCTGCCAGCACAAGCAGCGGCGCTGCAGCCTGGCCAGTGTGGAGATGAGCGCCGTGGCGCCGCGCCCGCCAGCAACGGGAACCTGCTGTACATCGGCTTCCGCGGCCTGGACGGGAGACGACAGGCGTGT
nachra7_Frag1011-1176_insertion1032	CCCGATAGCCGTCTCCGAACTGGTGCGCGTGGTTCCTGCGAAGCGGGAGACCGGGGTCTCTGAGCATGAAGAGGCCCGGGGAGGACAAGGTGCGCCCGGCCTGCCAGCACAAGCAGCGGCGCTGCAGCCTGGCCAGTGTGGAGATGAGCGCCGTGGCGCCGCGCCCGCCAGCAACGGGAACCTGCTGTACATCGGCTTCCGCGGCCTGGACGGGAGACGACAGGCGTGT
nachra7_Frag1011-1176_insertion1035	CCCGATAGCCGTCTCCGAACTGGTGCGCGTGGTTCCTGCGAATGAGCGGGAGACCGGGGTCTCTGAGCAAGAGGCCCGGGGAGGACAAGGTGCGCCCGGCCTGCCAGCACAAGCAGCGGCGCTGCAGCCTGGCCAGTGTGGAGATGAGCGCCGTGGCGCCGCGCCCGCCAGCAACGGGAACCTGCTGTACATCGGCTTCCGCGGCCTGGACGGGAGACGACAGGCGTGT
nachra7_Frag1011-1176_insertion1038	CCCGATAGCCGTCTCCGAACTGGTGCGCGTGGTTCCTGCGAATGAAGAGCGGGAGACCGGGGTCTCTGAGCAGGCCCGGGGAGGACAAGGTGCGCCCGGCCTGCCAGCACAAGCAGCGGCGCTGCAGCCTGGCCAGTGTGGAGATGAGCGCCGTGGCGCCGCGCCCGCCAGCAACGGGAACCTGCTGTACATCGGCTTCCGCGGCCTGGACGGGAGACGACAGGCGTGT
nachra7_Frag1011-1176_insertion1041	CCCGATAGCCGTCTCCGAACTGGTGCGCGTGGTTCCTGCGAATGAAGAGGAGCGGGAGACCGGGGTCTCTGAGCCCCGGGGAGGACAAGGTGCGCCCGGCCTGCCAGCACAAGCAGCGGCGCTGCAGCCTGGCCAGTGTGGAGATGAGCGCCGTGGCGCCGCGCCCGCCAGCAACGGGAACCTGCTGTACATCGGCTTCCGCGGCCTGGACGGGAGACGACAGGCGTGT
nachra7_Frag1011-1176_insertion1044	CCCGATAGCCGTCTCCGAACTGGTGCGCGTGGTTCCTGCGAATGAAGAGGCCCAGCGGGAGACCGGGGTCTCTGAGCGGGGAGGACAAGGTGCGCCCGGCCTGCCAGCACAAGCAGCGGCGCTGCAGCCTGGCCAGTGTGGAGATGAGCGCCGTGGCGCCGCGCCCGCCAGCAACGGGAACCTGCTGTACATCGGCTTCCGCGGCCTGGACGGGAGACGACAGGCGTGT
nachra7_Frag1011-1176_insertion1047	CCCGATAGCCGTCTCCGAACTGGTGCGCGTGGTTCCTGCGAATGAAGAGGCCCGGGAGCGGGAGACCGGGGTCTCTGAGCGAGGACAAGGTGCGCCCGGCCTGCCAGCACAAGCAGCGGCGCTGCAGCCTGGCCAGTGTGGAGATGAGCGCCGTGGCGCCGCGCCCGCCAGCAACGGGAACCTGCTGTACATCGGCTTCCGCGGCCTGGACGGGAGACGACAGGCGTGT
nachra7_Frag1011-1176_insertion1050	CCCGATAGCCGTCTCCGAACTGGTGCGCGTGGTTCCTGCGAATGAAGAGGCCCGGGGAGAGCGGGAGACCGGGGTCTCTGAGCGACAAGGTGCGCCCGGCCTGCCAGCACAAGCAGCGGCGCTGCAGCCTGGCCAGTGTGGAGATGAGCGCCGTGGCGCCGCGCCCGCCAGCAACGGGAACCTGCTGTACATCGGCTTCCGCGGCCTGGACGGGAGACGACAGGCGTGT
nachra7_Frag1011-1176_insertion1053	CCCGATAGCCGTCTCCGAACTGGTGCGCGTGGTTCCTGCGAATGAAGAGGCCCGGGGAGGACAGCGGGAGACCGGGGTCTCTGAGCAAGGTGCGCCCGGCCTGCCAGCACAAGCAGCGGCGCTGCAGCCTGGCCAGTGTGGAGATGAGCGCCGTGGCGCCGCGCCCGCCAGCAACGGGAACCTGCTGTACATCGGCTTCCGCGGCCTGGACGGGAGACGACAGGCGTGT
nachra7_Frag1011-1176_insertion1056	CCCGATAGCCGTCTCCGAACTGGTGCGCGTGGTTCCTGCGAATGAAGAGGCCCGGGGAGGACAAGAGCGGGAGACCGGGGTCTCTGAGCGTGCGCCCGGCCTGCCAGCACAAGCAGCGGCGCTGCAGCCTGGCCAGTGTGGAGATGAGCGCCGTGGCGCCGCGCCCGCCAGCAACGGGAACCTGCTGTACATCGGCTTCCGCGGCCTGGACGGGAGACGACAGGCGTGT
nachra7_Frag1011-1176_insertion1059	CCCGATAGCCGTCTCCGAACTGGTGCGCGTGGTTCCTGCGAATGAAGAGGCCCGGGGAGGACAAGGTGAGCGGGAGACCGGGGTCTCTGAGCCGCCCGGCCTGCCAGCACAAGCAGCGGCGCTGCAGCCTGGCCAGTGTGGAGATGAGCGCCGTGGCGCCGCGCCCGCCAGCAACGGGAACCTGCTGTACATCGGCTTCCGCGGCCTGGACGGGAGACGACAGGCGTGT
nachra7_Frag1011-1176_insertion1062	CCCGATAGCCGTCTCCGAACTGGTGCGCGTGGTTCCTGCGAATGAAGAGGCCCGGGGAGGACAAGGTGCGCAGCGGGAGACCGGGGTCTCTGAGCCCGGCCTGCCAGCACAAGCAGCGGCGCTGCAGCCTGGCCAGTGTGGAGATGAGCGCCGTGGCGCCGCGCCCGCCAGCAACGGGAACCTGCTGTACATCGGCTTCCGCGGCCTGGACGGGAGACGACAGGCGTGT
nachra7_Frag1011-1176_insertion1065	CCCGATAGCCGTCTCCGAACTGGTGCGCGTGGTTCCTGCGAATGAAGAGGCCCGGGGAGGACAAGGTGCGCCCGAGCGGGAGACCGGGGTCTCTGAGCGCCTGCCAGCACAAGCAGCGGCGCTGCAGCCTGGCCAGTGTGGAGATGAGCGCCGTGGCGCCGCGCCCGCCAGCAACGGGAACCTGCTGTACATCGGCTTCCGCGGCCTGGACGGGAGACGACAGGCGTGT
nachra7_Frag1011-1176_insertion1068	CCCGATAGCCGTCTCCGAACTGGTGCGCGTGGTTCCTGCGAATGAAGAGGCCCGGGGAGGACAAGGTGCGCCCGGCCAGCGGGAGACCGGGGTCTCTGAGCTGCCAGCACAAGCAGCGGCGCTGCAGCCTGGCCAGTGTGGAGATGAGCGCCGTGGCGCCGCGCCCGCCAGCAACGGGAACCTGCTGTACATCGGCTTCCGCGGCCTGGACGGGAGACGACAGGCGTGT
nachra7_Frag1011-1176_insertion1071	CCCGATAGCCGTCTCCGAACTGGTGCGCGTGGTTCCTGCGAATGAAGAGGCCCGGGGAGGACAAGGTGCGCCCGGCCTGCAGCGGGAGACCGGGGTCTCTGAGCCAGCACAAGCAGCGGCGCTGCAGCCTGGCCAGTGTGGAGATGAGCGCCGTGGCGCCGCGCCCGCCAGCAACGGGAACCTGCTGTACATCGGCTTCCGCGGCCTGGACGGGAGACGACAGGCGTGT
nachra7_Frag1011-1176_insertion1074	CCCGATAGCCGTCTCCGAACTGGTGCGCGTGGTTCCTGCGAATGAAGAGGCCCGGGGAGGACAAGGTGCGCCCGGCCTGCCAGAGCGGGAGACCGGGGTCTCTGAGCCACAAGCAGCGGCGCTGCAGCCTGGCCAGTGTGGAGATGAGCGCCGTGGCGCCGCGCCCGCCAGCAACGGGAACCTGCTGTACATCGGCTTCCGCGGCCTGGACGGGAGACGACAGGCGTGT
nachra7_Frag1011-1176_insertion1077	CCCGATAGCCGTCTCCGAACTGGTGCGCGTGGTTCCTGCGAATGAAGAGGCCCGGGGAGGACAAGGTGCGCCCGGCCTGCCAGCACAGCGGGAGACCGGGGTCTCTGAGCAAGCAGCGGCGCTGCAGCCTGGCCAGTGTGGAGATGAGCGCCGTGGCGCCGCGCCCGCCAGCAACGGGAACCTGCTGTACATCGGCTTCCGCGGCCTGGACGGGAGACGACAGGCGTGT
nachra7_Frag1011-1176_insertion1080	CCCGATAGCCGTCTCCGAACTGGTGCGCGTGGTTCCTGCGAATGAAGAGGCCCGGGGAGGACAAGGTGCGCCCGGCCTGCCAGCACAAGAGCGGGAGACCGGGGTCTCTGAGCCAGCGGCGCTGCAGCCTGGCCAGTGTGGAGATGAGCGCCGTGGCGCCGCGCCCGCCAGCAACGGGAACCTGCTGTACATCGGCTTCCGCGGCCTGGACGGGAGACGACAGGCGTGT
nachra7_Frag1011-1176_insertion1083	CCCGATAGCCGTCTCCGAACTGGTGCGCGTGGTTCCTGCGAATGAAGAGGCCCGGGGAGGACAAGGTGCGCCCGGCCTGCCAGCACAAGCAGAGCGGGAGACCGGGGTCTCTGAGCCGGCGCTGCAGCCTGGCCAGTGTGGAGATGAGCGCCGTGGCGCCGCGCCCGCCAGCAACGGGAACCTGCTGTACATCGGCTTCCGCGGCCTGGACGGGAGACGACAGGCGTGT
nachra7_Frag1011-1176_insertion1086	CCCGATAGCCGTCTCCGAACTGGTGCGCGTGGTTCCTGCGAATGAAGAGGCCCGGGGAGGACAAGGTGCGCCCGGCCTGCCAGCACAAGCAGCGGAGCGGGAGACCGGGGTCTCTGAGCCGCTGCAGCCTGGCCAGTGTGGAGATGAGCGCCGTGGCGCCGCGCCCGCCAGCAACGGGAACCTGCTGTACATCGGCTTCCGCGGCCTGGACGGGAGACGACAGGCGTGT
nachra7_Frag1011-1176_insertion1089	CCCGATAGCCGTCTCCGAACTGGTGCGCGTGGTTCCTGCGAATGAAGAGGCCCGGGGAGGACAAGGTGCGCCCGGCCTGCCAGCACAAGCAGCGGCGCAGCGGGAGACCGGGGTCTCTGAGCTGCAGCCTGGCCAGTGTGGAGATGAGCGCCGTGGCGCCGCGCCCGCCAGCAACGGGAACCTGCTGTACATCGGCTTCCGCGGCCTGGACGGGAGACGACAGGCGTGT
nachra7_Frag1011-1176_insertion1092	CCCGATAGCCGTCTCCGAACTGGTGCGCGTGGTTCCTGCGAATGAAGAGGCCCGGGGAGGACAAGGTGCGCCCGGCCTGCCAGCACAAGCAGCGGCGCTGCAGCGGGAGACCGGGGTCTCTGAGCAGCCTGGCCAGTGTGGAGATGAGCGCCGTGGCGCCGCGCCCGCCAGCAACGGGAACCTGCTGTACATCGGCTTCCGCGGCCTGGACGGGAGACGACAGGCGTGT
nachra7_Frag1011-1176_insertion1095	CCCGATAGCCGTCTCCGAACTGGTGCGCGTGGTTCCTGCGAATGAAGAGGCCCGGGGAGGACAAGGTGCGCCCGGCCTGCCAGCACAAGCAGCGGCGCTGCAGCAGCGGGAGACCGGGGTCTCTGAGCCTGGCCAGTGTGGAGATGAGCGCCGTGGCGCCGCGCCCGCCAGCAACGGGAACCTGCTGTACATCGGCTTCCGCGGCCTGGACGGGAGACGACAGGCGTGT
nachra7_Frag1011-1176_insertion1098	CCCGATAGCCGTCTCCGAACTGGTGCGCGTGGTTCCTGCGAATGAAGAGGCCCGGGGAGGACAAGGTGCGCCCGGCCTGCCAGCACAAGCAGCGGCGCTGCAGCCTGAGCGGGAGACCGGGGTCTCTGAGCGCCAGTGTGGAGATGAGCGCCGTGGCGCCGCGCCCGCCAGCAACGGGAACCTGCTGTACATCGGCTTCCGCGGCCTGGACGGGAGACGACAGGCGTGT
nachra7_Frag1011-1176_insertion1101	CCCGATAGCCGTCTCCGAACTGGTGCGCGTGGTTCCTGCGAATGAAGAGGCCCGGGGAGGACAAGGTGCGCCCGGCCTGCCAGCACAAGCAGCGGCGCTGCAGCCTGGCCAGCGGGAGACCGGGGTCTCTGAGCAGTGTGGAGATGAGCGCCGTGGCGCCGCGCCCGCCAGCAACGGGAACCTGCTGTACATCGGCTTCCGCGGCCTGGACGGGAGACGACAGGCGTGT
nachra7_Frag1011-1176_insertion1104	CCCGATAGCCGTCTCCGAACTGGTGCGCGTGGTTCCTGCGAATGAAGAGGCCCGGGGAGGACAAGGTGCGCCCGGCCTGCCAGCACAAGCAGCGGCGCTGCAGCCTGGCCAGTAGCGGGAGACCGGGGTCTCTGAGCGTGGAGATGAGCGCCGTGGCGCCGCGCCCGCCAGCAACGGGAACCTGCTGTACATCGGCTTCCGCGGCCTGGACGGGAGACGACAGGCGTGT
nachra7_Frag1011-1176_insertion1107	CCCGATAGCCGTCTCCGAACTGGTGCGCGTGGTTCCTGCGAATGAAGAGGCCCGGGGAGGACAAGGTGCGCCCGGCCTGCCAGCACAAGCAGCGGCGCTGCAGCCTGGCCAGTGTGAGCGGGAGACCGGGGTCTCTGAGCGAGATGAGCGCCGTGGCGCCGCGCCCGCCAGCAACGGGAACCTGCTGTACATCGGCTTCCGCGGCCTGGACGGGAGACGACAGGCGTGT
nachra7_Frag1011-1176_insertion1110	CCCGATAGCCGTCTCCGAACTGGTGCGCGTGGTTCCTGCGAATGAAGAGGCCCGGGGAGGACAAGGTGCGCCCGGCCTGCCAGCACAAGCAGCGGCGCTGCAGCCTGGCCAGTGTGGAGAGCGGGAGACCGGGGTCTCTGAGCATGAGCGCCGTGGCGCCGCGCCCGCCAGCAACGGGAACCTGCTGTACATCGGCTTCCGCGGCCTGGACGGGAGACGACAGGCGTGT
nachra7_Frag1011-1176_insertion1113	CCCGATAGCCGTCTCCGAACTGGTGCGCGTGGTTCCTGCGAATGAAGAGGCCCGGGGAGGACAAGGTGCGCCCGGCCTGCCAGCACAAGCAGCGGCGCTGCAGCCTGGCCAGTGTGGAGATGAGCGGGAGACCGGGGTCTCTGAGCAGCGCCGTGGCGCCGCGCCCGCCAGCAACGGGAACCTGCTGTACATCGGCTTCCGCGGCCTGGACGGGAGACGACAGGCGTGT
nachra7_Frag1011-1176_insertion1116	CCCGATAGCCGTCTCCGAACTGGTGCGCGTGGTTCCTGCGAATGAAGAGGCCCGGGGAGGACAAGGTGCGCCCGGCCTGCCAGCACAAGCAGCGGCGCTGCAGCCTGGCCAGTGTGGAGATGAGCAGCGGGAGACCGGGGTCTCTGAGCGCCGTGGCGCCGCGCCCGCCAGCAACGGGAACCTGCTGTACATCGGCTTCCGCGGCCTGGACGGGAGACGACAGGCGTGT
nachra7_Frag1011-1176_insertion1119	CCCGATAGCCGTCTCCGAACTGGTGCGCGTGGTTCCTGCGAATGAAGAGGCCCGGGGAGGACAAGGTGCGCCCGGCCTGCCAGCACAAGCAGCGGCGCTGCAGCCTGGCCAGTGTGGAGATGAGCGCCAGCGGGAGACCGGGGTCTCTGAGCGTGGCGCCGCGCCCGCCAGCAACGGGAACCTGCTGTACATCGGCTTCCGCGGCCTGGACGGGAGACGACAGGCGTGT
nachra7_Frag1011-1176_insertion1122	CCCGATAGCCGTCTCCGAACTGGTGCGCGTGGTTCCTGCGAATGAAGAGGCCCGGGGAGGACAAGGTGCGCCCGGCCTGCCAGCACAAGCAGCGGCGCTGCAGCCTGGCCAGTGTGGAGATGAGCGCCGTGAGCGGGAGACCGGGGTCTCTGAGCGCGCCGCGCCCGCCAGCAACGGGAACCTGCTGTACATCGGCTTCCGCGGCCTGGACGGGAGACGACAGGCGTGT
nachra7_Frag1011-1176_insertion1125	CCCGATAGCCGTCTCCGAACTGGTGCGCGTGGTTCCTGCGAATGAAGAGGCCCGGGGAGGACAAGGTGCGCCCGGCCTGCCAGCACAAGCAGCGGCGCTGCAGCCTGGCCAGTGTGGAGATGAGCGCCGTGGCGAGCGGGAGACCGGGGTCTCTGAGCCCGCGCCCGCCAGCAACGGGAACCTGCTGTACATCGGCTTCCGCGGCCTGGACGGGAGACGACAGGCGTGT
nachra7_Frag1011-1176_insertion1128	CCCGATAGCCGTCTCCGAACTGGTGCGCGTGGTTCCTGCGAATGAAGAGGCCCGGGGAGGACAAGGTGCGCCCGGCCTGCCAGCACAAGCAGCGGCGCTGCAGCCTGGCCAGTGTGGAGATGAGCGCCGTGGCGCCGAGCGGGAGACCGGGGTCTCTGAGCCGCCCGCCAGCAACGGGAACCTGCTGTACATCGGCTTCCGCGGCCTGGACGGGAGACGACAGGCGTGT
nachra7_Frag1011-1176_insertion1131	CCCGATAGCCGTCTCCGAACTGGTGCGCGTGGTTCCTGCGAATGAAGAGGCCCGGGGAGGACAAGGTGCGCCCGGCCTGCCAGCACAAGCAGCGGCGCTGCAGCCTGGCCAGTGTGGAGATGAGCGCCGTGGCGCCGCGCAGCGGGAGACCGGGGTCTCTGAGCCCGCCAGCAACGGGAACCTGCTGTACATCGGCTTCCGCGGCCTGGACGGGAGACGACAGGCGTGT
nachra7_Frag1011-1176_insertion1134	CCCGATAGCCGTCTCCGAACTGGTGCGCGTGGTTCCTGCGAATGAAGAGGCCCGGGGAGGACAAGGTGCGCCCGGCCTGCCAGCACAAGCAGCGGCGCTGCAGCCTGGCCAGTGTGGAGATGAGCGCCGTGGCGCCGCGCCCGAGCGGGAGACCGGGGTCTCTGAGCCCAGCAACGGGAACCTGCTGTACATCGGCTTCCGCGGCCTGGACGGGAGACGACAGGCGTGT
nachra7_Frag1011-1176_insertion1137	CCCGATAGCCGTCTCCGAACTGGTGCGCGTGGTTCCTGCGAATGAAGAGGCCCGGGGAGGACAAGGTGCGCCCGGCCTGCCAGCACAAGCAGCGGCGCTGCAGCCTGGCCAGTGTGGAGATGAGCGCCGTGGCGCCGCGCCCGCCAAGCGGGAGACCGGGGTCTCTGAGCGCAACGGGAACCTGCTGTACATCGGCTTCCGCGGCCTGGACGGGAGACGACAGGCGTGT
nachra7_Frag1011-1176_insertion1140	CCCGATAGCCGTCTCCGAACTGGTGCGCGTGGTTCCTGCGAATGAAGAGGCCCGGGGAGGACAAGGTGCGCCCGGCCTGCCAGCACAAGCAGCGGCGCTGCAGCCTGGCCAGTGTGGAGATGAGCGCCGTGGCGCCGCGCCCGCCAGCAAGCGGGAGACCGGGGTCTCTGAGCACGGGAACCTGCTGTACATCGGCTTCCGCGGCCTGGACGGGAGACGACAGGCGTGT
nachra7_Frag1011-1176_insertion1143	CCCGATAGCCGTCTCCGAACTGGTGCGCGTGGTTCCTGCGAATGAAGAGGCCCGGGGAGGACAAGGTGCGCCCGGCCTGCCAGCACAAGCAGCGGCGCTGCAGCCTGGCCAGTGTGGAGATGAGCGCCGTGGCGCCGCGCCCGCCAGCAACGAGCGGGAGACCGGGGTCTCTGAGCGGAACCTGCTGTACATCGGCTTCCGCGGCCTGGACGGGAGACGACAGGCGTGT
nachra7_Frag1011-1176_insertion1146	CCCGATAGCCGTCTCCGAACTGGTGCGCGTGGTTCCTGCGAATGAAGAGGCCCGGGGAGGACAAGGTGCGCCCGGCCTGCCAGCACAAGCAGCGGCGCTGCAGCCTGGCCAGTGTGGAGATGAGCGCCGTGGCGCCGCGCCCGCCAGCAACGGGAAGCGGGAGACCGGGGTCTCTGAGCACCTGCTGTACATCGGCTTCCGCGGCCTGGACGGGAGACGACAGGCGTGT
nachra7_Frag1011-1176_insertion1149	CCCGATAGCCGTCTCCGAACTGGTGCGCGTGGTTCCTGCGAATGAAGAGGCCCGGGGAGGACAAGGTGCGCCCGGCCTGCCAGCACAAGCAGCGGCGCTGCAGCCTGGCCAGTGTGGAGATGAGCGCCGTGGCGCCGCGCCCGCCAGCAACGGGAACCAGCGGGAGACCGGGGTCTCTGAGCTGCTGTACATCGGCTTCCGCGGCCTGGACGGGAGACGACAGGCGTGT
nachra7_Frag1011-1176_insertion1152	CCCGATAGCCGTCTCCGAACTGGTGCGCGTGGTTCCTGCGAATGAAGAGGCCCGGGGAGGACAAGGTGCGCCCGGCCTGCCAGCACAAGCAGCGGCGCTGCAGCCTGGCCAGTGTGGAGATGAGCGCCGTGGCGCCGCGCCCGCCAGCAACGGGAACCTGCAGCGGGAGACCGGGGTCTCTGAGCTGTACATCGGCTTCCGCGGCCTGGACGGGAGACGACAGGCGTGT
nachra7_Frag1011-1176_insertion1155	CCCGATAGCCGTCTCCGAACTGGTGCGCGTGGTTCCTGCGAATGAAGAGGCCCGGGGAGGACAAGGTGCGCCCGGCCTGCCAGCACAAGCAGCGGCGCTGCAGCCTGGCCAGTGTGGAGATGAGCGCCGTGGCGCCGCGCCCGCCAGCAACGGGAACCTGCTGTAGCGGGAGACCGGGGTCTCTGAGCACATCGGCTTCCGCGGCCTGGACGGGAGACGACAGGCGTGT
nachra7_Frag1011-1176_insertion1158	CCCGATAGCCGTCTCCGAACTGGTGCGCGTGGTTCCTGCGAATGAAGAGGCCCGGGGAGGACAAGGTGCGCCCGGCCTGCCAGCACAAGCAGCGGCGCTGCAGCCTGGCCAGTGTGGAGATGAGCGCCGTGGCGCCGCGCCCGCCAGCAACGGGAACCTGCTGTACAAGCGGGAGACCGGGGTCTCTGAGCTCGGCTTCCGCGGCCTGGACGGGAGACGACAGGCGTGT
nachra7_Frag1011-1176_insertion1161	CCCGATAGCCGTCTCCGAACTGGTGCGCGTGGTTCCTGCGAATGAAGAGGCCCGGGGAGGACAAGGTGCGCCCGGCCTGCCAGCACAAGCAGCGGCGCTGCAGCCTGGCCAGTGTGGAGATGAGCGCCGTGGCGCCGCGCCCGCCAGCAACGGGAACCTGCTGTACATCGAGCGGGAGACCGGGGTCTCTGAGCGCTTCCGCGGCCTGGACGGGAGACGACAGGCGTGT
nachra7_Frag1011-1176_insertion1164	CCCGATAGCCGTCTCCGAACTGGTGCGCGTGGTTCCTGCGAATGAAGAGGCCCGGGGAGGACAAGGTGCGCCCGGCCTGCCAGCACAAGCAGCGGCGCTGCAGCCTGGCCAGTGTGGAGATGAGCGCCGTGGCGCCGCGCCCGCCAGCAACGGGAACCTGCTGTACATCGGCTAGCGGGAGACCGGGGTCTCTGAGCTCCGCGGCCTGGACGGGAGACGACAGGCGTGT
nachra7_Frag1011-1176_insertion1167	CCCGATAGCCGTCTCCGAACTGGTGCGCGTGGTTCCTGCGAATGAAGAGGCCCGGGGAGGACAAGGTGCGCCCGGCCTGCCAGCACAAGCAGCGGCGCTGCAGCCTGGCCAGTGTGGAGATGAGCGCCGTGGCGCCGCGCCCGCCAGCAACGGGAACCTGCTGTACATCGGCTTCCAGCGGGAGACCGGGGTCTCTGAGCGCGGCCTGGACGGGAGACGACAGGCGTGT
nachra7_Frag1011-1176_insertion1170	CCCGATAGCCGTCTCCGAACTGGTGCGCGTGGTTCCTGCGAATGAAGAGGCCCGGGGAGGACAAGGTGCGCCCGGCCTGCCAGCACAAGCAGCGGCGCTGCAGCCTGGCCAGTGTGGAGATGAGCGCCGTGGCGCCGCGCCCGCCAGCAACGGGAACCTGCTGTACATCGGCTTCCGCGAGCGGGAGACCGGGGTCTCTGAGCGCCTGGACGGGAGACGACAGGCGTGT
nachra7_Frag1011-1176_insertion1173	CCCGATAGCCGTCTCCGAACTGGTGCGCGTGGTTCCTGCGAATGAAGAGGCCCGGGGAGGACAAGGTGCGCCCGGCCTGCCAGCACAAGCAGCGGCGCTGCAGCCTGGCCAGTGTGGAGATGAGCGCCGTGGCGCCGCGCCCGCCAGCAACGGGAACCTGCTGTACATCGGCTTCCGCGGCCAGCGGGAGACCGGGGTCTCTGAGCTGGACGGGAGACGACAGGCGTGT
nachra7_Frag1011-1176_insertion1176	CCCGATAGCCGTCTCCGAACTGGTGCGCGTGGTTCCTGCGAATGAAGAGGCCCGGGGAGGACAAGGTGCGCCCGGCCTGCCAGCACAAGCAGCGGCGCTGCAGCCTGGCCAGTGTGGAGATGAGCGCCGTGGCGCCGCGCCCGCCAGCAACGGGAACCTGCTGTACATCGGCTTCCGCGGCCTGGAGCGGGAGACCGGGGTCTCTGAGCACGGGAGACGACAGGCGTGT
nachra7_Frag1179-1341_insertion1179	TTATAATCATCCGTCTCCGGACAGCGGGAGACCGGGGTCTCTGAGCGGCGTGCACTGTGTCCCGACCCCCGACTCTGGGGTAGTGTGTGGCCGCATGGCCTGCTCCCCCACGCACGATGAGCACCTCCTGCACGGCGGGCAACCCCCCGAGGGGGACCCGGACTTGGCCAAGATCCTGGAGGAGGTCCGCTACATTGCCAACCGCTTCCGCTGGAGACGCGAGCACACAT
nachra7_Frag1179-1341_insertion1182	TTATAATCATCCGTCTCCGGACGGCAGCGGGAGACCGGGGTCTCTGAGCGTGCACTGTGTCCCGACCCCCGACTCTGGGGTAGTGTGTGGCCGCATGGCCTGCTCCCCCACGCACGATGAGCACCTCCTGCACGGCGGGCAACCCCCCGAGGGGGACCCGGACTTGGCCAAGATCCTGGAGGAGGTCCGCTACATTGCCAACCGCTTCCGCTGGAGACGCGAGCACACAT
nachra7_Frag1179-1341_insertion1185	TTATAATCATCCGTCTCCGGACGGCGTGAGCGGGAGACCGGGGTCTCTGAGCCACTGTGTCCCGACCCCCGACTCTGGGGTAGTGTGTGGCCGCATGGCCTGCTCCCCCACGCACGATGAGCACCTCCTGCACGGCGGGCAACCCCCCGAGGGGGACCCGGACTTGGCCAAGATCCTGGAGGAGGTCCGCTACATTGCCAACCGCTTCCGCTGGAGACGCGAGCACACAT
nachra7_Frag1179-1341_insertion1188	TTATAATCATCCGTCTCCGGACGGCGTGCACAGCGGGAGACCGGGGTCTCTGAGCTGTGTCCCGACCCCCGACTCTGGGGTAGTGTGTGGCCGCATGGCCTGCTCCCCCACGCACGATGAGCACCTCCTGCACGGCGGGCAACCCCCCGAGGGGGACCCGGACTTGGCCAAGATCCTGGAGGAGGTCCGCTACATTGCCAACCGCTTCCGCTGGAGACGCGAGCACACAT
nachra7_Frag1179-1341_insertion1191	TTATAATCATCCGTCTCCGGACGGCGTGCACTGTAGCGGGAGACCGGGGTCTCTGAGCGTCCCGACCCCCGACTCTGGGGTAGTGTGTGGCCGCATGGCCTGCTCCCCCACGCACGATGAGCACCTCCTGCACGGCGGGCAACCCCCCGAGGGGGACCCGGACTTGGCCAAGATCCTGGAGGAGGTCCGCTACATTGCCAACCGCTTCCGCTGGAGACGCGAGCACACAT
nachra7_Frag1179-1341_insertion1194	TTATAATCATCCGTCTCCGGACGGCGTGCACTGTGTCAGCGGGAGACCGGGGTCTCTGAGCCCGACCCCCGACTCTGGGGTAGTGTGTGGCCGCATGGCCTGCTCCCCCACGCACGATGAGCACCTCCTGCACGGCGGGCAACCCCCCGAGGGGGACCCGGACTTGGCCAAGATCCTGGAGGAGGTCCGCTACATTGCCAACCGCTTCCGCTGGAGACGCGAGCACACAT
nachra7_Frag1179-1341_insertion1197	TTATAATCATCCGTCTCCGGACGGCGTGCACTGTGTCCCGAGCGGGAGACCGGGGTCTCTGAGCACCCCCGACTCTGGGGTAGTGTGTGGCCGCATGGCCTGCTCCCCCACGCACGATGAGCACCTCCTGCACGGCGGGCAACCCCCCGAGGGGGACCCGGACTTGGCCAAGATCCTGGAGGAGGTCCGCTACATTGCCAACCGCTTCCGCTGGAGACGCGAGCACACAT
nachra7_Frag1179-1341_insertion1200	TTATAATCATCCGTCTCCGGACGGCGTGCACTGTGTCCCGACCAGCGGGAGACCGGGGTCTCTGAGCCCCGACTCTGGGGTAGTGTGTGGCCGCATGGCCTGCTCCCCCACGCACGATGAGCACCTCCTGCACGGCGGGCAACCCCCCGAGGGGGACCCGGACTTGGCCAAGATCCTGGAGGAGGTCCGCTACATTGCCAACCGCTTCCGCTGGAGACGCGAGCACACAT
nachra7_Frag1179-1341_insertion1203	TTATAATCATCCGTCTCCGGACGGCGTGCACTGTGTCCCGACCCCCAGCGGGAGACCGGGGTCTCTGAGCGACTCTGGGGTAGTGTGTGGCCGCATGGCCTGCTCCCCCACGCACGATGAGCACCTCCTGCACGGCGGGCAACCCCCCGAGGGGGACCCGGACTTGGCCAAGATCCTGGAGGAGGTCCGCTACATTGCCAACCGCTTCCGCTGGAGACGCGAGCACACAT
nachra7_Frag1179-1341_insertion1206	TTATAATCATCCGTCTCCGGACGGCGTGCACTGTGTCCCGACCCCCGACAGCGGGAGACCGGGGTCTCTGAGCTCTGGGGTAGTGTGTGGCCGCATGGCCTGCTCCCCCACGCACGATGAGCACCTCCTGCACGGCGGGCAACCCCCCGAGGGGGACCCGGACTTGGCCAAGATCCTGGAGGAGGTCCGCTACATTGCCAACCGCTTCCGCTGGAGACGCGAGCACACAT
nachra7_Frag1179-1341_insertion1209	TTATAATCATCCGTCTCCGGACGGCGTGCACTGTGTCCCGACCCCCGACTCTAGCGGGAGACCGGGGTCTCTGAGCGGGGTAGTGTGTGGCCGCATGGCCTGCTCCCCCACGCACGATGAGCACCTCCTGCACGGCGGGCAACCCCCCGAGGGGGACCCGGACTTGGCCAAGATCCTGGAGGAGGTCCGCTACATTGCCAACCGCTTCCGCTGGAGACGCGAGCACACAT
nachra7_Frag1179-1341_insertion1212	TTATAATCATCCGTCTCCGGACGGCGTGCACTGTGTCCCGACCCCCGACTCTGGGAGCGGGAGACCGGGGTCTCTGAGCGTAGTGTGTGGCCGCATGGCCTGCTCCCCCACGCACGATGAGCACCTCCTGCACGGCGGGCAACCCCCCGAGGGGGACCCGGACTTGGCCAAGATCCTGGAGGAGGTCCGCTACATTGCCAACCGCTTCCGCTGGAGACGCGAGCACACAT
nachra7_Frag1179-1341_insertion1215	TTATAATCATCCGTCTCCGGACGGCGTGCACTGTGTCCCGACCCCCGACTCTGGGGTAAGCGGGAGACCGGGGTCTCTGAGCGTGTGTGGCCGCATGGCCTGCTCCCCCACGCACGATGAGCACCTCCTGCACGGCGGGCAACCCCCCGAGGGGGACCCGGACTTGGCCAAGATCCTGGAGGAGGTCCGCTACATTGCCAACCGCTTCCGCTGGAGACGCGAGCACACAT
nachra7_Frag1179-1341_insertion1218	TTATAATCATCCGTCTCCGGACGGCGTGCACTGTGTCCCGACCCCCGACTCTGGGGTAGTGAGCGGGAGACCGGGGTCTCTGAGCTGTGGCCGCATGGCCTGCTCCCCCACGCACGATGAGCACCTCCTGCACGGCGGGCAACCCCCCGAGGGGGACCCGGACTTGGCCAAGATCCTGGAGGAGGTCCGCTACATTGCCAACCGCTTCCGCTGGAGACGCGAGCACACAT
nachra7_Frag1179-1341_insertion1221	TTATAATCATCCGTCTCCGGACGGCGTGCACTGTGTCCCGACCCCCGACTCTGGGGTAGTGTGTAGCGGGAGACCGGGGTCTCTGAGCGGCCGCATGGCCTGCTCCCCCACGCACGATGAGCACCTCCTGCACGGCGGGCAACCCCCCGAGGGGGACCCGGACTTGGCCAAGATCCTGGAGGAGGTCCGCTACATTGCCAACCGCTTCCGCTGGAGACGCGAGCACACAT
nachra7_Frag1179-1341_insertion1224	TTATAATCATCCGTCTCCGGACGGCGTGCACTGTGTCCCGACCCCCGACTCTGGGGTAGTGTGTGGCAGCGGGAGACCGGGGTCTCTGAGCCGCATGGCCTGCTCCCCCACGCACGATGAGCACCTCCTGCACGGCGGGCAACCCCCCGAGGGGGACCCGGACTTGGCCAAGATCCTGGAGGAGGTCCGCTACATTGCCAACCGCTTCCGCTGGAGACGCGAGCACACAT
nachra7_Frag1179-1341_insertion1227	TTATAATCATCCGTCTCCGGACGGCGTGCACTGTGTCCCGACCCCCGACTCTGGGGTAGTGTGTGGCCGCAGCGGGAGACCGGGGTCTCTGAGCATGGCCTGCTCCCCCACGCACGATGAGCACCTCCTGCACGGCGGGCAACCCCCCGAGGGGGACCCGGACTTGGCCAAGATCCTGGAGGAGGTCCGCTACATTGCCAACCGCTTCCGCTGGAGACGCGAGCACACAT
nachra7_Frag1179-1341_insertion1230	TTATAATCATCCGTCTCCGGACGGCGTGCACTGTGTCCCGACCCCCGACTCTGGGGTAGTGTGTGGCCGCATGAGCGGGAGACCGGGGTCTCTGAGCGCCTGCTCCCCCACGCACGATGAGCACCTCCTGCACGGCGGGCAACCCCCCGAGGGGGACCCGGACTTGGCCAAGATCCTGGAGGAGGTCCGCTACATTGCCAACCGCTTCCGCTGGAGACGCGAGCACACAT
nachra7_Frag1179-1341_insertion1233	TTATAATCATCCGTCTCCGGACGGCGTGCACTGTGTCCCGACCCCCGACTCTGGGGTAGTGTGTGGCCGCATGGCCAGCGGGAGACCGGGGTCTCTGAGCTGCTCCCCCACGCACGATGAGCACCTCCTGCACGGCGGGCAACCCCCCGAGGGGGACCCGGACTTGGCCAAGATCCTGGAGGAGGTCCGCTACATTGCCAACCGCTTCCGCTGGAGACGCGAGCACACAT
nachra7_Frag1179-1341_insertion1236	TTATAATCATCCGTCTCCGGACGGCGTGCACTGTGTCCCGACCCCCGACTCTGGGGTAGTGTGTGGCCGCATGGCCTGCAGCGGGAGACCGGGGTCTCTGAGCTCCCCCACGCACGATGAGCACCTCCTGCACGGCGGGCAACCCCCCGAGGGGGACCCGGACTTGGCCAAGATCCTGGAGGAGGTCCGCTACATTGCCAACCGCTTCCGCTGGAGACGCGAGCACACAT
nachra7_Frag1179-1341_insertion1239	TTATAATCATCCGTCTCCGGACGGCGTGCACTGTGTCCCGACCCCCGACTCTGGGGTAGTGTGTGGCCGCATGGCCTGCTCCAGCGGGAGACCGGGGTCTCTGAGCCCCACGCACGATGAGCACCTCCTGCACGGCGGGCAACCCCCCGAGGGGGACCCGGACTTGGCCAAGATCCTGGAGGAGGTCCGCTACATTGCCAACCGCTTCCGCTGGAGACGCGAGCACACAT
nachra7_Frag1179-1341_insertion1242	TTATAATCATCCGTCTCCGGACGGCGTGCACTGTGTCCCGACCCCCGACTCTGGGGTAGTGTGTGGCCGCATGGCCTGCTCCCCCAGCGGGAGACCGGGGTCTCTGAGCACGCACGATGAGCACCTCCTGCACGGCGGGCAACCCCCCGAGGGGGACCCGGACTTGGCCAAGATCCTGGAGGAGGTCCGCTACATTGCCAACCGCTTCCGCTGGAGACGCGAGCACACAT
nachra7_Frag1179-1341_insertion1245	TTATAATCATCCGTCTCCGGACGGCGTGCACTGTGTCCCGACCCCCGACTCTGGGGTAGTGTGTGGCCGCATGGCCTGCTCCCCCACGAGCGGGAGACCGGGGTCTCTGAGCCACGATGAGCACCTCCTGCACGGCGGGCAACCCCCCGAGGGGGACCCGGACTTGGCCAAGATCCTGGAGGAGGTCCGCTACATTGCCAACCGCTTCCGCTGGAGACGCGAGCACACAT
nachra7_Frag1179-1341_insertion1248	TTATAATCATCCGTCTCCGGACGGCGTGCACTGTGTCCCGACCCCCGACTCTGGGGTAGTGTGTGGCCGCATGGCCTGCTCCCCCACGCACAGCGGGAGACCGGGGTCTCTGAGCGATGAGCACCTCCTGCACGGCGGGCAACCCCCCGAGGGGGACCCGGACTTGGCCAAGATCCTGGAGGAGGTCCGCTACATTGCCAACCGCTTCCGCTGGAGACGCGAGCACACAT
nachra7_Frag1179-1341_insertion1251	TTATAATCATCCGTCTCCGGACGGCGTGCACTGTGTCCCGACCCCCGACTCTGGGGTAGTGTGTGGCCGCATGGCCTGCTCCCCCACGCACGATAGCGGGAGACCGGGGTCTCTGAGCGAGCACCTCCTGCACGGCGGGCAACCCCCCGAGGGGGACCCGGACTTGGCCAAGATCCTGGAGGAGGTCCGCTACATTGCCAACCGCTTCCGCTGGAGACGCGAGCACACAT
nachra7_Frag1179-1341_insertion1254	TTATAATCATCCGTCTCCGGACGGCGTGCACTGTGTCCCGACCCCCGACTCTGGGGTAGTGTGTGGCCGCATGGCCTGCTCCCCCACGCACGATGAGAGCGGGAGACCGGGGTCTCTGAGCCACCTCCTGCACGGCGGGCAACCCCCCGAGGGGGACCCGGACTTGGCCAAGATCCTGGAGGAGGTCCGCTACATTGCCAACCGCTTCCGCTGGAGACGCGAGCACACAT
nachra7_Frag1179-1341_insertion1257	TTATAATCATCCGTCTCCGGACGGCGTGCACTGTGTCCCGACCCCCGACTCTGGGGTAGTGTGTGGCCGCATGGCCTGCTCCCCCACGCACGATGAGCACAGCGGGAGACCGGGGTCTCTGAGCCTCCTGCACGGCGGGCAACCCCCCGAGGGGGACCCGGACTTGGCCAAGATCCTGGAGGAGGTCCGCTACATTGCCAACCGCTTCCGCTGGAGACGCGAGCACACAT
nachra7_Frag1179-1341_insertion1260	TTATAATCATCCGTCTCCGGACGGCGTGCACTGTGTCCCGACCCCCGACTCTGGGGTAGTGTGTGGCCGCATGGCCTGCTCCCCCACGCACGATGAGCACCTCAGCGGGAGACCGGGGTCTCTGAGCCTGCACGGCGGGCAACCCCCCGAGGGGGACCCGGACTTGGCCAAGATCCTGGAGGAGGTCCGCTACATTGCCAACCGCTTCCGCTGGAGACGCGAGCACACAT
nachra7_Frag1179-1341_insertion1263	TTATAATCATCCGTCTCCGGACGGCGTGCACTGTGTCCCGACCCCCGACTCTGGGGTAGTGTGTGGCCGCATGGCCTGCTCCCCCACGCACGATGAGCACCTCCTGAGCGGGAGACCGGGGTCTCTGAGCCACGGCGGGCAACCCCCCGAGGGGGACCCGGACTTGGCCAAGATCCTGGAGGAGGTCCGCTACATTGCCAACCGCTTCCGCTGGAGACGCGAGCACACAT
nachra7_Frag1179-1341_insertion1266	TTATAATCATCCGTCTCCGGACGGCGTGCACTGTGTCCCGACCCCCGACTCTGGGGTAGTGTGTGGCCGCATGGCCTGCTCCCCCACGCACGATGAGCACCTCCTGCACAGCGGGAGACCGGGGTCTCTGAGCGGCGGGCAACCCCCCGAGGGGGACCCGGACTTGGCCAAGATCCTGGAGGAGGTCCGCTACATTGCCAACCGCTTCCGCTGGAGACGCGAGCACACAT
nachra7_Frag1179-1341_insertion1269	TTATAATCATCCGTCTCCGGACGGCGTGCACTGTGTCCCGACCCCCGACTCTGGGGTAGTGTGTGGCCGCATGGCCTGCTCCCCCACGCACGATGAGCACCTCCTGCACGGCAGCGGGAGACCGGGGTCTCTGAGCGGGCAACCCCCCGAGGGGGACCCGGACTTGGCCAAGATCCTGGAGGAGGTCCGCTACATTGCCAACCGCTTCCGCTGGAGACGCGAGCACACAT
nachra7_Frag1179-1341_insertion1272	TTATAATCATCCGTCTCCGGACGGCGTGCACTGTGTCCCGACCCCCGACTCTGGGGTAGTGTGTGGCCGCATGGCCTGCTCCCCCACGCACGATGAGCACCTCCTGCACGGCGGGAGCGGGAGACCGGGGTCTCTGAGCCAACCCCCCGAGGGGGACCCGGACTTGGCCAAGATCCTGGAGGAGGTCCGCTACATTGCCAACCGCTTCCGCTGGAGACGCGAGCACACAT
nachra7_Frag1179-1341_insertion1275	TTATAATCATCCGTCTCCGGACGGCGTGCACTGTGTCCCGACCCCCGACTCTGGGGTAGTGTGTGGCCGCATGGCCTGCTCCCCCACGCACGATGAGCACCTCCTGCACGGCGGGCAAAGCGGGAGACCGGGGTCTCTGAGCCCCCCCGAGGGGGACCCGGACTTGGCCAAGATCCTGGAGGAGGTCCGCTACATTGCCAACCGCTTCCGCTGGAGACGCGAGCACACAT
nachra7_Frag1179-1341_insertion1278	TTATAATCATCCGTCTCCGGACGGCGTGCACTGTGTCCCGACCCCCGACTCTGGGGTAGTGTGTGGCCGCATGGCCTGCTCCCCCACGCACGATGAGCACCTCCTGCACGGCGGGCAACCCAGCGGGAGACCGGGGTCTCTGAGCCCCGAGGGGGACCCGGACTTGGCCAAGATCCTGGAGGAGGTCCGCTACATTGCCAACCGCTTCCGCTGGAGACGCGAGCACACAT
nachra7_Frag1179-1341_insertion1281	TTATAATCATCCGTCTCCGGACGGCGTGCACTGTGTCCCGACCCCCGACTCTGGGGTAGTGTGTGGCCGCATGGCCTGCTCCCCCACGCACGATGAGCACCTCCTGCACGGCGGGCAACCCCCCAGCGGGAGACCGGGGTCTCTGAGCGAGGGGGACCCGGACTTGGCCAAGATCCTGGAGGAGGTCCGCTACATTGCCAACCGCTTCCGCTGGAGACGCGAGCACACAT
nachra7_Frag1179-1341_insertion1284	TTATAATCATCCGTCTCCGGACGGCGTGCACTGTGTCCCGACCCCCGACTCTGGGGTAGTGTGTGGCCGCATGGCCTGCTCCCCCACGCACGATGAGCACCTCCTGCACGGCGGGCAACCCCCCGAGAGCGGGAGACCGGGGTCTCTGAGCGGGGACCCGGACTTGGCCAAGATCCTGGAGGAGGTCCGCTACATTGCCAACCGCTTCCGCTGGAGACGCGAGCACACAT
nachra7_Frag1179-1341_insertion1287	TTATAATCATCCGTCTCCGGACGGCGTGCACTGTGTCCCGACCCCCGACTCTGGGGTAGTGTGTGGCCGCATGGCCTGCTCCCCCACGCACGATGAGCACCTCCTGCACGGCGGGCAACCCCCCGAGGGGAGCGGGAGACCGGGGTCTCTGAGCGACCCGGACTTGGCCAAGATCCTGGAGGAGGTCCGCTACATTGCCAACCGCTTCCGCTGGAGACGCGAGCACACAT
nachra7_Frag1179-1341_insertion1290	TTATAATCATCCGTCTCCGGACGGCGTGCACTGTGTCCCGACCCCCGACTCTGGGGTAGTGTGTGGCCGCATGGCCTGCTCCCCCACGCACGATGAGCACCTCCTGCACGGCGGGCAACCCCCCGAGGGGGACAGCGGGAGACCGGGGTCTCTGAGCCCGGACTTGGCCAAGATCCTGGAGGAGGTCCGCTACATTGCCAACCGCTTCCGCTGGAGACGCGAGCACACAT

nachra7_Frag1179-1341_insertion1293 TTATAATCATCCGTCTCCGGACGGCGTGCACTGTGTCCCGACCCCCGACTCTGGGGTAGTGTGTGGCCGCATGGCCTGCTCCCCACGCACGATGAGCACCTCCTGCACGGCGGGCAACCCCCGAGGGGGACCCGAGCGGGAGACCGGGGTCTCTGAGCGACTTGGCCAAGATCCTGGAGGAGGTCCGCTACATTGCCAACCGCTTCCGCTGGAGACGCGAGCACACAT
nachra7_Frag1179-1341_insertion1296 TTATAATCATCCGTCTCCGGACGGCGTGCACTGTGTCCCGACCCCCGACTCTGGGGTAGTGTGTGGCCGCATGGCCTGCTCCCCACGCACGATGAGCACCTCCTGCACGGCGGGCAACCCCCGAGGGGGACCCGGACAGCGGGAGACCGGGGTCTCTGAGCTTGGCCAAGATCCTGGAGGAGGTCCGCTACATTGCCAACCGCTTCCGCTGGAGACGCGAGCACACAT
nachra7_Frag1179-1341_insertion1299 TTATAATCATCCGTCTCCGGACGGCGTGCACTGTGTCCCGACCCCCGACTCTGGGGTAGTGTGTGGCCGCATGGCCTGCTCCCCACGCACGATGAGCACCTCCTGCACGGCGGGCAACCCCCGAGGGGGACCCGGACTTGAGCGGGAGACCGGGGTCTCTGAGCGCCAAGATCCTGGAGGAGGTCCGCTACATTGCCAACCGCTTCCGCTGGAGACGCGAGCACACAT
nachra7_Frag1179-1341_insertion1302 TTATAATCATCCGTCTCCGGACGGCGTGCACTGTGTCCCGACCCCCGACTCTGGGGTAGTGTGTGGCCGCATGGCCTGCTCCCCACGCACGATGAGCACCTCCTGCACGGCGGGCAACCCCCGAGGGGGACCCGGACTTGGCCAGCGGGAGACCGGGGTCTCTGAGCAAGATCCTGGAGGAGGTCCGCTACATTGCCAACCGCTTCCGCTGGAGACGCGAGCACACAT
nachra7_Frag1179-1341_insertion1305 TTATAATCATCCGTCTCCGGACGGCGTGCACTGTGTCCCGACCCCCGACTCTGGGGTAGTGTGTGGCCGCATGGCCTGCTCCCCACGCACGATGAGCACCTCCTGCACGGCGGGCAACCCCCGAGGGGGACCCGGACTTGGCCAAGAGCGGGAGACCGGGGTCTCTGAGCATCCTGGAGGAGGTCCGCTACATTGCCAACCGCTTCCGCTGGAGACGCGAGCACACAT
nachra7_Frag1179-1341_insertion1308 TTATAATCATCCGTCTCCGGACGGCGTGCACTGTGTCCCGACCCCCGACTCTGGGGTAGTGTGTGGCCGCATGGCCTGCTCCCCACGCACGATGAGCACCTCCTGCACGGCGGGCAACCCCCGAGGGGGACCCGGACTTGGCCAAGATCAGCGGGAGACCGGGGTCTCTGAGCCTGGAGGAGGTCCGCTACATTGCCAACCGCTTCCGCTGGAGACGCGAGCACACAT
nachra7_Frag1179-1341_insertion1311 TTATAATCATCCGTCTCCGGACGGCGTGCACTGTGTCCCGACCCCCGACTCTGGGGTAGTGTGTGGCCGCATGGCCTGCTCCCCACGCACGATGAGCACCTCCTGCACGGCGGGCAACCCCCGAGGGGGACCCGGACTTGGCCAAGATCCTGAGCGGGAGACCGGGGTCTCTGAGCGAGGAGGTCCGCTACATTGCCAACCGCTTCCGCTGGAGACGCGAGCACACAT
nachra7_Frag1179-1341_insertion1314 TTATAATCATCCGTCTCCGGACGGCGTGCACTGTGTCCCGACCCCCGACTCTGGGGTAGTGTGTGGCCGCATGGCCTGCTCCCCACGCACGATGAGCACCTCCTGCACGGCGGGCAACCCCCGAGGGGGACCCGGACTTGGCCAAGATCCTGGAGAGCGGGAGACCGGGGTCTCTGAGCGAGGTCCGCTACATTGCCAACCGCTTCCGCTGGAGACGCGAGCACACAT
nachra7_Frag1179-1341_insertion1317 TTATAATCATCCGTCTCCGGACGGCGTGCACTGTGTCCCGACCCCCGACTCTGGGGTAGTGTGTGGCCGCATGGCCTGCTCCCCACGCACGATGAGCACCTCCTGCACGGCGGGCAACCCCCGAGGGGGACCCGGACTTGGCCAAGATCCTGGAGGAGAGCGGGAGACCGGGGTCTCTGAGCGTCCGCTACATTGCCAACCGCTTCCGCTGGAGACGCGAGCACACAT
nachra7_Frag1179-1341_insertion1320 TTATAATCATCCGTCTCCGGACGGCGTGCACTGTGTCCCGACCCCCGACTCTGGGGTAGTGTGTGGCCGCATGGCCTGCTCCCCACGCACGATGAGCACCTCCTGCACGGCGGGCAACCCCCGAGGGGGACCCGGACTTGGCCAAGATCCTGGAGGAGGTCAGCGGGAGACCGGGGTCTCTGAGCCGCTACATTGCCAACCGCTTCCGCTGGAGACGCGAGCACACAT
nachra7_Frag1179-1341_insertion1323 TTATAATCATCCGTCTCCGGACGGCGTGCACTGTGTCCCGACCCCCGACTCTGGGGTAGTGTGTGGCCGCATGGCCTGCTCCCCACGCACGATGAGCACCTCCTGCACGGCGGGCAACCCCCGAGGGGGACCCGGACTTGGCCAAGATCCTGGAGGAGGTCCGCAGCGGGAGACCGGGGTCTCTGAGCTACATTGCCAACCGCTTCCGCTGGAGACGCGAGCACACAT
nachra7_Frag1179-1341_insertion1326 TTATAATCATCCGTCTCCGGACGGCGTGCACTGTGTCCCGACCCCCGACTCTGGGGTAGTGTGTGGCCGCATGGCCTGCTCCCCACGCACGATGAGCACCTCCTGCACGGCGGGCAACCCCCGAGGGGGACCCGGACTTGGCCAAGATCCTGGAGGAGGTCCGCTACAGCGGGAGACCGGGGTCTCTGAGCATTGCCAACCGCTTCCGCTGGAGACGCGAGCACACAT
nachra7_Frag1179-1341_insertion1329 TTATAATCATCCGTCTCCGGACGGCGTGCACTGTGTCCCGACCCCCGACTCTGGGGTAGTGTGTGGCCGCATGGCCTGCTCCCCACGCACGATGAGCACCTCCTGCACGGCGGGCAACCCCCGAGGGGGACCCGGACTTGGCCAAGATCCTGGAGGAGGTCCGCTACATTAGCGGGAGACCGGGGTCTCTGAGCGCCAACCGCTTCCGCTGGAGACGCGAGCACACAT
nachra7_Frag1179-1341_insertion1332 TTATAATCATCCGTCTCCGGACGGCGTGCACTGTGTCCCGACCCCCGACTCTGGGGTAGTGTGTGGCCGCATGGCCTGCTCCCCACGCACGATGAGCACCTCCTGCACGGCGGGCAACCCCCGAGGGGGACCCGGACTTGGCCAAGATCCTGGAGGAGGTCCGCTACATTGCCAGCGGGAGACCGGGGTCTCTGAGCAACCGCTTCCGCTGGAGACGCGAGCACACAT
nachra7_Frag1179-1341_insertion1335 TTATAATCATCCGTCTCCGGACGGCGTGCACTGTGTCCCGACCCCCGACTCTGGGGTAGTGTGTGGCCGCATGGCCTGCTCCCCACGCACGATGAGCACCTCCTGCACGGCGGGCAACCCCCGAGGGGGACCCGGACTTGGCCAAGATCCTGGAGGAGGTCCGCTACATTGCCAACAGCGGGAGACCGGGGTCTCTGAGCCGCTTCCGCTGGAGACGCGAGCACACAT
nachra7_Frag1179-1341_insertion1338 TTATAATCATCCGTCTCCGGACGGCGTGCACTGTGTCCCGACCCCCGACTCTGGGGTAGTGTGTGGCCGCATGGCCTGCTCCCCACGCACGATGAGCACCTCCTGCACGGCGGGCAACCCCCGAGGGGGACCCGGACTTGGCCAAGATCCTGGAGGAGGTCCGCTACATTGCCAACCGCAGCGGGAGACCGGGGTCTCTGAGCTTCCGCTGGAGACGCGAGCACACAT
nachra7_Frag1179-1341_insertion1341 TTATAATCATCCGTCTCCGGACGGCGTGCACTGTGTCCCGACCCCCGACTCTGGGGTAGTGTGTGGCCGCATGGCCTGCTCCCCACGCACGATGAGCACCTCCTGCACGGCGGGCAACCCCCGAGGGGGACCCGGACTTGGCCAAGATCCTGGAGGAGGTCCGCTACATTGCCAACCGCTTCAGCGGGAGACCGGGGTCTCTGAGCCGCTGGAGACGCGAGCACACAT
nachra7_Frag1344-1506_insertion1344 TAGATTCCCTTCGTCTCCCCGCAGCGGGAGACCGGGGTCTCTGAGCTGCCAGGACGAAAGCGAGGCGGTCTGCAGCGAGTGGAAGTTCGCCGCCTGTGTGGTGGACCGCCTGTGCCTCATGGCCTTCTCGGTCTTCACCATCATCTGCACCATCGGCATCCTGATGTCGGCTCCCAACTTCGTGGAGGCCGTGTCCAAAGACTTTGCGGCTGGGAGACGCCGACAAAAGT
nachra7_Frag1344-1506_insertion1347 TAGATTCCCTTCGTCTCCCCGCTGCAGCGGGAGACCGGGGTCTCTGAGCCAGGACGAAAGCGAGGCGGTCTGCAGCGAGTGGAAGTTCGCCGCCTGTGTGGTGGACCGCCTGTGCCTCATGGCCTTCTCGGTCTTCACCATCATCTGCACCATCGGCATCCTGATGTCGGCTCCCAACTTCGTGGAGGCCGTGTCCAAAGACTTTGCGGCTGGGAGACGCCGACAAAAGT
nachra7_Frag1344-1506_insertion1350 TAGATTCCCTTCGTCTCCCCGCTGCCAGAGCGGGAGACCGGGGTCTCTGAGCGACGAAAGCGAGGCGGTCTGCAGCGAGTGGAAGTTCGCCGCCTGTGTGGTGGACCGCCTGTGCCTCATGGCCTTCTCGGTCTTCACCATCATCTGCACCATCGGCATCCTGATGTCGGCTCCCAACTTCGTGGAGGCCGTGTCCAAAGACTTTGCGGCTGGGAGACGCCGACAAAAGT
nachra7_Frag1344-1506_insertion1353 TAGATTCCCTTCGTCTCCCCGCTGCCAGGACAGCGGGAGACCGGGGTCTCTGAGCGAAAGCGAGGCGGTCTGCAGCGAGTGGAAGTTCGCCGCCTGTGTGGTGGACCGCCTGTGCCTCATGGCCTTCTCGGTCTTCACCATCATCTGCACCATCGGCATCCTGATGTCGGCTCCCAACTTCGTGGAGGCCGTGTCCAAAGACTTTGCGGCTGGGAGACGCCGACAAAAGT
nachra7_Frag1344-1506_insertion1356 TAGATTCCCTTCGTCTCCCCGCTGCCAGGACGAAAGCGGGAGACCGGGGTCTCTGAGCAGCGAGGCGGTCTGCAGCGAGTGGAAGTTCGCCGCCTGTGTGGTGGACCGCCTGTGCCTCATGGCCTTCTCGGTCTTCACCATCATCTGCACCATCGGCATCCTGATGTCGGCTCCCAACTTCGTGGAGGCCGTGTCCAAAGACTTTGCGGCTGGGAGACGCCGACAAAAGT
nachra7_Frag1344-1506_insertion1359 TAGATTCCCTTCGTCTCCCCGCTGCCAGGACGAAAGCAGCGGGAGACCGGGGTCTCTGAGCGAGGCGGTCTGCAGCGAGTGGAAGTTCGCCGCCTGTGTGGTGGACCGCCTGTGCCTCATGGCCTTCTCGGTCTTCACCATCATCTGCACCATCGGCATCCTGATGTCGGCTCCCAACTTCGTGGAGGCCGTGTCCAAAGACTTTGCGGCTGGGAGACGCCGACAAAAGT
nachra7_Frag1344-1506_insertion1362 TAGATTCCCTTCGTCTCCCCGCTGCCAGGACGAAAGCGAGAGCGGGAGACCGGGGTCTCTGAGCGCGGTCTGCAGCGAGTGGAAGTTCGCCGCCTGTGTGGTGGACCGCCTGTGCCTCATGGCCTTCTCGGTCTTCACCATCATCTGCACCATCGGCATCCTGATGTCGGCTCCCAACTTCGTGGAGGCCGTGTCCAAAGACTTTGCGGCTGGGAGACGCCGACAAAAGT
nachra7_Frag1344-1506_insertion1365 TAGATTCCCTTCGTCTCCCCGCTGCCAGGACGAAAGCGAGGCGAGCGGGAGACCGGGGTCTCTGAGCGTCTGCAGCGAGTGGAAGTTCGCCGCCTGTGTGGTGGACCGCCTGTGCCTCATGGCCTTCTCGGTCTTCACCATCATCTGCACCATCGGCATCCTGATGTCGGCTCCCAACTTCGTGGAGGCCGTGTCCAAAGACTTTGCGGCTGGGAGACGCCGACAAAAGT
nachra7_Frag1344-1506_insertion1368 TAGATTCCCTTCGTCTCCCCGCTGCCAGGACGAAAGCGAGGCGGTCAGCGGGAGACCGGGGTCTCTGAGCTGCAGCGAGTGGAAGTTCGCCGCCTGTGTGGTGGACCGCCTGTGCCTCATGGCCTTCTCGGTCTTCACCATCATCTGCACCATCGGCATCCTGATGTCGGCTCCCAACTTCGTGGAGGCCGTGTCCAAAGACTTTGCGGCTGGGAGACGCCGACAAAAGT
nachra7_Frag1344-1506_insertion1371 TAGATTCCCTTCGTCTCCCCGCTGCCAGGACGAAAGCGAGGCGGTCTGCAGCGGGAGACCGGGGTCTCTGAGCAGCGAGTGGAAGTTCGCCGCCTGTGTGGTGGACCGCCTGTGCCTCATGGCCTTCTCGGTCTTCACCATCATCTGCACCATCGGCATCCTGATGTCGGCTCCCAACTTCGTGGAGGCCGTGTCCAAAGACTTTGCGGCTGGGAGACGCCGACAAAAGT
nachra7_Frag1344-1506_insertion1374 TAGATTCCCTTCGTCTCCCCGCTGCCAGGACGAAAGCGAGGCGGTCTGCAGCAGCGGGAGACCGGGGTCTCTGAGCGAGTGGAAGTTCGCCGCCTGTGTGGTGGACCGCCTGTGCCTCATGGCCTTCTCGGTCTTCACCATCATCTGCACCATCGGCATCCTGATGTCGGCTCCCAACTTCGTGGAGGCCGTGTCCAAAGACTTTGCGGCTGGGAGACGCCGACAAAAGT
nachra7_Frag1344-1506_insertion1377 TAGATTCCCTTCGTCTCCCCGCTGCCAGGACGAAAGCGAGGCGGTCTGCAGCGAGAGCGGGAGACCGGGGTCTCTGAGCTGGAAGTTCGCCGCCTGTGTGGTGGACCGCCTGTGCCTCATGGCCTTCTCGGTCTTCACCATCATCTGCACCATCGGCATCCTGATGTCGGCTCCCAACTTCGTGGAGGCCGTGTCCAAAGACTTTGCGGCTGGGAGACGCCGACAAAAGT
nachra7_Frag1344-1506_insertion1380 TAGATTCCCTTCGTCTCCCCGCTGCCAGGACGAAAGCGAGGCGGTCTGCAGCGAGTGGAGCGGGAGACCGGGGTCTCTGAGCAAGTTCGCCGCCTGTGTGGTGGACCGCCTGTGCCTCATGGCCTTCTCGGTCTTCACCATCATCTGCACCATCGGCATCCTGATGTCGGCTCCCAACTTCGTGGAGGCCGTGTCCAAAGACTTTGCGGCTGGGAGACGCCGACAAAAGT
nachra7_Frag1344-1506_insertion1383 TAGATTCCCTTCGTCTCCCCGCTGCCAGGACGAAAGCGAGGCGGTCTGCAGCGAGTGGAAGAGCGGGAGACCGGGGTCTCTGAGCTTCGCCGCCTGTGTGGTGGACCGCCTGTGCCTCATGGCCTTCTCGGTCTTCACCATCATCTGCACCATCGGCATCCTGATGTCGGCTCCCAACTTCGTGGAGGCCGTGTCCAAAGACTTTGCGGCTGGGAGACGCCGACAAAAGT
nachra7_Frag1344-1506_insertion1386 TAGATTCCCTTCGTCTCCCCGCTGCCAGGACGAAAGCGAGGCGGTCTGCAGCGAGTGGAAGTTCAGCGGGAGACCGGGGTCTCTGAGCGCCGCCTGTGTGGTGGACCGCCTGTGCCTCATGGCCTTCTCGGTCTTCACCATCATCTGCACCATCGGCATCCTGATGTCGGCTCCCAACTTCGTGGAGGCCGTGTCCAAAGACTTTGCGGCTGGGAGACGCCGACAAAAGT
nachra7_Frag1344-1506_insertion1389 TAGATTCCCTTCGTCTCCCCGCTGCCAGGACGAAAGCGAGGCGGTCTGCAGCGAGTGGAAGTTCGCCAGCGGGAGACCGGGGTCTCTGAGCGCCTGTGTGGTGGACCGCCTGTGCCTCATGGCCTTCTCGGTCTTCACCATCATCTGCACCATCGGCATCCTGATGTCGGCTCCCAACTTCGTGGAGGCCGTGTCCAAAGACTTTGCGGCTGGGAGACGCCGACAAAAGT
nachra7_Frag1344-1506_insertion1392 TAGATTCCCTTCGTCTCCCCGCTGCCAGGACGAAAGCGAGGCGGTCTGCAGCGAGTGGAAGTTCGCCGCCAGCGGGAGACCGGGGTCTCTGAGCTGTGTGGTGGACCGCCTGTGCCTCATGGCCTTCTCGGTCTTCACCATCATCTGCACCATCGGCATCCTGATGTCGGCTCCCAACTTCGTGGAGGCCGTGTCCAAAGACTTTGCGGCTGGGAGACGCCGACAAAAGT
nachra7_Frag1344-1506_insertion1395 TAGATTCCCTTCGTCTCCCCGCTGCCAGGACGAAAGCGAGGCGGTCTGCAGCGAGTGGAAGTTCGCCGCCTGTAGCGGGAGACCGGGGTCTCTGAGCGTGGTGGACCGCCTGTGCCTCATGGCCTTCTCGGTCTTCACCATCATCTGCACCATCGGCATCCTGATGTCGGCTCCCAACTTCGTGGAGGCCGTGTCCAAAGACTTTGCGGCTGGGAGACGCCGACAAAAGT
nachra7_Frag1344-1506_insertion1398 TAGATTCCCTTCGTCTCCCCGCTGCCAGGACGAAAGCGAGGCGGTCTGCAGCGAGTGGAAGTTCGCCGCCTGTGTGAGCGGGAGACCGGGGTCTCTGAGCGTGGACCGCCTGTGCCTCATGGCCTTCTCGGTCTTCACCATCATCTGCACCATCGGCATCCTGATGTCGGCTCCCAACTTCGTGGAGGCCGTGTCCAAAGACTTTGCGGCTGGGAGACGCCGACAAAAGT
nachra7_Frag1344-1506_insertion1401 TAGATTCCCTTCGTCTCCCCGCTGCCAGGACGAAAGCGAGGCGGTCTGCAGCGAGTGGAAGTTCGCCGCCTGTGTGGTGAGCGGGAGACCGGGGTCTCTGAGCGACCGCCTGTGCCTCATGGCCTTCTCGGTCTTCACCATCATCTGCACCATCGGCATCCTGATGTCGGCTCCCAACTTCGTGGAGGCCGTGTCCAAAGACTTTGCGGCTGGGAGACGCCGACAAAAGT
nachra7_Frag1344-1506_insertion1404 TAGATTCCCTTCGTCTCCCCGCTGCCAGGACGAAAGCGAGGCGGTCTGCAGCGAGTGGAAGTTCGCCGCCTGTGTGGTGGACAGCGGGAGACCGGGGTCTCTGAGCCGCCTGTGCCTCATGGCCTTCTCGGTCTTCACCATCATCTGCACCATCGGCATCCTGATGTCGGCTCCCAACTTCGTGGAGGCCGTGTCCAAAGACTTTGCGGCTGGGAGACGCCGACAAAAGT
nachra7_Frag1344-1506_insertion1407 TAGATTCCCTTCGTCTCCCCGCTGCCAGGACGAAAGCGAGGCGGTCTGCAGCGAGTGGAAGTTCGCCGCCTGTGTGGTGGACCGCAGCGGGAGACCGGGGTCTCTGAGCCTGTGCCTCATGGCCTTCTCGGTCTTCACCATCATCTGCACCATCGGCATCCTGATGTCGGCTCCCAACTTCGTGGAGGCCGTGTCCAAAGACTTTGCGGCTGGGAGACGCCGACAAAAGT
nachra7_Frag1344-1506_insertion1410 TAGATTCCCTTCGTCTCCCCGCTGCCAGGACGAAAGCGAGGCGGTCTGCAGCGAGTGGAAGTTCGCCGCCTGTGTGGTGGACCGCCTGAGCGGGAGACCGGGGTCTCTGAGCTGCCTCATGGCCTTCTCGGTCTTCACCATCATCTGCACCATCGGCATCCTGATGTCGGCTCCCAACTTCGTGGAGGCCGTGTCCAAAGACTTTGCGGCTGGGAGACGCCGACAAAAGT
nachra7_Frag1344-1506_insertion1413 TAGATTCCCTTCGTCTCCCCGCTGCCAGGACGAAAGCGAGGCGGTCTGCAGCGAGTGGAAGTTCGCCGCCTGTGTGGTGGACCGCCTGTGCAGCGGGAGACCGGGGTCTCTGAGCCTCATGGCCTTCTCGGTCTTCACCATCATCTGCACCATCGGCATCCTGATGTCGGCTCCCAACTTCGTGGAGGCCGTGTCCAAAGACTTTGCGGCTGGGAGACGCCGACAAAAGT
nachra7_Frag1344-1506_insertion1416 TAGATTCCCTTCGTCTCCCCGCTGCCAGGACGAAAGCGAGGCGGTCTGCAGCGAGTGGAAGTTCGCCGCCTGTGTGGTGGACCGCCTGTGCCTCAGCGGGAGACCGGGGTCTCTGAGCATGGCCTTCTCGGTCTTCACCATCATCTGCACCATCGGCATCCTGATGTCGGCTCCCAACTTCGTGGAGGCCGTGTCCAAAGACTTTGCGGCTGGGAGACGCCGACAAAAGT
nachra7_Frag1344-1506_insertion1419 TAGATTCCCTTCGTCTCCCCGCTGCCAGGACGAAAGCGAGGCGGTCTGCAGCGAGTGGAAGTTCGCCGCCTGTGTGGTGGACCGCCTGTGCCTCATGAGCGGGAGACCGGGGTCTCTGAGCGCCTTCTCGGTCTTCACCATCATCTGCACCATCGGCATCCTGATGTCGGCTCCCAACTTCGTGGAGGCCGTGTCCAAAGACTTTGCGGCTGGGAGACGCCGACAAAAGT
nachra7_Frag1344-1506_insertion1422 TAGATTCCCTTCGTCTCCCCGCTGCCAGGACGAAAGCGAGGCGGTCTGCAGCGAGTGGAAGTTCGCCGCCTGTGTGGTGGACCGCCTGTGCCTCATGGCCAGCGGGAGACCGGGGTCTCTGAGCTTCTCGGTCTTCACCATCATCTGCACCATCGGCATCCTGATGTCGGCTCCCAACTTCGTGGAGGCCGTGTCCAAAGACTTTGCGGCTGGGAGACGCCGACAAAAGT
nachra7_Frag1344-1506_insertion1425 TAGATTCCCTTCGTCTCCCCGCTGCCAGGACGAAAGCGAGGCGGTCTGCAGCGAGTGGAAGTTCGCCGCCTGTGTGGTGGACCGCCTGTGCCTCATGGCCTTCAGCGGGAGACCGGGGTCTCTGAGCTCGGTCTTCACCATCATCTGCACCATCGGCATCCTGATGTCGGCTCCCAACTTCGTGGAGGCCGTGTCCAAAGACTTTGCGGCTGGGAGACGCCGACAAAAGT
nachra7_Frag1344-1506_insertion1428 TAGATTCCCTTCGTCTCCCCGCTGCCAGGACGAAAGCGAGGCGGTCTGCAGCGAGTGGAAGTTCGCCGCCTGTGTGGTGGACCGCCTGTGCCTCATGGCCTTCTCGAGCGGGAGACCGGGGTCTCTGAGCGTCTTCACCATCATCTGCACCATCGGCATCCTGATGTCGGCTCCCAACTTCGTGGAGGCCGTGTCCAAAGACTTTGCGGCTGGGAGACGCCGACAAAAGT
nachra7_Frag1344-1506_insertion1431 TAGATTCCCTTCGTCTCCCCGCTGCCAGGACGAAAGCGAGGCGGTCTGCAGCGAGTGGAAGTTCGCCGCCTGTGTGGTGGACCGCCTGTGCCTCATGGCCTTCTCGGTCAGCGGGAGACCGGGGTCTCTGAGCTTCACCATCATCTGCACCATCGGCATCCTGATGTCGGCTCCCAACTTCGTGGAGGCCGTGTCCAAAGACTTTGCGGCTGGGAGACGCCGACAAAAGT
nachra7_Frag1344-1506_insertion1434 TAGATTCCCTTCGTCTCCCCGCTGCCAGGACGAAAGCGAGGCGGTCTGCAGCGAGTGGAAGTTCGCCGCCTGTGTGGTGGACCGCCTGTGCCTCATGGCCTTCTCGGTCTTCAGCGGGAGACCGGGGTCTCTGAGCACCATCATCTGCACCATCGGCATCCTGATGTCGGCTCCCAACTTCGTGGAGGCCGTGTCCAAAGACTTTGCGGCTGGGAGACGCCGACAAAAGT
nachra7_Frag1344-1506_insertion1437 TAGATTCCCTTCGTCTCCCCGCTGCCAGGACGAAAGCGAGGCGGTCTGCAGCGAGTGGAAGTTCGCCGCCTGTGTGGTGGACCGCCTGTGCCTCATGGCCTTCTCGGTCTTCACCAGCGGGAGACCGGGGTCTCTGAGCATCATCTGCACCATCGGCATCCTGATGTCGGCTCCCAACTTCGTGGAGGCCGTGTCCAAAGACTTTGCGGCTGGGAGACGCCGACAAAAGT
nachra7_Frag1344-1506_insertion1440 TAGATTCCCTTCGTCTCCCCGCTGCCAGGACGAAAGCGAGGCGGTCTGCAGCGAGTGGAAGTTCGCCGCCTGTGTGGTGGACCGCCTGTGCCTCATGGCCTTCTCGGTCTTCACCATCAGCGGGAGACCGGGGTCTCTGAGCATCTGCACCATCGGCATCCTGATGTCGGCTCCCAACTTCGTGGAGGCCGTGTCCAAAGACTTTGCGGCTGGGAGACGCCGACAAAAGT
nachra7_Frag1344-1506_insertion1443 TAGATTCCCTTCGTCTCCCCGCTGCCAGGACGAAAGCGAGGCGGTCTGCAGCGAGTGGAAGTTCGCCGCCTGTGTGGTGGACCGCCTGTGCCTCATGGCCTTCTCGGTCTTCACCATCATCAGCGGGAGACCGGGGTCTCTGAGCTGCACCATCGGCATCCTGATGTCGGCTCCCAACTTCGTGGAGGCCGTGTCCAAAGACTTTGCGGCTGGGAGACGCCGACAAAAGT
nachra7_Frag1344-1506_insertion1446 TAGATTCCCTTCGTCTCCCCGCTGCCAGGACGAAAGCGAGGCGGTCTGCAGCGAGTGGAAGTTCGCCGCCTGTGTGGTGGACCGCCTGTGCCTCATGGCCTTCTCGGTCTTCACCATCATCTGCAGCGGGAGACCGGGGTCTCTGAGCACCATCGGCATCCTGATGTCGGCTCCCAACTTCGTGGAGGCCGTGTCCAAAGACTTTGCGGCTGGGAGACGCCGACAAAAGT
nachra7_Frag1344-1506_insertion1449 TAGATTCCCTTCGTCTCCCCGCTGCCAGGACGAAAGCGAGGCGGTCTGCAGCGAGTGGAAGTTCGCCGCCTGTGTGGTGGACCGCCTGTGCCTCATGGCCTTCTCGGTCTTCACCATCATCTGCACCAGCGGGAGACCGGGGTCTCTGAGCATCGGCATCCTGATGTCGGCTCCCAACTTCGTGGAGGCCGTGTCCAAAGACTTTGCGGCTGGGAGACGCCGACAAAAGT
nachra7_Frag1344-1506_insertion1452 TAGATTCCCTTCGTCTCCCCGCTGCCAGGACGAAAGCGAGGCGGTCTGCAGCGAGTGGAAGTTCGCCGCCTGTGTGGTGGACCGCCTGTGCCTCATGGCCTTCTCGGTCTTCACCATCATCTGCACCATCAGCGGGAGACCGGGGTCTCTGAGCGGCATCCTGATGTCGGCTCCCAACTTCGTGGAGGCCGTGTCCAAAGACTTTGCGGCTGGGAGACGCCGACAAAAGT
nachra7_Frag1344-1506_insertion1455 TAGATTCCCTTCGTCTCCCCGCTGCCAGGACGAAAGCGAGGCGGTCTGCAGCGAGTGGAAGTTCGCCGCCTGTGTGGTGGACCGCCTGTGCCTCATGGCCTTCTCGGTCTTCACCATCATCTGCACCATCGGCAGCGGGAGACCGGGGTCTCTGAGCATCCTGATGTCGGCTCCCAACTTCGTGGAGGCCGTGTCCAAAGACTTTGCGGCTGGGAGACGCCGACAAAAGT
nachra7_Frag1344-1506_insertion1458 TAGATTCCCTTCGTCTCCCCGCTGCCAGGACGAAAGCGAGGCGGTCTGCAGCGAGTGGAAGTTCGCCGCCTGTGTGGTGGACCGCCTGTGCCTCATGGCCTTCTCGGTCTTCACCATCATCTGCACCATCGGCATCAGCGGGAGACCGGGGTCTCTGAGCCTGATGTCGGCTCCCAACTTCGTGGAGGCCGTGTCCAAAGACTTTGCGGCTGGGAGACGCCGACAAAAGT
nachra7_Frag1344-1506_insertion1461 TAGATTCCCTTCGTCTCCCCGCTGCCAGGACGAAAGCGAGGCGGTCTGCAGCGAGTGGAAGTTCGCCGCCTGTGTGGTGGACCGCCTGTGCCTCATGGCCTTCTCGGTCTTCACCATCATCTGCACCATCGGCATCCTGAGCGGGAGACCGGGGTCTCTGAGCATGTCGGCTCCCAACTTCGTGGAGGCCGTGTCCAAAGACTTTGCGGCTGGGAGACGCCGACAAAAGT
nachra7_Frag1344-1506_insertion1464 TAGATTCCCTTCGTCTCCCCGCTGCCAGGACGAAAGCGAGGCGGTCTGCAGCGAGTGGAAGTTCGCCGCCTGTGTGGTGGACCGCCTGTGCCTCATGGCCTTCTCGGTCTTCACCATCATCTGCACCATCGGCATCCTGATGAGCGGGAGACCGGGGTCTCTGAGCTCGGCTCCCAACTTCGTGGAGGCCGTGTCCAAAGACTTTGCGGCTGGGAGACGCCGACAAAAGT
nachra7_Frag1344-1506_insertion1467 TAGATTCCCTTCGTCTCCCCGCTGCCAGGACGAAAGCGAGGCGGTCTGCAGCGAGTGGAAGTTCGCCGCCTGTGTGGTGGACCGCCTGTGCCTCATGGCCTTCTCGGTCTTCACCATCATCTGCACCATCGGCATCCTGATGTCGAGCGGGAGACCGGGGTCTCTGAGCGCTCCCAACTTCGTGGAGGCCGTGTCCAAAGACTTTGCGGCTGGGAGACGCCGACAAAAGT
nachra7_Frag1344-1506_insertion1470 TAGATTCCCTTCGTCTCCCCGCTGCCAGGACGAAAGCGAGGCGGTCTGCAGCGAGTGGAAGTTCGCCGCCTGTGTGGTGGACCGCCTGTGCCTCATGGCCTTCTCGGTCTTCACCATCATCTGCACCATCGGCATCCTGATGTCGGCTAGCGGGAGACCGGGGTCTCTGAGCCCCAACTTCGTGGAGGCCGTGTCCAAAGACTTTGCGGCTGGGAGACGCCGACAAAAGT
nachra7_Frag1344-1506_insertion1473 TAGATTCCCTTCGTCTCCCCGCTGCCAGGACGAAAGCGAGGCGGTCTGCAGCGAGTGGAAGTTCGCCGCCTGTGTGGTGGACCGCCTGTGCCTCATGGCCTTCTCGGTCTTCACCATCATCTGCACCATCGGCATCCTGATGTCGGCTCCCAGCGGGAGACCGGGGTCTCTGAGCAACTTCGTGGAGGCCGTGTCCAAAGACTTTGCGGCTGGGAGACGCCGACAAAAGT
nachra7_Frag1344-1506_insertion1476 TAGATTCCCTTCGTCTCCCCGCTGCCAGGACGAAAGCGAGGCGGTCTGCAGCGAGTGGAAGTTCGCCGCCTGTGTGGTGGACCGCCTGTGCCTCATGGCCTTCTCGGTCTTCACCATCATCTGCACCATCGGCATCCTGATGTCGGCTCCCAACAGCGGGAGACCGGGGTCTCTGAGCTTCGTGGAGGCCGTGTCCAAAGACTTTGCGGCTGGGAGACGCCGACAAAAGT
nachra7_Frag1344-1506_insertion1479 TAGATTCCCTTCGTCTCCCCGCTGCCAGGACGAAAGCGAGGCGGTCTGCAGCGAGTGGAAGTTCGCCGCCTGTGTGGTGGACCGCCTGTGCCTCATGGCCTTCTCGGTCTTCACCATCATCTGCACCATCGGCATCCTGATGTCGGCTCCCAACTTCAGCGGGAGACCGGGGTCTCTGAGCGTGGAGGCCGTGTCCAAAGACTTTGCGGCTGGGAGACGCCGACAAAAGT
nachra7_Frag1344-1506_insertion1482 TAGATTCCCTTCGTCTCCCCGCTGCCAGGACGAAAGCGAGGCGGTCTGCAGCGAGTGGAAGTTCGCCGCCTGTGTGGTGGACCGCCTGTGCCTCATGGCCTTCTCGGTCTTCACCATCATCTGCACCATCGGCATCCTGATGTCGGCTCCCAACTTCGTGAGCGGGAGACCGGGGTCTCTGAGCGAGGCCGTGTCCAAAGACTTTGCGGCTGGGAGACGCCGACAAAAGT
nachra7_Frag1344-1506_insertion1485 TAGATTCCCTTCGTCTCCCCGCTGCCAGGACGAAAGCGAGGCGGTCTGCAGCGAGTGGAAGTTCGCCGCCTGTGTGGTGGACCGCCTGTGCCTCATGGCCTTCTCGGTCTTCACCATCATCTGCACCATCGGCATCCTGATGTCGGCTCCCAACTTCGTGGAGAGCGGGAGACCGGGGTCTCTGAGCGCCGTGTCCAAAGACTTTGCGGCTGGGAGACGCCGACAAAAGT
nachra7_Frag1344-1506_insertion1488 TAGATTCCCTTCGTCTCCCCGCTGCCAGGACGAAAGCGAGGCGGTCTGCAGCGAGTGGAAGTTCGCCGCCTGTGTGGTGGACCGCCTGTGCCTCATGGCCTTCTCGGTCTTCACCATCATCTGCACCATCGGCATCCTGATGTCGGCTCCCAACTTCGTGGAGGCCAGCGGGAGACCGGGGTCTCTGAGCGTGTCCAAAGACTTTGCGGCTGGGAGACGCCGACAAAAGT
nachra7_Frag1344-1506_insertion1491 TAGATTCCCTTCGTCTCCCCGCTGCCAGGACGAAAGCGAGGCGGTCTGCAGCGAGTGGAAGTTCGCCGCCTGTGTGGTGGACCGCCTGTGCCTCATGGCCTTCTCGGTCTTCACCATCATCTGCACCATCGGCATCCTGATGTCGGCTCCCAACTTCGTGGAGGCCGTGAGCGGGAGACCGGGGTCTCTGAGCTCCAAAGACTTTGCGGCTGGGAGACGCCGACAAAAGT
nachra7_Frag1344-1506_insertion1494 TAGATTCCCTTCGTCTCCCCGCTGCCAGGACGAAAGCGAGGCGGTCTGCAGCGAGTGGAAGTTCGCCGCCTGTGTGGTGGACCGCCTGTGCCTCATGGCCTTCTCGGTCTTCACCATCATCTGCACCATCGGCATCCTGATGTCGGCTCCCAACTTCGTGGAGGCCGTGTCCAGCGGGAGACCGGGGTCTCTGAGCAAAGACTTTGCGGCTGGGAGACGCCGACAAAAGT
nachra7_Frag1344-1506_insertion1497 TAGATTCCCTTCGTCTCCCCGCTGCCAGGACGAAAGCGAGGCGGTCTGCAGCGAGTGGAAGTTCGCCGCCTGTGTGGTGGACCGCCTGTGCCTCATGGCCTTCTCGGTCTTCACCATCATCTGCACCATCGGCATCCTGATGTCGGCTCCCAACTTCGTGGAGGCCGTGTCCAAAAGCGGGAGACCGGGGTCTCTGAGCGACTTTGCGGCTGGGAGACGCCGACAAAAGT
nachra7_Frag1344-1506_insertion1500 TAGATTCCCTTCGTCTCCCCGCTGCCAGGACGAAAGCGAGGCGGTCTGCAGCGAGTGGAAGTTCGCCGCCTGTGTGGTGGACCGCCTGTGCCTCATGGCCTTCTCGGTCTTCACCATCATCTGCACCATCGGCATCCTGATGTCGGCTCCCAACTTCGTGGAGGCCGTGTCCAAAGACAGCGGGAGACCGGGGTCTCTGAGCTTTGCGGCTGGGAGACGCCGACAAAAGT
nachra7_Frag1344-1506_insertion1503 TAGATTCCCTTCGTCTCCCCGCTGCCAGGACGAAAGCGAGGCGGTCTGCAGCGAGTGGAAGTTCGCCGCCTGTGTGGTGGACCGCCTGTGCCTCATGGCCTTCTCGGTCTTCACCATCATCTGCACCATCGGCATCCTGATGTCGGCTCCCAACTTCGTGGAGGCCGTGTCCAAAGACTTTAGCGGGAGACCGGGGTCTCTGAGCGCGGCTGGGAGACGCCGACAAAAGT
nachra7_Frag1344-1506_insertion1506 TAGATTCCCTTCGTCTCCCCGCTGCCAGGACGAAAGCGAGGCGGTCTGCAGCGAGTGGAAGTTCGCCGCCTGTGTGGTGGACCGCCTGTGCCTCATGGCCTTCTCGGTCTTCACCATCATCTGCACCATCGGCATCCTGATGTCGGCTCCCAACTTCGTGGAGGCCGTGTCCAAAGACTTTGCGAGCGGGAGACCGGGGTCTCTGAGCGCTGGGAGACGCCGACAAAAGT
kir21_Frag3-162_insertion3 ACAGGTGGGATACGTCTCCCATGAGCGGGAGACCGGGGTCTCTGAGCGGATCAGTGCGAACCAATCGGTATTCTATCGTATCAAGCGAGGAGGACGGGATGAAACTGGCAACCATGGCTGTAGCCAACGGTTTTGGGAATGGGAAATCTAAGGTGCATACCCGCCAACAGTGCCGGTCACGCTTTGTCAAGAAAGATGGCCACTGTAATGGGAGACGGTATGGCGAGTCG
kir21_Frag3-162_insertion6 ACAGGTGGGATACGTCTCCCATGGGAAGCGGGAGACCGGGGTCTCTGAGCTCAGTGCGAACCAATCGGTATTCTATCGTATCAAGCGAGGAGGACGGGATGAAACTGGCAACCATGGCTGTAGCCAACGGTTTTGGGAATGGGAAATCTAAGGTGCATACCCGCCAACAGTGCCGGTCACGCTTTGTCAAGAAAGATGGCCACTGTAATGGGAGACGGTATGGCGAGTCG
kir21_Frag3-162_insertion9 ACAGGTGGGATACGTCTCCCATGGGATCAAGCGGGAGACCGGGGTCTCTGAGCGTGCGAACCAATCGGTATTCTATCGTATCAAGCGAGGAGGACGGGATGAAACTGGCAACCATGGCTGTAGCCAACGGTTTTGGGAATGGGAAATCTAAGGTGCATACCCGCCAACAGTGCCGGTCACGCTTTGTCAAGAAAGATGGCCACTGTAATGGGAGACGGTATGGCGAGTCG
kir21_Frag3-162_insertion12 ACAGGTGGGATACGTCTCCCATGGGATCAGTGAGCGGGAGACCGGGGTCTCTGAGCCGAACCAATCGGTATTCTATCGTATCAAGCGAGGAGGACGGGATGAAACTGGCAACCATGGCTGTAGCCAACGGTTTTGGGAATGGGAAATCTAAGGTGCATACCCGCCAACAGTGCCGGTCACGCTTTGTCAAGAAAGATGGCCACTGTAATGGGAGACGGTATGGCGAGTCG
kir21_Frag3-162_insertion15 ACAGGTGGGATACGTCTCCCATGGGATCAGTGCGAAGCGGGAGACCGGGGTCTCTGAGCACCAATCGGTATTCTATCGTATCAAGCGAGGAGGACGGGATGAAACTGGCAACCATGGCTGTAGCCAACGGTTTTGGGAATGGGAAATCTAAGGTGCATACCCGCCAACAGTGCCGGTCACGCTTTGTCAAGAAAGATGGCCACTGTAATGGGAGACGGTATGGCGAGTCG
kir21_Frag3-162_insertion18 ACAGGTGGGATACGTCTCCCATGGGATCAGTGCGAACCAGCGGGAGACCGGGGTCTCTGAGCAATCGGTATTCTATCGTATCAAGCGAGGAGGACGGGATGAAACTGGCAACCATGGCTGTAGCCAACGGTTTTGGGAATGGGAAATCTAAGGTGCATACCCGCCAACAGTGCCGGTCACGCTTTGTCAAGAAAGATGGCCACTGTAATGGGAGACGGTATGGCGAGTCG
kir21_Frag3-162_insertion21 ACAGGTGGGATACGTCTCCCATGGGATCAGTGCGAACCAATAGCGGGAGACCGGGGTCTCTGAGCCGGTATTCTATCGTATCAAGCGAGGAGGACGGGATGAAACTGGCAACCATGGCTGTAGCCAACGGTTTTGGGAATGGGAAATCTAAGGTGCATACCCGCCAACAGTGCCGGTCACGCTTTGTCAAGAAAGATGGCCACTGTAATGGGAGACGGTATGGCGAGTCG
kir21_Frag3-162_insertion24 ACAGGTGGGATACGTCTCCCATGGGATCAGTGCGAACCAATCGGAGCGGGAGACCGGGGTCTCTGAGCTATTCTATCGTATCAAGCGAGGAGGACGGGATGAAACTGGCAACCATGGCTGTAGCCAACGGTTTTGGGAATGGGAAATCTAAGGTGCATACCCGCCAACAGTGCCGGTCACGCTTTGTCAAGAAAGATGGCCACTGTAATGGGAGACGGTATGGCGAGTCG
kir21_Frag3-162_insertion27 ACAGGTGGGATACGTCTCCCATGGGATCAGTGCGAACCAATCGGTATAGCGGGAGACCGGGGTCTCTGAGCTCTATCGTATCAAGCGAGGAGGACGGGATGAAACTGGCAACCATGGCTGTAGCCAACGGTTTTGGGAATGGGAAATCTAAGGTGCATACCCGCCAACAGTGCCGGTCACGCTTTGTCAAGAAAGATGGCCACTGTAATGGGAGACGGTATGGCGAGTCG
kir21_Frag3-162_insertion30 ACAGGTGGGATACGTCTCCCATGGGATCAGTGCGAACCAATCGGTATTCTAGCGGGAGACCGGGGTCTCTGAGCATCGTATCAAGCGAGGAGGACGGGATGAAACTGGCAACCATGGCTGTAGCCAACGGTTTTGGGAATGGGAAATCTAAGGTGCATACCCGCCAACAGTGCCGGTCACGCTTTGTCAAGAAAGATGGCCACTGTAATGGGAGACGGTATGGCGAGTCG
kir21_Frag3-162_insertion33 ACAGGTGGGATACGTCTCCCATGGGATCAGTGCGAACCAATCGGTATTCTATCAGCGGGAGACCGGGGTCTCTGAGCGTATCAAGCGAGGAGGACGGGATGAAACTGGCAACCATGGCTGTAGCCAACGGTTTTGGGAATGGGAAATCTAAGGTGCATACCCGCCAACAGTGCCGGTCACGCTTTGTCAAGAAAGATGGCCACTGTAATGGGAGACGGTATGGCGAGTCG
kir21_Frag3-162_insertion36 ACAGGTGGGATACGTCTCCCATGGGATCAGTGCGAACCAATCGGTATTCTATCGTAAGCGGGAGACCGGGGTCTCTGAGCTCAAGCGAGGAGGACGGGATGAAACTGGCAACCATGGCTGTAGCCAACGGTTTTGGGAATGGGAAATCTAAGGTGCATACCCGCCAACAGTGCCGGTCACGCTTTGTCAAGAAAGATGGCCACTGTAATGGGAGACGGTATGGCGAGTCG
kir21_Frag3-162_insertion39 ACAGGTGGGATACGTCTCCCATGGGATCAGTGCGAACCAATCGGTATTCTATCGTATCAAGCGGGAGACCGGGGTCTCTGAGCAGCGAGGAGGACGGGATGAAACTGGCAACCATGGCTGTAGCCAACGGTTTTGGGAATGGGAAATCTAAGGTGCATACCCGCCAACAGTGCCGGTCACGCTTTGTCAAGAAAGATGGCCACTGTAATGGGAGACGGTATGGCGAGTCG
kir21_Frag3-162_insertion42 ACAGGTGGGATACGTCTCCCATGGGATCAGTGCGAACCAATCGGTATTCTATCGTATCAAGCAGCGGGAGACCGGGGTCTCTGAGCGAGGAGGACGGGATGAAACTGGCAACCATGGCTGTAGCCAACGGTTTTGGGAATGGGAAATCTAAGGTGCATACCCGCCAACAGTGCCGGTCACGCTTTGTCAAGAAAGATGGCCACTGTAATGGGAGACGGTATGGCGAGTCG
kir21_Frag3-162_insertion45 ACAGGTGGGATACGTCTCCCATGGGATCAGTGCGAACCAATCGGTATTCTATCGTATCAAGCGAGAGCGGGAGACCGGGGTCTCTGAGCGAGGACGGGATGAAACTGGCAACCATGGCTGTAGCCAACGGTTTTGGGAATGGGAAATCTAAGGTGCATACCCGCCAACAGTGCCGGTCACGCTTTGTCAAGAAAGATGGCCACTGTAATGGGAGACGGTATGGCGAGTCG
kir21_Frag3-162_insertion48 ACAGGTGGGATACGTCTCCCATGGGATCAGTGCGAACCAATCGGTATTCTATCGTATCAAGCGAGGAGAGCGGGAGACCGGGGTCTCTGAGCGACGGGATGAAACTGGCAACCATGGCTGTAGCCAACGGTTTTGGGAATGGGAAATCTAAGGTGCATACCCGCCAACAGTGCCGGTCACGCTTTGTCAAGAAAGATGGCCACTGTAATGGGAGACGGTATGGCGAGTCG
kir21_Frag3-162_insertion51 ACAGGTGGGATACGTCTCCCATGGGATCAGTGCGAACCAATCGGTATTCTATCGTATCAAGCGAGGAGGACAGCGGGAGACCGGGGTCTCTGAGCGGGATGAAACTGGCAACCATGGCTGTAGCCAACGGTTTTGGGAATGGGAAATCTAAGGTGCATACCCGCCAACAGTGCCGGTCACGCTTTGTCAAGAAAGATGGCCACTGTAATGGGAGACGGTATGGCGAGTCG
kir21_Frag3-162_insertion54 ACAGGTGGGATACGTCTCCCATGGGATCAGTGCGAACCAATCGGTATTCTATCGTATCAAGCGAGGAGGACGGGAGCGGGAGACCGGGGTCTCTGAGCATGAAACTGGCAACCATGGCTGTAGCCAACGGTTTTGGGAATGGGAAATCTAAGGTGCATACCCGCCAACAGTGCCGGTCACGCTTTGTCAAGAAAGATGGCCACTGTAATGGGAGACGGTATGGCGAGTCG
kir21_Frag3-162_insertion57 ACAGGTGGGATACGTCTCCCATGGGATCAGTGCGAACCAATCGGTATTCTATCGTATCAAGCGAGGAGGACGGGATGAGCGGGAGACCGGGGTCTCTGAGCAAACTGGCAACCATGGCTGTAGCCAACGGTTTTGGGAATGGGAAATCTAAGGTGCATACCCGCCAACAGTGCCGGTCACGCTTTGTCAAGAAAGATGGCCACTGTAATGGGAGACGGTATGGCGAGTCG
kir21_Frag3-162_insertion60 ACAGGTGGGATACGTCTCCCATGGGATCAGTGCGAACCAATCGGTATTCTATCGTATCAAGCGAGGAGGACGGGATGAAAAGCGGGAGACCGGGGTCTCTGAGCCTGGCAACCATGGCTGTAGCCAACGGTTTTGGGAATGGGAAATCTAAGGTGCATACCCGCCAACAGTGCCGGTCACGCTTTGTCAAGAAAGATGGCCACTGTAATGGGAGACGGTATGGCGAGTCG
kir21_Frag3-162_insertion63 ACAGGTGGGATACGTCTCCCATGGGATCAGTGCGAACCAATCGGTATTCTATCGTATCAAGCGAGGAGGACGGGATGAAACTGAGCGGGAGACCGGGGTCTCTGAGCGCAACCATGGCTGTAGCCAACGGTTTTGGGAATGGGAAATCTAAGGTGCATACCCGCCAACAGTGCCGGTCACGCTTTGTCAAGAAAGATGGCCACTGTAATGGGAGACGGTATGGCGAGTCG
kir21_Frag3-162_insertion66 ACAGGTGGGATACGTCTCCCATGGGATCAGTGCGAACCAATCGGTATTCTATCGTATCAAGCGAGGAGGACGGGATGAAACTGGCAAGCGGGAGACCGGGGTCTCTGAGCACCATGGCTGTAGCCAACGGTTTTGGGAATGGGAAATCTAAGGTGCATACCCGCCAACAGTGCCGGTCACGCTTTGTCAAGAAAGATGGCCACTGTAATGGGAGACGGTATGGCGAGTCG
kir21_Frag3-162_insertion69 ACAGGTGGGATACGTCTCCCATGGGATCAGTGCGAACCAATCGGTATTCTATCGTATCAAGCGAGGAGGACGGGATGAAACTGGCAACCAGCGGGAGACCGGGGTCTCTGAGCATGGCTGTAGCCAACGGTTTTGGGAATGGGAAATCTAAGGTGCATACCCGCCAACAGTGCCGGTCACGCTTTGTCAAGAAAGATGGCCACTGTAATGGGAGACGGTATGGCGAGTCG
kir21_Frag3-162_insertion72 ACAGGTGGGATACGTCTCCCATGGGATCAGTGCGAACCAATCGGTATTCTATCGTATCAAGCGAGGAGGACGGGATGAAACTGGCAACCATGAGCGGGAGACCGGGGTCTCTGAGCGCTGTAGCCAACGGTTTTGGGAATGGGAAATCTAAGGTGCATACCCGCCAACAGTGCCGGTCACGCTTTGTCAAGAAAGATGGCCACTGTAATGGGAGACGGTATGGCGAGTCG
kir21_Frag3-162_insertion75 ACAGGTGGGATACGTCTCCCATGGGATCAGTGCGAACCAATCGGTATTCTATCGTATCAAGCGAGGAGGACGGGATGAAACTGGCAACCATGGCTAGCGGGAGACCGGGGTCTCTGAGCGTAGCCAACGGTTTTGGGAATGGGAAATCTAAGGTGCATACCCGCCAACAGTGCCGGTCACGCTTTGTCAAGAAAGATGGCCACTGTAATGGGAGACGGTATGGCGAGTCG
kir21_Frag3-162_insertion78 ACAGGTGGGATACGTCTCCCATGGGATCAGTGCGAACCAATCGGTATTCTATCGTATCAAGCGAGGAGGACGGGATGAAACTGGCAACCATGGCTGTAAGCGGGAGACCGGGGTCTCTGAGCGCCAACGGTTTTGGGAATGGGAAATCTAAGGTGCATACCCGCCAACAGTGCCGGTCACGCTTTGTCAAGAAAGATGGCCACTGTAATGGGAGACGGTATGGCGAGTCG
kir21_Frag3-162_insertion81 ACAGGTGGGATACGTCTCCCATGGGATCAGTGCGAACCAATCGGTATTCTATCGTATCAAGCGAGGAGGACGGGATGAAACTGGCAACCATGGCTGTAGCCAGCGGGAGACCGGGGTCTCTGAGCAACGGTTTTGGGAATGGGAAATCTAAGGTGCATACCCGCCAACAGTGCCGGTCACGCTTTGTCAAGAAAGATGGCCACTGTAATGGGAGACGGTATGGCGAGTCG
kir21_Frag3-162_insertion84 ACAGGTGGGATACGTCTCCCATGGGATCAGTGCGAACCAATCGGTATTCTATCGTATCAAGCGAGGAGGACGGGATGAAACTGGCAACCATGGCTGTAGCCAACAGCGGGAGACCGGGGTCTCTGAGCGGTTTTGGGAATGGGAAATCTAAGGTGCATACCCGCCAACAGTGCCGGTCACGCTTTGTCAAGAAAGATGGCCACTGTAATGGGAGACGGTATGGCGAGTCG
kir21_Frag3-162_insertion87 ACAGGTGGGATACGTCTCCCATGGGATCAGTGCGAACCAATCGGTATTCTATCGTATCAAGCGAGGAGGACGGGATGAAACTGGCAACCATGGCTGTAGCCAACGGTAGCGGGAGACCGGGGTCTCTGAGCTTTGGGAATGGGAAATCTAAGGTGCATACCCGCCAACAGTGCCGGTCACGCTTTGTCAAGAAAGATGGCCACTGTAATGGGAGACGGTATGGCGAGTCG
kir21_Frag3-162_insertion90 ACAGGTGGGATACGTCTCCCATGGGATCAGTGCGAACCAATCGGTATTCTATCGTATCAAGCGAGGAGGACGGGATGAAACTGGCAACCATGGCTGTAGCCAACGGTTTTAGCGGGAGACCGGGGTCTCTGAGCGGGAATGGGAAATCTAAGGTGCATACCCGCCAACAGTGCCGGTCACGCTTTGTCAAGAAAGATGGCCACTGTAATGGGAGACGGTATGGCGAGTCG
kir21_Frag3-162_insertion93 ACAGGTGGGATACGTCTCCCATGGGATCAGTGCGAACCAATCGGTATTCTATCGTATCAAGCGAGGAGGACGGGATGAAACTGGCAACCATGGCTGTAGCCAACGGTTTTGGGAGCGGGAGACCGGGGTCTCTGAGCAATGGGAAATCTAAGGTGCATACCCGCCAACAGTGCCGGTCACGCTTTGTCAAGAAAGATGGCCACTGTAATGGGAGACGGTATGGCGAGTCG
kir21_Frag3-162_insertion96 ACAGGTGGGATACGTCTCCCATGGGATCAGTGCGAACCAATCGGTATTCTATCGTATCAAGCGAGGAGGACGGGATGAAACTGGCAACCATGGCTGTAGCCAACGGTTTTGGGAATAGCGGGAGACCGGGGTCTCTGAGCGGGAAATCTAAGGTGCATACCCGCCAACAGTGCCGGTCACGCTTTGTCAAGAAAGATGGCCACTGTAATGGGAGACGGTATGGCGAGTCG
kir21_Frag3-162_insertion99 ACAGGTGGGATACGTCTCCCATGGGATCAGTGCGAACCAATCGGTATTCTATCGTATCAAGCGAGGAGGACGGGATGAAACTGGCAACCATGGCTGTAGCCAACGGTTTTGGGAATGGGAGCGGGAGACCGGGGTCTCTGAGCAAATCTAAGGTGCATACCCGCCAACAGTGCCGGTCACGCTTTGTCAAGAAAGATGGCCACTGTAATGGGAGACGGTATGGCGAGTCG
kir21_Frag3-162_insertion102 ACAGGTGGGATACGTCTCCCATGGGATCAGTGCGAACCAATCGGTATTCTATCGTATCAAGCGAGGAGGACGGGATGAAACTGGCAACCATGGCTGTAGCCAACGGTTTTGGGAATGGGAAAAGCGGGAGACCGGGGTCTCTGAGCTCTAAGGTGCATACCCGCCAACAGTGCCGGTCACGCTTTGTCAAGAAAGATGGCCACTGTAATGGGAGACGGTATGGCGAGTCG
kir21_Frag3-162_insertion105 ACAGGTGGGATACGTCTCCCATGGGATCAGTGCGAACCAATCGGTATTCTATCGTATCAAGCGAGGAGGACGGGATGAAACTGGCAACCATGGCTGTAGCCAACGGTTTTGGGAATGGGAAATCTAGCGGGAGACCGGGGTCTCTGAGCAAGGTGCATACCCGCCAACAGTGCCGGTCACGCTTTGTCAAGAAAGATGGCCACTGTAATGGGAGACGGTATGGCGAGTCG
kir21_Frag3-162_insertion108 ACAGGTGGGATACGTCTCCCATGGGATCAGTGCGAACCAATCGGTATTCTATCGTATCAAGCGAGGAGGACGGGATGAAACTGGCAACCATGGCTGTAGCCAACGGTTTTGGGAATGGGAAATCTAAGAGCGGGAGACCGGGGTCTCTGAGCGTGCATACCCGCCAACAGTGCCGGTCACGCTTTGTCAAGAAAGATGGCCACTGTAATGGGAGACGGTATGGCGAGTCG
kir21_Frag3-162_insertion111 ACAGGTGGGATACGTCTCCCATGGGATCAGTGCGAACCAATCGGTATTCTATCGTATCAAGCGAGGAGGACGGGATGAAACTGGCAACCATGGCTGTAGCCAACGGTTTTGGGAATGGGAAATCTAAGGTGAGCGGGAGACCGGGGTCTCTGAGCCATACCCGCCAACAGTGCCGGTCACGCTTTGTCAAGAAAGATGGCCACTGTAATGGGAGACGGTATGGCGAGTCG
kir21_Frag3-162_insertion114 ACAGGTGGGATACGTCTCCCATGGGATCAGTGCGAACCAATCGGTATTCTATCGTATCAAGCGAGGAGGACGGGATGAAACTGGCAACCATGGCTGTAGCCAACGGTTTTGGGAATGGGAAATCTAAGGTGCATAGCGGGAGACCGGGGTCTCTGAGCACCCGCCAACAGTGCCGGTCACGCTTTGTCAAGAAAGATGGCCACTGTAATGGGAGACGGTATGGCGAGTCG
kir21_Frag3-162_insertion117 ACAGGTGGGATACGTCTCCCATGGGATCAGTGCGAACCAATCGGTATTCTATCGTATCAAGCGAGGAGGACGGGATGAAACTGGCAACCATGGCTGTAGCCAACGGTTTTGGGAATGGGAAATCTAAGGTGCATACCAGCGGGAGACCGGGGTCTCTGAGCCGCCAACAGTGCCGGTCACGCTTTGTCAAGAAAGATGGCCACTGTAATGGGAGACGGTATGGCGAGTCG
kir21_Frag3-162_insertion120 ACAGGTGGGATACGTCTCCCATGGGATCAGTGCGAACCAATCGGTATTCTATCGTATCAAGCGAGGAGGACGGGATGAAACTGGCAACCATGGCTGTAGCCAACGGTTTTGGGAATGGGAAATCTAAGGTGCATACCCGCAGCGGGAGACCGGGGTCTCTGAGCCAACAGTGCCGGTCACGCTTTGTCAAGAAAGATGGCCACTGTAATGGGAGACGGTATGGCGAGTCG
kir21_Frag3-162_insertion123 ACAGGTGGGATACGTCTCCCATGGGATCAGTGCGAACCAATCGGTATTCTATCGTATCAAGCGAGGAGGACGGGATGAAACTGGCAACCATGGCTGTAGCCAACGGTTTTGGGAATGGGAAATCTAAGGTGCATACCCGCCAAAGCGGGAGACCGGGGTCTCTGAGCCAGTGCCGGTCACGCTTTGTCAAGAAAGATGGCCACTGTAATGGGAGACGGTATGGCGAGTCG
kir21_Frag3-162_insertion126 ACAGGTGGGATACGTCTCCCATGGGATCAGTGCGAACCAATCGGTATTCTATCGTATCAAGCGAGGAGGACGGGATGAAACTGGCAACCATGGCTGTAGCCAACGGTTTTGGGAATGGGAAATCTAAGGTGCATACCCGCCAACAGAGCGGGAGACCGGGGTCTCTGAGCTGCCGGTCACGCTTTGTCAAGAAAGATGGCCACTGTAATGGGAGACGGTATGGCGAGTCG
kir21_Frag3-162_insertion129 ACAGGTGGGATACGTCTCCCATGGGATCAGTGCGAACCAATCGGTATTCTATCGTATCAAGCGAGGAGGACGGGATGAAACTGGCAACCATGGCTGTAGCCAACGGTTTTGGGAATGGGAAATCTAAGGTGCATACCCGCCAACAGTGCAGCGGGAGACCGGGGTCTCTGAGCCGGTCACGCTTTGTCAAGAAAGATGGCCACTGTAATGGGAGACGGTATGGCGAGTCG
kir21_Frag3-162_insertion132 ACAGGTGGGATACGTCTCCCATGGGATCAGTGCGAACCAATCGGTATTCTATCGTATCAAGCGAGGAGGACGGGATGAAACTGGCAACCATGGCTGTAGCCAACGGTTTTGGGAATGGGAAATCTAAGGTGCATACCCGCCAACAGTGCCGGAGCGGGAGACCGGGGTCTCTGAGCTCACGCTTTGTCAAGAAAGATGGCCACTGTAATGGGAGACGGTATGGCGAGTCG
kir21_Frag3-162_insertion135 ACAGGTGGGATACGTCTCCCATGGGATCAGTGCGAACCAATCGGTATTCTATCGTATCAAGCGAGGAGGACGGGATGAAACTGGCAACCATGGCTGTAGCCAACGGTTTTGGGAATGGGAAATCTAAGGTGCATACCCGCCAACAGTGCCGGTCAAGCGGGAGACCGGGGTCTCTGAGCCGCTTTGTCAAGAAAGATGGCCACTGTAATGGGAGACGGTATGGCGAGTCG
kir21_Frag3-162_insertion138 ACAGGTGGGATACGTCTCCCATGGGATCAGTGCGAACCAATCGGTATTCTATCGTATCAAGCGAGGAGGACGGGATGAAACTGGCAACCATGGCTGTAGCCAACGGTTTTGGGAATGGGAAATCTAAGGTGCATACCCGCCAACAGTGCCGGTCACGCAGCGGGAGACCGGGGTCTCTGAGCTTTGTCAAGAAAGATGGCCACTGTAATGGGAGACGGTATGGCGAGTCG
kir21_Frag3-162_insertion141 ACAGGTGGGATACGTCTCCCATGGGATCAGTGCGAACCAATCGGTATTCTATCGTATCAAGCGAGGAGGACGGGATGAAACTGGCAACCATGGCTGTAGCCAACGGTTTTGGGAATGGGAAATCTAAGGTGCATACCCGCCAACAGTGCCGGTCACGCTTTAGCGGGAGACCGGGGTCTCTGAGCGTCAAGAAAGATGGCCACTGTAATGGGAGACGGTATGGCGAGTCG
kir21_Frag3-162_insertion144 ACAGGTGGGATACGTCTCCCATGGGATCAGTGCGAACCAATCGGTATTCTATCGTATCAAGCGAGGAGGACGGGATGAAACTGGCAACCATGGCTGTAGCCAACGGTTTTGGGAATGGGAAATCTAAGGTGCATACCCGCCAACAGTGCCGGTCACGCTTTGTCAGCGGGAGACCGGGGTCTCTGAGCAAGAAAGATGGCCACTGTAATGGGAGACGGTATGGCGAGTCG
kir21_Frag3-162_insertion147 ACAGGTGGGATACGTCTCCCATGGGATCAGTGCGAACCAATCGGTATTCTATCGTATCAAGCGAGGAGGACGGGATGAAACTGGCAACCATGGCTGTAGCCAACGGTTTTGGGAATGGGAAATCTAAGGTGCATACCCGCCAACAGTGCCGGTCACGCTTTGTCAAGAGCGGGAGACCGGGGTCTCTGAGCAAAGATGGCCACTGTAATGGGAGACGGTATGGCGAGTCG
kir21_Frag3-162_insertion150 ACAGGTGGGATACGTCTCCCATGGGATCAGTGCGAACCAATCGGTATTCTATCGTATCAAGCGAGGAGGACGGGATGAAACTGGCAACCATGGCTGTAGCCAACGGTTTTGGGAATGGGAAATCTAAGGTGCATACCCGCCAACAGTGCCGGTCACGCTTTGTCAAGAAAAGCGGGAGACCGGGGTCTCTGAGCGATGGCCACTGTAATGGGAGACGGTATGGCGAGTCG
kir21_Frag3-162_insertion153 ACAGGTGGGATACGTCTCCCATGGGATCAGTGCGAACCAATCGGTATTCTATCGTATCAAGCGAGGAGGACGGGATGAAACTGGCAACCATGGCTGTAGCCAACGGTTTTGGGAATGGGAAATCTAAGGTGCATACCCGCCAACAGTGCCGGTCACGCTTTGTCAAGAAAGATAGCGGGAGACCGGGGTCTCTGAGCGGCCACTGTAATGGGAGACGGTATGGCGAGTCG
kir21_Frag3-162_insertion156 ACAGGTGGGATACGTCTCCCATGGGATCAGTGCGAACCAATCGGTATTCTATCGTATCAAGCGAGGAGGACGGGATGAAACTGGCAACCATGGCTGTAGCCAACGGTTTTGGGAATGGGAAATCTAAGGTGCATACCCGCCAACAGTGCCGGTCACGCTTTGTCAAGAAAGATGGCAGCGGGAGACCGGGGTCTCTGAGCCACTGTAATGGGAGACGGTATGGCGAGTCG
kir21_Frag3-162_insertion159 ACAGGTGGGATACGTCTCCCATGGGATCAGTGCGAACCAATCGGTATTCTATCGTATCAAGCGAGGAGGACGGGATGAAACTGGCAACCATGGCTGTAGCCAACGGTTTTGGGAATGGGAAATCTAAGGTGCATACCCGCCAACAGTGCCGGTCACGCTTTGTCAAGAAAGATGGCCACAGCGGGAGACCGGGGTCTCTGAGCTGTAATGGGAGACGGTATGGCGAGTCG
kir21_Frag3-162_insertion162 ACAGGTGGGATACGTCTCCCATGGGATCAGTGCGAACCAATCGGTATTCTATCGTATCAAGCGAGGAGGACGGGATGAAACTGGCAACCATGGCTGTAGCCAACGGTTTTGGGAATGGGAAATCTAAGGTGCATACCCGCCAACAGTGCCGGTCACGCTTTGTCAAGAAAGATGGCCACTGTAGCGGGAGACCGGGGTCTCTGAGCAATGGGAGACGGTATGGCGAGTCG
kir21_Frag165-330_insertion165 CGTCTGTGACGTCTCCTAATAGCGGGAGACCGGGGTCTCTGAGCGTGCAGTTCATAAACGTGGGGGAAAAGGGTCAACGGTACTTGGCCGATATTTTCACAACCTGCGTTGATATCCGCTGGCGCTGGATGTTGGTTATTTTTTGTCTCGCCTTCGTACTGTCTTGGCTCTTCTTTGGCTGCGTTTTTTGGTTGATCGCTTTGTTGCATGGAGGGAGACGGGTGCATGCG
kir21_Frag165-330_insertion168 CGTCTGTGACGTCTCCTAATGTGAGCGGGAGACCGGGGTCTCTGAGCCAGTTCATAAACGTGGGGGAAAAGGGTCAACGGTACTTGGCCGATATTTTCACAACCTGCGTTGATATCCGCTGGCGCTGGATGTTGGTTATTTTTTGTCTCGCCTTCGTACTGTCTTGGCTCTTCTTTGGCTGCGTTTTTTGGTTGATCGCTTTGTTGCATGGAGGGAGACGGGTGCATGCG
kir21_Frag165-330_insertion171 CGTCTGTGACGTCTCCTAATGTGCAGAGCGGGAGACCGGGGTCTCTGAGCTTCATAAACGTGGGGGAAAAGGGTCAACGGTACTTGGCCGATATTTTCACAACCTGCGTTGATATCCGCTGGCGCTGGATGTTGGTTATTTTTTGTCTCGCCTTCGTACTGTCTTGGCTCTTCTTTGGCTGCGTTTTTTGGTTGATCGCTTTGTTGCATGGAGGGAGACGGGTGCATGCG
kir21_Frag165-330_insertion174 CGTCTGTGACGTCTCCTAATGTGCAGTTCAGCGGGAGACCGGGGTCTCTGAGCATAAACGTGGGGGAAAAGGGTCAACGGTACTTGGCCGATATTTTCACAACCTGCGTTGATATCCGCTGGCGCTGGATGTTGGTTATTTTTTGTCTCGCCTTCGTACTGTCTTGGCTCTTCTTTGGCTGCGTTTTTTGGTTGATCGCTTTGTTGCATGGAGGGAGACGGGTGCATGCG
kir21_Frag165-330_insertion177 CGTCTGTGACGTCTCCTAATGTGCAGTTCATAAGCGGGAGACCGGGGTCTCTGAGCAACGTGGGGGAAAAGGGTCAACGGTACTTGGCCGATATTTTCACAACCTGCGTTGATATCCGCTGGCGCTGGATGTTGGTTATTTTTTGTCTCGCCTTCGTACTGTCTTGGCTCTTCTTTGGCTGCGTTTTTTGGTTGATCGCTTTGTTGCATGGAGGGAGACGGGTGCATGCG
kir21_Frag165-330_insertion180 CGTCTGTGACGTCTCCTAATGTGCAGTTCATAAACAGCGGGAGACCGGGGTCTCTGAGCGTGGGGGAAAAGGGTCAACGGTACTTGGCCGATATTTTCACAACCTGCGTTGATATCCGCTGGCGCTGGATGTTGGTTATTTTTTGTCTCGCCTTCGTACTGTCTTGGCTCTTCTTTGGCTGCGTTTTTTGGTTGATCGCTTTGTTGCATGGAGGGAGACGGGTGCATGCG
kir21_Frag165-330_insertion183 CGTCTGTGACGTCTCCTAATGTGCAGTTCATAAACGTGAGCGGGAGACCGGGGTCTCTGAGCGGGGAAAAGGGTCAACGGTACTTGGCCGATATTTTCACAACCTGCGTTGATATCCGCTGGCGCTGGATGTTGGTTATTTTTTGTCTCGCCTTCGTACTGTCTTGGCTCTTCTTTGGCTGCGTTTTTTGGTTGATCGCTTTGTTGCATGGAGGGAGACGGGTGCATGCG
kir21_Frag165-330_insertion186 CGTCTGTGACGTCTCCTAATGTGCAGTTCATAAACGTGGGGAGCGGGAGACCGGGGTCTCTGAGCGAAAAGGGTCAACGGTACTTGGCCGATATTTTCACAACCTGCGTTGATATCCGCTGGCGCTGGATGTTGGTTATTTTTTGTCTCGCCTTCGTACTGTCTTGGCTCTTCTTTGGCTGCGTTTTTTGGTTGATCGCTTTGTTGCATGGAGGGAGACGGGTGCATGCG
kir21_Frag165-330_insertion189 CGTCTGTGACGTCTCCTAATGTGCAGTTCATAAACGTGGGGGAAAGCGGGAGACCGGGGTCTCTGAGCAAGGGTCAACGGTACTTGGCCGATATTTTCACAACCTGCGTTGATATCCGCTGGCGCTGGATGTTGGTTATTTTTTGTCTCGCCTTCGTACTGTCTTGGCTCTTCTTTGGCTGCGTTTTTTGGTTGATCGCTTTGTTGCATGGAGGGAGACGGGTGCATGCG
kir21_Frag165-330_insertion192 CGTCTGTGACGTCTCCTAATGTGCAGTTCATAAACGTGGGGGAAAAGAGCGGGAGACCGGGGTCTCTGAGCGGTCAACGGTACTTGGCCGATATTTTCACAACCTGCGTTGATATCCGCTGGCGCTGGATGTTGGTTATTTTTTGTCTCGCCTTCGTACTGTCTTGGCTCTTCTTTGGCTGCGTTTTTTGGTTGATCGCTTTGTTGCATGGAGGGAGACGGGTGCATGCG
kir21_Frag165-330_insertion195 CGTCTGTGACGTCTCCTAATGTGCAGTTCATAAACGTGGGGGAAAAGGGTAGCGGGAGACCGGGGTCTCTGAGCCAACGGTACTTGGCCGATATTTTCACAACCTGCGTTGATATCCGCTGGCGCTGGATGTTGGTTATTTTTTGTCTCGCCTTCGTACTGTCTTGGCTCTTCTTTGGCTGCGTTTTTTGGTTGATCGCTTTGTTGCATGGAGGGAGACGGGTGCATGCG
kir21_Frag165-330_insertion198 CGTCTGTGACGTCTCCTAATGTGCAGTTCATAAACGTGGGGGAAAAGGGTCAAAGCGGGAGACCGGGGTCTCTGAGCCGGTACTTGGCCGATATTTTCACAACCTGCGTTGATATCCGCTGGCGCTGGATGTTGGTTATTTTTTGTCTCGCCTTCGTACTGTCTTGGCTCTTCTTTGGCTGCGTTTTTTGGTTGATCGCTTTGTTGCATGGAGGGAGACGGGTGCATGCG
kir21_Frag165-330_insertion201 CGTCTGTGACGTCTCCTAATGTGCAGTTCATAAACGTGGGGGAAAAGGGTCAACGGAGCGGGAGACCGGGGTCTCTGAGCTACTTGGCCGATATTTTCACAACCTGCGTTGATATCCGCTGGCGCTGGATGTTGGTTATTTTTTGTCTCGCCTTCGTACTGTCTTGGCTCTTCTTTGGCTGCGTTTTTTGGTTGATCGCTTTGTTGCATGGAGGGAGACGGGTGCATGCG
kir21_Frag165-330_insertion204 CGTCTGTGACGTCTCCTAATGTGCAGTTCATAAACGTGGGGGAAAAGGGTCAACGGTACAGCGGGAGACCGGGGTCTCTGAGCTTGGCCGATATTTTCACAACCTGCGTTGATATCCGCTGGCGCTGGATGTTGGTTATTTTTTGTCTCGCCTTCGTACTGTCTTGGCTCTTCTTTGGCTGCGTTTTTTGGTTGATCGCTTTGTTGCATGGAGGGAGACGGGTGCATGCG
kir21_Frag165-330_insertion207 CGTCTGTGACGTCTCCTAATGTGCAGTTCATAAACGTGGGGGAAAAGGGTCAACGGTACTTGAGCGGGAGACCGGGGTCTCTGAGCGCCGATATTTTCACAACCTGCGTTGATATCCGCTGGCGCTGGATGTTGGTTATTTTTTGTCTCGCCTTCGTACTGTCTTGGCTCTTCTTTGGCTGCGTTTTTTGGTTGATCGCTTTGTTGCATGGAGGGAGACGGGTGCATGCG
kir21_Frag165-330_insertion210 CGTCTGTGACGTCTCCTAATGTGCAGTTCATAAACGTGGGGGAAAAGGGTCAACGGTACTTGGCCAGCGGGAGACCGGGGTCTCTGAGCGATATTTTCACAACCTGCGTTGATATCCGCTGGCGCTGGATGTTGGTTATTTTTTGTCTCGCCTTCGTACTGTCTTGGCTCTTCTTTGGCTGCGTTTTTTGGTTGATCGCTTTGTTGCATGGAGGGAGACGGGTGCATGCG
kir21_Frag165-330_insertion213 CGTCTGTGACGTCTCCTAATGTGCAGTTCATAAACGTGGGGGAAAAGGGTCAACGGTACTTGGCCGATAGCGGGAGACCGGGGTCTCTGAGCATTTTCACAACCTGCGTTGATATCCGCTGGCGCTGGATGTTGGTTATTTTTTGTCTCGCCTTCGTACTGTCTTGGCTCTTCTTTGGCTGCGTTTTTTGGTTGATCGCTTTGTTGCATGGAGGGAGACGGGTGCATGCG
kir21_Frag165-330_insertion216 CGTCTGTGACGTCTCCTAATGTGCAGTTCATAAACGTGGGGGAAAAGGGTCAACGGTACTTGGCCGATATTAGCGGGAGACCGGGGTCTCTGAGCTTCACAACCTGCGTTGATATCCGCTGGCGCTGGATGTTGGTTATTTTTTGTCTCGCCTTCGTACTGTCTTGGCTCTTCTTTGGCTGCGTTTTTTGGTTGATCGCTTTGTTGCATGGAGGGAGACGGGTGCATGCG
kir21_Frag165-330_insertion219 CGTCTGTGACGTCTCCTAATGTGCAGTTCATAAACGTGGGGGAAAAGGGTCAACGGTACTTGGCCGATATTTTCAGCGGGAGACCGGGGTCTCTGAGCACAACCTGCGTTGATATCCGCTGGCGCTGGATGTTGGTTATTTTTTGTCTCGCCTTCGTACTGTCTTGGCTCTTCTTTGGCTGCGTTTTTTGGTTGATCGCTTTGTTGCATGGAGGGAGACGGGTGCATGCG
kir21_Frag165-330_insertion222 CGTCTGTGACGTCTCCTAATGTGCAGTTCATAAACGTGGGGGAAAAGGGTCAACGGTACTTGGCCGATATTTTCACAAGCGGGAGACCGGGGTCTCTGAGCACCTGCGTTGATATCCGCTGGCGCTGGATGTTGGTTATTTTTTGTCTCGCCTTCGTACTGTCTTGGCTCTTCTTTGGCTGCGTTTTTTGGTTGATCGCTTTGTTGCATGGAGGGAGACGGGTGCATGCG
kir21_Frag165-330_insertion225 CGTCTGTGACGTCTCCTAATGTGCAGTTCATAAACGTGGGGGAAAAGGGTCAACGGTACTTGGCCGATATTTTCACAACCAGCGGGAGACCGGGGTCTCTGAGCTGCGTTGATATCCGCTGGCGCTGGATGTTGGTTATTTTTTGTCTCGCCTTCGTACTGTCTTGGCTCTTCTTTGGCTGCGTTTTTTGGTTGATCGCTTTGTTGCATGGAGGGAGACGGGTGCATGCG
kir21_Frag165-330_insertion228 CGTCTGTGACGTCTCCTAATGTGCAGTTCATAAACGTGGGGGAAAAGGGTCAACGGTACTTGGCCGATATTTTCACAACCTGCAGCGGGAGACCGGGGTCTCTGAGCGTTGATATCCGCTGGCGCTGGATGTTGGTTATTTTTTGTCTCGCCTTCGTACTGTCTTGGCTCTTCTTTGGCTGCGTTTTTTGGTTGATCGCTTTGTTGCATGGAGGGAGACGGGTGCATGCG
kir21_Frag165-330_insertion231 CGTCTGTGACGTCTCCTAATGTGCAGTTCATAAACGTGGGGGAAAAGGGTCAACGGTACTTGGCCGATATTTTCACAACCTGCGTTAGCGGGAGACCGGGGTCTCTGAGCGATATCCGCTGGCGCTGGATGTTGGTTATTTTTTGTCTCGCCTTCGTACTGTCTTGGCTCTTCTTTGGCTGCGTTTTTTGGTTGATCGCTTTGTTGCATGGAGGGAGACGGGTGCATGCG
kir21_Frag165-330_insertion234 CGTCTGTGACGTCTCCTAATGTGCAGTTCATAAACGTGGGGGAAAAGGGTCAACGGTACTTGGCCGATATTTTCACAACCTGCGTTGATAGCGGGAGACCGGGGTCTCTGAGCATCCGCTGGCGCTGGATGTTGGTTATTTTTTGTCTCGCCTTCGTACTGTCTTGGCTCTTCTTTGGCTGCGTTTTTTGGTTGATCGCTTTGTTGCATGGAGGGAGACGGGTGCATGCG
kir21_Frag165-330_insertion237 CGTCTGTGACGTCTCCTAATGTGCAGTTCATAAACGTGGGGGAAAAGGGTCAACGGTACTTGGCCGATATTTTCACAACCTGCGTTGATATCAGCGGGAGACCGGGGTCTCTGAGCCGCTGGCGCTGGATGTTGGTTATTTTTTGTCTCGCCTTCGTACTGTCTTGGCTCTTCTTTGGCTGCGTTTTTTGGTTGATCGCTTTGTTGCATGGAGGGAGACGGGTGCATGCG
kir21_Frag165-330_insertion240 CGTCTGTGACGTCTCCTAATGTGCAGTTCATAAACGTGGGGGAAAAGGGTCAACGGTACTTGGCCGATATTTTCACAACCTGCGTTGATATCCGCAGCGGGAGACCGGGGTCTCTGAGCTGGCGCTGGATGTTGGTTATTTTTTGTCTCGCCTTCGTACTGTCTTGGCTCTTCTTTGGCTGCGTTTTTTGGTTGATCGCTTTGTTGCATGGAGGGAGACGGGTGCATGCG
kir21_Frag165-330_insertion243 CGTCTGTGACGTCTCCTAATGTGCAGTTCATAAACGTGGGGGAAAAGGGTCAACGGTACTTGGCCGATATTTTCACAACCTGCGTTGATATCCGCTGGAGCGGGAGACCGGGGTCTCTGAGCCGCTGGATGTTGGTTATTTTTTGTCTCGCCTTCGTACTGTCTTGGCTCTTCTTTGGCTGCGTTTTTTGGTTGATCGCTTTGTTGCATGGAGGGAGACGGGTGCATGCG
kir21_Frag165-330_insertion246 CGTCTGTGACGTCTCCTAATGTGCAGTTCATAAACGTGGGGGAAAAGGGTCAACGGTACTTGGCCGATATTTTCACAACCTGCGTTGATATCCGCTGGCGCAGCGGGAGACCGGGGTCTCTGAGCTGGATGTTGGTTATTTTTTGTCTCGCCTTCGTACTGTCTTGGCTCTTCTTTGGCTGCGTTTTTTGGTTGATCGCTTTGTTGCATGGAGGGAGACGGGTGCATGCG
kir21_Frag165-330_insertion249 CGTCTGTGACGTCTCCTAATGTGCAGTTCATAAACGTGGGGGAAAAGGGTCAACGGTACTTGGCCGATATTTTCACAACCTGCGTTGATATCCGCTGGCGCTGGAGCGGGAGACCGGGGTCTCTGAGCATGTTGGTTATTTTTTGTCTCGCCTTCGTACTGTCTTGGCTCTTCTTTGGCTGCGTTTTTTGGTTGATCGCTTTGTTGCATGGAGGGAGACGGGTGCATGCG
kir21_Frag165-330_insertion252 CGTCTGTGACGTCTCCTAATGTGCAGTTCATAAACGTGGGGGAAAAGGGTCAACGGTACTTGGCCGATATTTTCACAACCTGCGTTGATATCCGCTGGCGCTGGATGAGCGGGAGACCGGGGTCTCTGAGCTTGGTTATTTTTTGTCTCGCCTTCGTACTGTCTTGGCTCTTCTTTGGCTGCGTTTTTTGGTTGATCGCTTTGTTGCATGGAGGGAGACGGGTGCATGCG
kir21_Frag165-330_insertion255 CGTCTGTGACGTCTCCTAATGTGCAGTTCATAAACGTGGGGGAAAAGGGTCAACGGTACTTGGCCGATATTTTCACAACCTGCGTTGATATCCGCTGGCGCTGGATGTTGAGCGGGAGACCGGGGTCTCTGAGCGTTATTTTTTGTCTCGCCTTCGTACTGTCTTGGCTCTTCTTTGGCTGCGTTTTTTGGTTGATCGCTTTGTTGCATGGAGGGAGACGGGTGCATGCG
kir21_Frag165-330_insertion258 CGTCTGTGACGTCTCCTAATGTGCAGTTCATAAACGTGGGGGAAAAGGGTCAACGGTACTTGGCCGATATTTTCACAACCTGCGTTGATATCCGCTGGCGCTGGATGTTGGTTAGCGGGAGACCGGGGTCTCTGAGCATTTTTTGTCTCGCCTTCGTACTGTCTTGGCTCTTCTTTGGCTGCGTTTTTTGGTTGATCGCTTTGTTGCATGGAGGGAGACGGGTGCATGCG
kir21_Frag165-330_insertion261 CGTCTGTGACGTCTCCTAATGTGCAGTTCATAAACGTGGGGGAAAAGGGTCAACGGTACTTGGCCGATATTTTCACAACCTGCGTTGATATCCGCTGGCGCTGGATGTTGGTTATTAGCGGGAGACCGGGGTCTCTGAGCTTTTGTCTCGCCTTCGTACTGTCTTGGCTCTTCTTTGGCTGCGTTTTTTGGTTGATCGCTTTGTTGCATGGAGGGAGACGGGTGCATGCG
kir21_Frag165-330_insertion264 CGTCTGTGACGTCTCCTAATGTGCAGTTCATAAACGTGGGGGAAAAGGGTCAACGGTACTTGGCCGATATTTTCACAACCTGCGTTGATATCCGCTGGCGCTGGATGTTGGTTATTTTTAGCGGGAGACCGGGGTCTCTGAGCTGTCTCGCCTTCGTACTGTCTTGGCTCTTCTTTGGCTGCGTTTTTTGGTTGATCGCTTTGTTGCATGGAGGGAGACGGGTGCATGCG
kir21_Frag165-330_insertion267 CGTCTGTGACGTCTCCTAATGTGCAGTTCATAAACGTGGGGGAAAAGGGTCAACGGTACTTGGCCGATATTTTCACAACCTGCGTTGATATCCGCTGGCGCTGGATGTTGGTTATTTTTTGTAGCGGGAGACCGGGGTCTCTGAGCCTCGCCTTCGTACTGTCTTGGCTCTTCTTTGGCTGCGTTTTTTGGTTGATCGCTTTGTTGCATGGAGGGAGACGGGTGCATGCG
kir21_Frag165-330_insertion270 CGTCTGTGACGTCTCCTAATGTGCAGTTCATAAACGTGGGGGAAAAGGGTCAACGGTACTTGGCCGATATTTTCACAACCTGCGTTGATATCCGCTGGCGCTGGATGTTGGTTATTTTTTGTCTCAGCGGGAGACCGGGGTCTCTGAGCGCCTTCGTACTGTCTTGGCTCTTCTTTGGCTGCGTTTTTTGGTTGATCGCTTTGTTGCATGGAGGGAGACGGGTGCATGCG

kir21_Frag165-330_insertion273	CGTCTGTGACGTCTCCTAATGTGCAGTTCATAAACGTGGGGGAAAAGGGTCAACGGTACTTGGCCGATATTTTCACAACCTGCGTTGATATCCGCTGGCGCTGGATGTTGGTTATTTTTTGTCTCGCCAGCGGGAGACCGGGGTCTCTGAGCTTCGTACTGTCTTGGCTCTTCTTTGGCTGCGTTTTTTGGTTGATCGCTTTGTTGCATGGAGGGAGACGGGTGCATGCG
kir21_Frag165-330_insertion276	CGTCTGTGACGTCTCCTAATGTGCAGTTCATAAACGTGGGGGAAAAGGGTCAACGGTACTTGGCCGATATTTTCACAACCTGCGTTGATATCCGCTGGCGCTGGATGTTGGTTATTTTTTGTCTCGCCTTCAGCGGGAGACCGGGGTCTCTGAGCGTACTGTCTTGGCTCTTCTTTGGCTGCGTTTTTTGGTTGATCGCTTTGTTGCATGGAGGGAGACGGGTGCATGCG
kir21_Frag165-330_insertion279	CGTCTGTGACGTCTCCTAATGTGCAGTTCATAAACGTGGGGGAAAAGGGTCAACGGTACTTGGCCGATATTTTCACAACCTGCGTTGATATCCGCTGGCGCTGGATGTTGGTTATTTTTTGTCTCGCCTTCGTAAGCGGGAGACCGGGGTCTCTGAGCCTGTCTTGGCTCTTCTTTGGCTGCGTTTTTTGGTTGATCGCTTTGTTGCATGGAGGGAGACGGGTGCATGCG
kir21_Frag165-330_insertion282	CGTCTGTGACGTCTCCTAATGTGCAGTTCATAAACGTGGGGGAAAAGGGTCAACGGTACTTGGCCGATATTTTCACAACCTGCGTTGATATCCGCTGGCGCTGGATGTTGGTTATTTTTTGTCTCGCCTTCGTACTGAGCGGGAGACCGGGGTCTCTGAGCTCTTGGCTCTTCTTTGGCTGCGTTTTTTGGTTGATCGCTTTGTTGCATGGAGGGAGACGGGTGCATGCG
kir21_Frag165-330_insertion285	CGTCTGTGACGTCTCCTAATGTGCAGTTCATAAACGTGGGGGAAAAGGGTCAACGGTACTTGGCCGATATTTTCACAACCTGCGTTGATATCCGCTGGCGCTGGATGTTGGTTATTTTTTGTCTCGCCTTCGTACTGTCTAGCGGGAGACCGGGGTCTCTGAGCTGGCTCTTCTTTGGCTGCGTTTTTTGGTTGATCGCTTTGTTGCATGGAGGGAGACGGGTGCATGCG
kir21_Frag165-330_insertion288	CGTCTGTGACGTCTCCTAATGTGCAGTTCATAAACGTGGGGGAAAAGGGTCAACGGTACTTGGCCGATATTTTCACAACCTGCGTTGATATCCGCTGGCGCTGGATGTTGGTTATTTTTTGTCTCGCCTTCGTACTGTCTTGGAGCGGGAGACCGGGGTCTCTGAGCCTCTTCTTTGGCTGCGTTTTTTGGTTGATCGCTTTGTTGCATGGAGGGAGACGGGTGCATGCG
kir21_Frag165-330_insertion291	CGTCTGTGACGTCTCCTAATGTGCAGTTCATAAACGTGGGGGAAAAGGGTCAACGGTACTTGGCCGATATTTTCACAACCTGCGTTGATATCCGCTGGCGCTGGATGTTGGTTATTTTTTGTCTCGCCTTCGTACTGTCTTGGCTCAGCGGGAGACCGGGGTCTCTGAGCTTCTTTGGCTGCGTTTTTTGGTTGATCGCTTTGTTGCATGGAGGGAGACGGGTGCATGCG
kir21_Frag165-330_insertion294	CGTCTGTGACGTCTCCTAATGTGCAGTTCATAAACGTGGGGGAAAAGGGTCAACGGTACTTGGCCGATATTTTCACAACCTGCGTTGATATCCGCTGGCGCTGGATGTTGGTTATTTTTTGTCTCGCCTTCGTACTGTCTTGGCTCTTCAGCGGGAGACCGGGGTCTCTGAGCTTTGGCTGCGTTTTTTGGTTGATCGCTTTGTTGCATGGAGGGAGACGGGTGCATGCG
kir21_Frag165-330_insertion297	CGTCTGTGACGTCTCCTAATGTGCAGTTCATAAACGTGGGGGAAAAGGGTCAACGGTACTTGGCCGATATTTTCACAACCTGCGTTGATATCCGCTGGCGCTGGATGTTGGTTATTTTTTGTCTCGCCTTCGTACTGTCTTGGCTCTTCTTTAGCGGGAGACCGGGGTCTCTGAGCGGCTGCGTTTTTTGGTTGATCGCTTTGTTGCATGGAGGGAGACGGGTGCATGCG
kir21_Frag165-330_insertion300	CGTCTGTGACGTCTCCTAATGTGCAGTTCATAAACGTGGGGGAAAAGGGTCAACGGTACTTGGCCGATATTTTCACAACCTGCGTTGATATCCGCTGGCGCTGGATGTTGGTTATTTTTTGTCTCGCCTTCGTACTGTCTTGGCTCTTCTTTGGCAGCGGGAGACCGGGGTCTCTGAGCTGCGTTTTTTGGTTGATCGCTTTGTTGCATGGAGGGAGACGGGTGCATGCG
kir21_Frag165-330_insertion303	CGTCTGTGACGTCTCCTAATGTGCAGTTCATAAACGTGGGGGAAAAGGGTCAACGGTACTTGGCCGATATTTTCACAACCTGCGTTGATATCCGCTGGCGCTGGATGTTGGTTATTTTTTGTCTCGCCTTCGTACTGTCTTGGCTCTTCTTTGGCTGCAGCGGGAGACCGGGGTCTCTGAGCGTTTTTTGGTTGATCGCTTTGTTGCATGGAGGGAGACGGGTGCATGCG
kir21_Frag165-330_insertion306	CGTCTGTGACGTCTCCTAATGTGCAGTTCATAAACGTGGGGGAAAAGGGTCAACGGTACTTGGCCGATATTTTCACAACCTGCGTTGATATCCGCTGGCGCTGGATGTTGGTTATTTTTTGTCTCGCCTTCGTACTGTCTTGGCTCTTCTTTGGCTGCGTTAGCGGGAGACCGGGGTCTCTGAGCTTTTGGTTGATCGCTTTGTTGCATGGAGGGAGACGGGTGCATGCG
kir21_Frag165-330_insertion309	CGTCTGTGACGTCTCCTAATGTGCAGTTCATAAACGTGGGGGAAAAGGGTCAACGGTACTTGGCCGATATTTTCACAACCTGCGTTGATATCCGCTGGCGCTGGATGTTGGTTATTTTTTGTCTCGCCTTCGTACTGTCTTGGCTCTTCTTTGGCTGCGTTTTTAGCGGGAGACCGGGGTCTCTGAGCTGGTTGATCGCTTTGTTGCATGGAGGGAGACGGGTGCATGCG
kir21_Frag165-330_insertion312	CGTCTGTGACGTCTCCTAATGTGCAGTTCATAAACGTGGGGGAAAAGGGTCAACGGTACTTGGCCGATATTTTCACAACCTGCGTTGATATCCGCTGGCGCTGGATGTTGGTTATTTTTTGTCTCGCCTTCGTACTGTCTTGGCTCTTCTTTGGCTGCGTTTTTTGGAGCGGGAGACCGGGGTCTCTGAGCTTGATCGCTTTGTTGCATGGAGGGAGACGGGTGCATGCG
kir21_Frag165-330_insertion315	CGTCTGTGACGTCTCCTAATGTGCAGTTCATAAACGTGGGGGAAAAGGGTCAACGGTACTTGGCCGATATTTTCACAACCTGCGTTGATATCCGCTGGCGCTGGATGTTGGTTATTTTTTGTCTCGCCTTCGTACTGTCTTGGCTCTTCTTTGGCTGCGTTTTTTGGTTGAGCGGGAGACCGGGGTCTCTGAGCATCGCTTTGTTGCATGGAGGGAGACGGGTGCATGCG
kir21_Frag165-330_insertion318	CGTCTGTGACGTCTCCTAATGTGCAGTTCATAAACGTGGGGGAAAAGGGTCAACGGTACTTGGCCGATATTTTCACAACCTGCGTTGATATCCGCTGGCGCTGGATGTTGGTTATTTTTTGTCTCGCCTTCGTACTGTCTTGGCTCTTCTTTGGCTGCGTTTTTTGGTTGATCAGCGGGAGACCGGGGTCTCTGAGCGCTTTGTTGCATGGAGGGAGACGGGTGCATGCG
kir21_Frag165-330_insertion321	CGTCTGTGACGTCTCCTAATGTGCAGTTCATAAACGTGGGGGAAAAGGGTCAACGGTACTTGGCCGATATTTTCACAACCTGCGTTGATATCCGCTGGCGCTGGATGTTGGTTATTTTTTGTCTCGCCTTCGTACTGTCTTGGCTCTTCTTTGGCTGCGTTTTTTGGTTGATCGCTAGCGGGAGACCGGGGTCTCTGAGCTTGTTGCATGGAGGGAGACGGGTGCATGCG
kir21_Frag165-330_insertion324	CGTCTGTGACGTCTCCTAATGTGCAGTTCATAAACGTGGGGGAAAAGGGTCAACGGTACTTGGCCGATATTTTCACAACCTGCGTTGATATCCGCTGGCGCTGGATGTTGGTTATTTTTTGTCTCGCCTTCGTACTGTCTTGGCTCTTCTTTGGCTGCGTTTTTTGGTTGATCGCTTTGAGCGGGAGACCGGGGTCTCTGAGCTTGCATGGAGGGAGACGGGTGCATGCG
kir21_Frag165-330_insertion327	CGTCTGTGACGTCTCCTAATGTGCAGTTCATAAACGTGGGGGAAAAGGGTCAACGGTACTTGGCCGATATTTTCACAACCTGCGTTGATATCCGCTGGCGCTGGATGTTGGTTATTTTTTGTCTCGCCTTCGTACTGTCTTGGCTCTTCTTTGGCTGCGTTTTTTGGTTGATCGCTTTGTTGAGCGGGAGACCGGGGTCTCTGAGCCATGGAGGGAGACGGGTGCATGCG
kir21_Frag165-330_insertion330	CGTCTGTGACGTCTCCTAATGTGCAGTTCATAAACGTGGGGGAAAAGGGTCAACGGTACTTGGCCGATATTTTCACAACCTGCGTTGATATCCGCTGGCGCTGGATGTTGGTTATTTTTTGTCTCGCCTTCGTACTGTCTTGGCTCTTCTTTGGCTGCGTTTTTTGGTTGATCGCTTTGTTGCATAGCGGGAGACCGGGGTCTCTGAGCGGAGGGAGACGGGTGCATGCG
kir21_Frag333-495_insertion333	GCGTATTAGAACGTCTCCTGGAAGCGGGAGACCGGGGTCTCTGAGCGATTTGGACACCGATTATAAAGATGATGATGATAAATCCAAGGTATCCAAGGCCTGTGTCTCCGAAGTAAATTCCTTTACCGCAGCTTTCCTTTTCTCTATCGAAACACAAACCACTATCGGATACGGGTTCCGATGCGTCACAGACGAATGCCCAATAGCCGTTTGGAGACGGATGGTGAGAG
kir21_Frag333-495_insertion336	GCGTATTAGAACGTCTCCTGGAGATAGCGGGAGACCGGGGTCTCTGAGCTTGGACACCGATTATAAAGATGATGATGATAAATCCAAGGTATCCAAGGCCTGTGTCTCCGAAGTAAATTCCTTTACCGCAGCTTTCCTTTTCTCTATCGAAACACAAACCACTATCGGATACGGGTTCCGATGCGTCACAGACGAATGCCCAATAGCCGTTTGGAGACGGATGGTGAGAG
kir21_Frag333-495_insertion339	GCGTATTAGAACGTCTCCTGGAGATTTGAGCGGGAGACCGGGGTCTCTGAGCGACACCGATTATAAAGATGATGATGATAAATCCAAGGTATCCAAGGCCTGTGTCTCCGAAGTAAATTCCTTTACCGCAGCTTTCCTTTTCTCTATCGAAACACAAACCACTATCGGATACGGGTTCCGATGCGTCACAGACGAATGCCCAATAGCCGTTTGGAGACGGATGGTGAGAG
kir21_Frag333-495_insertion342	GCGTATTAGAACGTCTCCTGGAGATTTGGACAGCGGGAGACCGGGGTCTCTGAGCACCGATTATAAAGATGATGATGATAAATCCAAGGTATCCAAGGCCTGTGTCTCCGAAGTAAATTCCTTTACCGCAGCTTTCCTTTTCTCTATCGAAACACAAACCACTATCGGATACGGGTTCCGATGCGTCACAGACGAATGCCCAATAGCCGTTTGGAGACGGATGGTGAGAG
kir21_Frag333-495_insertion345	GCGTATTAGAACGTCTCCTGGAGATTTGGACACCAGCGGGAGACCGGGGTCTCTGAGCGATTATAAAGATGATGATGATAAATCCAAGGTATCCAAGGCCTGTGTCTCCGAAGTAAATTCCTTTACCGCAGCTTTCCTTTTCTCTATCGAAACACAAACCACTATCGGATACGGGTTCCGATGCGTCACAGACGAATGCCCAATAGCCGTTTGGAGACGGATGGTGAGAG
kir21_Frag333-495_insertion348	GCGTATTAGAACGTCTCCTGGAGATTTGGACACCGATAGCGGGAGACCGGGGTCTCTGAGCTATAAAGATGATGATGATAAATCCAAGGTATCCAAGGCCTGTGTCTCCGAAGTAAATTCCTTTACCGCAGCTTTCCTTTTCTCTATCGAAACACAAACCACTATCGGATACGGGTTCCGATGCGTCACAGACGAATGCCCAATAGCCGTTTGGAGACGGATGGTGAGAG
kir21_Frag333-495_insertion351	GCGTATTAGAACGTCTCCTGGAGATTTGGACACCGATTATAGCGGGAGACCGGGGTCTCTGAGCAAAGATGATGATGATAAATCCAAGGTATCCAAGGCCTGTGTCTCCGAAGTAAATTCCTTTACCGCAGCTTTCCTTTTCTCTATCGAAACACAAACCACTATCGGATACGGGTTCCGATGCGTCACAGACGAATGCCCAATAGCCGTTTGGAGACGGATGGTGAGAG
kir21_Frag333-495_insertion354	GCGTATTAGAACGTCTCCTGGAGATTTGGACACCGATTATAAAAGCGGGAGACCGGGGTCTCTGAGCGATGATGATAAATCCAAGGTATCCAAGGCCTGTGTCTCCGAAGTAAATTCCTTTACCGCAGCTTTCCTTTTCTCTATCGAAACACAAACCACTATCGGATACGGGTTCCGATGCGTCACAGACGAATGCCCAATAGCCGTTTGGAGACGGATGGTGAGAG
kir21_Frag333-495_insertion357	GCGTATTAGAACGTCTCCTGGAGATTTGGACACCGATTATAAAGATAGCGGGAGACCGGGGTCTCTGAGCGATGATGATAAATCCAAGGTATCCAAGGCCTGTGTCTCCGAAGTAAATTCCTTTACCGCAGCTTTCCTTTTCTCTATCGAAACACAAACCACTATCGGATACGGGTTCCGATGCGTCACAGACGAATGCCCAATAGCCGTTTGGAGACGGATGGTGAGAG
kir21_Frag333-495_insertion360	GCGTATTAGAACGTCTCCTGGAGATTTGGACACCGATTATAAAGATGATAGCGGGAGACCGGGGTCTCTGAGCGATGATAAATCCAAGGTATCCAAGGCCTGTGTCTCCGAAGTAAATTCCTTTACCGCAGCTTTCCTTTTCTCTATCGAAACACAAACCACTATCGGATACGGGTTCCGATGCGTCACAGACGAATGCCCAATAGCCGTTTGGAGACGGATGGTGAGAG
kir21_Frag333-495_insertion363	GCGTATTAGAACGTCTCCTGGAGATTTGGACACCGATTATAAAGATGATGATAGCGGGAGACCGGGGTCTCTGAGCGATAAATCCAAGGTATCCAAGGCCTGTGTCTCCGAAGTAAATTCCTTTACCGCAGCTTTCCTTTTCTCTATCGAAACACAAACCACTATCGGATACGGGTTCCGATGCGTCACAGACGAATGCCCAATAGCCGTTTGGAGACGGATGGTGAGAG
kir21_Frag333-495_insertion366	GCGTATTAGAACGTCTCCTGGAGATTTGGACACCGATTATAAAGATGATGATGATAGCGGGAGACCGGGGTCTCTGAGCAAATCCAAGGTATCCAAGGCCTGTGTCTCCGAAGTAAATTCCTTTACCGCAGCTTTCCTTTTCTCTATCGAAACACAAACCACTATCGGATACGGGTTCCGATGCGTCACAGACGAATGCCCAATAGCCGTTTGGAGACGGATGGTGAGAG
kir21_Frag333-495_insertion369	GCGTATTAGAACGTCTCCTGGAGATTTGGACACCGATTATAAAGATGATGATGATAAAAGCGGGAGACCGGGGTCTCTGAGCTCCAAGGTATCCAAGGCCTGTGTCTCCGAAGTAAATTCCTTTACCGCAGCTTTCCTTTTCTCTATCGAAACACAAACCACTATCGGATACGGGTTCCGATGCGTCACAGACGAATGCCCAATAGCCGTTTGGAGACGGATGGTGAGAG
kir21_Frag333-495_insertion372	GCGTATTAGAACGTCTCCTGGAGATTTGGACACCGATTATAAAGATGATGATGATAAATCCAGCGGGAGACCGGGGTCTCTGAGCAAGGTATCCAAGGCCTGTGTCTCCGAAGTAAATTCCTTTACCGCAGCTTTCCTTTTCTCTATCGAAACACAAACCACTATCGGATACGGGTTCCGATGCGTCACAGACGAATGCCCAATAGCCGTTTGGAGACGGATGGTGAGAG
kir21_Frag333-495_insertion375	GCGTATTAGAACGTCTCCTGGAGATTTGGACACCGATTATAAAGATGATGATGATAAATCCAAGAGCGGGAGACCGGGGTCTCTGAGCGTATCCAAGGCCTGTGTCTCCGAAGTAAATTCCTTTACCGCAGCTTTCCTTTTCTCTATCGAAACACAAACCACTATCGGATACGGGTTCCGATGCGTCACAGACGAATGCCCAATAGCCGTTTGGAGACGGATGGTGAGAG
kir21_Frag333-495_insertion378	GCGTATTAGAACGTCTCCTGGAGATTTGGACACCGATTATAAAGATGATGATGATAAATCCAAGGTAAGCGGGAGACCGGGGTCTCTGAGCTCCAAGGCCTGTGTCTCCGAAGTAAATTCCTTTACCGCAGCTTTCCTTTTCTCTATCGAAACACAAACCACTATCGGATACGGGTTCCGATGCGTCACAGACGAATGCCCAATAGCCGTTTGGAGACGGATGGTGAGAG
kir21_Frag333-495_insertion381	GCGTATTAGAACGTCTCCTGGAGATTTGGACACCGATTATAAAGATGATGATGATAAATCCAAGGTATCCAGCGGGAGACCGGGGTCTCTGAGCAAGGCCTGTGTCTCCGAAGTAAATTCCTTTACCGCAGCTTTCCTTTTCTCTATCGAAACACAAACCACTATCGGATACGGGTTCCGATGCGTCACAGACGAATGCCCAATAGCCGTTTGGAGACGGATGGTGAGAG
kir21_Frag333-495_insertion384	GCGTATTAGAACGTCTCCTGGAGATTTGGACACCGATTATAAAGATGATGATGATAAATCCAAGGTATCCAAGAGCGGGAGACCGGGGTCTCTGAGCGCCTGTGTCTCCGAAGTAAATTCCTTTACCGCAGCTTTCCTTTTCTCTATCGAAACACAAACCACTATCGGATACGGGTTCCGATGCGTCACAGACGAATGCCCAATAGCCGTTTGGAGACGGATGGTGAGAG
kir21_Frag333-495_insertion387	GCGTATTAGAACGTCTCCTGGAGATTTGGACACCGATTATAAAGATGATGATGATAAATCCAAGGTATCCAAGGCCAGCGGGAGACCGGGGTCTCTGAGCTGTGTCTCCGAAGTAAATTCCTTTACCGCAGCTTTCCTTTTCTCTATCGAAACACAAACCACTATCGGATACGGGTTCCGATGCGTCACAGACGAATGCCCAATAGCCGTTTGGAGACGGATGGTGAGAG
kir21_Frag333-495_insertion390	GCGTATTAGAACGTCTCCTGGAGATTTGGACACCGATTATAAAGATGATGATGATAAATCCAAGGTATCCAAGGCCTGTAGCGGGAGACCGGGGTCTCTGAGCGTCTCCGAAGTAAATTCCTTTACCGCAGCTTTCCTTTTCTCTATCGAAACACAAACCACTATCGGATACGGGTTCCGATGCGTCACAGACGAATGCCCAATAGCCGTTTGGAGACGGATGGTGAGAG
kir21_Frag333-495_insertion393	GCGTATTAGAACGTCTCCTGGAGATTTGGACACCGATTATAAAGATGATGATGATAAATCCAAGGTATCCAAGGCCTGTGTCAGCGGGAGACCGGGGTCTCTGAGCTCCGAAGTAAATTCCTTTACCGCAGCTTTCCTTTTCTCTATCGAAACACAAACCACTATCGGATACGGGTTCCGATGCGTCACAGACGAATGCCCAATAGCCGTTTGGAGACGGATGGTGAGAG
kir21_Frag333-495_insertion396	GCGTATTAGAACGTCTCCTGGAGATTTGGACACCGATTATAAAGATGATGATGATAAATCCAAGGTATCCAAGGCCTGTGTCTCCAGCGGGAGACCGGGGTCTCTGAGCGAAGTAAATTCCTTTACCGCAGCTTTCCTTTTCTCTATCGAAACACAAACCACTATCGGATACGGGTTCCGATGCGTCACAGACGAATGCCCAATAGCCGTTTGGAGACGGATGGTGAGAG
kir21_Frag333-495_insertion399	GCGTATTAGAACGTCTCCTGGAGATTTGGACACCGATTATAAAGATGATGATGATAAATCCAAGGTATCCAAGGCCTGTGTCTCCGAAAGCGGGAGACCGGGGTCTCTGAGCGTAAATTCCTTTACCGCAGCTTTCCTTTTCTCTATCGAAACACAAACCACTATCGGATACGGGTTCCGATGCGTCACAGACGAATGCCCAATAGCCGTTTGGAGACGGATGGTGAGAG
kir21_Frag333-495_insertion402	GCGTATTAGAACGTCTCCTGGAGATTTGGACACCGATTATAAAGATGATGATGATAAATCCAAGGTATCCAAGGCCTGTGTCTCCGAAGTAAGCGGGAGACCGGGGTCTCTGAGCAATTCCTTTACCGCAGCTTTCCTTTTCTCTATCGAAACACAAACCACTATCGGATACGGGTTCCGATGCGTCACAGACGAATGCCCAATAGCCGTTTGGAGACGGATGGTGAGAG
kir21_Frag333-495_insertion405	GCGTATTAGAACGTCTCCTGGAGATTTGGACACCGATTATAAAGATGATGATGATAAATCCAAGGTATCCAAGGCCTGTGTCTCCGAAGTAAATAGCGGGAGACCGGGGTCTCTGAGCTCCTTTACCGCAGCTTTCCTTTTCTCTATCGAAACACAAACCACTATCGGATACGGGTTCCGATGCGTCACAGACGAATGCCCAATAGCCGTTTGGAGACGGATGGTGAGAG
kir21_Frag333-495_insertion408	GCGTATTAGAACGTCTCCTGGAGATTTGGACACCGATTATAAAGATGATGATGATAAATCCAAGGTATCCAAGGCCTGTGTCTCCGAAGTAAATTCCAGCGGGAGACCGGGGTCTCTGAGCTTTACCGCAGCTTTCCTTTTCTCTATCGAAACACAAACCACTATCGGATACGGGTTCCGATGCGTCACAGACGAATGCCCAATAGCCGTTTGGAGACGGATGGTGAGAG
kir21_Frag333-495_insertion411	GCGTATTAGAACGTCTCCTGGAGATTTGGACACCGATTATAAAGATGATGATGATAAATCCAAGGTATCCAAGGCCTGTGTCTCCGAAGTAAATTCCTTTAGCGGGAGACCGGGGTCTCTGAGCACCGCAGCTTTCCTTTTCTCTATCGAAACACAAACCACTATCGGATACGGGTTCCGATGCGTCACAGACGAATGCCCAATAGCCGTTTGGAGACGGATGGTGAGAG
kir21_Frag333-495_insertion414	GCGTATTAGAACGTCTCCTGGAGATTTGGACACCGATTATAAAGATGATGATGATAAATCCAAGGTATCCAAGGCCTGTGTCTCCGAAGTAAATTCCTTTACCAGCGGGAGACCGGGGTCTCTGAGCGCAGCTTTCCTTTTCTCTATCGAAACACAAACCACTATCGGATACGGGTTCCGATGCGTCACAGACGAATGCCCAATAGCCGTTTGGAGACGGATGGTGAGAG
kir21_Frag333-495_insertion417	GCGTATTAGAACGTCTCCTGGAGATTTGGACACCGATTATAAAGATGATGATGATAAATCCAAGGTATCCAAGGCCTGTGTCTCCGAAGTAAATTCCTTTACCGCAAGCGGGAGACCGGGGTCTCTGAGCGCTTTCCTTTTCTCTATCGAAACACAAACCACTATCGGATACGGGTTCCGATGCGTCACAGACGAATGCCCAATAGCCGTTTGGAGACGGATGGTGAGAG
kir21_Frag333-495_insertion420	GCGTATTAGAACGTCTCCTGGAGATTTGGACACCGATTATAAAGATGATGATGATAAATCCAAGGTATCCAAGGCCTGTGTCTCCGAAGTAAATTCCTTTACCGCAGCTAGCGGGAGACCGGGGTCTCTGAGCTTCCTTTTCTCTATCGAAACACAAACCACTATCGGATACGGGTTCCGATGCGTCACAGACGAATGCCCAATAGCCGTTTGGAGACGGATGGTGAGAG
kir21_Frag333-495_insertion423	GCGTATTAGAACGTCTCCTGGAGATTTGGACACCGATTATAAAGATGATGATGATAAATCCAAGGTATCCAAGGCCTGTGTCTCCGAAGTAAATTCCTTTACCGCAGCTTTCAGCGGGAGACCGGGGTCTCTGAGCCTTTTCTCTATCGAAACACAAACCACTATCGGATACGGGTTCCGATGCGTCACAGACGAATGCCCAATAGCCGTTTGGAGACGGATGGTGAGAG
kir21_Frag333-495_insertion426	GCGTATTAGAACGTCTCCTGGAGATTTGGACACCGATTATAAAGATGATGATGATAAATCCAAGGTATCCAAGGCCTGTGTCTCCGAAGTAAATTCCTTTACCGCAGCTTTCCTTAGCGGGAGACCGGGGTCTCTGAGCTTCTCTATCGAAACACAAACCACTATCGGATACGGGTTCCGATGCGTCACAGACGAATGCCCAATAGCCGTTTGGAGACGGATGGTGAGAG
kir21_Frag333-495_insertion429	GCGTATTAGAACGTCTCCTGGAGATTTGGACACCGATTATAAAGATGATGATGATAAATCCAAGGTATCCAAGGCCTGTGTCTCCGAAGTAAATTCCTTTACCGCAGCTTTCCTTTTCAGCGGGAGACCGGGGTCTCTGAGCTCTATCGAAACACAAACCACTATCGGATACGGGTTCCGATGCGTCACAGACGAATGCCCAATAGCCGTTTGGAGACGGATGGTGAGAG
kir21_Frag333-495_insertion432	GCGTATTAGAACGTCTCCTGGAGATTTGGACACCGATTATAAAGATGATGATGATAAATCCAAGGTATCCAAGGCCTGTGTCTCCGAAGTAAATTCCTTTACCGCAGCTTTCCTTTTCTCTAGCGGGAGACCGGGGTCTCTGAGCATCGAAACACAAACCACTATCGGATACGGGTTCCGATGCGTCACAGACGAATGCCCAATAGCCGTTTGGAGACGGATGGTGAGAG
kir21_Frag333-495_insertion435	GCGTATTAGAACGTCTCCTGGAGATTTGGACACCGATTATAAAGATGATGATGATAAATCCAAGGTATCCAAGGCCTGTGTCTCCGAAGTAAATTCCTTTACCGCAGCTTTCCTTTTCTCTATCAGCGGGAGACCGGGGTCTCTGAGCGAAACACAAACCACTATCGGATACGGGTTCCGATGCGTCACAGACGAATGCCCAATAGCCGTTTGGAGACGGATGGTGAGAG
kir21_Frag333-495_insertion438	GCGTATTAGAACGTCTCCTGGAGATTTGGACACCGATTATAAAGATGATGATGATAAATCCAAGGTATCCAAGGCCTGTGTCTCCGAAGTAAATTCCTTTACCGCAGCTTTCCTTTTCTCTATCGAAAGCGGGAGACCGGGGTCTCTGAGCACACAAACCACTATCGGATACGGGTTCCGATGCGTCACAGACGAATGCCCAATAGCCGTTTGGAGACGGATGGTGAGAG
kir21_Frag333-495_insertion441	GCGTATTAGAACGTCTCCTGGAGATTTGGACACCGATTATAAAGATGATGATGATAAATCCAAGGTATCCAAGGCCTGTGTCTCCGAAGTAAATTCCTTTACCGCAGCTTTCCTTTTCTCTATCGAAACAAGCGGGAGACCGGGGTCTCTGAGCCAAACCACTATCGGATACGGGTTCCGATGCGTCACAGACGAATGCCCAATAGCCGTTTGGAGACGGATGGTGAGAG
kir21_Frag333-495_insertion444	GCGTATTAGAACGTCTCCTGGAGATTTGGACACCGATTATAAAGATGATGATGATAAATCCAAGGTATCCAAGGCCTGTGTCTCCGAAGTAAATTCCTTTACCGCAGCTTTCCTTTTCTCTATCGAAACACAAAGCGGGAGACCGGGGTCTCTGAGCACCACTATCGGATACGGGTTCCGATGCGTCACAGACGAATGCCCAATAGCCGTTTGGAGACGGATGGTGAGAG
kir21_Frag333-495_insertion447	GCGTATTAGAACGTCTCCTGGAGATTTGGACACCGATTATAAAGATGATGATGATAAATCCAAGGTATCCAAGGCCTGTGTCTCCGAAGTAAATTCCTTTACCGCAGCTTTCCTTTTCTCTATCGAAACACAAACCAGCGGGAGACCGGGGTCTCTGAGCACTATCGGATACGGGTTCCGATGCGTCACAGACGAATGCCCAATAGCCGTTTGGAGACGGATGGTGAGAG
kir21_Frag333-495_insertion450	GCGTATTAGAACGTCTCCTGGAGATTTGGACACCGATTATAAAGATGATGATGATAAATCCAAGGTATCCAAGGCCTGTGTCTCCGAAGTAAATTCCTTTACCGCAGCTTTCCTTTTCTCTATCGAAACACAAACCACTAGCGGGAGACCGGGGTCTCTGAGCATCGGATACGGGTTCCGATGCGTCACAGACGAATGCCCAATAGCCGTTTGGAGACGGATGGTGAGAG
kir21_Frag333-495_insertion453	GCGTATTAGAACGTCTCCTGGAGATTTGGACACCGATTATAAAGATGATGATGATAAATCCAAGGTATCCAAGGCCTGTGTCTCCGAAGTAAATTCCTTTACCGCAGCTTTCCTTTTCTCTATCGAAACACAAACCACTATCAGCGGGAGACCGGGGTCTCTGAGCGATACGGGTTCCGATGCGTCACAGACGAATGCCCAATAGCCGTTTGGAGACGGATGGTGAGAG
kir21_Frag333-495_insertion456	GCGTATTAGAACGTCTCCTGGAGATTTGGACACCGATTATAAAGATGATGATGATAAATCCAAGGTATCCAAGGCCTGTGTCTCCGAAGTAAATTCCTTTACCGCAGCTTTCCTTTTCTCTATCGAAACACAAACCACTATCGGAAGCGGGAGACCGGGGTCTCTGAGCTACGGGTTCCGATGCGTCACAGACGAATGCCCAATAGCCGTTTGGAGACGGATGGTGAGAG
kir21_Frag333-495_insertion459	GCGTATTAGAACGTCTCCTGGAGATTTGGACACCGATTATAAAGATGATGATGATAAATCCAAGGTATCCAAGGCCTGTGTCTCCGAAGTAAATTCCTTTACCGCAGCTTTCCTTTTCTCTATCGAAACACAAACCACTATCGGATACAGCGGGAGACCGGGGTCTCTGAGCGGGTTCCGATGCGTCACAGACGAATGCCCAATAGCCGTTTGGAGACGGATGGTGAGAG
kir21_Frag333-495_insertion462	GCGTATTAGAACGTCTCCTGGAGATTTGGACACCGATTATAAAGATGATGATGATAAATCCAAGGTATCCAAGGCCTGTGTCTCCGAAGTAAATTCCTTTACCGCAGCTTTCCTTTTCTCTATCGAAACACAAACCACTATCGGATACGGGAGCGGGAGACCGGGGTCTCTGAGCTTCCGATGCGTCACAGACGAATGCCCAATAGCCGTTTGGAGACGGATGGTGAGAG
kir21_Frag333-495_insertion465	GCGTATTAGAACGTCTCCTGGAGATTTGGACACCGATTATAAAGATGATGATGATAAATCCAAGGTATCCAAGGCCTGTGTCTCCGAAGTAAATTCCTTTACCGCAGCTTTCCTTTTCTCTATCGAAACACAAACCACTATCGGATACGGGTTCAGCGGGAGACCGGGGTCTCTGAGCGATGCGTCACAGACGAATGCCCAATAGCCGTTTGGAGACGGATGGTGAGAG
kir21_Frag333-495_insertion468	GCGTATTAGAACGTCTCCTGGAGATTTGGACACCGATTATAAAGATGATGATGATAAATCCAAGGTATCCAAGGCCTGTGTCTCCGAAGTAAATTCCTTTACCGCAGCTTTCCTTTTCTCTATCGAAACACAAACCACTATCGGATACGGGTTCCGAAGCGGGAGACCGGGGTCTCTGAGCTGCGTCACAGACGAATGCCCAATAGCCGTTTGGAGACGGATGGTGAGAG
kir21_Frag333-495_insertion471	GCGTATTAGAACGTCTCCTGGAGATTTGGACACCGATTATAAAGATGATGATGATAAATCCAAGGTATCCAAGGCCTGTGTCTCCGAAGTAAATTCCTTTACCGCAGCTTTCCTTTTCTCTATCGAAACACAAACCACTATCGGATACGGGTTCCGATGCAGCGGGAGACCGGGGTCTCTGAGCGTCACAGACGAATGCCCAATAGCCGTTTGGAGACGGATGGTGAGAG
kir21_Frag333-495_insertion474	GCGTATTAGAACGTCTCCTGGAGATTTGGACACCGATTATAAAGATGATGATGATAAATCCAAGGTATCCAAGGCCTGTGTCTCCGAAGTAAATTCCTTTACCGCAGCTTTCCTTTTCTCTATCGAAACACAAACCACTATCGGATACGGGTTCCGATGCGTCAGCGGGAGACCGGGGTCTCTGAGCACAGACGAATGCCCAATAGCCGTTTGGAGACGGATGGTGAGAG
kir21_Frag333-495_insertion477	GCGTATTAGAACGTCTCCTGGAGATTTGGACACCGATTATAAAGATGATGATGATAAATCCAAGGTATCCAAGGCCTGTGTCTCCGAAGTAAATTCCTTTACCGCAGCTTTCCTTTTCTCTATCGAAACACAAACCACTATCGGATACGGGTTCCGATGCGTCACAAGCGGGAGACCGGGGTCTCTGAGCGACGAATGCCCAATAGCCGTTTGGAGACGGATGGTGAGAG
kir21_Frag333-495_insertion480	GCGTATTAGAACGTCTCCTGGAGATTTGGACACCGATTATAAAGATGATGATGATAAATCCAAGGTATCCAAGGCCTGTGTCTCCGAAGTAAATTCCTTTACCGCAGCTTTCCTTTTCTCTATCGAAACACAAACCACTATCGGATACGGGTTCCGATGCGTCACAGACAGCGGGAGACCGGGGTCTCTGAGCGAATGCCCAATAGCCGTTTGGAGACGGATGGTGAGAG
kir21_Frag333-495_insertion483	GCGTATTAGAACGTCTCCTGGAGATTTGGACACCGATTATAAAGATGATGATGATAAATCCAAGGTATCCAAGGCCTGTGTCTCCGAAGTAAATTCCTTTACCGCAGCTTTCCTTTTCTCTATCGAAACACAAACCACTATCGGATACGGGTTCCGATGCGTCACAGACGAAAGCGGGAGACCGGGGTCTCTGAGCTGCCCAATAGCCGTTTGGAGACGGATGGTGAGAG
kir21_Frag333-495_insertion486	GCGTATTAGAACGTCTCCTGGAGATTTGGACACCGATTATAAAGATGATGATGATAAATCCAAGGTATCCAAGGCCTGTGTCTCCGAAGTAAATTCCTTTACCGCAGCTTTCCTTTTCTCTATCGAAACACAAACCACTATCGGATACGGGTTCCGATGCGTCACAGACGAATGCAGCGGGAGACCGGGGTCTCTGAGCCCAATAGCCGTTTGGAGACGGATGGTGAGAG
kir21_Frag333-495_insertion489	GCGTATTAGAACGTCTCCTGGAGATTTGGACACCGATTATAAAGATGATGATGATAAATCCAAGGTATCCAAGGCCTGTGTCTCCGAAGTAAATTCCTTTACCGCAGCTTTCCTTTTCTCTATCGAAACACAAACCACTATCGGATACGGGTTCCGATGCGTCACAGACGAATGCCCAAGCGGGAGACCGGGGTCTCTGAGCATAGCCGTTTGGAGACGGATGGTGAGAG
kir21_Frag333-495_insertion492	GCGTATTAGAACGTCTCCTGGAGATTTGGACACCGATTATAAAGATGATGATGATAAATCCAAGGTATCCAAGGCCTGTGTCTCCGAAGTAAATTCCTTTACCGCAGCTTTCCTTTTCTCTATCGAAACACAAACCACTATCGGATACGGGTTCCGATGCGTCACAGACGAATGCCCAATAAGCGGGAGACCGGGGTCTCTGAGCGCCGTTTGGAGACGGATGGTGAGAG
kir21_Frag333-495_insertion495	GCGTATTAGAACGTCTCCTGGAGATTTGGACACCGATTATAAAGATGATGATGATAAATCCAAGGTATCCAAGGCCTGTGTCTCCGAAGTAAATTCCTTTACCGCAGCTTTCCTTTTCTCTATCGAAACACAAACCACTATCGGATACGGGTTCCGATGCGTCACAGACGAATGCCCAATAGCCAGCGGGAGACCGGGGTCTCTGAGCGTTTGGAGACGGATGGTGAGAG
kir21_Frag498-660_insertion498	GCTGAGAGGTACGTCTCCCGTTAGCGGGAGACCGGGGTCTCTGAGCTTCATGGTTGTCTTTCAATCAATAGTGGGCTGTATTATCGATGCATTTATCATTGGGGCCGTGATGGCAAAAATGGCTAAGCCCAAAAAAAGAAATGAGACATTGGTTTTCAGTCACAACGCTGTGATTGCCATGAGGGATGGCAAGCTGTGCCTCATGTGGAGGGGGAGACGAGTGCGACACA
kir21_Frag498-660_insertion501	GCTGAGAGGTACGTCTCCCGTTTTCAGCGGGAGACCGGGGTCTCTGAGCATGGTTGTCTTTCAATCAATAGTGGGCTGTATTATCGATGCATTTATCATTGGGGCCGTGATGGCAAAAATGGCTAAGCCCAAAAAAAGAAATGAGACATTGGTTTTCAGTCACAACGCTGTGATTGCCATGAGGGATGGCAAGCTGTGCCTCATGTGGAGGGGGAGACGAGTGCGACACA
kir21_Frag498-660_insertion504	GCTGAGAGGTACGTCTCCCGTTTTCATGAGCGGGAGACCGGGGTCTCTGAGCGTTGTCTTTCAATCAATAGTGGGCTGTATTATCGATGCATTTATCATTGGGGCCGTGATGGCAAAAATGGCTAAGCCCAAAAAAAGAAATGAGACATTGGTTTTCAGTCACAACGCTGTGATTGCCATGAGGGATGGCAAGCTGTGCCTCATGTGGAGGGGGAGACGAGTGCGACACA
kir21_Frag498-660_insertion507	GCTGAGAGGTACGTCTCCCGTTTTCATGGTTAGCGGGAGACCGGGGTCTCTGAGCGTCTTTCAATCAATAGTGGGCTGTATTATCGATGCATTTATCATTGGGGCCGTGATGGCAAAAATGGCTAAGCCCAAAAAAAGAAATGAGACATTGGTTTTCAGTCACAACGCTGTGATTGCCATGAGGGATGGCAAGCTGTGCCTCATGTGGAGGGGGAGACGAGTGCGACACA
kir21_Frag498-660_insertion510	GCTGAGAGGTACGTCTCCCGTTTTCATGGTTGTCAGCGGGAGACCGGGGTCTCTGAGCTTTCAATCAATAGTGGGCTGTATTATCGATGCATTTATCATTGGGGCCGTGATGGCAAAAATGGCTAAGCCCAAAAAAAGAAATGAGACATTGGTTTTCAGTCACAACGCTGTGATTGCCATGAGGGATGGCAAGCTGTGCCTCATGTGGAGGGGGAGACGAGTGCGACACA
kir21_Frag498-660_insertion513	GCTGAGAGGTACGTCTCCCGTTTTCATGGTTGTCTTTAGCGGGAGACCGGGGTCTCTGAGCCAATCAATAGTGGGCTGTATTATCGATGCATTTATCATTGGGGCCGTGATGGCAAAAATGGCTAAGCCCAAAAAAAGAAATGAGACATTGGTTTTCAGTCACAACGCTGTGATTGCCATGAGGGATGGCAAGCTGTGCCTCATGTGGAGGGGGAGACGAGTGCGACACA
kir21_Frag498-660_insertion516	GCTGAGAGGTACGTCTCCCGTTTTCATGGTTGTCTTTCAAAGCGGGAGACCGGGGTCTCTGAGCTCAATAGTGGGCTGTATTATCGATGCATTTATCATTGGGGCCGTGATGGCAAAAATGGCTAAGCCCAAAAAAAGAAATGAGACATTGGTTTTCAGTCACAACGCTGTGATTGCCATGAGGGATGGCAAGCTGTGCCTCATGTGGAGGGGGAGACGAGTGCGACACA
kir21_Frag498-660_insertion519	GCTGAGAGGTACGTCTCCCGTTTTCATGGTTGTCTTTCAATCAAGCGGGAGACCGGGGTCTCTGAGCATAGTGGGCTGTATTATCGATGCATTTATCATTGGGGCCGTGATGGCAAAAATGGCTAAGCCCAAAAAAAGAAATGAGACATTGGTTTTCAGTCACAACGCTGTGATTGCCATGAGGGATGGCAAGCTGTGCCTCATGTGGAGGGGGAGACGAGTGCGACACA
kir21_Frag498-660_insertion522	GCTGAGAGGTACGTCTCCCGTTTTCATGGTTGTCTTTCAATCAATAAGCGGGAGACCGGGGTCTCTGAGCGTGGGCTGTATTATCGATGCATTTATCATTGGGGCCGTGATGGCAAAAATGGCTAAGCCCAAAAAAAGAAATGAGACATTGGTTTTCAGTCACAACGCTGTGATTGCCATGAGGGATGGCAAGCTGTGCCTCATGTGGAGGGGGAGACGAGTGCGACACA
kir21_Frag498-660_insertion525	GCTGAGAGGTACGTCTCCCGTTTTCATGGTTGTCTTTCAATCAATAGTGAGCGGGAGACCGGGGTCTCTGAGCGGCTGTATTATCGATGCATTTATCATTGGGGCCGTGATGGCAAAAATGGCTAAGCCCAAAAAAAGAAATGAGACATTGGTTTTCAGTCACAACGCTGTGATTGCCATGAGGGATGGCAAGCTGTGCCTCATGTGGAGGGGGAGACGAGTGCGACACA
kir21_Frag498-660_insertion528	GCTGAGAGGTACGTCTCCCGTTTTCATGGTTGTCTTTCAATCAATAGTGGGCAGCGGGAGACCGGGGTCTCTGAGCTGTATTATCGATGCATTTATCATTGGGGCCGTGATGGCAAAAATGGCTAAGCCCAAAAAAAGAAATGAGACATTGGTTTTCAGTCACAACGCTGTGATTGCCATGAGGGATGGCAAGCTGTGCCTCATGTGGAGGGGGAGACGAGTGCGACACA
kir21_Frag498-660_insertion531	GCTGAGAGGTACGTCTCCCGTTTTCATGGTTGTCTTTCAATCAATAGTGGGCTGTAGCGGGAGACCGGGGTCTCTGAGCATTATCGATGCATTTATCATTGGGGCCGTGATGGCAAAAATGGCTAAGCCCAAAAAAAGAAATGAGACATTGGTTTTCAGTCACAACGCTGTGATTGCCATGAGGGATGGCAAGCTGTGCCTCATGTGGAGGGGGAGACGAGTGCGACACA
kir21_Frag498-660_insertion534	GCTGAGAGGTACGTCTCCCGTTTTCATGGTTGTCTTTCAATCAATAGTGGGCTGTATTAGCGGGAGACCGGGGTCTCTGAGCATCGATGCATTTATCATTGGGGCCGTGATGGCAAAAATGGCTAAGCCCAAAAAAAGAAATGAGACATTGGTTTTCAGTCACAACGCTGTGATTGCCATGAGGGATGGCAAGCTGTGCCTCATGTGGAGGGGGAGACGAGTGCGACACA
kir21_Frag498-660_insertion537	GCTGAGAGGTACGTCTCCCGTTTTCATGGTTGTCTTTCAATCAATAGTGGGCTGTATTATCAGCGGGAGACCGGGGTCTCTGAGCGATGCATTTATCATTGGGGCCGTGATGGCAAAAATGGCTAAGCCCAAAAAAAGAAATGAGACATTGGTTTTCAGTCACAACGCTGTGATTGCCATGAGGGATGGCAAGCTGTGCCTCATGTGGAGGGGGAGACGAGTGCGACACA
kir21_Frag498-660_insertion540	GCTGAGAGGTACGTCTCCCGTTTTCATGGTTGTCTTTCAATCAATAGTGGGCTGTATTATCGATAGCGGGAGACCGGGGTCTCTGAGCGCATTTATCATTGGGGCCGTGATGGCAAAAATGGCTAAGCCCAAAAAAAGAAATGAGACATTGGTTTTCAGTCACAACGCTGTGATTGCCATGAGGGATGGCAAGCTGTGCCTCATGTGGAGGGGGAGACGAGTGCGACACA
kir21_Frag498-660_insertion543	GCTGAGAGGTACGTCTCCCGTTTTCATGGTTGTCTTTCAATCAATAGTGGGCTGTATTATCGATGCAAGCGGGAGACCGGGGTCTCTGAGCTTTATCATTGGGGCCGTGATGGCAAAAATGGCTAAGCCCAAAAAAAGAAATGAGACATTGGTTTTCAGTCACAACGCTGTGATTGCCATGAGGGATGGCAAGCTGTGCCTCATGTGGAGGGGGAGACGAGTGCGACACA
kir21_Frag498-660_insertion546	GCTGAGAGGTACGTCTCCCGTTTTCATGGTTGTCTTTCAATCAATAGTGGGCTGTATTATCGATGCATTTAGCGGGAGACCGGGGTCTCTGAGCATCATTGGGGCCGTGATGGCAAAAATGGCTAAGCCCAAAAAAAGAAATGAGACATTGGTTTTCAGTCACAACGCTGTGATTGCCATGAGGGATGGCAAGCTGTGCCTCATGTGGAGGGGGAGACGAGTGCGACACA
kir21_Frag498-660_insertion549	GCTGAGAGGTACGTCTCCCGTTTTCATGGTTGTCTTTCAATCAATAGTGGGCTGTATTATCGATGCATTTATCAGCGGGAGACCGGGGTCTCTGAGCATTGGGGCCGTGATGGCAAAAATGGCTAAGCCCAAAAAAAGAAATGAGACATTGGTTTTCAGTCACAACGCTGTGATTGCCATGAGGGATGGCAAGCTGTGCCTCATGTGGAGGGGGAGACGAGTGCGACACA
kir21_Frag498-660_insertion552	GCTGAGAGGTACGTCTCCCGTTTTCATGGTTGTCTTTCAATCAATAGTGGGCTGTATTATCGATGCATTTATCATTAGCGGGAGACCGGGGTCTCTGAGCGGGGCCGTGATGGCAAAAATGGCTAAGCCCAAAAAAAGAAATGAGACATTGGTTTTCAGTCACAACGCTGTGATTGCCATGAGGGATGGCAAGCTGTGCCTCATGTGGAGGGGGAGACGAGTGCGACACA
kir21_Frag498-660_insertion555	GCTGAGAGGTACGTCTCCCGTTTTCATGGTTGTCTTTCAATCAATAGTGGGCTGTATTATCGATGCATTTATCATTGGGAGCGGGAGACCGGGGTCTCTGAGCGCCGTGATGGCAAAAATGGCTAAGCCCAAAAAAAGAAATGAGACATTGGTTTTCAGTCACAACGCTGTGATTGCCATGAGGGATGGCAAGCTGTGCCTCATGTGGAGGGGGAGACGAGTGCGACACA
kir21_Frag498-660_insertion558	GCTGAGAGGTACGTCTCCCGTTTTCATGGTTGTCTTTCAATCAATAGTGGGCTGTATTATCGATGCATTTATCATTGGGGCCAGCGGGAGACCGGGGTCTCTGAGCGTGATGGCAAAAATGGCTAAGCCCAAAAAAAGAAATGAGACATTGGTTTTCAGTCACAACGCTGTGATTGCCATGAGGGATGGCAAGCTGTGCCTCATGTGGAGGGGGAGACGAGTGCGACACA
kir21_Frag498-660_insertion561	GCTGAGAGGTACGTCTCCCGTTTTCATGGTTGTCTTTCAATCAATAGTGGGCTGTATTATCGATGCATTTATCATTGGGGCCGTGAGCGGGAGACCGGGGTCTCTGAGCATGGCAAAAATGGCTAAGCCCAAAAAAAGAAATGAGACATTGGTTTTCAGTCACAACGCTGTGATTGCCATGAGGGATGGCAAGCTGTGCCTCATGTGGAGGGGGAGACGAGTGCGACACA
kir21_Frag498-660_insertion564	GCTGAGAGGTACGTCTCCCGTTTTCATGGTTGTCTTTCAATCAATAGTGGGCTGTATTATCGATGCATTTATCATTGGGGCCGTGATGAGCGGGAGACCGGGGTCTCTGAGCGCAAAAATGGCTAAGCCCAAAAAAAGAAATGAGACATTGGTTTTCAGTCACAACGCTGTGATTGCCATGAGGGATGGCAAGCTGTGCCTCATGTGGAGGGGGAGACGAGTGCGACACA
kir21_Frag498-660_insertion567	GCTGAGAGGTACGTCTCCCGTTTTCATGGTTGTCTTTCAATCAATAGTGGGCTGTATTATCGATGCATTTATCATTGGGGCCGTGATGGCAAGCGGGAGACCGGGGTCTCTGAGCAAAATGGCTAAGCCCAAAAAAAGAAATGAGACATTGGTTTTCAGTCACAACGCTGTGATTGCCATGAGGGATGGCAAGCTGTGCCTCATGTGGAGGGGGAGACGAGTGCGACACA
kir21_Frag498-660_insertion570	GCTGAGAGGTACGTCTCCCGTTTTCATGGTTGTCTTTCAATCAATAGTGGGCTGTATTATCGATGCATTTATCATTGGGGCCGTGATGGCAAAAAGCGGGAGACCGGGGTCTCTGAGCATGGCTAAGCCCAAAAAAAGAAATGAGACATTGGTTTTCAGTCACAACGCTGTGATTGCCATGAGGGATGGCAAGCTGTGCCTCATGTGGAGGGGGAGACGAGTGCGACACA
kir21_Frag498-660_insertion573	GCTGAGAGGTACGTCTCCCGTTTTCATGGTTGTCTTTCAATCAATAGTGGGCTGTATTATCGATGCATTTATCATTGGGGCCGTGATGGCAAAAATGAGCGGGAGACCGGGGTCTCTGAGCGCTAAGCCCAAAAAAAGAAATGAGACATTGGTTTTCAGTCACAACGCTGTGATTGCCATGAGGGATGGCAAGCTGTGCCTCATGTGGAGGGGGAGACGAGTGCGACACA
kir21_Frag498-660_insertion576	GCTGAGAGGTACGTCTCCCGTTTTCATGGTTGTCTTTCAATCAATAGTGGGCTGTATTATCGATGCATTTATCATTGGGGCCGTGATGGCAAAAATGGCTAGCGGGAGACCGGGGTCTCTGAGCAAGCCCAAAAAAAGAAATGAGACATTGGTTTTCAGTCACAACGCTGTGATTGCCATGAGGGATGGCAAGCTGTGCCTCATGTGGAGGGGGAGACGAGTGCGACACA
kir21_Frag498-660_insertion579	GCTGAGAGGTACGTCTCCCGTTTTCATGGTTGTCTTTCAATCAATAGTGGGCTGTATTATCGATGCATTTATCATTGGGGCCGTGATGGCAAAAATGGCTAAGAGCGGGAGACCGGGGTCTCTGAGCCCCAAAAAAAGAAATGAGACATTGGTTTTCAGTCACAACGCTGTGATTGCCATGAGGGATGGCAAGCTGTGCCTCATGTGGAGGGGGAGACGAGTGCGACACA
kir21_Frag498-660_insertion582	GCTGAGAGGTACGTCTCCCGTTTTCATGGTTGTCTTTCAATCAATAGTGGGCTGTATTATCGATGCATTTATCATTGGGGCCGTGATGGCAAAAATGGCTAAGCCCAGCGGGAGACCGGGGTCTCTGAGCAAAAAAAGAAATGAGACATTGGTTTTCAGTCACAACGCTGTGATTGCCATGAGGGATGGCAAGCTGTGCCTCATGTGGAGGGGGAGACGAGTGCGACACA
kir21_Frag498-660_insertion585	GCTGAGAGGTACGTCTCCCGTTTTCATGGTTGTCTTTCAATCAATAGTGGGCTGTATTATCGATGCATTTATCATTGGGGCCGTGATGGCAAAAATGGCTAAGCCCAAAAGCGGGAGACCGGGGTCTCTGAGCAAAGAAATGAGACATTGGTTTTCAGTCACAACGCTGTGATTGCCATGAGGGATGGCAAGCTGTGCCTCATGTGGAGGGGGAGACGAGTGCGACACA
kir21_Frag498-660_insertion588	GCTGAGAGGTACGTCTCCCGTTTTCATGGTTGTCTTTCAATCAATAGTGGGCTGTATTATCGATGCATTTATCATTGGGGCCGTGATGGCAAAAATGGCTAAGCCCAAAAAAAGCGGGAGACCGGGGTCTCTGAGCAGAATGAGACATTGGTTTTCAGTCACAACGCTGTGATTGCCATGAGGGATGGCAAGCTGTGCCTCATGTGGAGGGGGAGACGAGTGCGACACA
kir21_Frag498-660_insertion591	GCTGAGAGGTACGTCTCCCGTTTTCATGGTTGTCTTTCAATCAATAGTGGGCTGTATTATCGATGCATTTATCATTGGGGCCGTGATGGCAAAAATGGCTAAGCCCAAAAAAAGAAGCGGGAGACCGGGGTCTCTGAGCAATGAGACATTGGTTTTCAGTCACAACGCTGTGATTGCCATGAGGGATGGCAAGCTGTGCCTCATGTGGAGGGGGAGACGAGTGCGACACA
kir21_Frag498-660_insertion594	GCTGAGAGGTACGTCTCCCGTTTTCATGGTTGTCTTTCAATCAATAGTGGGCTGTATTATCGATGCATTTATCATTGGGGCCGTGATGGCAAAAATGGCTAAGCCCAAAAAAAGAAATAGCGGGAGACCGGGGTCTCTGAGCGAGACATTGGTTTTCAGTCACAACGCTGTGATTGCCATGAGGGATGGCAAGCTGTGCCTCATGTGGAGGGGGAGACGAGTGCGACACA
kir21_Frag498-660_insertion597	GCTGAGAGGTACGTCTCCCGTTTTCATGGTTGTCTTTCAATCAATAGTGGGCTGTATTATCGATGCATTTATCATTGGGGCCGTGATGGCAAAAATGGCTAAGCCCAAAAAAAGAAATGAGAGCGGGAGACCGGGGTCTCTGAGCACATTGGTTTTCAGTCACAACGCTGTGATTGCCATGAGGGATGGCAAGCTGTGCCTCATGTGGAGGGGGAGACGAGTGCGACACA
kir21_Frag498-660_insertion600	GCTGAGAGGTACGTCTCCCGTTTTCATGGTTGTCTTTCAATCAATAGTGGGCTGTATTATCGATGCATTTATCATTGGGGCCGTGATGGCAAAAATGGCTAAGCCCAAAAAAAGAAATGAGACAAGCGGGAGACCGGGGTCTCTGAGCTTGGTTTTCAGTCACAACGCTGTGATTGCCATGAGGGATGGCAAGCTGTGCCTCATGTGGAGGGGGAGACGAGTGCGACACA
kir21_Frag498-660_insertion603	GCTGAGAGGTACGTCTCCCGTTTTCATGGTTGTCTTTCAATCAATAGTGGGCTGTATTATCGATGCATTTATCATTGGGGCCGTGATGGCAAAAATGGCTAAGCCCAAAAAAAGAAATGAGACATTGAGCGGGAGACCGGGGTCTCTGAGCGTTTTCAGTCACAACGCTGTGATTGCCATGAGGGATGGCAAGCTGTGCCTCATGTGGAGGGGGAGACGAGTGCGACACA
kir21_Frag498-660_insertion606	GCTGAGAGGTACGTCTCCCGTTTTCATGGTTGTCTTTCAATCAATAGTGGGCTGTATTATCGATGCATTTATCATTGGGGCCGTGATGGCAAAAATGGCTAAGCCCAAAAAAAGAAATGAGACATTGGTTAGCGGGAGACCGGGGTCTCTGAGCTTCAGTCACAACGCTGTGATTGCCATGAGGGATGGCAAGCTGTGCCTCATGTGGAGGGGGAGACGAGTGCGACACA
kir21_Frag498-660_insertion609	GCTGAGAGGTACGTCTCCCGTTTTCATGGTTGTCTTTCAATCAATAGTGGGCTGTATTATCGATGCATTTATCATTGGGGCCGTGATGGCAAAAATGGCTAAGCCCAAAAAAAGAAATGAGACATTGGTTTTCAGCGGGAGACCGGGGTCTCTGAGCAGTCACAACGCTGTGATTGCCATGAGGGATGGCAAGCTGTGCCTCATGTGGAGGGGGAGACGAGTGCGACACA
kir21_Frag498-660_insertion612	GCTGAGAGGTACGTCTCCCGTTTTCATGGTTGTCTTTCAATCAATAGTGGGCTGTATTATCGATGCATTTATCATTGGGGCCGTGATGGCAAAAATGGCTAAGCCCAAAAAAAGAAATGAGACATTGGTTTTCAGTAGCGGGAGACCGGGGTCTCTGAGCCACAACGCTGTGATTGCCATGAGGGATGGCAAGCTGTGCCTCATGTGGAGGGGGAGACGAGTGCGACACA
kir21_Frag498-660_insertion615	GCTGAGAGGTACGTCTCCCGTTTTCATGGTTGTCTTTCAATCAATAGTGGGCTGTATTATCGATGCATTTATCATTGGGGCCGTGATGGCAAAAATGGCTAAGCCCAAAAAAAGAAATGAGACATTGGTTTTCAGTCACAGCGGGAGACCGGGGTCTCTGAGCAACGCTGTGATTGCCATGAGGGATGGCAAGCTGTGCCTCATGTGGAGGGGGAGACGAGTGCGACACA
kir21_Frag498-660_insertion618	GCTGAGAGGTACGTCTCCCGTTTTCATGGTTGTCTTTCAATCAATAGTGGGCTGTATTATCGATGCATTTATCATTGGGGCCGTGATGGCAAAAATGGCTAAGCCCAAAAAAAGAAATGAGACATTGGTTTTCAGTCACAACAGCGGGAGACCGGGGTCTCTGAGCGCTGTGATTGCCATGAGGGATGGCAAGCTGTGCCTCATGTGGAGGGGGAGACGAGTGCGACACA
kir21_Frag498-660_insertion621	GCTGAGAGGTACGTCTCCCGTTTTCATGGTTGTCTTTCAATCAATAGTGGGCTGTATTATCGATGCATTTATCATTGGGGCCGTGATGGCAAAAATGGCTAAGCCCAAAAAAAGAAATGAGACATTGGTTTTCAGTCACAACGCTAGCGGGAGACCGGGGTCTCTGAGCGTGATTGCCATGAGGGATGGCAAGCTGTGCCTCATGTGGAGGGGGAGACGAGTGCGACACA
kir21_Frag498-660_insertion624	GCTGAGAGGTACGTCTCCCGTTTTCATGGTTGTCTTTCAATCAATAGTGGGCTGTATTATCGATGCATTTATCATTGGGGCCGTGATGGCAAAAATGGCTAAGCCCAAAAAAAGAAATGAGACATTGGTTTTCAGTCACAACGCTGTGAGCGGGAGACCGGGGTCTCTGAGCATTGCCATGAGGGATGGCAAGCTGTGCCTCATGTGGAGGGGGAGACGAGTGCGACACA
kir21_Frag498-660_insertion627	GCTGAGAGGTACGTCTCCCGTTTTCATGGTTGTCTTTCAATCAATAGTGGGCTGTATTATCGATGCATTTATCATTGGGGCCGTGATGGCAAAAATGGCTAAGCCCAAAAAAAGAAATGAGACATTGGTTTTCAGTCACAACGCTGTGATTAGCGGGAGACCGGGGTCTCTGAGCGCCATGAGGGATGGCAAGCTGTGCCTCATGTGGAGGGGGAGACGAGTGCGACACA
kir21_Frag498-660_insertion630	GCTGAGAGGTACGTCTCCCGTTTTCATGGTTGTCTTTCAATCAATAGTGGGCTGTATTATCGATGCATTTATCATTGGGGCCGTGATGGCAAAAATGGCTAAGCCCAAAAAAAGAAATGAGACATTGGTTTTCAGTCACAACGCTGTGATTGCCAGCGGGAGACCGGGGTCTCTGAGCATGAGGGATGGCAAGCTGTGCCTCATGTGGAGGGGGAGACGAGTGCGACACA
kir21_Frag498-660_insertion633	GCTGAGAGGTACGTCTCCCGTTTTCATGGTTGTCTTTCAATCAATAGTGGGCTGTATTATCGATGCATTTATCATTGGGGCCGTGATGGCAAAAATGGCTAAGCCCAAAAAAAGAAATGAGACATTGGTTTTCAGTCACAACGCTGTGATTGCCATGAGCGGGAGACCGGGGTCTCTGAGCAGGGATGGCAAGCTGTGCCTCATGTGGAGGGGGAGACGAGTGCGACACA
kir21_Frag498-660_insertion636	GCTGAGAGGTACGTCTCCCGTTTTCATGGTTGTCTTTCAATCAATAGTGGGCTGTATTATCGATGCATTTATCATTGGGGCCGTGATGGCAAAAATGGCTAAGCCCAAAAAAAGAAATGAGACATTGGTTTTCAGTCACAACGCTGTGATTGCCATGAGGAGCGGGAGACCGGGGTCTCTGAGCGATGGCAAGCTGTGCCTCATGTGGAGGGGGAGACGAGTGCGACACA
kir21_Frag498-660_insertion639	GCTGAGAGGTACGTCTCCCGTTTTCATGGTTGTCTTTCAATCAATAGTGGGCTGTATTATCGATGCATTTATCATTGGGGCCGTGATGGCAAAAATGGCTAAGCCCAAAAAAAGAAATGAGACATTGGTTTTCAGTCACAACGCTGTGATTGCCATGAGGGATAGCGGGAGACCGGGGTCTCTGAGCGGCAAGCTGTGCCTCATGTGGAGGGGGAGACGAGTGCGACACA
kir21_Frag498-660_insertion642	GCTGAGAGGTACGTCTCCCGTTTTCATGGTTGTCTTTCAATCAATAGTGGGCTGTATTATCGATGCATTTATCATTGGGGCCGTGATGGCAAAAATGGCTAAGCCCAAAAAAAGAAATGAGACATTGGTTTTCAGTCACAACGCTGTGATTGCCATGAGGGATGGCAGCGGGAGACCGGGGTCTCTGAGCAAGCTGTGCCTCATGTGGAGGGGGAGACGAGTGCGACACA
kir21_Frag498-660_insertion645	GCTGAGAGGTACGTCTCCCGTTTTCATGGTTGTCTTTCAATCAATAGTGGGCTGTATTATCGATGCATTTATCATTGGGGCCGTGATGGCAAAAATGGCTAAGCCCAAAAAAAGAAATGAGACATTGGTTTTCAGTCACAACGCTGTGATTGCCATGAGGGATGGCAAGAGCGGGAGACCGGGGTCTCTGAGCCTGTGCCTCATGTGGAGGGGGAGACGAGTGCGACACA
kir21_Frag498-660_insertion648	GCTGAGAGGTACGTCTCCCGTTTTCATGGTTGTCTTTCAATCAATAGTGGGCTGTATTATCGATGCATTTATCATTGGGGCCGTGATGGCAAAAATGGCTAAGCCCAAAAAAAGAAATGAGACATTGGTTTTCAGTCACAACGCTGTGATTGCCATGAGGGATGGCAAGCTGAGCGGGAGACCGGGGTCTCTGAGCTGCCTCATGTGGAGGGGGAGACGAGTGCGACACA
kir21_Frag498-660_insertion651	GCTGAGAGGTACGTCTCCCGTTTTCATGGTTGTCTTTCAATCAATAGTGGGCTGTATTATCGATGCATTTATCATTGGGGCCGTGATGGCAAAAATGGCTAAGCCCAAAAAAAGAAATGAGACATTGGTTTTCAGTCACAACGCTGTGATTGCCATGAGGGATGGCAAGCTGTGCAGCGGGAGACCGGGGTCTCTGAGCCTCATGTGGAGGGGGAGACGAGTGCGACACA
kir21_Frag498-660_insertion654	GCTGAGAGGTACGTCTCCCGTTTTCATGGTTGTCTTTCAATCAATAGTGGGCTGTATTATCGATGCATTTATCATTGGGGCCGTGATGGCAAAAATGGCTAAGCCCAAAAAAAGAAATGAGACATTGGTTTTCAGTCACAACGCTGTGATTGCCATGAGGGATGGCAAGCTGTGCCTCAGCGGGAGACCGGGGTCTCTGAGCATGTGGAGGGGGAGACGAGTGCGACACA
kir21_Frag498-660_insertion657	GCTGAGAGGTACGTCTCCCGTTTTCATGGTTGTCTTTCAATCAATAGTGGGCTGTATTATCGATGCATTTATCATTGGGGCCGTGATGGCAAAAATGGCTAAGCCCAAAAAAAGAAATGAGACATTGGTTTTCAGTCACAACGCTGTGATTGCCATGAGGGATGGCAAGCTGTGCCTCATGAGCGGGAGACCGGGGTCTCTGAGCTGGAGGGGGAGACGAGTGCGACACA
kir21_Frag498-660_insertion660	GCTGAGAGGTACGTCTCCCGTTTTCATGGTTGTCTTTCAATCAATAGTGGGCTGTATTATCGATGCATTTATCATTGGGGCCGTGATGGCAAAAATGGCTAAGCCCAAAAAAAGAAATGAGACATTGGTTTTCAGTCACAACGCTGTGATTGCCATGAGGGATGGCAAGCTGTGCCTCATGTGGAGCGGGAGACCGGGGTCTCTGAGCAGGGGGAGACGAGTGCGACACA
kir21_Frag663-822_insertion663	TTTGGCTATTCCCGTCTCCGAGGAGCGGGAGACCGGGGTCTCTGAGCGTGGGCAATCTGAGAAAGTCCCACCTCGTAGAGGCCCATGTACGAGCACAACTGCTGAAATCACGCATAACTTCAGAAGGAGAGTACATACCACTCGATCAGATTGATATCAATGTGGGCTTCGATAGCGGCATTGACAGGATCTTTCTCGTTAGCCCAATCAGGAGACGTGCGACTCGAAAG
kir21_Frag663-822_insertion666	TTTGGCTATTCCCGTCTCCGAGGGTGAGCGGGAGACCGGGGTCTCTGAGCGGCAATCTGAGAAAGTCCCACCTCGTAGAGGCCCATGTACGAGCACAACTGCTGAAATCACGCATAACTTCAGAAGGAGAGTACATACCACTCGATCAGATTGATATCAATGTGGGCTTCGATAGCGGCATTGACAGGATCTTTCTCGTTAGCCCAATCAGGAGACGTGCGACTCGAAAG
kir21_Frag663-822_insertion669	TTTGGCTATTCCCGTCTCCGAGGGTGGGCAGCGGGAGACCGGGGTCTCTGAGCAATCTGAGAAAGTCCCACCTCGTAGAGGCCCATGTACGAGCACAACTGCTGAAATCACGCATAACTTCAGAAGGAGAGTACATACCACTCGATCAGATTGATATCAATGTGGGCTTCGATAGCGGCATTGACAGGATCTTTCTCGTTAGCCCAATCAGGAGACGTGCGACTCGAAAG
kir21_Frag663-822_insertion672	TTTGGCTATTCCCGTCTCCGAGGGTGGGCAATAGCGGGAGACCGGGGTCTCTGAGCCTGAGAAAGTCCCACCTCGTAGAGGCCCATGTACGAGCACAACTGCTGAAATCACGCATAACTTCAGAAGGAGAGTACATACCACTCGATCAGATTGATATCAATGTGGGCTTCGATAGCGGCATTGACAGGATCTTTCTCGTTAGCCCAATCAGGAGACGTGCGACTCGAAAG
kir21_Frag663-822_insertion675	TTTGGCTATTCCCGTCTCCGAGGGTGGGCAATCTGAGCGGGAGACCGGGGTCTCTGAGCAGAAAGTCCCACCTCGTAGAGGCCCATGTACGAGCACAACTGCTGAAATCACGCATAACTTCAGAAGGAGAGTACATACCACTCGATCAGATTGATATCAATGTGGGCTTCGATAGCGGCATTGACAGGATCTTTCTCGTTAGCCCAATCAGGAGACGTGCGACTCGAAAG
kir21_Frag663-822_insertion678	TTTGGCTATTCCCGTCTCCGAGGGTGGGCAATCTGAGAAGCGGGAGACCGGGGTCTCTGAGCAAGTCCCACCTCGTAGAGGCCCATGTACGAGCACAACTGCTGAAATCACGCATAACTTCAGAAGGAGAGTACATACCACTCGATCAGATTGATATCAATGTGGGCTTCGATAGCGGCATTGACAGGATCTTTCTCGTTAGCCCAATCAGGAGACGTGCGACTCGAAAG
kir21_Frag663-822_insertion681	TTTGGCTATTCCCGTCTCCGAGGGTGGGCAATCTGAGAAAGAGCGGGAGACCGGGGTCTCTGAGCTCCCACCTCGTAGAGGCCCATGTACGAGCACAACTGCTGAAATCACGCATAACTTCAGAAGGAGAGTACATACCACTCGATCAGATTGATATCAATGTGGGCTTCGATAGCGGCATTGACAGGATCTTTCTCGTTAGCCCAATCAGGAGACGTGCGACTCGAAAG
kir21_Frag663-822_insertion684	TTTGGCTATTCCCGTCTCCGAGGGTGGGCAATCTGAGAAAGTCCAGCGGGAGACCGGGGTCTCTGAGCCACCTCGTAGAGGCCCATGTACGAGCACAACTGCTGAAATCACGCATAACTTCAGAAGGAGAGTACATACCACTCGATCAGATTGATATCAATGTGGGCTTCGATAGCGGCATTGACAGGATCTTTCTCGTTAGCCCAATCAGGAGACGTGCGACTCGAAAG
kir21_Frag663-822_insertion687	TTTGGCTATTCCCGTCTCCGAGGGTGGGCAATCTGAGAAAGTCCCACAGCGGGAGACCGGGGTCTCTGAGCCTCGTAGAGGCCCATGTACGAGCACAACTGCTGAAATCACGCATAACTTCAGAAGGAGAGTACATACCACTCGATCAGATTGATATCAATGTGGGCTTCGATAGCGGCATTGACAGGATCTTTCTCGTTAGCCCAATCAGGAGACGTGCGACTCGAAAG
kir21_Frag663-822_insertion690	TTTGGCTATTCCCGTCTCCGAGGGTGGGCAATCTGAGAAAGTCCCACCTCAGCGGGAGACCGGGGTCTCTGAGCGTAGAGGCCCATGTACGAGCACAACTGCTGAAATCACGCATAACTTCAGAAGGAGAGTACATACCACTCGATCAGATTGATATCAATGTGGGCTTCGATAGCGGCATTGACAGGATCTTTCTCGTTAGCCCAATCAGGAGACGTGCGACTCGAAAG
kir21_Frag663-822_insertion693	TTTGGCTATTCCCGTCTCCGAGGGTGGGCAATCTGAGAAAGTCCCACCTCGTAAGCGGGAGACCGGGGTCTCTGAGCGAGGCCCATGTACGAGCACAACTGCTGAAATCACGCATAACTTCAGAAGGAGAGTACATACCACTCGATCAGATTGATATCAATGTGGGCTTCGATAGCGGCATTGACAGGATCTTTCTCGTTAGCCCAATCAGGAGACGTGCGACTCGAAAG
kir21_Frag663-822_insertion696	TTTGGCTATTCCCGTCTCCGAGGGTGGGCAATCTGAGAAAGTCCCACCTCGTAGAGAGCGGGAGACCGGGGTCTCTGAGCGCCCATGTACGAGCACAACTGCTGAAATCACGCATAACTTCAGAAGGAGAGTACATACCACTCGATCAGATTGATATCAATGTGGGCTTCGATAGCGGCATTGACAGGATCTTTCTCGTTAGCCCAATCAGGAGACGTGCGACTCGAAAG
kir21_Frag663-822_insertion699	TTTGGCTATTCCCGTCTCCGAGGGTGGGCAATCTGAGAAAGTCCCACCTCGTAGAGGCCAGCGGGAGACCGGGGTCTCTGAGCCATGTACGAGCACAACTGCTGAAATCACGCATAACTTCAGAAGGAGAGTACATACCACTCGATCAGATTGATATCAATGTGGGCTTCGATAGCGGCATTGACAGGATCTTTCTCGTTAGCCCAATCAGGAGACGTGCGACTCGAAAG
kir21_Frag663-822_insertion702	TTTGGCTATTCCCGTCTCCGAGGGTGGGCAATCTGAGAAAGTCCCACCTCGTAGAGGCCCATAGCGGGAGACCGGGGTCTCTGAGCGTACGAGCACAACTGCTGAAATCACGCATAACTTCAGAAGGAGAGTACATACCACTCGATCAGATTGATATCAATGTGGGCTTCGATAGCGGCATTGACAGGATCTTTCTCGTTAGCCCAATCAGGAGACGTGCGACTCGAAAG
kir21_Frag663-822_insertion705	TTTGGCTATTCCCGTCTCCGAGGGTGGGCAATCTGAGAAAGTCCCACCTCGTAGAGGCCCATGTAAGCGGGAGACCGGGGTCTCTGAGCCGAGCACAACTGCTGAAATCACGCATAACTTCAGAAGGAGAGTACATACCACTCGATCAGATTGATATCAATGTGGGCTTCGATAGCGGCATTGACAGGATCTTTCTCGTTAGCCCAATCAGGAGACGTGCGACTCGAAAG
kir21_Frag663-822_insertion708	TTTGGCTATTCCCGTCTCCGAGGGTGGGCAATCTGAGAAAGTCCCACCTCGTAGAGGCCCATGTACGAAGCGGGAGACCGGGGTCTCTGAGCGCACAACTGCTGAAATCACGCATAACTTCAGAAGGAGAGTACATACCACTCGATCAGATTGATATCAATGTGGGCTTCGATAGCGGCATTGACAGGATCTTTCTCGTTAGCCCAATCAGGAGACGTGCGACTCGAAAG
kir21_Frag663-822_insertion711	TTTGGCTATTCCCGTCTCCGAGGGTGGGCAATCTGAGAAAGTCCCACCTCGTAGAGGCCCATGTACGAGCAAGCGGGAGACCGGGGTCTCTGAGCCAACTGCTGAAATCACGCATAACTTCAGAAGGAGAGTACATACCACTCGATCAGATTGATATCAATGTGGGCTTCGATAGCGGCATTGACAGGATCTTTCTCGTTAGCCCAATCAGGAGACGTGCGACTCGAAAG
kir21_Frag663-822_insertion714	TTTGGCTATTCCCGTCTCCGAGGGTGGGCAATCTGAGAAAGTCCCACCTCGTAGAGGCCCATGTACGAGCACAAAGCGGGAGACCGGGGTCTCTGAGCCTGCTGAAATCACGCATAACTTCAGAAGGAGAGTACATACCACTCGATCAGATTGATATCAATGTGGGCTTCGATAGCGGCATTGACAGGATCTTTCTCGTTAGCCCAATCAGGAGACGTGCGACTCGAAAG
kir21_Frag663-822_insertion717	TTTGGCTATTCCCGTCTCCGAGGGTGGGCAATCTGAGAAAGTCCCACCTCGTAGAGGCCCATGTACGAGCACAACTGAGCGGGAGACCGGGGTCTCTGAGCCTGAAATCACGCATAACTTCAGAAGGAGAGTACATACCACTCGATCAGATTGATATCAATGTGGGCTTCGATAGCGGCATTGACAGGATCTTTCTCGTTAGCCCAATCAGGAGACGTGCGACTCGAAAG
kir21_Frag663-822_insertion720	TTTGGCTATTCCCGTCTCCGAGGGTGGGCAATCTGAGAAAGTCCCACCTCGTAGAGGCCCATGTACGAGCACAACTGCTGAGCGGGAGACCGGGGTCTCTGAGCAAATCACGCATAACTTCAGAAGGAGAGTACATACCACTCGATCAGATTGATATCAATGTGGGCTTCGATAGCGGCATTGACAGGATCTTTCTCGTTAGCCCAATCAGGAGACGTGCGACTCGAAAG
kir21_Frag663-822_insertion723	TTTGGCTATTCCCGTCTCCGAGGGTGGGCAATCTGAGAAAGTCCCACCTCGTAGAGGCCCATGTACGAGCACAACTGCTGAAAAGCGGGAGACCGGGGTCTCTGAGCTCACGCATAACTTCAGAAGGAGAGTACATACCACTCGATCAGATTGATATCAATGTGGGCTTCGATAGCGGCATTGACAGGATCTTTCTCGTTAGCCCAATCAGGAGACGTGCGACTCGAAAG
kir21_Frag663-822_insertion726	TTTGGCTATTCCCGTCTCCGAGGGTGGGCAATCTGAGAAAGTCCCACCTCGTAGAGGCCCATGTACGAGCACAACTGCTGAAATCAAGCGGGAGACCGGGGTCTCTGAGCCGCATAACTTCAGAAGGAGAGTACATACCACTCGATCAGATTGATATCAATGTGGGCTTCGATAGCGGCATTGACAGGATCTTTCTCGTTAGCCCAATCAGGAGACGTGCGACTCGAAAG
kir21_Frag663-822_insertion729	TTTGGCTATTCCCGTCTCCGAGGGTGGGCAATCTGAGAAAGTCCCACCTCGTAGAGGCCCATGTACGAGCACAACTGCTGAAATCACGCAGCGGGAGACCGGGGTCTCTGAGCATAACTTCAGAAGGAGAGTACATACCACTCGATCAGATTGATATCAATGTGGGCTTCGATAGCGGCATTGACAGGATCTTTCTCGTTAGCCCAATCAGGAGACGTGCGACTCGAAAG
kir21_Frag663-822_insertion732	TTTGGCTATTCCCGTCTCCGAGGGTGGGCAATCTGAGAAAGTCCCACCTCGTAGAGGCCCATGTACGAGCACAACTGCTGAAATCACGCATAAGCGGGAGACCGGGGTCTCTGAGCACTTCAGAAGGAGAGTACATACCACTCGATCAGATTGATATCAATGTGGGCTTCGATAGCGGCATTGACAGGATCTTTCTCGTTAGCCCAATCAGGAGACGTGCGACTCGAAAG
kir21_Frag663-822_insertion735	TTTGGCTATTCCCGTCTCCGAGGGTGGGCAATCTGAGAAAGTCCCACCTCGTAGAGGCCCATGTACGAGCACAACTGCTGAAATCACGCATAACTAGCGGGAGACCGGGGTCTCTGAGCTCAGAAGGAGAGTACATACCACTCGATCAGATTGATATCAATGTGGGCTTCGATAGCGGCATTGACAGGATCTTTCTCGTTAGCCCAATCAGGAGACGTGCGACTCGAAAG
kir21_Frag663-822_insertion738	TTTGGCTATTCCCGTCTCCGAGGGTGGGCAATCTGAGAAAGTCCCACCTCGTAGAGGCCCATGTACGAGCACAACTGCTGAAATCACGCATAACTTCAAGCGGGAGACCGGGGTCTCTGAGCGAAGGAGAGTACATACCACTCGATCAGATTGATATCAATGTGGGCTTCGATAGCGGCATTGACAGGATCTTTCTCGTTAGCCCAATCAGGAGACGTGCGACTCGAAAG
kir21_Frag663-822_insertion741	TTTGGCTATTCCCGTCTCCGAGGGTGGGCAATCTGAGAAAGTCCCACCTCGTAGAGGCCCATGTACGAGCACAACTGCTGAAATCACGCATAACTTCAGAAAGCGGGAGACCGGGGTCTCTGAGCGGAGAGTACATACCACTCGATCAGATTGATATCAATGTGGGCTTCGATAGCGGCATTGACAGGATCTTTCTCGTTAGCCCAATCAGGAGACGTGCGACTCGAAAG
kir21_Frag663-822_insertion744	TTTGGCTATTCCCGTCTCCGAGGGTGGGCAATCTGAGAAAGTCCCACCTCGTAGAGGCCCATGTACGAGCACAACTGCTGAAATCACGCATAACTTCAGAAGGAAGCGGGAGACCGGGGTCTCTGAGCGAGTACATACCACTCGATCAGATTGATATCAATGTGGGCTTCGATAGCGGCATTGACAGGATCTTTCTCGTTAGCCCAATCAGGAGACGTGCGACTCGAAAG
kir21_Frag663-822_insertion747	TTTGGCTATTCCCGTCTCCGAGGGTGGGCAATCTGAGAAAGTCCCACCTCGTAGAGGCCCATGTACGAGCACAACTGCTGAAATCACGCATAACTTCAGAAGGAGAGAGCGGGAGACCGGGGTCTCTGAGCTACATACCACTCGATCAGATTGATATCAATGTGGGCTTCGATAGCGGCATTGACAGGATCTTTCTCGTTAGCCCAATCAGGAGACGTGCGACTCGAAAG
kir21_Frag663-822_insertion750	TTTGGCTATTCCCGTCTCCGAGGGTGGGCAATCTGAGAAAGTCCCACCTCGTAGAGGCCCATGTACGAGCACAACTGCTGAAATCACGCATAACTTCAGAAGGAGAGTACAGCGGGAGACCGGGGTCTCTGAGCATACCACTCGATCAGATTGATATCAATGTGGGCTTCGATAGCGGCATTGACAGGATCTTTCTCGTTAGCCCAATCAGGAGACGTGCGACTCGAAAG
kir21_Frag663-822_insertion753	TTTGGCTATTCCCGTCTCCGAGGGTGGGCAATCTGAGAAAGTCCCACCTCGTAGAGGCCCATGTACGAGCACAACTGCTGAAATCACGCATAACTTCAGAAGGAGAGTACATAAGCGGGAGACCGGGGTCTCTGAGCCCACTCGATCAGATTGATATCAATGTGGGCTTCGATAGCGGCATTGACAGGATCTTTCTCGTTAGCCCAATCAGGAGACGTGCGACTCGAAAG
kir21_Frag663-822_insertion756	TTTGGCTATTCCCGTCTCCGAGGGTGGGCAATCTGAGAAAGTCCCACCTCGTAGAGGCCCATGTACGAGCACAACTGCTGAAATCACGCATAACTTCAGAAGGAGAGTACATACCAAGCGGGAGACCGGGGTCTCTGAGCCTCGATCAGATTGATATCAATGTGGGCTTCGATAGCGGCATTGACAGGATCTTTCTCGTTAGCCCAATCAGGAGACGTGCGACTCGAAAG

kir21_Frag663-822_insertion759	TTTGGCTATTCCCGTCTCCGAGGGTGGGCAATCTGAGAAAGTCCCACCTCGTAGAGGCCCATGTACGAGCACAACTGCTGAAATCACGCATAACTTCAGAAGGAGAGTACATACCACTCAGCGGGAGACCGGGGTCTCTGAGCGATCAGATTGATATCAATGTGGGCTTCGATAGCGGCATTGACAGGATCTTTCTCGTTAGCCCAATCAGGAGACGTGCGACTCGAAAG
kir21_Frag663-822_insertion762	TTTGGCTATTCCCGTCTCCGAGGGTGGGCAATCTGAGAAAGTCCCACCTCGTAGAGGCCCATGTACGAGCACAACTGCTGAAATCACGCATAACTTCAGAAGGAGAGTACATACCACTCGATAGCGGGAGACCGGGGTCTCTGAGCCAGATTGATATCAATGTGGGCTTCGATAGCGGCATTGACAGGATCTTTCTCGTTAGCCCAATCAGGAGACGTGCGACTCGAAAG
kir21_Frag663-822_insertion765	TTTGGCTATTCCCGTCTCCGAGGGTGGGCAATCTGAGAAAGTCCCACCTCGTAGAGGCCCATGTACGAGCACAACTGCTGAAATCACGCATAACTTCAGAAGGAGAGTACATACCACTCGATCAGAGCGGGAGACCGGGGTCTCTGAGCATTGATATCAATGTGGGCTTCGATAGCGGCATTGACAGGATCTTTCTCGTTAGCCCAATCAGGAGACGTGCGACTCGAAAG
kir21_Frag663-822_insertion768	TTTGGCTATTCCCGTCTCCGAGGGTGGGCAATCTGAGAAAGTCCCACCTCGTAGAGGCCCATGTACGAGCACAACTGCTGAAATCACGCATAACTTCAGAAGGAGAGTACATACCACTCGATCAGATTAGCGGGAGACCGGGGTCTCTGAGCGATATCAATGTGGGCTTCGATAGCGGCATTGACAGGATCTTTCTCGTTAGCCCAATCAGGAGACGTGCGACTCGAAAG
kir21_Frag663-822_insertion771	TTTGGCTATTCCCGTCTCCGAGGGTGGGCAATCTGAGAAAGTCCCACCTCGTAGAGGCCCATGTACGAGCACAACTGCTGAAATCACGCATAACTTCAGAAGGAGAGTACATACCACTCGATCAGATTGATAGCGGGAGACCGGGGTCTCTGAGCATCAATGTGGGCTTCGATAGCGGCATTGACAGGATCTTTCTCGTTAGCCCAATCAGGAGACGTGCGACTCGAAAG
kir21_Frag663-822_insertion774	TTTGGCTATTCCCGTCTCCGAGGGTGGGCAATCTGAGAAAGTCCCACCTCGTAGAGGCCCATGTACGAGCACAACTGCTGAAATCACGCATAACTTCAGAAGGAGAGTACATACCACTCGATCAGATTGATATCAGCGGGAGACCGGGGTCTCTGAGCAATGTGGGCTTCGATAGCGGCATTGACAGGATCTTTCTCGTTAGCCCAATCAGGAGACGTGCGACTCGAAAG
kir21_Frag663-822_insertion777	TTTGGCTATTCCCGTCTCCGAGGGTGGGCAATCTGAGAAAGTCCCACCTCGTAGAGGCCCATGTACGAGCACAACTGCTGAAATCACGCATAACTTCAGAAGGAGAGTACATACCACTCGATCAGATTGATATCAATAGCGGGAGACCGGGGTCTCTGAGCGTGGGCTTCGATAGCGGCATTGACAGGATCTTTCTCGTTAGCCCAATCAGGAGACGTGCGACTCGAAAG
kir21_Frag663-822_insertion780	TTTGGCTATTCCCGTCTCCGAGGGTGGGCAATCTGAGAAAGTCCCACCTCGTAGAGGCCCATGTACGAGCACAACTGCTGAAATCACGCATAACTTCAGAAGGAGAGTACATACCACTCGATCAGATTGATATCAATGTGAGCGGGAGACCGGGGTCTCTGAGCGGCTTCGATAGCGGCATTGACAGGATCTTTCTCGTTAGCCCAATCAGGAGACGTGCGACTCGAAAG
kir21_Frag663-822_insertion783	TTTGGCTATTCCCGTCTCCGAGGGTGGGCAATCTGAGAAAGTCCCACCTCGTAGAGGCCCATGTACGAGCACAACTGCTGAAATCACGCATAACTTCAGAAGGAGAGTACATACCACTCGATCAGATTGATATCAATGTGGGCAGCGGGAGACCGGGGTCTCTGAGCTTCGATAGCGGCATTGACAGGATCTTTCTCGTTAGCCCAATCAGGAGACGTGCGACTCGAAAG
kir21_Frag663-822_insertion786	TTTGGCTATTCCCGTCTCCGAGGGTGGGCAATCTGAGAAAGTCCCACCTCGTAGAGGCCCATGTACGAGCACAACTGCTGAAATCACGCATAACTTCAGAAGGAGAGTACATACCACTCGATCAGATTGATATCAATGTGGGCTTCAGCGGGAGACCGGGGTCTCTGAGCGATAGCGGCATTGACAGGATCTTTCTCGTTAGCCCAATCAGGAGACGTGCGACTCGAAAG
kir21_Frag663-822_insertion789	TTTGGCTATTCCCGTCTCCGAGGGTGGGCAATCTGAGAAAGTCCCACCTCGTAGAGGCCCATGTACGAGCACAACTGCTGAAATCACGCATAACTTCAGAAGGAGAGTACATACCACTCGATCAGATTGATATCAATGTGGGCTTCGATAGCGGGAGACCGGGGTCTCTGAGCAGCGGCATTGACAGGATCTTTCTCGTTAGCCCAATCAGGAGACGTGCGACTCGAAAG
kir21_Frag663-822_insertion792	TTTGGCTATTCCCGTCTCCGAGGGTGGGCAATCTGAGAAAGTCCCACCTCGTAGAGGCCCATGTACGAGCACAACTGCTGAAATCACGCATAACTTCAGAAGGAGAGTACATACCACTCGATCAGATTGATATCAATGTGGGCTTCGATAGCAGCGGGAGACCGGGGTCTCTGAGCGGCATTGACAGGATCTTTCTCGTTAGCCCAATCAGGAGACGTGCGACTCGAAAG
kir21_Frag663-822_insertion795	TTTGGCTATTCCCGTCTCCGAGGGTGGGCAATCTGAGAAAGTCCCACCTCGTAGAGGCCCATGTACGAGCACAACTGCTGAAATCACGCATAACTTCAGAAGGAGAGTACATACCACTCGATCAGATTGATATCAATGTGGGCTTCGATAGCGGCAGCGGGAGACCGGGGTCTCTGAGCATTGACAGGATCTTTCTCGTTAGCCCAATCAGGAGACGTGCGACTCGAAAG
kir21_Frag663-822_insertion798	TTTGGCTATTCCCGTCTCCGAGGGTGGGCAATCTGAGAAAGTCCCACCTCGTAGAGGCCCATGTACGAGCACAACTGCTGAAATCACGCATAACTTCAGAAGGAGAGTACATACCACTCGATCAGATTGATATCAATGTGGGCTTCGATAGCGGCATTAGCGGGAGACCGGGGTCTCTGAGCGACAGGATCTTTCTCGTTAGCCCAATCAGGAGACGTGCGACTCGAAAG
kir21_Frag663-822_insertion801	TTTGGCTATTCCCGTCTCCGAGGGTGGGCAATCTGAGAAAGTCCCACCTCGTAGAGGCCCATGTACGAGCACAACTGCTGAAATCACGCATAACTTCAGAAGGAGAGTACATACCACTCGATCAGATTGATATCAATGTGGGCTTCGATAGCGGCATTGACAGCGGGAGACCGGGGTCTCTGAGCAGGATCTTTCTCGTTAGCCCAATCAGGAGACGTGCGACTCGAAAG
kir21_Frag663-822_insertion804	TTTGGCTATTCCCGTCTCCGAGGGTGGGCAATCTGAGAAAGTCCCACCTCGTAGAGGCCCATGTACGAGCACAACTGCTGAAATCACGCATAACTTCAGAAGGAGAGTACATACCACTCGATCAGATTGATATCAATGTGGGCTTCGATAGCGGCATTGACAGGAGCGGGAGACCGGGGTCTCTGAGCATCTTTCTCGTTAGCCCAATCAGGAGACGTGCGACTCGAAAG
kir21_Frag663-822_insertion807	TTTGGCTATTCCCGTCTCCGAGGGTGGGCAATCTGAGAAAGTCCCACCTCGTAGAGGCCCATGTACGAGCACAACTGCTGAAATCACGCATAACTTCAGAAGGAGAGTACATACCACTCGATCAGATTGATATCAATGTGGGCTTCGATAGCGGCATTGACAGGATCAGCGGGAGACCGGGGTCTCTGAGCTTTCTCGTTAGCCCAATCAGGAGACGTGCGACTCGAAAG
kir21_Frag663-822_insertion810	TTTGGCTATTCCCGTCTCCGAGGGTGGGCAATCTGAGAAAGTCCCACCTCGTAGAGGCCCATGTACGAGCACAACTGCTGAAATCACGCATAACTTCAGAAGGAGAGTACATACCACTCGATCAGATTGATATCAATGTGGGCTTCGATAGCGGCATTGACAGGATCTTTAGCGGGAGACCGGGGTCTCTGAGCCTCGTTAGCCCAATCAGGAGACGTGCGACTCGAAAG
kir21_Frag663-822_insertion813	TTTGGCTATTCCCGTCTCCGAGGGTGGGCAATCTGAGAAAGTCCCACCTCGTAGAGGCCCATGTACGAGCACAACTGCTGAAATCACGCATAACTTCAGAAGGAGAGTACATACCACTCGATCAGATTGATATCAATGTGGGCTTCGATAGCGGCATTGACAGGATCTTTCTCAGCGGGAGACCGGGGTCTCTGAGCGTTAGCCCAATCAGGAGACGTGCGACTCGAAAG
kir21_Frag663-822_insertion816	TTTGGCTATTCCCGTCTCCGAGGGTGGGCAATCTGAGAAAGTCCCACCTCGTAGAGGCCCATGTACGAGCACAACTGCTGAAATCACGCATAACTTCAGAAGGAGAGTACATACCACTCGATCAGATTGATATCAATGTGGGCTTCGATAGCGGCATTGACAGGATCTTTCTCGTTAGCGGGAGACCGGGGTCTCTGAGCAGCCCAATCAGGAGACGTGCGACTCGAAAG
kir21_Frag663-822_insertion819	TTTGGCTATTCCCGTCTCCGAGGGTGGGCAATCTGAGAAAGTCCCACCTCGTAGAGGCCCATGTACGAGCACAACTGCTGAAATCACGCATAACTTCAGAAGGAGAGTACATACCACTCGATCAGATTGATATCAATGTGGGCTTCGATAGCGGCATTGACAGGATCTTTCTCGTTAGCAGCGGGAGACCGGGGTCTCTGAGCCCAATCAGGAGACGTGCGACTCGAAAG
kir21_Frag663-822_insertion822	TTTGGCTATTCCCGTCTCCGAGGGTGGGCAATCTGAGAAAGTCCCACCTCGTAGAGGCCCATGTACGAGCACAACTGCTGAAATCACGCATAACTTCAGAAGGAGAGTACATACCACTCGATCAGATTGATATCAATGTGGGCTTCGATAGCGGCATTGACAGGATCTTTCTCGTTAGCCCAAGCGGGAGACCGGGGTCTCTGAGCATCAGGAGACGTGCGACTCGAAAG
kir21_Frag825-984_insertion825	TATTTCCGCTCTCGTCTCCAATCAGCGGGAGACCGGGGTCTCTGAGCACCATCGTCCACGAGATTGATGAGGATTCTCCTCTTTATGACCTCTCCAAACAAGACATCGACAACGCTGATTTCGAAATTGTCGTTATACTGGAAGGGATGGTAGAGGCCACCGCTATGACAACCCAATGTCGAAGTAGTTATCTTGCCAATGAAATCCTTTGGAGACGAGGGTACGAAAAT
kir21_Frag825-984_insertion828	TATTTCCGCTCTCGTCTCCAATCACCAGCGGGAGACCGGGGTCTCTGAGCATCGTCCACGAGATTGATGAGGATTCTCCTCTTTATGACCTCTCCAAACAAGACATCGACAACGCTGATTTCGAAATTGTCGTTATACTGGAAGGGATGGTAGAGGCCACCGCTATGACAACCCAATGTCGAAGTAGTTATCTTGCCAATGAAATCCTTTGGAGACGAGGGTACGAAAAT
kir21_Frag825-984_insertion831	TATTTCCGCTCTCGTCTCCAATCACCATCAGCGGGAGACCGGGGTCTCTGAGCGTCCACGAGATTGATGAGGATTCTCCTCTTTATGACCTCTCCAAACAAGACATCGACAACGCTGATTTCGAAATTGTCGTTATACTGGAAGGGATGGTAGAGGCCACCGCTATGACAACCCAATGTCGAAGTAGTTATCTTGCCAATGAAATCCTTTGGAGACGAGGGTACGAAAAT
kir21_Frag825-984_insertion834	TATTTCCGCTCTCGTCTCCAATCACCATCGTCAGCGGGAGACCGGGGTCTCTGAGCCACGAGATTGATGAGGATTCTCCTCTTTATGACCTCTCCAAACAAGACATCGACAACGCTGATTTCGAAATTGTCGTTATACTGGAAGGGATGGTAGAGGCCACCGCTATGACAACCCAATGTCGAAGTAGTTATCTTGCCAATGAAATCCTTTGGAGACGAGGGTACGAAAAT
kir21_Frag825-984_insertion837	TATTTCCGCTCTCGTCTCCAATCACCATCGTCCACAGCGGGAGACCGGGGTCTCTGAGCGAGATTGATGAGGATTCTCCTCTTTATGACCTCTCCAAACAAGACATCGACAACGCTGATTTCGAAATTGTCGTTATACTGGAAGGGATGGTAGAGGCCACCGCTATGACAACCCAATGTCGAAGTAGTTATCTTGCCAATGAAATCCTTTGGAGACGAGGGTACGAAAAT
kir21_Frag825-984_insertion840	TATTTCCGCTCTCGTCTCCAATCACCATCGTCCACGAGAGCGGGAGACCGGGGTCTCTGAGCATTGATGAGGATTCTCCTCTTTATGACCTCTCCAAACAAGACATCGACAACGCTGATTTCGAAATTGTCGTTATACTGGAAGGGATGGTAGAGGCCACCGCTATGACAACCCAATGTCGAAGTAGTTATCTTGCCAATGAAATCCTTTGGAGACGAGGGTACGAAAAT
kir21_Frag825-984_insertion843	TATTTCCGCTCTCGTCTCCAATCACCATCGTCCACGAGATTAGCGGGAGACCGGGGTCTCTGAGCGATGAGGATTCTCCTCTTTATGACCTCTCCAAACAAGACATCGACAACGCTGATTTCGAAATTGTCGTTATACTGGAAGGGATGGTAGAGGCCACCGCTATGACAACCCAATGTCGAAGTAGTTATCTTGCCAATGAAATCCTTTGGAGACGAGGGTACGAAAAT
kir21_Frag825-984_insertion846	TATTTCCGCTCTCGTCTCCAATCACCATCGTCCACGAGATTGATAGCGGGAGACCGGGGTCTCTGAGCGAGGATTCTCCTCTTTATGACCTCTCCAAACAAGACATCGACAACGCTGATTTCGAAATTGTCGTTATACTGGAAGGGATGGTAGAGGCCACCGCTATGACAACCCAATGTCGAAGTAGTTATCTTGCCAATGAAATCCTTTGGAGACGAGGGTACGAAAAT
kir21_Frag825-984_insertion849	TATTTCCGCTCTCGTCTCCAATCACCATCGTCCACGAGATTGATGAGAGCGGGAGACCGGGGTCTCTGAGCGATTCTCCTCTTTATGACCTCTCCAAACAAGACATCGACAACGCTGATTTCGAAATTGTCGTTATACTGGAAGGGATGGTAGAGGCCACCGCTATGACAACCCAATGTCGAAGTAGTTATCTTGCCAATGAAATCCTTTGGAGACGAGGGTACGAAAAT
kir21_Frag825-984_insertion852	TATTTCCGCTCTCGTCTCCAATCACCATCGTCCACGAGATTGATGAGGATAGCGGGAGACCGGGGTCTCTGAGCTCTCCTCTTTATGACCTCTCCAAACAAGACATCGACAACGCTGATTTCGAAATTGTCGTTATACTGGAAGGGATGGTAGAGGCCACCGCTATGACAACCCAATGTCGAAGTAGTTATCTTGCCAATGAAATCCTTTGGAGACGAGGGTACGAAAAT
kir21_Frag825-984_insertion855	TATTTCCGCTCTCGTCTCCAATCACCATCGTCCACGAGATTGATGAGGATTCTAGCGGGAGACCGGGGTCTCTGAGCCCTCTTTATGACCTCTCCAAACAAGACATCGACAACGCTGATTTCGAAATTGTCGTTATACTGGAAGGGATGGTAGAGGCCACCGCTATGACAACCCAATGTCGAAGTAGTTATCTTGCCAATGAAATCCTTTGGAGACGAGGGTACGAAAAT
kir21_Frag825-984_insertion858	TATTTCCGCTCTCGTCTCCAATCACCATCGTCCACGAGATTGATGAGGATTCTCCTAGCGGGAGACCGGGGTCTCTGAGCCTTTATGACCTCTCCAAACAAGACATCGACAACGCTGATTTCGAAATTGTCGTTATACTGGAAGGGATGGTAGAGGCCACCGCTATGACAACCCAATGTCGAAGTAGTTATCTTGCCAATGAAATCCTTTGGAGACGAGGGTACGAAAAT
kir21_Frag825-984_insertion861	TATTTCCGCTCTCGTCTCCAATCACCATCGTCCACGAGATTGATGAGGATTCTCCTCTTAGCGGGAGACCGGGGTCTCTGAGCTATGACCTCTCCAAACAAGACATCGACAACGCTGATTTCGAAATTGTCGTTATACTGGAAGGGATGGTAGAGGCCACCGCTATGACAACCCAATGTCGAAGTAGTTATCTTGCCAATGAAATCCTTTGGAGACGAGGGTACGAAAAT
kir21_Frag825-984_insertion864	TATTTCCGCTCTCGTCTCCAATCACCATCGTCCACGAGATTGATGAGGATTCTCCTCTTTATAGCGGGAGACCGGGGTCTCTGAGCGACCTCTCCAAACAAGACATCGACAACGCTGATTTCGAAATTGTCGTTATACTGGAAGGGATGGTAGAGGCCACCGCTATGACAACCCAATGTCGAAGTAGTTATCTTGCCAATGAAATCCTTTGGAGACGAGGGTACGAAAAT
kir21_Frag825-984_insertion867	TATTTCCGCTCTCGTCTCCAATCACCATCGTCCACGAGATTGATGAGGATTCTCCTCTTTATGACAGCGGGAGACCGGGGTCTCTGAGCCTCTCCAAACAAGACATCGACAACGCTGATTTCGAAATTGTCGTTATACTGGAAGGGATGGTAGAGGCCACCGCTATGACAACCCAATGTCGAAGTAGTTATCTTGCCAATGAAATCCTTTGGAGACGAGGGTACGAAAAT
kir21_Frag825-984_insertion870	TATTTCCGCTCTCGTCTCCAATCACCATCGTCCACGAGATTGATGAGGATTCTCCTCTTTATGACCTCAGCGGGAGACCGGGGTCTCTGAGCTCCAAACAAGACATCGACAACGCTGATTTCGAAATTGTCGTTATACTGGAAGGGATGGTAGAGGCCACCGCTATGACAACCCAATGTCGAAGTAGTTATCTTGCCAATGAAATCCTTTGGAGACGAGGGTACGAAAAT
kir21_Frag825-984_insertion873	TATTTCCGCTCTCGTCTCCAATCACCATCGTCCACGAGATTGATGAGGATTCTCCTCTTTATGACCTCTCCAGCGGGAGACCGGGGTCTCTGAGCAAACAAGACATCGACAACGCTGATTTCGAAATTGTCGTTATACTGGAAGGGATGGTAGAGGCCACCGCTATGACAACCCAATGTCGAAGTAGTTATCTTGCCAATGAAATCCTTTGGAGACGAGGGTACGAAAAT
kir21_Frag825-984_insertion876	TATTTCCGCTCTCGTCTCCAATCACCATCGTCCACGAGATTGATGAGGATTCTCCTCTTTATGACCTCTCCAAAAGCGGGAGACCGGGGTCTCTGAGCCAAGACATCGACAACGCTGATTTCGAAATTGTCGTTATACTGGAAGGGATGGTAGAGGCCACCGCTATGACAACCCAATGTCGAAGTAGTTATCTTGCCAATGAAATCCTTTGGAGACGAGGGTACGAAAAT
kir21_Frag825-984_insertion879	TATTTCCGCTCTCGTCTCCAATCACCATCGTCCACGAGATTGATGAGGATTCTCCTCTTTATGACCTCTCCAAACAAAGCGGGAGACCGGGGTCTCTGAGCGACATCGACAACGCTGATTTCGAAATTGTCGTTATACTGGAAGGGATGGTAGAGGCCACCGCTATGACAACCCAATGTCGAAGTAGTTATCTTGCCAATGAAATCCTTTGGAGACGAGGGTACGAAAAT
kir21_Frag825-984_insertion882	TATTTCCGCTCTCGTCTCCAATCACCATCGTCCACGAGATTGATGAGGATTCTCCTCTTTATGACCTCTCCAAACAAGACAGCGGGAGACCGGGGTCTCTGAGCATCGACAACGCTGATTTCGAAATTGTCGTTATACTGGAAGGGATGGTAGAGGCCACCGCTATGACAACCCAATGTCGAAGTAGTTATCTTGCCAATGAAATCCTTTGGAGACGAGGGTACGAAAAT
kir21_Frag825-984_insertion885	TATTTCCGCTCTCGTCTCCAATCACCATCGTCCACGAGATTGATGAGGATTCTCCTCTTTATGACCTCTCCAAACAAGACATCAGCGGGAGACCGGGGTCTCTGAGCGACAACGCTGATTTCGAAATTGTCGTTATACTGGAAGGGATGGTAGAGGCCACCGCTATGACAACCCAATGTCGAAGTAGTTATCTTGCCAATGAAATCCTTTGGAGACGAGGGTACGAAAAT
kir21_Frag825-984_insertion888	TATTTCCGCTCTCGTCTCCAATCACCATCGTCCACGAGATTGATGAGGATTCTCCTCTTTATGACCTCTCCAAACAAGACATCGACAGCGGGAGACCGGGGTCTCTGAGCAACGCTGATTTCGAAATTGTCGTTATACTGGAAGGGATGGTAGAGGCCACCGCTATGACAACCCAATGTCGAAGTAGTTATCTTGCCAATGAAATCCTTTGGAGACGAGGGTACGAAAAT
kir21_Frag825-984_insertion891	TATTTCCGCTCTCGTCTCCAATCACCATCGTCCACGAGATTGATGAGGATTCTCCTCTTTATGACCTCTCCAAACAAGACATCGACAACAGCGGGAGACCGGGGTCTCTGAGCGCTGATTTCGAAATTGTCGTTATACTGGAAGGGATGGTAGAGGCCACCGCTATGACAACCCAATGTCGAAGTAGTTATCTTGCCAATGAAATCCTTTGGAGACGAGGGTACGAAAAT
kir21_Frag825-984_insertion894	TATTTCCGCTCTCGTCTCCAATCACCATCGTCCACGAGATTGATGAGGATTCTCCTCTTTATGACCTCTCCAAACAAGACATCGACAACGCTAGCGGGAGACCGGGGTCTCTGAGCGATTTCGAAATTGTCGTTATACTGGAAGGGATGGTAGAGGCCACCGCTATGACAACCCAATGTCGAAGTAGTTATCTTGCCAATGAAATCCTTTGGAGACGAGGGTACGAAAAT
kir21_Frag825-984_insertion897	TATTTCCGCTCTCGTCTCCAATCACCATCGTCCACGAGATTGATGAGGATTCTCCTCTTTATGACCTCTCCAAACAAGACATCGACAACGCTGATAGCGGGAGACCGGGGTCTCTGAGCTTCGAAATTGTCGTTATACTGGAAGGGATGGTAGAGGCCACCGCTATGACAACCCAATGTCGAAGTAGTTATCTTGCCAATGAAATCCTTTGGAGACGAGGGTACGAAAAT
kir21_Frag825-984_insertion900	TATTTCCGCTCTCGTCTCCAATCACCATCGTCCACGAGATTGATGAGGATTCTCCTCTTTATGACCTCTCCAAACAAGACATCGACAACGCTGATTTCAGCGGGAGACCGGGGTCTCTGAGCGAAATTGTCGTTATACTGGAAGGGATGGTAGAGGCCACCGCTATGACAACCCAATGTCGAAGTAGTTATCTTGCCAATGAAATCCTTTGGAGACGAGGGTACGAAAAT
kir21_Frag825-984_insertion903	TATTTCCGCTCTCGTCTCCAATCACCATCGTCCACGAGATTGATGAGGATTCTCCTCTTTATGACCTCTCCAAACAAGACATCGACAACGCTGATTTCGAAAGCGGGAGACCGGGGTCTCTGAGCATTGTCGTTATACTGGAAGGGATGGTAGAGGCCACCGCTATGACAACCCAATGTCGAAGTAGTTATCTTGCCAATGAAATCCTTTGGAGACGAGGGTACGAAAAT
kir21_Frag825-984_insertion906	TATTTCCGCTCTCGTCTCCAATCACCATCGTCCACGAGATTGATGAGGATTCTCCTCTTTATGACCTCTCCAAACAAGACATCGACAACGCTGATTTCGAAATTAGCGGGAGACCGGGGTCTCTGAGCGTCGTTATACTGGAAGGGATGGTAGAGGCCACCGCTATGACAACCCAATGTCGAAGTAGTTATCTTGCCAATGAAATCCTTTGGAGACGAGGGTACGAAAAT
kir21_Frag825-984_insertion909	TATTTCCGCTCTCGTCTCCAATCACCATCGTCCACGAGATTGATGAGGATTCTCCTCTTTATGACCTCTCCAAACAAGACATCGACAACGCTGATTTCGAAATTGTCAGCGGGAGACCGGGGTCTCTGAGCGTTATACTGGAAGGGATGGTAGAGGCCACCGCTATGACAACCCAATGTCGAAGTAGTTATCTTGCCAATGAAATCCTTTGGAGACGAGGGTACGAAAAT
kir21_Frag825-984_insertion912	TATTTCCGCTCTCGTCTCCAATCACCATCGTCCACGAGATTGATGAGGATTCTCCTCTTTATGACCTCTCCAAACAAGACATCGACAACGCTGATTTCGAAATTGTCGTTAGCGGGAGACCGGGGTCTCTGAGCATACTGGAAGGGATGGTAGAGGCCACCGCTATGACAACCCAATGTCGAAGTAGTTATCTTGCCAATGAAATCCTTTGGAGACGAGGGTACGAAAAT
kir21_Frag825-984_insertion915	TATTTCCGCTCTCGTCTCCAATCACCATCGTCCACGAGATTGATGAGGATTCTCCTCTTTATGACCTCTCCAAACAAGACATCGACAACGCTGATTTCGAAATTGTCGTTATAAGCGGGAGACCGGGGTCTCTGAGCCTGGAAGGGATGGTAGAGGCCACCGCTATGACAACCCAATGTCGAAGTAGTTATCTTGCCAATGAAATCCTTTGGAGACGAGGGTACGAAAAT
kir21_Frag825-984_insertion918	TATTTCCGCTCTCGTCTCCAATCACCATCGTCCACGAGATTGATGAGGATTCTCCTCTTTATGACCTCTCCAAACAAGACATCGACAACGCTGATTTCGAAATTGTCGTTATACTGAGCGGGAGACCGGGGTCTCTGAGCGAAGGGATGGTAGAGGCCACCGCTATGACAACCCAATGTCGAAGTAGTTATCTTGCCAATGAAATCCTTTGGAGACGAGGGTACGAAAAT
kir21_Frag825-984_insertion921	TATTTCCGCTCTCGTCTCCAATCACCATCGTCCACGAGATTGATGAGGATTCTCCTCTTTATGACCTCTCCAAACAAGACATCGACAACGCTGATTTCGAAATTGTCGTTATACTGGAAAGCGGGAGACCGGGGTCTCTGAGCGGGATGGTAGAGGCCACCGCTATGACAACCCAATGTCGAAGTAGTTATCTTGCCAATGAAATCCTTTGGAGACGAGGGTACGAAAAT
kir21_Frag825-984_insertion924	TATTTCCGCTCTCGTCTCCAATCACCATCGTCCACGAGATTGATGAGGATTCTCCTCTTTATGACCTCTCCAAACAAGACATCGACAACGCTGATTTCGAAATTGTCGTTATACTGGAAGGGAGCGGGAGACCGGGGTCTCTGAGCATGGTAGAGGCCACCGCTATGACAACCCAATGTCGAAGTAGTTATCTTGCCAATGAAATCCTTTGGAGACGAGGGTACGAAAAT
kir21_Frag825-984_insertion927	TATTTCCGCTCTCGTCTCCAATCACCATCGTCCACGAGATTGATGAGGATTCTCCTCTTTATGACCTCTCCAAACAAGACATCGACAACGCTGATTTCGAAATTGTCGTTATACTGGAAGGGATGAGCGGGAGACCGGGGTCTCTGAGCGTAGAGGCCACCGCTATGACAACCCAATGTCGAAGTAGTTATCTTGCCAATGAAATCCTTTGGAGACGAGGGTACGAAAAT
kir21_Frag825-984_insertion930	TATTTCCGCTCTCGTCTCCAATCACCATCGTCCACGAGATTGATGAGGATTCTCCTCTTTATGACCTCTCCAAACAAGACATCGACAACGCTGATTTCGAAATTGTCGTTATACTGGAAGGGATGGTAAGCGGGAGACCGGGGTCTCTGAGCGAGGCCACCGCTATGACAACCCAATGTCGAAGTAGTTATCTTGCCAATGAAATCCTTTGGAGACGAGGGTACGAAAAT
kir21_Frag825-984_insertion933	TATTTCCGCTCTCGTCTCCAATCACCATCGTCCACGAGATTGATGAGGATTCTCCTCTTTATGACCTCTCCAAACAAGACATCGACAACGCTGATTTCGAAATTGTCGTTATACTGGAAGGGATGGTAGAGAGCGGGAGACCGGGGTCTCTGAGCGCCACCGCTATGACAACCCAATGTCGAAGTAGTTATCTTGCCAATGAAATCCTTTGGAGACGAGGGTACGAAAAT
kir21_Frag825-984_insertion936	TATTTCCGCTCTCGTCTCCAATCACCATCGTCCACGAGATTGATGAGGATTCTCCTCTTTATGACCTCTCCAAACAAGACATCGACAACGCTGATTTCGAAATTGTCGTTATACTGGAAGGGATGGTAGAGGCCAGCGGGAGACCGGGGTCTCTGAGCACCGCTATGACAACCCAATGTCGAAGTAGTTATCTTGCCAATGAAATCCTTTGGAGACGAGGGTACGAAAAT
kir21_Frag825-984_insertion939	TATTTCCGCTCTCGTCTCCAATCACCATCGTCCACGAGATTGATGAGGATTCTCCTCTTTATGACCTCTCCAAACAAGACATCGACAACGCTGATTTCGAAATTGTCGTTATACTGGAAGGGATGGTAGAGGCCACCAGCGGGAGACCGGGGTCTCTGAGCGCTATGACAACCCAATGTCGAAGTAGTTATCTTGCCAATGAAATCCTTTGGAGACGAGGGTACGAAAAT
kir21_Frag825-984_insertion942	TATTTCCGCTCTCGTCTCCAATCACCATCGTCCACGAGATTGATGAGGATTCTCCTCTTTATGACCTCTCCAAACAAGACATCGACAACGCTGATTTCGAAATTGTCGTTATACTGGAAGGGATGGTAGAGGCCACCGCTAGCGGGAGACCGGGGTCTCTGAGCATGACAACCCAATGTCGAAGTAGTTATCTTGCCAATGAAATCCTTTGGAGACGAGGGTACGAAAAT
kir21_Frag825-984_insertion945	TATTTCCGCTCTCGTCTCCAATCACCATCGTCCACGAGATTGATGAGGATTCTCCTCTTTATGACCTCTCCAAACAAGACATCGACAACGCTGATTTCGAAATTGTCGTTATACTGGAAGGGATGGTAGAGGCCACCGCTATGAGCGGGAGACCGGGGTCTCTGAGCACAACCCAATGTCGAAGTAGTTATCTTGCCAATGAAATCCTTTGGAGACGAGGGTACGAAAAT
kir21_Frag825-984_insertion948	TATTTCCGCTCTCGTCTCCAATCACCATCGTCCACGAGATTGATGAGGATTCTCCTCTTTATGACCTCTCCAAACAAGACATCGACAACGCTGATTTCGAAATTGTCGTTATACTGGAAGGGATGGTAGAGGCCACCGCTATGACAAGCGGGAGACCGGGGTCTCTGAGCACCCAATGTCGAAGTAGTTATCTTGCCAATGAAATCCTTTGGAGACGAGGGTACGAAAAT
kir21_Frag825-984_insertion951	TATTTCCGCTCTCGTCTCCAATCACCATCGTCCACGAGATTGATGAGGATTCTCCTCTTTATGACCTCTCCAAACAAGACATCGACAACGCTGATTTCGAAATTGTCGTTATACTGGAAGGGATGGTAGAGGCCACCGCTATGACAACCAGCGGGAGACCGGGGTCTCTGAGCCAATGTCGAAGTAGTTATCTTGCCAATGAAATCCTTTGGAGACGAGGGTACGAAAAT
kir21_Frag825-984_insertion954	TATTTCCGCTCTCGTCTCCAATCACCATCGTCCACGAGATTGATGAGGATTCTCCTCTTTATGACCTCTCCAAACAAGACATCGACAACGCTGATTTCGAAATTGTCGTTATACTGGAAGGGATGGTAGAGGCCACCGCTATGACAACCCAAAGCGGGAGACCGGGGTCTCTGAGCTGTCGAAGTAGTTATCTTGCCAATGAAATCCTTTGGAGACGAGGGTACGAAAAT
kir21_Frag825-984_insertion957	TATTTCCGCTCTCGTCTCCAATCACCATCGTCCACGAGATTGATGAGGATTCTCCTCTTTATGACCTCTCCAAACAAGACATCGACAACGCTGATTTCGAAATTGTCGTTATACTGGAAGGGATGGTAGAGGCCACCGCTATGACAACCCAATGTAGCGGGAGACCGGGGTCTCTGAGCCGAAGTAGTTATCTTGCCAATGAAATCCTTTGGAGACGAGGGTACGAAAAT
kir21_Frag825-984_insertion960	TATTTCCGCTCTCGTCTCCAATCACCATCGTCCACGAGATTGATGAGGATTCTCCTCTTTATGACCTCTCCAAACAAGACATCGACAACGCTGATTTCGAAATTGTCGTTATACTGGAAGGGATGGTAGAGGCCACCGCTATGACAACCCAATGTCGAAGCGGGAGACCGGGGTCTCTGAGCAGTAGTTATCTTGCCAATGAAATCCTTTGGAGACGAGGGTACGAAAAT
kir21_Frag825-984_insertion963	TATTTCCGCTCTCGTCTCCAATCACCATCGTCCACGAGATTGATGAGGATTCTCCTCTTTATGACCTCTCCAAACAAGACATCGACAACGCTGATTTCGAAATTGTCGTTATACTGGAAGGGATGGTAGAGGCCACCGCTATGACAACCCAATGTCGAAGTAGCGGGAGACCGGGGTCTCTGAGCAGTTATCTTGCCAATGAAATCCTTTGGAGACGAGGGTACGAAAAT
kir21_Frag825-984_insertion966	TATTTCCGCTCTCGTCTCCAATCACCATCGTCCACGAGATTGATGAGGATTCTCCTCTTTATGACCTCTCCAAACAAGACATCGACAACGCTGATTTCGAAATTGTCGTTATACTGGAAGGGATGGTAGAGGCCACCGCTATGACAACCCAATGTCGAAGTAGTAGCGGGAGACCGGGGTCTCTGAGCTATCTTGCCAATGAAATCCTTTGGAGACGAGGGTACGAAAAT
kir21_Frag825-984_insertion969	TATTTCCGCTCTCGTCTCCAATCACCATCGTCCACGAGATTGATGAGGATTCTCCTCTTTATGACCTCTCCAAACAAGACATCGACAACGCTGATTTCGAAATTGTCGTTATACTGGAAGGGATGGTAGAGGCCACCGCTATGACAACCCAATGTCGAAGTAGTTATAGCGGGAGACCGGGGTCTCTGAGCCTTGCCAATGAAATCCTTTGGAGACGAGGGTACGAAAAT
kir21_Frag825-984_insertion972	TATTTCCGCTCTCGTCTCCAATCACCATCGTCCACGAGATTGATGAGGATTCTCCTCTTTATGACCTCTCCAAACAAGACATCGACAACGCTGATTTCGAAATTGTCGTTATACTGGAAGGGATGGTAGAGGCCACCGCTATGACAACCCAATGTCGAAGTAGTTATCTTAGCGGGAGACCGGGGTCTCTGAGCGCCAATGAAATCCTTTGGAGACGAGGGTACGAAAAT
kir21_Frag825-984_insertion975	TATTTCCGCTCTCGTCTCCAATCACCATCGTCCACGAGATTGATGAGGATTCTCCTCTTTATGACCTCTCCAAACAAGACATCGACAACGCTGATTTCGAAATTGTCGTTATACTGGAAGGGATGGTAGAGGCCACCGCTATGACAACCCAATGTCGAAGTAGTTATCTTGCCAGCGGGAGACCGGGGTCTCTGAGCAATGAAATCCTTTGGAGACGAGGGTACGAAAAT
kir21_Frag825-984_insertion978	TATTTCCGCTCTCGTCTCCAATCACCATCGTCCACGAGATTGATGAGGATTCTCCTCTTTATGACCTCTCCAAACAAGACATCGACAACGCTGATTTCGAAATTGTCGTTATACTGGAAGGGATGGTAGAGGCCACCGCTATGACAACCCAATGTCGAAGTAGTTATCTTGCCAATAGCGGGAGACCGGGGTCTCTGAGCGAAATCCTTTGGAGACGAGGGTACGAAAAT
kir21_Frag825-984_insertion981	TATTTCCGCTCTCGTCTCCAATCACCATCGTCCACGAGATTGATGAGGATTCTCCTCTTTATGACCTCTCCAAACAAGACATCGACAACGCTGATTTCGAAATTGTCGTTATACTGGAAGGGATGGTAGAGGCCACCGCTATGACAACCCAATGTCGAAGTAGTTATCTTGCCAATGAAAGCGGGAGACCGGGGTCTCTGAGCATCCTTTGGAGACGAGGGTACGAAAAT
kir21_Frag825-984_insertion984	TATTTCCGCTCTCGTCTCCAATCACCATCGTCCACGAGATTGATGAGGATTCTCCTCTTTATGACCTCTCCAAACAAGACATCGACAACGCTGATTTCGAAATTGTCGTTATACTGGAAGGGATGGTAGAGGCCACCGCTATGACAACCCAATGTCGAAGTAGTTATCTTGCCAATGAAATCAGCGGGAGACCGGGGTCTCTGAGCCTTTGGAGACGAGGGTACGAAAAT
kir21_Frag987-1146_insertion987	CTAAATAGTGCTCGTCTCCCCTTAGCGGGAGACCGGGGTCTCTGAGCTGGGGCCATCGCTACGAACCTGTCTTGTTTGAGGAGAAGCACTATTATAAGGTCGACTACTCTAGGTTCCACAAAACATACGAAGTTCCTAACACACCATTGTGTAGTGCTCGGGACCTTGCAGAGAAGAAGTACATTCTGTCTAACGCAAACTCTTTCTGTTGGAGACGCCCGAGGGGAGAA
kir21_Frag987-1146_insertion990	CTAAATAGTGCTCGTCTCCCCTTTGGAGCGGGAGACCGGGGTCTCTGAGCGGCCATCGCTACGAACCTGTCTTGTTTGAGGAGAAGCACTATTATAAGGTCGACTACTCTAGGTTCCACAAAACATACGAAGTTCCTAACACACCATTGTGTAGTGCTCGGGACCTTGCAGAGAAGAAGTACATTCTGTCTAACGCAAACTCTTTCTGTTGGAGACGCCCGAGGGGAGAA
kir21_Frag987-1146_insertion993	CTAAATAGTGCTCGTCTCCCCTTTGGGGCAGCGGGAGACCGGGGTCTCTGAGCCATCGCTACGAACCTGTCTTGTTTGAGGAGAAGCACTATTATAAGGTCGACTACTCTAGGTTCCACAAAACATACGAAGTTCCTAACACACCATTGTGTAGTGCTCGGGACCTTGCAGAGAAGAAGTACATTCTGTCTAACGCAAACTCTTTCTGTTGGAGACGCCCGAGGGGAGAA
kir21_Frag987-1146_insertion996	CTAAATAGTGCTCGTCTCCCCTTTGGGGCCATAGCGGGAGACCGGGGTCTCTGAGCCGCTACGAACCTGTCTTGTTTGAGGAGAAGCACTATTATAAGGTCGACTACTCTAGGTTCCACAAAACATACGAAGTTCCTAACACACCATTGTGTAGTGCTCGGGACCTTGCAGAGAAGAAGTACATTCTGTCTAACGCAAACTCTTTCTGTTGGAGACGCCCGAGGGGAGAA
kir21_Frag987-1146_insertion999	CTAAATAGTGCTCGTCTCCCCTTTGGGGCCATCGCAGCGGGAGACCGGGGTCTCTGAGCTACGAACCTGTCTTGTTTGAGGAGAAGCACTATTATAAGGTCGACTACTCTAGGTTCCACAAAACATACGAAGTTCCTAACACACCATTGTGTAGTGCTCGGGACCTTGCAGAGAAGAAGTACATTCTGTCTAACGCAAACTCTTTCTGTTGGAGACGCCCGAGGGGAGAA
kir21_Frag987-1146_insertion1002	CTAAATAGTGCTCGTCTCCCCTTTGGGGCCATCGCTACAGCGGGAGACCGGGGTCTCTGAGCGAACCTGTCTTGTTTGAGGAGAAGCACTATTATAAGGTCGACTACTCTAGGTTCCACAAAACATACGAAGTTCCTAACACACCATTGTGTAGTGCTCGGGACCTTGCAGAGAAGAAGTACATTCTGTCTAACGCAAACTCTTTCTGTTGGAGACGCCCGAGGGGAGAA
kir21_Frag987-1146_insertion1005	CTAAATAGTGCTCGTCTCCCCTTTGGGGCCATCGCTACGAAAGCGGGAGACCGGGGTCTCTGAGCCCTGTCTTGTTTGAGGAGAAGCACTATTATAAGGTCGACTACTCTAGGTTCCACAAAACATACGAAGTTCCTAACACACCATTGTGTAGTGCTCGGGACCTTGCAGAGAAGAAGTACATTCTGTCTAACGCAAACTCTTTCTGTTGGAGACGCCCGAGGGGAGAA
kir21_Frag987-1146_insertion1008	CTAAATAGTGCTCGTCTCCCCTTTGGGGCCATCGCTACGAACCTAGCGGGAGACCGGGGTCTCTGAGCGTCTTGTTTGAGGAGAAGCACTATTATAAGGTCGACTACTCTAGGTTCCACAAAACATACGAAGTTCCTAACACACCATTGTGTAGTGCTCGGGACCTTGCAGAGAAGAAGTACATTCTGTCTAACGCAAACTCTTTCTGTTGGAGACGCCCGAGGGGAGAA
kir21_Frag987-1146_insertion1011	CTAAATAGTGCTCGTCTCCCCTTTGGGGCCATCGCTACGAACCTGTCAGCGGGAGACCGGGGTCTCTGAGCTTGTTTGAGGAGAAGCACTATTATAAGGTCGACTACTCTAGGTTCCACAAAACATACGAAGTTCCTAACACACCATTGTGTAGTGCTCGGGACCTTGCAGAGAAGAAGTACATTCTGTCTAACGCAAACTCTTTCTGTTGGAGACGCCCGAGGGGAGAA
kir21_Frag987-1146_insertion1014	CTAAATAGTGCTCGTCTCCCCTTTGGGGCCATCGCTACGAACCTGTCTTGAGCGGGAGACCGGGGTCTCTGAGCTTTGAGGAGAAGCACTATTATAAGGTCGACTACTCTAGGTTCCACAAAACATACGAAGTTCCTAACACACCATTGTGTAGTGCTCGGGACCTTGCAGAGAAGAAGTACATTCTGTCTAACGCAAACTCTTTCTGTTGGAGACGCCCGAGGGGAGAA
kir21_Frag987-1146_insertion1017	CTAAATAGTGCTCGTCTCCCCTTTGGGGCCATCGCTACGAACCTGTCTTGTTTAGCGGGAGACCGGGGTCTCTGAGCGAGGAGAAGCACTATTATAAGGTCGACTACTCTAGGTTCCACAAAACATACGAAGTTCCTAACACACCATTGTGTAGTGCTCGGGACCTTGCAGAGAAGAAGTACATTCTGTCTAACGCAAACTCTTTCTGTTGGAGACGCCCGAGGGGAGAA
kir21_Frag987-1146_insertion1020	CTAAATAGTGCTCGTCTCCCCTTTGGGGCCATCGCTACGAACCTGTCTTGTTTGAGAGCGGGAGACCGGGGTCTCTGAGCGAGAAGCACTATTATAAGGTCGACTACTCTAGGTTCCACAAAACATACGAAGTTCCTAACACACCATTGTGTAGTGCTCGGGACCTTGCAGAGAAGAAGTACATTCTGTCTAACGCAAACTCTTTCTGTTGGAGACGCCCGAGGGGAGAA
kir21_Frag987-1146_insertion1023	CTAAATAGTGCTCGTCTCCCCTTTGGGGCCATCGCTACGAACCTGTCTTGTTTGAGGAGAGCGGGAGACCGGGGTCTCTGAGCAAGCACTATTATAAGGTCGACTACTCTAGGTTCCACAAAACATACGAAGTTCCTAACACACCATTGTGTAGTGCTCGGGACCTTGCAGAGAAGAAGTACATTCTGTCTAACGCAAACTCTTTCTGTTGGAGACGCCCGAGGGGAGAA
kir21_Frag987-1146_insertion1026	CTAAATAGTGCTCGTCTCCCCTTTGGGGCCATCGCTACGAACCTGTCTTGTTTGAGGAGAAGAGCGGGAGACCGGGGTCTCTGAGCCACTATTATAAGGTCGACTACTCTAGGTTCCACAAAACATACGAAGTTCCTAACACACCATTGTGTAGTGCTCGGGACCTTGCAGAGAAGAAGTACATTCTGTCTAACGCAAACTCTTTCTGTTGGAGACGCCCGAGGGGAGAA
kir21_Frag987-1146_insertion1029	CTAAATAGTGCTCGTCTCCCCTTTGGGGCCATCGCTACGAACCTGTCTTGTTTGAGGAGAAGCACAGCGGGAGACCGGGGTCTCTGAGCTATTATAAGGTCGACTACTCTAGGTTCCACAAAACATACGAAGTTCCTAACACACCATTGTGTAGTGCTCGGGACCTTGCAGAGAAGAAGTACATTCTGTCTAACGCAAACTCTTTCTGTTGGAGACGCCCGAGGGGAGAA
kir21_Frag987-1146_insertion1032	CTAAATAGTGCTCGTCTCCCCTTTGGGGCCATCGCTACGAACCTGTCTTGTTTGAGGAGAAGCACTATAGCGGGAGACCGGGGTCTCTGAGCTATAAGGTCGACTACTCTAGGTTCCACAAAACATACGAAGTTCCTAACACACCATTGTGTAGTGCTCGGGACCTTGCAGAGAAGAAGTACATTCTGTCTAACGCAAACTCTTTCTGTTGGAGACGCCCGAGGGGAGAA
kir21_Frag987-1146_insertion1035	CTAAATAGTGCTCGTCTCCCCTTTGGGGCCATCGCTACGAACCTGTCTTGTTTGAGGAGAAGCACTATTATAGCGGGAGACCGGGGTCTCTGAGCAAGGTCGACTACTCTAGGTTCCACAAAACATACGAAGTTCCTAACACACCATTGTGTAGTGCTCGGGACCTTGCAGAGAAGAAGTACATTCTGTCTAACGCAAACTCTTTCTGTTGGAGACGCCCGAGGGGAGAA
kir21_Frag987-1146_insertion1038	CTAAATAGTGCTCGTCTCCCCTTTGGGGCCATCGCTACGAACCTGTCTTGTTTGAGGAGAAGCACTATTATAAGAGCGGGAGACCGGGGTCTCTGAGCGTCGACTACTCTAGGTTCCACAAAACATACGAAGTTCCTAACACACCATTGTGTAGTGCTCGGGACCTTGCAGAGAAGAAGTACATTCTGTCTAACGCAAACTCTTTCTGTTGGAGACGCCCGAGGGGAGAA
kir21_Frag987-1146_insertion1041	CTAAATAGTGCTCGTCTCCCCTTTGGGGCCATCGCTACGAACCTGTCTTGTTTGAGGAGAAGCACTATTATAAGGTCAGCGGGAGACCGGGGTCTCTGAGCGACTACTCTAGGTTCCACAAAACATACGAAGTTCCTAACACACCATTGTGTAGTGCTCGGGACCTTGCAGAGAAGAAGTACATTCTGTCTAACGCAAACTCTTTCTGTTGGAGACGCCCGAGGGGAGAA
kir21_Frag987-1146_insertion1044	CTAAATAGTGCTCGTCTCCCCTTTGGGGCCATCGCTACGAACCTGTCTTGTTTGAGGAGAAGCACTATTATAAGGTCGACAGCGGGAGACCGGGGTCTCTGAGCTACTCTAGGTTCCACAAAACATACGAAGTTCCTAACACACCATTGTGTAGTGCTCGGGACCTTGCAGAGAAGAAGTACATTCTGTCTAACGCAAACTCTTTCTGTTGGAGACGCCCGAGGGGAGAA
kir21_Frag987-1146_insertion1047	CTAAATAGTGCTCGTCTCCCCTTTGGGGCCATCGCTACGAACCTGTCTTGTTTGAGGAGAAGCACTATTATAAGGTCGACTACAGCGGGAGACCGGGGTCTCTGAGCTCTAGGTTCCACAAAACATACGAAGTTCCTAACACACCATTGTGTAGTGCTCGGGACCTTGCAGAGAAGAAGTACATTCTGTCTAACGCAAACTCTTTCTGTTGGAGACGCCCGAGGGGAGAA
kir21_Frag987-1146_insertion1050	CTAAATAGTGCTCGTCTCCCCTTTGGGGCCATCGCTACGAACCTGTCTTGTTTGAGGAGAAGCACTATTATAAGGTCGACTACTCTAGCGGGAGACCGGGGTCTCTGAGCAGGTTCCACAAAACATACGAAGTTCCTAACACACCATTGTGTAGTGCTCGGGACCTTGCAGAGAAGAAGTACATTCTGTCTAACGCAAACTCTTTCTGTTGGAGACGCCCGAGGGGAGAA
kir21_Frag987-1146_insertion1053	CTAAATAGTGCTCGTCTCCCCTTTGGGGCCATCGCTACGAACCTGTCTTGTTTGAGGAGAAGCACTATTATAAGGTCGACTACTCTAGGAGCGGGAGACCGGGGTCTCTGAGCTTCCACAAAACATACGAAGTTCCTAACACACCATTGTGTAGTGCTCGGGACCTTGCAGAGAAGAAGTACATTCTGTCTAACGCAAACTCTTTCTGTTGGAGACGCCCGAGGGGAGAA
kir21_Frag987-1146_insertion1056	CTAAATAGTGCTCGTCTCCCCTTTGGGGCCATCGCTACGAACCTGTCTTGTTTGAGGAGAAGCACTATTATAAGGTCGACTACTCTAGGTTCAGCGGGAGACCGGGGTCTCTGAGCCACAAAACATACGAAGTTCCTAACACACCATTGTGTAGTGCTCGGGACCTTGCAGAGAAGAAGTACATTCTGTCTAACGCAAACTCTTTCTGTTGGAGACGCCCGAGGGGAGAA
kir21_Frag987-1146_insertion1059	CTAAATAGTGCTCGTCTCCCCTTTGGGGCCATCGCTACGAACCTGTCTTGTTTGAGGAGAAGCACTATTATAAGGTCGACTACTCTAGGTTCCACAGCGGGAGACCGGGGTCTCTGAGCAAAACATACGAAGTTCCTAACACACCATTGTGTAGTGCTCGGGACCTTGCAGAGAAGAAGTACATTCTGTCTAACGCAAACTCTTTCTGTTGGAGACGCCCGAGGGGAGAA
kir21_Frag987-1146_insertion1062	CTAAATAGTGCTCGTCTCCCCTTTGGGGCCATCGCTACGAACCTGTCTTGTTTGAGGAGAAGCACTATTATAAGGTCGACTACTCTAGGTTCCACAAAAGCGGGAGACCGGGGTCTCTGAGCACATACGAAGTTCCTAACACACCATTGTGTAGTGCTCGGGACCTTGCAGAGAAGAAGTACATTCTGTCTAACGCAAACTCTTTCTGTTGGAGACGCCCGAGGGGAGAA
kir21_Frag987-1146_insertion1065	CTAAATAGTGCTCGTCTCCCCTTTGGGGCCATCGCTACGAACCTGTCTTGTTTGAGGAGAAGCACTATTATAAGGTCGACTACTCTAGGTTCCACAAAACAAGCGGGAGACCGGGGTCTCTGAGCTACGAAGTTCCTAACACACCATTGTGTAGTGCTCGGGACCTTGCAGAGAAGAAGTACATTCTGTCTAACGCAAACTCTTTCTGTTGGAGACGCCCGAGGGGAGAA
kir21_Frag987-1146_insertion1068	CTAAATAGTGCTCGTCTCCCCTTTGGGGCCATCGCTACGAACCTGTCTTGTTTGAGGAGAAGCACTATTATAAGGTCGACTACTCTAGGTTCCACAAAACATACAGCGGGAGACCGGGGTCTCTGAGCGAAGTTCCTAACACACCATTGTGTAGTGCTCGGGACCTTGCAGAGAAGAAGTACATTCTGTCTAACGCAAACTCTTTCTGTTGGAGACGCCCGAGGGGAGAA
kir21_Frag987-1146_insertion1071	CTAAATAGTGCTCGTCTCCCCTTTGGGGCCATCGCTACGAACCTGTCTTGTTTGAGGAGAAGCACTATTATAAGGTCGACTACTCTAGGTTCCACAAAACATACGAAAGCGGGAGACCGGGGTCTCTGAGCGTTCCTAACACACCATTGTGTAGTGCTCGGGACCTTGCAGAGAAGAAGTACATTCTGTCTAACGCAAACTCTTTCTGTTGGAGACGCCCGAGGGGAGAA
kir21_Frag987-1146_insertion1074	CTAAATAGTGCTCGTCTCCCCTTTGGGGCCATCGCTACGAACCTGTCTTGTTTGAGGAGAAGCACTATTATAAGGTCGACTACTCTAGGTTCCACAAAACATACGAAGTTAGCGGGAGACCGGGGTCTCTGAGCCCTAACACACCATTGTGTAGTGCTCGGGACCTTGCAGAGAAGAAGTACATTCTGTCTAACGCAAACTCTTTCTGTTGGAGACGCCCGAGGGGAGAA
kir21_Frag987-1146_insertion1077	CTAAATAGTGCTCGTCTCCCCTTTGGGGCCATCGCTACGAACCTGTCTTGTTTGAGGAGAAGCACTATTATAAGGTCGACTACTCTAGGTTCCACAAAACATACGAAGTTCCTAGCGGGAGACCGGGGTCTCTGAGCAACACACCATTGTGTAGTGCTCGGGACCTTGCAGAGAAGAAGTACATTCTGTCTAACGCAAACTCTTTCTGTTGGAGACGCCCGAGGGGAGAA
kir21_Frag987-1146_insertion1080	CTAAATAGTGCTCGTCTCCCCTTTGGGGCCATCGCTACGAACCTGTCTTGTTTGAGGAGAAGCACTATTATAAGGTCGACTACTCTAGGTTCCACAAAACATACGAAGTTCCTAACAGCGGGAGACCGGGGTCTCTGAGCACACCATTGTGTAGTGCTCGGGACCTTGCAGAGAAGAAGTACATTCTGTCTAACGCAAACTCTTTCTGTTGGAGACGCCCGAGGGGAGAA
kir21_Frag987-1146_insertion1083	CTAAATAGTGCTCGTCTCCCCTTTGGGGCCATCGCTACGAACCTGTCTTGTTTGAGGAGAAGCACTATTATAAGGTCGACTACTCTAGGTTCCACAAAACATACGAAGTTCCTAACACAAGCGGGAGACCGGGGTCTCTGAGCCCATTGTGTAGTGCTCGGGACCTTGCAGAGAAGAAGTACATTCTGTCTAACGCAAACTCTTTCTGTTGGAGACGCCCGAGGGGAGAA
kir21_Frag987-1146_insertion1086	CTAAATAGTGCTCGTCTCCCCTTTGGGGCCATCGCTACGAACCTGTCTTGTTTGAGGAGAAGCACTATTATAAGGTCGACTACTCTAGGTTCCACAAAACATACGAAGTTCCTAACACACCAAGCGGGAGACCGGGGTCTCTGAGCTTGTGTAGTGCTCGGGACCTTGCAGAGAAGAAGTACATTCTGTCTAACGCAAACTCTTTCTGTTGGAGACGCCCGAGGGGAGAA
kir21_Frag987-1146_insertion1089	CTAAATAGTGCTCGTCTCCCCTTTGGGGCCATCGCTACGAACCTGTCTTGTTTGAGGAGAAGCACTATTATAAGGTCGACTACTCTAGGTTCCACAAAACATACGAAGTTCCTAACACACCATTGAGCGGGAGACCGGGGTCTCTGAGCTGTAGTGCTCGGGACCTTGCAGAGAAGAAGTACATTCTGTCTAACGCAAACTCTTTCTGTTGGAGACGCCCGAGGGGAGAA
kir21_Frag987-1146_insertion1092	CTAAATAGTGCTCGTCTCCCCTTTGGGGCCATCGCTACGAACCTGTCTTGTTTGAGGAGAAGCACTATTATAAGGTCGACTACTCTAGGTTCCACAAAACATACGAAGTTCCTAACACACCATTGTGTAGCGGGAGACCGGGGTCTCTGAGCAGTGCTCGGGACCTTGCAGAGAAGAAGTACATTCTGTCTAACGCAAACTCTTTCTGTTGGAGACGCCCGAGGGGAGAA
kir21_Frag987-1146_insertion1095	CTAAATAGTGCTCGTCTCCCCTTTGGGGCCATCGCTACGAACCTGTCTTGTTTGAGGAGAAGCACTATTATAAGGTCGACTACTCTAGGTTCCACAAAACATACGAAGTTCCTAACACACCATTGTGTAGTAGCGGGAGACCGGGGTCTCTGAGCGCTCGGGACCTTGCAGAGAAGAAGTACATTCTGTCTAACGCAAACTCTTTCTGTTGGAGACGCCCGAGGGGAGAA
kir21_Frag987-1146_insertion1098	CTAAATAGTGCTCGTCTCCCCTTTGGGGCCATCGCTACGAACCTGTCTTGTTTGAGGAGAAGCACTATTATAAGGTCGACTACTCTAGGTTCCACAAAACATACGAAGTTCCTAACACACCATTGTGTAGTGCTAGCGGGAGACCGGGGTCTCTGAGCCGGGACCTTGCAGAGAAGAAGTACATTCTGTCTAACGCAAACTCTTTCTGTTGGAGACGCCCGAGGGGAGAA
kir21_Frag987-1146_insertion1101	CTAAATAGTGCTCGTCTCCCCTTTGGGGCCATCGCTACGAACCTGTCTTGTTTGAGGAGAAGCACTATTATAAGGTCGACTACTCTAGGTTCCACAAAACATACGAAGTTCCTAACACACCATTGTGTAGTGCTCGGAGCGGGAGACCGGGGTCTCTGAGCGACCTTGCAGAGAAGAAGTACATTCTGTCTAACGCAAACTCTTTCTGTTGGAGACGCCCGAGGGGAGAA
kir21_Frag987-1146_insertion1104	CTAAATAGTGCTCGTCTCCCCTTTGGGGCCATCGCTACGAACCTGTCTTGTTTGAGGAGAAGCACTATTATAAGGTCGACTACTCTAGGTTCCACAAAACATACGAAGTTCCTAACACACCATTGTGTAGTGCTCGGGACAGCGGGAGACCGGGGTCTCTGAGCCTTGCAGAGAAGAAGTACATTCTGTCTAACGCAAACTCTTTCTGTTGGAGACGCCCGAGGGGAGAA
kir21_Frag987-1146_insertion1107	CTAAATAGTGCTCGTCTCCCCTTTGGGGCCATCGCTACGAACCTGTCTTGTTTGAGGAGAAGCACTATTATAAGGTCGACTACTCTAGGTTCCACAAAACATACGAAGTTCCTAACACACCATTGTGTAGTGCTCGGGACCTTAGCGGGAGACCGGGGTCTCTGAGCGCAGAGAAGAAGTACATTCTGTCTAACGCAAACTCTTTCTGTTGGAGACGCCCGAGGGGAGAA
kir21_Frag987-1146_insertion1110	CTAAATAGTGCTCGTCTCCCCTTTGGGGCCATCGCTACGAACCTGTCTTGTTTGAGGAGAAGCACTATTATAAGGTCGACTACTCTAGGTTCCACAAAACATACGAAGTTCCTAACACACCATTGTGTAGTGCTCGGGACCTTGCAAGCGGGAGACCGGGGTCTCTGAGCGAGAAGAAGTACATTCTGTCTAACGCAAACTCTTTCTGTTGGAGACGCCCGAGGGGAGAA
kir21_Frag987-1146_insertion1113	CTAAATAGTGCTCGTCTCCCCTTTGGGGCCATCGCTACGAACCTGTCTTGTTTGAGGAGAAGCACTATTATAAGGTCGACTACTCTAGGTTCCACAAAACATACGAAGTTCCTAACACACCATTGTGTAGTGCTCGGGACCTTGCAGAGAGCGGGAGACCGGGGTCTCTGAGCAAGAAGTACATTCTGTCTAACGCAAACTCTTTCTGTTGGAGACGCCCGAGGGGAGAA
kir21_Frag987-1146_insertion1116	CTAAATAGTGCTCGTCTCCCCTTTGGGGCCATCGCTACGAACCTGTCTTGTTTGAGGAGAAGCACTATTATAAGGTCGACTACTCTAGGTTCCACAAAACATACGAAGTTCCTAACACACCATTGTGTAGTGCTCGGGACCTTGCAGAGAAGAGCGGGAGACCGGGGTCTCTGAGCAAGTACATTCTGTCTAACGCAAACTCTTTCTGTTGGAGACGCCCGAGGGGAGAA
kir21_Frag987-1146_insertion1119	CTAAATAGTGCTCGTCTCCCCTTTGGGGCCATCGCTACGAACCTGTCTTGTTTGAGGAGAAGCACTATTATAAGGTCGACTACTCTAGGTTCCACAAAACATACGAAGTTCCTAACACACCATTGTGTAGTGCTCGGGACCTTGCAGAGAAGAAGAGCGGGAGACCGGGGTCTCTGAGCTACATTCTGTCTAACGCAAACTCTTTCTGTTGGAGACGCCCGAGGGGAGAA
kir21_Frag987-1146_insertion1122	CTAAATAGTGCTCGTCTCCCCTTTGGGGCCATCGCTACGAACCTGTCTTGTTTGAGGAGAAGCACTATTATAAGGTCGACTACTCTAGGTTCCACAAAACATACGAAGTTCCTAACACACCATTGTGTAGTGCTCGGGACCTTGCAGAGAAGAAGTACAGCGGGAGACCGGGGTCTCTGAGCATTCTGTCTAACGCAAACTCTTTCTGTTGGAGACGCCCGAGGGGAGAA
kir21_Frag987-1146_insertion1125	CTAAATAGTGCTCGTCTCCCCTTTGGGGCCATCGCTACGAACCTGTCTTGTTTGAGGAGAAGCACTATTATAAGGTCGACTACTCTAGGTTCCACAAAACATACGAAGTTCCTAACACACCATTGTGTAGTGCTCGGGACCTTGCAGAGAAGAAGTACATTAGCGGGAGACCGGGGTCTCTGAGCCTGTCTAACGCAAACTCTTTCTGTTGGAGACGCCCGAGGGGAGAA
kir21_Frag987-1146_insertion1128	CTAAATAGTGCTCGTCTCCCCTTTGGGGCCATCGCTACGAACCTGTCTTGTTTGAGGAGAAGCACTATTATAAGGTCGACTACTCTAGGTTCCACAAAACATACGAAGTTCCTAACACACCATTGTGTAGTGCTCGGGACCTTGCAGAGAAGAAGTACATTCTGAGCGGGAGACCGGGGTCTCTGAGCTCTAACGCAAACTCTTTCTGTTGGAGACGCCCGAGGGGAGAA
kir21_Frag987-1146_insertion1131	CTAAATAGTGCTCGTCTCCCCTTTGGGGCCATCGCTACGAACCTGTCTTGTTTGAGGAGAAGCACTATTATAAGGTCGACTACTCTAGGTTCCACAAAACATACGAAGTTCCTAACACACCATTGTGTAGTGCTCGGGACCTTGCAGAGAAGAAGTACATTCTGTCTAGCGGGAGACCGGGGTCTCTGAGCAACGCAAACTCTTTCTGTTGGAGACGCCCGAGGGGAGAA
kir21_Frag987-1146_insertion1134	CTAAATAGTGCTCGTCTCCCCTTTGGGGCCATCGCTACGAACCTGTCTTGTTTGAGGAGAAGCACTATTATAAGGTCGACTACTCTAGGTTCCACAAAACATACGAAGTTCCTAACACACCATTGTGTAGTGCTCGGGACCTTGCAGAGAAGAAGTACATTCTGTCTAACAGCGGGAGACCGGGGTCTCTGAGCGCAAACTCTTTCTGTTGGAGACGCCCGAGGGGAGAA
kir21_Frag987-1146_insertion1137	CTAAATAGTGCTCGTCTCCCCTTTGGGGCCATCGCTACGAACCTGTCTTGTTTGAGGAGAAGCACTATTATAAGGTCGACTACTCTAGGTTCCACAAAACATACGAAGTTCCTAACACACCATTGTGTAGTGCTCGGGACCTTGCAGAGAAGAAGTACATTCTGTCTAACGCAAGCGGGAGACCGGGGTCTCTGAGCAACTCTTTCTGTTGGAGACGCCCGAGGGGAGAA
kir21_Frag987-1146_insertion1140	CTAAATAGTGCTCGTCTCCCCTTTGGGGCCATCGCTACGAACCTGTCTTGTTTGAGGAGAAGCACTATTATAAGGTCGACTACTCTAGGTTCCACAAAACATACGAAGTTCCTAACACACCATTGTGTAGTGCTCGGGACCTTGCAGAGAAGAAGTACATTCTGTCTAACGCAAACAGCGGGAGACCGGGGTCTCTGAGCTCTTTCTGTTGGAGACGCCCGAGGGGAGAA
kir21_Frag987-1146_insertion1143	CTAAATAGTGCTCGTCTCCCCTTTGGGGCCATCGCTACGAACCTGTCTTGTTTGAGGAGAAGCACTATTATAAGGTCGACTACTCTAGGTTCCACAAAACATACGAAGTTCCTAACACACCATTGTGTAGTGCTCGGGACCTTGCAGAGAAGAAGTACATTCTGTCTAACGCAAACTCTAGCGGGAGACCGGGGTCTCTGAGCTTCTGTTGGAGACGCCCGAGGGGAGAA
kir21_Frag987-1146_insertion1146	CTAAATAGTGCTCGTCTCCCCTTTGGGGCCATCGCTACGAACCTGTCTTGTTTGAGGAGAAGCACTATTATAAGGTCGACTACTCTAGGTTCCACAAAACATACGAAGTTCCTAACACACCATTGTGTAGTGCTCGGGACCTTGCAGAGAAGAAGTACATTCTGTCTAACGCAAACTCTTTCAGCGGGAGACCGGGGTCTCTGAGCTGTTGGAGACGCCCGAGGGGAGAA
kir21_Frag1149-1308_insertion1149	TGATCTAAGTGGCGTCTCCCTGTAGCGGGAGACCGGGGTCTCTGAGCTACGAGAACGAAGTAGCTCTCACATCAAAGGAAGAAGAAGAAGATTCAGAGAACGGAGTTCCCGAGTCAACCAGCACCGACTCTCCTCCCGGCATTGACCTGCACAACCAAGCCAGTGTCCCCCTGGAGCCTAGACCTCTTCGGAGAGAGAGTGAAATCGCTGGGAGACGCGGAATAACTGTG
kir21_Frag1149-1308_insertion1152	TGATCTAAGTGGCGTCTCCCTGTTACAGCGGGAGACCGGGGTCTCTGAGCGAGAACGAAGTAGCTCTCACATCAAAGGAAGAAGAAGAAGATTCAGAGAACGGAGTTCCCGAGTCAACCAGCACCGACTCTCCTCCCGGCATTGACCTGCACAACCAAGCCAGTGTCCCCCTGGAGCCTAGACCTCTTCGGAGAGAGAGTGAAATCGCTGGGAGACGCGGAATAACTGTG
kir21_Frag1149-1308_insertion1155	TGATCTAAGTGGCGTCTCCCTGTTACGAGAGCGGGAGACCGGGGTCTCTGAGCAACGAAGTAGCTCTCACATCAAAGGAAGAAGAAGAAGATTCAGAGAACGGAGTTCCCGAGTCAACCAGCACCGACTCTCCTCCCGGCATTGACCTGCACAACCAAGCCAGTGTCCCCCTGGAGCCTAGACCTCTTCGGAGAGAGAGTGAAATCGCTGGGAGACGCGGAATAACTGTG
kir21_Frag1149-1308_insertion1158	TGATCTAAGTGGCGTCTCCCTGTTACGAGAACAGCGGGAGACCGGGGTCTCTGAGCGAAGTAGCTCTCACATCAAAGGAAGAAGAAGAAGATTCAGAGAACGGAGTTCCCGAGTCAACCAGCACCGACTCTCCTCCCGGCATTGACCTGCACAACCAAGCCAGTGTCCCCCTGGAGCCTAGACCTCTTCGGAGAGAGAGTGAAATCGCTGGGAGACGCGGAATAACTGTG
kir21_Frag1149-1308_insertion1161	TGATCTAAGTGGCGTCTCCCTGTTACGAGAACGAAAGCGGGAGACCGGGGTCTCTGAGCGTAGCTCTCACATCAAAGGAAGAAGAAGAAGATTCAGAGAACGGAGTTCCCGAGTCAACCAGCACCGACTCTCCTCCCGGCATTGACCTGCACAACCAAGCCAGTGTCCCCCTGGAGCCTAGACCTCTTCGGAGAGAGAGTGAAATCGCTGGGAGACGCGGAATAACTGTG
kir21_Frag1149-1308_insertion1164	TGATCTAAGTGGCGTCTCCCTGTTACGAGAACGAAGTAAGCGGGAGACCGGGGTCTCTGAGCGCTCTCACATCAAAGGAAGAAGAAGAAGATTCAGAGAACGGAGTTCCCGAGTCAACCAGCACCGACTCTCCTCCCGGCATTGACCTGCACAACCAAGCCAGTGTCCCCCTGGAGCCTAGACCTCTTCGGAGAGAGAGTGAAATCGCTGGGAGACGCGGAATAACTGTG
kir21_Frag1149-1308_insertion1167	TGATCTAAGTGGCGTCTCCCTGTTACGAGAACGAAGTAGCTAGCGGGAGACCGGGGTCTCTGAGCCTCACATCAAAGGAAGAAGAAGAAGATTCAGAGAACGGAGTTCCCGAGTCAACCAGCACCGACTCTCCTCCCGGCATTGACCTGCACAACCAAGCCAGTGTCCCCCTGGAGCCTAGACCTCTTCGGAGAGAGAGTGAAATCGCTGGGAGACGCGGAATAACTGTG
kir21_Frag1149-1308_insertion1170	TGATCTAAGTGGCGTCTCCCTGTTACGAGAACGAAGTAGCTCTCAGCGGGAGACCGGGGTCTCTGAGCACATCAAAGGAAGAAGAAGAAGATTCAGAGAACGGAGTTCCCGAGTCAACCAGCACCGACTCTCCTCCCGGCATTGACCTGCACAACCAAGCCAGTGTCCCCCTGGAGCCTAGACCTCTTCGGAGAGAGAGTGAAATCGCTGGGAGACGCGGAATAACTGTG
kir21_Frag1149-1308_insertion1173	TGATCTAAGTGGCGTCTCCCTGTTACGAGAACGAAGTAGCTCTCACAAGCGGGAGACCGGGGTCTCTGAGCTCAAAGGAAGAAGAAGAAGATTCAGAGAACGGAGTTCCCGAGTCAACCAGCACCGACTCTCCTCCCGGCATTGACCTGCACAACCAAGCCAGTGTCCCCCTGGAGCCTAGACCTCTTCGGAGAGAGAGTGAAATCGCTGGGAGACGCGGAATAACTGTG
kir21_Frag1149-1308_insertion1176	TGATCTAAGTGGCGTCTCCCTGTTACGAGAACGAAGTAGCTCTCACATCAAGCGGGAGACCGGGGTCTCTGAGCAAGGAAGAAGAAGAAGATTCAGAGAACGGAGTTCCCGAGTCAACCAGCACCGACTCTCCTCCCGGCATTGACCTGCACAACCAAGCCAGTGTCCCCCTGGAGCCTAGACCTCTTCGGAGAGAGAGTGAAATCGCTGGGAGACGCGGAATAACTGTG
kir21_Frag1149-1308_insertion1179	TGATCTAAGTGGCGTCTCCCTGTTACGAGAACGAAGTAGCTCTCACATCAAAGAGCGGGAGACCGGGGTCTCTGAGCGAAGAAGAAGAAGATTCAGAGAACGGAGTTCCCGAGTCAACCAGCACCGACTCTCCTCCCGGCATTGACCTGCACAACCAAGCCAGTGTCCCCCTGGAGCCTAGACCTCTTCGGAGAGAGAGTGAAATCGCTGGGAGACGCGGAATAACTGTG
kir21_Frag1149-1308_insertion1182	TGATCTAAGTGGCGTCTCCCTGTTACGAGAACGAAGTAGCTCTCACATCAAAGGAAAGCGGGAGACCGGGGTCTCTGAGCGAAGAAGAAGATTCAGAGAACGGAGTTCCCGAGTCAACCAGCACCGACTCTCCTCCCGGCATTGACCTGCACAACCAAGCCAGTGTCCCCCTGGAGCCTAGACCTCTTCGGAGAGAGAGTGAAATCGCTGGGAGACGCGGAATAACTGTG
kir21_Frag1149-1308_insertion1185	TGATCTAAGTGGCGTCTCCCTGTTACGAGAACGAAGTAGCTCTCACATCAAAGGAAGAAAGCGGGAGACCGGGGTCTCTGAGCGAAGAAGATTCAGAGAACGGAGTTCCCGAGTCAACCAGCACCGACTCTCCTCCCGGCATTGACCTGCACAACCAAGCCAGTGTCCCCCTGGAGCCTAGACCTCTTCGGAGAGAGAGTGAAATCGCTGGGAGACGCGGAATAACTGTG
kir21_Frag1149-1308_insertion1188	TGATCTAAGTGGCGTCTCCCTGTTACGAGAACGAAGTAGCTCTCACATCAAAGGAAGAAGAAAGCGGGAGACCGGGGTCTCTGAGCGAAGATTCAGAGAACGGAGTTCCCGAGTCAACCAGCACCGACTCTCCTCCCGGCATTGACCTGCACAACCAAGCCAGTGTCCCCCTGGAGCCTAGACCTCTTCGGAGAGAGAGTGAAATCGCTGGGAGACGCGGAATAACTGTG
kir21_Frag1149-1308_insertion1191	TGATCTAAGTGGCGTCTCCCTGTTACGAGAACGAAGTAGCTCTCACATCAAAGGAAGAAGAAGAAAGCGGGAGACCGGGGTCTCTGAGCGATTCAGAGAACGGAGTTCCCGAGTCAACCAGCACCGACTCTCCTCCCGGCATTGACCTGCACAACCAAGCCAGTGTCCCCCTGGAGCCTAGACCTCTTCGGAGAGAGAGTGAAATCGCTGGGAGACGCGGAATAACTGTG
kir21_Frag1149-1308_insertion1194	TGATCTAAGTGGCGTCTCCCTGTTACGAGAACGAAGTAGCTCTCACATCAAAGGAAGAAGAAGAAGATAGCGGGAGACCGGGGTCTCTGAGCTCAGAGAACGGAGTTCCCGAGTCAACCAGCACCGACTCTCCTCCCGGCATTGACCTGCACAACCAAGCCAGTGTCCCCCTGGAGCCTAGACCTCTTCGGAGAGAGAGTGAAATCGCTGGGAGACGCGGAATAACTGTG
kir21_Frag1149-1308_insertion1197	TGATCTAAGTGGCGTCTCCCTGTTACGAGAACGAAGTAGCTCTCACATCAAAGGAAGAAGAAGAAGATTCAAGCGGGAGACCGGGGTCTCTGAGCGAGAACGGAGTTCCCGAGTCAACCAGCACCGACTCTCCTCCCGGCATTGACCTGCACAACCAAGCCAGTGTCCCCCTGGAGCCTAGACCTCTTCGGAGAGAGAGTGAAATCGCTGGGAGACGCGGAATAACTGTG
kir21_Frag1149-1308_insertion1200	TGATCTAAGTGGCGTCTCCCTGTTACGAGAACGAAGTAGCTCTCACATCAAAGGAAGAAGAAGAAGATTCAGAGAGCGGGAGACCGGGGTCTCTGAGCAACGGAGTTCCCGAGTCAACCAGCACCGACTCTCCTCCCGGCATTGACCTGCACAACCAAGCCAGTGTCCCCCTGGAGCCTAGACCTCTTCGGAGAGAGAGTGAAATCGCTGGGAGACGCGGAATAACTGTG
kir21_Frag1149-1308_insertion1203	TGATCTAAGTGGCGTCTCCCTGTTACGAGAACGAAGTAGCTCTCACATCAAAGGAAGAAGAAGAAGATTCAGAGAACAGCGGGAGACCGGGGTCTCTGAGCGGAGTTCCCGAGTCAACCAGCACCGACTCTCCTCCCGGCATTGACCTGCACAACCAAGCCAGTGTCCCCCTGGAGCCTAGACCTCTTCGGAGAGAGAGTGAAATCGCTGGGAGACGCGGAATAACTGTG
kir21_Frag1149-1308_insertion1206	TGATCTAAGTGGCGTCTCCCTGTTACGAGAACGAAGTAGCTCTCACATCAAAGGAAGAAGAAGAAGATTCAGAGAACGGAAGCGGGAGACCGGGGTCTCTGAGCGTTCCCGAGTCAACCAGCACCGACTCTCCTCCCGGCATTGACCTGCACAACCAAGCCAGTGTCCCCCTGGAGCCTAGACCTCTTCGGAGAGAGAGTGAAATCGCTGGGAGACGCGGAATAACTGTG
kir21_Frag1149-1308_insertion1209	TGATCTAAGTGGCGTCTCCCTGTTACGAGAACGAAGTAGCTCTCACATCAAAGGAAGAAGAAGAAGATTCAGAGAACGGAGTTAGCGGGAGACCGGGGTCTCTGAGCCCCGAGTCAACCAGCACCGACTCTCCTCCCGGCATTGACCTGCACAACCAAGCCAGTGTCCCCCTGGAGCCTAGACCTCTTCGGAGAGAGAGTGAAATCGCTGGGAGACGCGGAATAACTGTG
kir21_Frag1149-1308_insertion1212	TGATCTAAGTGGCGTCTCCCTGTTACGAGAACGAAGTAGCTCTCACATCAAAGGAAGAAGAAGAAGATTCAGAGAACGGAGTTCCCAGCGGGAGACCGGGGTCTCTGAGCGAGTCAACCAGCACCGACTCTCCTCCCGGCATTGACCTGCACAACCAAGCCAGTGTCCCCCTGGAGCCTAGACCTCTTCGGAGAGAGAGTGAAATCGCTGGGAGACGCGGAATAACTGTG
kir21_Frag1149-1308_insertion1215	TGATCTAAGTGGCGTCTCCCTGTTACGAGAACGAAGTAGCTCTCACATCAAAGGAAGAAGAAGAAGATTCAGAGAACGGAGTTCCCGAGAGCGGGAGACCGGGGTCTCTGAGCTCAACCAGCACCGACTCTCCTCCCGGCATTGACCTGCACAACCAAGCCAGTGTCCCCCTGGAGCCTAGACCTCTTCGGAGAGAGAGTGAAATCGCTGGGAGACGCGGAATAACTGTG
kir21_Frag1149-1308_insertion1218	TGATCTAAGTGGCGTCTCCCTGTTACGAGAACGAAGTAGCTCTCACATCAAAGGAAGAAGAAGAAGATTCAGAGAACGGAGTTCCCGAGTCAAGCGGGAGACCGGGGTCTCTGAGCACCAGCACCGACTCTCCTCCCGGCATTGACCTGCACAACCAAGCCAGTGTCCCCCTGGAGCCTAGACCTCTTCGGAGAGAGAGTGAAATCGCTGGGAGACGCGGAATAACTGTG
kir21_Frag1149-1308_insertion1221	TGATCTAAGTGGCGTCTCCCTGTTACGAGAACGAAGTAGCTCTCACATCAAAGGAAGAAGAAGAAGATTCAGAGAACGGAGTTCCCGAGTCAACCAGCGGGAGACCGGGGTCTCTGAGCAGCACCGACTCTCCTCCCGGCATTGACCTGCACAACCAAGCCAGTGTCCCCCTGGAGCCTAGACCTCTTCGGAGAGAGAGTGAAATCGCTGGGAGACGCGGAATAACTGTG
kir21_Frag1149-1308_insertion1224	TGATCTAAGTGGCGTCTCCCTGTTACGAGAACGAAGTAGCTCTCACATCAAAGGAAGAAGAAGAAGATTCAGAGAACGGAGTTCCCGAGTCAACCAGCAGCGGGAGACCGGGGTCTCTGAGCACCGACTCTCCTCCCGGCATTGACCTGCACAACCAAGCCAGTGTCCCCCTGGAGCCTAGACCTCTTCGGAGAGAGAGTGAAATCGCTGGGAGACGCGGAATAACTGTG
kir21_Frag1149-1308_insertion1227	TGATCTAAGTGGCGTCTCCCTGTTACGAGAACGAAGTAGCTCTCACATCAAAGGAAGAAGAAGAAGATTCAGAGAACGGAGTTCCCGAGTCAACCAGCACCAGCGGGAGACCGGGGTCTCTGAGCGACTCTCCTCCCGGCATTGACCTGCACAACCAAGCCAGTGTCCCCCTGGAGCCTAGACCTCTTCGGAGAGAGAGTGAAATCGCTGGGAGACGCGGAATAACTGTG
kir21_Frag1149-1308_insertion1230	TGATCTAAGTGGCGTCTCCCTGTTACGAGAACGAAGTAGCTCTCACATCAAAGGAAGAAGAAGAAGATTCAGAGAACGGAGTTCCCGAGTCAACCAGCACCGACAGCGGGAGACCGGGGTCTCTGAGCTCTCCTCCCGGCATTGACCTGCACAACCAAGCCAGTGTCCCCCTGGAGCCTAGACCTCTTCGGAGAGAGAGTGAAATCGCTGGGAGACGCGGAATAACTGTG
kir21_Frag1149-1308_insertion1233	TGATCTAAGTGGCGTCTCCCTGTTACGAGAACGAAGTAGCTCTCACATCAAAGGAAGAAGAAGAAGATTCAGAGAACGGAGTTCCCGAGTCAACCAGCACCGACTCTAGCGGGAGACCGGGGTCTCTGAGCCCTCCCGGCATTGACCTGCACAACCAAGCCAGTGTCCCCCTGGAGCCTAGACCTCTTCGGAGAGAGAGTGAAATCGCTGGGAGACGCGGAATAACTGTG
kir21_Frag1149-1308_insertion1236	TGATCTAAGTGGCGTCTCCCTGTTACGAGAACGAAGTAGCTCTCACATCAAAGGAAGAAGAAGAAGATTCAGAGAACGGAGTTCCCGAGTCAACCAGCACCGACTCTCCTAGCGGGAGACCGGGGTCTCTGAGCCCCGGCATTGACCTGCACAACCAAGCCAGTGTCCCCCTGGAGCCTAGACCTCTTCGGAGAGAGAGTGAAATCGCTGGGAGACGCGGAATAACTGTG
kir21_Frag1149-1308_insertion1239	TGATCTAAGTGGCGTCTCCCTGTTACGAGAACGAAGTAGCTCTCACATCAAAGGAAGAAGAAGAAGATTCAGAGAACGGAGTTCCCGAGTCAACCAGCACCGACTCTCCTCCCAGCGGGAGACCGGGGTCTCTGAGCGGCATTGACCTGCACAACCAAGCCAGTGTCCCCCTGGAGCCTAGACCTCTTCGGAGAGAGAGTGAAATCGCTGGGAGACGCGGAATAACTGTG
kir21_Frag1149-1308_insertion1242	TGATCTAAGTGGCGTCTCCCTGTTACGAGAACGAAGTAGCTCTCACATCAAAGGAAGAAGAAGAAGATTCAGAGAACGGAGTTCCCGAGTCAACCAGCACCGACTCTCCTCCCGGCAGCGGGAGACCGGGGTCTCTGAGCATTGACCTGCACAACCAAGCCAGTGTCCCCCTGGAGCCTAGACCTCTTCGGAGAGAGAGTGAAATCGCTGGGAGACGCGGAATAACTGTG

kir21_Frag1149-1308_insertion1245	TGATCTAAGTGGCGTCTCCCTGTTACGAGAACGAAGTAGCTCTCACATCAAAGGAAGAAGAAGAAGATTCAGAGAACGGAGTTCCCGAGTCAACCAGCACCGACTCTCCTCCCGGCATTAGCGGGAGACCGGGGTCTCTGAGCGACCTGCACAACCAAGCCAGTGTCCCCCTGGAGCCTAGACCTCTTCGGAGAGAGAGTGAAATCGCTGGGAGACGCGGAATAACTGTG
kir21_Frag1149-1308_insertion1248	TGATCTAAGTGGCGTCTCCCTGTTACGAGAACGAAGTAGCTCTCACATCAAAGGAAGAAGAAGAAGATTCAGAGAACGGAGTTCCCGAGTCAACCAGCACCGACTCTCCTCCCGGCATTGACAGCGGGAGACCGGGGTCTCTGAGCCTGCACAACCAAGCCAGTGTCCCCCTGGAGCCTAGACCTCTTCGGAGAGAGAGTGAAATCGCTGGGAGACGCGGAATAACTGTG
kir21_Frag1149-1308_insertion1251	TGATCTAAGTGGCGTCTCCCTGTTACGAGAACGAAGTAGCTCTCACATCAAAGGAAGAAGAAGAAGATTCAGAGAACGGAGTTCCCGAGTCAACCAGCACCGACTCTCCTCCCGGCATTGACCTGAGCGGGAGACCGGGGTCTCTGAGCCACAACCAAGCCAGTGTCCCCCTGGAGCCTAGACCTCTTCGGAGAGAGAGTGAAATCGCTGGGAGACGCGGAATAACTGTG
kir21_Frag1149-1308_insertion1254	TGATCTAAGTGGCGTCTCCCTGTTACGAGAACGAAGTAGCTCTCACATCAAAGGAAGAAGAAGAAGATTCAGAGAACGGAGTTCCCGAGTCAACCAGCACCGACTCTCCTCCCGGCATTGACCTGCACAGCGGGAGACCGGGGTCTCTGAGCAACCAAGCCAGTGTCCCCCTGGAGCCTAGACCTCTTCGGAGAGAGAGTGAAATCGCTGGGAGACGCGGAATAACTGTG
kir21_Frag1149-1308_insertion1257	TGATCTAAGTGGCGTCTCCCTGTTACGAGAACGAAGTAGCTCTCACATCAAAGGAAGAAGAAGAAGATTCAGAGAACGGAGTTCCCGAGTCAACCAGCACCGACTCTCCTCCCGGCATTGACCTGCACAACAGCGGGAGACCGGGGTCTCTGAGCCAAGCCAGTGTCCCCCTGGAGCCTAGACCTCTTCGGAGAGAGAGTGAAATCGCTGGGAGACGCGGAATAACTGTG
kir21_Frag1149-1308_insertion1260	TGATCTAAGTGGCGTCTCCCTGTTACGAGAACGAAGTAGCTCTCACATCAAAGGAAGAAGAAGAAGATTCAGAGAACGGAGTTCCCGAGTCAACCAGCACCGACTCTCCTCCCGGCATTGACCTGCACAACCAAAGCGGGAGACCGGGGTCTCTGAGCGCCAGTGTCCCCCTGGAGCCTAGACCTCTTCGGAGAGAGAGTGAAATCGCTGGGAGACGCGGAATAACTGTG
kir21_Frag1149-1308_insertion1263	TGATCTAAGTGGCGTCTCCCTGTTACGAGAACGAAGTAGCTCTCACATCAAAGGAAGAAGAAGAAGATTCAGAGAACGGAGTTCCCGAGTCAACCAGCACCGACTCTCCTCCCGGCATTGACCTGCACAACCAAGCCAGCGGGAGACCGGGGTCTCTGAGCAGTGTCCCCCTGGAGCCTAGACCTCTTCGGAGAGAGAGTGAAATCGCTGGGAGACGCGGAATAACTGTG
kir21_Frag1149-1308_insertion1266	TGATCTAAGTGGCGTCTCCCTGTTACGAGAACGAAGTAGCTCTCACATCAAAGGAAGAAGAAGAAGATTCAGAGAACGGAGTTCCCGAGTCAACCAGCACCGACTCTCCTCCCGGCATTGACCTGCACAACCAAGCCAGTAGCGGGAGACCGGGGTCTCTGAGCGTCCCCCTGGAGCCTAGACCTCTTCGGAGAGAGAGTGAAATCGCTGGGAGACGCGGAATAACTGTG
kir21_Frag1149-1308_insertion1269	TGATCTAAGTGGCGTCTCCCTGTTACGAGAACGAAGTAGCTCTCACATCAAAGGAAGAAGAAGAAGATTCAGAGAACGGAGTTCCCGAGTCAACCAGCACCGACTCTCCTCCCGGCATTGACCTGCACAACCAAGCCAGTGTCAGCGGGAGACCGGGGTCTCTGAGCCCCCTGGAGCCTAGACCTCTTCGGAGAGAGAGTGAAATCGCTGGGAGACGCGGAATAACTGTG
kir21_Frag1149-1308_insertion1272	TGATCTAAGTGGCGTCTCCCTGTTACGAGAACGAAGTAGCTCTCACATCAAAGGAAGAAGAAGAAGATTCAGAGAACGGAGTTCCCGAGTCAACCAGCACCGACTCTCCTCCCGGCATTGACCTGCACAACCAAGCCAGTGTCCCCAGCGGGAGACCGGGGTCTCTGAGCCTGGAGCCTAGACCTCTTCGGAGAGAGAGTGAAATCGCTGGGAGACGCGGAATAACTGTG
kir21_Frag1149-1308_insertion1275	TGATCTAAGTGGCGTCTCCCTGTTACGAGAACGAAGTAGCTCTCACATCAAAGGAAGAAGAAGAAGATTCAGAGAACGGAGTTCCCGAGTCAACCAGCACCGACTCTCCTCCCGGCATTGACCTGCACAACCAAGCCAGTGTCCCCCTGAGCGGGAGACCGGGGTCTCTGAGCGAGCCTAGACCTCTTCGGAGAGAGAGTGAAATCGCTGGGAGACGCGGAATAACTGTG
kir21_Frag1149-1308_insertion1278	TGATCTAAGTGGCGTCTCCCTGTTACGAGAACGAAGTAGCTCTCACATCAAAGGAAGAAGAAGAAGATTCAGAGAACGGAGTTCCCGAGTCAACCAGCACCGACTCTCCTCCCGGCATTGACCTGCACAACCAAGCCAGTGTCCCCCTGGAGAGCGGGAGACCGGGGTCTCTGAGCCCTAGACCTCTTCGGAGAGAGAGTGAAATCGCTGGGAGACGCGGAATAACTGTG
kir21_Frag1149-1308_insertion1281	TGATCTAAGTGGCGTCTCCCTGTTACGAGAACGAAGTAGCTCTCACATCAAAGGAAGAAGAAGAAGATTCAGAGAACGGAGTTCCCGAGTCAACCAGCACCGACTCTCCTCCCGGCATTGACCTGCACAACCAAGCCAGTGTCCCCCTGGAGCCTAGCGGGAGACCGGGGTCTCTGAGCAGACCTCTTCGGAGAGAGAGTGAAATCGCTGGGAGACGCGGAATAACTGTG
kir21_Frag1149-1308_insertion1284	TGATCTAAGTGGCGTCTCCCTGTTACGAGAACGAAGTAGCTCTCACATCAAAGGAAGAAGAAGAAGATTCAGAGAACGGAGTTCCCGAGTCAACCAGCACCGACTCTCCTCCCGGCATTGACCTGCACAACCAAGCCAGTGTCCCCCTGGAGCCTAGAAGCGGGAGACCGGGGTCTCTGAGCCCTCTTCGGAGAGAGAGTGAAATCGCTGGGAGACGCGGAATAACTGTG
kir21_Frag1149-1308_insertion1287	TGATCTAAGTGGCGTCTCCCTGTTACGAGAACGAAGTAGCTCTCACATCAAAGGAAGAAGAAGAAGATTCAGAGAACGGAGTTCCCGAGTCAACCAGCACCGACTCTCCTCCCGGCATTGACCTGCACAACCAAGCCAGTGTCCCCCTGGAGCCTAGACCTAGCGGGAGACCGGGGTCTCTGAGCCTTCGGAGAGAGAGTGAAATCGCTGGGAGACGCGGAATAACTGTG
kir21_Frag1149-1308_insertion1290	TGATCTAAGTGGCGTCTCCCTGTTACGAGAACGAAGTAGCTCTCACATCAAAGGAAGAAGAAGAAGATTCAGAGAACGGAGTTCCCGAGTCAACCAGCACCGACTCTCCTCCCGGCATTGACCTGCACAACCAAGCCAGTGTCCCCCTGGAGCCTAGACCTCTTAGCGGGAGACCGGGGTCTCTGAGCCGGAGAGAGAGTGAAATCGCTGGGAGACGCGGAATAACTGTG
kir21_Frag1149-1308_insertion1293	TGATCTAAGTGGCGTCTCCCTGTTACGAGAACGAAGTAGCTCTCACATCAAAGGAAGAAGAAGAAGATTCAGAGAACGGAGTTCCCGAGTCAACCAGCACCGACTCTCCTCCCGGCATTGACCTGCACAACCAAGCCAGTGTCCCCCTGGAGCCTAGACCTCTTCGGAGCGGGAGACCGGGGTCTCTGAGCAGAGAGAGTGAAATCGCTGGGAGACGCGGAATAACTGTG
kir21_Frag1149-1308_insertion1296	TGATCTAAGTGGCGTCTCCCTGTTACGAGAACGAAGTAGCTCTCACATCAAAGGAAGAAGAAGAAGATTCAGAGAACGGAGTTCCCGAGTCAACCAGCACCGACTCTCCTCCCGGCATTGACCTGCACAACCAAGCCAGTGTCCCCCTGGAGCCTAGACCTCTTCGGAGAAGCGGGAGACCGGGGTCTCTGAGCGAGAGTGAAATCGCTGGGAGACGCGGAATAACTGTG
kir21_Frag1149-1308_insertion1299	TGATCTAAGTGGCGTCTCCCTGTTACGAGAACGAAGTAGCTCTCACATCAAAGGAAGAAGAAGAAGATTCAGAGAACGGAGTTCCCGAGTCAACCAGCACCGACTCTCCTCCCGGCATTGACCTGCACAACCAAGCCAGTGTCCCCCTGGAGCCTAGACCTCTTCGGAGAGAGAGCGGGAGACCGGGGTCTCTGAGCAGTGAAATCGCTGGGAGACGCGGAATAACTGTG
kir21_Frag1149-1308_insertion1302	TGATCTAAGTGGCGTCTCCCTGTTACGAGAACGAAGTAGCTCTCACATCAAAGGAAGAAGAAGAAGATTCAGAGAACGGAGTTCCCGAGTCAACCAGCACCGACTCTCCTCCCGGCATTGACCTGCACAACCAAGCCAGTGTCCCCCTGGAGCCTAGACCTCTTCGGAGAGAGAGTAGCGGGAGACCGGGGTCTCTGAGCGAAATCGCTGGGAGACGCGGAATAACTGTG
kir21_Frag1149-1308_insertion1305	TGATCTAAGTGGCGTCTCCCTGTTACGAGAACGAAGTAGCTCTCACATCAAAGGAAGAAGAAGAAGATTCAGAGAACGGAGTTCCCGAGTCAACCAGCACCGACTCTCCTCCCGGCATTGACCTGCACAACCAAGCCAGTGTCCCCCTGGAGCCTAGACCTCTTCGGAGAGAGAGTGAAAGCGGGAGACCGGGGTCTCTGAGCATCGCTGGGAGACGCGGAATAACTGTG
kir21_Frag1149-1308_insertion1308	TGATCTAAGTGGCGTCTCCCTGTTACGAGAACGAAGTAGCTCTCACATCAAAGGAAGAAGAAGAAGATTCAGAGAACGGAGTTCCCGAGTCAACCAGCACCGACTCTCCTCCCGGCATTGACCTGCACAACCAAGCCAGTGTCCCCCTGGAGCCTAGACCTCTTCGGAGAGAGAGTGAAATCAGCGGGAGACCGGGGTCTCTGAGCGCTGGGAGACGCGGAATAACTGTG
